# Genome mining reveals the genus *Xanthomonas* to be a promising reservoir for new bioactive non-ribosomally synthesized peptides

**DOI:** 10.1186/1471-2164-14-658

**Published:** 2013-09-27

**Authors:** Monique Royer, Ralf Koebnik, Mélanie Marguerettaz, Valérie Barbe, Guillaume P Robin, Chrystelle Brin, Sébastien Carrere, Camila Gomez, Manuela Hügelland, Ginka H Völler, Julie Noëll, Isabelle Pieretti, Saskia Rausch, Valérie Verdier, Stéphane Poussier, Philippe Rott, Roderich D Süssmuth, Stéphane Cociancich

**Affiliations:** 1 CIRAD, UMR BGPI, Montpellier Cedex 5, F-34398, France; 2 IRD, UMR RPB, Montpellier Cedex F-34394, France; 3 CEA/DSV/IG/Genoscope, Centre National de Séquençage, Evry Cedex F-91057, France; 4 INRA, UMR IRHS, Beaucouzé F-49071, France; 5 INRA, UMR LIPM, Castanet-Tolosan Cedex F-31326, France; 6 Institut für Chemie, Technische Universität Berlin, Berlin D-10623, Germany; 7 UMR PVBMT, Université de la Réunion, Saint-Denis, La Réunion F-97715, France

## Abstract

**Background:**

Various bacteria can use non-ribosomal peptide synthesis (NRPS) to produce peptides or other small molecules. Conserved features within the NRPS machinery allow the type, and sometimes even the structure, of the synthesized polypeptide to be predicted. Thus, bacterial genome mining *via in silico* analyses of NRPS genes offers an attractive opportunity to uncover new bioactive non-ribosomally synthesized peptides. *Xanthomonas* is a large genus of Gram-negative bacteria that cause disease in hundreds of plant species. To date, the only known small molecule synthesized by NRPS in this genus is albicidin produced by *Xanthomonas albilineans*. This study aims to estimate the biosynthetic potential of *Xanthomonas* spp. by *in silico* analyses of NRPS genes with unknown function recently identified in the sequenced genomes of *X. albilineans* and related species of *Xanthomonas*.

**Results:**

We performed *in silico* analyses of NRPS genes present in all published genome sequences of *Xanthomonas* spp., as well as in unpublished draft genome sequences of *Xanthomonas oryzae* pv*. oryzae* strain BAI3 and *Xanthomonas* spp. strain XaS3. These two latter strains, together with *X. albilineans* strain GPE PC73 and *X. oryzae* pv. *oryzae* strains X8-1A and X11-5A, possess novel NRPS gene clusters and share related NRPS-associated genes such as those required for the biosynthesis of non-proteinogenic amino acids or the secretion of peptides. *In silico* prediction of peptide structures according to NRPS architecture suggests eight different peptides, each specific to its producing strain. Interestingly, these eight peptides cannot be assigned to any known gene cluster or related to known compounds from natural product databases. PCR screening of a collection of 94 plant pathogenic bacteria indicates that these novel NRPS gene clusters are specific to the genus *Xanthomonas* and are also present in *Xanthomonas translucens* and *X. oryzae* pv. *oryzicola*. Further genome mining revealed other novel NRPS genes specific to *X. oryzae* pv. *oryzicola* or *Xanthomonas sacchari*.

**Conclusions:**

This study revealed the significant potential of the genus *Xanthomonas* to produce new non-ribosomally synthesized peptides. Interestingly, this biosynthetic potential seems to be specific to strains of *Xanthomonas* associated with monocotyledonous plants, suggesting a putative involvement of non-ribosomally synthesized peptides in plant-bacteria interactions.

## Background

Various bacteria, most prominently belonging to the orders *Bacillales*, *Pseudomonadales* or *Actinomycetales*, use non-ribosomal peptide synthesis (NRPS) to produce peptides or other small molecules. These molecules exhibit broad structural diversity and display biological activities that range from adaptation to unfavorable environments, communication or competition with other microorganisms in their natural habitat, or even to action as virulence factors (for review [1,2]). Non-ribosomal peptide synthetases (NRPSs) are megaenzymes, usually with a multimodular structure, that catalyze the non-ribosomal assembly of peptides from proteinogenic and non-proteinogenic amino acids [3,4]. A basic module consists of an adenylation domain (A-domain) responsible for amino acid activation; a peptidyl carrier protein domain (PCP-domain)—usually adjacent to the A-domain—for thioesterification of the activated amino acid; a condensation domain (C-domain), which performs transpeptidation between the upstream and downstream peptidyl and amino acyl thioesters to elongate the growing peptide chain. In addition to this basic subset of core domains, each NRPS system also has a chain-terminating thioesterase domain (TE-domain) that is responsible for detachment of the mature polypeptide. Typically, NRPS initiation modules lack a C-domain [3]. The exceptions are initiation modules of NRPS involved in biosynthesis of cyclic lipopeptides [5]. C-domains present in these initiation modules catalyze *N*-acylation of the first amino acid with a fatty acid (with a β-hydroxy-carboxylic acid). Phylogenetic analyses of C-domains in NRPSs showed that C-domains of initiation modules, termed starter C-domains, segregate in a separate phylogenetic clade distant from the C-domains of elongation NRPS modules [6]. In addition to the essential domains mentioned above (A, PCP, C and TE), optional auxiliary domains such as epimerization (E) or heterocyclization (Cy) domains can be found within some NRPS modules. Commonly, epimerization domains are located C-terminally of PCP-domains and perform epimerization of the last amino acid of the adjacent peptidyl-thioester. Interestingly, biochemical data showed that C-domains involved in assembly of arthrofactin not only catalyze transpeptidation between the upstream peptidyl thioester and the downstream amino acyl thioester, but also catalyze epimerization of the last amino acid from the upstream peptidyl thioester from l into d configuration [7]. These C-domains have been termed dual C/E domains [7]. Phylogenetic analyses of C-domains in NRPSs also showed that dual C/E domains segregate in a separate phylogenetic clade [6]. Because of their wide structural complexity and diversity, natural products synthesized by NRPSs constitute a nearly inexhaustible source of new small molecules that might yield lead compounds in drug discovery [8].

The A-domains are the gatekeepers of biosynthesis of the polypeptide due to the specificity of substrate binding pockets of NRPSs for their cognate substrates. Compared to the 22 proteinogenic amino acids used in ribosomal protein synthesis, the utilization of hundreds of different non-proteinogenic amino acids has been described for NRPS. Each substrate binding pocket of NRPSs is specific for its amino acid substrate, and predictive models based on domain arrangement and on the sequence of modules have been deduced. The predictive power of these models has been refined from sequence analyses of NRPS A-domains with known specificity combined with examples of crystal structures of A-domains [9-11], thus identifying amino acid residues crucial for A-domain specificity. These models postulate specificity-conferring signatures for NRPS A-domains consisting of ten critical amino acid residues putatively involved in amino acid or aryl acid substrate specificity. The number of NRPS modules and their domain organization within the enzymes determine the structure of the final peptide product. Using *in silico* analyses of the NRPS genes, these conserved features within the NRPS machinery allow prediction of the type, and sometimes even the structure, of the synthesized polypeptide. It is therefore possible to investigate the biosynthetic potential of a given bacterium by analysis of the architecture of its NRPS gene clusters.

The transfer of a phosphopantetheinyl group to PCP-domains is required for posttranslational activation of NRPSs. Inactive apo-NRPSs are converted to their active holo-forms by transfer of the 4′-phosphopantetheinyl (P-pant) moiety of coenzyme A to the sidechain hydroxyl of a serine residue in the PCP-domains. The P-pant moiety serves to tether covalently the growing polypeptide being assembled by NRPSs. Transfer of the P-pant moiety from coenzyme A to a serine residue is catalyzed by 4′-phosphopantetheinyl transferase (PPTase). PPTases are involved not only in posttranslational activation of NRPS but also in posttranslational activation of fatty acid and polyketide synthases that do not contain PCP-domains but instead have acyl carrier protein (ACP) domains. All PPTases are recognized by a common signature sequence (V/I)G(I/V)D …x40-45… (F/W)(S/C/T)xKE(S/A)xxK, but overall sequence similarities are low [12-15]. Carrier protein specificity (ACP or PCP) has been determined experimentally for some PPTases. For example, in *Escherichia coli*, the EntD PPTase involved in biosynthesis of the siderophore enterobactin was shown experimentally to be active only on PCP-domains and not on ACP-domains [12,14].

The *Xanthomonadaceae* are a family of Gram-negative bacteria belonging to the order *Xanthomonadales* in the gamma subdivision of the *Proteobacteria*[16]. Members of this family are typically characterized as environmental organisms, and occupy diverse ecological niches such as soil and water, as well as plant tissues. Many *Xanthomonadaceae*, especially species from the genera *Xanthomonas* and *Xylella*, cause plant diseases, and only one, *Stenotrophomonas maltophilia*, is known to be an opportunistic human pathogen. The genus *Xanthomonas* consists of 27 plant-associated species, many of which cause important diseases of crops or ornamental plants. Individual species comprise multiple pathovars, characterized by distinctive host specificity or mode of infection. Collectively, members of this genus cause diseases on at least 120 monocotyledonous and 260 dicotyledonous crop plants.

*Xanthomonas albilineans* is a systemic, xylem-invading pathogen that causes leaf scald—a lethal disease of sugarcane (interspecific hybrids of *Saccharum* spp.) [17]. Leaf scald symptoms vary from a single, white, narrow, sharply defined stripe to complete wilting and necrosis of infected leaves, leading to plant death. The only pathogenicity factor of *X. albilineans* to be extensively studied to date is albicidin—a secreted non-ribosomally synthesized peptide with phytotoxic and antibiotic properties [18]. Albicidin is a potent DNA gyrase inhibitor with a novel mode of action [19]. Albicidin targets chloroplastic DNA gyrase A, inhibits chloroplast DNA replication and blocks chloroplast differentiation, resulting in the white foliar stripe symptoms [18]. Albicidin also targets bacterial DNA gyrase A and, as a consequence, exhibits a potent antibiotic activity against a wide range of Gram-positive and Gram-negative bacteria [20]. This antibiotic activity may help *X. albilineans* to combat rival microorganisms during sugarcane invasion. The complete albicidin biosynthesis gene cluster, called XALB1, was cloned and sequenced from *X. albilineans* strain Xa23R1 [21]. XALB1 encodes three large NRPS genes and also resistance, regulatory and tailoring genes [21-24]. A PPTase gene, annotated as *xabA* or *albXXI*, was shown to be required for albicidin biosynthesis in *X. albilineans* strains LS155 and Xa23R1, respectively [21,25]. The *E. coli entD* gene restored the biosynthesis of albicidin in a *xabA* knockout mutant of *X. albilineans* strain LS155 [25], demonstrating that *xabA* (or *albXXI*) has the same PPTase activity as *entD*, which is active only on PCP-domains. Preliminary analyses by nuclear magnetic resonance (NMR) spectroscopy and mass spectrometry (MS) did not allow the determination of the structure of albicidin [20,26]. However, these studies showed that albicidin contains about 38 carbon atoms and has an estimated molecular mass of 842 Da. *In silico* analyses of XALB1 provided further insights into the structure of this pathotoxin, and suggested that NRPSs involved in biosynthesis of albicidin incorporate as yet unknown non-proteinogenic substrates [21]. Thus, the accumulated data obtained for albicidin and its biosynthesis gene cluster suggest that it is a potent DNA gyrase inhibitor with a novel mode of action, and therefore might constitute a lead structure for novel antibiotics.

The genome of *X. albilineans* strain GPE PC73 was recently sequenced [27]. This genome contains, in addition to three NRPS genes encoding albicidin biosynthesis [21], seven novel NRPS genes that share similarities with NRPS genes present in the genomes of two recently sequenced American *Xanthomonas oryzae* pv. *oryzae* strains [28]. These novel NRPS genes cannot be assigned to any structure or function since there are no orthologous genes in other bacteria. Interestingly, *X. oryzae* possesses an ortholog of the PPTase gene *xabA* (or *albXXI*) which is hereafter referred to as XaPPTase because of its occurence in two species of *Xanthomonas*.

*X. oryzae* pathovars are the causal agents of two important diseases of rice: bacterial leaf blight caused by *X. oryzae* pv. *oryzae*, and bacterial leaf streak caused by *X. oryzae* pv. *oryzicola*. The XaPPTase gene is found in all six genome sequences of *X. oryzae* published to date (the completed genome sequences of the three Asian *X. oryzae* pv. *oryzae* strains MAFF 311018, KACC10331 and PXO99A [29-31]; the non annotated draft sequences of the genomes of the two American *X. oryzae* pv. *oryzae* strains X11-5A and X8-1A [28]; and the completed sequence of the genome of *X. oryzae* pv. *oryzicola* strain BLS256 [32]). The presence of the XaPPTase gene in all six genomes indicates that all strains of *X. oryzae* likely possess NRPS genes and produce non-ribosomally synthesized peptides.

The objective of this study was to estimate the biosynthetic potential of *X. albilineans* and *X. oryzae* by *in silico* analyses of their uncharacterized NRPS gene clusters and to identify other bacterial candidates for extended genome mining by PCR screening of a collection of 94 plant pathogenic bacteria for the presence of the XaPPTase gene and other NRPS-associated genes. To complete available published genomic data, we included in our analyses unpublished draft genome sequences of the African *X. oryzae* pv*. oryzae* strain BAI3 isolated in Burkina Faso [33] and the *Xanthomonas* spp. strain XaS3 isolated in Guadeloupe (French Caribbean island). To date, no genome sequence of an African *X. oryzae* pv*. oryzae* strain is available in public databases but a draft sequence of the genome of African strain AXO1947 of *X. oryzae* pv. *oryzae* was used recently to identify candidate type III secretion system effector genes [34]. *Xanthomonas* spp. strain XaS3 was isolated from the surface of a sugarcane leaf and was characterized initially as a member of the species *X. albilineans*[35]. However, recent MLSA (multi locus sequence analyses) analyses showed that this strain, which does not possess the albicidin biosynthesis gene cluster, is close to *X. albilineans* but belongs to a separate phylogenetic clade (I. Pieretti, unpublished data).

## Results and discussion

### Features of the XaPPTase genes in *X. albilineans* and *X. oryzae*

**Figure 1:**
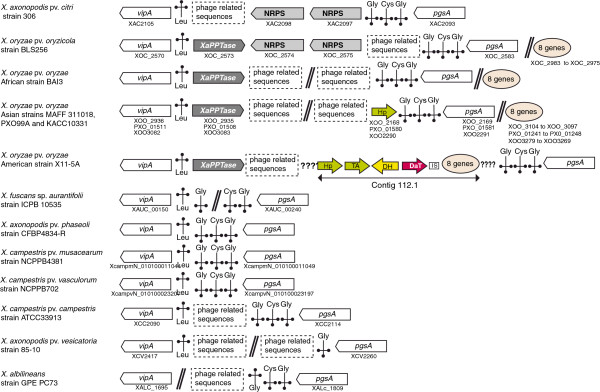
**Physical map of the genomic region containing the XaPPTase gene in *Xanthomonas oryzae *strains and of the corresponding region in other sequenced species of *Xanthomonas*.** White arrows: gene *vipA* (probable UDP-glucose/GDP-mannose dehydrogenase gene) and gene *pgsA* (probable CDP-diacylglycerol-glycerol-3-phosphate-3-phosphatidyltransferase gene). Dark grey arrows: XaPPTase gene (encoding a 4′-phosphopantetheinyl transferase active on peptidyl carrier protein domains). Light grey arrows: NRPS (nonribosomal peptide synthesis) genes. Coloured arrows correspond to genes described in Figures 3 and 4. Hp: hypothetical protein gene (ortholog of accession Dd703_3065 of strain Ech703 of *Dickeya dadantii*). TA: transaminase gene (ortholog of accession Dd703_3064 of strain Ech703 of *D. dadantii*). DH: lactate dehydrogenase gene. DaT: gene involved in biosynthesis of 2,4-diaminobutyric acid (Dab). Length of arrows is not proportional to the length of genes. Salmon-coloured oval circle: Eight genes present in contig 112.1 of *X. oryzae* strain X11-5A that are conserved in other strains of *X. oryzae* and are not predicted to be involved in NRPS biosynthesis. : tRNA. Orientation of the tag indicates the orientation of the tRNA gene in the genomic regions. Amino acid specificity of each tRNA is indicated above or below each tag according to the orientation of the tRNA gene. ???? : undetermined sequence located between contigs. Two contigs separated by this tag may be located in two different genomic regions (they are not necessarily contiguous).  : this tag indicates that corresponding genomic regions are not contiguous. Data on strain CFBP4834-R of *X. axonopodis* pv. *phaseoli* were obtained from an unpublished finished genome sequence (M.-A. Jacques, personal communication).

In strains LS155, Xa23R1 and GPE PC73 of *X. albilineans*, as well as in the *Xanthomonas* spp. strain XaS3, the XaPPTase gene is located between the *rpsF* gene encoding the 30S ribosomal protein S6 (XALc_1735) and a gene encoding an iron-sulfur cluster assembly protein (XALc_1737). These latter two genes are conserved and contiguous in other sequenced species of *Xanthomonas*. In all sequenced strains of *X. oryzae*, including strain BAI3, the XaPPTase gene is located in a region containing several tRNA genes and phage-related sequences (Figure 1). In strain BLS256 of *X. oryzae* pv. *oryzicola*, this region also contains two large NRPS genes that are partially conserved and located in the same region in strain 306 of *Xanthomonas axonopodis* pv. *citri* (Figure 1). However, the XaPPTase gene is not conserved in strain 306*.* The region between the tRNA genes does not contain any genes in several other species of *Xanthomonas* (Figure 1), suggesting that XaPPTase and/or NRPS genes were acquired by lateral gene transfer.

The XaPPTase genes from *X. albilineans* and *X. oryzae*, which are located in two different genomic regions, share only 51% amino acid similarity, although their PPTase signature sequences are very similar (Table 1). The reciprocal best BLAST hit in GenBank for the XaPPTase gene of *X. albilineans* is the XaPPTase gene from *X. oryzae*. This indicates that these genes might have been derived from a common ancestor. Signature sequences of all XaPPTase genes are very similar to the signature sequence of *entD* gene of *E. coli* (Table 1). *X. albilineans* and *X. oryzae* possess another PPTase gene, *hetI*, which is conserved in all sequenced species of *Xanthomonas*. Signature sequences of *hetI* of *X. albilineans* and *X. oryzae* are both similar to the signature sequence of a PPTase of *Mycobacterium tuberculosis* that was shown experimentally to be active only on ACP-domains of fatty acid and polyketide synthases and not on PCP-domains [15] (Table 1).

**Table 1:** **Similarity between XaPPTase and other PPTases involved in NRPS and fatty acid biosynthesis in bacteria (from **[25]**)**

**Pathways**	** Proteins**	** Organisms **^**a**^	**Experimentally-determined specificities (A/P)**^**b**^	** Domain I**	**Spacing between domains I and II (in aa)**	** Domain II**	**Overall amino acid identities / similarities with XabA**
Albicidin and unknown	XabA	*X. albilineans* str. LS155	P^c^	G**VGID**LERP	--(x)39--	**FS**A**KES**LF**K**AAY	-
	AlbXXI	*X. albilineans* str. Xa23R1	?	G**VGID**LERP	--(x)39--	**FS**A**KES**LF**K**AAY	100% /-
	XaPPTase	*X. albilineans* str. GPE PC73	?	G**VGID**LERP	--(x)39--	**FS**A**KES**LF**K**AAY	94% / 95%
	XaPPTase	*Xanthomonas spp.* str. XaS3	?	G**VGID**LERM	--(x)39--	**FS**A**KES**LF**K**AAY	83% / 87%
Unknown	XaPPTase	All sequenced *X. oryzae* strains*	?	G**IGID**LEHL	--(x)38--	**FS**A**KES**LF**K**ASF	40% / 51%
Unknown	XaPPTase	*X. sacchari* str. NCPPB4393	?	G**IG**L**D**VERV	--(x)38--	**FS**A**KES**FY**K**AAA	41% / 55%
Unknown	BBta_3710	*Bradyrhizobium* spp. BTAi	?	AL**G**L**D**IEDV	--(x)35--	**FS**A**KEA**YY**K**CQY	25% / 36%
Enterobactin	EntD	*E. coli* str. K12 substr. MG1655	P^d^	P**IGID**IEEI	--(x)36--	**FS**A**KES**AF**K**ASE	23% / 31%
Mycobactin	PptT	*M. tuberculosis* str. CSU93	P^e^	S**VGID**AEPH	--(x)35--	**FC**A**KEA**TY**K**AWF	25% / 34%
Gramicidin	Gsp	*Bacillus brevis* str. ATCC 9999	P^d^	P**VGID**IERI	--(x)35--	**WT**I**KES**YI**K**AIG	14% / 21%
Surfactin	Sfp	*Bacillus subtilis* str. RB14	A/P^f^	P**IGID**IEKT	--(x)35--	**WS**M**KES**FI**K**QEG	17% / 25%
Fatty acids	AcpS	*E. coli* str. K12 substr. MG1655	A^d^	GL**G**T**D**IVEI	--(x)40--	**F**AV**KEA**AA**K**AFG	9% / 14%
Fatty acids	AcpT	*E. coli* str. K12 substr. MG1655	?	E**VG**C**D**IEVI	--(x)34--	**WT**R**KEA**IV**K**QRG	13% / 22%
Fatty acids	AcpS	*M. tuberculosis* str. CSU93	A^e^	G**VGID**LVSI	--(x)41--	**W**AA**KEA**VI**K**AWS	11% / 17%
Unknown	HetI	*X. albilineans* str. GPE PC73	?	RL**GVD**IERQ	--(x)37--	**WC**A**KEA**LL**K**AHG	25% / 31%
Unknown	HetI	*Xanthomonas spp.* str. XaS3	?	RL**GVD**IERQ	--(x)37--	**WC**A**KEA**LL**K**AHG	22% / 28%
Unknown	HetI	All sequenced *X. oryzae* strains	?	RL**GVD**LERI	--(x)37 --	**WC**A**KEA**LL**K**AYG	20% / 25%
Consensus				(V/I)G(I/V)D**		(F/W)(S/C/TxKE(S/A)xxK**	

^a^ str. = strain; substr. = substrain.

^b^ A, specific for acyl carrier protein (ACP) domains; P, specific for peptidyl carrier protein (PCP) domains.

^c^[25].

^d^[12].

^e^[15].

^f^[36].

*: XaPPTase gene from strain PXO99A was mis-annotated (correct start codon is upstream of the one deposited in GenBank).

**: Consensus signature sequences of PPTase domains as defined by [14,15].

XaPPTase proteins of strains LS155 and Xa23R1 of *X. albilineans* are 100% identical, but they are only 94% and 83% identical to XaPPTase proteins of strains GPE PC73 and XaS3, respectively. However, XaPPTase signatures are 100% identical in strains LS155, Xa23R1 and GPE PC73, differing only in one amino acid residue in strain XaS3 (Table 1).

### Genome mining reveals three novel NRPS loci in the genome of *X. albilineans* strain GPE PC73

In addition to the three NRPS genes characterized previously as being involved in the biosynthesis of albicidin [21], the chromosome of strain GPE PC73 possesses seven large multimodular NRPS genes clustered in three loci, hereafter referred to as META-A, META-B and META-C, respectively. These non-characterized NRPS genes share no similarity with NRPS genes from the biosynthesis gene cluster of albicidin.

**Figure 2:**
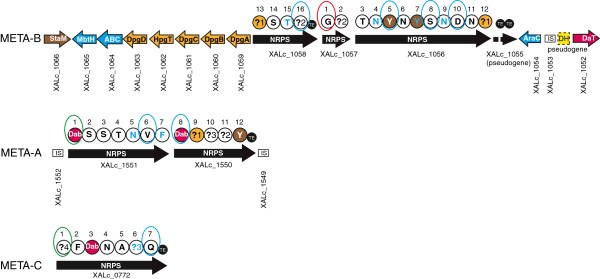
**Representation of the NRPS loci META-A, META-B and META-C of *X. albilineans *strain GPE PC73.** Brown arrow: *staM* gene predicted to be required for β−hydroxylation of an unidentified amino acid. Purple arrow: *daT* gene predicted to be required for biosynthesis of Dab (2,4-diamino butyric acid). Orange arrows: genes predicted to be required for biosynthesis of Dpg (3,5-dihydroxyphenylglycine). Dark blue arrows: other genes conserved in all META-B loci (ABC transporter, MbtH-like protein and transcriptional regulator AraC). Black arrows: NRPS genes. Length of arrows is not proportional to the length of genes. NRPS modules are represented by circles. Large circles indicate complete NRPS modules (containing domains C, A and PCP). The amino acid predicted to be assembled by the corresponding module is indicated within each circle. ?X: unknown amino acid specific to the NRPS signature UnknownX (Additional file 1). Brown circles: NRPS modules predicted to be specific for tyrosine. Purple circles: NRPS modules predicted to be specific to Dab. Orange circles: NRPS modules predicted to be specific to Dpg. The sequential order of amino acid incorporation from the N- to the C-terminus is illustrated by the number above each circle. Small black circles represent TE domains. The starter module circled in red exhibits a C-domain belonging to the same phylogenetic clade as starter C-domains identified by Rausch et al. [6] (Additional file 2). Starter modules circled in green indicate a C-domain belonging to the distant phylogenetic clade containing starter C-domains of loci META-A and META-C (Additional file 2). Elongation modules circled in blue indicate a C-domain belonging to the same phylogenetic clade as dual C/E domains identified by Rausch et al. [6] (Additional file 2). Amino acids predicted to be epimerized by these domains are in blue. IS: insertion sequence. Dotted box: pseudogene.

Interestingly, the loci META-A and META-C encode NRPSs only, and no NRPS-associated proteins, *e.g.* tailoring enzymes, in the proximity of these gene clusters. META-A is adjacent to the albicidin NRPS gene cluster XALB1, from which it is separated by the terminus of replication. META-A, which is flanked by IS elements, includes two large NRPS genes: XALc_1551 (22,629 bp) and XALc_1550 (16,965 bp). These two genes encode a NRPS system consisting of 12 modules each containing the characteristic domain arrangement C-A-PCP. A TE-domain is located at the C-terminus of XALc_1550 (Figure 2; Additional file 1). META-C, which is not flanked by IS elements, contains a single NRPS gene, XALc_0772 (23,289 bp). This gene encodes a NRPS system consisting of seven modules with a C-A-PCP domain arrangement, followed by a chain-terminating TE-domain (Figure 2; Additional file 1). Phylogenetic analysis of C-domains revealed that the C-domains of the first modules of XALc_1551 and XALc_0772 cluster in a separate clade, distinct from that of the starter C-domains identified by Rausch et al. [6] in numerous bacteria (Additional file 2). This indicates that these two starter C-domains, which share 81% amino acid similarity, are unusual. The reciprocal best BLAST hit in GenBank for both starter C-domains of META-A and META-C is the N-terminal C-domain of a NRPS gene belonging to the plant-associated bacterium *Bradyrhizobium* spp. strain BTAi [37].

The META-B biosynthesis gene cluster of strain GPE PC73 contains four NRPS genes: XALc_1058 (13,686 bp), XALc_1057 (6,378 bp), XALc_1056 (32,124 bp) and XALc_1055 (1,824 bp) (Figure 2; Additional file 1). These four genes encode a NRPS system consisting of a total of 16 modules with a continuous C-A-PCP domain arrangement. Two TE-domains were identified in META-B: (i) a single TE-domain at the C-terminus of the NRPS XALc_1058, and (ii) one double TE-domain on its own encoded by a separate gene (XALc_1055) (Figure 2). Like the domain arrangement of META-A and META-C, only essential elongation domains (A, PCP, C and TE) were identified in META-B. Phylogenetic analysis showed that the C-domain of the first module of XALc_1057 belongs to the same clade as starter C-domains identified by Rausch et al. [6], indicating that it also corresponds to a starter C-domain (Additional file 2). Therefore, the first module of XALc_1057 was assigned as the initiation module of META-B (Figure 2). The last module of XALc_1058, which harbors a TE domain, was assigned as the termination module (Figure 2). XALc_1055 is the only gene available in GenBank to encode a ‘stand-alone’ double TE domain, indicating that this gene is not associated to any NRPS system and should be considered as a pseudogene.

In addition to these four NRPS genes, the META-B locus encodes an ABC transporter (XALc_1064), an AraC transcriptional regulator (XALc_1054) and a MbtH-like protein (XALc_1065). The ABC transporter likely is the corresponding transporter for secretion of small molecule(s) synthesized by META-B. The AraC protein may be required for specific transcriptional regulation of genes present in META-B. Finally, the MbtH-like protein shares similarities with proteins involved in biosyntheses of non-ribosomal peptides recently described in other bacteria [38,39]. Some genes in the META-B gene cluster are predicted to be required for the biosyntheses of three non-proteinogenic amino acids: an unidentified β-hydroxy-amino acid, 2,4-diamino butyric acid (Dab), and 3,5-dihydroxyphenyl-glycine (Dpg). XALc_1066 shares 52% amino acid similarity with *staM*, which was assigned in *Streptomyces toyocaensis* as the gene responsible for β-hydroxylation of amino acids, preferably tyrosine [40]. The protein encoded by XALc_1052 is 52% similar to a diaminobutyrate transaminase characterized in *Acinetobacter baumannii* as catalyzing the conversion of aspartate semialdehyde (an intermediate in the biosynthesis of various amino acids) to Dab [41]. Genes predicted to be required for biosynthesis of Dpg share high similarities with genes previously characterized in *Amycolatopsis balhimycina*[42] and *S. toyocaensis*[40], respectively (Table 2). Based on these similarities, XALc_1059 is predicted to encode a polyketide synthase (DpgA) that may generate 3,5-dihydroxyphenylacetyl-CoA from four molecules of malonyl-CoA, with the assistance of XALc_1060 and XALc_1063 (which encode proteins DpgB and DpgD, respectively, and which may exhibit dehydratase activity). XALc_1061 is predicted to encode dioxygenase DpgC, which may convert 3,5-dihydroxyphenylacetyl-CoA into 3,5-dihydroxyphenyl-glyoxylate, and subsequently generate Dpg after transamination by XALc_1062 (HpgT, hydroxyphenyl-glycine transaminase).

**Table 2:** **Similarities between genes predicted to be required for biosynthesis of Dpg in strain GPE PC73 of *Xanthomonas albilineans *and genes previously characterized from strain DSM 5908 of *Amycolatopsis balhimycina ***[42]**and from strain NRRL15009 of *Streptomyces toyocaensis ***[40]

**Genes of strain GPE PC73**	**Genes of strain DSM 5908 of *A. balhimycina***	**Overall amino acid identities / similarities with genes of strain DSM 5908**	**Genes of strain NRRL15009 of *S. toyocaensis***	**Overall amino acid identities / similarities with genes of strain NRRL15009**	**Functions**
XALc_1059	DpgA (CAC48378)	55% / 69%	DpgA (AAM80548)	54% / 67%	Type III chalcone synthase, generates 3,5-dihydroxyphenylacetyl-CoA from four malonyl-CoA
XALc_1060	DpgB (CAC48379)	31% / 46%	DpgB (AAM80547)	29% / 45%	Belongs to crotonase/Enoyl-CoA hydratase superfamily, enhances DpgA activity
XALc_1061	DpgC (CAC48380)	48% / 62%	DpgC (AAM80546)	48% / 62%	Metal- and cofactor-free 3,5-dihydroxyphenylacetyl-CoA 1,2-dioxygenase, converts 3,5-dihydroxyphenylacetyl-CoA into 3,5-dihydroxyphenylglyoxylate*
XALc_1062	HpgT (CAC48367)	43% / 58%	HpgT (AAM80549)	42% / 58%	Transaminase, generates 3,5-dihydroxyphenyl-glycine from 3,5-dihydroxyphenylglyoxylate
XALc_1063	DpgD (CAC48381)	62% / 74%	DpgD (AAM80545)	61% / 74%	Belongs to crotonase/Enoyl-CoA hydratase superfamily, enhances DpgA activity

* This function was characterized for biosynthesis of vancomycin [43].

The identity of several amino acids recognized and activated by the NRPSs of META-A, META-B or META-C is predicted on the basis of the analysis of specificity-conferring signatures of A-domains (Figure 2; Additional file 1). Interestingly, signatures specific to Dab were identified in both loci META-A and META-C, but remarkably not in the locus of META-B, which encodes the gene required for biosynthesis of Dab (XALc_1052). This finding suggests the occurrence of cross-talk between the three NRPS loci. Four unknown substrate specificity-conferring signatures were identified in the NRPSs of META-A, META-B or META-C (Unknown1 to Unknown4). Phylogenetic analysis of C-domains also showed that C-domains of two modules of META-A, four modules of META-B and one module of META-C segregate in the same clade as dual C/E domains identified by Rausch et al. [6] in numerous bacteria (Additional file 2). By analogy to previously identified dual C/E domains from arthrofactin NRPS, these latter dual C/E domains are predicted to follow modules that assemble amino acids with d-configuration (Figure 2; Additional file 1).

### Genome mining reveals short NRPS genes in the genome of strains GPE PC73 and XaS3

Two short NRPS genes are also present on the chromosome of strain GPE PC73 in two additional loci. These two short NRPS genes, XALc_0364 (4,047 bp) and XALc_1145 (4,011 bp), each encode only one NRPS module, with a C-A-PCP-TE domain arrangement, and overlap at their 3′ end with a glycosyltransferase gene (overlapping sequence is 4 bp and 8 bp in length for XALc_0364 and XALc_1145, respectively). XALc_0364 and XALc_1145 share 66% amino acid similarity but do not share the same substrate-specificity conferring signature (Additional file 3). XALc_0364 harbors a signature specific to Gly, and XALc_1145 harbors an unknown signature that differs from unknown signatures identified in other NRPS loci of strain GPE PC73. Their respective overlapping glycosyltransferase genes (XALc_0365 and XALc_1144) share 62% amino acid similarity (Additional file 3). The C-domains of these two short NRPS genes segregate in the same phylogenetic clade as starter C-domains identified by Rausch et al. [6]. Genes similar to the short NRPS genes of strain GPE PC73, *i.e.* encoding only one NRPS module with a C-A-PCP-TE domain arrangement and overlapping a glycosyltransferase gene at their 3′-end, were found in the genome of the marine hydrocarbonoclastic bacterium *Alcanivorax borkumensis* strain SK2 (Additional file 3). Interestingly, in this strain, a XaPPTase-like gene (ABO_1782) is contiguous with the glycosyltransferase gene (ABO_1783) and the short NRPS gene (ABO_1784), confirming that a PPTase specific to PCP-domains is required for the unknown function of these unusually small NRPS systems. Similar short NRPS genes were found in the genome of two other species of *Xanthomonas*, which nevertheless do not possess the XaPPTase gene (*X. campestris* pv. *campestris* and *X. axonopodis* pv. *citri*; Additional file 3). This indicates that these genes were present in the common ancestor of *Xanthomonas* but may have conserved their function only in species that conserved the XaPPTase gene. Short NRPS genes were not found in any sequenced strains of *X. oryzae*, including strain BAI3. However, orthologs of these genes were found in *Xanthomonas* spp. strain XaS3. The only short NRPS gene present in strain XaS3 shares 94% and 67% amino acid similarity with the two short NRPS genes XALc_0364 and XALc_1145 of strain GPE PC73, respectively, and exhibits the same Gly-specificity conferring signature as in XALc_0364 (Additional file 3). The overlapping glycosyltransferase gene of the short NRPS gene of strain XaS3 shares 92% and 62% amino acid similarity with the two glycosyltransferase genes XALc_0365 and XALc_1144, respectively, of strain GPE PC73 (Additional file 3).

### Genome mining uncovers a META-B-like NRPS gene cluster in strains X11-5A, BAI3 and XaS3

**Figure 3:**
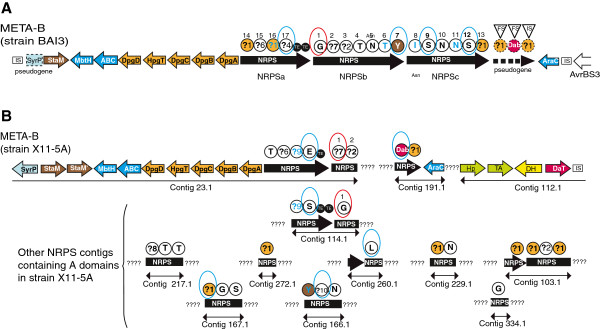
**Representation of NRPS sequences of strains BAI3 and X11-5A of *X. oryzae *pv. *oryzae. *A.** Representation of the NRPS locus META-B of *X. oryzae* pv. *oryzae* strain BAI3. **B.** Representation of contigs of *X. oryzae* pv. *oryzae* strain X11-5A that contain a nucleotide sequence encoding at least one A domain and/or a nucleotide sequence encoding non-NRPS genes similar to genes found in the locus META-B of strain *X. albilineans* GPE PC73. ???? : undetermined sequence located between contigs. Two contigs separated by this tag may be located in two different genomic regions (they are not necessarily contiguous). Brown arrows: *staM* and *staM’* genes predicted to be required for β-hydroxylation of two unidentified amino acids, respectively. Purple arrow: *daT* gene predicted to be required for biosynthesis of Dab (2,4-diamino butyric acid). Orange arrows: genes predicted to be required for biosynthesis of Dpg (3,5-dihydroxyphenyl-glycine). Dark blue arrows: other genes conserved in all META-B loci (ABC transporter, MbtH-like protein and transcriptional regulator AraC). Green arrows and yellow arrow: genes conserved in strains X11-5A and XaS3 predicted to be required for biosynthesis of an unknown non-proteinogenic amino acid (Hp: hypothetical protein gene, TA: transaminase gene, DH: lactate dehydrogenase gene). Light blue arrow: *syrP* gene predicted to be required for β-hydroxylation of an unknown amino acid. Black arrows: NRPS genes. Length of arrows is not proportional to the length of genes. All information regarding characteristics and color-code for NRPS modules are as detailed in Figure 2. IS: insertion sequence. FS: frameshift mutation. Dotted box or dotted arrow: pseudogene. Dotted circle: non-functional module.

Genes similar to those from the gene cluster META-B of strain GPE PC73 were found in the recently published [28] draft genome sequences of the two *X. oryzae* pv. *oryzae* strains X11-5A and X8-1A, and in unpublished draft genome sequences of *Xanthomonas* spp. strain XaS3 and *X. oryzae* pv. *oryzae* strain BAI3. In these draft sequences, fragments of NRPS genes were distributed in several independent contigs. BLAST analyses indicated that the META-B gene cluster was very similar in strains X11-5A and X8-1A. We therefore analyzed only strain X11-5A (Figure 3). For strains X11-5A and XaS3, *in silico* analyses were performed directly on the draft sequence by analyzing each contig independently. For strain BAI3, we performed additional cloning and sequencing experiments to assemble the contigs and determine the complete sequence of an 82-kb region containing META-B.

**Figure 4:**
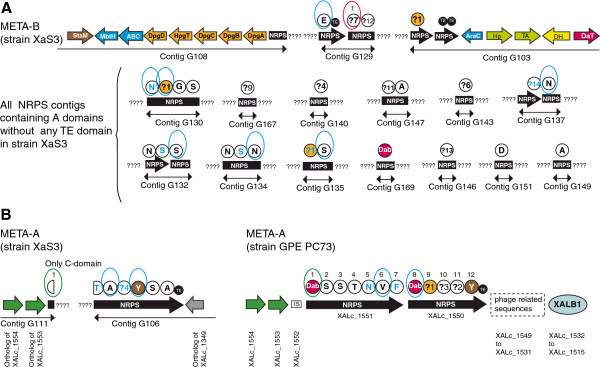
**Representation of NRPS sequences of *Xanthomonas *spp. strain XaS3. A.** Representation of contigs of *Xanthomonas* spp. strain XaS3 that contain a nucleotide sequence encoding at least one A domain and/or a nucleotide sequence encoding non-NRPS genes similar to genes found in the locus META-B of strain *X. albilineans* GPE PC73. **B.** Comparison of the locus META-A of *X. albilineans* strain GPE PC73 with the two contigs of *Xanthomonas* spp. strain XaS3 predicted to belong to a locus similar to this locus META-A. Brown arrows: *staM* and *staM’* genes predicted to be required for β-hydroxylation of two unidentified amino acids, respectively. Purple arrow: *daT* gene predicted to be required for biosynthesis of Dab (2,4-diamino butyric acid). Orange arrows: genes predicted to be required for biosynthesis of Dpg (3,5-dihydroxyphenyl-glycine). Dark blue arrows: other genes conserved in all META-B loci (ABC transporter, MbtH-like protein and transcriptional regulator AraC). Green arrows and yellow arrow: genes conserved in strains X11-5A and XaS3 predicted to be required for biosynthesis of an unknown non-proteinogenic amino acid (Hp: hypothetical protein gene; TA: transaminase gene; DH: lactate dehydrogenase gene). Black arrows: NRPS genes. Length of arrows is not proportional to the length of genes. All information regarding characteristics and color-code for NRPS modules are as detailed in Figure 2. IS: insertion sequence. XALB1: albicidin biosynthesis gene cluster. Incomplete circles: incomplete nucleotide sequence located at the end or at the beginning of a contig encoding only one part of a module.

All NRPS-associated genes found in the META-B gene cluster of strain GPE PC73 are conserved in strains BAI3, X11-5A and XaS3 (Figures 3 and 4; Table 3), except for XALc_1052 (the *daT* gene required for biosynthesis of Dab), which is absent in strain BAI3. Interestingly, the META-B loci of strains X11-5A and XaS3 both contain four additional genes that may be required for the biosynthesis of additional unknown non-proteinogenic amino acids (Table 3). These genes encode a protein sharing 58% amino acid similarity with the protein encoded by the *staM* gene of *S. toyocaensis* (*StaM’*), a transaminase (TA), a lactate dehydrogenase (DH) and a hypothetical protein (HP), respectively. This hypothetical protein has a superfamily domain that is found in a variety of structurally related metalloproteins, including type I extradiol dioxygenases, glyoxalase I and a group of antibiotic resistance proteins. Orthologs of this hypothetical protein (Dd703_3065) and of the transaminase (Dd703_3064) are present in a NRPS gene cluster in the genome of the phytopathogenic strain Ech703 of *Dickeya dadantii*, supporting the assumption that both genes are required for biosynthesis of non-ribosomally synthesized peptides. Orthologs of the hypothetical protein Dd703_3065 are also present in the three Asian *X. oryzae* pv. *oryzae* strains MAFF 311018, PXO99A and KACC10331 in the region containing the XaPPTase gene (Figure 1). This suggests that, in the common ancestor of *X. oryzae* strains, META-B may have been already present in the same genomic region as the XaPPTase gene. Contig 112.1 of strain X11-5A, which contains the *daT* gene and the three additional genes mentioned above, also contains eight genes that are not predicted to be required for NRPS on the basis of their sequence. These eight genes are conserved in all other *X. oryzae* strains but are present elsewhere in the genome (Figure 1). This finding supports the conclusion that recombination events had shaped the META-B locus in the ancestor of strain X11-5A. Remnants of the gene encoding the lactate dehydrogenase DH were found in strain GPE PC73 between genes XALc_1052 and XALc_1053, confirming its ancestral origin (Figure 2). In strain X11-5A, the META-B locus contains a *syrP* gene sharing 44% amino acid similarity with the *syrP* gene of *Pseudomonas syringae* pv. *syringae* strain B728a, which was shown to be required for β-hydroxylation of the aspartyl residue in the phytotoxin syringomycin E [44]. No aspartyl residue is predicted to be assembled by META-B in strain X11-5A, suggesting that the *syrP* gene of strain X11-5A may be required for β-hydroxylation of another residue. In strain BAI3, the *syrP* gene should be considered as a pseudogene because it contains a non-sense mutation (Figure 3). The *syrP* gene is also not conserved in the META-B loci of strains GPE PC73 or XaS3. However, in strain GPE PC73, the albicidin biosynthesis gene cluster XALB1 does possess a *syrP* gene (XALc_1524), which shares 42% and 49% amino acid similarity with the *syrP* gene of *P. syringae* pv. *syringae* and the *syrP* gene of strain X11-5A, respectively. The *syrP* gene XALc_1524 may be involved in β-hydroxylation of residues that are assembled by XALB1, but also by META-A, META-B and META-C.

**Table 3:** **Similarities between NRPS-associated genes present in loci META-B of *Xanthomonas *spp. XaS3, *X. albilineans *strain GPE PC73 and *X. oryzae *pv. *oryzae *strains BAI3 and X11-5A**

**Proteins encoded in the locus META-B of *Xanthomonas *spp. strain XaS3**	**Predicted functions**	**Overall amino acid identities / similarities with proteins encoded by the locus META-B of *Xanthomonas *spp. XaS3**		
		***X. albilineans *strain GPE PC73**	***X. oryzae *pv. *oryzae *strain BAI3**	***X. oryzae *pv. *oryzae *strain X11-5A**
StaM	β-hydroxylation	98% / 99%	90% / 95%	90% / 94%
StaM’	β-hydroxylation	Not encoded	Not encoded	87% / 92%
MbtH	Assembly of amino acids by NRPS	95% / 96%	94% / 96%	94% / 96%
ABC	Secretion of the synthesized peptides	91% / 93%	78% / 83%	77% / 81%
DpgD	Biosynthesis of Dpg	94% / 96%	86% / 90%	86% / 90%
HpgT	Biosynthesis of Dpg	90% / 93%	80% / 88%	83% / 91%
DpgC	Biosynthesis of Dpg	92% / 95%	80% / 87%	81% / 88%
DpgB	Biosynthesis of Dpg	78% / 86%	69% / 79%	69% / 79%
DpgA	Biosynthesis of Dpg	92% / 94%	85% / 89%	86% / 90%
AraC	Transcriptional regulation	93% / 96%	57% / 72%	57% / 71%
DaT	Biosynthesis of Dab	95% / 97%	Not encoded	76% / 84%
Hypothetical protein	Unknown	Not encoded	Not encoded	76% / 84%
Transaminase	Unknown	Not encoded	Not encoded	74% / 82%
Lactate dehydrogenase	Unknown	Not encoded	Not encoded	79% / 89%

Dab: 2,4-diamino butyric acid; Dpg: 3,5-dihydroxyphenyl-glycine.

C-domains very similar to the starter C-domain of the locus META-B of strain GPE PC73 were found in strains XaS3, X11-5A and BAI-3, confirming that these three strains possess a NRPS gene cluster similar to META-B. Additionally, a C-domain very similar to the starter C-domain of META-A was found in another contig of strain XaS3 (Figure 4). While sharing common characteristics, NRPS genes present in loci META-A, META-B or META-C in strains GPE PC73, BAI3, X11-5A or XaS3 are predicted to encode different NRPS systems, each being strain-specific. NRPS genes of each strain are described and discussed separately below.

### Genomic features of NRPS genes associated with the META-B gene cluster of strain BAI3

The META-B locus of African strain BAI3 of *X. oryzae* pv. *oryzae*, which is situated next to a transcription activator-like (TAL) effector locus, contains three NRPS genes: NRPSa (14,484 bp), NRPSb (22,410 bp) and NRPSc (19,479 bp) (Figure 3). These three genes encode a NRPS system consisting of 17 modules of the characteristic domain arrangement C-A-PCP. One double TE-domain is present at the terminal module of NRPSa. Phylogenetic analysis identified the N-terminally located C-domain of the first module of NRPSb as the starter C-domain (Additional file 2). This C-starter domain shares 74% amino acid similarity with the starter C-domain of the META-B locus of strain GPE PC73 (Additional file 4). Therefore, the first module of NRPSb was assigned as the initiation module and the last module of NRPSa, which harbors the TE domain, was assigned as the termination module (Figure 3). Phylogenetic analysis of C-domains identified four modules exhibiting a dual C/E domain that are predicted to follow modules that assemble an amino acid with d-configuration in strain BAI3 (Figure 3; Additional file 1).

The META-B biosynthesis gene cluster of strain BAI3 also contains a pseudogene consisting of three degenerated NRPS modules that contain a frame-shift mutation or an insertion sequence, respectively (Figure 3). Ten out of 17 amino acids assembled by the locus META-B of strain BAI3 were predicted on the basis of analysis of specificity-conferring signatures of A-domains (Additional file 1; see Figure 3). Interestingly, one of the degenerated modules exhibits a signature specific to Dab, suggesting that a putative ancestor of strain BAI3 possessed the gene *daT* required for biosynthesis of Dab. Moreover, five unknown specificity-conferring signatures of A-domains were found in strain BAI3. Remarkably, the META-B locus is the only NRPS locus identified in the whole genome of strain BAI3.

### Genomic features of NRPS genes associated with the META-B gene cluster of strain X11-5A

The contig 23.1 of the American strain X11-5A of *X. oryzae* pv. *oryzae* contains several NRPS-associated genes also present in the META-B gene cluster of strain GPE PC73. Two contigs (23.1 and 114.1) of strain X11-5A each contain a nucleotide sequence encoding a chain-terminating TE-domain located just upstream from a nucleotide sequence encoding a C-domain that shares more than 75% amino acid similarity with starter C-domains of the META-B loci of strains GPE PC73 and BAI3, and which segregates in the same phylogenetic clade as these starter C-domains (Additional files 2, 4). Therefore, both contigs 23.1 and 114.1 should belong to the META-B locus, suggesting that, in strain X11-5A, this locus possesses two starter C-domains and two chain-terminating TE domains, and consequently encodes two independent NRPS systems that should be involved in the biosynthesis of two different compounds. The older ancestor of META-B, considered similar to locus META-B of strain X11-5A, likely also encoded two NRPS systems. In strain BAI3, a pseudogene consisting of three degenerated NRPS modules, as well as a *syrP* pseudogene, may be remnants of lost ancestral genes that were required for a second NRPS system likely encoded by the older ancestor of META-B. Similarly, a pseudogene encoding a stand alone double TE-domain in strain GPE PC73 (XALc_1055) may be a remnant of a lost ancestral NRPS gene that was required for a second NRPS system. A total of 29 different nucleotide sequences encoding A-domains were found in all contigs of strain X11-5A (Figure 3; Additional file 1). The absence of nucleotide sequences encoding a TE-domain other than those encoded by contigs 23.1 and 114.1 suggests that all these 29 sequences encoding A-domains belong to the META-B gene cluster of strain X11-5A. Indeed, the total number of nucleotide sequences encoding A-domains belonging to this gene cluster may be even higher because of repeated DNA regions that are not included in the draft genome sequence and that may encode additional A-domains. Amino acids assembled by 14 of these 29 A-domains were predicted on the basis of specificity-conferring signatures of A-domains (Additional file 1; see Figure 3). Seven unknown specificity-conferring signatures of A-domains were identified in strain X11-5A (Additional file 1). Phylogenetic analysis of C-domains identified six modules exhibiting dual C/E domains in this strain (see Figure 3; Additional file 1).

### Genomic features of NRPS genes associated with gene clusters META-A and META-B of *Xanthomonas* spp. strain XaS3

The nucleotide sequence of contig G129 of *Xanthomonas* spp. strain XaS3 encodes a TE-domain located just upstream from a sequence encoding a starter C-domain. This C-domain shares more than 71% amino acid similarity with starter C-domains of the META-B loci of strains GPE PC73 and BAI3, which segregate in the phylogenetic clade of starter C-domains (Additional files 2 and 4). Therefore, contig G129 should belong to the META-B locus. Contigs G108 and G103 are also predicted to belong to the META-B gene cluster because they contain several NRPS-associated genes specific to this locus (Figure 4). Contig G103 contains the same gene as strain GPE PC73 encoding the stand alone double TE-domain predicted to be a pseudogene (ortholog of XALc_1055). Based on these resemblances with the META-B locus of strain GPE PC73, strain XaS3 is predicted to possess a META-B gene cluster (Figure 4) encoding one NRPS system involved in the biosynthesis of one peptide. Contig G111 of strain XaS3 contains a starter C-domain located at the same position as the starter C-domain of locus META-A of strain GPE PC73, and downstream genes orthologous to XALc_1553 and XALc_1554 (Figure 4). This starter C-domain shares 82% amino acid similarity with the starter C-domain of the locus META-A of strain GPE PC73 (Additional file 4). Contig G106 of strain XaS3 contains an incomplete NRPS gene encoding six NRPS modules, with the last module containing a chain-terminating TE domain. The 5′-part of this incomplete NRPS gene is located in a region that does not encode any NRPS in strain GPE PC73, upstream from the orthologous gene of XALc_1349 (Figure 4). However, based on phylogenetic analyses of C-domains, strain XaS3 possesses only two starter C-domains, indicating that contig G106 belongs to the same META-A gene cluster as the starter C-domain of contig G111 (Figure 4). The 5′-part of META-A in strain GPE PC73 is located upstream of both the terminus of replication and the albicidin biosynthesis gene cluster XALB1, which is not present in the genome of strain XaS3. Acquisition of XALB1 by the ancestor of *X. albilineans* or, alternatively, loss of XALB1 by the ancestor of strain XaS3 may explain why the upstream segments of META-A from strains GPE PC73 and XaS3 are located in two different regions. A total of 33 different nucleotide sequences encoding A-domains were found in all contigs of strain XaS3 (Figure 4; Additional file 1): four of these are located in contig G129, which contains nucleotide sequences encoding the chain-terminating TE-domain and the starter C-domain of META-B; and six are located in contig G106 containing the incomplete NRPS gene of META-A. The remaining 23 nucleotide sequences encoding A-domains, which are not located in contigs encoding TE-domains, may belong either to META-A or META-B. The total number of nucleotide sequences encoding A-domains in strain XaS3 may be higher than 33 because of repeated DNA regions that are not included in the draft genome sequence and that may encode additional modules. Amino acids assembled from 21 of these 33 A-domains were predicted on the basis of specificity-conferring signatures of A-domains (Figure 4; Additional file 1). Nine unknown specificity-conferring signatures of A-domains were found in strain XaS3 (Additional file 1). Phylogenetic analysis identified nine modules exhibiting a dual C/E domain (Figure 4; Additional file 1).

### Genome mining reveals a NRPS gene cluster specific to *X. oryzae* pv*. oryzicola* strain BLS256

**Figure 5:**
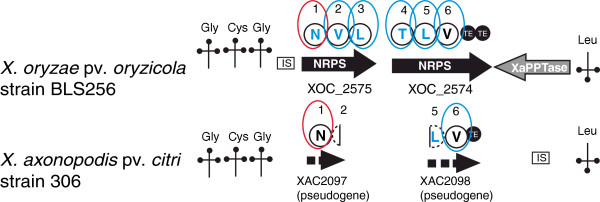
**Representation of the NRPS locus present in the same region as XaPPTase in *X. oryzae *pv. *oryzicola *strain BLS256.** Comparison with the similar genomic region of *X. axonopodis* pv. *citri* strain 306. NRPS modules are represented by circles. Large circles indicate complete NRPS modules (containing domains C, A and PCP). The amino acid predicted to be assembled by the corresponding module is indicated within each circle. Small black circles represent TE domains. Black arrows: NRPS genes. The starter module circled in red exhibits a C-domain belonging to the same phylogenetic clade as starter C-domains identified by Rausch et al. [6] (Additional file 2). Elongation modules circled in blue exhibit a C-domain belonging to the same phylogenetic clade as dual C/E domains identified by Rausch et al. [6] (Additional file 2). Amino acids predicted to be epimerized by these domains are in blue. : tRNA. Orientation of the tag indicates the orientation of the tRNA gene in the genomic regions. Amino acid specificity of each tRNA is indicated above or below each tag according to the orientation of the tRNA gene. IS: insertion sequence. Dotted arrows: pseudogenes. Dotted and incomplete circles: incomplete and non-functional modules.

In *X. oryzae* pv. *oryzicola* strain BLS256, the region containing the XaPPTase gene also contains two large NRPS genes: XOC_2575 (9,513 bp) and XOC_2574 (11,622 bp). These two genes encode a NRPS consisting of six modules each with the three domains (C-A-PCP) followed by a chain-terminating double TE domain (Figure 5). Only the A, PCP, C and TE domains were identified and no auxiliary domains were found in this system. These genes are the only NRPS genes present in strain BLS256. Phylogenetic analysis of C-domains showed that the C-domain of the first module of XOC_2575 belongs to the same clade as starter C-domains identified by Rausch et al. [6]. Elongation modules of strain BLS256, that all exhibit a dual C/E domain, are predicted to catalyze epimerization of five amino acids (Figure 5; Additional file 1). The double TE-domain might catalyze cyclization of the peptide. Orthologs of NRPS genes of strain BLS256, which are present in *X. axonopodis* pv. *citri* strain 306, are only partially conserved and do not encode a complete NRPS system; these should be considered as pseudogenes (Figure 5).

### Genome mining of *Xanthomonas sacchari* reveals genes required for biosynthesis of new non-ribosomal peptide(s)

A non-annotated draft sequence of the genome of *X. sacchari* strain NCPPB4393, isolated from an insect collected on a diseased banana plant, was published recently [45]. The species *X. sacchari*, which is phylogenetically related to *X. albilineans*, was also isolated on sugarcane and milled rice [46,47]. However, no disease caused by this species on any plant has been described to date. Interestingly, the genome of *X. sacchari* strain NCPPB4393 contains the XaPPTase gene (Table 1), which is not located at the same position as on the chromosomes of *X. albilineans* or *X. oryzae*. This genome also contains the genes *daT* (42% identical to XALc_1054), *staM* (38% identical to XALc_1066) and *syrP* (36% identical to XALc_1524). These three latter genes are clustered in a single locus. In addition, the genome of *X. sacchari* strain NCPPB4393 contains several contigs encoding NRPS-related sequences, among them adenylation domains specific to Dab, valine, glutamic acid, phenylalanine, asparagine, proline and an unknown substrate (unknown signature DAWLLGCTFK). However, the NCPPB4393 genome does not contain any sequences that are closely related to META-A, META-B, META-C, XALB1 or the short NRPS genes described above, indicating that non-ribosomal peptide(s) potentially produced by *X. sacchari* is/are specific to this species.

### Genome mining by *in silico* analysis demonstrates a great biosynthetic potential for non-ribosomally synthesized peptides in the genus *Xanthomonas*

While sharing numerous common characteristics, the META-B gene clusters of all four investigated *Xanthomonas* spp. strains do not encode identical small molecules. This was concluded from the A-domain specificities of the NRPSs, and the hypothetical peptides are therefore considered strain-specific. Starter C-domains of all four loci META-B share at least 71% amino acid similarity (Additional file 4) suggesting that, although there is currently no experimental evidence, they might be expected to effect *N-*acylation with a structurally related β-hydroxy-carboxylic acid. Furthermore, the four META-B loci exhibit several C/E dual domains, indicating that they catalyze the assembly of residues with d-configuration. BLAST analyses identified only 188 GenBank entries that exhibit similar dual C/E domains. These 188 entries include nine genes analyzed in the present study (five NRPS genes of strain GPE PC73, two NRPS genes of strain BLS256, and two pseudogenes of strain 306 of *X. axonopodis* pv. *citri*). Draft genome sequence data of strains XaS3 and X11-5A did not indicate the number or distribution of residues in peptides assembled by their respective META-B gene cluster. However, data clearly suggest that these peptides are specific to each strain. In strain X11-5A, the META-B biosynthesis gene cluster is even predicted to control the biosynthesis of two different lipopeptides. A total of five different lipopeptides that do not resemble any compound described to date are predicted for the four *Xanthomonas* spp. strains due to the presence of the META-B gene cluster. These five lipopeptides could be secreted by the ABC transporter encoded by orthologs of XALc_1064, which are present in all META-B gene clusters.

The only common characteristic shared between the loci META-A and META-C of strain GPE PC73 and the locus META-A of strain XaS3, is their unusual starter C-domain, which segregates in a distant phylogenetic clade and shares less that 42% amino acid similarity with starter C-domains of META-B loci or with starter C-domains identified in numerous bacteria by Rausch et al. [6] (Additional file 4). These unusual starter C-domains might catalyze linkage of the first residue with a non-amino acid substrate. A total of three different compounds, not resembling any known compound, are predicted to be produced by *Xanthomonas* spp. strains GPE PC73 and XaS3 by the gene clusters META-A or META-C. Short NRPS genes of strain GPE PC73 might be involved in the biosynthesis of two different small molecules consisting of an amino acid (Gly for XALc_0364 and an unknown amino acid for XALc_1145) linked to a β-hydroxy-carboxylic acid and a glycosyl residue. The only short NRPS gene of strain XaS3 might be involved in biosynthesis of a small molecule identical to that synthesized by XALc_0364 because of the high similarity of these two genes. According to *in silico* analyses, strain BLS256 could synthesize a cyclolipopeptide with the amino acid sequence d-Asn-d-Val-d-Leu-d-Thr-d-Leu-l-Val, which does not resemble any compound described to date (Figure 5). *X. sacchari* strain NCPPB4393 may also produce new non ribosomal peptide(s). Transporters involved in secretion of these unknown compounds remain to be identified as they are not encoded by the NRPS loci.

A total of 14 unknown specificity-conferring signatures of A-domains were found in the four strains GPE PC73, XaS3, X11-5A and BAI3. Only signatures “Unknown2” and “Unknown4” were found in a recently published database that includes 5,118 adenylation domains with unknown signatures [48]. The other 12 unknown NRPS signatures are not present in this database and could be specific to the genus *Xanthomonas*. The signature “Unknown1” is predicted to be specific for Dpg because of its presence in all four strains and because of the absence in these strains of any signature similar to those specific to the non-proteinogenic amino acid identified by [48]. Strains XaS3 and X11-5A possess a higher number of unknown signatures, suggesting that the three genes specific to both strains (transaminase, lactate dehydrogenase and a hypothetical protein) are involved in the biosynthesis of additional non-proteinogenic amino acids. However, these two strains share only one specific unknown signature (“Unknown9”), whereas some unknown signatures are shared by three strains (“Unknown2, -4, -6 and -7”). Eight other unknown signatures are present in only one strain (“Unknown3, -5, -8, -10, -11, -12, -13, and -14”). “Unknown5” was found only in the short gene XALc_1145 of strain GPE PC73. The presence of a high number of unknown signatures specific to the genus *Xanthomonas* suggests that numerous unknown non-proteinogenic amino acids are assembled by the novel NRPS genes identified in the genome of the four *Xanthomonas* spp. The biosynthetic capacities for those amino acids have yet to be identified. In conclusion, our results yield the expectation that numerous non-proteinogenic amino acids as well as residues with d-configuration should confer unusual structures to compounds synthesized by *Xanthomonas* spp. strains; identification of these compounds represents an exciting area for future study in these organisms.

### PCR screening of 94 plant pathogenic bacteria for the XaPPTase gene and genes associated to NRPS in META-B

A collection of 94 strains belonging to the main genera of plant-pathogenic bacteria was screened by PCR. Primers designed to amplify DNA fragments of the XaPPTase gene, the ABC transporter gene present in META-B, the *dpgB* gene, the *dpgC* gene, the *hpgT* gene and the *daT* gene, were designed (Additional file 4). The XaPPTase gene was found in all analyzed strains of *X. albilineans* and *X. oryzae*. It was also found in strain CFBP2539 of *Xanthomonas translucens* pv. *secalis*, strain CFBP4642 of *Xanthomonas cassavae*, strain CFBP2431 of *Pseudomonas corrugata*, strain CFBP5593 of *Pseudomonas brassicacearum* and strain CFBP1192 of *Xylophilus ampelinus* (Additional file 5)*.* The XaPPTase gene of *X. cassavae* is very similar to that of *X. oryzae*, while the XaPPTase gene of *P. corrugata* is very similar to that of *P. brassicacearum* (Additional file 5)*.*

The ABC transporter gene was found in all four analyzed strains of *X. albilineans*, in all five analyzed African strains of *X. oryzae* pv. *oryzae*, in strains UPB497 and CFBP2286 of *X. oryzae* pv. *oryzicola* and in strain CFBP2539 of *X. translucens* pv. *secalis* (Additional file 5)*.* Three additional genes of META-B (the *dpgB* gene, the *dpgC* gene and the *hpgT* gene) were found in all strains possessing the META-B ABC transporter, except for strain CFBP2286 of *X. oryzae* pv. *oryzicola*, which does not possess any of these genes (Additional file 5)*.* The *daT* gene was found in all analyzed strains of *X. albilineans*, in strain UPB497 of *X. oryzae* pv. *oryzicola* and in strain CFBP2539 of *X. translucens* pv. *secalis* (Additional file 5)*.* In summary, the META-B gene cluster seems to be present in all analyzed strains of *X. albilineans*, in all analyzed African strains of *X. oryzae* pv. *oryzae*, in strain UPB497 of *X. oryzae* pv. *oryzicola* and in strain CFBP2539 of *X. translucens* pv. *secalis*.

## Conclusions

This study analyzed published genome sequences of *Xanthomonas* spp. together with unpublished draft genome sequences of *Xanthomonas* spp. XaS3 and *X. oryzae* pv. *oryzae* strain BAI3 for their genetic capacity to producing small molecules. Table 4 summarizes the *in silico* data on the non-ribosomally synthesized peptides predicted to be produced by these *Xanthomonas* strains. Unfortunately, because of the presence of unknown signatures and unusual starter C-domains, we were unable to predict possible structures of any of the products of the identified biosynthesis gene clusters. However, this study revealed that four strains of the genus *Xanthomonas* possess up to three novel homologous gene clusters, termed META-A, META-B or META-C, encoding NRPS peptides. A phosphopantetheinyl transferase (XaPPTase), which is essential for activation of NRPS enzymes, was identified in all strains investigated. Furthermore, sequence alignment of these META-clusters from *Xanthomonas* strains is indicative of the biosynthesis of lipopeptides or peptides linked to another non-amino acid substrate, and involving the biosynthesis and incorporation of non-proteinogenic amino acids Dpg, Dab and amino acid(s) of unknown identity including at least one β-hydroxy-amino acid. The identity of several amino acids could not be predicted from the signature sequences of A-domain specificities. Hence, at least eight different peptides may be synthesized, the partial sequences of which have been predicted. This study revealed that each peptidic sequence is strain-specific (Table 4). This suggests that other *Xanthomonas* spp. strains, although they all possess the META-B locus, are expected to produce structurally different peptides, varying in size and amino acid composition. Small molecules synthesized by other NRPS genes analyzed in strain BLS256 of *X. oryzae* pv. *oryzicola*, strain NCPPB4393 of *X. sacchari* and strain GPE PC73 of *X. albilineans* are also likely candidates for the biosynthesis of new metabolites from *Xanthomonas* spp. strains (Table 4). If these biosynthetic pathways are functional, the resulting peptides may exert different biological functions in plant–bacteria interactions. Interestingly, these biosynthetic pathways seem to be shared only by strains of *Xanthomonas* associated with monocotyledonous plants, suggesting a putative involvement of novel non-ribosomally synthesized peptides in plant–bacteria interactions. The XaPPTase gene might be a useful probe for further genome mining of *Xanthomonas* spp. and related strains. In summary, this extensive *in silico* study shows that the genus *Xanthomonas* constitutes a promising reservoir of new non-ribosomally synthesized peptides. Experimental elucidation of this promising biosynthetic potential should contribute to the study of plant-bacteria interactions as well as to drug discovery. Interestingly, a first step of this elucidation was recently achieved with the isolation of the lipopeptide synthesized by the gene cluster META-B of *X. albilineans* strain GPE PC73. The nominal molecular mass of this lipopeptide is 2,293 Da and subsequent MS/MS-experiments revealed an amino acid sequence which excellently matches the one predicted by *in silico* analysis of A-domain specificities of META-B NRPSs (G.H. Völler, unpublished data).

**Table 4:** ***In silico *predicted data on non-ribosomally synthesized peptides potentially produced by *Xanthomonas *strains**

***Xanthomonas *strains**	**Non-ribosomally synthesized peptides**	
	Lipopeptides containing non-proteinogenic amino acids Dpg, Dab and/or at least one β-hydroxy-amino acid*	**Others**
*X. albilineans* strain GPE PC73	- Lipopeptide synthesized by META-B with the amino acid sequence Gly-Unk2-Thr-D-Asn-Tyr-Asn-D-Tyr-Ser-D-Asn-Asp-Asn-Dpg-Dpg-Ser-D-Thr-Unk2	- Glycine linked to a β-hydroxy-carboxylic acid and a glycosyl residue
		- Unknown amino acid linked to a β-hydroxycarboxylic acid and a glycosyl residue
	- Lipopeptide synthesized by META-A with the amino acid sequence Dab-Ser-Ser-Thr-D-Asn-Val-D-Phe-Dab-Dpg-Unk3-Unk2-Tyr	
	- Lipopeptide synthesized by META-C with the amino acid sequence Unk4-Phe-Dab-Asn-Ala-D-Unk3-Gln	
*Xanthomonas* spp*.* strain XaS3	- Two lipopeptides synthesized by META-B and META-A, respectively.**	- Glycine linked to a β-hydroxy-carboxylic acid and a glycosyl residue
*X. oryzae* pv. *oryzae* strain BAI3	-Lipopeptide synthesized by META-B with the amino acid sequence Gly-Unk7-Unk2-Thr-Asn-D-Thr-Tyr-D-Ile-Ser-Asn-D-Asn-Ser-Dpg-Dpg-Unk6-D-Dpg-Unk4	
*X. oryzae* pv. *oryzae* strain X11-5A	- Two lipopeptides synthesized by META-B.**	
*X. oryzae* pv. *oryzicola* strain BLS256		- A cyclolipopeptide with the amino acid sequence D-Asn-D-Val-D-Leu-D-Thr-D-Leu-Val
*X. sacchari* strain NCPPB4393		No precise data on the number and nature of the non-ribosomally synthesized peptides produced by this strain because of the lack of information (only draft genome sequence including several unassembled contigs that contain NRPS sequences)

Unk: Unknown amino acid; Dab: 2,4-diamino butyric acid; Dpg: 3,5-dihydroxyphenyl-glycine.

* The number and nature of amino acid(s) β-hydroxylated by proteins encoded by *staM*, *staM’* or *syrP* remain unidentified.

** Only partial amino acid sequences of these lipopeptides are available due to insufficient information (contigs containing gene clusters META-B or META-A are unassembled).

## Methods

### Bacterial strains

*X. albilineans* strain GPE PC73 isolated in Guadeloupe was sequenced recently [27]. *X. albilineans* strains were grown for 48h on modified Wilbrink’s medium [49] or on XAS selective growth medium [50] at 28°C. *X. oryzae* pv. *oryzae* strain BAI3 (isolated in Burkina Faso [33]) was grown for 24h on PSA medium [51] supplemented with appropriate antibiotics at 28°C.

### Design of PCR primers

Primers used to screen a collection of 94 plant pathogenic bacteria for the presence of the XaPPTase gene as well as genes associated to NRPS in META-B by PCR were designed based on genome sequence information of strains *X. albilineans* GPE PC73, *X. oryzae* pv. *oryzae* BAI3, and *X. translucens* pv. *undulosa* UPB513 (Claude Bragard, unpublished data). Primer sequences are listed in Additional file 4. Primers used to determine the complete sequence of the 82-kb length region containing META-B in strain BAI3 were designed based on the draft genome sequence of *X. oryzae* pv. *oryzae* strain BAI3 (Genbank accession n° JQ348075).

### PCR screening for the presence of the XaPPTase gene and genes associated to NRPS in META-B

DNA templates were prepared by suspending a freshly grown colony in 100 μl sterile nuclease-free water. PCR amplifications were performed in an automated thermal cycler (GeneAmp PCR System 9700; Life Technologies, Carlsbad, CA, USA). The 20-μl PCR reaction mix consisted of 5 μl bacterial suspension, 4 μl of 5x GoTaq buffer (Promega, Madison, WI, USA), 250 μM dNTP mix, 0.2 μM of each primer, 1 unit of GoTaq Polymerase (Promega, Madison, WI, USA), and sterile nuclease-free water to final volume. The PCR program was 94°C for 4 min, 35 cycles at 94°C for 30 seconds, Tm (melting temperature) for 30 seconds, and 72°C for 30 seconds, with a final 72°C extension for 8 min. A 8-μl aliquot of each amplified product was analyzed by electrophoresis through a 1.5% agarose gel. PCR products were sequenced with primers used for their respective amplification. Screened bacteria are listed in Additional file 6.

### Genome sequencing

The draft genome sequence of strain BAI3 of *X. oryzae* pv. *oryzae* was determined by a Sanger/pyrosequencing hybrid approach. A shotgun library was constructed with 10-kb sized fragments obtained after mechanical shearing of the total genomic DNA, and cloned into the vector pCNS (pSU-derived). Sequencing with vector-based primers was carried out on an ABI 3730 Applera Sequencer. A total of 5,921 reads (~1 fold coverage) were analyzed and assembled with 518,656 (~23 fold coverage) 454 GS FLX reads (Roche Applied Science; http://www.roche.com). Sequence assembly was performed using Arachne “HybridAssemble” version (Broad Institute; http://www.broad.mit.edu/), which combines the 454 contigs with Sanger reads. As the Sanger reads contribution was not quite sufficient for the scaffolding, a mate-paired 454 library with 8-kb insert size was constructed and 179,755 (~4 fold coverage) 454 GS FLX reads were added into the assembly. To further improve quality, Illumina technology was applied (36-bp reads at ~50 fold coverage), eventually resulting in an assembly of 67 scaffolds with a mean scaffold length of 80 kb.

Draft genome sequence of *Xanthomonas* spp. strain XaS3 (also called strain GPE39), was obtained using short read technology only. After mechanical shearing of genomic DNA, fragments with an insert size of 300–500 bp were used for Illumina library construction (http://www.illumina.com). A total of 64,891,306 GAIIx reads were produced, corresponding to a paired-end sequencing with 2 × 76 nt reading lengths. Assembly was performed using a combination of the SOAPdenovo (1.05) and Velvet (1.1.04) short read assemblers [52,53]. In a first step, SOAPdenovo was run with kmer values ranging from 25 to 73 (step of 4) to generate contigs. In a second step, Velvet was run with parameters “-cov_cutoff 5 -min_contig_lgth 100 -max_divergence 0.05 -exp_cov auto -min_pair_count 10” to generate scaffolds, by using raw reads and SOAPdenovo contigs as input data. The range of kmer values used for Velvet was the same as for SOAPdenovo. The assembly with the maximum N50 value was selected, and remaining gaps were filled with SOAPGapCloser software. The XaS3 draft assembly spans 3,529,206 bp, and consists of 73 contigs of length ranging from 526 to 512,021 bp with a N50 of 168,272 bp. Annotated nucleotide sequences of the regions identified in these 73 contigs as containing A-domains and/or NRPS associated genes are provided in Additional file 7.

### Determination of the complete sequence of the 82-kb length region containing META-B in *X. oryzae* pv*. oryzae* strain BAI3

On the basis of the draft genome sequence of strain BAI3, three scaffold DNA sequences were identified as belonging to META-B. The sizes of these DNA regions are 35,406 bp, 16,409 bp and 22,029 bp, respectively. Seventeen clones from a 10-kb shotgun library of strain BAI3 were identified by BLAST analyses to contain fragments of the META-B NRPS cluster. These clones are ABP0AAB3YI02, ABP0AAB3YP05, ABP0AAB8YK02, ABP0AAB8YE14, ABP0AAB5YJ09, ABP0AAB4YK08, ABP0AAB3YL17, ABP0AAB6YE03, ABP0AAB6YC14, ABP0AAB5YG12, ABP0AAB7YB12, ABP0AAB5YK06, ABP0AAB5YJ04, ABP0AAB7YD04, ABP0AAB3YG10, ABP0AAB5YE07 and ABP0AAB3YM11. Sequences of the insert ends were mapped onto the three scaffold DNA sequences. Four clones (ABP0AAB4YK08, ABP0AAB3YL17, ABP0AAB7YB12 and ABP0AAB5YK06) were identified as bridging two of the three scaffold DNA sequences. All other clones were mapped to within one of the three scaffold DNA sequences. Clones ABP0AAB4YK08, ABP0AAB3YL17, ABP0AAB7YB12 and ABP0AAB5YK06 were digested with restriction enzyme *Xho*I and partial digestion with restriction enzyme *Sal*I followed by re-ligation. The restriction site *Xho*I is present only in the polylinker of pCNS vector and several *Sal*I restriction sites are present in the inserts. Clone ABP0AAB7YB12 was also digested with restriction enzyme *Bam*HI followed by re-ligation. The borders of the resulting clones were sequenced using universal primers (M13R for *Bam*HI borders and M13F for *Xho*I borders). One clone resulting from an internal *Sal*I-mediated deletion of clone ABP0AAB5YK06, and harboring an insert of 3.6 kb, was sequenced with primers MRK16, MRK17, MRK18, MRK19 and MRK20 (Additional file 8). A clone resulting from an internal *Sal*I-mediated deletion of clone ABP0AAB7YB12 and harboring an insert of 1.9 kb was sequenced with primers MRK19 and MRK20 (Additional file 8). Clone ABP0AAB5YK06 was sequenced with primers MRK7, MRK8, MRK18, MRK22R and MRK23R. The new sequence information allowed the three scaffold DNA sequences to be merged into a contiguous scaffold sequence of 82 kb. Contigs of strain BAI3 were mapped to this sequence and three sets of primers were designed in order to sequence (i) gaps between contigs, (ii) regions between NRPS and a *tal* gene, and (iii) regions with frameshifts, respectively (Additional file 8). These primers were used to sequence a clone resulting from an internal *Sal*I-mediated deletion of clone ABP0AAB4YK08 and harboring an insert of 5.0 kb and to sequence clones ABP0AAB3YG10, ABP0AAB3YM11, ABP0AAB5YE07, ABP0AAB5YJ04, ABP0AAB6YC14, ABP0AAB6YE03, ABP0AAB7YB12 and ABP0AAB7YD04 (Additional file 8). DNA sequencing was performed by Beckman Coulter Genomics (Takely, UK). Sequence reads were used to assemble the complete 81,740-bp META-B region of strain BAI3 (Genbank accession n° JQ348075).

### *In silico* analyses of NRPS

Specificity of adenylation domains in NRPS and signatures were predicted using software available at http://nrps.informatik.uni-tuebingen.de/Controller?cmd=SubmitJob[54]. *In silico* analyses were performed on NRPS genes present in the finished annotated genome sequence of *X. albilineans* strain GPE PC73 (GenBank accession n°: NC_013722.1), in the non-annotated draft genome sequence of *X. oryzae* pv*. oryzae* strain X11-5A (GenBank accession n°: AFHK00000000.1; sequences analyzed in the current study are annotated in Additional file 9), in the unpublished annotated sequence of the 82-kb length region containing META-B in *X. oryzae* pv*. oryzae* strain BAI-3 (Genbank accession n° JQ348075), in the unpublished sequence of the contigs of strain *Xanthomonas* spp. XaS3 (sequences analyzed in the current study were annotated in Additional file 7), in the finished annotated genome sequence of *X. oryzae* pv. *oryzicola* strain BLS256 (nucleotide GenBank accession n°: CP003057.1), in the finished annotated genome sequence of *X. axonopodis* pv. *citri* strain 306 (nucleotide GenBank accession n°: AE008923.1), and in the non-annotated draft genome sequence of *X. sacchari* strain NCPPB4393 (nucleotide GenBank accession n°: AGDB00000000.1).

### Phylogenetic analysis

The phylogenetic tree presented in Additional file 2 was reconstructed using the maximum likelihood method implemented in the PhyML program. The LG substitution model was selected, assuming an estimated proportion of invariant sites (of 0.01) and four gamma-distributed rate categories to account for rate heterogeneity across sites. The locus analyzed was the C-domain. Multiple alignments of the amino acid sequences of the C-domain and for all taxa were performed using ClustalW. The phylogenetic tree was calculated with PhyML; http://atgc.lirmm.fr/phyml/, version 2.4.4. Five hundred bootstrap replicates were performed with PhyML program. Data are available from the Dryad Digital Repository: http://doi.org/10.5061/dryad.gh7h8.

## Abbreviations

A-domain: Adenylation domain; ACP-domain: Acyl carrier protein domain; C-domain: Condensation domain; Cy-domain: Heterocyclization domain; Dab: 2,4-diamino butyric acid; Dpg: 3,5-dihydroxyphenyl-glycine; E-domain: Epimerization domain; C/E domain: Condensation/epimerization domain; NMR: Nuclear magnetic resonance; NRPS: Non-ribosomal peptide synthesis; NRPSs: Non-ribosomal peptide synthetases; MLSA: Multi locus sequence analyses; MS: Mass spectrometry; PCP-domain: Peptidyl carrier protein domain; P-pant: 4′-phosphopantetheinyl; PPTase: 4'-phosphopantetheinyl transferase; TAL: Transcription activator-like; TE-domain: Thioesterase domain.

## Competing interests

The authors declare that they have no competing interests.

## Authors’ contributions

MR supervised cloning experiments for determination of the complete sequence of the 82-kb length region containing META-B in strain BAI3, performed most of *in silico* analysis of NRPS loci, conceived the study and drafted part of the manuscript. RK discovered the presence of large NRPS in the genome of strain BAI3, contributed to determination and analysis of the sequence of contigs of strain BAI3 and drafted part of the manuscript. MM contributed to *in silico* analysis of NRPS loci and drafted part of the manuscript. VB provided sequencing reads used to determine the draft genome sequences of strains BAI3 and XaS3. SC (Carrere) contributed to determination of the sequence of contigs of strains BAI3 and XaS3. GPR, CG, MH, GHV and SR contributed to *in silico* analysis of NRPS loci. CB performed PCR screening of the collection of 94 plant pathogenic bacteria. JN performed cloning experiments for determination of the 82-kb length region containing META-B in strain BAI3. IP performed phylogenetic analysis and drafted part of the manuscript. VV conceived the sequencing project of strain BAI3. SP supervised PCR screening of the collection of 94 plant pathogenic bacteria. PR conceived the sequencing project of strains XaS3 and GPE PC73 and revised the manuscript. RDS and SC (Cociancich) conceived the study, contributed to *in silico* analysis of NRPS loci, supervised the preparation of the manuscript and drafted part of the manuscript. All authors read and approved the final manuscript.

## Supplementary Material

Additional file 1 **Domains, signature sequences and predicted assembled residues of the NRPS genes described in this study.** Sheet 1: Domains, signature sequences and predicted assembled residues of the NRPS genes present in loci META-A, META-B and META-C of *X. albilineans* strain GPE PC73. Sheet 2: Domains, signature sequences and predicted assembled residues of the NRPS genes present in the gene cluster META-B of *X. oryzae* pv. *oryzae* strain BAI3. Sheet 3: Domains, signature sequences and predicted assembled residues of the NRPS genes present in the NRPS contigs of *X. oryzae* pv. *oryzae* strain X11-5A. Sheet 4: Domains, signature sequences and predicted assembled residues of the NRPS genes present in the NRPS contigs of *Xanthomonas* spp. strain XaS3. Sheet 5: Domains, signature sequences and predicted assembled residues of the NRPS genes identified in the genome of *X. oryzae* pv. *oryzicola* strain BLS256 in the same region as the XaPPTase gene. Click here for file

Additional file 2 **Tree of the amino acid sequences of C-domains of strains GPE PC73, XaS3, X11-5A, BAI3, BTAi and BLS256 together with C-domains identified by Rausch et al. [**[6]**] as starter C-domains or as dual C/E domains.** The tree was constructed using the maximum likelihood method and GTR as substitution model. Bootstrap percentages retrieved in 100 replications are shown at the main nodes. The scale bar (0.2) indicates the number of amino acid substitutions per site. Click here for file

Additional file 3 **Comparison of short NRPS genes and their associated overlapping glycosyltransferase genes of *X. albilineans* strain GPE PC73 with similar genes present in the genome of other bacteria. Table A**: Comparison of short NRPS genes. **Table B**: Comparison of glycosyltransferase genes. Presence/absence of a gene similar to the XaPPTase gene in the genome of other bacteria. Click here for file

Additional file 4 Primers used for PCR screening of a collection of 94 plant pathogenic bacteria for the presence of XaPPTase gene and several genes associated to NRPS in META-B. Click here for file

Additional file 5 Summary of the results of the PCR screening of the collection of 94 plant pathogenic strains. Click here for file

Additional file 6 Collection of strains screened for the presence of XaPPTase gene and genes associated with NRPS in META-B. Click here for file

Additional file 7 Annotated nucleotide sequence of the regions encoding A-domains and/or NRPS associated genes in the contigs of strain XaS3. Click here for file

Additional file 8 Primers used to determine the 82-kb length region containing META-B in strain BAI-3. Click here for file

Additional file 9 **Annotation of the contigs of the published draft genome sequence of *X. oryzae* pv. *oryzae* strain X11-5A which were analysed in the current manuscript.** Click here for file
