# Supplementary material for: Genome mining reveals the genus Xanthomonas to be a promising reservoir for new bioactive non-ribosomally synthesized peptides
Source: BMC Genomics. 2013 Sep 27;14:658. doi: 10.1186/1471-2164-14-658 (PMC3849588; doi:10.1186/1471-2164-14-658)

**Additional file 2:** **Tree of the amino acid sequences of C-domains of strains GPE PC73, XaS3, X11-5A, BAI3, and BLS256 together with C-domains identified by Rausch et al. [**6**] as starter C-domains or as dual C/E-domains. The tree was constructed using the maximum likelihood method and GTR as substitution model. Bootstrap percentages retrieved in 100 replications are shown at the main nodes. The scale bar (0.2) indicates the number of amino acid substitutions per site.**

C-domains belonging to the same clade as dual C/E-domains are in blue.

C-domains belonging to the same clade as starter C-domains are in red.

Putative starter C-domains of the loci META-A and META-C of strain GPE PC73, the contig G111 of strain XaS3 and the locus of strain BTAi similar to META-A and META-C are in green.

C-domains Ax, Bx ad Cx correspond to C-domains of modules of the loci META-A, META-B and META-C of strain GPE PC73, respectively.

C-domains Ox correspond to C-domains of modules of the locus META-B of strain BAI3.

C-domains USxxx/x correspond to C-domains of modules of contigs of strain X11-5A.

C-domains Gxxx/x correspond to C-domains of modules of contigs of strain XaS3.

C-domains bradyx correspond to C-domains of the locus of *Bradyrhizobium* spp. strain BTAi similar to META-A and META-C (genes Bbta_6814, Bbta_6813, Bbta_6812)

C-domains XOCx correspond to C-domains of the locus NRPS located in the same region as XaPPTase in strain BLS256.

C-domains 0364 and 1145 correspond to C-domains of short NRPS genes XALc_0364 and XALc_1145 of strain GPE PC73, respectively.

C-domain 0354XaS3 corresponds to the short NRPS gene of strain XaS3.

C-domain Bbta4110 corresponds to the short NRPS gene of strain BTAi.

C-domains identified by Rausch et al. [[6](#_ENREF_7)] as starter C-domains were tagged “Starter1” to “Starter15” as follows:

**Starter1**: Pseusyrin.NP_792633.1.m_1_leu

**Starter2**: Pseusp.Q84BQ6.arfA_1_leu

**Starter3**: Pseufluor.YP_259252.1.m_1_leu

**Starter4**: Baciliche.YP_077640.1.lchAA_1_gln

**Starter5**: Nocafarci.YP_117314.1.m_1_orn_lys_arg

**Starter6**: Nocafarci.YP_119006.1.m_1_tyr

**Starter7**: Nocafarci.YP_119328.1.m_1_ser

**Starter8**: Nocafarci.YP_121279.1.m_1_ser

**Starter9**: Strecoeli.NP_627443.1.m_1_ser

**Starter10**: Strchrys.O68487.acmB_1_thr

**Starter11**: Erwicarot.YP_049593.1.m_1_gln

**Starter12**: Strprist.Q54959.snbC_1_thr

**Starter13**: Bacisubti.NP_388230.1.srfAA_1_glu

**Starter14**: Bacisubti.NP_389716.1.ppsA_1_glu

**Starter15**: Baciliche.YP_090052.1.m_1_gln

C-domains identified by Rausch et al. [[6](#_ENREF_7)] as Dual C/E-domains were tagged “DualC/E1” to “DualC/E18” as follows:

**DualC/E1:**Photlumin.NP_929905.1.m_9_thr_TO_val

**DualC/E2:**Photlumin.NP_930489.1.m_2_val_TO_trp

**DualC/E3:**Photlumin.NP_929905.1.m_6_bht_TO_trp

**DualC/E4:**Bradjapon.NP_768748.1.m_3_ser_TO_phe

**DualC/E5:**Chroviola.NP_902472.1.m_3_val_TO_ile_dual

**DualC/E6:**Chroviola.NP_902472.1.m_1_thr_dual

**DualC/E7:**Burkmalle.YP_106216.1.m_2_glu_TO_gly

**DualC/E8:**Burkpseud.YP_111641.1.m_3_thr_TO_leu

**DualC/E9:**Burkpseud.YP_111641.1.m_1_glu_gln

**DualC/E10:**Pseusyrin.NP_792633.1.m_2_leu_TO_leu

**DualC/E11:**Ralssolan.NP_522203.1.m_3_ser_TO_gly

**DualC/E12:**Ralssolan.NP_522203.1.m_1_val

**DualC/E13:**Pseufluor.YP_259253.1.m_4_leu_TO_ser

**DualC/E14:**Pseufluor.YP_259253.1.m_2_thr_TO_ile

**DualC/E15:**Pseusyrin.NP_792634.1.m_3_thr_TO_val

**DualC/E16:**Pseusyrin.NP_792634.1.m_5_leu_TO_leu

**DualC/E17:**Erwicarot.YP_049592.1.m_4_ser_TO_tyr_bht

**DualC/E18:**Erwicarot.YP_049593.1.m_2_gln_TO_asn


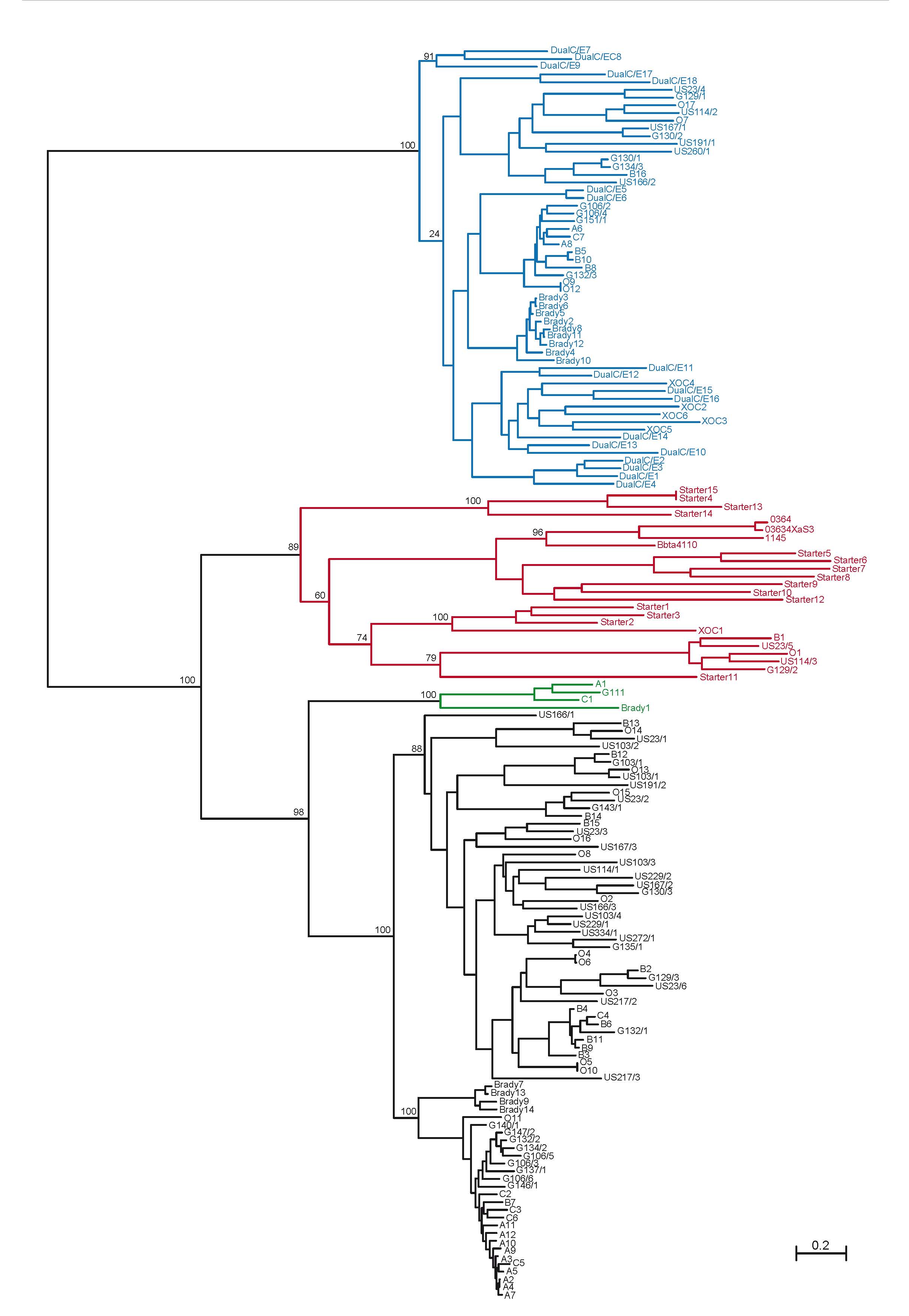

Supplement: Additional file 2 — Tree of the amino acid sequences of C-domains of strains GPE PC73, XaS3, X11-5A, BAI3, BTAi and BLS256 together with C-domains identified by Rausch et al. [[6]] as starter C-domains or as dual C/E domains. The tree was constructed using the maximum likelihood method and GTR as substitution model. Bootstrap percentages retrieved in 100 replications are shown at the main nodes. The scale bar (0.2) indicates the number of amino acid substitutions per site. [file 1471-2164-14-658-S2.docx]
