# Supplementary material for: Genome mining reveals the genus Xanthomonas to be a promising reservoir for new bioactive non-ribosomally synthesized peptides
Source: BMC Genomics. 2013 Sep 27;14:658. doi: 10.1186/1471-2164-14-658 (PMC3849588; doi:10.1186/1471-2164-14-658)
Supplement: Additional file 3 — Comparison of short NRPS genes and their associated overlapping glycosyltransferase genes of X. albilineans strain GPE PC73 with similar genes present in the genome of other bacteria. Table A: Comparison of short NRPS genes. Table B: Comparison of glycosyltransferase genes. Presence/absence of a gene similar to the XaPPTase gene in the genome of other bacteria. [file 1471-2164-14-658-S3.docx]

**Additional file 3: Comparison of short NRPS genes and their associated overlapping glycosyltransferase genes of *X. albilineans* strain GPE PC73 with similar genes present in the genome of other bacteria.**

**A.** Comparison of short NRPS genes.

| Bacteria | NRPS genes | Amino acid identities with XALc_0364 | Amino acid identities with  XALc_1145 | NRPS domains | Signatures |
| --- | --- | --- | --- | --- | --- |
| *X. albilineans* str. GPE PC73 | XALc_0364 | 100% / - | 50% / 66% | C-A-PCP-TE | DILQLGLIWK(Gly) |
| *X. albilineans* str. GPE PC73 | XALc_1145 | 50% / 66% | 100% /- | C-A-PCP-TE | DMLELGMVWK  (Unknown5) |
| *X. albilineans* str. XaS3 | Ortholog of XALc_0364 | 91%/ 94% | 51% / 67% | C-A-PCP-TE | DILQLGLIWK(Gly) |
| *X. campestris* pv. *campestris* str. ATCC 33913 | XCC3867 | 66% / 77% | 52% / 68% | C-A-PCP-TE | DILQLGLIWK(Gly) |
| *X. campestris* pv. *campestris* str. 8004 | XC_3952 | 66% / 77% | 52% / 68% | C-A-PCP-TE | DILQLGLIWK(Gly) |
| *X. campestris* pv. *campestris* str B100 | Xccb100_4052 | 66% / 77% | 52% / 68% | C-A-PCP-TE | DILQLGLIWK(Gly) |
| *X. axonopodis* pv. *citri* str. 306 | XAC3922 | 65% / 77% | 52% / 67% | C-A-PCP-TE | DILQLGLIWK(Gly) |
| *Bradyrhizobium* sp. BTAi1 | Bbta_4110 | 34% / 47% | 33% / 48% | C-A-PCP-TE | DAMLIGAICK  (UnknownBrady2) |
| *Alcanivorax borkumensis* str. SK2 | ABO_1784 | 38% / 54% | 39% / 57% | C-A-PCP-TE | DILQLGLIWK(Gly) |

C : condensation domain ; A : adenylation domain ; PCP : peptidyl carrier protein domain ; TE : thioesterase domain ; DH : dehydrogenase domain.

**B.** Comparison of glycosyltransferase genes. Presence/absence of a gene similar to the XaPPTase gene in the genome of other bacteria.

| Bacteria | Glycosyltransferase genes | Amino acid identities /similarities with XALc_0365 | Amino acid identities /similarities with  XALc_1144 | Genes similar to the XaPPTase gene |
| --- | --- | --- | --- | --- |
| *X. albilineans* str. GPE PC73 | XALc_0365 | 100 %/- | 49% / 62% | XALc_1736 |
| *X. albilineans* str. GPE PC73 | XALc_1144 | 49% / 62% | 100 %/- | XALc_1736 |
| *X. albilineans* str. XaS3 | Ortholog of XALc_0365 | 88% / 92% | 48% / 62% | Ortholog of XALc_1736 |
| *X. campestris* pv. *campestris* str. ATCC 33913 | XCC3866 | 64% / 73% | 52% / 67% | Absent in the genome |
| *X. campestris* pv. *campestris* str. 8004 | XC_3951 | 64% / 73% | 52% / 67% | Absent in the genome |
| *X. campestris* pv. *campestris* str. B100 | Xccb100_4051 | 64% / 73% | 52% / 67% | Absent in the genome |
| *X. axonopodis* pv. *citri* str. 306 | XAC3921 | 62% / 71% | 51% / 66% | Absent in the genome |
| *Bradyrhizobium* sp. BTAi1 | Bbta_4109 | 23% /34% | 23% /33% | Bbta_3710 |
| *Alcanivorax borkumensis* str. SK2 | ABO_1783 | 41% / 53% | 41% / 55% | ABO_1782 |
