# Supplementary material for: Genome mining reveals the genus Xanthomonas to be a promising reservoir for new bioactive non-ribosomally synthesized peptides
Source: BMC Genomics. 2013 Sep 27;14:658. doi: 10.1186/1471-2164-14-658 (PMC3849588; doi:10.1186/1471-2164-14-658)
Supplement: Additional file 4 — Primers used for PCR screening of a collection of 94 plant pathogenic bacteria for the presence of XaPPTase gene and several genes associated to NRPS in META-B. [file 1471-2164-14-658-S4.docx]

**Additional file 4: Primers used for PCR screening**

| **Names of primers** | **Sequences of primers** | **Expected sizes of PCR product*** | **Target gene(s)** |
| --- | --- | --- | --- |
| PPTASEF and PPTASER | AGCGTKCGCAAGCGYCAG and CYTTGAACAGGCTTTCCTT | 378 or 384 bp | XaPPTase gene (orthologs of XALc_1736) |
|  |  |  |  |
|  |  |  |  |
|  |  |  |  |
| ABCF and ABCR | TGTTGACCAACAGCCTGCT and AATGCATGCTGGTGTCGAA | 311 bp | The ABC transporter gene present in gene cluster META-B (orthologs of XALc_1064) |
|  |  |  |  |
|  |  |  |  |
| DpgB2 and DpgB7R | CGCTGGGAAAAGGCGATGCG and CCAACTGCGCCATGCGCGCG | 241 bp | The DpgB gene present in gene cluster META-B (orthologs of XALc_1060) |
|  |  |  |  |
|  |  |  |  |
|  |  |  |  |
| DpgB2 and DpgC1R | CGCTGGGAAAAGGCGATGCG and GCCACCGCTTCGCTGCGCAT | 1,640 bp | The DpgB and DpgC genes present in gene cluster META-B (orthologs of XALc_1060 and XALc_1061) |
|  |  |  |  |
|  |  |  |  |
|  |  |  |  |
| DpgB7 and DpgC1R | CGCGCGCATGGCGCAGTTGG and GCCACCGCTTCGCTGCGCAT | 1,389 bp | The DpgB and DpgC genes present in gene cluster META-B (orthologs of XALc_1060 and XALc_1061) |
|  |  |  |  |
|  |  |  |  |
|  |  |  |  |
| DpgB7 and DpgC5R | CGCGCGCATGGCGCAGTTGG and CGGAAATCCTCCGGCGATTC | 1,440 bp | The DpgB and DpgC genes present in gene cluster META-B (orthologs of XALc_1060 and XALc_1061) |
|  |  |  |  |
|  |  |  |  |
|  |  |  |  |
| DpgC5 and HpgT2R | GAATCGCCGGAGGATTTCCG and GGCAAGGCGATCTTGGCGAA | 914 bp | The HpgT and DpgC genes present in gene cluster META-B (orthologs of XALc_1062 and XALc_1061) |
|  |  |  |  |
|  |  |  |  |
|  |  |  |  |
| DpgC1 and HpgT2R | ATGCGCAGCGAAGCGGTGGC and GGCAAGGCGATCTTGGCGAA | 965 bp | The HpgT and DpgC genes present in gene cluster META-B (orthologs of XALc_1062 and XALc_1061) |
|  |  |  |  |
|  |  |  |  |
|  |  |  |  |
| DaT1 and DaT2R | GGCCACAACGATCCGGACAT and TGTTGCCGCGGAAGGTGCCG | 772 bp | The DaT gene present in gene cluster META-B (orthologs of XALc_1052) |
|  |  |  |  |
|  |  |  |  |
|  |  |  |  |
| * The size differ according to the strain (*X. albilineans* strain GPE PC73 or *X. oryzae* pv. *oryzae* strain BAI3) | | | |
