# Supplementary material for: Genome mining reveals the genus Xanthomonas to be a promising reservoir for new bioactive non-ribosomally synthesized peptides
Source: BMC Genomics. 2013 Sep 27;14:658. doi: 10.1186/1471-2164-14-658 (PMC3849588; doi:10.1186/1471-2164-14-658)
Supplement: Additional file 5 — Summary of the results of the PCR screening of the collection of 94 plant pathogenic strains. [file 1471-2164-14-658-S5.doc]

**Additional file 5: Summary of the results of PCR screening of the collection 94 plant pathogenic strains**

|  |  | **Analysis of the sequence of the corresponding PCR products** | | |
| --- | --- | --- | --- | --- |
| **Names of primers** | **Strains for which a PCR product at the expected size was obtained** | **Overall aminoacid identities with genes XALc_1736 or XALc_1064 of strain GPE PC73 of *X. albilineans*** | **Overall aminoacid identities with the XaPPTase gene or the ABC transporter gene of strain BAI3 of *X. oryzae* pv. *oryzae*** | **Overall nucleotide identities with gene PSEBR_a4280 of strain NFM421 of *Pseudomonas brassicacearum*** |
| PPTASEF  and  PPTASER | Strain CFBP2431 *Pseudomonas corrugata* | 41% | 40% | 84% |
|  | Strain CFBP5593 *Pseudomonas brassicacearum* | 42% | 39% | 100% |
|  | Strains CFBP4642 *Xanthomonas cassavae* | 45% | 86% | 39% |
|  | Strains CFBP1192 *Xylophilus ampelinus* | 45% | 45% | 41% |
|  | All 14 strains of *Xanthomonas* *oryzae* pv. *oryzae* | not sequenced | not sequenced | not sequenced |
|  | All 6 strains of *Xanthomonas oryzae* pv. *oryzicola* | not sequenced | not sequenced | not sequenced |
|  | Strain CFBP2539 of *Xanthomonas transluscens* pv. *secalis* | not sequenced | not sequenced | not sequenced |
|  | All 4 strains of *Xanthomonas albilineans* | not sequenced | not sequenced | not sequenced |
| ABCF  and  ABCR | All 5 African strains of *Xanthomonas* *oryzae* pv. *oryzae* | not sequenced | not sequenced | not sequenced |
|  | Strain UPB497 of *Xanthomonas oryzae* pv. *oryzicola* | 91% | 92% | _ |
|  | Strain CFBP2286 of *Xanthomonas oryzae* pv. *oryzicola* | 86% | 100% | _ |
|  | Strain CFBP2539 of *Xanthomonas transluscens* pv. *secalis* | 91% | 93% | _ |
|  | All 4 strains of *Xanthomonas albilineans* | not sequenced | not sequenced | not sequenced |
| DpgB2  and  DpgB7R | All 5 African strains of *Xanthomonas* *oryzae* pv. *oryzae* | not sequenced | not sequenced | not sequenced |
|  | Strain UPB497 of *Xanthomonas oryzae* pv. *oryzicola* | not sequenced | not sequenced | not sequenced |
|  | Strain CFBP2539 of *Xanthomonas transluscens* pv. *secalis* | not sequenced | not sequenced | not sequenced |
|  | Strains CFBP7063 and CFBP1943 of *Xanthomonas albilineans* | not sequenced | not sequenced | not sequenced |
| DpgB2  and  DpgC1R | Strains CFBP7063 and CFBP1943 of *Xanthomonas albilineans* | not sequenced | not sequenced | not sequenced |
|  |  |  |  |  |
|  |  |  |  |  |
|  |  |  |  |  |
| DpgB7  and  DpgC1R | All 5 African strains of *Xanthomonas* *oryzae* pv. *oryzae* | not sequenced | not sequenced | not sequenced |
|  |  |  |  |  |
|  | Strain CFBP2539 of *Xanthomonas transluscens* pv. *secalis* | not sequenced | not sequenced | not sequenced |
|  | Strains CFBP7063 and CFBP1943 of *Xanthomonas albilineans* | not sequenced | not sequenced | not sequenced |
| DpgB7  and  DpgC5R | All 5 African strains of *Xanthomonas* *oryzae* pv. *oryzae* | not sequenced | not sequenced | not sequenced |
|  | Strain UPB497 of *Xanthomonas oryzae* pv. *oryzicola* | not sequenced | not sequenced | not sequenced |
|  | Strain CFBP2539 of *Xanthomonas transluscens* pv. *secalis* | not sequenced | not sequenced | not sequenced |
|  | Strains CFBP7063 and CFBP1943 of *Xanthomonas albilineans* | not sequenced | not sequenced | not sequenced |
| DpgC5  and  HpgT2R | All 5 African strains of *Xanthomonas* *oryzae* pv. *oryzae* | not sequenced | not sequenced | not sequenced |
|  | Strain UPB497 of *Xanthomonas oryzae* pv. *oryzicola* | not sequenced | not sequenced | not sequenced |
|  | Strain CFBP2539 of *Xanthomonas transluscens* pv. *secalis* | not sequenced | not sequenced | not sequenced |
|  | Strains CFBP7063 and CFBP1943 of *Xanthomonas albilineans* | not sequenced | not sequenced | not sequenced |
| DpgC1  and  HpgT2R | All 5 African strains of *Xanthomonas* *oryzae* pv. *oryzae* | not sequenced | not sequenced | not sequenced |
|  | Strain UPB497 of *Xanthomonas oryzae* pv. *oryzicola* | not sequenced | not sequenced | not sequenced |
|  | Strain CFBP2539 of *Xanthomonas transluscens* pv. *secalis* | not sequenced | not sequenced | not sequenced |
|  | Strains CFBP7063 and CFBP1943 of *Xanthomonas albilineans* | not sequenced | not sequenced | not sequenced |
| DaT1  and  DaT2R |  |  |  |  |
|  | Strain UPB497 of *Xanthomonas oryzae* pv. *oryzicola* | not sequenced | not sequenced | not sequenced |
|  | Strain CFBP2539 of *Xanthomonas transluscens* pv. *secalis* | not sequenced | not sequenced | not sequenced |
|  | Strains CFBP7063 and CFBP1943 of *Xanthomonas albilineans* | not sequenced | not sequenced | not sequenced |
