# Supplementary material for: Genome mining reveals the genus Xanthomonas to be a promising reservoir for new bioactive non-ribosomally synthesized peptides
Source: BMC Genomics. 2013 Sep 27;14:658. doi: 10.1186/1471-2164-14-658 (PMC3849588; doi:10.1186/1471-2164-14-658)
Supplement: Additional file 6 — Collection of strains screened for the presence of XaPPTase gene and genes associated with NRPS in META-B. [file 1471-2164-14-658-S6.docx]

**Additional file 6: Collection of strains screened for the presence of XaPPTase gene and genes associated with NRPS in META-B**

| **Strains** | **No.** | **Geographic origins** | **Hosts** |
| --- | --- | --- | --- |
| *Xanthomonas fragariae* | CFBP6766 | United States | *Fragaria* sp. |
| *Xanthomonas horturum* pv. *pelargonii* | CFBP2533 | New Zealand | *Pelargonium peltatum* |
| *Xanthomonas populi* | CFBP1817 | France | *Populus euramericana* |
| *Xanthomonas arboricola* pv. *pruni* | CFBP3894 | New Zealand | *Prunus salicina* |
| *Xanthomonas arboricola* pv. *corylina* | CFBP1159 | United States | *Corylus maxima* |
| *Xanthomonas arboricola* pv*. juglandis* | CFBP1022 | France | *Juglans regia* |
| *Xanthomonas cassavae* | CFBP4642 | Malawi | *Manihot esculenta* |
| *Xanthomonas codiaei* | CFBP4690 | United States | *Codiacum varieganum* |
| *Xanthomonas cynarae* | CFBP4188 | France | *Cynara scolymus* |
| *Xanthomonas cucurbitae* | CFBP2542 | New Zealand | *Cucurbita maxima* |
| *Xanthomonas oryzae* pv. *oryzae* | CFBP7088 | Korea | *Oryza sativa* |
| *Xanthomonas oryzae* pv. *oryzae* | 12842 | Japan | *Oryza sativa* |
| *Xanthomonas oryzae* pv. *oryzae* | CFBP7203 | Philipines | *Oryza sativa* |
| *Xanthomonas oryzae* pv. *oryzae* | CFBP7206 | Philipines | *Oryza sativa* |
| *Xanthomonas oryzae* pv. *oryzae* | CFBP7212 | Chine | *Oryza sativa* |
| *Xanthomonas oryzae* pv. *oryzae* | CFBP7213 | Indonesia | *Oryza sativa* |
| *Xanthomonas oryzae* pv. *oryzae* | CFBP7230 | India | *Oryza sativa* |
| *Xanthomonas oryzae* pv. *oryzae* | CFBP7234 | Columbia | *Oryza sativa* |
| *Xanthomonas oryzae* pv. *oryzae* | CFBP7235 | Columbia | *Oryza sativa* |
| *Xanthomonas oryzae* pv. *oryzae* | CFBP1947 | Cameroun | *Oryza sativa* |
| *Xanthomonas oryzae* pv. *oryzae* | CFBP1949 | Mali | *Oryza sativa* |
| *Xanthomonas oryzae* pv. *oryzae* | 12897 | Burkina Faso | *Oryza sativa* |
| *Xanthomonas oryzae* pv. *oryzae* | 12898 | Burkina Faso | *Oryza glaberrima* |
| *Xanthomonas oryzae* pv. *oryzae* | 12901 | Mali | *Oryza sativa* |
| *Xanthomonas oryzae* pv. *oryzicola* | CFBP7109 | Philipines | *Oryza sativa* |
| *Xanthomonas oryzae* pv. *oryzicola* | UPB497 | Malaysia | *Oryza sativa* |
| *Xanthomonas oryzae* pv. *oryzicola* | CFBP2286 | Malaysia | *Oryza sativa* |
| *Xanthomonas oryzae* pv. *oryzicola* | 12902 | Mali | *Oryza sativa* |
| *Xanthomonas oryzae* pv. *oryzicola* | 12903 | Mali | *Oryza sativa* |
| *Xanthomonas oryzae* pv. *oryzicola* | 12904 | Mali | *Oryza sativa* |
| *Xanthomonas vasicola* pv. *holcicola* | CFBP2543 | New Zealand | *Sorghum vulgare* |
| *Xanthomonas pisi* | CFBP4643 | Japan | *Pisum sativum* |
| *Xanthomonas melonis* | CFBP4644 | Brazil | *Cucumis melo* |
| *Xanthomonas translucens* pv. *graminis* | CFBP3524 | Switzerland | *Dactylis glomerata* |
| *Xanthomonas translucens* pv. *poae* | CFBP2057 | Switzerland | *Poa* sp. |
| *Xanthomonas translucens* pv. *secalis* | CFBP2539 | Canada | *Secale cereale* |
| *Xanthomonas hyacinthi* | CFBP1156 | The Netherlands | *Hyacinthus orientalis* |
| *Xanthomonas sacchari* | CFBP4641 | Guadeloupe | *Saccharum officinarum* |
| *Xanthomonas theicola* | CFBP4691 | Japan | *Camellia sinensis* |
| *Xanthomonas campestris* pv. *campestris* | CFBP5241 | United Kingdom | *Brassica olaracea* |
| *Xanthomonas campestris* pv. *raphani* | CFBP5827 | United States | *Raphanus sativus* |
| *Xanthomonas campestris* pv. *armoraciae* | 756C | ? | *B. oleracea var. capitata* |
| *Xanthomonas campestris* pv. *incanae* | CFBP2527 | United States | *Matthiola incana* |
| *Xanthomonas vasicola* pv. *musacearum* | CFBP7122 | Ethiopia | *Musa sp.* |
| *Xanthomonas* sp. "false red stripe" | XfrsBRA69 | Brazil | *Saccharum officinarum* |
| *Xylophilus ampelinus* | CFBP1192 | Crete | *Vitis vinifera* |
| *Agrobacterium tumefaciens* | CFBP2413 | ? | ? |
| *Acidovorax valerianellae* | CFBP4730 | France | *Valerianella locusta* |
| *Acidovorax anthurii* | CFBP3232 | Martinique | *Anthurium* *sp*. |
| *Acidovorax avenae* subsp*. avenae* | CFBP1201 | Réunion island | *Saccharum officinarum* |
| *Acidovorax avenae* subsp*. cattleyae* | CFBP2423 | United States | ? |
| *Acidovorax avenae* subsp*.citrulli* | CFBP4459 | United States | *Citrullus lanatus* |
| *Acidovorax delafieldii* | CFBP2442 | United States | ? |
| *Acidovorax facilis* | CFBP2441 | United States | ? |
| *Acidovorax konjaci* | CFBP4460 | Japan | *Amorphophallus rivieri* cv. *durieu* |
| *Acidovorax temperans* | CFBP3610 | Sweden | ? |
| *Clavibacter michiganensis* subsp. *michiganensis* | CFBP4999 | Hungary | *Lycopersicon esculentum* |
| *Burkholderia andropogonis* | CFBP2421 | United States | *Sorghum vulgare* |
| *Dickeya diffenbaciae* | CFBP2051 | United States | *Diffenbachia* sp. |
| *Pectobacterium atrosepticum* | CFBP1526 | United Kingdom | *Solanum tuberosum* |
| *Pseudomonas corrugata* | CFBP2431 | United Kingdom | *Lycopersicon esculentum* |
| *Pseudomonas cichorii* | CFBP2101 | ? | *Cichorium endivia* |
| *Pseudomonas brassicacearum* | CFBP5593 | France | *Brassica napus* |
| *Pseudomonas savastanoï* pv*. phaseolicola* | CFBP1390 | Canada | *Phaseolus vulgaris* |
| *Pseudomonas syringae* pv*. syringae* | CFBP1392 | United Kingdom | *Syringa vulgaris* |
| *Erwinia amylovora* | CFBP1430 | France | *Crataegus oxyacantha* |
| *Ralstonia solanacearum* | CFBP1960 | Algeria | *Capsicum annuum* |
| *Dickeya sp.* | CFBP1537 | Australia | *Saccharum officinarum* |
| *Herbaspirillum rubrisubalbicans* | CFBP1202 | Jamaica | *Saccharum officinarum* |
| *Xanthomonas albilineans* | CFBP7063 | Guadeloupe | *Saccharum officinarum* |
| *Xanthomonas albilineans* | CFBP1943 | Burkina Faso | *Saccharum officinarum* |
| *Xanthomonas albilineans* | CFBP2378 | Réunion island | *Saccharum officinarum* |
| *Xanthomonas albilineans* | CFBP2383 | South Africa | *Saccharum officinarum* |
| *Xanthomonas axonopodis* | CFBP4924 | Colombia | *Axonopus scoparius* |
| *Xanthomonas axonopodis* pv*. manihotis* | CFBP1865 | Colombia | *Manihot esculenta* |
| *Xanthomonas axonopodis* pv. *alfalfae* | CFBP3836 | Sudan | *Medicago sativa* |
| *Xanthomonas axonopodis* pv. *glycines* | CFBP1559 | France | *Glycine* *hispida* |
| *Xanthomonas axonopodis* pv. *malvacearum* | CFBP2530 | Sudan | *Gossypium hirsutum* |
| *Xanthomonas axonopodis* pv*. citrumelo* | CFBP3371 | ? | *Citrus* sp. |
| *Xanthomonas axonopodis* pv*. citri* | CFBP3369 | United States | *Citrus* sp. |
| *Xanthomonas axonopodis* pv. *allii* | CFBP6369 | Réunion island | *Allium cepa* |
| *Xanthomonas axonopodis* pv*. begoniae* | CFBP1421 | New Zealand | *Begonia* sp. |
| *Xanthomonas axonopodis* pv. *aurantifolii* | CFBP3528 | Argentina | *Citrus* *limon* |
| *Xanthomonas axonopodis* pv*. vesicatoria* | CFBP5618 | United States | *Lycopersicon esculentum* |
| *Xanthomonas axonopodis* pv*. vasculorum* | CFBP5823 | Australie | *Saccharum officinarum* |
| *Xanthomonas axonopodis* pv*. vasculorum* | CFBP1289 | Réunion island | *Saccharum officinarum* |
| *Xanthomonas axonopodis* pv*. vasculorum* | CFBP1215 | Kenya | *Saccharum officinarum* |
| *Xanthomonas axonopodis* pv*. vasculorum* | CFBP5830 | Malaysia | *Saccharum officinarum* |
| *Xanthomonas axonopodis* pv*. vasculorum* | CFBP5698 | Trinidad | *Saccharum officinarum* |
| *Xanthomonas axonopodis* pv. *phaseoli* (GL1) | CFBP6987 | United States | *Phaseolus vulgaris* |
| *Xanthomonas axonopodis* pv. *phaseoli* (GL2) | CFBP6989 | Réunion island | *Phaseolus vulgaris* |
| *Xanthomonas axonopodis* pv. *phaseoli* (GL3) | CFBP6992 | Réunion island | *Phaseolus vulgaris* |
| *Xanthomonas axonopodis* pv. *phaseoli* variant *fuscans* | CFBP4834 | France | *Phaseolus vulgaris* |
| *Xanthomonas axonopodis* pv*. vasculorum* | CFBP5696 | Réunion island | *Thysanolena maxima* |
| *Xanthomonas axonopodis* pv. *phaseoli* (GL1) | CFBP6164 | Romania | *Phaseolus vulgaris* |
