# Supplementary material for: Genome mining reveals the genus Xanthomonas to be a promising reservoir for new bioactive non-ribosomally synthesized peptides
Source: BMC Genomics. 2013 Sep 27;14:658. doi: 10.1186/1471-2164-14-658 (PMC3849588; doi:10.1186/1471-2164-14-658)
Supplement: Additional file 7 — Annotated nucleotide sequence of the regions encoding A-domains and/or NRPS associated genes in the contigs of strain XaS3. [file 1471-2164-14-658-S7.doc]

**Additional file 8 : Annotated nucleotide sequence of the regions encoding A-domains and/or NRPS associated genes in the contigs of strain XaS3.**

**List of these contigs:**

**- Contig G103 (length = 227,269 bases; annotation of the first 13,400 bases on page 1)**

**- Contig G106 (length = 170,046 bases; annotation of the first 24,000 bases on page 6)**

**- Contig G108 (length = 154,304 bases; annotation of the last 14,000 bases on page 16)**

**- Contig G111 (length = 139,076 bases; annotation of the last 4,400 bases on page 23)**

**- Contig G129 (length = 13,594 bases; annotation on page 25)**

**- Contig G130 (length = 12,805 bases; annotation on page 32)**

**- Contig G132 (length = 10,718 bases; annotation on page 37)**

**- Contig G134 (length = 10,162 bases; annotation on page 41)**

**- Contig G135 (length = 9,027 bases; annotation on page 45)**

**- Contig G137 (length = 7,985 bases; annotation on page 49)**

**- Contig G140 (length = 5,840 bases; annotation on page 51)**

**- Contig G143 (length = 5,242 bases; annotation on page 52)**

**- Contig G146 (length = 4,673 bases; annotation on page 54)**

**- Contig G147 (length = 4,580 bases; annotation on page 56)**

**- Contig G149 (length = 4,434 bases; annotation on page 58)**

**- Contig G151 (length = 4,032 bases; annotation on page 60)**

**- Contig G167 (length = 877 bases; annotation on page 63)**

**- Contig G169 (length = 824 bases; annotation on page 63)**

LOCUS Contig G103 13400 bp DNA linear BCT 30-JAN-2012

DEFINITION Xanthomonas spp. strain XaS3 genomic region encoding NRPS.

SOURCE Xanthomonas spp. strain XaS3

ORGANISM Xanthomonas spp. strain XaS3

Bacteria; Proteobacteria; Gammaproteobacteria; Xanthomonadales;

Xanthomonadaceae; Xanthomonas.

REFERENCE Contig G103 (bases 1 to 13400)

AUTHORS Royer et al. Submitted to BMC Genomics.

TITLE Genome mining indicates that the genus Xanthomonas is a promising

reservoir for new bioactive non-ribosomally synthesized peptides

JOURNAL Submitted to BMC Genomics.

REFERENCE 2 (bases 1 to 13400)

FEATURES Location/Qualifiers

source 1..13400

/organism='Xanthomonas spp.'

/mol_type='other DNA'

/strain='XaS3'

/isolation_source='sugarcane leaf water droplets'

/host='Sugarcane'

/db_xref='taxon: GPE 39'

/country='Guadeloupe'

/collection_date='2003'

/collected_by='Rosiane BOISNE-NOC'

/note='[cultured bacterial source]'

gene <2..3301

/gene ='NRPS'

CDS <2..3301

/gene ='NRPS'

/note='incomplete NRPS, no start codon'

/codon_start=1

/translation='IRSTLGLELPLATLFAQPRLRELAQALDNTATSTLPAIVPADRHAPLPLSFAQQRLWFLNQLDPRTGTTYLMHDGVRLSGTLDVTALRRALDRIVARHDALRTHFSSVDDVPCQIVAAPFALALPLIDVSDEADTEAAARAHALAEASTGFDLANGPLIRGRLLRLAEHEHVLLLSMHHIISDGWSIGVLIEELGALYTAFVSDAPDPLPPLPIQYADYAAWQRRWIDDQQQQRQLGYWCDQLRDAPALLELPTDRPRPPVQDTAGDDVELVLDADLSMRLQALALEQGTTSFALLLAAWAVLLTRYSGQTDVVIGTPSAGRRHSELEPLIGFFVNTLPLRIDLSARPSFIALLEQVQRNLVAAQANESIPYERIIEAVGPARSLAHAPLCQAMFSSDTTPSRALDLPGLQLCAYPNEHRVAQFDLSLDMQIAPSRIGGVLRYATALFDRNTMQGYLAHYARLLAALVDAPTLAVDRHPLVDARQWHALQQWNRTTALLQAPTTLHHAFQAQARRTPDAIAVIDGTQHLCYAELDAHSDRIAQHLVQAGVAPGTCVATLLPRSAALVAAQLGILKAGAAYVPLDPQQPAARHAQLVDACQARAIVHAPGEAPPWANVPCLPITLDMIAVMPFAAPPLPARAPAYVMYTSGSSGTPKGVVIPHQAVLNLVHAPDYAHWQAQDRFAFASNPAFDSSTLEVWAPLLSGGSVVVVPQEVMLDPSVLADFARDHAITVLILVAGVLRAYASELARALPTLRYLITGGDIADPQALAMLLHNNPPQTLLQTYGPTETTQFVTAMALSDVADDGRRIPIGKPIGNLRVHVLDRHRQAVPVGMQGELHIAGLGLALGYLGQPGLTADHFVPDPFSEEPGARMYRTGDLGRWRADGLLECLGRGDAQSKIRGFRLEPGEIEAALQRHRQISQALVRVREDMPGQRRLVAYVIGTDAVSAPEPSALRRHLAASLPEYMLPDAYVDMQAWPLTANGKVDVRALPAPDDAQRGIAEVEPPQGESECALAQIWCELLGVGSVNRHDNFFDIGGHSLLAVQLTTRIQAKLERRLPLSRLFAEPTLARMAAAMADSHVASSAPIAALVDRSRYYD'

gene 3523..5349

/gene ='NRPS'

CDS 3523..5349

/gene ='NRPS'

/note='complete NRPS, ortholog of XALc_1055'

/codon_start=1

/translation='MPIAHHVFHHMPAHPTSRQQADPQLGGPNARDSHALVVRAHGTRPPLFVVPTGAGDIAYAFALAEHLEADIPVYALPWPDPLPTMMETLAAQMVETIRAVQPHGPYHLFGDSSGGLLAYAIAQHLGIHNESVAFLGLLDCHCPDRAPDPMTLDETCKQHLLARMKLLLQRTPADHDAATQAALDELVAKAEQASSQALAAYAHADRLLNDLAARAQTSVEQIFHTSRMRATFERMWPTYWVQRLAAHCPLTVFHASEPFPEDTTLGWTRLLPARQVQSVAVPGHHGTLIEAEHLPRLGRCLSSALCACGSTVAASAYEPAFTLQSARPTAPVVVCVPGAGDSMTGFVDLSTALGQSCHVIGMQPRGTDGSGPPFGSVELAAQHYLDALPSNANNAAALHLIGHSFGGWVVLEMALRLHALGRPPASLTLIDTRPPHRALAPHDCKRDIIVDYFLDALQMRVRTPLNIDRQALHRLGQDALIQSVHRVMVEHGLMPPRSRSEAIRGSLTTFAQCCRTTYTPAKPYPGTLHLVLVDNALHEKTQLADTYQGLSEAWTPHAADLRPWHGPGNHMTVLAKPQSQTLAEWWMSNVRDAATATTASALPAAAVGR'

gene complement (5575.. 6564)

/gene ='AraC'

CDS complement (5575.. 6564)

/gene ='AraC'

/note='transcriptional regulator, ortholog of XALc_1054'

/codon_start=1

/translation='MVEARVAPVSSVFDAFFTSQVHPGERVPRWLDWMARSIGVDRPGIRARRKFSFSPVQPVFDGCINQLRLGDVHLAKVLATGHEFHLTLADNDDERPRILVALQQKGASRMVCDGVSTALRAGELAVLQIDRQFCFASEASVENYFIWFAQPRRAMKLVNEVASQRLCGPAPMQRLACSLIERLFSEPGLCNSDSEHFLAQALTNLLELTFKESSDVQPPLEPNRPSREMVVQFVERNLRDPELSPESIARALGWSKRTVYRAFKSSDGESLNSYLWRRRVEQCAQELRGPGNQSITGIAYSFGFSSSPHFSRLFKQHMGASPLRYRRGDH'

gene 7272..7775

/gene ='Hp'

CDS 7272..7775

/gene ='Hp'

/note='Hypothetical protein, ortholog of ortholog of XOO_2168'

/codon_start=1

/translation='MADTGRSPESALQYHTIDHIALAVQDLEAAIALFRDQLGFTLTKRRHITGQRTGMLSAEMQHGAITFVLCQGTEPQSQVSRLIEHHGVGVAHIALRVDNTHAAVQRLRGRGLRFDTDVIQGDGLRQAFSSRDAATGLSFEFIERNGEAGFQESSVAELFAQLERSGAY'

gene 7808..9127

/gene ='TA'

CDS 7808..9127

/gene ='TA'

/note='Transaminase'

/codon_start=1

/translation='MHSPTAAHATGGNRLEVMNFLNEIASRHPDAISFVSGRPSNDFFDLQAWMQALPRFADHLARRHRLTPSQSLDTIAQYGATQGIVNDLIVAQLAIDEGVPASSERLLVTAGCQEALQLCVTELCREPGDVVLVRNPTYIGITGVADSQRIPITAFNRGDRADVGALAETLDALEAAGQRARLLYLVPDFDNPTGSVLARTQREAIIALCAARGVVILEDNPYGLFAFEAERLPTMRALDRHGCVIYLGTYSKTLCPGLRVGFAVLPPTLFGDACAADALQQALTQRKSFGTLNTGQIAQAIVGGVLLEHGGSLEALIQAPRAQYRRNRDRMLECLSKELGTLAADVQWNVPSGGFFLIVSLPFVFAEEEAARCARDYGVLVMPLCFFAIDGGWRQHVRLAYSHVATEAIVEGIARFARFVRDRLHALATAQTPLPLPDCA'

gene complement (9296..10429)

/gene ='DH'

CDS complement (9296..10429)

/gene ='DH'

/note= 'Dehydrogenase'

/codon_start=1

/translation='MQTLTCLDDVKALARRRVPRMFYDYVDAGSWSQSTLHANRRDFDALALRQRIGCDVARRSTAACMLGRQVSMPVALAPTGLAGLIWANGEILGARAAEAFGVPFVLSMMSICALEEVCAVLRAPCWFQLYPMRDRGIVAALIERATRAGCAALVLTLDVPFMGQRHADLRNGLSVPPRLKPAALLDFLAHPRWALGMLRTRRRCFGNLVDYAPAGNDLRTLSGWTAQQFDAGIGWEDVAWVRSRWRGTLVIKGVLDAEDARQAIAAGADALVVSNHGGRQLDGACSSVRALPAIAELAAGRADVHLDSGIRCGQDVLKALALGASATYIGRAWLYGLAALGQAGVAHVLALIQRELELTMALCGRTEIAAVDRGILLS'

gene 10731.. 12011

/gene ='DaT'

CDS 10731.. 12011

/gene ='DaT'

/note=' diaminobutyrate--2-oxoglutarate aminotransferase'

/codon_start=1

/translation='MSDPRLIFQRHESNVRSYCRSFDAMFVRASGSLMFDAQGRRYIDFLAGCGSLNYGHNDPDMAEALIGHLRNGGLALSLDMYSQAKHDFIDAFVERILKPRGLTHRLQFTGPTGTNAVEAALKLARKITGRHNVIAFSNAYHGLSMGALATTGNRHHRMELAHTGVTRLPYDGYLGESVDSAALLEGMLDDPSGGIDPPAAIILELVQGEGGLNVASTPWLRRIFAAARRHGALVIVDDVQAGCGRSGGFFSFDGLDLVPDLIVLSKSLSGFGLPFSLLLVAPEHDQWRPGQHNGTFRGNNHAMLTASVALHKFWADDTFATQAAARGQRVTEALTRIAAQVPGARVKGRGMFMGIDVGTTQLAASARAHAFKHGLVIESAGPNDEVLKVMAPLTTPDALLDEGLDILADAVLHACEQTQQVKLSRVA'

ORIGIN

1 CATTCGTAGCACGCTGGGATTGGAACTGCCGCTGGCGACGCTGTTCGCGCAACCACGTCT

61 GCGCGAGTTGGCCCAAGCTCTGGACAACACGGCCACCAGCACCCTGCCCGCCATCGTGCC

121 GGCAGATCGTCATGCGCCGCTACCGCTGTCCTTCGCGCAGCAACGGCTGTGGTTCTTGAA

181 CCAACTCGATCCACGCACCGGCACCACGTATCTGATGCATGACGGCGTGCGCCTGAGCGG

241 TACGCTGGATGTCACGGCGTTGCGCCGCGCATTGGATCGCATCGTCGCCCGCCACGATGC

301 CTTGCGTACACATTTTTCGAGCGTGGACGACGTTCCCTGTCAGATCGTCGCAGCGCCATT

361 TGCATTGGCGCTGCCGTTGATCGACGTGAGCGACGAAGCGGACACGGAGGCCGCCGCGCG

421 TGCACATGCCCTCGCCGAAGCCAGCACCGGCTTCGACCTCGCCAACGGGCCACTGATCCG

481 TGGCCGTCTGCTGCGCCTGGCCGAACATGAGCATGTATTGCTGCTCAGCATGCATCACAT

541 CATCTCAGACGGATGGTCCATCGGCGTCCTGATCGAGGAACTGGGCGCCTTGTATACCGC

601 GTTCGTCAGCGATGCGCCCGATCCGTTGCCGCCATTGCCGATCCAATACGCGGACTACGC

661 CGCCTGGCAACGTCGCTGGATCGATGACCAGCAGCAACAGCGCCAATTGGGTTACTGGTG

721 CGATCAGTTGCGCGATGCTCCGGCGCTGCTGGAATTGCCGACGGACCGGCCACGCCCGCC

781 GGTACAGGACACCGCAGGCGACGATGTGGAACTGGTGCTGGACGCAGACCTGAGCATGCG

841 CCTGCAGGCATTGGCGCTGGAGCAAGGCACCACGAGCTTCGCGCTGCTGCTGGCAGCGTG

901 GGCGGTGTTGTTGACGCGCTACAGCGGCCAAACCGATGTGGTGATCGGCACACCGTCGGC

961 CGGCCGTCGCCACAGCGAACTGGAACCGCTGATCGGCTTCTTCGTCAACACCTTGCCGCT

1021 GCGCATCGACCTGTCGGCTCGGCCCAGCTTCATTGCATTGCTGGAGCAGGTGCAGCGCAA

1081 CCTGGTGGCGGCGCAAGCCAACGAAAGCATCCCATACGAACGCATCATCGAAGCGGTAGG

1141 CCCGGCGCGCAGCCTGGCGCATGCACCGTTGTGCCAAGCGATGTTCTCCTCCGATACCAC

1201 GCCGTCGCGCGCACTCGACCTGCCGGGATTGCAACTGTGCGCCTACCCGAACGAGCATCG

1261 GGTCGCTCAATTCGATCTGTCGCTGGACATGCAGATTGCGCCATCGAGGATCGGCGGCGT

1321 GCTGCGTTACGCCACGGCGCTGTTCGATCGCAACACCATGCAGGGGTATCTCGCGCATTA

1381 CGCACGCCTGCTGGCCGCACTGGTCGATGCACCGACGCTGGCGGTGGACCGACACCCGCT

1441 CGTGGATGCGAGGCAATGGCACGCGTTGCAGCAATGGAACCGCACCACCGCCCTATTGCA

1501 GGCACCGACGACGCTGCACCATGCGTTCCAGGCGCAAGCCCGCCGCACACCGGACGCGAT

1561 CGCCGTCATCGACGGCACGCAGCACCTGTGTTACGCCGAACTGGACGCGCACAGCGACCG

1621 GATCGCGCAGCACCTGGTGCAGGCTGGCGTGGCCCCTGGCACATGCGTGGCGACGCTGCT

1681 GCCGCGCAGTGCCGCACTGGTCGCCGCGCAACTGGGCATCCTCAAGGCGGGCGCGGCGTA

1741 TGTGCCGTTGGATCCGCAGCAACCGGCGGCCCGACACGCTCAGTTGGTCGATGCCTGCCA

1801 GGCGCGTGCCATCGTGCATGCGCCCGGCGAGGCACCGCCATGGGCGAACGTCCCCTGTCT

1861 GCCGATCACGCTGGACATGATCGCGGTGATGCCATTCGCCGCGCCGCCACTGCCAGCCCG

1921 CGCACCGGCGTATGTCATGTACACCTCCGGTTCCAGCGGCACGCCCAAAGGCGTGGTCAT

1981 CCCGCATCAGGCCGTCCTCAACCTGGTGCATGCTCCGGACTACGCCCATTGGCAGGCGCA

2041 GGACCGTTTCGCATTCGCGTCCAACCCGGCGTTCGACTCCAGCACGCTGGAAGTGTGGGC

2101 GCCGTTGTTGAGCGGCGGCAGCGTGGTGGTCGTGCCGCAGGAGGTGATGCTCGACCCGAG

2161 CGTACTGGCAGACTTCGCCCGCGATCACGCCATCACCGTACTGATCCTGGTCGCCGGTGT

2221 GCTGCGTGCCTATGCGAGCGAACTGGCACGCGCACTGCCGACCTTGCGCTATCTGATTAC

2281 CGGTGGCGACATCGCCGATCCGCAGGCGCTCGCCATGCTGCTACACAACAACCCGCCACA

2341 GACCTTGCTGCAGACCTATGGCCCGACCGAAACCACGCAGTTCGTCACCGCCATGGCGTT

2401 GAGCGACGTGGCGGACGATGGCCGGCGCATTCCCATCGGCAAACCGATCGGCAATCTGCG

2461 CGTACATGTCCTGGACCGCCATCGTCAGGCCGTGCCGGTGGGGATGCAGGGCGAACTGCA

2521 TATCGCCGGCCTGGGCCTGGCGCTGGGTTATTTGGGTCAACCAGGGTTGACCGCCGACCA

2581 CTTCGTCCCCGATCCCTTCAGCGAAGAACCGGGAGCGCGCATGTACCGGACCGGCGACCT

2641 CGGCCGCTGGCGTGCCGACGGCCTGCTCGAATGCCTGGGCCGAGGTGATGCGCAAAGCAA

2701 GATCCGCGGGTTCCGGCTGGAACCGGGCGAGATCGAGGCGGCGTTGCAGCGCCATCGGCA

2761 GATCAGTCAAGCGCTGGTGCGCGTGCGCGAGGACATGCCGGGGCAACGTCGTCTGGTGGC

2821 CTATGTGATCGGGACCGATGCCGTATCGGCACCGGAGCCGAGCGCACTGCGTCGCCACTT

2881 GGCCGCCAGCTTGCCCGAGTACATGCTGCCAGACGCCTACGTGGACATGCAGGCCTGGCC

2941 ACTGACCGCCAACGGCAAGGTGGATGTGCGTGCGCTGCCTGCCCCCGACGACGCCCAGCG

3001 TGGCATCGCCGAGGTCGAGCCGCCACAGGGCGAGAGCGAATGTGCACTGGCGCAGATCTG

3061 GTGCGAATTGCTCGGCGTCGGCAGTGTCAACCGACACGACAACTTCTTCGACATCGGCGG

3121 CCATTCGTTACTGGCGGTGCAACTGACCACGCGCATTCAGGCAAAGCTCGAACGCCGACT

3181 GCCGCTGTCGCGTTTGTTCGCCGAGCCCACGCTCGCGCGCATGGCTGCGGCAATGGCCGA

3241 CAGCCACGTCGCTTCCAGCGCCCCGATCGCCGCATTGGTGGACCGTAGCCGTTACTACGA

3301 CTGATCCCGAAGACCCGGACACGCACTTGTCTGACCTGGATCCCATGCCAGGCAAGTGCG

3361 TGTCACATCGAGTCAAGCGCACCCGCGCGGCAACGGAGAAAAAGCGCACTCACACTGCGC

3421 TTTCGGTGCATCGCACGCAATGGGTCTACGTGCGAAGCGTCGCTGGCACCGCTTCTTCTA

3481 ACTTGGCGAACATTCGCCGACAGCGCTATGGTTGCCGCTGAACTGCCCATCGCCCACCAC

3541 GTTTTCCATCACATGCCCGCACATCCCACATCACGCCAGCAGGCCGATCCGCAGCTCGGC

3601 GGCCCCAACGCACGGGACAGCCATGCGCTCGTCGTCCGCGCCCACGGGACACGTCCGCCG

3661 CTGTTCGTAGTGCCGACCGGCGCCGGAGATATCGCCTACGCCTTCGCGCTGGCAGAACAT

3721 CTCGAAGCGGACATCCCGGTCTATGCCCTGCCCTGGCCAGACCCGTTACCGACCATGATG

3781 GAAACGCTGGCCGCACAGATGGTCGAAACGATCCGCGCAGTGCAGCCACATGGCCCGTAT

3841 CACCTGTTCGGCGACTCCTCCGGCGGCTTGCTCGCCTATGCCATCGCCCAGCACTTGGGC

3901 ATACACAACGAGTCGGTGGCCTTCCTGGGCCTGCTCGATTGCCACTGCCCTGACCGCGCG

3961 CCGGATCCCATGACGTTGGATGAGACGTGCAAACAGCACCTGCTCGCACGCATGAAACTG

4021 CTACTGCAGCGCACACCGGCGGACCACGACGCTGCCACCCAGGCGGCGCTCGACGAGCTG

4081 GTTGCGAAAGCAGAACAGGCATCGTCACAAGCGCTGGCCGCGTATGCCCACGCGGATCGG

4141 CTGTTGAACGATCTGGCCGCGCGCGCGCAGACCAGCGTGGAGCAGATCTTCCACACCAGC

4201 CGGATGCGTGCAACATTCGAAAGGATGTGGCCCACGTATTGGGTACAACGGCTTGCCGCG

4261 CACTGCCCACTGACGGTATTCCATGCGTCCGAGCCGTTCCCCGAGGACACGACGCTCGGC

4321 TGGACACGGCTGTTGCCGGCACGCCAGGTGCAATCGGTGGCCGTCCCCGGCCATCATGGC

4381 ACGCTGATCGAAGCCGAACATCTACCGCGCCTGGGTCGGTGTCTGTCGAGCGCGCTCTGC

4441 GCGTGCGGGTCCACGGTTGCGGCATCGGCTTACGAACCCGCATTCACGCTGCAATCGGCG

4501 CGCCCGACCGCGCCCGTGGTCGTCTGTGTGCCGGGTGCGGGCGACAGCATGACAGGCTTC

4561 GTCGACCTGAGCACCGCGCTGGGACAGAGCTGCCATGTGATCGGCATGCAACCGCGTGGC

4621 ACCGACGGCAGTGGCCCGCCGTTCGGTTCGGTGGAACTTGCCGCACAACACTATCTCGAT

4681 GCCTTGCCCAGCAACGCCAACAATGCCGCTGCCCTGCATCTGATCGGTCATTCGTTCGGC

4741 GGCTGGGTGGTGCTGGAGATGGCATTACGCCTGCATGCCCTCGGCCGCCCGCCCGCCAGC

4801 CTGACCCTGATCGACACCCGTCCTCCACATCGCGCGCTGGCACCGCACGACTGCAAGCGC

4861 GACATCATCGTGGACTATTTCCTCGACGCATTGCAGATGCGTGTACGCACGCCACTGAAC

4921 ATCGACCGCCAGGCGCTGCATCGTCTCGGCCAGGATGCGTTGATTCAATCGGTGCATCGG

4981 GTCATGGTGGAACACGGACTGATGCCGCCACGCTCGCGCAGCGAGGCGATCCGCGGCAGC

5041 CTGACCACGTTCGCCCAGTGCTGCCGAACCACCTACACGCCGGCCAAGCCCTATCCAGGC

5101 ACGTTGCATCTGGTCCTTGTCGACAACGCCCTGCACGAAAAAACGCAACTGGCCGACACG

5161 TATCAGGGACTGAGCGAAGCCTGGACCCCCCATGCCGCCGATCTACGCCCCTGGCACGGC

5221 CCCGGCAATCACATGACCGTGCTTGCCAAACCGCAGAGTCAGACCTTGGCCGAGTGGTGG

5281 ATGTCCAACGTGCGCGATGCAGCGACCGCCACAACCGCCTCCGCCCTGCCTGCTGCAGCA

5341 GTCGGCAGGTAATCGCACGTTTCGGGTTACCGGGACGACGCATCGGCTTCGCCCGGCACA

5401 GCGCAGGAGCGTGACCGCGCGAAGCAGGAGGCAGACGCCTCCCTCGACAATGGCGGGCAG

5461 GCGCGGTGACACGTGGCATTCGGAACCTGAGCGTGCGAAAACCGCAAGCGCACGCCTTGC

5521 GTCCAAGGCAACACGGCACCAGCGCCGTTGCCATCGGCGCCCCGCGCGGACTCAATGATC

5581 GCCGCGACGATACCGCAACGGCGAGGCGCCCATGTGCTGCTTGAACAAGCGACTGAAATG

5641 CGGAGAGCTACTGAAGCCGAAAGAATAGGCAATCCCGGTAATGCTCTGGTTGCCTGGCCC

5701 ACGCAACTCTTGCGCGCACTGTTCGACCCGTCGTCGCCACAAATAACTGTTCAGACTCTC

5761 GCCGTCGCTGCTCTTGAATGCGCGATAGACCGTGCGTTTGCTCCAGCCCAGCGCACGCGC

5821 GATGCTCTCCGGGGAAAGCTCCGGGTCGCGCAAATTGCGTTCGACGAACTGCACCACCAT

5881 CTCCCGCGACGGCCGGTTGGGTTCCAACGGTGGCTGCACGTCGCTGGACTCTTTGAACGT

5941 CAGTTCAAGCAAATTGGTCAGGGCTTGCGCGAGGAAGTGCTCGCTGTCGGAATTGCACAG

6001 CCCCGGCTCGCTGAACAAACGCTCGATCAGACTGCATGCCAGCCGCTGCATCGGCGCCGG

6061 TCCGCACAGCCGCTGCGATGCGACCTCATTGACCAGCTTCATCGCGCGCCGCGGCTGCGC

6121 AAACCAAATGAAGTAATTTTCGACACTGGCCTCACTGGCGAAACAAAACTGCCGATCGAT

6181 CTGCAACACGGCCAGCTCGCCGGCGCGCAGTGCGGTCGAGACGCCATCGCACACCATCCG

6241 GCTCGCGCCCTTTTGCTGCAGCGCCACCAGGATGCGCGGCCGCTCATCGTCGTTGTCGGC

6301 CAGAGTCAGATGGAATTCATGTCCTGTCGCCAACACCTTGGCAAGATGCACATCTCCAAG

6361 GCGTAGCTGGTTGATACACCCGTCGAACACCGGTTGCACAGGCGAAAAAGAGAATTTGCG

6421 CCGCGCCCGTATGCCAGGCCGATCCACGCCGATCGAACGCGCCATCCAATCGAGCCAGCG

6481 CGGCACGCGCTCGCCAGGATGCACTTGCGAAGTAAAAAATGCATCGAAGACGCTGGACAC

6541 GGGAGCAACCCGTGCTTCCACCACATCACGAGGAGACTCTCGCTGCACTCGGCATATCTT

6601 AGCCATCTCATCCGGCCTCAGAAGTCAGTTTCTGGGCGGCGCATGCTATGCAGAAATTGC

6661 GTCCCTCTTATGACAAATCTCGCACAAGAGAATTGTATCGAAACACAAAATTCATTTCTT

6721 AAACATAAGTTTCATGTAAATAAAATTGATTTTAAACGTTGCCACATGCCCCCCCCTTCC

6781 CGACTGCGCCTGGCGGCATTGCAACAAACCGCGCCACGGCTGACATGAAGACGCAGGGCA

6841 AACTGAAACAACCGCTCTCCGACATCGAGACAGCGCAAAAATCGAACAGGCGAACGCACG

6901 CCCCACTGGCTGCCGCGACGCATGGACGCAGATCGTCGGCGACGAACACGCACGCGGTCT

6961 CCAGCACCTGAGGTTGCCGCGCAGCAAGCAGTGCCGGCCCTGCCTATCCGAAAATGACGG

7021 CCGCGCACGCATCCACAAATCTGCAGGCGAAATTGTCATCGCAGCTTCGCATTGGCAGCG

7081 TAGCCTGACCGGGCATGCGCAACTTCCCGTTAGATGCGAACCGCGCAGAACGTCTGCCGC

7141 ATCCCAAGTTGCCATGCCACTCATTCAGTGAACACACGTTGGCCGACGGTCTCGCCGCAC

7201 AGACAGATGAATCTCGCGCACGGCATGACATCGATTATCGGCACACCTTGCGACAACGAA

7261 CGGAGCACTCCATGGCCGATACTGGCAGATCCCCCGAAAGCGCGTTGCAGTACCACACGA

7321 TCGATCACATCGCGCTTGCCGTACAGGACCTTGAAGCGGCCATCGCGCTGTTTCGCGACC

7381 AACTTGGCTTCACCTTGACCAAGCGCCGCCACATCACCGGGCAACGTACCGGCATGCTCT

7441 CGGCGGAAATGCAACATGGCGCGATCACCTTCGTCCTGTGTCAAGGCACGGAACCGCAGT

7501 CTCAGGTCTCGCGCCTGATCGAACATCATGGCGTGGGCGTGGCCCACATCGCCTTGCGCG

7561 TGGACAATACGCACGCCGCTGTACAGCGTCTGCGTGGGCGCGGCTTGCGCTTCGACACCG

7621 ATGTGATCCAGGGCGATGGATTGCGCCAGGCATTTTCCAGCCGCGACGCGGCCACCGGAC

7681 TGAGCTTCGAATTCATCGAACGCAATGGCGAAGCCGGCTTCCAGGAGTCTTCCGTCGCCG

7741 AACTCTTCGCGCAACTAGAACGCAGCGGCGCCTATTGATCGATCCACGTCCACGCCGGAG

7801 CCTGTCCATGCACAGCCCCACTGCCGCCCATGCCACTGGCGGCAATCGCCTGGAGGTCAT

7861 GAATTTTCTGAATGAAATCGCCTCGCGCCATCCCGATGCGATTTCCTTTGTCTCCGGGCG

7921 CCCGAGCAACGACTTTTTCGATCTGCAGGCATGGATGCAGGCGCTGCCGCGCTTTGCCGA

7981 CCACCTCGCTCGACGGCATCGGCTGACGCCCTCCCAATCGCTGGATACGATCGCACAGTA

8041 CGGCGCGACCCAGGGCATCGTGAACGACCTGATCGTCGCGCAACTGGCCATCGACGAGGG

8101 CGTACCGGCCAGCAGCGAACGTCTGTTGGTCACCGCCGGCTGCCAGGAAGCGCTGCAGTT

8161 GTGCGTCACCGAGCTGTGCCGGGAGCCAGGCGATGTCGTGCTGGTGCGCAACCCGACCTA

8221 TATCGGCATTACCGGCGTCGCCGATAGCCAGCGGATCCCGATCACCGCATTCAACCGTGG

8281 CGACCGCGCCGATGTCGGCGCATTGGCCGAAACGCTCGACGCACTGGAAGCCGCCGGACA

8341 ACGCGCACGCCTGCTCTACCTGGTCCCGGATTTCGACAACCCCACCGGCAGCGTCCTGGC

8401 GCGCACGCAACGCGAAGCGATCATTGCCTTGTGCGCGGCACGCGGCGTCGTGATCCTGGA

8461 GGACAACCCCTACGGACTGTTCGCGTTCGAGGCCGAACGCCTGCCCACCATGCGCGCACT

8521 GGACCGCCACGGCTGTGTGATCTACCTGGGCACCTATTCCAAGACCTTATGTCCCGGACT

8581 GCGCGTGGGCTTCGCGGTCCTGCCACCGACGTTATTCGGCGATGCCTGCGCCGCAGATGC

8641 CTTGCAGCAGGCGCTGACGCAGCGGAAAAGTTTCGGCACACTGAACACCGGTCAAATCGC

8701 CCAAGCCATTGTCGGCGGTGTCTTGCTCGAACACGGCGGCAGCCTGGAGGCGTTGATCCA

8761 GGCCCCACGCGCGCAGTACCGGCGTAATCGCGACCGCATGCTGGAGTGCCTGAGCAAAGA

8821 ACTTGGCACGCTGGCAGCGGATGTGCAGTGGAATGTACCGTCCGGCGGATTCTTCCTGAT

8881 CGTCTCGCTCCCGTTCGTGTTCGCCGAAGAGGAAGCCGCGCGTTGCGCACGCGACTACGG

8941 CGTCCTAGTGATGCCCTTGTGTTTCTTCGCCATCGACGGTGGCTGGCGCCAACACGTGCG

9001 GCTTGCCTACAGTCATGTGGCGACGGAAGCGATCGTCGAGGGTATCGCACGCTTTGCGCG

9061 CTTCGTGCGCGACCGGCTACACGCACTGGCAACGGCGCAAACGCCGCTGCCCCTGCCCGA

9121 CTGCGCTTGAAACGGCCGCAACCGCCTCACGACAACATGCGCGGGCGGTGAGCACTTGGC

9181 ACACCCAATTCGCCCCTGCCCCTCGCGTCGGCCACAAGCATCCACGACACACGCCGCGCC

9241 CGCGCCTGACAGCGACGCCATCGGTGCGATCGACGCGCTCGCAGCGCCCTCCTCACGACA

9301 ACAGGATTCCACGGTCGACCGCCGCGATTTCCGTGCGTCCGCACAATGCCATCGTCAACT

9361 CCAGTTCGCGCTGGATCAACGCCAGCACATGTGCCACCCCCGCCTGCCCCAGGGCGGCAA

9421 GACCATACAACCACGCACGACCGATATAGGTCGCACTGGCGCCGAGCGCCAACGCCTTGA

9481 GCACGTCCTGGCCGCAGCGAATGCCGCTGTCCAGATGCACATCGGCACGGCCAGCGGCCA

9541 ACTCCGCAATGGCGGGCAACGCGCGTACGCTGGAACACGCGCCATCGAGCTGGCGGCCGC

9601 CGTGATTGCTGACGACCAGCGCATCGGCACCAGCGGCAATGGCCTGACGCGCGTCCTCCG

9661 CATCCAGCACGCCCTTGATTACCAACGTCCCACGCCAACGCGAACGGACCCATGCCACAT

9721 CCTCCCACCCGATCCCCGCATCGAATTGCTGCGCGGTCCAACCGGATAGCGTCCGCAAGT

9781 CGTTGCCGGCAGGCGCATAGTCCACGAGATTGCCGAAGCAGCGACGACGCGTGCGCAACA

9841 TCCCCAAGGCCCAGCGCGGATGCGCGAGAAAATCCAGCAATGCCGCAGGCTTGAGCCGCG

9901 GCGGCACACTCAGGCCATTGCGCAGATCGGCATGACGCTGGCCCATGAACGGCACATCCA

9961 GGGTGAGGACCAGCGCCGCGCAGCCTGCTCGTGTCGCACGCTCGATCAGCGCCGCCACGA

10021 TGCCGCGATCGCGCATCGGATAAAGCTGGAACCAGCATGGCGCCCGCAGCACCGCACAGA

10081 CTTCTTCCAGCGCACAGATGCTCATCATCGACAGTACAAACGGCACACCGAACGCTTCGG

10141 CTGCACGCGCGCCCAGGATCTCGCCATTGGCCCAGATCAGCCCCGCCAATCCGGTGGGTG

10201 CCAGCGCAACCGGCATGCTGACCTGGCGCCCCAGCATGCACGCGGCGGTGCTGCGCCGCG

10261 CGACATCGCAGCCGATGCGTTGACGCAGCGCCAGCGCATCGAAGTCACGACGGTTGGCAT

10321 GCAAGGTCGACTGCGACCAACTACCGGCATCCACGTAGTCATAGAACATGCGCGGCACGC

10381 GGCGCCGCGCCAAGGCTTTCACATCGTCCAAGCACGTCAGCGTCTGCATCCGCATAGGCT

10441 AGATCCTGTAAATGACCAGAATACGTTCATCCGGCGCAACACACGATTATTCCTGACGAC

10501 CACAGAAAGAACATCCGCCTACAAAAAAAACATCTTTCCGTCACATAGCGGTCAATACAT

10561 GGACCGATACTTGGCCTAGCAAGTAAAAAGTGAACACATCAGGCGCGATACCACGAGCAT

10621 AGCGACGACTGCGGCCAGATACCCGGGCCGAGCAGAACCTACGACATCATATTCACAACA

10681 CTCGGCGTACTTAAGCACACGCCGACTTCGCTACACGATTAGAGAGCCACATGAGCGATC

10741 CTCGCTTGATATTCCAACGCCACGAATCCAACGTCCGCAGCTACTGCCGCTCCTTTGATG

10801 CCATGTTCGTCCGCGCATCGGGAAGCCTGATGTTCGATGCACAGGGTCGTCGCTACATCG

10861 ACTTCCTAGCCGGCTGCGGCTCGCTCAATTACGGACACAACGATCCAGACATGGCCGAGG

10921 CACTGATCGGGCACCTACGCAACGGCGGCTTGGCGCTCTCGCTGGATATGTACTCGCAGG

10981 CAAAGCACGACTTCATCGATGCTTTCGTGGAAAGAATCCTCAAGCCGCGCGGACTTACCC

11041 ACCGCCTGCAGTTCACCGGCCCGACCGGTACCAATGCAGTCGAAGCCGCGCTCAAGCTGG

11101 CACGCAAGATTACTGGGCGTCACAACGTCATTGCCTTCAGTAATGCCTACCATGGCTTAT

11161 CGATGGGAGCACTGGCGACGACCGGCAATCGCCACCATCGCATGGAACTAGCGCACACCG

11221 GCGTCACCCGACTGCCCTACGACGGCTATCTCGGCGAGAGCGTGGATAGCGCCGCTTTGC

11281 TGGAAGGCATGCTCGACGACCCCTCCGGCGGCATCGATCCACCCGCCGCGATCATCCTGG

11341 AACTGGTGCAAGGCGAAGGCGGGCTCAATGTGGCGTCCACGCCCTGGTTGCGCCGCATAT

11401 TCGCCGCTGCCCGCCGCCACGGCGCGCTGGTCATCGTCGACGATGTCCAGGCAGGTTGCG

11461 GACGCAGCGGAGGCTTCTTCAGTTTCGATGGGCTGGACCTGGTCCCAGACCTGATCGTCC

11521 TGTCCAAATCCCTATCCGGTTTCGGCCTGCCATTCTCGCTACTGCTGGTCGCACCCGAGC

11581 ATGACCAATGGCGTCCTGGCCAACACAACGGCACCTTCCGCGGCAACAACCATGCGATGC

11641 TCACCGCCAGCGTCGCGCTGCATAAATTCTGGGCCGACGACACATTCGCCACGCAAGCGG

11701 CAGCGCGTGGTCAGCGCGTGACCGAAGCACTGACGCGGATCGCCGCGCAGGTGCCGGGGG

11761 CACGCGTCAAGGGGCGCGGCATGTTCATGGGGATAGATGTCGGGACCACTCAACTCGCCG

11821 CCTCGGCACGCGCGCATGCGTTCAAACACGGCTTGGTGATCGAAAGCGCTGGCCCGAACG

11881 ACGAAGTACTGAAAGTAATGGCTCCGTTGACCACACCCGATGCCCTACTGGATGAGGGCC

11941 TGGACATTCTTGCGGATGCGGTACTGCATGCCTGCGAGCAAACGCAACAGGTCAAACTGA

12001 GCCGGGTGGCATAAGACCGAGGTACTCCAAGCAGCTCGATGGCCGCGCTGCTGCGGCCGA

12061 CCCGGCTGTCTTGAGCGTTCCGTGCGCAGCGAGTGCTCACATTGCCCCGGCACTGCCACC

12121 ACAGGTTCGTTGTTCGTCCACGCCTGCCTGTCATCGCACACAGCGTAGGAAGCCTTTAAC

12181 GCCGCCCGGTCAACGCACGTCCCGGCCTTTCCTTCGTTGCGTTTGACGCTCGAATCCAGA

12241 CAAGGCAGGCATCGCACCCCAGGCACGCTGGCCCAAAATGAAAAGCCCCGCAACGCGGGG

12301 CTTTTGGGATCTGCGCGACACGGACGAAACCGTGTAACTCAGAACGGAATATCGTCGTCG

12361 GCGAAATCATCCATCGGTGCCGATTGCGACGGTGCCGGCTGTTGGCGTCGTGGAGCATAG

12421 TCCTGCCCGCCACCGCCCATGTCCGAACGCTCCTGACGCGGTGGTGCCGAACGCGCCGGG

12481 CGGTCGCCGCCGCCCATACCGCCGCCGCCTTCGCCACGGCCGCCAAGCATTTGCATTTCA

12541 TCGGCGACGATGTCCGTGGAATACTTCTCCACGCCGTCCTGGCCGGTGTACTTGTCGTAG

12601 CGCAGCGAACCTTCGACGTAGACCGAACTCCCTTTACGCAGGTACTCGCCAGCGATTTCG

12661 CCCAGCTTTCCGAAGAACACCACGCGATGCCACTCGGTGCGCTCCTGCTGGTTGCCGTCG

12721 CGGTCCTTGCGCACGCTGGTTGTCGCCAGACTGACGCGGGTGATGGCCATGCCGGACTGG

12781 GTGTACTTGGTGTCGGGATCGTTGCCGAGATTGCCGACGAGGATGACTTTATTGATGCCG

12841 CGGGCCATGAGCTGCTTCCGTCTGGGGTCCCCACGGCCAGAGGGCACGCGGAGCGGTAAA

12901 GAACACGATTAGGAACTCCTAATCCGCCAATCCTAACATTGCCGGGCTGGCCCCACCCGC

12961 GCCGAACGCCTGCAAAGGCCTAGGGAAACCCCGCGCCACACGACCGCAATGCCGGCAACG

13021 CAGCACCGGCAAGGCTCTCCAGCCCCCGCCCCACACCGCCCAGACCAGCCCGATCACGCC

13081 CGGCGCAGGACGGATGTCGCCGCGCAGCCTATACTCCGCGCACGCATCCAGCCTTGTCCG

13141 CATGAGCATCGCCGAAGACCTCCCCACCCTGGGCCTGCCCCAGATCCAGATGCTCGCCGC

13201 AGCCGACATGGCGGCAGTCGATACCTTGATCCGCCACCGTCTGGCGTCGGACGTGGTGCT

13261 GATCAACCAGATCGCCGACCACATCATCTCCGCCGGCGGCAAACGCCTGCGGCCGATGCT

13321 GGTCATGCTGGCCGGCCGCGCCTGCGATGGACATGGACCAAAGCACCACCAGTTGGCGGC

13381 GATCATCGAATTCATCCACA

LOCUS Contig G106 24000 bp DNA linear BCT 30-JAN-2012

DEFINITION Xanthomonas spp. strain XaS3 genomic region encoding NRPS.

SOURCE Xanthomonas spp. strain XaS3

ORGANISM Xanthomonas spp. strain XaS3

Bacteria; Proteobacteria; Gammaproteobacteria; Xanthomonadales;

Xanthomonadaceae; Xanthomonas.

REFERENCE Contig G106 (bases 1 to 24000)

AUTHORS Royer et al. Submitted to BMC Genomics.

TITLE Genome mining indicates that the genus Xanthomonas is a promising

reservoir for new bioactive non-ribosomally synthesized peptides

JOURNAL Submitted to BMC Genomics.

REFERENCE 2 (bases 1 to 24000)

FEATURES Location/Qualifiers

source 1..24000

/organism='Xanthomonas spp.'

/mol_type='other DNA'

/strain='XaS3'

/isolation_source='sugarcane leaf water droplets'

/host='Sugarcane'

/db_xref='taxon: GPE 39'

/country='Guadeloupe'

/collection_date='2003'

/collected_by='Rosiane BOISNE-NOC'

/note='[cultured bacterial source]'

gene <2..18763

/gene ='NRPS'

CDS <2..18763

/gene ='NRPS'

/note='incomplete NRPS, no start codon'

/codon_start=1

/translation='LDEAERHQVLTQWNATKADYPRDACVHALFEAQVARDPSAIAIVQNNVTLTYGELNARSNQLAHYLRELGVRPDDRVAVCVQRSVEMVVALLAVLKAGGAYVPLDPAYPSERLAYMQADCGALAMLTDTASRQLIEDITTSVVIVDLQTDSEHWAHLPDSNPDRYANGLTASHLAYVIYTSGSTGVPKGVMTEHRNVVGLFGATEERFEFSSDDVWTLFHSYAFDFSVWEIFGALLYGGRLVVVPWETARSPDAFHELVCRESVTVLNQTPSAFKSFIAAQGSSGSEHCLRMVIFGGEALDVAALKPWFERERNLATHLVNMYGITETTVHVTWRSLTVTDVDWRGGSPIGRPIANTRTYILDGHGAPVPIGVVGELYIGGEGVARGYLNREDLTAARFIADPFSDTADARMYRSGDLGRWRTDGTIEYVGRNDHQVKIRGFRIELGEIEARLSAHADVRECVVVALEGAAAGTEKRLVAYWVAAGHVTSEPLGAESLRSWLSDTLPDYMVPAAYVQLDRLPLTPNGKLDHKALPAPDGSAYAAPAYEAPQGAIEQTIAAIWCDLLGLESIGRHDNFFALGGHSLLVVTLTERMRQQGFHADLRTLFANPTLAALAAASGGLSIIVPPNRIEPDSVEITPEMLPLVELTQQQIDSIVAMTPGGSANIQDIYPLAPLQEGIFFHHLLQREGDAYLLPNLIAFDSRSRLDVFVDALQCVIDRHDILRTAVLCEGLAAPVQVVWRHAPLLIEEVCLDGACDNVAELLQSRFDPSCWRMDVRQAPLMRGFAAYDPASSRWLLQLLSHHLALDHTTLEIVLDEIRCHLCAETASLPAPLPFRNFVAQAFLGVSVEEHEAYFRTMLGDVDEPCAPFGLLDVQGDGSDVEEVRIDLSDRLSALLRSQARALGVSVASLFHLAWAQVVARTTGHERVVFGTVLFGRMQGGAGADRTLGIFINTLPLRIEIDDVSVVESVRIVQQRLAGLLRHEHAPLSLVQRCSGVPAPAPLFTSLLNYRYSVQTEGSAETMAWDGIERISGHESTNYPLGVSVDDVGFGFSIKVQTQRPLVPARICTFLVNALEGLADALAHAPETAVRDLDVLPEAERHQVLMQWNATTNDYPREACVHELFEAQVARTPSAIAVVQGEVSLTYRELNARANGLAHSLRELGVCPDDRVAICMQRSVEMVVAVLAVLKAGGAYVPLDPAYPVERLAYMLEDSAPVAVLAQTSTLNLLLTASAPIINLDESHWQDRSVSNLSMDGLTSTHLAYVIYTSGSTGRPKGVMIEHRNTVNLLAWAQRSFAASVLEKVLFSTSLNFDLSVYECFVPLVCGGAIEVVDNLLAMQADGQGVTLINTTPSALKGWLESGGRGEGVHTVNVCGEVLKRQVVEDLFAKTQIERLCNLYGPSETTTYSSWVSMERSDGFVSHIGTPLDNTQFYVLDTHRQPVPVGVTGELYIGGAGVARGYLNRGDLTAARFLMDPFSADPTARMYRTGDLGRWRADGTLEFVGRNDHQVKIRGFRIELGEIEARLSAHVDVRECVVVALEDATGSDKRLVAYWVGAQDATHESLGVESLRSWLSDTLPDYMVPAAYVQLDRLPLTPNGKLDRKSLPAPDATAYAAPAYEAPQGEVEHTIAAIWRELLGLESIGRHDNFFALGGHSLLAVRVASRLRQELGVEIGVAELFANATLKDLAACVASSSAAILPAILPLQPDAPRVLSFAQQRLWFLSQFEGVSEAYHISGGLRLRGVLDVQALQRALDRIVARHASLRTSFALVDGQALQHVADEDSGFHLIDHDLREVPDREAVLEQLLAEEVQTPFALEQGPLIRGRLVRLADDESALFVTMHHIVSDGWSMGILINELSVLYRAFAHGEADPLAPLPIQYADYASWQRQWLTGDVLEQQATYWRKTLSDAPVLLELPTDRPRPAQQDHAGAMLEVIVDPQQAQALKALSQRHGLTLYMTLLASWALLLSRLSGQDDVVIGSPVANRGRSETEGLIGFFVNTLALRVELSGSPTLAQLLASVKNRALQAQAHQDIPFEQVVELVQPPRSLAHTPLFQVMFSWQNAPQGELDLGAIEASGLSAIQTSAQFDLSLSLVESQEGIIGSLAYATALFERSTLERWMGHWQHLLDAMAAEGAEHQALDRLPLLDDAERYQVLTQWNATAADYPRDACVHELFEAQVARTPSAIAVVQGEVSLTYGELNARANRLAHCLRELGVRPDDRVAICVQRSVEMVVALLAVLKAGGAYVPLDPAYPPERLAYMQSDCGAVVVLTDTASRHLVEHPTASTVVVDLQADGQRWAHLPDSNPDRNAVGLTSRHLAYVIYTSGSTGMPKGVMIEHRGCVNLYHHYAICYLRSGDKVLVLSSFSFDLTLKNIISPLFLGCAVELAPAGVVVGSRILNLLESSGAALINCAPSQLHGVLDDFESKLRVASLAKLRYIILGGEKIKIDLISEWISGSEKYVFINSYGPTEITDVAVDGVIKGGDVSESMPIGRPIANTRIYILDMHGAPVPIGAVGELYIGGDGVGRGYLNRDDLTAARFLADPFSADPTARMYRSGDLGRWRTDGTIEFVGRNDHQVKIRGFRIELGEIEARLSAHADVRECVVVPMEDATGNDKRLVAYLVAAEGVMSEHLGAESLRSWLSDTLPDYMVPAAYVQLDRLPLTPNGKLDRNALPAPDEAAYAVRAYEAPQGAIEQAIAAIWRDLLGLEAIGRHDNFFALGGHSLLVVTLTERMRQQGLQADLRTLFATPTLVALAAASGGVSVSVPPNGIMRDTTLITPEMLPLAVLTQQQIDSIVAMTPGGSANIQDIYPLAPLQEGIFFHHLLQREGDAYLQPNLIAFDNRSRLDVFVDALQCVIDRHDILRTAVAWEGLAAPMQVVWRHAPLLIEEVCLDEADGDVAMQLQSRFDPRHWRLDVRHAPLMRGFTAYDSVNGRWLLQLLSHHMALDHTTLEILLEEVRCHLCGDADSLLPPLPFRNFVAQALLGVSHEEHEAYFSTMLGDVDEPCAPFGLIDTQGDGSDLEEVRLNLPDRLSLSLRNHARTLGVSTASLFHLAWAQVVARATGHERVVFGTVLFGRMQGGAGADRALGMFINTLPLRIEIDGSSVVESVRLMQQRLAGLLRHEHAPLSLVQRCSGVPAPAPLFTSLFNYRYSGQAEGVAETMAWEGIETLSSHERTNYPLGVSVDDVGSAFVLTVQAQRPLVPERICAFLLKALESLANALADAPKTPVCDLDVLPEAERHQLLTQWNATATDYPRDKCVHELFEEQVARAPLAIAVVQGEVSLTYAELNARANRLAHYLRELGVRPDDRVAICVQRSVEMVVAVLAVLKAGGAYVPLDPAYPPDRVAYMQVDCDAVAVLTDSASRHLIENNATSVVIVDLQADAAQWQHLPDSNPDHHAIGLTARHLAYVIYTSGSTGKPKGSMNEHRSVVNLALAQIGAFHVEEDSRILQFSSLSFDAFASELLVTLFCGASLYIADQGDVLAGETLTKLLAENEISHVTLPPSVLHQMPEEPRLSSLKTLVVAGEALSVAIVERWAEGRRLINAYGPTETTVCACVHECDANVAGAPPIGRPIANVRIYILDMHGAPVPVGVVGELYIGGDGVGRGYLNRDDLTAARFLMDPFSADPTARMYRTGDLGRWHTDGTIEFVGRNDQQVKIRGFRIELGEIEARLGTHADVRECVVVALEDAAGSDKRLVAYWVATQDATHAPLGVESLRSWLSDTLPDYMVPAAYVQLDRLPLTPNGKLDHKALPAPDATAYAAPAYEAPQGEVEHTIAAIWCDLLGLESIGRHDNFFALGGHSLLAVRVASRLRQELGVEIGVAELFANATLKDLAACVASSSAAILPPILPLQPDAPHVLSFAQQRLWFLSQFEGISQAYHISGGLRLRGVLDAQALQRALDRIVARHASLRTTFALVDGQALQQIADEESGFHLLTHDLCGVPDREEALEKLLTEEAHAPFALEQGPLIRGRLIRLADDESVLFVTMHHIVSDGWSMGVLINELSVLYRAFAHGEADPLAPLPIQYTDYASWQRQWLAGEVLEQQATYWREALSGAPVLLELPTDRPRPGRPARQDHAGAMLEVVVEPHQAQALKALSQRHGLTLYMTLLASWALLLSRLSGQDDVVIGSPVANRGRSETEGLIGFFVNTLALRVELSGSPTLEQLLASVKNRTLQAQAHQDIPFEQVVELLQPPRSLAHAPLFQVMFAWQNTPQGELDLGELDASGLGVAQTSAQFDLSLSLVESEEGIVGSLAYATALFERSTLERWMGHWRHLLAAMVAEGAEDQAVDRLPLLDDAERYQVLTQWNATAADYPRDACVHELFEAQVALDPSAIAVVQGEVSLTYGELNARANRLAHYLRELGVRPDDRVAICVQRSVEMVVAVLAVLKAGGAYVPLDTAYPPERLSYMLTDCGAVAVLTDDASRPLVEDNAKSGVIVDLQTEDERWQHLPDRNPDRDANGLTANHLAYVIYTSGSTGMPKGAMNAHRGVVNRLVWMQEAYTLDRSEVVLQKTPISFDVSVWELFWPLLSGARVQLAQPEGHKDPVYLKALIRDTQITTLHFVPSMLRALIEHGDDDPCTGVKRVICSGEALPAVLAERAQAVFPSSEIFNLYGPTEAAVDVTAWRYCAGWENAAILPIGRPIANTQIYILDVQGAPVPIGVVGELYIGGDGVGRGYLNREDLSAERFLTDPFSTDPTARMYRTGDLGRWRADGTIEFVGRNDHQVKIRGFRIELGEIEARLSAHVDVRECVVVALEDATGSDKRLVAYWVGRQDATHTPLGAESLRSWLSDTLPDYMIPAAYVQLDRLPLTPNGKLDRKALPAPDGSAYAAPAYEAPQGAIEQTIAAIWCDLLGLESIGRHDNFFALGGHSLLAVRVASRLRQELGVEIGLAELFAHATLKDLAACVASSSAAILPPILPLQPDAPRVLSFAQQRLWFLSQFEGVSQAYHISGGLRLRGALDTQALQRALDRIVARHASLRTTFALVDGQALQHIAAEESGFHLIDHDLRGVPDREKALEKLLTEEAHAPFVLEQGPLIRGRLIQLADDESVLFVTMHHIVSDGWSMGVLINELSVLYRAFAHGEADPLAPLPIQYADYASWQRQWLAGEILEQQATYWRETLSGAPVLLELPTDRPRPARQDHAGAMLKVVVDPQRTHALKALSQRHGLTLYMTLLASWALLLSRLSGQDDVVIGSPVANRGRSETEGLIGFFMNMLALRVEFSNSPTLAQLLALVRERALQAQAHQDIPFEQVVELIQPPRSLAHTPLFQVMFAWQNTPQGALDLGAIEVSGLGVAQTSAQFDLSLSLVESEEGIVGRLTYATALFEHATVQRWMGHWQHLLDAMVADGAEDRAVDRLPLLDDAERHQVLMQWNATTKDYPSDACVHELFEAQVARTPSAIAVVQGEVSLTYDELNARANCLAHSLRELGVCPDDRVAICMQRSVEMVVAVLAVLKAGGAYVPLDPAYPVERLAYMLEDSAPVAVLAQTSTLTLLLTASVPIINLDESHWQDRSVSNLSMDGLTSAHLAYVIYTSGSTGRPKGVMIEHRNTVNLLAWAQRSFAASVLEKVLFSTSLNFDLSVYECFVPLVCGGAIEVVDNLLAMQADGQGVTLINTTPSALKGWLESGGRGEGVHTVNVCGEVLKRQVVEDLFAKTQVERLCNLYGPSETTTYSSWVSMERSDGFVSHIGTPLDNTQFYVLDTHRQPVPVGVTGELYIGGAGVARGYLNHGDLTAERFLMDPFSADPTARMYRTGDLGRWRADGALEFVGRNDHQVKIRGFRIELGEIEARLSAHVDVRECVVVALEDATGSDKRLVAYWVATQDATHESLGVENLRSWLSDTLPDYMVPAAYVQLDRLPLTPNGKLDRKALPAPDATAYAAPAYEAPQGEVEHTIAAIWCDLLGLESIGRHDNFFALGGHSLLAVQMVSRLNHAGIENKISDLFSNPILMSLAAHLQNEVYRGKFNGLIPFREEGDENPLFMLYEASGSVLYAKNIEKFLRAGMPIYGIECPVDFSIDTIQGMAADAIKKMRNLQSSGPYRLAGWSFGGLVAYEMASQLVDANLQVEFLGLLDTSHLSTDAKIKNSMDSARKIDCDYVIRKLFETGFGHKVDSSEKINELSDLVKLYQEMGFSSDFIREGELMDYAMRVSHHINASIAYYAKSIPMRVHLFEASQFSEDNPFLGSPAQSWQGVLPENLIRVIEVPGTHQTMMESPNVEILSRVLSEAIKNGLTDPQS'

gene complement (20602..23358)

/gene ='acnA'

CDS complement (20602..23358)

/gene ='acnA'

/note='Aconitate hydratase 1, ortholog of XALc_1349'

/codon_start=1

/translation='MHDTFSSRASLEVNGKRYTYFSLSRLGQRFDIARLPYSLKILLENLLRHEDGGATVSKEHIEAVARWNPTAEPDTEIAFMPARVVLQDFTGVPCVVDLAAMRDAVVKLGGRPEQINPLIPSELVIDHSVQVDVFGKPDALDLNGKIEFQRNKERYGFLRWGQKAFDNFKVVPPNTGIVHQVNLEHLARVVMTGERDGEAIAYPDTVFGTDSHTTMINGIGVLGWGVGGIEAEAAMLGQPSSMLIPQVVGFKLSGKLPEGVTATDLVLTVTQMLRAHGVVGKFVEFFGDGLQHLPLADRATIGNMAPEYGATCGIFPIDAESLTYLRLSGRNAEQIALVEAYAKAQGLWHDAESPHAHYSATLELDMSQVKPSLAGPKRPQDRVLLEDMQRNFRDNLVPFAQTRAKRRGDTMQEDRLKNEGGGGTAVGTQAAQAQHADTSGAGWQLRDGAVVIAAITSCTNTSNPAVMLGAGLLARNAVAKGLKAQPWVKTSLGPGSLVVTDYLKKAGVMHDLETLGFYVVGYGCTTCIGNSGPLPEDVSAAIAKDDLVVTSVLSGNRNFEGRVHPEVKMNYLASPPLVVAYAIAGTTDIDLTREPLGTGRDGQPVYLRDIWPSNKAIGDTIAATVGPEMFEQNYADVFKGDSRWNTIASPDGELYAWDQASTYIKNPPYFDGMTMQVGHIEDVHGARILALFGDSITTDHISPAGNIKKDSPAGRYLQERGVQPADFNSYGSRRGNDEVMVRGTFANIRIKNLMFGGEEGGNTLYHPAGGGQPQKQSIYDAAMQYKADGVPLVVIGGKEYGTGSSRDWAAKGTHLLGVKAVIAESFERIHRSNLVGMGVLPLQFLDGQNAQTLGLDGSEILHIAGLQDGSSKRATVTATASNGSTQTFTVAVMLLTPKEVEYFRHGGLLQYVLRQLTRR'

ORIGIN

1 GCTGGACGAGGCCGAACGCCATCAGGTGCTGACGCAGTGGAATGCGACCAAGGCGGATTA

61 TCCTCGCGATGCCTGTGTGCACGCATTGTTCGAGGCGCAGGTGGCGCGTGATCCATCGGC

121 AATTGCGATTGTTCAAAACAATGTCACGCTGACGTATGGCGAGTTGAATGCGCGTTCCAA

181 CCAGTTGGCGCATTACCTGCGCGAGTTGGGTGTGCGCCCAGATGACCGCGTGGCAGTGTG

241 CGTGCAGCGCAGTGTCGAGATGGTGGTGGCGTTGCTGGCGGTGTTGAAGGCGGGCGGTGC

301 GTATGTGCCGCTGGATCCAGCGTATCCATCCGAGCGGCTGGCTTATATGCAAGCCGATTG

361 CGGTGCGCTGGCGATGTTGACGGACACCGCCAGCCGCCAGCTGATCGAGGACATTACAAC

421 TTCGGTGGTGATCGTCGATCTGCAAACTGACAGTGAGCATTGGGCGCACCTGCCAGACAG

481 CAATCCCGACCGCTACGCCAATGGCCTGACTGCAAGCCATCTGGCGTATGTCATCTACAC

541 CTCTGGATCGACCGGTGTGCCGAAGGGTGTGATGACCGAACATCGGAATGTCGTGGGTCT

601 CTTTGGGGCAACAGAAGAGCGCTTCGAATTTTCCTCGGATGACGTGTGGACACTCTTTCA

661 TTCATACGCCTTCGACTTTTCGGTTTGGGAAATATTCGGCGCATTGCTTTATGGAGGACG

721 TCTTGTCGTCGTGCCATGGGAGACTGCGCGCTCGCCAGATGCGTTCCATGAACTTGTCTG

781 TCGCGAATCCGTGACGGTTTTAAACCAGACGCCGAGTGCTTTTAAGTCATTCATTGCCGC

841 GCAGGGCTCCAGCGGTTCGGAGCATTGTCTGCGGATGGTCATCTTTGGTGGCGAAGCATT

901 GGACGTCGCGGCCTTGAAGCCTTGGTTCGAGCGTGAGCGCAACCTGGCGACGCATCTGGT

961 CAATATGTACGGGATTACGGAAACCACGGTGCATGTGACGTGGCGGTCTCTCACCGTGAC

1021 CGATGTGGACTGGCGTGGTGGTAGTCCGATTGGTCGTCCGATCGCCAATACGCGTACCTA

1081 CATCCTCGATGGACATGGTGCGCCGGTGCCGATCGGGGTGGTGGGCGAGTTGTATATCGG

1141 TGGCGAGGGTGTGGCACGTGGATATCTGAATCGCGAGGACTTGACCGCCGCGCGCTTTAT

1201 TGCCGATCCGTTCTCCGATACAGCCGATGCACGGATGTACCGCAGCGGCGATCTTGGGCG

1261 TTGGCGTACCGACGGCACAATCGAGTATGTGGGACGTAACGATCATCAAGTCAAGATCCG

1321 TGGTTTCCGCATTGAATTGGGTGAAATCGAGGCGCGACTGAGTGCGCATGCGGATGTGCG

1381 CGAATGCGTGGTGGTGGCGCTGGAAGGTGCGGCTGCTGGCACCGAGAAGCGTCTGGTGGC

1441 GTATTGGGTCGCTGCCGGGCATGTAACGTCCGAGCCTCTTGGCGCGGAAAGCTTGCGCAG

1501 TTGGTTGTCGGATACCTTGCCGGATTACATGGTGCCTGCCGCCTATGTGCAGTTGGATCG

1561 CTTGCCGTTGACTCCGAACGGCAAGTTGGATCACAAGGCATTACCCGCACCGGATGGCTC

1621 GGCCTATGCAGCGCCTGCGTATGAAGCACCGCAGGGCGCGATTGAACAAACCATTGCCGC

1681 GATTTGGTGTGATCTGCTGGGTCTGGAAAGCATCGGGCGGCACGATAACTTCTTCGCGCT

1741 CGGTGGACATTCGTTGTTGGTAGTGACGCTGACCGAGCGGATGCGTCAGCAAGGGTTTCA

1801 CGCCGATTTGCGCACACTGTTCGCCAACCCGACGCTTGCGGCACTGGCCGCGGCGAGCGG

1861 TGGCTTATCGATCATTGTGCCGCCTAACCGCATTGAGCCTGATAGCGTAGAGATCACGCC

1921 TGAAATGCTGCCGCTGGTGGAGCTCACTCAGCAACAGATCGATAGCATCGTCGCAATGAC

1981 CCCTGGCGGCTCGGCCAATATCCAGGATATCTATCCGCTTGCTCCATTGCAGGAAGGTAT

2041 CTTTTTCCATCACCTGCTGCAGCGCGAAGGCGATGCCTATCTGCTGCCGAATCTGATCGC

2101 ATTCGACAGTCGTTCGCGTCTGGACGTATTTGTCGATGCACTGCAATGTGTGATCGATCG

2161 TCACGACATCCTGCGCACGGCGGTGCTCTGTGAAGGACTCGCCGCACCTGTGCAGGTGGT

2221 GTGGCGGCATGCGCCATTACTAATCGAGGAAGTTTGCCTAGATGGCGCCTGCGATAATGT

2281 GGCGGAACTGCTGCAATCGCGCTTCGATCCAAGCTGCTGGCGCATGGATGTGCGTCAAGC

2341 GCCGCTGATGAGGGGTTTTGCCGCATACGATCCGGCCAGTAGTCGCTGGTTGCTGCAGTT

2401 GCTCAGTCACCATCTTGCCCTGGATCACACCACGTTGGAGATCGTGCTGGATGAAATCAG

2461 ATGCCATTTGTGCGCTGAAACAGCGAGCTTGCCGGCTCCGCTGCCGTTCCGCAATTTTGT

2521 GGCGCAGGCATTTCTGGGTGTCAGTGTCGAAGAGCACGAGGCATATTTCCGCACAATGCT

2581 CGGCGATGTGGATGAGCCATGTGCACCATTTGGCCTGCTCGATGTACAGGGTGATGGATC

2641 GGATGTGGAGGAAGTACGGATCGATCTATCGGATCGGCTGTCGGCATTGCTACGCAGCCA

2701 GGCGCGTGCGCTTGGCGTGAGTGTGGCAAGCCTGTTCCATCTGGCCTGGGCACAAGTGGT

2761 AGCGCGTACCACAGGTCATGAGCGTGTTGTATTCGGAACCGTGCTGTTTGGTCGGATGCA

2821 AGGCGGAGCGGGAGCCGACAGAACGCTTGGCATTTTCATCAATACTTTGCCCCTGCGCAT

2881 TGAGATTGATGATGTCAGCGTGGTGGAAAGTGTGCGAATAGTGCAGCAGCGCCTTGCCGG

2941 GCTGTTGCGACACGAGCATGCGCCGTTGTCGTTGGTGCAGCGTTGCAGTGGTGTTCCCGC

3001 ACCGGCACCCTTGTTTACCTCATTGCTCAATTACCGGTATAGCGTCCAGACAGAAGGATC

3061 TGCCGAAACGATGGCGTGGGATGGGATAGAGAGGATTTCAGGACATGAGAGTACGAATTA

3121 TCCGCTTGGTGTTTCTGTAGACGATGTTGGGTTTGGTTTCTCGATAAAAGTACAGACACA

3181 GCGGCCATTGGTTCCGGCACGGATATGCACTTTCCTGGTGAATGCGCTTGAGGGTCTTGC

3241 CGATGCGCTTGCCCATGCACCGGAGACAGCGGTGCGAGATCTCGACGTCTTGCCTGAGGC

3301 CGAGCGTCATCAGGTGTTGATGCAGTGGAATGCAACGACGAATGATTATCCGCGCGAGGC

3361 GTGTGTACATGAACTGTTCGAGGCACAGGTGGCACGAACACCATCCGCCATCGCGGTGGT

3421 GCAGGGTGAAGTGTCGCTGACGTATCGCGAATTGAACGCACGTGCCAACGGTCTGGCGCA

3481 TTCTCTGCGCGAGTTGGGTGTATGCCCGGATGATCGCGTGGCGATCTGCATGCAGCGCAG

3541 TGTGGAGATGGTGGTCGCGGTGCTTGCGGTGCTGAAGGCCGGTGGTGCCTATGTACCACT

3601 GGACCCGGCTTATCCTGTCGAACGTCTTGCCTATATGTTGGAAGACAGCGCTCCGGTTGC

3661 GGTTCTGGCGCAGACATCGACCTTGAACCTGCTGCTGACCGCATCCGCGCCGATCATCAA

3721 TCTGGACGAGTCGCACTGGCAAGATCGGTCCGTGTCGAATCTGTCGATGGATGGTCTGAC

3781 CTCGACGCATCTGGCTTACGTGATCTACACCTCCGGCTCCACGGGTCGCCCCAAGGGCGT

3841 GATGATCGAACACCGCAATACGGTGAATCTGCTGGCTTGGGCACAGCGTTCTTTCGCAGC

3901 ATCGGTCCTGGAAAAAGTCCTGTTCTCCACGTCGTTGAATTTCGACCTATCGGTCTATGA

3961 ATGCTTTGTGCCTCTGGTGTGCGGTGGTGCAATTGAGGTGGTCGACAATCTGCTTGCCAT

4021 GCAGGCAGATGGGCAGGGTGTCACGTTGATTAACACCACGCCTTCCGCATTGAAGGGGTG

4081 GTTGGAATCAGGCGGACGAGGCGAGGGCGTGCATACGGTCAACGTGTGTGGTGAAGTGTT

4141 GAAGCGTCAGGTGGTGGAAGATCTTTTTGCCAAGACGCAAATCGAACGGCTGTGCAACCT

4201 ATATGGCCCGTCGGAAACCACGACGTATTCGAGTTGGGTGTCGATGGAGCGCAGCGACGG

4261 TTTTGTGTCTCATATCGGCACGCCGTTGGACAATACACAGTTCTATGTGCTGGATACGCA

4321 TCGTCAGCCTGTGCCGGTGGGTGTGACGGGTGAACTGTATATCGGTGGTGCCGGTGTAGC

4381 GCGTGGTTATCTGAATCGCGGTGATTTGACCGCCGCGCGCTTCCTCATGGATCCATTCAG

4441 TGCCGATCCGACGGCACGGATGTATCGCACTGGCGATCTGGGTCGCTGGCGTGCCGATGG

4501 CACGCTTGAGTTTGTGGGACGCAACGACCATCAGGTCAAGATTCGTGGTTTCCGCATCGA

4561 GTTGGGAGAGATCGAGGCGCGGCTGAGCGCGCATGTGGATGTGCGCGAGTGTGTGGTGGT

4621 GGCGCTGGAAGATGCCACGGGCAGCGACAAGCGATTGGTGGCGTATTGGGTTGGTGCGCA

4681 AGATGCGACGCACGAATCCCTTGGCGTGGAAAGCTTGCGCAGTTGGCTGTCGGACACGTT

4741 GCCGGATTACATGGTGCCGGCGGCTTATGTGCAGTTGGATCGCCTACCGCTGACCCCGAA

4801 TGGCAAGCTGGATCGCAAGTCATTACCCGCGCCGGATGCGACAGCCTATGCCGCACCTGC

4861 GTATGAAGCACCGCAGGGTGAGGTTGAACACACCATTGCCGCCATCTGGCGTGAGCTGCT

4921 GGGTCTGGAGAGCATCGGGCGGCACGACAACTTCTTTGCGCTTGGTGGACATTCGTTGTT

4981 GGCGGTGCGGGTTGCCTCTCGCTTGCGTCAGGAATTGGGTGTCGAGATCGGCGTAGCGGA

5041 GTTGTTTGCCAATGCAACGCTGAAGGACCTTGCCGCGTGCGTGGCCTCTTCGTCCGCTGC

5101 GATCTTGCCGGCGATCCTGCCATTGCAGCCGGATGCGCCGCGTGTGCTGTCGTTTGCACA

5161 GCAACGGCTCTGGTTCCTGTCGCAGTTCGAAGGTGTCAGTGAGGCGTATCACATCAGCGG

5221 CGGCTTGCGTTTGCGTGGTGTGTTGGATGTGCAGGCGTTGCAACGTGCGTTGGATCGCAT

5281 CGTGGCTCGACATGCGTCGCTGCGCACGAGCTTCGCGCTGGTCGATGGGCAGGCGTTGCA

5341 GCATGTCGCTGACGAGGACAGCGGTTTCCATCTGATCGATCACGATCTGCGTGAGGTGCC

5401 TGATCGCGAGGCTGTGCTGGAGCAGCTATTGGCGGAGGAGGTGCAGACACCGTTCGCGCT

5461 GGAACAAGGCCCGTTGATCCGTGGTCGACTGGTTCGGCTTGCCGATGACGAGTCCGCCCT

5521 CTTCGTCACGATGCATCACATCGTCTCGGATGGATGGTCGATGGGGATTTTGATCAACGA

5581 GCTGAGCGTGTTGTATCGGGCGTTCGCACATGGCGAAGCCGATCCGCTAGCACCGTTGCC

5641 AATCCAGTACGCCGATTATGCAAGCTGGCAGCGGCAGTGGTTGACAGGAGATGTGTTGGA

5701 GCAGCAAGCAACCTACTGGCGCAAGACGCTATCGGATGCGCCGGTGTTGCTGGAACTGCC

5761 CACGGATCGTCCGCGTCCGGCTCAGCAGGACCATGCTGGCGCGATGTTGGAGGTCATCGT

5821 CGATCCGCAGCAAGCACAAGCGTTGAAGGCCCTGAGCCAGCGCCATGGCCTGACGCTATA

5881 TATGACCCTCCTGGCGAGTTGGGCGTTGCTGCTATCGCGTCTTTCCGGGCAGGACGATGT

5941 CGTCATCGGCAGCCCAGTGGCCAATCGTGGTCGTTCGGAAACCGAAGGGCTGATCGGGTT

6001 CTTCGTCAACACGCTGGCGTTGCGAGTGGAGTTGTCTGGCTCGCCGACGCTGGCGCAATT

6061 GCTGGCCTCGGTGAAGAACCGCGCACTGCAAGCGCAGGCACATCAGGACATCCCGTTTGA

6121 ACAGGTGGTGGAGTTAGTACAGCCGCCGCGCAGTCTGGCGCATACGCCGCTGTTCCAGGT

6181 GATGTTCTCGTGGCAGAACGCGCCTCAGGGTGAGCTGGATCTTGGTGCAATTGAGGCGAG

6241 CGGTCTGAGCGCAATACAGACGAGCGCGCAGTTCGACCTGTCGTTGTCGTTGGTTGAGAG

6301 TCAGGAGGGGATCATCGGCAGTCTGGCTTATGCCACGGCATTGTTCGAGCGTTCGACGCT

6361 GGAGCGGTGGATGGGCCATTGGCAGCATCTGTTGGATGCGATGGCGGCCGAGGGCGCCGA

6421 ACATCAGGCATTAGATCGTCTGCCGTTGCTGGACGATGCCGAGCGCTATCAGGTGCTGAC

6481 GCAGTGGAATGCAACCGCAGCAGATTATCCGCGCGATGCCTGTGTACATGAATTGTTCGA

6541 GGCACAGGTAGCACGGACACCATCTGCAATTGCGGTGGTGCAAGGCGAAGTGTCGCTGAC

6601 GTATGGCGAGTTGAACGCACGTGCCAACCGTCTGGCGCATTGCCTGCGTGAGTTGGGCGT

6661 ACGCCCAGATGATCGTGTGGCCATCTGCGTGCAGCGTAGTGTGGAAATGGTGGTGGCGTT

6721 GCTTGCGGTGCTGAAGGCGGGCGGTGCGTATGTGCCGTTAGATCCGGCTTATCCACCGGA

6781 GCGACTGGCCTATATGCAGTCAGATTGCGGCGCGGTGGTGGTGTTGACGGATACCGCCAG

6841 CCGCCACTTGGTCGAGCATCCGACTGCTTCAACGGTGGTCGTCGATCTGCAAGCCGACGG

6901 CCAGCGCTGGGCACACCTGCCCGACAGCAATCCGGATCGCAACGCCGTTGGTCTGACCTC

6961 GCGTCATCTGGCGTATGTGATTTATACGTCCGGATCGACCGGTATGCCGAAGGGCGTCAT

7021 GATCGAGCACCGGGGTTGTGTCAACTTATATCATCACTATGCCATTTGCTATTTAAGGAG

7081 TGGCGATAAGGTATTGGTCTTATCTTCTTTTTCCTTTGATTTGACGCTTAAGAATATTAT

7141 TTCCCCATTGTTTTTGGGCTGTGCTGTCGAATTGGCACCGGCGGGTGTGGTGGTCGGATC

7201 ACGTATCCTGAATCTTCTTGAGTCAAGTGGCGCTGCATTGATAAATTGCGCGCCAAGTCA

7261 ATTGCATGGAGTTTTAGATGATTTTGAATCGAAACTAAGAGTAGCCTCGCTAGCGAAACT

7321 TCGCTATATCATACTGGGGGGAGAGAAGATAAAAATTGATCTGATTTCTGAATGGATTTC

7381 AGGTTCTGAGAAATATGTATTTATAAATAGCTATGGGCCAACTGAAATCACCGACGTTGC

7441 GGTCGATGGCGTCATCAAGGGGGGGGATGTTTCAGAGTCGATGCCGATTGGCCGTCCAAT

7501 CGCTAACACGCGCATCTACATCTTGGATATGCACGGTGCACCGGTTCCGATTGGGGCGGT

7561 GGGCGAGTTGTATATCGGCGGCGATGGTGTGGGGCGTGGATATTTGAATCGTGACGATTT

7621 GACCGCCGCGCGCTTCCTCGCGGATCCATTCAGTGCCGATCCGACAGCGCGCATGTATCG

7681 CAGTGGCGATTTAGGGCGCTGGCGAACCGATGGCACGATTGAGTTTGTGGGTCGCAACGA

7741 TCACCAAGTCAAGATTCGTGGTTTCCGTATTGAACTCGGCGAGATCGAGGCAAGATTGAG

7801 CGCACATGCGGATGTGCGCGAGTGCGTGGTGGTGCCCATGGAGGATGCCACAGGCAACGA

7861 CAAGCGACTGGTGGCGTATTTAGTCGCTGCCGAGGGTGTGATGTCCGAGCATCTTGGCGC

7921 AGAAAGCTTGCGTAGTTGGTTGTCGGACACGTTGCCCGATTACATGGTTCCAGCGGCCTA

7981 TGTGCAGTTGGATCGCCTGCCGCTGACTCCCAACGGCAAGTTGGATCGCAACGCATTGCC

8041 CGCACCGGATGAGGCAGCCTACGCGGTGCGTGCGTATGAAGCACCTCAGGGTGCCATCGA

8101 ACAAGCCATTGCGGCGATCTGGCGCGACCTACTTGGCCTGGAGGCCATCGGGCGGCACGA

8161 TAACTTCTTCGCCCTCGGCGGCCACTCGCTGCTGGTAGTGACGCTGACCGAGCGGATGCG

8221 TCAGCAAGGGTTGCAGGCCGATTTGCGCACCCTGTTTGCCACCCCGACGCTTGTGGCACT

8281 GGCCGCAGCGAGCGGTGGCGTGTCGGTCAGCGTTCCGCCCAACGGCATTATGCGCGATAC

8341 CACGCTGATCACACCGGAGATGCTGCCGTTGGCGGTCCTCACTCAGCAACAGATCGATAG

8401 TATCGTCGCGATGACCCCTGGCGGCTCTGCCAATATCCAGGACATCTATCCGCTTGCTCC

8461 ATTGCAGGAAGGCATCTTTTTCCATCATCTGTTGCAGCGCGAAGGCGATGCCTATCTGCA

8521 ACCGAACCTGATCGCGTTCGACAACCGTTCGCGTCTGGACGTGTTTGTCGATGCACTGCA

8581 ATGTGTGATTGATCGTCACGATATCCTGCGCACAGCGGTGGCCTGGGAAGGACTTGCTGC

8641 ACCGATGCAGGTCGTGTGGCGGCATGCGCCACTCCTGATTGAGGAGGTGTGCCTGGACGA

8701 AGCCGATGGCGATGTGGCCATGCAACTGCAATCGCGTTTCGATCCGCGGCACTGGCGTTT

8761 GGATGTACGTCATGCGCCACTGATGCGTGGTTTTACTGCATACGACTCGGTCAATGGTCG

8821 CTGGTTGCTGCAATTGCTCAGCCATCACATGGCCCTGGATCACACCACGCTGGAAATCCT

8881 ATTGGAGGAAGTCCGGTGCCACTTGTGCGGTGATGCCGACAGTTTGCTTCCCCCACTGCC

8941 GTTCCGCAATTTTGTGGCGCAGGCACTGCTGGGCGTGAGCCACGAGGAGCACGAAGCATA

9001 TTTCAGCACGATGCTCGGCGATGTGGATGAGCCCTGTGCACCATTCGGTCTGATCGACAC

9061 ACAGGGCGATGGATCCGACCTGGAAGAGGTGCGGCTCAATCTGCCGGATCGACTGTCGTT

9121 GTCGTTGCGCAACCATGCGCGCACGCTTGGGGTGAGTACGGCCAGCCTGTTCCATCTGGC

9181 CTGGGCGCAAGTGGTGGCGCGCGCCACGGGGCATGAGCGTGTTGTGTTCGGAACGGTGCT

9241 GTTTGGCCGTATGCAGGGCGGAGCGGGAGCTGATCGTGCACTGGGCATGTTCATCAATAC

9301 CTTGCCATTGCGGATCGAGATTGATGGGTCCAGCGTAGTAGAGAGCGTGCGTCTGATGCA

9361 GCAGCGCCTTGCCGGGCTGTTGCGACACGAACATGCGCCGTTGTCGTTAGTGCAGCGTTG

9421 TAGTGGTGTTCCTGCGCCCGCGCCGTTGTTTACCTCACTGTTCAATTATCGGTACAGCGG

9481 CCAGGCAGAAGGCGTTGCCGAGACGATGGCGTGGGAGGGGATCGAGACATTGTCCAGTCA

9541 CGAGCGGACAAATTATCCGCTTGGCGTTTCTGTGGATGATGTGGGATCTGCTTTCGTGCT

9601 GACGGTGCAGGCGCAGCGACCTCTGGTTCCGGAACGGATATGTGCGTTCCTGCTGAAGGC

9661 GCTGGAGAGTCTTGCCAATGCGCTTGCCGATGCGCCAAAGACGCCGGTGTGCGATCTCGA

9721 CGTTCTGCCTGAGGCCGAACGGCATCAGCTGCTGACCCAGTGGAATGCAACCGCGACAGA

9781 TTATCCGCGCGATAAGTGTGTGCACGAGTTGTTCGAGGAGCAAGTGGCGCGGGCACCATT

9841 GGCCATCGCGGTGGTGCAGGGTGAAGTATCGCTGACCTATGCCGAGTTGAACGCGCGTGC

9901 TAACCGTCTGGCGCATTATCTACGCGAATTGGGTGTGCGCCCGGATGATCGTGTGGCGAT

9961 CTGTGTGCAGCGCAGCGTGGAAATGGTCGTGGCGGTGCTTGCGGTGCTGAAGGCGGGTGG

10021 TGCGTATGTGCCGCTTGACCCTGCTTATCCGCCGGATCGAGTGGCTTATATGCAGGTCGA

10081 TTGCGACGCGGTCGCGGTGCTGACAGATTCGGCCAGCCGTCATTTGATCGAGAACAATGC

10141 CACTTCGGTGGTGATCGTTGATCTGCAAGCCGACGCTGCGCAGTGGCAGCATTTGCCCGA

10201 CAGCAACCCCGACCACCACGCGATTGGCCTGACTGCGCGGCATCTGGCGTATGTGATCTA

10261 CACGTCCGGATCGACTGGTAAGCCGAAGGGGTCGATGAACGAGCATCGCAGCGTCGTGAA

10321 TCTTGCGTTGGCGCAAATAGGTGCATTCCATGTTGAAGAAGACAGCCGCATCCTGCAGTT

10381 TTCTTCGTTGAGTTTTGATGCCTTCGCTTCTGAGTTGCTGGTGACGCTGTTTTGTGGAGC

10441 TTCGCTGTATATCGCCGACCAAGGTGATGTATTGGCAGGTGAGACGCTCACGAAACTATT

10501 GGCGGAAAATGAAATCAGTCACGTCACGTTGCCTCCTTCGGTACTGCATCAAATGCCTGA

10561 AGAGCCAAGGCTTTCGTCTCTGAAGACACTCGTCGTTGCCGGCGAAGCCCTCTCTGTGGC

10621 CATCGTGGAACGTTGGGCTGAAGGAAGACGCTTGATCAATGCATACGGTCCCACGGAGAC

10681 GACGGTCTGCGCCTGTGTGCATGAATGCGATGCGAATGTGGCGGGTGCACCACCGATTGG

10741 TCGTCCGATTGCCAATGTGCGGATCTATATTCTGGATATGCACGGCGCACCAGTTCCTGT

10801 TGGAGTGGTTGGCGAGTTGTATATCGGTGGTGATGGTGTGGGACGTGGTTATCTGAATCG

10861 CGACGATTTGACCGCCGCGCGCTTCCTCATGGATCCATTCAGTGCCGATCCGACGGCACG

10921 GATGTATCGCACTGGCGATCTGGGTCGCTGGCATACCGATGGCACGATTGAGTTTGTGGG

10981 ACGCAACGATCAGCAGGTCAAGATCCGTGGTTTCCGTATTGAATTAGGCGAGATCGAGGC

11041 ACGGCTGGGCACACATGCGGATGTGCGCGAGTGTGTTGTGGTGGCGCTGGAAGATGCCGC

11101 GGGCAGCGACAAGCGATTGGTGGCGTATTGGGTTGCGACGCAGGATGCGACGCACGCGCC

11161 GCTTGGCGTGGAAAGCTTGCGCAGTTGGCTGTCGGATACGTTGCCGGATTACATGGTGCC

11221 GGCGGCTTATGTGCAGTTGGATCGCCTACCGCTTACCCCGAACGGCAAGTTGGATCACAA

11281 GGCATTACCCGCGCCGGATGCTACAGCCTATGCCGCGCCTGCGTATGAAGCACCGCAGGG

11341 TGAGGTTGAACACACCATTGCCGCGATCTGGTGTGACCTGCTGGGTCTAGAGAGCATCGG

11401 GCGGCACGACAACTTCTTTGCGCTCGGTGGACATTCGTTGTTGGCGGTGCGAGTTGCCTC

11461 TCGCCTGCGTCAGGAATTGGGTGTCGAGATCGGCGTGGCGGAGTTGTTTGCCAATGCAAC

11521 GCTGAAGGACCTTGCCGCGTGTGTGGCCTCTTCGTCCGCTGCGATCTTGCCGCCGATCCT

11581 GCCCTTGCAGCCGGATGCGCCGCACGTGCTGTCCTTTGCACAGCAACGGCTCTGGTTCCT

11641 GTCGCAGTTCGAGGGTATCAGTCAGGCGTATCACATCAGTGGCGGGCTAAGGTTGCGTGG

11701 TGTGTTGGATGCGCAGGCGTTGCAACGTGCGTTGGATCGCATCGTGGCCCGACATGCGTC

11761 GCTGCGCACGACCTTCGCGCTGGTCGATGGGCAGGCGTTGCAGCAAATCGCTGATGAAGA

11821 AAGCGGCTTCCATTTGCTCACCCACGATCTGTGCGGTGTACCTGATCGTGAGGAAGCGCT

11881 TGAAAAACTGCTGACCGAGGAGGCACATGCGCCATTTGCATTGGAGCAAGGCCCACTCAT

11941 CCGTGGCCGGCTGATACGACTTGCCGATGACGAGTCCGTCCTCTTCGTCACGATGCACCA

12001 CATCGTCTCGGATGGTTGGTCGATGGGAGTTTTGATCAACGAGCTGAGCGTGTTGTATCG

12061 GGCCTTTGCACATGGCGAGGCCGATCCGCTGGCGCCGTTGCCGATCCAGTACACCGATTA

12121 TGCAAGCTGGCAGCGGCAATGGCTGGCGGGCGAGGTACTGGAGCAACAGGCCACGTATTG

12181 GCGTGAGGCACTGTCCGGCGCACCGGTATTGCTGGAACTGCCAACGGATCGTCCACGTCC

12241 AGGGCGTCCAGCGCGTCAGGACCATGCCGGCGCGATGTTGGAGGTGGTCGTCGAGCCGCA

12301 CCAAGCACAGGCGTTGAAGGCGCTGAGCCAGCGCCATGGTCTGACGCTATATATGACCCT

12361 CCTGGCGAGCTGGGCGTTGCTGCTATCGCGTCTATCCGGCCAGGACGATGTGGTGATCGG

12421 CAGTCCGGTGGCTAATCGTGGGAGATCGGAGACAGAAGGACTGATCGGCTTTTTCGTCAA

12481 CACGCTGGCGTTGCGAGTGGAGTTGTCCGGTTCGCCGACGTTGGAGCAACTGCTGGCCTC

12541 GGTGAAGAACCGCACACTGCAAGCGCAGGCACATCAGGATATCCCGTTCGAGCAGGTGGT

12601 CGAACTGCTGCAACCGCCACGCAGTCTGGCGCATGCGCCGCTGTTCCAAGTGATGTTTGC

12661 TTGGCAGAACACGCCGCAGGGGGAGTTGGATCTTGGCGAGCTCGATGCCAGCGGATTGGG

12721 TGTTGCGCAGACGAGCGCGCAGTTCGACCTGTCGTTGTCGTTGGTCGAGAGTGAGGAGGG

12781 GATCGTCGGCAGTCTGGCCTATGCCACGGCATTGTTCGAGCGTTCGACGCTGGAGCGGTG

12841 GATGGGCCATTGGCGGCATCTGTTGGCCGCGATGGTCGCCGAGGGTGCCGAAGATCAGGC

12901 AGTAGATCGTCTGCCGTTGCTGGACGATGCCGAGCGCTATCAGGTGCTGACGCAGTGGAA

12961 TGCAACGGCAGCAGATTATCCACGCGATGCCTGTGTGCACGAGTTGTTCGAGGCACAGGT

13021 GGCGCTTGATCCATCAGCCATCGCGGTAGTGCAGGGCGAGGTATCGCTGACCTATGGCGA

13081 GTTGAACGCGCGTGCCAACCGTCTGGCGCATTACCTGCGCGAGTTGGGCGTGCGCCCGGA

13141 TGATCGTGTGGCGATCTGTGTGCAGCGTAGTGTGGAGATGGTGGTGGCAGTGCTTGCGGT

13201 GCTGAAGGCCGGCGGTGCCTATGTGCCGTTGGATACGGCCTATCCGCCGGAGCGCTTGTC

13261 CTACATGCTCACGGATTGCGGCGCGGTGGCGGTGCTGACGGATGACGCGAGCCGTCCTCT

13321 TGTCGAGGACAATGCCAAATCAGGTGTGATCGTCGATCTGCAAACCGAGGACGAGCGCTG

13381 GCAGCATTTGCCAGACCGCAATCCCGACCGCGATGCCAATGGTCTGACCGCAAATCACCT

13441 TGCATATGTGATCTATACGTCCGGATCGACGGGGATGCCGAAGGGCGCGATGAACGCGCA

13501 TCGCGGCGTCGTCAACCGTCTGGTGTGGATGCAAGAAGCCTACACGCTGGATCGATCCGA

13561 GGTTGTTTTACAAAAAACACCGATCAGCTTCGATGTCTCGGTCTGGGAACTGTTCTGGCC

13621 GCTGCTGAGCGGTGCGCGTGTGCAGTTGGCGCAACCAGAAGGGCATAAAGATCCGGTGTA

13681 TCTAAAGGCGTTGATCCGCGATACGCAGATCACCACCTTGCATTTCGTACCGTCGATGCT

13741 TCGGGCGTTGATCGAGCATGGCGATGATGATCCGTGTACTGGGGTCAAGCGAGTTATTTG

13801 TAGTGGCGAAGCCTTGCCGGCAGTGCTTGCAGAGCGTGCCCAGGCGGTTTTTCCATCATC

13861 TGAGATTTTCAACCTTTATGGACCGACAGAAGCGGCAGTGGATGTCACCGCCTGGCGCTA

13921 TTGTGCGGGATGGGAAAATGCCGCAATTCTCCCGATTGGTCGTCCAATCGCGAACACGCA

13981 GATCTATATCCTTGATGTACAAGGTGCACCGGTTCCGATTGGAGTGGTGGGCGAGTTGTA

14041 CATTGGCGGCGATGGCGTGGGGCGTGGGTATCTGAATCGCGAGGACCTGAGCGCCGAACG

14101 CTTCCTCACCGATCCATTTAGTACCGATCCGACGGCGCGGATGTATCGCACTGGTGATCT

14161 GGGGCGTTGGCGTGCCGATGGCACGATTGAGTTTGTCGGACGCAACGATCACCAAGTCAA

14221 GATCCGTGGTTTCCGAATCGAGTTGGGCGAGATCGAGGCGCGGTTGAGCGCGCATGTGGA

14281 TGTGCGCGAGTGTGTGGTGGTGGCGCTGGAAGATGCCACGGGCAGCGACAAGCGATTGGT

14341 GGCGTATTGGGTTGGTAGGCAGGATGCGACGCACACGCCTCTTGGCGCGGAAAGCTTGCG

14401 CAGTTGGCTGTCGGATACCTTGCCGGATTACATGATCCCAGCAGCCTATGTGCAGTTGGA

14461 TCGCTTGCCGCTGACTCCGAACGGCAAGTTGGATCGCAAGGCATTACCCGCGCCAGATGG

14521 CTCGGCCTATGCAGCGCCTGCGTATGAAGCACCGCAGGGCGCGATTGAACAAACCATTGC

14581 CGCGATTTGGTGTGATCTGCTGGGTCTGGAGAGCATCGGGCGACACGATAACTTCTTCGC

14641 GCTCGGTGGACATTCGTTGTTGGCAGTGCGAGTTGCTTCGCGCCTGCGTCAGGAATTGGG

14701 TGTCGAGATCGGCTTGGCGGAGTTGTTCGCGCATGCAACGCTGAAGGACCTTGCCGCCTG

14761 CGTGGCATCTTCGTCCGCTGCGATCTTGCCGCCGATCCTGCCCTTGCAGCCGGATGCGCC

14821 GCGCGTGCTGTCCTTTGCACAGCAACGGCTCTGGTTCCTGTCGCAGTTCGAGGGTGTCAG

14881 TCAGGCGTATCACATCAGCGGCGGGCTAAGGTTGCGCGGGGCGCTGGATACGCAGGCGCT

14941 GCAGCGTGCGTTGGATCGCATCGTGGCTCGACATGCGTCGCTGCGCACGACCTTCGCGCT

15001 GGTCGATGGGCAGGCGTTGCAGCACATCGCTGCCGAGGAGAGCGGCTTCCATTTAATCGA

15061 TCACGATCTGCGCGGTGTGCCTGATCGTGAGAAAGCGCTCGAAAAACTGCTGACCGAGGA

15121 GGCACATGCGCCATTTGTATTGGAGCAAGGCCCACTCATCCGTGGTCGGCTGATACAACT

15181 TGCCGATGACGAGTCCGTCCTCTTCGTCACGATGCACCACATCGTCTCGGATGGTTGGTC

15241 GATGGGAGTTTTGATCAATGAGCTGAGCGTGTTGTATCGGGCCTTTGCACATGGCGAAGC

15301 CGATCCGCTAGCACCGTTGCCAATCCAGTACGCCGATTATGCAAGCTGGCAGCGGCAATG

15361 GCTGGCGGGCGAGATACTGGAGCAGCAGGCCACATATTGGCGCGAGACGCTGTCTGGTGC

15421 GCCGGTGTTGCTGGAACTGCCAACGGATCGTCCACGTCCAGCGAGACAGGACCATGCCGG

15481 TGCGATGCTGAAGGTGGTCGTCGATCCGCAGCGGACGCATGCACTCAAAGCACTGAGCCA

15541 GCGGCATGGTCTGACGCTATACATGACCCTCCTAGCGAGTTGGGCGCTACTGCTGTCACG

15601 CCTGTCCGGGCAAGACGATGTGGTGATCGGTAGTCCAGTGGCTAATCGTGGTAGATCCGA

15661 GACCGAAGGGCTGATCGGGTTTTTCATGAACATGCTGGCACTGCGGGTGGAGTTCTCCAA

15721 CTCGCCGACGCTTGCGCAATTGCTGGCCTTGGTGAGGGAGCGTGCACTGCAGGCGCAGGC

15781 GCATCAGGACATCCCGTTCGAGCAGGTGGTCGAACTGATTCAACCGCCACGCAGTCTGGC

15841 GCATACGCCACTGTTTCAGGTGATGTTCGCGTGGCAGAACACACCGCAGGGCGCGCTGGA

15901 TCTTGGCGCAATCGAGGTCAGTGGATTGGGTGTTGCACAGACGAGCGCGCAGTTCGACCT

15961 GTCCTTGTCGTTGGTTGAGAGCGAGGAGGGGATCGTCGGTCGTCTGACCTATGCCACCGC

16021 GCTGTTCGAGCATGCGACGGTGCAGCGATGGATGGGCCATTGGCAGCATCTGTTGGATGC

16081 GATGGTGGCCGATGGTGCTGAAGATCGGGCGGTGGACCGTCTGCCGTTGCTGGACGATGC

16141 CGAGCGTCATCAGGTGTTGATGCAGTGGAATGCAACGACGAAGGACTATCCAAGCGATGC

16201 CTGTGTACACGAACTGTTCGAGGCACAGGTGGCACGGACACCATCCGCCATCGCGGTGGT

16261 GCAGGGTGAAGTGTCGCTGACGTATGACGAGTTGAATGCACGTGCCAACTGTCTGGCGCA

16321 TTCTCTGCGGGAGTTGGGTGTATGCCCGGATGATCGCGTGGCGATCTGCATGCAGCGCAG

16381 TGTGGAGATGGTGGTCGCGGTGCTTGCGGTGCTGAAGGCCGGTGGTGCCTATGTACCACT

16441 GGACCCGGCTTATCCTGTCGAACGTCTTGCCTATATGTTGGAAGACAGCGCTCCGGTTGC

16501 GGTTCTGGCGCAGACATCGACCTTGACCCTGCTACTGACCGCATCCGTGCCGATCATCAA

16561 TCTGGATGAGTCGCACTGGCAAGATCGGTCCGTGTCGAATCTGTCGATGGATGGTCTGAC

16621 CTCGGCGCATCTGGCTTACGTGATCTACACCTCCGGCTCCACGGGCCGGCCCAAGGGCGT

16681 GATGATCGAACACCGCAATACGGTGAATCTGCTGGCTTGGGCGCAGCGTTCTTTCGCAGC

16741 ATCGGTCCTGGAAAAAGTCCTGTTCTCCACGTCGTTGAACTTCGACCTATCGGTCTATGA

16801 ATGCTTTGTGCCTTTGGTGTGCGGTGGTGCAATTGAGGTGGTCGACAATCTGCTTGCCAT

16861 GCAGGCAGATGGGCAGGGTGTCACGTTGATTAACACCACGCCTTCCGCATTGAAGGGGTG

16921 GTTGGAATCAGGCGGACGAGGCGAGGGCGTCCATACGGTCAACGTGTGTGGTGAAGTGTT

16981 GAAGCGTCAGGTAGTGGAGGATCTGTTTGCCAAGACGCAAGTCGAACGGCTGTGCAACCT

17041 ATATGGCCCGTCGGAAACCACGACGTATTCGAGTTGGGTATCGATGGAGCGCAGCGACGG

17101 TTTTGTGTCTCATATCGGCACGCCGTTGGACAATACACAGTTCTATGTGCTGGATACGCA

17161 TCGTCAGCCTGTGCCGGTGGGTGTGACGGGTGAACTGTATATCGGTGGTGCCGGTGTAGC

17221 GCGTGGTTATCTGAATCACGGTGATTTGACTGCCGAACGCTTCCTCATGGATCCATTCAG

17281 TGCCGATCCGACGGCGCGGATGTATCGCACTGGTGATCTGGGGCGCTGGCGTGCCGATGG

17341 CGCGCTTGAGTTTGTGGGGCGCAACGACCATCAGGTCAAAATCCGTGGTTTCCGCATCGA

17401 GTTGGGAGAGATCGAGGCGCGGCTGAGCGCGCATGTGGATGTGCGCGAATGTGTGGTGGT

17461 GGCGCTGGAAGATGCCACGGGCAGCGACAAGCGATTGGTGGCGTATTGGGTTGCGACGCA

17521 GGATGCGACGCACGAGTCCCTTGGCGTGGAAAACTTGCGCAGTTGGCTCTCGGACACGTT

17581 GCCGGATTACATGGTGCCGGCGGCTTATGTGCAGTTGGATCGCCTGCCGCTGACCCCGAA

17641 CGGCAAGCTGGATCGCAAGGCATTGCCCGCGCCGGATGCGACAGCTTATGCCGCGCCTGC

17701 GTATGAAGCACCGCAGGGTGAGGTTGAACACACCATTGCCGCGATCTGGTGTGACCTGCT

17761 GGGTCTAGAGAGCATCGGGCGGCACGACAACTTCTTTGCGCTCGGTGGACATTCGTTGTT

17821 GGCGGTGCAGATGGTAAGTCGCCTCAATCACGCTGGGATTGAAAATAAAATTAGTGATTT

17881 GTTCTCCAATCCAATCCTGATGTCGTTGGCGGCACATCTACAAAATGAAGTGTATAGGGG

17941 GAAATTTAATGGATTGATTCCTTTTAGGGAAGAAGGTGATGAGAATCCACTTTTCATGCT

18001 ATATGAGGCATCAGGAAGTGTCCTGTATGCAAAAAATATTGAAAAATTCTTGCGCGCGGG

18061 AATGCCGATTTATGGGATCGAATGTCCTGTGGATTTTTCAATAGATACCATACAAGGCAT

18121 GGCTGCTGATGCGATCAAGAAGATGCGCAATTTGCAGAGTTCAGGTCCATATCGTCTGGC

18181 TGGCTGGTCTTTTGGTGGGCTAGTGGCTTATGAAATGGCAAGTCAACTTGTAGATGCAAA

18241 TTTGCAAGTCGAATTTTTAGGTCTTCTCGACACTAGCCATCTGTCGACTGACGCTAAAAT

18301 TAAGAATTCAATGGACTCTGCACGCAAAATAGATTGCGATTATGTAATTAGAAAGTTGTT

18361 TGAAACAGGATTTGGTCATAAAGTTGATTCGAGTGAAAAAATTAATGAATTAAGTGATCT

18421 TGTTAAGTTGTATCAAGAGATGGGTTTTTCTTCGGATTTTATCAGGGAAGGTGAGTTGAT

18481 GGACTACGCCATGCGCGTCAGTCACCACATCAATGCGTCTATTGCATATTATGCAAAATC

18541 AATTCCGATGCGGGTGCACTTATTTGAGGCGTCCCAATTTTCCGAGGATAATCCATTCCT

18601 GGGGAGTCCAGCTCAGAGTTGGCAAGGCGTATTGCCTGAGAATTTAATTCGCGTCATCGA

18661 AGTGCCTGGCACACATCAAACTATGATGGAGTCACCTAATGTCGAAATTTTGTCACGGGT

18721 TCTTTCAGAGGCTATTAAGAACGGTTTGACTGACCCACAGAGCTGAGGCGCTTGCGGCTG

18781 CTGGAAGAGGAAAATCGCAAGCTCAAACAGTTGGTTGCCGACCTGAGCCTGGACAAGGCG

18841 ATGCTGCAGGAGGTGGTCATAAAAAAGCTCTGAGGCCTGCCCAGAAGCGCGACTGGGTAG

18901 GCCGGCTGAAGGAGCGTTTTGGGTGAGTGAGCGGCATGCGCTGCGTGTCGTTGCAATGTC

18961 GCGCTCGGCGTTTTCCTACAAGGCCAAGGCGCGCGATTGCAGCGCCATCCGTCTGCGGAT

19021 GCGCGAGATCCCCCAGACGTGTGTGCACTTCACTGATGGTGGTGGATCACTTCACTCATG

19081 AGTGTCTGGACATGGTCGTGGACCAGTCGTTGAAAGGCGAGGACGTTGCCGATGCGATGA

19141 CACGGCTGGTGGTGCAGCGGGCAAGCCTGCGGCGATCAAGCTGGACAAAGGGAGTGAGTT

19201 TGCCGGCAAGCTGATGGATCGATGGGGACATGAAAACGGGGGCAGCCGGACTTCTCCCGA

19261 CGCGGAACGCCAACCGAGAACGCCTTGGTGGAAGGCTTCAACGGACGCTTGCGGCAGGAA

19321 GTCAAACTCCGGACTCTAAGCCCGATCACGACTTAAGGCGGTGGGCGTCGGAGTCGCGCC

19381 AGCTCCGCGTCAAGATGTTCGGCTAAGACCGCGTGTCGATGAGTCCGCTATGAAAAGCCT

19441 GGTGACACACTCGGCTTGACAGGTCAGGCCGTCGGTCGCATGATGTAGGTTATTGATTTG

19501 TAAGGTATATGTAATTTATACTGCAGAGTGCCATGATCCAGCTCCTTTTGATGTGTGTTG

19561 ATTCGTTAGAGGGGTTTTTCGGAATTATCTGAGAGCTCACAGTTTGGCATGTCGATATTT

19621 TATGTATTGCCTGGATTTGCTTTTTGCGGATCAATGAATGGGCAGTATAAACCAATTAAA

19681 CGTATGAGGTTTTATTTTTTATGTGCTGGAATGGACAAGCTTCCACGGCCGTAGCTGCTT

19741 GTGGATTATTAGGTTGCGCGTATTCTGCTTATAAAAAAGAGCCGTACGCATTGTGGGGAT

19801 GTCTGGCGTTTTTTTCGCTTATGGAAATACTTCAGGCGTTCACCTATACGGTGATTAACT

19861 CCTGTAGCAACCCCATGAATCAGGTCGCCACGTTACTTGGTTTTCTTCATATCGCGATAC

19921 AGCCGTTTTTCATAAATGCAATATCTCTTTATTTTATTCCTGAGGCGGCAAGGAATAAGG

19981 TGAAGAATTATGCGTATTTCGTCTGTTTTGTTTGTCTCATCTTGATGATTATGAAGATAT

20041 ATCCCTTCGAATGGGCGCATCACGTCCCCAGGGGAGCTGCACTTTGCGCCGACCGTCTAT

20101 GCTCGGTCAGTGGTAACTGGCACATCGCTTGGGAGCTGCCTGTTAACGATATTCTCAACA

20161 TCACCATCTTCAACATCAAGATCGTGTGGGCGCCGTATATTGTCGCCGCCTTCGTCGTTC

20221 CGCTGTTGTATGGCTCGTGGCGCTTCACGATTTTCCACCTGGTGATGGGGCCGTTCCTAT

20281 CCCGACTCACCACGGACAACCCGAATGAATTTCCTGCGGTCTGGTGCTTGCTCTCGATTG

20341 GATTTTTATTGCTGGTGATCAAGACACCTGTGCGTAGTTGGCTTTTTGTCAAGACGGTCT

20401 GGTGGTATAGAGGCGGCGCCCCGAAGAGTCAGTCTGCATAAGCGCGGCAAGCAGGGACTG

20461 ATTCAAGCCGCAAGCGCAACACCTGCAGCCCGATAAGGGCTGCAGGTGTCTTAATTTCAC

20521 CGCCGCAGTTCGGTACAGCCAACTTTGGGTCCGCTCCCTTCTGCCTGTTTTCATGGAGCG

20581 TGTTGGATCCAGCGAGGATCAGCGACGGGTCAGTTGTCGCAGCACGTATTGCAAGAGGCC

20641 GCCGTGGCGGAAGTATTCGACTTCCTTCGGTGTCAGTAGCATCACCGCCACCGTGAAGGT

20701 CTGCGTGCTGCCGTTGGAGGCGGTCGCGGTGACGGTGGCGCGCTTGCTGCTGCCGTCCTG

20761 CAGGCCGGCGATGTGCAGGATCTCGGAGCCGTCCAGGCCCAGTGTCTGCGCGTTCTGTCC

20821 ATCCAGGAACTGCAGTGGCAGGACGCCCATGCCGACCAGGTTGGAGCGGTGGATCCGCTC

20881 GAAGCTCTCGGCGATCACCGCCTTGACCCCGAGCAGGTGGGTGCCCTTGGCCGCCCAGTC

20941 GCGCGAGGAGCCGGTGCCGTATTCTTTGCCGCCGATCACCACCAGCGGCACGCCATCGGC

21001 TTTGTACTGCATCGCCGCGTCGTAGATCGACTGCTTCTGCGGTTGTCCGCCGCCGGCTGG

21061 GTGGTACAGCGTATTGCCGCCTTCTTCGCCGCCGAACATCAGGTTCTTGATGCGGATGTT

21121 GGCGAAAGTGCCGCGGACCATCACTTCGTCATTGCCTCGGCGGCTGCCGTAGCTGTTGAA

21181 GTCCGCCGGTTGCACCCCGCGTTCTTGCAGGTAACGGCCTGCCGGTGAATCTTTCTTGAT

21241 GTTGCCGGCTGGGGAAATATGGTCGGTGGTGATCGAGTCGCCGAATAGCGCGAGGATGCG

21301 CGCGCCATGCACGTCCTCAATGTGGCCGACCTGCATGGTCATGCCGTCGAAGTAGGGTGG

21361 GTTCTTGATGTAGGTCGATGCCTGGTCCCAGGCGTACAGCTCGCCATCGGGCGAGGCGAT

21421 GGTGTTCCAGCGGCTGTCGCCCTTGAACACGTCGGCATAGTTCTGCTCGAACATCTCCGG

21481 GCCGACGGTGGCGGCGATGGTGTCGCCGATGGCTTTGTTGCTTGGCCAGATATCGCGCAG

21541 GTAGACCGGCTGGCCGTCGCGACCGGTGCCCAGCGGCTCGCGGGTGAGATCGATGTCGGT

21601 GGTGCCGGCGATGGCATAGGCGACCACCAGTGGCGGCGAGGCCAGGTAGTTCATTTTCAC

21661 TTCCGGATGCACGCGGCCTTCGAAGTTGCGGTTGCCCGATAGCACCGAGGTCACGACCAA

21721 GTCGTCCTTGGCGATGGCTGCCGACACATCTTCCGGCAGCGGGCCGGAGTTACCGATGCA

21781 GGTGGTGCAGCCATAGCCGACCACGTAGAAGCCGAGCGTCTCCAGGTCGTGCATGACGCC

21841 GGCTTTTTTCAGATAGTCGGTGACCACCAACGAGCCTGGGCCGAGCGAGGTCTTGACCCA

21901 CGGTTGCGCCTTCAGCCCTTTGGCCACGGCATTGCGTGCGAGCAGACCGGCGCCGAGCAT

21961 CACCGCTGGGTTGGAGGTGTTGGTGCAGGAGGTGATCGCGGCGATCACCACTGCACCATC

22021 GCGTAGCTGCCAGCCGGCGCCGCTGGTGTCGGCGTGCTGCGCCTGCGCCGCTTGGGTGCC

22081 GACGGCAGTGCCACCGCCGCCTTCGTTTTTGAGCCGGTCTTCCTGCATGGTGTCGCCGCG

22141 GCGCTTGGCACGCGTCTGGGCGAATGGCACCAGGTTGTCGCGGAAGTTGCGCTGCATGTC

22201 CTCCAGGAGTACGCGGTCCTGCGGCCGCTTGGGGCCGGCCAGCGACGGTTTGACCTGGCT

22261 CATGTCCAGCTCCAGCGTGGCGCTGTAGTGGGCGTGCGGACTCTCGGCGTCGTGCCACAG

22321 GCCCTGTGCTTTGGCGTAGGCTTCGACCAGGGCGATTTGCTCGGCATTGCGTCCGGACAG

22381 GCGCAGGTAAGTCAACGACTCGGCGTCGATCGGGAAGATGCCGCAGGTCGCGCCGTATTC

22441 GGGGGCCATGTTGCCGATGGTGGCGCGGTCGGCCAGCGGCAGGTGTTGCAAGCCATCGCC

22501 AAAGAACTCGACGAACTTGCCGACCACGCCATGTGCGCGCAGCATTTGCGTGACCGTCAG

22561 TACCAGATCGGTGGCGGTGACGCCTTCGGGCAGCTTGCCGCTGAGCTTGAACCCCACCAC

22621 CTGCGGAATCAGCATCGAGGAGGGTTGGCCGAGCATCGCCGCTTCCGCTTCGATGCCGCC

22681 AACGCCCCAGCCGAGGACACCGATGCCGTTGATCATGGTGGTGTGGCTGTCGGTGCCGAA

22741 CACGGTGTCCGGATAGGCGATTGCCTCGCCGTCGCGTTCGCCGGTCATCACCACCCGCGC

22801 CAGGTGTTCGAGATTGACCTGATGCACGATGCCGGTATTGGGTGGCACCACTTTGAAGTT

22861 GTCGAACGCTTTCTGGCCCCAGCGCAGGAAGCCGTAGCGTTCTTTGTTGCGCTGGAACTC

22921 GATCTTGCCGTTGAGGTCGAGGGCGTCGGGTTTGCCGAACACATCGACCTGCACCGAGTG

22981 GTCGATCACCAGTTCCGAAGGGATCAGCGGATTGATCTGCTCGGGCCGCCCGCCGAGTTT

23041 GACCACCGCGTCGCGCATCGCCGCCAGATCCACCACGCAGGGCACGCCGGTGAAGTCCTG

23101 CAACACCACGCGGGCCGGCATGAACGCAATCTCGGTGTCTGGCTCGGCGGTGGGATTCCA

23161 GCGTGCCACCGCTTCGATGTGTTCCTTGCTCACGGTCGCGCCGCCGTCCTCGTGGCGCAG

23221 CAGGTTCTCCAGCAGGATCTTCAGCGAGTAGGGCAGGCGGGCGATGTCGAAGCGCTGGCC

23281 TAGCCGCGACAGACTGAAGTATGTGTAGCGCTTGCCGTTAACCTCCAACGAGGCGCGGCT

23341 GGAAAAGGTGTCGTGCATGATCGAACTCCTATGGCATCGAGGCTGTAGGGACGTACCGCA

23401 GTCTGACTGATTCAGTGTACACGTTGGCTTGCGGCCGGGGATCGCCTACATGCTTGAGTA

23461 TGTGCAATTTAGTATGTAAAATTTGTACGTACTTGAAACGGAGCCGCCATGGAAGCCATC

23521 GTCGCCGAGCGCGGACAGATCACCTTGCCCAAGGCCGTGCGCGATGCTCTGGGTTTGAGC

23581 AAGGGCACGGTGCTGAAAGTGGAGCTAGACGGCGGCCGCATCGTCCTGCGCAAGAGCGTG

23641 GACGATGCGATCTCGCGTGCGCGCGGTCGCTTCAAGCTCGACGGCTTCGCCAGCACCGAT

23701 GAGGCGATGCGGGCGATCCGTGGCCGTGCGCCGGGCGATCCATTCGAGTCGGACGCCACG

23761 GCGTGATCGCGATCGACTCCTCGGTGCTGGTGGACCTGCTGGCCGACAGCCCGCAGGCTG

23821 ATGCCGCCGAAGCCTGTCTGCGCCAGTGCTTGAGTACCGGCCCGGTGGTGGTCTGCGGTA

23881 TCGTCTTGGCCGAGGTCTGCGCGGCGCTGCGCGATGGCGCCGAGGCGCTGTCGGTGCTGG

23941 AGGAAATGAGCATCCGTTTCAATGCCCTGGAATCCAAGTCGGCGTTGCGCGCTGGCGAGA

LOCUS Contig G108 14000 bp DNA linear BCT 30-JAN-2012

DEFINITION Xanthomonas spp. strain XaS3 genomic region encoding NRPS.

SOURCE Xanthomonas spp. strain XaS3

ORGANISM Xanthomonas spp. strain XaS3

Bacteria; Proteobacteria; Gammaproteobacteria; Xanthomonadales;

Xanthomonadaceae; Xanthomonas.

REFERENCE Contig G108 (bases 1 to 14000)

AUTHORS Royer et al. Submitted to BMC Genomics.

TITLE Genome mining indicates that the genus Xanthomonas is a promising

reservoir for new bioactive non-ribosomally synthesized peptides

JOURNAL Submitted to BMC Genomics.

REFERENCE 2 (bases 1 to 14000)

FEATURES Location/Qualifiers

source 1..14000

/organism='Xanthomonas spp.'

/mol_type='other DNA'

/strain='XaS3'

/isolation_source='sugarcane leaf water droplets'

/host='Sugarcane'

/db_xref='taxon: GPE 39'

/country='Guadeloupe'

/collection_date='2003'

/collected_by='Rosiane BOISNE-NOC'

/note='[cultured bacterial source]'

gene 819..2402

/gene ='staM'

CDS 819..2402

/gene ='staM'

/note=' putative amino acid β-hydroxylase'

/codon_start=1

/translation='MMTSPWFRLSEHVRLLPRVNGWFAHPYLISPLTFGLYTQHSHLSMMESFLEDPEQHRAALREPDMRGGPFIDHAGDVADIRAFRDQTLQRCATQLQYADAISAVYSVLQSEAKGAGLAGLYAKFDPLIRDKLEIFYDVSKQPGVRFMERQFYASSAYDPSLQSAVLEPVSDQERAFALSTPQLPSAAGSLELNAPFADPFWDQLCGGMADAEALIDLILSHAPSGTTREDARALLTDAPLPEHPAPDGVRVRYFGHACVLIEGAGVSILIDPLISYPGECAIDHFTFDDLPAKIDYLLITHPHQDHVVMESLLRIRHRVGTVVVGRAGGGDLQDISLKLCLEQCGFAHVVELADYEELRFPRGRIVGAPFYGEHADLDIRAKLVHAVELDGKVCVLFADSRPPTVECYAQLKAMFPQIHCMFLGMECVGAPATWLYGPLLQKMLTRGEDQSRRLDGCDHALASALQAFFLPERLYVYAMGAEPWVTHITSILYSEDLPQFREARQLEATARAAGQHAELLFGRCEITL'

gene 2420..4015

/gene ='staM''

CDS 2420..4015

/gene ='staM''

/note='putative amino acid β-hydroxylase'

/codon_start=1

/translation='MSDARGYLRANAVARPLLNHWILWDMLIPPAQAALVVAKQQVPILESYLRAPEQHAQAARNPALMGGPWINYPTPKTAEIGQLLERTRAVQADALALAAALQALDDLLRQQATGQSLEALYAQIPDTLAGLVELVYDLNDHPGLRVLEGVLYRSRFYRRDLQQFAFDLVEHDWLPYERSTPVLESPDTPVLTLPWNDDRLDMLFSAERTPVVIDALAEQLGISATQRAGFARLFDTAPPTQRHSPPAAGMRIRYFGHACLLIESAHTSILTDPFVAYDYPADPPRYTLADLPARIDYALITHGHSDHIRPEILLRLRHRIGTVVVPHSAGRRLQDPSLKLMLHALGFERVIELHEFERIALADGAITALPFLGEHSDLDIQGKAGYHIRLQGHSAACLADSCNLDPHLYRHVAAELGPIDALFLGMECEGSPLSWGYGHLLSRRIDPKLDRSRRDRGSHADEAIALIACWPARSAYVYAMGQEPWLNHVLAINQSGEHLGLREAERFLSHCRTHGIDGERLFAMRELVLSAH'

gene complement (4118..4333)

/gene ='mbtH'

CDS complement (4118..4333)

/gene ='mbtH'

/codon_start=1

/translation='MSSQDDEQDQNYVVVINHEEQYALWPDYAEVPNGWSVVFGSNPKQACLDYVNEHWTDMRPKSLRDAMAAAGH'

gene complement (4438..6168)

/gene ='ABC'

CDS complement (4438..6168)

/gene ='ABC'

/note='cyclic peptide ABC transporter'

/codon_start=1

/translation='MFIRCYLRRHAVTLAGMSLLSAASAGASMLLLDYLNATATNLLHTPMGAALLHGALLLGTALVVRLLSARLAAHVGSGLMAELRGELSTRFLELPLEQLMHRKHAVFGALIGDVGRLTQMIQMGPVLLTNSLLSVGGLLYLSWVSLPLFAVVVPFIGLSGVLFYFTRRFTGPAFDRMRKAEERLNGLLRTLVEGKKELTLSPARAGHFTHMQLRPAIENARRTQFDTSMHWGISDACAELIGYGWVLAAILAGRYLFAVPSPTILHFVITGLFISGPLNALFDLGAQVSGASASVRHLREVGLDDAASVPPAPALAAHAQADRVPVSVEATWTTLRLERVIYRYTGANGDHFQFGPVDLSVRRGETVFVTGGNGSGKSTLLLLLSGLLQPSEGRILVDGRVLGEDLALAQYRTMFSAVFFDFMLFEHVIGADAAPADPAQVQAWLERVNLDAKVNFDHIHGVFSQVELSQGQRKRLALVQACLDDRQIMLFDELTADQDQAFRERFYTVLLPALRASGKTLVLVTHDQGYRQVADRVLALDYGCLAESSLPHKEPGDPHTEAPTLHGRQAQGIVSGA'

gene complement (6200..6994)

/gene ='DpgD'

CDS complement (6200..6994)

/gene ='DpgD'

/codon_start=1

/translation='MKAMPVVFERDGHVARITLNRPRVLNALDLATHAALADAWDSFERDDTLWVAVLSGSGERAFSVGQDLKELAVRLHAGAPSSSFGSQGAPGWPRLTERFDLCKPVIAKVNGLALGGGFELALACDIIVAADTAEFALPEARLGLIPGAGGVFRLTRQLPYRTAMGYLLSGRRMPAARALDLGLVNEVVAADQLDACVERWVQDLLACAPLSLRAIKQAAAASANLPLAEAFATRYSWEERRRLSQDSREGPLAFVEKRPPRWSGQ'

gene complement (6994..8313)

/gene ='HpgT'

CDS complement (6994..8313)

/gene ='HpgT'

/codon_start=1

/translation='MAAVPPPAPAMLEPAELHPCLDEPVLGAIDFLNEVIDRYPEAISFAPGAPAATLLEALDLNVYLQGYLAHLMRVEGLTEQQVRRRLFQYGPSRGIINALLAQALRQDQGLDVAPSHIVVTVGFQEAMFLVLRALFAQSSDVLAVVQPCFVGAMGAARTLAIPLLGIDEVDGRVDLDRLHRACTESRAQGRRVRALYVAPDISNPSGSLLDRAQRQALLDAADAHDFYVLEDSTYGFTLRPEHAVPSLKAMDRSGRVIHLGTFAKIALPGIRVGFVIADQDVAGQPPRVLADALSALKSMVTVNTSPVCQAIAAGMLLASGGSLAALGADRAVFYRGKLACLLAALQRELGDVVARHPALHWNRPQGGFFVCLHLGVTVDTALLELAARDYGVLWTPMRMFYVDEGGQQTLRLSCSYLSDAQIEEGVKRLRRFLCDPRVLG'

gene complement (8337..9611)

/gene ='DpgC'

CDS complement (8337..9611)

/gene ='DpgC'

/codon_start=1

/translation='MTPLAEQLAQVRAVAAWGEQQLLALPAPAQRDQGQAQRAAAVHARCRAVRMEFMRQHGGWLYAALTQAHGLSLRLDEVLRAAADAVPGLLPSQAQWAREHACIQAEKEGWEIEQAIVLWGVLRLPDCGHHLIDSMLRPTARALALLPQFRNDAQVQIGPVHVQRHDGAAWLTVANQTGLNAEDNALVEAMEVAVDLALLDDEVMVGVVRGGPMEHPKYRDRRVFCSGINLQHLYQGNISFVDFLLRREFTYLNKIRRGLCWPGADVREQGIEKPWVAAVDAFAIGGGLQLMLTFDHVVAERQAYFVLPAAKEGIVPGSANLRLGARVGHRVSRDMILRGRIIQAEEPDAAGLADDVVASDAMDATIAHAVQRMRSEAVAPNRRMLRVAEESPEDFRRYMAEFVLVQSSRIYANDVLARLRQRWAA'

gene complement (9611..10354)

/gene ='DpgB'

CDS complement (9611..10354)

/gene ='DpgB'

/codon_start=1

/translation='MSDSSSPVLHLVHLEIDATSVLSDELTQRLGRACDEVEDASTPAVLLLHLRGAATLPVEASWPGPVNLDVIGRWEKAMRRFERLEGVSLACIDNACTAGMLDLLLAVDRRIAVPGASIRMHSRGEAAWPSMAMRRMSTRMGIDAARTVFLFETQLGCARMAQLGVVDQVSEDAQGAIGAWLASLAEVDLQALPGRRLLLLEGAAHSYEQAVGQHLAACDMELRRRQRRLAAQATAEHPHGTCMPMASA'

gene complement (10399..11499)

/gene ='DpgA'

CDS complement (10399..11499)

/gene ='DpgA'

/codon_start=1

/translation='MNFNAHVASTSTPTFIASRIRGVGTATPARRYSQSDVLRRFGIEDRRRRQVFLNNGIDSRHLALPESDEAGNAPRETQARLLDKHRDIGLEIGERALTRCLQSIGASLDDVRYLCCVTTTGLLTPGFSSLLIQRLGMRQDCVRLDVVGMGCNAGLNGFNAAVNWTHANAGKLAVLLCIEVCSAAYVDDDGIETAVVNSLFGDGAAALAVIADGDERGDVHAGPRVCKFASQVIPEALDAMRFAWDETHGKFHFRLHKDVPYVVGANAPTVVQRLLEGTGLRRRDIAHWLVHSGGRKVIDAICANLMLTSHDMRHTIEVLREHGNMSSGSFLFSYARLLDEGQIRPGDWGVMMTMGPGSSIETALLRW'

gene 11951..13998>

/gene ='NRPS'

CDS 11951..13998>

/gene ='NRPS'

/note='incomplete NRPS, no stop codon'

/codon_start=1

/translation='MDNSAKSLPASFVQKRMWLLARLDPQASVTYHIANGVRLTGALDIHALQAALDRIVARHEVLRTSLIEVNGQIRQRIHPSMRFTLVHEDARACATDPAALARIAQQEAVRRFDFTLGAPVRGRLLRLAAQEHILLLTFHHIACDGWSIGVLLRELESLYGAFVRQQADTLPPLPIQYADYAIWQSEQLQGDTLEAQLRFWTTQLADAPTLLPLPTDRARPPLQDYRGASVDTLLPPALGQQLNALARRHGCSLFATVLAGWALLLTRISASNDVVIGTPTAGRTHPDLEALIGCFINTLALRFTLRGTPTVAQWLAHVREVVLNAQDHQELPFERVVEALQPSRSLSHTPIFQTLFSLDGFSRQQSLQLPGLHLEPLPDTADMCAFDLILTMQESETGLRARLKYPVALFDRDTMERRLAQFVTLLQAMVADDTQSVAQLPLLPPQERTQLQHMSQSAPAVDLPQASLHALFAQQAARTPEAPAIVSATGTLSYAAVLAQAHAVAQQLIAIGVRPGDRVAVVLPRAAHLIVAELGVLHCGAAYVPLDPAHPSERLAELVTHCAACAVLSERAIALPPLQQPRLDLDHAAYVPLDPAHPSERLAELLAHCAACAVLSERAIALPPLQQPRLDLDQTLSYAAVLAQSHAVAQQLIAIGVRPGDRVAVVLPRAAHLIVAELGVLHC'

ORIGIN

1 CGCCGGTTCGCTGACCTACCTGGGCGCGCTGTTCCTGGTGCTGCTGGCACCGCTGTTCGC

61 GGCGCTGTGGGCGTGGCTGGCGCGGCGCGGGTGCGAACCGTCCAAGCCGGCCAAGTCGGC

121 GCTGGGCCTGCTACTGGCCGGACTCTCGTTCATCCCGTTGGCACTGGCCGCGCAGCAGGT

181 AGGCAGCAGCGGGCAGGTTGCCAGCGTCTGGTGGCTGGTGCTGGCCTACTTTGTGCTGGA

241 GGTCGGCGAGATGTGCCTGTCACCGGTCGGCCTATCGGCGGTGAGCCAACTTGCGATGCC

301 GCGCGTGGTCAGCCTGATGATGGGCACCTGGTTCCTGGCGACCGCGTTCTCGGAGACGCT

361 GGCGGCGCTGTTCGGCAAGCTGGCTGCGATCGAGGTGCCGGATGGTCAGTCGCTCGACCT

421 CGCCGACGCCGCCGCCAAGTACGCGCATCTGTTCTGGCTGATGTTGTGGATCGGCGCCGG

481 CTGTGCGGTGCTGGCCTTCCTCGCCGCCCCGCTATTGCGGCGGATGATGCACGGGGTGCG

541 CTAAGAGGAGCTCACAAAACGACTGCGTCGCCGCCGCAGGGGCGCGGGCAGTGCTCGGAA

601 TCGGTATGGACCGCCTGTACACGGCGCTTCCTGCGCGCTGTCCGCACCCACCCGACAGCG

661 ACTGGCGAGGTTTTATTAGCCACTCTAAAAATCCCGAACCGCACCTGCCCCCCCGCGTGG

721 CGCGCTGCGGCAAGCCGCTGCCACGATGGTGCAAGCCGCCGCGCGCCCTGCAATGCCAAA

781 CTGCACGCCCACGCTGCCGCACGTTTCTAGGCCCGCCCATGATGACATCCCCCTGGTTCC

841 GGCTCTCCGAGCATGTCCGTCTGCTTCCGCGCGTCAACGGCTGGTTTGCGCATCCCTATC

901 TGATCTCGCCGTTGACCTTCGGCCTGTACACGCAACACTCGCATCTATCGATGATGGAGT

961 CGTTTCTGGAGGATCCGGAGCAGCACCGGGCGGCGCTGCGCGAGCCGGACATGCGCGGCG

1021 GCCCCTTCATCGATCATGCCGGCGATGTGGCCGACATCCGCGCTTTCCGCGACCAGACCC

1081 TACAACGCTGCGCGACGCAGTTGCAGTATGCCGATGCGATCAGCGCGGTCTACAGTGTGT

1141 TGCAGAGCGAGGCCAAGGGTGCCGGCCTGGCCGGGCTATACGCCAAGTTCGACCCGCTGA

1201 TCCGCGACAAGCTGGAAATCTTCTACGACGTCAGCAAGCAGCCTGGCGTGCGCTTCATGG

1261 AGCGCCAGTTCTACGCCAGCAGCGCCTACGACCCCAGCCTGCAGAGTGCGGTGCTGGAAC

1321 CGGTCAGCGACCAGGAGCGCGCGTTCGCGCTCAGCACGCCGCAATTGCCATCGGCCGCCG

1381 GCTCGCTGGAATTGAACGCGCCGTTCGCCGACCCGTTCTGGGACCAGTTGTGCGGCGGCA

1441 TGGCCGATGCCGAGGCGCTGATCGATCTGATCCTGAGTCACGCGCCGAGCGGCACCACGC

1501 GCGAGGACGCCCGCGCGTTGCTGACCGACGCGCCGCTGCCCGAACATCCGGCGCCGGACG

1561 GCGTGCGCGTGCGTTACTTCGGCCATGCCTGCGTGCTGATCGAAGGCGCGGGCGTGAGCA

1621 TCCTGATCGATCCGCTGATCAGCTACCCCGGCGAATGCGCCATCGACCATTTCACCTTCG

1681 ACGATCTACCGGCGAAGATCGATTACCTGCTGATTACCCATCCGCACCAGGACCATGTGG

1741 TGATGGAATCGCTGCTGCGCATCCGTCACCGCGTCGGCACCGTGGTGGTGGGACGCGCCG

1801 GCGGCGGCGATTTGCAGGATATCTCGCTGAAGCTGTGCCTGGAGCAATGCGGCTTTGCCC

1861 ATGTGGTGGAACTGGCCGACTACGAAGAACTGCGCTTCCCGCGCGGAAGAATCGTCGGCG

1921 CACCGTTCTACGGCGAGCATGCGGATCTGGATATCCGCGCCAAACTGGTTCACGCAGTGG

1981 AATTGGATGGGAAGGTCTGCGTGCTGTTCGCCGACTCGCGTCCACCGACAGTCGAATGCT

2041 ATGCGCAACTGAAAGCCATGTTTCCGCAGATCCACTGCATGTTCCTGGGCATGGAGTGTG

2101 TCGGCGCGCCAGCGACCTGGCTGTACGGCCCGCTGCTGCAGAAGATGCTGACCCGTGGCG

2161 AGGATCAATCGCGCCGGCTCGATGGCTGCGACCACGCCCTGGCCAGCGCGCTGCAGGCGT

2221 TCTTCCTACCCGAGCGGCTCTACGTCTACGCCATGGGTGCCGAACCCTGGGTGACCCACA

2281 TCACCAGCATCCTGTATTCGGAGGATCTGCCGCAATTCCGCGAAGCACGCCAATTGGAAG

2341 CGACCGCACGCGCAGCGGGCCAGCATGCCGAACTGCTATTCGGCCGTTGCGAAATCACCC

2401 TCTGAGTCAGGCACGCGCCATGTCCGATGCACGCGGTTACTTGCGCGCCAATGCGGTGGC

2461 GCGCCCATTGCTGAACCACTGGATTCTATGGGACATGCTGATTCCACCGGCGCAGGCGGC

2521 GCTGGTGGTGGCCAAGCAGCAAGTGCCGATTCTGGAATCGTATCTGCGCGCGCCCGAACA

2581 GCATGCGCAGGCGGCGCGGAACCCGGCGCTGATGGGGGGGCCATGGATCAACTACCCCAC

2641 CCCCAAGACTGCCGAGATCGGCCAGTTGCTCGAACGCACCCGCGCCGTGCAAGCCGATGC

2701 ACTCGCCCTGGCGGCGGCGCTGCAAGCACTGGACGACCTGCTGCGCCAACAGGCCACCGG

2761 GCAATCGCTGGAAGCGCTGTACGCGCAGATTCCCGACACGCTGGCCGGACTGGTGGAACT

2821 GGTCTACGACCTCAACGATCATCCCGGCTTGCGTGTCCTGGAAGGCGTGCTGTATCGCAG

2881 CCGGTTCTATCGACGCGACCTGCAACAGTTCGCCTTCGACCTGGTCGAGCACGATTGGTT

2941 GCCCTACGAACGCAGCACCCCGGTACTGGAATCACCCGATACGCCAGTGCTGACCCTGCC

3001 GTGGAACGATGATCGGCTGGACATGCTGTTTTCCGCCGAGCGCACTCCCGTCGTGATCGA

3061 CGCCTTGGCCGAGCAGCTCGGCATCAGCGCAACGCAGCGCGCCGGATTCGCGCGTTTGTT

3121 CGATACCGCGCCGCCCACGCAACGGCATTCCCCACCCGCGGCCGGCATGCGGATCCGTTA

3181 TTTCGGCCATGCTTGCCTGTTGATCGAGTCGGCGCATACCAGCATCCTCACCGATCCGTT

3241 CGTCGCCTACGACTATCCGGCCGACCCGCCACGCTACACGCTGGCCGACCTGCCCGCGCG

3301 CATCGACTATGCGCTCATCACACATGGTCATAGCGACCATATCCGTCCGGAAATCCTGCT

3361 GCGGCTCCGGCATCGGATCGGCACCGTGGTGGTGCCACACAGCGCCGGGCGGCGCCTGCA

3421 GGACCCCTCGCTGAAACTGATGCTGCATGCGCTGGGCTTCGAGCGGGTGATCGAGCTGCA

3481 CGAGTTCGAACGCATCGCACTGGCCGATGGCGCGATCACCGCGCTGCCGTTCCTCGGCGA

3541 ACACAGCGATCTGGATATCCAGGGCAAGGCCGGCTACCACATTCGTCTGCAAGGCCATAG

3601 CGCGGCCTGCCTGGCCGACTCCTGCAACCTCGATCCGCATCTCTACCGGCATGTGGCCGC

3661 CGAGCTTGGCCCCATCGATGCCCTGTTCCTGGGGATGGAATGCGAAGGGTCGCCGCTGAG

3721 CTGGGGCTATGGGCATTTATTGAGCCGACGCATCGATCCGAAACTCGATCGCAGCCGACG

3781 CGATCGCGGCTCGCATGCGGACGAAGCCATCGCCCTGATCGCATGCTGGCCGGCGCGCAG

3841 CGCTTACGTGTATGCAATGGGCCAGGAGCCCTGGCTGAATCATGTGCTGGCGATCAACCA

3901 ATCCGGCGAACACTTGGGCTTGCGTGAGGCCGAGCGGTTCCTGAGCCACTGCCGCACCCA

3961 CGGCATCGACGGTGAGCGCCTGTTCGCGATGCGGGAACTGGTGCTGTCGGCGCATTGAGC

4021 AGGAGCAACACAACGGCGCATGGCGCGCGCCCTCACCAGCCGCCCCCTCCCGACGACGAC

4081 ACACGCCACCAGGAGGGGGAACGCGATGAGACGTTCAGTGTCCCGCCGCCGCCATCGCGT

4141 CGCGCAGGCTTTTCGGACGCATATCGGTCCAGTGCTCGTTGACATAGTCCAGGCAGGCCT

4201 GCTTCGGATTCGAGCCGAACACCACCGACCACCCGTTCGGGACCTCGGCATAATCCGGCC

4261 ACAATGCATATTGCTCCTCGTGGTTGATTACCACGACGTAGTTCTGATCTTGTTCGTCGT

4321 CTTGGCTTGACATCTGTGCGTCCTCCGTTAGGAATGAGTGGCGGCATGTTGCACGCATCT

4381 GACGATGCGCGGGGCGCCGGCCGCACGTTCGACGCGCCCGTGAATCCGGTTCGATCAGGC

4441 GCCCGACACGATACCCTGAGCCTGTCGGCCGTGCAGCGTCGGTGCCTCGGTGTGCGGGTC

4501 GCCTGGTTCCTTATGCGGCAGCGACGATTCGGCCAGGCATCCATAGTCCAGCGCCAACAC

4561 ACGGTCGGCGACCTGGCGGTAACCCTGGTCGTGGGTGACCAACACCAATGTCTTGCCACT

4621 GGCGCGCAATGCCGGCAACAACACCGTATAGAAGCGCTCGCGAAACGCCTGGTCCTGATC

4681 GGCGGTGAGTTCGTCGAACAGCATGATCTGACGGTCGTCCAAGCACGCTTGCACCAGGGC

4741 CAGGCGCTTGCGCTGCCCCTGCGACAACTCCACCTGCGAGAACACACCGTGGATGTGATC

4801 GAAATTAACCTTCGCATCCAGATTCACCCGCTCCAGCCAAGCCTGCACCTGGGCCGGATC

4861 GGCCGGCGCGGCATCGGCGCCGATCACGTGTTCGAACAACATGAAATCGAAGAAGACCGC

4921 GCTGAACATGGTCCGATATTGCGCCAACGCAAGATCTTCGCCCAGCACGCGGCCATCGAC

4981 GAGAATGCGCCCCTCGCTGGGCTGCAGCAATCCGCTGAGCAACAACAACAACGTCGATTT

5041 GCCGCTGCCATTACCGCCGGTGACGAACACGGTCTCGCCGCGCCGCACGCTCAGATCCAC

5101 CGGACCGAACTGAAAGTGGTCGCCATTGGCGCCGGTATAGCGATAGATCACCCGCTCCAG

5161 ACGCAGCGTGGTCCATGTGGCCTCCACCGATACGGGCACGCGGTCGGCCTGGGCGTGCGC

5221 GGCCAGTGCGGGAGCAGGCGGTACGCTCGCCGCATCGTCGAGCCCCACCTCGCGCAGATG

5281 CCGCACGCTCGCCGATGCGCCACTGACTTGCGCGCCAAGATCGAACAACGCATTGAGCGG

5341 GCCGCTGATGAACAAGCCGGTGATGACGAAGTGCAAAATCGTCGGCGACGGTACCGCGAA

5401 CAGATAACGCCCTGCCAGGATCGCCGCCAATACCCAGCCATACCCGATCAACTCGGCGCA

5461 GGCATCGCTGATGCCCCAATGCATGCTGGTGTCGAACTGCGTCCGGCGCGCGTTCTCGAT

5521 TGCCGGGCGCAACTGCATGTGCGTGAAATGCCCGGCACGCGCCGGCGACAAGGTCAGTTC

5581 CTTCTTGCCCTCCACCAACGTGCGCAGCAGTCCATTGAGGCGCTCCTCGGCCTTGCGCAT

5641 GCGGTCGAAAGCCGGCCCGGTGAAGCGCCGAGTGAAATAGAACAACACACCCGACAGGCC

5701 GATGAACGGCACCACCACCGCGAACAACGGCAGCGAAACCCAGGACAGATACAGCAGCCC

5761 TCCCACCGACAGCAGGCTATTGGTCAACAGCACCGGCCCCATCTGGATCATCTGCGTCAG

5821 CCGACCGACATCGCCGATCAGCGCGCCGAACACGGCGTGCTTGCGATGCATCAATTGCTC

5881 CAGCGGCAACTCCAGAAAACGCGTGGACAGTTCCCCGCGCAGTTCCGCCATCAATCCGCT

5941 GCCCACGTGCGCCGCAAGGCGCGCGGACAACAGGCGCACCACCAGCGCGGTGCCAAGCAG

6001 CAACGCACCGTGCAGCAGCGCGGCGCCCATCGGCGTGTGCAGCAGGTTGGTCGCGGTCGC

6061 GTTGAGATAATCCAGCAGCAACATCGAGGCGCCCGCACTGGCCGCCGACAGCAGCGACAT

6121 GCCCGCCAAGGTCACGGCATGACGGCGTAGATAGCACCGAATGAACATCAGACCACTCCC

6181 TCTGGCAAGCGCGCGCTCATTGCCCACTCCAACGCGGCGGACGCTTTTCCACGAAGGCCA

6241 GCGGCCCTTCGCGGCTGTCCTGACTGAGGCGCCGTCGTTCTTCCCAGGAATACCGGGTGG

6301 CGAACGCCTCGGCCAGCGGCAGGTTGGCCGACGCCGCCGCGGCTTGTTTGATCGCGCGCA

6361 GCGACAGCGGCGCGCACGCCAGTAGGTCCTGCACCCAGCGCTCCACGCAGGCATCCAACT

6421 GGTCCGCCGCGACCACCTCGTTGACCAATCCCAAGTCCAAGGCCCGCGCGGCCGGCATCC

6481 GCCGACCGCTCAGCAGATAGCCCATCGCGGTGCGATACGGCAGCTGCCGGGTCAGGCGGA

6541 ACACGCCACCGGCACCGGGGATCAGGCCGAGCCGCGCCTCGGGCAAGGCGAATTCGGCGG

6601 TGTCCGCGGCGACGATGATGTCGCACGCCAAAGCCAACTCGAAGCCACCGCCCAGCGCCA

6661 GGCCATTGACCTTGGCGATCACCGGCTTACACAGATCAAAGCGCTCGGTCAGACGTGGCC

6721 AACCCGGCGCGCCCTGACTGCCGAACGACGACGACGGCGCACCGGCGTGCAGACGCACCG

6781 CCAATTCCTTCAAATCCTGTCCGACCGAGAACGCGCGCTCGCCACTGCCGCTGAGCACCG

6841 CCACCCACAGCGTGTCGTCGCGCTCGAAGCTGTCCCAGGCATCGGCCAACGCCGCATGCG

6901 TGGCCAGATCGAGCGCATTCAACACCCGCGGACGGTTAAGGGTGATACGCGCGACATGTC

6961 CATCGCGTTCGAACACTACCGGCATCGCCTTCATCCGAGCACCCGCGGATCGCACAGGAA

7021 CCGGCGCAGACGCTTCACGCCCTCTTCGATTTGCGCATCGCTCAGATAACTGCACGACAG

7081 ACGCAGCGTCTGCTGCCCGCCCTCGTCCACATAGAACATCCGCATCGGTGTCCACAGCAC

7141 GCCGTAATCGCGCGCGGCCAGCTCCAGTAAGGCCGTATCCACCGTCACCCCCAGGTGCAG

7201 GCAGACGAAAAACCCACCTTGTGGCCGGTTCCAATGCAAGGCCGGATGTCTGGCGACCAC

7261 ATCGCCGAGTTCGCGCTGCAACGCCGCCAACAGACAAGCCAGCTTGCCCCGATAGAACAC

7321 CGCGCGATCCGCACCCAAGGCCGCGAGCGAACCGCCACTGGCCAGCAGCATGCCCGCGGC

7381 AATCGCCTGGCAGACCGGCGAGGTATTGACCGTCACCATGCTTTTGAGCGCGGACAGCGC

7441 ATCGGCCAGCACGCGCGGCGGCTGCCCGGCCACGTCCTGATCGGCGATCACGAAACCGAC

7501 GCGGATGCCCGGCAAGGCAATCTTGGCGAACGTGCCTAGATGGATCACGCGCCCGCTGCG

7561 GTCCATCGCCTTCAACGAGGGAACCGCATGCTCGGGGCGGAGGGTAAAACCGTAGGTGCT

7621 GTCTTCCAGCACGTAGAAATCGTGCGCGTCTGCCGCATCCAGCAAGGCCTGGCGCTGCGC

7681 GCGATCCAGCAGACTGCCGGAGGGATTGGAGATATCCGGCGCCACGTACAATGCGCGTAC

7741 CCGGCGGCCCTGCGCACGCGACTCGGTACAGGCGCGATGCAGGCGATCCAGATCGACGCG

7801 ACCGTCGACCTCGTCGATGCCCAGCAGCGGGATCGCGAGGGTGCGCGCCGCGCCCATCGC

7861 ACCGACGAAACACGGCTGCACCACCGCCAGGACGTCGCTTGACTGGGCGAACAAGGCCCG

7921 TAACACCAGGAACATCGCTTCCTGAAAACCCACGGTGACCACGATGTGCGACGGCGCGAC

7981 GTCCAGCCCTTGATCCTGGCGCAGCGCCTGCGCGAGCAGCGCATTGATGATGCCGCGACT

8041 GGGGCCGTACTGAAACAGACGCCGCCGCACCTGCTGTTCGGTGAGCCCCTCGACACGCAT

8101 CAGATGTGCGAGATAGCCCTGCAGATAAACATTCAGATCCAACGCTTCCAGCAACGTCGC

8161 CGCCGGTGCGCCAGGCGCGAACGAGATCGCCTCCGGATAGCGATCGATCACCTCGTTGAG

8221 AAAATCGATCGCTCCCAGCACCGGCTCGTCGAGGCAGGGATGCAACTCGGCCGGCTCCAG

8281 CATCGCCGGTGCAGGCGGTGGCACAGCGGCCACGCCGGCATCGCCTTGCCCACTCATGCG

8341 GCCCACCGCTGACGCAGACGGGCCAGCACATCGTTGGCATAGATACGACTGGATTGCACC

8401 AACACGAACTCGGCCATGTAACGGCGGAAATCCTCCGGCGATTCTTCCGCCACCCGCAAC

8461 ATGCGCCGATTGGGCGCCACCGCCTCGCTACGCATGCGCTGCACGGCATGGGCGATGGTC

8521 GCGTCCATGGCATCGCTGGCGACGACGTCGTCGGCCAGGCCCGCGGCATCGGGTTCCTCG

8581 GCCTGGATGATGCGTCCGCGCAGGATCATGTCGCGACTGACCCGATGCCCCACCCGCGCG

8641 CCTAAACGCAGATTGGCCGAACCCGGCACGATGCCCTCCTTGGCCGCGGGCAGCACGAAA

8701 TAGGCCTGGCGTTCGGCCACCACATGATCGAAGGTCAACATCAATTGCAGGCCGCCGCCA

8761 ATGGCGAACGCATCCACCGCCGCCACCCAGGGTTTCTCGATGCCCTGCTCGCGCACATCC

8821 GCGCCCGGCCAGCACAGGCCACGGCGAATCTTATTGAGATAGGTGAACTCGCGGCGCAGC

8881 AGGAAATCGACGAAGGAAATATTTCCCTGGTACAGATGCTGCAGATTGATCCCAGAGCAG

8941 AACACCCGTCGATCGCGGTACTTGGGGTGTTCCATCGGACCGCCACGCACGACCCCGACC

9001 ATCACCTCATCGTCGAGCAGGGCGAGATCGACCGCCACTTCCATCGCCTCGACCAGTGCA

9061 TTGTCCTCGGCATTCAGGCCAGTCTGATTGGCGACGGTCAACCAGGCCGCGCCATCGTGA

9121 CGCTGCACATGCACCGGCCCGATCTGCACCTGCGCGTCATTACGGAACTGCGGCAGCAAC

9181 GCCAACGCGCGCGCGGTCGGACGCAACATGCTGTCGATCAGATGGTGGCCGCAGTCGGGC

9241 AGGCGCAACACGCCCCACAGGACGATGGCCTGTTCGATCTCCCAGCCCTCTTTTTCGGCC

9301 TGGATGCAAGCGTGTTCGCGCGCCCATTGCGCCTGACTCGGCAACAAGCCGGGCACCGCA

9361 TCGGCAGCGGCACGCAAGACCTCGTCCAGGCGCAGCGACAACCCATGCGCCTGAGTGAGC

9421 GCCGCGTACAACCAGCCACCATGTTGGCGCATGAACTCCATCCGCACGGCCCTGCAACGC

9481 GCATGTACCGCGGCGGCGCGCTGCGCCTGCCCTTGGTCGCGTTGCGCAGGGGCCGGCAAG

9541 GCAAGCAGTTGCTGCTCGCCCCAGGCCGCCACTGCGCGCACTTGCGCCAGTTGCTCGGCC

9601 AGTGGGGTCATGCCGACGCCATCGGCATGCAGGTGCCGTGCGGATGTTCGGCGGTGGCCT

9661 GTGCCGCCAGCCGCCGCTGGCGGCGACGCAGTTCCATGTCGCACGCGGCCAGGTGTTGAC

9721 CCACGGCCTGCTCGTAGGAATGGGCCGCTCCTTCGAGCAGCAACAAGCGCCGGCCGGGCA

9781 GCGCCTGTAGGTCCACCTCCGCCAACGACGCCAGCCATGCGCCAATCGCGCCCTGAGCAT

9841 CCTCGCTGACTTGATCGACCACGCCCAACTGCGCCATCCGCGCGCAACCCAATTGCGTTT

9901 CAAACAGGAACACCGTGCGCGCAGCGTCGATGCCCATCCGCGTGCTCATGCGCCGCATCG

9961 CCATGCTCGGCCACGCGGCTTCGCCGCGGCTGTGCATCCGGATACTGGCACCAGGCACCG

10021 CGATGCGTCGGTCCACCGCCAGCAGCAGATCCAACATCCCCGCGGTGCAGGCATTGTCGA

10081 TGCAGGCCAAGGACACTCCCTCCAGCCGCTCGAAGCGACGCATGGCCTTTTCCCAGCGAC

10141 CGATCACATCCAGATTCACTGGCCCCGGCCACGATGCCTCAACCGGCAGCGTGGCCGCAC

10201 CACGCAGGTGCAGGAGCAACACGGCAGGTGTGCTCGCATCCTCCACCTCATCGCAGGCGC

10261 GACCGAGCCGTTGAGTCAATTCGTCGCTCAGCACACTGGTGGCGTCAATCTCCAGATGGA

10321 CGAGATGCAAGACGGGACTAGAAGAGTCGCTCATGAAAAATCCGCAAGATCGATAGAACG

10381 GGGGCCAGGGACAACTTACCAGCGCAACAAGGCCGTCTCGATCGACGATCCCGGCCCCAT

10441 CGTCATCATCACGCCCCAATCGCCGGGGCGGATCTGCCCCTCATCGAGCAGTCGCGCGTA

10501 AGAGAACAGGAACGAACCGCTGGACATATTGCCGTGCTCGCGCAACACCTCGATGGTATG

10561 GCGCATGTCGTGACTGGTCAGCATCAAGTTGGCGCAGATCGCATCGATGACCTTGCGCCC

10621 GCCCGAATGCACCAACCAGTGGGCGATATCGCGGCGACGCAGGCCAGTCCCCTCCAGCAA

10681 ACGCTGCACCACGGTGGGCGCATTGGCCCCCACCACGTAAGGCACGTCTTTATGCAGACG

10741 GAAATGGAACTTGCCATGGGTTTCATCCCAGGCGAAGCGCATGGCATCCAGCGCTTCGGG

10801 AATCACCTGGCTGGCGAATTTGCACACGCGCGGCCCGGCATGAACATCGCCACGCTCATC

10861 GCCATCGGCGATCACCGCCAGAGCCGCCGCGCCATCGCCGAACAGGCTGTTGACGACCGC

10921 CGTTTCGATCCCGTCATCGTCGACATACGCCGCCGAACACACTTCGATGCACAACAGGAC

10981 CGCGAGTTTCCCCGCATTGGCATGCGTCCAGTTGACCGCCGCATTGAAGCCGTTGAGGCC

11041 GGCGTTGCAGCCCATCCCGACCACATCCAGGCGCACGCAGTCCTGACGCATTCCCAAGCG

11101 CTGGATCAGCAACGAACTGAAGCCAGGGGTGAGCAAGCCGGTGGTGGTCACGCAACACAG

11161 ATAGCGCACGTCGTCCAGCGAGGCGCCAATGGACTGCAGACAGCGGGTCAAGGCGCGCTC

11221 GCCGATTTCCAGGCCGATATCGCGGTGTTTGTCGAGCAGGCGCGCCTGCGTTTCGCGCGG

11281 CGCGTTGCCTGCTTCGTCGCTCTCCGGCAACGCCAGATGACGCGAGTCGATCCCATTGTT

11341 TAAAAACACCTGACGCCGACGCCGATCTTCGATACCGAAGCGCCTCAGCACATCCGATTG

11401 CGAATAACGGCGCGCCGGGGTGGCGGTACCAACACCGCGAATGCGCGACGCGATGAATGT

11461 CGGCGTGCTCGTCGATGCAACATGTGCATTGAAGTTCATGCTTATCCGAATTCCAGCGTT

11521 GTCCAAGGTGGATCGCCAAGCTGTTGGAGCGACCGAGGTGCACGACTATGGTGGGTGAGA

11581 CCCCGGCAGTGCACTTGCTCGTGTGCGCCGATGAGTTGCCTATCAGCGTCATCGTGCAGC

11641 GCGCACATGGTGTACCGCACATTGGCCCGAGGAACAGCATATTTCCTGCAGTCTAGCCAC

11701 GCCATGTGACGGAACGACGATCCACAGGCCAGGCCACCCAAGACAAACCTTCGTCACTGC

11761 CAGTCAAGCGCATGCAAGGGCGAACGCCTACAAATCCACTCTACGCAACCCTCAGTCGAG

11821 TCAGACACGTGCAACGGCCGATGTCGATGATCTTGCTTCACTGCGACAAACACCCACTGC

11881 GCTCTTGGTCGTGCTCCAACAAAACGGCCTTCCAGTCGCCGTTTCGCTTGCTGGAGGGTC

11941 GTGCGCATCATGGATAACTCCGCCAAGAGCCTGCCCGCCTCGTTCGTACAAAAGCGGATG

12001 TGGTTGCTCGCCAGGCTGGACCCACAGGCGTCCGTCACTTACCACATCGCCAATGGCGTT

12061 CGCCTGACCGGCGCGTTGGACATCCACGCCCTGCAAGCGGCGCTGGACCGCATCGTCGCG

12121 CGACACGAAGTGCTGCGTACCAGCCTGATCGAGGTGAATGGACAGATTCGTCAGCGAATC

12181 CACCCGAGCATGCGCTTCACGCTGGTCCATGAGGATGCGAGGGCTTGCGCGACAGATCCC

12241 GCCGCGCTCGCGCGCATCGCGCAACAGGAGGCGGTGCGCCGGTTCGACTTCACACTGGGC

12301 GCCCCGGTGCGCGGACGTCTGCTGCGTCTTGCCGCACAAGAGCACATCCTGTTGTTGACC

12361 TTCCATCACATCGCCTGCGATGGCTGGTCAATCGGCGTCTTGTTGCGCGAACTGGAAAGC

12421 CTGTATGGCGCCTTTGTCCGACAGCAAGCGGACACCTTGCCGCCCTTGCCGATCCAGTAC

12481 GCGGACTACGCGATCTGGCAGAGCGAGCAATTGCAAGGCGATACGCTCGAGGCACAACTG

12541 CGCTTCTGGACCACGCAACTGGCGGATGCACCGACGCTGCTGCCACTGCCGACCGACCGC

12601 GCCCGCCCCCCCCTTCAGGATTACCGCGGCGCGAGCGTAGACACCCTGCTCCCACCGGCA

12661 TTGGGACAACAGCTCAATGCCCTGGCACGGCGGCATGGCTGCTCGTTATTCGCCACGGTG

12721 CTGGCCGGATGGGCGCTGCTGCTGACGCGCATCAGTGCGAGCAACGATGTGGTGATCGGC

12781 ACGCCGACCGCCGGACGCACGCATCCGGACCTGGAAGCGTTGATCGGCTGCTTCATCAAC

12841 ACGCTGGCGCTGCGCTTCACCCTGCGCGGCACACCGACGGTGGCGCAATGGCTGGCACAC

12901 GTCCGCGAGGTGGTCCTGAATGCGCAGGATCACCAGGAATTGCCGTTCGAGCGCGTCGTC

12961 GAAGCGCTGCAACCCAGCCGTAGCCTCAGCCATACACCGATCTTCCAGACACTTTTCAGT

13021 CTGGACGGTTTCAGTCGCCAACAGTCGCTGCAACTGCCCGGATTGCACCTGGAACCGTTG

13081 CCGGACACGGCGGACATGTGCGCGTTCGACCTGATCCTGACGATGCAGGAATCCGAGACG

13141 GGGCTGCGGGCACGGCTCAAATACCCGGTTGCGCTGTTCGACCGCGACACCATGGAACGG

13201 CGTCTGGCGCAGTTCGTCACGCTATTGCAGGCCATGGTCGCCGACGACACGCAATCCGTC

13261 GCGCAACTGCCGCTGTTGCCGCCGCAAGAGCGCACCCAACTCCAGCACATGAGCCAAAGC

13321 GCGCCCGCAGTGGACCTGCCGCAGGCAAGCCTGCACGCATTGTTCGCACAGCAGGCCGCG

13381 CGCACGCCCGAGGCCCCGGCCATCGTCAGCGCGACCGGGACGCTGTCCTACGCCGCCGTG

13441 CTCGCCCAGGCGCACGCCGTGGCCCAGCAACTCATCGCCATCGGCGTGCGCCCCGGCGAT

13501 CGCGTGGCGGTGGTGCTGCCGCGCGCTGCGCACTTGATCGTCGCCGAACTGGGCGTGCTC

13561 CACTGTGGTGCCGCCTATGTGCCGCTGGATCCGGCGCATCCAAGCGAACGCCTGGCCGAA

13621 CTCGTCACGCACTGCGCCGCCTGCGCGGTGCTGAGCGAACGCGCGATCGCCCTGCCGCCA

13681 TTGCAACAACCACGCCTGGATCTGGATCACGCCGCCTATGTGCCGCTGGATCCGGCGCAT

13741 CCAAGCGAACGCCTGGCCGAACTCCTCGCGCACTGCGCCGCCTGCGCGGTGCTGAGCGAA

13801 CGCGCGATCGCCCTGCCGCCATTGCAACAACCACGCCTGGATCTGGATCAAACGCTGTCC

13861 TACGCCGCCGTGCTCGCCCAGTCGCACGCAGTGGCCCAGCAACTCATCGCCATCGGCGTG

13921 CGCCCCGGCGATCGCGTGGCGGTGGTGCTGCCGCGCGCTGCGCACTTGATCGTCGCCGAA

13981 CTGGGCGTGCTCCACTGTGG

LOCUS Contig G111 4400 bp DNA linear BCT 30-JAN-2012

DEFINITION Xanthomonas spp. strain XaS3 genomic region encoding NRPS.

SOURCE Xanthomonas spp. strain XaS3

ORGANISM Xanthomonas spp. strain XaS3

Bacteria; Proteobacteria; Gammaproteobacteria; Xanthomonadales;

Xanthomonadaceae; Xanthomonas.

REFERENCE Contig G111 (bases 1 to 4400)

AUTHORS Royer et al. Submitted to BMC Genomics.

TITLE Genome mining indicates that the genus Xanthomonas is a promising

reservoir for new bioactive non-ribosomally synthesized peptides

JOURNAL Submitted to BMC Genomics.

REFERENCE 2 (bases 1 to 4400)

FEATURES Location/Qualifiers

source 1..4400

/organism='Xanthomonas spp.'

/mol_type='other DNA'

/strain='XaS3'

/isolation_source='sugarcane leaf water droplets'

/host='Sugarcane'

/db_xref='taxon: GPE 39'

/country='Guadeloupe'

/collection_date='2003'

/collected_by='Rosiane BOISNE-NOC'

/note='[cultured bacterial source]'

gene 63..752

/gene ='ortholog of XALc_1554'

CDS 63..752

/gene ='ortholog of XALc_1554'

/codon_start=1

/translation='MHAKKILLVEDDADSASILEAYLRRDGFDVAVAEDGQKAVALHAQWKPDLVLLDIMLPILGGTEVLSTIRRSSDTPVIMVTAMGDEPEKLGALRYGADDYVVKPYSPKEVVARVYAVLRRTGSARGKEEELLRHDRLSVDTAAVRATVRDGQGRESPLELTPTEFNLLATLMKTPFKAFTRSELLEICLPDSDALERVVDAHVHNLRKKLEQEGIHGLLITVRAIGYRFQ'

gene 815..1936

/gene ='ortholog of XALc_1553'

CDS 815..1936

/gene ='ortholog of XALc_1553'

/codon_start=1

/translation='MSALAVLTIVVIALGMVLRFAIWDFSTLHRLPPQVRQEMLLLREDPHSNAQRLWQLFETYYDVVDFLPGLASRDWWLLAQMVLASIPVIVICGLIASRPLSRQFSNVAEAARRIRDGDFAARASVVPGAPDELAGLAMDFNSMTAQLQQYEREVRDSSAMLAHELRTPLNAAMGRIQGMLDDVFPSNPEQLSMVQRQLEQINRLVGDLHLLSMARANQLLLEPQCFPLGALVRERIAWVAQPLQEAGMEVQLCIPEQLSMHADRDRLGQVLSVLIDNALRYAVAGKQLSIQGQSENGDVLICIADRGPGVAPEVLSRMLDRFWRADDSRARHSGGSGLGLSIAAAICQAHGGTLEFANRDGGGLCARVRLPQVS'

gene 2844..4400>

/gene ='NRPS'

CDS 2844..4400>

/gene ='NRPS'

/note= 'incomplete NRPS, no stop codon'

/codon_start=1

/translation='MDEGLNICMGANEDAWFPLSENQRALWFLYKLHPDLQGTYNISFFARILGEIDLNLLKRAIVELASRHAMLRTRFRELNSVPEQMSERFVEAQLRLVDVSELDPLSLDELVIKDSFRPFDVTVAPLFRVDLYQVGVREGVLLVTFNHLVVDGWSFWRLIEELGNILDEMVGVAPLKPCHSYGHEPELSYSDYVKWQRDWLNGEDGDRQFTYWQKMLEDECPSLNLPARAPSALLPPASRGCMSFNLDAGLSVKLSEMAARNHASLYVVLLTTYFIFLRRLTGQDDFHVGSPMPARRGRKWSEVVGMFFNQIVLRAGFNSDLTVRELLEMVRSRVRRAMGNQDFPFSELVARLNPPREKNRSPFFQTMFLFQDARGSSDVLNILAGGEDLAPVSWGGLHLTHFCHPPISGAGALDLILEAVKSGNKIFFLLNFDSSLFEHSTLERWMEHWRHLLAAMVAEGAEDQLVDRLPLLDEAERHQLLTQWNATAVDYPRDACVHELFEMQVARAPSAIAVVQGER'

ORIGIN

1 TCGGCGGGCATGTTCAAGCCGCTTTTTAGGAATTTTCAAGATTCCATGAAGGGGTCCATT

61 TCATGCATGCAAAGAAGATCTTGCTGGTCGAAGACGATGCGGACAGCGCCAGCATCCTCG

121 AGGCCTACCTGCGCCGGGATGGTTTCGACGTTGCGGTGGCCGAGGATGGCCAGAAGGCGG

181 TGGCGCTGCATGCGCAGTGGAAGCCGGATTTGGTCCTGCTGGACATCATGTTGCCCATAC

241 TCGGCGGTACCGAGGTGCTGTCCACGATCCGCCGCAGCAGCGATACGCCGGTGATCATGG

301 TCACTGCGATGGGCGATGAGCCGGAAAAGCTCGGCGCGCTGCGTTACGGCGCCGACGACT

361 ATGTGGTCAAGCCATACAGCCCCAAAGAAGTCGTGGCAAGGGTCTACGCCGTGCTGCGCC

421 GCACCGGATCGGCGCGAGGCAAAGAAGAAGAGCTGTTGCGCCACGACCGGCTCAGCGTCG

481 ATACGGCTGCGGTGCGGGCCACGGTGCGTGATGGTCAGGGCCGCGAATCGCCGCTGGAAC

541 TGACGCCGACCGAGTTCAATCTCCTGGCGACGCTGATGAAGACCCCGTTCAAAGCGTTCA

601 CGCGCAGTGAATTGCTGGAAATCTGCCTGCCAGACAGCGATGCCCTGGAGCGTGTGGTGG

661 ACGCGCACGTGCATAACCTACGCAAGAAATTGGAGCAGGAAGGGATTCATGGCCTGCTGA

721 TAACCGTGCGCGCAATCGGCTATCGCTTCCAATGATGTGGCCATTTTGCCGTCGCACCGC

781 CCACGCGCCGTTGTGGCAATGGGTAGGGCTGCGCATGAGTGCTTTGGCGGTGTTGACCAT

841 CGTGGTGATCGCGTTGGGGATGGTGCTGCGCTTCGCGATCTGGGATTTCTCCACCCTACA

901 TCGGTTGCCACCGCAGGTGCGGCAGGAAATGCTGCTATTGCGCGAGGACCCGCACAGTAA

961 CGCGCAACGCCTATGGCAACTGTTCGAAACCTACTACGACGTGGTCGATTTCCTGCCCGG

1021 ACTGGCCAGTCGCGACTGGTGGTTATTGGCGCAGATGGTGCTGGCTTCGATCCCGGTGAT

1081 CGTGATCTGCGGGCTGATCGCATCGCGCCCATTGTCGCGACAGTTCTCCAACGTAGCCGA

1141 AGCCGCACGACGGATCCGCGATGGCGATTTCGCCGCGCGCGCTAGCGTAGTGCCGGGTGC

1201 GCCCGATGAACTGGCCGGCTTGGCGATGGATTTCAACAGCATGACCGCGCAACTGCAGCA

1261 ATACGAACGCGAAGTGCGCGACTCAAGCGCAATGCTGGCGCATGAGTTACGCACTCCGCT

1321 CAACGCTGCAATGGGGCGGATACAGGGCATGCTCGATGACGTATTCCCCAGCAATCCGGA

1381 ACAGTTATCCATGGTGCAGCGCCAGTTAGAGCAGATCAATCGCCTGGTCGGCGATCTGCA

1441 CTTGCTGTCGATGGCCAGAGCAAACCAGTTGTTACTGGAGCCGCAGTGCTTCCCGCTCGG

1501 CGCACTGGTGCGCGAACGCATCGCCTGGGTGGCACAGCCGCTGCAGGAAGCCGGGATGGA

1561 GGTGCAACTGTGTATTCCCGAGCAGTTGAGCATGCATGCCGATCGCGACCGCCTCGGTCA

1621 GGTTCTGTCAGTGCTGATCGACAACGCGCTGCGCTATGCCGTCGCCGGCAAGCAGTTGTC

1681 GATCCAGGGACAGTCCGAGAATGGCGATGTGCTGATCTGTATCGCTGACCGTGGTCCCGG

1741 GGTCGCGCCGGAGGTCCTGTCGCGGATGCTGGATCGCTTCTGGCGTGCCGACGATTCGCG

1801 TGCTCGCCATTCTGGCGGTAGTGGCCTGGGTTTATCCATTGCCGCAGCGATCTGCCAAGC

1861 ACATGGTGGCACCCTCGAATTCGCCAACCGCGATGGTGGTGGGTTGTGCGCACGGGTGCG

1921 TCTGCCACAGGTGTCATAAGCAGACGCTGCAAGTATTGGCGCGTACCACGCCGCTGTGGA

1981 CAGTCCGACACAAACACAGGACCGACGCAATGATCGCCCATGCACCTGCACCATGTGCCG

2041 CACGGCCTCTCGCTTGCGCGCCGGCCCTAGAGTTTTTTGCGATCAAATCCTTCATCGCCA

2101 CGTTGTCCAGGGCCAGCTCGGCGTACATCCGCTTGAGCTTGGCGTTCTCGGCCTTGATCT

2161 CCTTGACCCGCCGTAGCTCGGAAGCCTCCAAGCCGCCGTACTTGCTCTTCCACTGGTAAT

2221 ACGTTGCCGTGCTGATGCCGGCCTGGCGGCAGAGTCTTTGACCGGTAGCCCGGCGTCAGC

2281 CTGCTTGCTGGCTTGGGAAAGATGCCAGAAAGATCTACTGATGCGGTGTCTGCCAGATAG

2341 GGGAGCTTACGCTGCTCATTGACGCTCCCGAATTCGCCATTTTCCATGGCCTTGAGATGT

2401 CTAGGGAACTCTGGGCGTATCAGTCGCTATTGCTGTAACAATTAATTTGTACAATAAGAT

2461 TTATTTTTAAGGCAATGTGAAAATTAAGATTAATATAATTTAACATTATGAGGCATGTAT

2521 TTTTTAGCTTCTCGCTCATTGTCGGGCTGGCAACGAGGCTGCGACAGGTAATTCATTCAT

2581 GGACTAAAAATTCAGCATTGAAATATCGAGAGATTGAAATTAATTTTTCATTGGATCCTG

2641 CGGCTGCGCAATCCATGAAAAATAAATTAATTATTATTTTATAATTTAATTTAATTTAAT

2701 TTAATTTAATTTAATCATACTTTAATTTGGTGATGATAGAGCTTTCCATTCGCTAAATAA

2761 TTAAAATTTAGTTTAATCATGGGTTTTCGGTTGAAAACTAATTTTTCGAATTCGAAATAG

2821 AAGTAATCCAAGAGGTTTTTGCGATGGATGAAGGTTTAAATATTTGCATGGGCGCGAATG

2881 AGGATGCATGGTTTCCTTTGTCGGAAAATCAGCGCGCATTATGGTTTTTATATAAACTTC

2941 ATCCGGATTTGCAGGGAACTTACAATATAAGTTTTTTTGCGCGAATTCTTGGTGAGATCG

3001 ATCTTAACCTGTTGAAACGGGCGATTGTAGAGCTGGCAAGTCGTCATGCAATGCTGCGCA

3061 CTCGTTTTCGCGAGTTAAACTCAGTCCCTGAACAAATGAGCGAGCGTTTTGTAGAGGCGC

3121 AGCTCCGATTGGTCGACGTTTCTGAGTTGGATCCATTGTCTCTTGATGAGCTAGTTATAA

3181 AGGATAGTTTTCGGCCTTTTGATGTCACAGTCGCCCCGCTATTTAGAGTTGATCTTTACC

3241 AAGTTGGCGTGCGAGAAGGTGTTCTATTGGTGACATTCAATCATCTTGTAGTGGATGGTT

3301 GGTCATTTTGGCGTTTGATCGAAGAGCTAGGAAATATTTTGGATGAAATGGTTGGGGTGG

3361 CCCCATTGAAGCCATGTCATTCCTATGGTCACGAACCCGAACTGAGTTACTCGGATTATG

3421 TAAAATGGCAGCGTGACTGGTTGAATGGGGAAGACGGCGATAGGCAATTCACCTATTGGC

3481 AGAAAATGCTTGAGGATGAGTGTCCATCCTTGAATCTGCCAGCGCGTGCGCCGTCTGCGC

3541 TATTGCCTCCTGCATCCAGAGGTTGCATGTCATTTAATCTGGATGCAGGGTTGTCCGTTA

3601 AATTGAGCGAGATGGCAGCGCGCAATCATGCCTCGCTCTATGTGGTTCTTCTAACCACTT

3661 ATTTTATTTTTCTTCGCCGGCTTACGGGACAGGATGATTTTCATGTTGGCTCTCCGATGC

3721 CGGCTAGGAGAGGGCGGAAATGGTCTGAAGTTGTCGGGATGTTCTTTAATCAGATCGTCT

3781 TGCGCGCTGGTTTTAATTCTGACCTGACGGTCAGGGAATTGCTGGAGATGGTGCGTAGTC

3841 GTGTCAGACGCGCAATGGGAAATCAAGACTTTCCATTTTCCGAGCTCGTGGCGAGACTCA

3901 ATCCTCCTCGCGAGAAGAATCGTTCGCCATTTTTTCAGACCATGTTTTTGTTTCAGGATG

3961 CACGTGGCTCTTCTGATGTGTTGAACATTTTGGCTGGTGGTGAGGATCTTGCACCTGTAT

4021 CTTGGGGAGGCTTGCATCTTACTCATTTTTGTCATCCTCCGATTTCCGGTGCTGGAGCGC

4081 TTGACCTTATCCTGGAAGCAGTCAAATCCGGAAATAAGATATTTTTTCTTTTAAATTTCG

4141 ACTCCTCGCTTTTCGAGCATTCGACCCTGGAACGCTGGATGGAGCATTGGCGTCATTTGT

4201 TAGCTGCGATGGTGGCCGAGGGCGCCGAAGATCAGCTAGTGGATCGTCTGCCGTTGCTAG

4261 ACGAGGCCGAGCGTCATCAGCTATTGACGCAATGGAATGCAACAGCGGTGGATTATCCAC

4321 GCGATGCGTGTGTACATGAATTGTTCGAGATGCAGGTAGCACGTGCGCCTTCGGCAATCG

4381 CAGTTGTACAGGGTGAGCGA

LOCUS Contig G129 13594 bp DNA linear BCT 30-JAN-2012

DEFINITION Xanthomonas spp. strain XaS3 genomic region encoding NRPS.

SOURCE Xanthomonas spp. strain XaS3

ORGANISM Xanthomonas spp. strain XaS3

Bacteria; Proteobacteria; Gammaproteobacteria; Xanthomonadales;

Xanthomonadaceae; Xanthomonas.

REFERENCE Contig G129 (bases 1 to 13594)

AUTHORS Royer et al. Submitted to BMC Genomics.

TITLE Genome mining indicates that the genus Xanthomonas is a promising

reservoir for new bioactive non-ribosomally synthesized peptides

JOURNAL Submitted to BMC Genomics.

REFERENCE 2 (bases 1 to 13594)

FEATURES Location/Qualifiers

source 1.. 13594

/organism='Xanthomonas spp.'

/mol_type='other DNA'

/strain='XaS3'

/isolation_source='sugarcane leaf water droplets'

/host='Sugarcane'

/db_xref='taxon: GPE 39'

/country='Guadeloupe'

/collection_date='2003'

/collected_by='Rosiane BOISNE-NOC'

/note='[cultured bacterial source]'

gene <2..4825

/gene ='NRPS'

CDS <2..4825

/gene ='NRPS'

/note='incomplete NRPS, no start codon'

/codon_start=1

/translation='PFAEHPGQRMYRTGDLARWRSDGTLEFLGRNDAQVKIRGFRVEPAEVEAALRDCPGVREALVLTDTPRQGEHRLVAYVVGNASTPDTLRTRLSARLPAYMVPAAYVLLDALPLTPNGKLDRRALPTPEHDTHADEAYVAPQGLLEQTLATLWCDLLGVARVGRHDNFFALGGHSLLAVTLIERLRRHGWQVDVRSLFGTPTLSSLAANLQTTRSVIVPPNRIGADCPRITPELLPLADLTQAEIDLVVDSVEGGAANVQDIYPLAPLQEGLLFHHLTDPEHDPYLQHTLLSCATRAHLDRVLDALQTLIARHDILRTAIVWERLRAPLQVVWRHAPLPVQTHAWSGSPDDVQQAIAAQPPICLQRAPLLRAHVVHDPDQDRWLLGLQHHHMVMDHTTLELAMEELQACLSGRQDRLPPPLPFRDFIAHAQLGVPAAEHQAFFTEMLADVQAPTAPFGLTTIADAAVHSEQAQLPLHASLGQRLRAHASGMGVSVATLFHLAYALIVARSSGRDTVVFGTVLFGRMWAGDGADRVLGMFLNTLPLRLDCDGTELTQAIHALQHRLARLLRHEHAPLALAQRCSGVRPPAPLFTAVLNYRHAGSARATETDNDSDHAWEGIETLPVQEHTHYAILLSVNDHGETGDFSLSVQALPRIGAQRVARMMLQALTSLDEALRLAPTTPLHALDVVPAEDVAGPPTVATTPVPTVMASCVHPLFEQQVARHPQAIAVQSGERTLSYAQLNAQANRLAHHLIALGVGPDMRVALCLERSVEMMVAVLAILKAGAAYVPLDPTYPPQRLAFMLQDSAPHYLLSHTALRARLPHGHVPILWLDADAAWAQQPEHNPDPAAQGLGPQHLAYVIYTSGSSGRPKGVMVTHASVVNLWQALRPIMQTDTASTPARVSLNAALSFDASVKMWVQLLSGACLVIVPQELRLDGTALLAWLRQMRLDVLDCTPAQLHLLLDHGMLDAADGMPKHVLIGGEAIPPTLWQRLRACAQIAFFNVYGPTECTVDVTVAAIQDSTPQPTLGRPLPNVPLRLLDHRGRPVPVGVVGELMVGGVQVSRGYLHRPGLSAERFIPDPFATQPGQRMYRTGDLARWCADGTLEFLGRGDQQIKLRGFRIELGDIVAALRSCDGVREAVVVDHEDGHGDTRLVAYWVGDASATDGEHLRAQLAAHLPDYMLPSTYLRLDSLPLNANGKLDREALPPPQSATPANAAYVPPATPIERRLAKLWASVLGPQRIGRNDHFFELGGHSMSVVRLIAAAKRSNFELTVQMVYAAPTLQAQAACLSGDTQALGPHIVAARRHGKRPPVFVVPTGVGDITYAFELAAHLDADIPVYALPWPEPLPATMEALAAQMVEWIQAVQPHGPYHLLGYSSGGLLAYAIAQHFGQQAQPVAFLGLLDCDVPVATANTDTLEVAIAQALLRQLEGLRRYRPYLDRRDIQAGLDALLERIGDSAYAEMAAACADDPTLAQLAMEEQTTVADLLQNCVISTCFNRLWPTFTAQPLSAECRLSLFQAIEPEPATDAYGWQHLLPATQIERIPVGGEHTTLIEAEHIGGLARRIEEALDTTVHPSPNKRDSKSATVAAPHAADTDRMTDPATP'

gene 4944..11351

/gene ='NRPS'

CDS 4944..11351

/gene ='NRPS'

/codon_start=1

/translation='MSATALSTPVDAVLHPLSSAQQGIWLGQLLAPEQPSYTIGCAMTFDGTLQRARWERAIAITIARHDALRTVLVEACDGSPLPAQRVLDTLPFSLPWHDYSANADGEQCVHEHIQHALTRSFAHYGQPLWDIQWLQATATRGYCLYLCHHICMDGVSLGMLSQQIVDCYNRLLRGETDPATPAPSFLRAVESDRNYLDSNRYRRDLDYWCSHLAARPEPRYPGAASVRGRQPTVQLRCTLEPHIFPALSALAERLNGSFTTLVTACLAICLTRLSHQDASIALGLTVHNRHNAAERDMLGMLSTQLPLYLSVQAQAEIGTTMRDVVGALRQAMRHARFPLQHAVHHLRQAGQQALRPFDISISVEDFSAFGDHPIEGGVRSMRALHAGYEDSALNVFVRRYNAQSPTLLEFNVNPDRLPLPLAESTMAALPQMLLALLQDPQMPVWRIPLLPPTQRQQLLGAFNTPAAHAPHDTQVHHAFERQAAATPEAIALICDDVALHYAALEAQANQLAHHLHALGVAPDDRVAIRLPRGVAMVVAVLATLKAGAAYVPLDPTYPAERQAYMLQDCWAQVLLTTGADAATSSWPDAPRVVYLDAPQPAWHTLPTTPPARVGSPLHAAYIIYTSGSTGQPKGVVMPHGALLNLLQWEAAQGLADGLQALRTLQYSPLGFDASFQEIFSTLGTGGTLVLIDDVQRRDTHALYQRLCAQRIERLYVPYIALQALADTVLADPALDTLDCHLQQVLTAGEQLRITPSIRAFFAKRAACRLHNYYGPTETHVASAHRLPADTTQWPLLPPIGNALPRTPLYVLDRHRQPLPIGATGELYLAGVQVARGYLHRPALTAERFVPDPFTEDAGQRMYKTGDLARWRADGSVEFLGRNDDQIKLRGFRIEPGEIEAALHACPGVREAAVLLRGDRPGDTRLVAYLTGQGVHVEQVRDHLLARLPEYMVPSAYVVLASIPTTPHGKLDRHALPLPDASALAAQAYVAPRGEAETCLAMLWCELLGVRQVGRHDDFFALGGHSLLAVQLIARLRACRGVDLALRTFFAHPRLADLARALAHAPPSTLPTIVPVPHPDPLPLSFAQQRLWVLAQFDARANLAYLMPGVVTLRGILNPIALRQALDRLLARHDALRTHFVSTDAGPAQVIAPPHIAMPLECIDLRHHADPQAAAQRYIEQETTTAFALEHGPLLRGRLLQLAEDEHLLLVTLHHLIADGWSIGVLLRELGALYRAFVHGQPDPLPALPIQYPDYTLWQRRWIDGPLLQQQRQFWCEHLRDAPALLTLPTDRPRPPEQDYAGDAVPVAIDAARTQALIALSQRHGTTLFMTLLAAWGVLLARLAGQSQVVIGTPIAQRTRRELEPLIGLFVNTQALHLDLRADPCVADLLAQVRATALAAQAHQDLPFEQVIEALNPVRSLAHAPLFQVMFTWQNTPQVALALPDLQCQLLPAATREAKYDLDLDLHLEHGCIVGSLRFATALFDAATLQRQWDTLGVLLDGMLADDRAHVCKLPLLSPAQRQHLHTFTGHDAAATDPRSLPQWFAQQAAATPHTIALVDGDNTLSYQQLDRQTNRLAHHLIALGARPEHCVALCLQRGIAQILAVLAVLKAGAAYLPLDPSQPRERIATVLADAQPVFVLVDDADHIARPATLSAPIVAIAAAQAAAIDAPEHAPVLPPLCAQHLAYVIYTSGSTGKPKGVMVSHHALTTRLHALIDLYRLGPQERVLQFASLAFDVSAEELFGTLCSGATLVLRDDTWLDTERFWPQCAQAGISVVNLPTRFWAQLCAQSLAIPACVRQVIIGGEALTPAMRQHWIQHTRIPLLDAYGPTEAVMAATTQAVAADTPSGIGRPLAATRAYVLDGAAQPLPIGACGELSLGGVAVARGYLGRPDLTAERFVPDPFATQPGARMYRSGDLACWRADGTLEYLGRNDQQLKLRGFRIEPGEIEAALRSCHGVEDALVLAHDGAADGPRLVAYVMPAHNDLPAVRTQLRERLPDYMQPAAYVLLDSLPLTPGGKVDHRALPEPHDDAFGHPAYEAPDGPLEQALAQLWRELLGIERVGRHDSFLDLGGHSLLAVRLAAAIRRTLHCDLPIQQLFAQPTLQRMANLVLGTRLAQLQRDTATSLLSKVQVER'

gene 11432..13594>

/gene ='NRPS'

CDS 11432..13594>

/gene ='NRPS'

/note='incomplete NRPS, no stop codon'

/codon_start=1

/translation='MLTETAPAAEETIQPRDDDAPLPLSFAQQRLWFLAQFDNRAAQAYTLAGGVDLHGVLDVPALQQALDRIVARHEVLRTCFVASDDGATQVIAPADAGFALTCIDLRHTADAEVAAHAYAEQAARTPFDLSRGPLIRGCLLQLAEQQQRLLIGMHHSISDGWSIGILLRELGALYAAFAQGQPDPLPPLPIQYADYSLWQRRWLDGPLLQRQLAFWRAHLHGAPALLELPTDHPRPALQDYRGDSVEIALDGDLTAALRALSQRHGTTVFMTVLAGWAVLLSRLSGQDQVVIGAPVANRTRSELEGLIGFFVNAQALRIDLRGAPSVTDLLTQVRATALAAQDHQDVPFEQVIEALNPERSLSAQPVFQVVLTWQNVPDAELVLPDMRLQPIPAQGGDAKFDLEFSLHEQHERIVGSLGYATALFERSTIERHLAQFVTLLQGMVADDHARVAQLPLLPADERTQLQRFTVTETAPLAPATCIHRLFEAQVQRTPDAIALREGQRLLRYAELDARANRLAQSLRRSGVGVENRVALYLPRGIEQVVAVLATLKAGAAYVPLDPELPSERLAFLLEDSRPRAVLTCTDLQDRLPASRAMLRVSVLTLDDSTDTHSDDPGAPDVPGLCPDNLAYIIYTSGSTGKPKGTLLTHAGAAHYLQWAIATYRPQPSAVVSSSLSFDATLTSLLAPLLCGAQVELLPEHDTLDALRQRLCDPTPLGLVKL'

ORIGIN

1 TCCGTTCGCCGAACACCCCGGCCAGCGCATGTACCGCACCGGCGACCTGGCGCGCTGGCG

61 CAGCGACGGCACGCTGGAATTCCTCGGCCGCAACGACGCGCAGGTCAAGATCCGCGGCTT

121 CCGCGTGGAGCCGGCCGAGGTCGAAGCGGCGCTGCGCGACTGTCCCGGCGTGCGCGAAGC

181 GCTGGTACTGACCGACACACCACGCCAGGGCGAACATCGCCTGGTGGCCTATGTCGTCGG

241 CAACGCGAGCACTCCCGACACCCTGCGCACACGGCTGAGTGCGCGACTGCCGGCCTACAT

301 GGTGCCGGCCGCCTATGTCCTGCTCGATGCACTGCCGCTCACACCCAACGGTAAGCTCGA

361 CCGCCGGGCGCTGCCGACGCCGGAGCACGACACCCACGCCGATGAGGCCTATGTCGCGCC

421 GCAAGGCCTGCTCGAACAGACCCTGGCGACGCTGTGGTGCGATCTGCTCGGTGTCGCCCG

481 GGTCGGTCGTCACGATAATTTCTTCGCGCTCGGCGGGCATTCGCTGCTGGCGGTCACCCT

541 GATCGAACGGCTGCGTCGGCATGGCTGGCAGGTGGACGTGCGCAGCCTGTTCGGCACACC

601 GACGCTGTCCAGCCTGGCCGCCAATCTGCAAACCACCCGCAGCGTGATCGTCCCGCCCAA

661 CCGCATCGGCGCGGACTGCCCTCGCATCACTCCGGAGCTGCTGCCATTGGCAGACCTCAC

721 CCAGGCCGAGATCGACCTGGTGGTGGACAGCGTGGAAGGCGGCGCGGCTAACGTGCAGGA

781 CATCTATCCGCTGGCGCCATTGCAGGAAGGCCTGCTGTTCCATCACCTGACCGATCCGGA

841 GCACGATCCATACCTGCAACACACGCTGTTGTCCTGTGCCACGCGTGCACACCTGGACCG

901 TGTGCTGGACGCGCTGCAAACGCTGATCGCCCGCCACGACATCCTGCGCACCGCCATCGT

961 CTGGGAACGCCTGCGCGCGCCGCTGCAGGTGGTCTGGCGCCACGCACCATTGCCGGTGCA

1021 AACACACGCATGGAGTGGCTCGCCCGATGACGTGCAACAGGCCATCGCCGCACAGCCCCC

1081 CATCTGCCTGCAGCGGGCACCGTTGTTGCGCGCGCACGTGGTGCACGACCCGGACCAGGA

1141 TCGCTGGCTGCTGGGTCTGCAACACCACCACATGGTGATGGACCACACCACACTGGAACT

1201 GGCGATGGAAGAACTGCAGGCTTGCCTAAGCGGCCGCCAGGACCGCTTGCCGCCACCGCT

1261 GCCGTTCCGCGACTTCATCGCGCATGCGCAGTTGGGCGTGCCGGCAGCGGAACACCAAGC

1321 GTTCTTCACCGAGATGCTGGCCGATGTCCAGGCTCCCACCGCGCCGTTCGGGCTGACCAC

1381 CATCGCCGATGCCGCGGTGCACAGCGAACAAGCGCAGTTGCCGTTGCATGCGTCGCTCGG

1441 CCAGCGCCTGCGCGCGCATGCCAGCGGCATGGGCGTCAGTGTCGCGACCCTGTTCCATCT

1501 GGCCTATGCGTTGATCGTAGCGCGCAGCAGCGGGCGCGATACGGTGGTGTTCGGTACAGT

1561 GCTGTTCGGGCGGATGTGGGCCGGCGACGGCGCCGACCGGGTGCTCGGCATGTTCCTCAA

1621 CACCTTGCCACTGCGGCTGGATTGCGACGGCACCGAGCTGACCCAGGCAATCCACGCCCT

1681 GCAACACCGCCTTGCCCGCCTGTTGCGACACGAGCACGCGCCCTTGGCCCTGGCGCAACG

1741 CTGCAGCGGCGTGCGCCCTCCCGCGCCCTTGTTCACCGCCGTACTGAATTACCGCCATGC

1801 CGGCAGTGCGCGCGCGACCGAGACCGACAACGACAGCGACCACGCCTGGGAAGGCATCGA

1861 GACGCTGCCGGTACAGGAGCACACCCACTACGCGATCTTGCTGTCGGTCAACGACCACGG

1921 CGAAACCGGCGACTTTTCGCTGAGTGTGCAGGCACTGCCGAGGATCGGCGCCCAGCGCGT

1981 GGCCCGGATGATGCTGCAGGCGTTGACGTCGCTGGACGAAGCCTTACGCCTGGCACCGAC

2041 GACGCCACTGCATGCGCTGGACGTGGTACCAGCCGAAGACGTCGCCGGCCCGCCCACTGT

2101 CGCAACCACACCGGTGCCGACAGTGATGGCGTCCTGCGTGCATCCGCTATTCGAACAGCA

2161 GGTGGCACGCCATCCGCAGGCCATCGCGGTGCAATCGGGCGAGCGCACACTTTCCTACGC

2221 CCAACTCAATGCACAGGCCAATCGTCTTGCCCATCACCTGATCGCCCTCGGGGTGGGTCC

2281 GGACATGCGGGTGGCGCTGTGTCTGGAGCGCAGTGTGGAGATGATGGTCGCAGTGTTGGC

2341 GATCCTCAAGGCCGGTGCCGCCTACGTACCGTTGGACCCGACCTATCCACCGCAACGCCT

2401 GGCCTTCATGCTCCAGGACAGCGCACCGCATTACCTGCTCAGCCACACCGCACTGCGCGC

2461 GCGGCTGCCGCACGGCCATGTCCCGATCCTGTGGCTGGATGCGGACGCCGCCTGGGCGCA

2521 ACAGCCCGAACACAATCCCGATCCCGCCGCGCAGGGTCTCGGCCCGCAGCACCTGGCCTA

2581 TGTGATCTACACCTCCGGTTCCAGCGGACGTCCCAAGGGCGTAATGGTCACGCACGCGTC

2641 CGTGGTCAATCTCTGGCAGGCGCTGCGTCCGATCATGCAGACGGATACGGCGTCCACGCC

2701 AGCGCGTGTCTCACTCAATGCGGCGTTGTCGTTCGACGCCTCGGTCAAGATGTGGGTGCA

2761 ACTGCTCTCCGGCGCCTGCCTGGTGATCGTGCCGCAGGAACTTCGCCTGGACGGCACGGC

2821 GCTGCTGGCCTGGCTGCGGCAGATGCGTCTGGATGTGCTCGATTGCACACCGGCGCAACT

2881 GCACCTGCTGCTCGATCACGGCATGCTCGACGCAGCGGACGGGATGCCCAAACACGTGCT

2941 GATCGGTGGCGAAGCGATCCCACCGACGCTGTGGCAACGCCTGCGTGCGTGCGCGCAGAT

3001 CGCGTTTTTCAATGTGTACGGCCCGACCGAATGCACGGTGGATGTCACCGTCGCGGCGAT

3061 CCAGGACAGCACCCCGCAGCCCACGCTGGGCCGACCACTGCCCAACGTGCCCCTGCGCCT

3121 GCTCGACCACCGAGGCCGGCCGGTCCCGGTTGGCGTCGTCGGCGAACTCATGGTCGGTGG

3181 CGTGCAGGTGTCGCGTGGCTATCTGCACCGTCCGGGCTTGAGCGCCGAGCGCTTCATTCC

3241 CGACCCCTTCGCGACGCAGCCCGGCCAGCGCATGTACCGCACCGGCGACCTGGCACGCTG

3301 GTGCGCCGATGGCACGCTCGAATTCCTCGGCCGTGGCGATCAGCAAATCAAACTGCGCGG

3361 TTTCCGTATCGAACTGGGCGACATCGTCGCGGCACTACGCAGTTGCGATGGCGTGCGTGA

3421 AGCGGTCGTGGTCGACCACGAGGACGGCCACGGCGATACGCGCCTGGTCGCATATTGGGT

3481 CGGCGACGCGAGCGCTACCGACGGCGAGCATCTGCGTGCGCAACTGGCCGCGCATCTGCC

3541 CGACTACATGCTCCCCAGTACCTATCTGCGACTGGACAGCTTACCGCTGAACGCCAACGG

3601 CAAACTCGACCGTGAAGCGTTACCGCCGCCGCAGAGCGCAACGCCGGCCAATGCCGCCTA

3661 CGTACCACCGGCCACACCCATCGAGCGTCGTCTGGCCAAACTCTGGGCCAGCGTGCTGGG

3721 GCCGCAGCGGATCGGCCGCAACGACCATTTCTTCGAATTGGGCGGACATTCGATGTCGGT

3781 GGTGCGCTTGATCGCCGCCGCCAAACGCAGCAACTTCGAGCTGACCGTGCAGATGGTGTA

3841 CGCCGCGCCGACGCTGCAAGCCCAAGCCGCGTGCCTGAGCGGGGACACGCAGGCGCTCGG

3901 GCCGCACATCGTGGCCGCGCGCCGCCACGGCAAGCGCCCGCCGGTGTTCGTGGTGCCGAC

3961 CGGCGTTGGCGACATCACTTATGCGTTCGAACTGGCCGCCCACCTCGATGCGGACATTCC

4021 GGTCTACGCCCTACCGTGGCCGGAGCCGCTCCCGGCCACGATGGAAGCACTCGCCGCGCA

4081 GATGGTCGAGTGGATCCAGGCGGTGCAGCCGCACGGTCCATATCACCTGCTCGGCTATTC

4141 CTCCGGTGGCCTGCTCGCTTACGCCATCGCCCAGCACTTCGGCCAGCAGGCCCAGCCTGT

4201 CGCCTTCCTGGGGCTGCTCGATTGCGACGTCCCGGTCGCAACGGCGAACACCGACACGCT

4261 CGAAGTGGCGATCGCCCAGGCATTACTGCGCCAACTGGAAGGATTGCGGCGCTATCGTCC

4321 CTATCTGGACCGCCGCGACATCCAAGCGGGACTGGACGCACTGCTGGAACGCATCGGCGA

4381 CAGTGCATACGCGGAGATGGCGGCGGCCTGCGCAGACGATCCGACACTGGCGCAGTTGGC

4441 GATGGAAGAACAAACCACGGTCGCGGACCTGCTGCAAAACTGCGTCATCAGCACCTGCTT

4501 CAATCGCTTGTGGCCGACGTTCACCGCACAGCCATTGTCGGCGGAGTGCAGGCTGTCTCT

4561 CTTCCAGGCCATCGAACCGGAGCCGGCCACCGATGCCTATGGCTGGCAACACCTGCTGCC

4621 GGCGACGCAGATCGAACGGATCCCGGTCGGCGGCGAACACACCACGCTGATCGAGGCCGA

4681 GCACATCGGCGGGCTCGCACGCCGCATCGAGGAGGCGCTCGACACCACCGTGCACCCATC

4741 GCCCAACAAGCGCGACAGCAAGAGCGCCACCGTCGCTGCACCCCACGCCGCGGACACCGA

4801 CCGCATGACCGACCCCGCCACGCCATGAACGCCATGATCGTCTTCGCCCGACCTTTCGCC

4861 ACAAGACCCATGCCATGCAGATGCGCCGGCGGCGGCGGCATGGCCGTGATGGCACGCGGC

4921 ACGCCCGATCACCGGGAGATCGCATGAGCGCTACCGCCTTGTCCACTCCCGTGGATGCGG

4981 TGCTGCATCCGCTCAGTTCGGCACAGCAAGGCATCTGGCTCGGGCAACTGCTCGCACCGG

5041 AACAGCCGAGCTACACCATCGGCTGTGCGATGACTTTCGATGGCACCCTGCAACGCGCGC

5101 GATGGGAACGCGCCATCGCCATCACGATCGCCCGCCACGACGCCCTGCGCACCGTGTTGG

5161 TCGAAGCATGCGACGGTTCGCCACTGCCTGCTCAACGTGTACTCGACACGCTGCCCTTCT

5221 CGCTTCCTTGGCACGACTACAGCGCCAACGCCGATGGCGAGCAGTGCGTGCATGAGCACA

5281 TCCAGCACGCCCTCACCCGTTCGTTCGCGCATTACGGCCAACCGCTCTGGGATATCCAAT

5341 GGCTGCAGGCCACCGCCACACGCGGGTATTGCCTGTACCTGTGCCATCACATCTGCATGG

5401 ATGGCGTCTCGTTAGGCATGCTGTCCCAGCAAATCGTCGACTGCTACAACCGCCTACTGC

5461 GTGGAGAAACCGATCCAGCAACACCAGCGCCCTCGTTTCTGCGCGCAGTGGAGAGCGACC

5521 GGAACTACCTGGACTCCAACCGTTACCGTCGCGATCTTGACTACTGGTGTTCCCATCTGG

5581 CCGCGCGACCAGAACCGCGTTATCCCGGCGCGGCATCCGTGCGCGGACGCCAGCCCACCG

5641 TTCAATTACGTTGCACGCTGGAACCGCATATTTTCCCGGCATTGAGCGCGCTGGCCGAAC

5701 GCCTGAATGGATCGTTCACCACGCTCGTCACCGCCTGCCTCGCGATCTGCCTGACCAGAC

5761 TGAGTCACCAGGATGCTTCTATCGCGCTTGGTCTAACCGTACACAATCGCCACAACGCCG

5821 CCGAGCGCGACATGCTCGGCATGCTGTCCACGCAATTGCCGCTCTACCTGAGCGTGCAGG

5881 CGCAGGCGGAGATCGGCACCACCATGCGCGATGTCGTCGGCGCATTACGCCAGGCCATGC

5941 GCCACGCCCGCTTCCCCTTGCAACACGCCGTGCATCACCTGCGCCAAGCCGGACAACAAG

6001 CGCTGCGACCATTCGATATCAGCATCTCGGTCGAGGACTTCAGCGCGTTCGGCGATCACC

6061 CGATCGAAGGAGGGGTGAGATCCATGCGCGCGTTGCATGCCGGTTACGAGGACAGCGCAC

6121 TGAATGTGTTCGTGCGGCGCTATAACGCGCAGTCTCCAACCCTGCTGGAATTCAACGTCA

6181 ACCCGGACCGATTGCCTCTGCCACTGGCCGAGAGCACCATGGCCGCATTACCGCAGATGC

6241 TGCTGGCGTTGCTGCAAGACCCGCAGATGCCGGTGTGGCGCATTCCCTTGTTGCCCCCAA

6301 CGCAACGCCAGCAATTGCTTGGCGCGTTCAACACGCCTGCGGCACACGCGCCACACGATA

6361 CGCAGGTCCACCATGCCTTCGAGCGTCAGGCCGCCGCCACCCCGGAGGCCATCGCGCTCA

6421 TCTGCGACGACGTCGCGCTCCACTATGCCGCACTGGAAGCTCAGGCCAACCAACTGGCGC

6481 ATCACCTGCACGCATTGGGCGTGGCCCCCGACGACCGGGTGGCGATCCGCCTGCCGCGCG

6541 GGGTCGCCATGGTGGTGGCTGTCCTCGCCACGCTCAAGGCCGGCGCAGCCTACGTCCCAC

6601 TGGACCCGACCTACCCGGCCGAGCGCCAGGCCTACATGCTGCAGGATTGCTGGGCACAGG

6661 TACTGCTGACCACGGGCGCAGATGCCGCGACGTCGAGCTGGCCGGATGCACCGCGGGTGG

6721 TGTACCTCGACGCCCCGCAACCGGCCTGGCATACCCTGCCGACCACGCCACCGGCACGAG

6781 TCGGATCGCCTCTGCACGCGGCCTACATCATCTACACCTCCGGTTCCACCGGCCAACCCA

6841 AAGGCGTGGTGATGCCGCACGGCGCACTGCTCAACCTGCTGCAATGGGAAGCCGCGCAGG

6901 GCCTCGCCGACGGCCTGCAAGCATTGCGCACACTGCAATATTCGCCGCTCGGCTTCGATG

6961 CCAGCTTCCAGGAAATCTTCAGCACCCTCGGCACCGGCGGCACCTTGGTGCTGATCGACG

7021 ATGTGCAACGCCGCGATACCCATGCGCTGTACCAACGCCTCTGCGCCCAGCGTATCGAGC

7081 GCCTATATGTGCCGTACATCGCGCTGCAAGCGCTGGCCGACACGGTGCTGGCCGATCCCG

7141 CACTGGACACGCTGGACTGCCACCTGCAACAGGTACTCACCGCCGGCGAACAACTGCGCA

7201 TCACCCCGAGCATCCGCGCCTTCTTCGCCAAGCGCGCAGCGTGCCGGTTGCATAACTACT

7261 ACGGCCCCACCGAAACCCACGTCGCCTCCGCCCATCGGCTGCCCGCCGACACGACGCAAT

7321 GGCCGTTGCTGCCGCCAATCGGCAACGCCCTGCCACGCACGCCGTTGTATGTACTGGATC

7381 GCCACCGCCAACCATTACCCATCGGCGCCACCGGCGAGCTCTATCTGGCCGGCGTGCAGG

7441 TCGCACGCGGCTATCTGCACCGGCCAGCACTGACCGCCGAGCGCTTCGTGCCCGATCCAT

7501 TCACCGAAGATGCCGGCCAACGCATGTACAAGACCGGCGATCTCGCCCGCTGGCGCGCCG

7561 ATGGCAGCGTGGAATTCCTCGGCCGCAACGACGATCAGATCAAACTGCGTGGCTTTCGGA

7621 TCGAACCGGGCGAGATCGAAGCGGCACTGCATGCCTGTCCCGGCGTGCGCGAGGCGGCGG

7681 TCCTGCTACGCGGCGATCGCCCCGGCGACACACGTTTGGTGGCCTATCTGACCGGCCAGG

7741 GAGTCCACGTGGAACAGGTCCGCGATCACCTGCTCGCACGCCTGCCCGAGTACATGGTCC

7801 CGAGCGCGTATGTCGTGCTCGCCAGCATTCCGACCACGCCCCACGGCAAACTCGATCGCC

7861 ACGCATTGCCGCTGCCGGATGCCAGCGCCCTGGCCGCGCAGGCCTATGTCGCCCCGCGAG

7921 GCGAAGCGGAAACGTGCTTGGCCATGCTGTGGTGCGAGCTGCTCGGCGTCCGACAGGTCG

7981 GTCGTCACGACGACTTCTTCGCCCTGGGCGGTCACTCGCTGCTGGCCGTGCAATTGATCG

8041 CCCGGCTGCGTGCCTGCCGCGGCGTCGACCTCGCCTTGCGCACGTTCTTCGCCCACCCGC

8101 GCCTGGCCGATCTGGCCCGCGCGCTCGCACACGCGCCGCCCAGCACGCTGCCGACCATCG

8161 TACCGGTGCCGCATCCTGACCCACTCCCGCTATCCTTCGCCCAACAGCGCCTGTGGGTGC

8221 TGGCGCAGTTCGATGCACGTGCCAATCTGGCCTATCTGATGCCCGGCGTCGTGACCTTGC

8281 GCGGCATCTTGAACCCGATCGCGCTGCGGCAGGCACTGGACCGCCTGCTCGCCCGCCACG

8341 ATGCACTGCGCACCCACTTCGTCAGCACCGATGCCGGCCCCGCCCAGGTGATCGCACCGC

8401 CGCACATCGCCATGCCACTGGAGTGCATCGACCTGCGTCACCACGCCGATCCGCAGGCTG

8461 CCGCCCAGCGTTATATCGAGCAGGAAACCACCACCGCCTTCGCGCTGGAACATGGCCCGC

8521 TCTTGCGCGGACGCCTGCTGCAACTGGCCGAAGACGAACACCTGTTGCTGGTGACCCTGC

8581 ATCATCTGATCGCCGATGGTTGGTCGATCGGCGTATTGCTGCGCGAACTCGGCGCGCTCT

8641 ACCGCGCCTTCGTCCACGGCCAGCCCGATCCATTGCCAGCGCTGCCGATCCAATATCCCG

8701 ACTACACCCTGTGGCAGCGTCGCTGGATCGATGGCCCGCTGCTGCAACAACAGCGCCAGT

8761 TCTGGTGCGAGCATCTGCGCGATGCCCCCGCCCTGCTGACCCTGCCCACCGACCGCCCGC

8821 GTCCGCCCGAACAAGACTACGCAGGCGACGCCGTGCCGGTCGCCATCGATGCCGCACGCA

8881 CCCAGGCACTGATCGCACTGAGCCAGCGCCACGGCACCACCCTCTTCATGACCCTGCTCG

8941 CGGCCTGGGGCGTGCTGCTGGCGCGCCTGGCCGGCCAGAGCCAGGTGGTCATCGGCACCC

9001 CAATCGCCCAGCGCACGCGGCGCGAACTGGAGCCGTTGATCGGCTTGTTCGTCAACACCC

9061 AGGCCTTGCACCTGGACCTGCGCGCCGACCCCTGCGTGGCCGACCTCCTGGCCCAGGTGC

9121 GCGCCACCGCCCTGGCCGCGCAAGCACATCAGGATCTGCCCTTCGAACAGGTGATCGAAG

9181 CGCTCAACCCCGTGCGCAGCCTGGCCCATGCACCGCTGTTCCAGGTCATGTTCACCTGGC

9241 AGAACACCCCGCAGGTCGCCCTCGCCCTGCCCGATCTACAGTGCCAGCTACTGCCCGCAG

9301 CGACCCGCGAGGCCAAGTACGACCTGGACCTGGACCTGCACCTGGAACACGGCTGCATCG

9361 TCGGTAGCCTGCGCTTTGCCACCGCCTTGTTCGACGCCGCCACACTACAACGGCAATGGG

9421 ACACCCTCGGCGTGCTGCTCGACGGCATGCTCGCCGACGACCGCGCGCACGTGTGCAAAC

9481 TGCCGTTGCTCTCGCCCGCTCAACGCCAGCATCTGCACACCTTCACCGGGCATGACGCAG

9541 CCGCCACCGATCCGCGCAGTCTGCCGCAGTGGTTCGCCCAACAGGCCGCCGCGACACCGC

9601 ACACCATCGCCCTGGTCGATGGCGATAACACACTCAGTTACCAACAACTCGACCGCCAGA

9661 CCAACCGCCTGGCCCACCACCTCATCGCGCTGGGTGCACGGCCCGAGCACTGCGTGGCAC

9721 TGTGCCTGCAACGCGGTATCGCACAGATCCTCGCCGTGCTCGCCGTGCTCAAGGCAGGCG

9781 CCGCCTACCTACCGCTGGATCCCAGCCAGCCGCGCGAACGCATCGCCACCGTGTTGGCCG

9841 ATGCGCAGCCGGTGTTTGTGCTTGTCGACGACGCCGATCACATCGCCAGGCCAGCGACAC

9901 TCAGCGCTCCCATTGTCGCCATCGCCGCCGCACAGGCCGCCGCCATCGACGCGCCCGAGC

9961 ACGCCCCGGTGCTGCCGCCGCTATGCGCGCAACACTTGGCGTATGTCATCTACACCTCCG

10021 GCTCGACCGGCAAACCCAAGGGCGTGATGGTCTCCCACCACGCACTGACCACGCGCCTGC

10081 ACGCACTGATCGACCTGTACCGGCTAGGACCACAGGAGCGCGTGCTGCAATTTGCTTCAC

10141 TGGCTTTCGATGTCTCCGCCGAAGAGCTCTTCGGCACTTTGTGCAGCGGCGCCACCCTGG

10201 TCCTGCGCGACGACACCTGGCTGGACACCGAGCGGTTCTGGCCACAGTGCGCGCAAGCCG

10261 GCATCAGCGTGGTCAACCTGCCCACCCGCTTCTGGGCACAGCTCTGCGCACAGTCGCTGG

10321 CGATTCCCGCCTGCGTGCGTCAGGTCATCATCGGCGGCGAAGCGCTGACGCCCGCCATGC

10381 GCCAACACTGGATCCAGCACACACGCATCCCCCTGCTGGATGCCTATGGCCCGACCGAGG

10441 CCGTCATGGCGGCCACCACTCAGGCCGTCGCCGCCGACACCCCGAGCGGCATTGGACGCC

10501 CACTCGCGGCCACCCGAGCCTATGTACTGGATGGCGCCGCCCAGCCACTGCCGATCGGCG

10561 CCTGCGGCGAACTGTCTCTGGGTGGAGTGGCAGTCGCGCGCGGCTACCTGGGCCGCCCCG

10621 ACCTGACCGCCGAACGCTTCGTCCCCGATCCGTTCGCCACACAGCCCGGCGCACGCATGT

10681 ATCGCAGTGGCGATCTGGCCTGCTGGCGCGCCGATGGCACCCTGGAATACCTCGGCCGCA

10741 ACGACCAACAACTGAAGCTGCGCGGCTTCCGCATCGAACCTGGCGAGATCGAAGCGGCCC

10801 TGCGCAGTTGCCACGGCGTTGAGGATGCGTTGGTCTTGGCGCACGACGGCGCCGCCGACG

10861 GGCCACGGCTGGTGGCGTATGTGATGCCTGCGCACAACGACCTCCCGGCCGTGCGTACGC

10921 AACTGCGCGAGCGTCTGCCCGACTACATGCAACCGGCGGCCTATGTGCTGCTGGACAGCC

10981 TGCCGCTGACACCTGGCGGCAAAGTGGACCACCGCGCCTTGCCGGAACCACACGACGACG

11041 CGTTCGGACACCCCGCGTACGAAGCCCCCGATGGTCCGCTCGAACAAGCACTGGCGCAGC

11101 TGTGGCGCGAGCTGCTCGGCATCGAGCGCGTCGGTCGCCACGATAGCTTCCTCGACCTGG

11161 GCGGGCATTCCCTGCTCGCCGTGCGGCTAGCCGCCGCGATTCGTCGCACCCTGCACTGCG

11221 ATCTGCCGATCCAGCAACTGTTCGCACAACCGACCCTGCAGCGAATGGCCAACCTGGTGC

11281 TCGGCACGCGGCTTGCGCAATTGCAACGCGACACCGCGACCTCCCTCCTCTCCAAAGTTC

11341 AAGTGGAACGCTAACCACCATGTCCTCTTCCAGCATGTTTCCGCAGGATATCTCGACCCT

11401 GACCCGCGAGGAGACACATCGTCTCTGGGCCTTGTTGACCGAGACAGCACCTGCTGCGGA

11461 AGAGACGATCCAACCCCGCGACGACGACGCGCCACTGCCCCTGTCCTTCGCGCAACAACG

11521 GCTATGGTTCCTGGCGCAATTCGACAACCGCGCCGCGCAGGCCTACACCCTGGCCGGTGG

11581 CGTGGACCTGCACGGCGTGCTGGATGTGCCCGCACTGCAACAGGCACTGGATCGCATCGT

11641 CGCTCGCCACGAGGTGCTGCGCACGTGCTTCGTCGCCAGCGACGATGGCGCCACCCAGGT

11701 GATCGCGCCGGCCGATGCCGGCTTCGCGCTGACCTGCATCGATTTACGCCACACCGCCGA

11761 TGCCGAGGTTGCCGCGCACGCCTATGCCGAACAGGCAGCCCGCACGCCCTTCGATCTGAG

11821 CCGTGGTCCATTGATCCGTGGTTGCCTGTTACAACTGGCCGAGCAGCAACAGCGCCTGCT

11881 GATCGGCATGCACCACAGCATCTCCGATGGCTGGTCCATCGGCATCCTGTTGCGCGAGCT

11941 TGGCGCGCTGTATGCCGCCTTCGCGCAAGGCCAGCCTGATCCGTTGCCGCCGCTGCCGAT

12001 CCAATACGCCGACTACAGCCTGTGGCAACGCCGCTGGCTCGATGGGCCGCTGCTGCAACG

12061 GCAACTCGCGTTCTGGCGTGCACATCTGCATGGCGCGCCGGCGCTGCTGGAACTGCCCAC

12121 CGATCACCCGCGTCCGGCATTGCAGGATTATCGCGGCGATAGTGTCGAGATCGCCCTCGA

12181 TGGCGACCTCACCGCCGCGTTGCGTGCGCTGAGCCAACGCCACGGCACCACCGTGTTCAT

12241 GACCGTGCTGGCCGGCTGGGCCGTGCTGTTGTCGCGACTGTCCGGTCAAGATCAGGTCGT

12301 GATCGGCGCGCCTGTCGCCAACCGCACGCGCAGCGAACTCGAAGGATTGATCGGCTTCTT

12361 CGTCAACGCCCAGGCGCTGCGCATCGACCTGCGCGGCGCGCCCAGCGTCACCGATCTGTT

12421 GACGCAGGTGCGTGCCACCGCACTGGCAGCGCAGGATCATCAGGACGTGCCGTTCGAACA

12481 GGTGATCGAGGCGCTCAATCCCGAGCGGAGTCTGTCCGCGCAACCGGTGTTCCAGGTGGT

12541 GCTCACCTGGCAGAACGTCCCCGATGCCGAGTTGGTGCTGCCCGATATGCGCTTGCAGCC

12601 CATCCCCGCACAGGGCGGCGATGCTAAGTTTGATCTGGAGTTCTCGCTGCACGAACAGCA

12661 CGAGCGCATCGTCGGCAGCCTGGGGTATGCCACGGCGCTGTTCGAGCGCAGCACCATCGA

12721 ACGCCATCTGGCCCAGTTCGTGACCCTGTTGCAAGGCATGGTCGCCGACGACCACGCCCG

12781 TGTCGCGCAACTGCCGCTATTGCCTGCCGACGAACGCACGCAATTGCAACGCTTCACCGT

12841 CACCGAGACCGCGCCGCTGGCTCCGGCCACCTGCATCCATCGCTTGTTCGAAGCGCAGGT

12901 GCAACGCACGCCCGATGCCATCGCACTGCGAGAAGGACAACGCCTGCTGCGCTACGCCGA

12961 ACTGGATGCCCGCGCCAATCGGCTTGCTCAGAGTCTGCGTCGCTCCGGTGTCGGTGTGGA

13021 AAATCGTGTCGCGTTGTACCTGCCGCGCGGCATCGAACAAGTGGTCGCGGTGCTCGCCAC

13081 GCTCAAAGCGGGCGCGGCGTATGTGCCGCTGGATCCGGAACTGCCCAGCGAACGTCTGGC

13141 CTTCCTGCTGGAAGACAGCCGTCCACGCGCAGTGCTGACCTGCACCGATCTGCAGGATCG

13201 TCTGCCGGCCAGCCGCGCGATGCTGCGCGTGAGTGTGCTGACACTCGACGACAGCACGGA

13261 CACGCACAGCGATGATCCCGGCGCACCGGATGTTCCCGGCCTGTGCCCGGACAATCTGGC

13321 CTACATCATCTACACCTCCGGTTCCACCGGCAAACCCAAGGGCACCTTGCTCACGCATGC

13381 CGGTGCGGCGCATTACCTGCAATGGGCCATCGCCACCTATCGCCCGCAGCCCAGCGCGGT

13441 GGTGTCCTCGTCGTTGTCCTTCGACGCGACCTTGACGAGTCTGCTTGCTCCGCTGCTGTG

13501 CGGTGCGCAGGTCGAACTGCTACCCGAACACGACACGCTCGACGCCCTGCGGCAACGTCT

13561 GTGTGATCCGACCCCGCTGGGCCTGGTCAAACTC

LOCUS Contig G130 12805 bp DNA linear BCT 30-JAN-2012

DEFINITION Xanthomonas spp. strain XaS3 genomic region encoding NRPS.

SOURCE Xanthomonas spp. strain XaS3

ORGANISM Xanthomonas spp. strain XaS3

Bacteria; Proteobacteria; Gammaproteobacteria; Xanthomonadales;

Xanthomonadaceae; Xanthomonas.

REFERENCE Contig G130 (bases 1 to 12805)

AUTHORS Royer et al. Submitted to BMC Genomics.

TITLE Genome mining indicates that the genus Xanthomonas is a promising

reservoir for new bioactive non-ribosomally synthesized peptides

JOURNAL Submitted to BMC Genomics.

REFERENCE 2 (bases 1 to 12805)

FEATURES Location/Qualifiers

source 1.. 12805

/organism='Xanthomonas spp.'

/mol_type='other DNA'

/strain='XaS3'

/isolation_source='sugarcane leaf water droplets'

/host='Sugarcane'

/db_xref='taxon: GPE 39'

/country='Guadeloupe'

/collection_date='2003'

/collected_by='Rosiane BOISNE-NOC'

/note='[cultured bacterial source]'

gene <2..12805>

/gene ='NRPS'

CDS <2..12805>

/gene ='NRPS'

/note='incomplete NRPS, no start codon, no stop codon'

/codon_start=1

/translation='RLPEAMLPTAYVHLDTLPLTANGKLDRRALPAPEADALATQPYVAPQGERETLLAALWSELLGVEQVGRHDSFFALGGHSLLAISLIERLRQHGWQLQVRALFNAPALADLASTLTAASTLNIPPNRIAPDCTRITPELLPLVELSQTEIDAAVATVDGGTANVQDIYPLAPLQEGLLFHHLASPEGDAYLNISVLPFDSRTHLDAFLAALQAVIDRHDILRTGFAWQGLRTPVQVVWRHAPLPLQTHIIQAHDVLDALRERMDPSRFRLDVSRAPLIHAHLVEDPAHARWLLGLHSHHLMMDHTTLELLIEEVQAHLHGQQAQLPAPLPFRNFVAQARLGVSEAEHRAFFTQQLGDLDTPTAPFGLWDVRGTGADIEQTLQPLPDALSSALRQHARQLGVSPSSLFHLACALVLAQASGQDDVVFGTTLFGRMQGGHGADRVLGMFLNTLPIRLRRDGRSVTEAIRQTQQQLAQLLHHEHAPLALAQRCSSIAPPTPLFTALLNYRYVGGSAVQTPSETTQQPHDWHGLEMLAGLDRNNYPLTISIDDITATGGFAVEVKVDRHIGTERVIALVQQTMQALIQALEQAPDTALYALSLLPAAERAQLQRFTVTETAPLAPATCLHHLFEAQVQRTPDAIALREGTHTLRYAELDARANQLAQRLRRSGVGVENRVALYLPRCIEQVVALLATLKAGAAYVPLDPELPSERLAFLLEDSRPRAVLTCTDLQDRLPASRAMLRVSVLTLDDSTDTHSDDPGAPDVPGLCPDHLAYVIYTSGSTGQPKGTLLTHAGATHYLQWAIEHYRPQPSAVVSSSLSFDATLTSLLAPLLCGAQVELLPEHDTLDALRQRLCDPTPLGLVKLTPAHLEVLGQQLTDQQTPLSPAVMVIGGEALPPATLARWQALAPHTRLINEYGPTETVVGCAVHTTTADDAHASNGRVPIGKPIAHLRLYVLDAHGQPAPMGVAGELHIAGPQLARGYLGRADLTAERFIPDPFAEHPGQRMYRSGDVACWRADGTLEYLGRNDDQVKLRGFRIELGEIAAALRACAGVQDAAVLLREDTPGEPRLVAYVVTDHAEHADSLTLRDALSSRLPAVMVPAVYMHLEALPLTPNGKLDRRALPTPEQRDLGNALYVPPQGTLEQTLAQLWCDLLGVEQVGRHDSFFALGGHSLLGVKLIERLRLLGWQLEVRDLFNTPTLSGLSQTLTTTSMVSVPANPISAGCTRITPELLPLVTLTQAEIDGITSGLDGGVGNVQDIYPLAPLQRGLLYHHIAAPEDDIYLSSTVLAFDASDKRERFIAALEQVIARHDILRTAVFWQGLREPVQLVQRQAPLPVQHHACTGEDIATQLQHRLETQHRRIDLQQAPLLRAHLADAAPHGSWLLGLQYHHLVMDHTTLELVVEEVQAHLDGQQQHLPAPLPFRDFVAQACLGVSEEEHRAFFTQMLADVTEPTLPFGVSVVEGPLHEANLLLPDALAAMLREQARRLGVSTASLFHLTFALLLAQISGRDDVVFGTVLFGRLHASAGADRVLGLFLNTLPLRLRRDRTGVEQAVVQTQRLLAQLLHHEHAMLAQAQRCSGIQAPAPLFAALFNYRYRGGGNVLADDHARREAIWQGIETVHLRERTHYPLSLAINDDHDHGGFSLDVQTGQNLDPVALAQSMADLLQHLAQSLAEAPNRPLHALTRLRDAERTQVLEDFNASDATLSITGSVHQRFEQQARQTPDAVAIVHAQRTLRYAELDGQANRLAHHLRSLGATPGSTVAVAMARSLELIVAELAILKCGAAYVPLDIEHPAQRMRHILDACGATLLISHSNHDIAAGNAVRVDLDRLHLDGSHDAPSVPVWPDSPAYVMYTSGSTGMPKGVVIPHRAVLNFASQPGDARILAQDRVAFASNPAFDSSTLEVWSSLLNGATIVIVSPQVLRDPQALCAQIADMRISVLILVAGVLRAYAPMLSGKLPSLRVLMTGGDLADPHSHGLMLQAPGPDALLQTYGPTEATQFVTALALHHAPDPRQTVPIGRPLVNNRLFVLDRFGQPTAVGVEGALHIAGAQLALGYLGRPDLTAACFVPDPFAVQPGQRMYKTGDMARWRADGTLDFLGRRDEQVKIRGFRIELGEIVAALRACADVRDAVVVVDQDHAGHKRLVAYVVGAADLGQALPEQLATLLPDYMMPTAYVPLDALPLTANGKLDRRGLPAPDRQDFDTQAYQTPHPGREQCLAQLWSELLGVAQIGRQDHFFALGGHSLLAVQLIGRIRAQLGLDLSLSTLFAHPRLADLATAMDSAASAALAPILPVPRTGPLPLSFAQQRLWFLGRLDPHADLAYLMPMALRLRGTLQLHALEQALDRSVARHEILRSYVVLEDQTPFQHIAPPHIGFALERVDLRHAADPQQQIQQQIALETSTPFPAERPLIRGRLLRLADDDHVLLITLHHLVSDGWSMGVLVQELAALYRAYVEQRPDPLPPLSLQYADIAVWQRDWLSGEVLQRQRDFWIAHLHDAPTLLELPTDRPRPARQAYPGAALDVVLDATLTAALKAACQRHGTTLFMTLLAAWAVLLSRLSGQDRVVIGTPVANRHRSEFEPLIGLFANTQALCVDLRANPSVNALLAQVRRTALAAQEHQDLPFEQVIEALNPTRSLAHHPIFQAMFTWQNTPLDDIALPDLLVEGVDAALPTIKFDLDLSLREHDDRIVGSLGYACHLFERSTIERHLAQFLQVLRSLLDADTTRVAQLPLLPLAERECVLHAMHHALQIPPATPYVHQSFAAQAQRTPRHTALQCAADTLDYAALDARANQLAHHLIALGVVPEDRVAVCLPRGIDLIVALLATLKAGAAYLPLDPHYPPARLDAMLADAQPRVLLAHRDTALPLAQRESMHTVLLDAEIARWDCAPTHAPDVATLHPQHPAYVIYTSGSTGQPKGVVICHAALTQFLAAMQAQLPLSPEDRLLAVTTVCFDIAGLELFAPLVQGACVVIANDGAMQDPACWRQHLEQQAISVVQATPAFWQMLLDAGWQSHPGLRLLCGGDCLSQDLAQRLRAGGAPLWNLYGPTEATIWASLHPVLGDDAGSLVPLGRPLADTRMRLLDAHGHLVPLGVRGALFIAGPQLARGYLGRPDLTAERFLPDPFAEHPGQRMYKTGDLARWRADGVLEFLGRDDDQVKLRGFRIELGDIAAALRACTGVHEAVVLARQDIPGDTRLVAYVVGEHAMRAEHLRTQVAARLPDYMLPAAYVQLDALPLTANGKLDRKALPAPDADALAMQAYAAPEGELETQLAEIWRELLGVEQLGRHDNFFALGGHSLLATQLSARVRAVLGREIALATVFAHPQLAAFAQAVAQATASTVRPILPVSRSNPLPLSFAQQRLWFIDQLDAGAGSAYLMPTAMRLRGALNRDALHDALDRLVARHEALRTSVGFDGATPVQRIASPTIGFPLERIAAHDGVDPAALIQHHAAVEAHTPFDLSRGPLIRGRLVCLDVDDHVLCVTLHHLICDGWSMRQLIAELGTLYSAFARGLPDPLPALPIQYADYAVWQRSWIDEETLQRQRQFWIDHLHDAPALLDLPTDRPRPPVQDYRGHTLPFALDASLSAALKTLSLRHGSTLFMTLLAGWASVLARCANQARLVIGTPIANRPRHELEPLIGLFANTQALQIDLRDNPTVAALLAQVRETTLAAQAHQDLPFEQVIEALNPPRDLGHHPLFQTMLTWDAEHATDLAWPHVHTELIDQGSATIKFDLHLALQESDQGIVGSLGYATALFERSTIERHLAQFVTLLQGMVADDHARVAQLPLLPTDERAQLQTFNATASDLDGSGYLHRAIEAQAQRTPDAIALVDDGVELRYADLDARANQLAHHLIGLGVVPECVVAVCLPRGIDLLVALLAVLKAGGAYLPLDRDVPSARLHAMLADARPSVLLAHRDTAASLAQRDGMHTVLLDAEQTAWASVATHAAVVASLHPQHPAYVIYTSGSTGTPKGVVNTHAAIDNRLQWMQQALQLQPEQRVLQKTPVGFDVSVWELFWPLRVGACLVLAQPGGHKDPTYLHALIEEAAIDTVHFVPSMLRVFLDALPHGACASLRRIVCSGEALPADLAQAAHARLPQARLYNLYGPTEAAVDVSVWECRASDATQVPIGRPIANTQLHVCDAHAQPAPIGVAGELHIAGVQVARGYLGRPDLTAEHFVPDPFAAQPGARMYRSGDVARWRRDGALEYLGRNDDQVKRRGVRIELGEIAAALRTCAGVQDAAVLLREEMQGEPRLVAYVVGDADA'

ORIGIN

1 TCGCCTGCCCGAAGCGATGCTGCCGACGGCCTACGTCCACCTCGACACCTTGCCGCTGAC

61 CGCCAATGGCAAGCTGGATCGACGCGCGCTACCAGCGCCAGAAGCCGATGCGCTCGCGAC

121 CCAGCCTTACGTCGCCCCGCAAGGCGAACGCGAAACCCTGCTCGCCGCGCTATGGAGTGA

181 ACTCCTCGGCGTCGAACAGGTCGGCCGCCACGACAGTTTCTTCGCCCTCGGTGGGCACTC

241 GCTGCTGGCGATCAGTTTGATCGAACGCCTGCGCCAGCATGGCTGGCAGTTGCAGGTGCG

301 CGCCTTGTTCAACGCGCCCGCGCTGGCCGATCTGGCCAGCACCCTCACCGCCGCGTCCAC

361 GCTCAACATTCCCCCCAACCGCATCGCGCCCGATTGCACGCGCATCACACCGGAGTTGTT

421 GCCGCTGGTCGAACTCAGCCAGACCGAGATCGATGCCGCCGTGGCCACCGTCGACGGCGG

481 CACCGCCAACGTGCAGGACATCTATCCGCTGGCGCCACTGCAAGAGGGTCTGCTGTTCCA

541 TCACCTGGCCAGCCCCGAAGGCGATGCCTACCTCAACATCAGCGTGCTGCCCTTCGACAG

601 CCGCACGCACCTGGATGCCTTCCTCGCCGCGTTGCAAGCCGTCATCGACCGCCACGACAT

661 CCTGCGCACCGGCTTCGCCTGGCAGGGATTGCGCACACCGGTGCAAGTGGTCTGGCGGCA

721 TGCGCCGCTGCCGCTGCAGACGCACATCATCCAGGCTCACGATGTCTTGGATGCCTTGCG

781 CGAACGCATGGACCCCAGCCGCTTCCGTCTGGATGTCAGCCGGGCACCGCTGATCCACGC

841 CCACCTCGTCGAAGATCCCGCGCACGCACGCTGGCTGCTCGGCCTGCATAGCCATCACCT

901 GATGATGGATCACACAACGCTGGAGTTACTGATCGAGGAAGTGCAGGCGCATCTGCACGG

961 ACAACAGGCGCAGTTGCCGGCGCCGTTGCCGTTCCGCAACTTCGTCGCCCAGGCACGCCT

1021 GGGGGTGAGTGAAGCCGAACACCGTGCCTTCTTCACCCAGCAACTGGGCGATCTCGATAC

1081 GCCCACTGCGCCGTTCGGTTTGTGGGACGTACGCGGCACCGGCGCCGACATCGAACAGAC

1141 GTTGCAGCCGCTGCCCGACGCCCTGTCCAGCGCGCTGCGCCAGCATGCACGCCAACTCGG

1201 CGTCAGCCCGTCCAGCCTGTTCCATCTGGCCTGCGCACTGGTATTGGCGCAAGCCAGCGG

1261 CCAGGACGACGTGGTGTTCGGCACGACCTTGTTCGGCCGCATGCAAGGCGGTCACGGTGC

1321 CGACCGCGTCCTGGGCATGTTCCTCAACACGCTCCCGATCCGTCTGCGCCGCGATGGACG

1381 CAGCGTCACCGAAGCAATCCGCCAGACCCAGCAACAACTGGCGCAATTACTCCATCACGA

1441 ACACGCTCCATTGGCATTGGCGCAACGCTGTAGCAGCATCGCGCCGCCGACGCCGCTGTT

1501 CACCGCATTGCTCAACTATCGCTATGTCGGCGGCAGCGCGGTGCAGACACCCAGCGAGAC

1561 AACCCAACAGCCGCACGACTGGCATGGCCTGGAAATGCTGGCGGGACTGGATCGCAACAA

1621 CTATCCGCTGACGATCTCGATCGATGACATCACGGCAACCGGAGGTTTCGCGGTAGAGGT

1681 AAAGGTGGACCGGCACATCGGCACCGAGCGGGTCATCGCATTGGTGCAGCAAACCATGCA

1741 GGCATTGATCCAGGCGTTGGAGCAGGCGCCGGACACAGCCCTGTACGCACTGTCGCTGTT

1801 GCCTGCCGCCGAACGCGCGCAATTGCAGCGCTTCACTGTCACCGAGACCGCGCCGCTCGC

1861 CCCGGCGACCTGCCTCCATCACTTGTTCGAAGCGCAGGTACAACGCACGCCCGATGCCAT

1921 TGCGCTGCGCGAAGGCACACACACCCTGCGCTATGCCGAGCTGGATGCACGCGCCAACCA

1981 ACTCGCCCAGCGTCTGCGTCGCTCCGGCGTCGGTGTGGAGAATCGGGTCGCGTTGTACCT

2041 GCCGCGCTGCATCGAACAAGTGGTCGCGCTACTCGCCACGCTCAAAGCCGGCGCAGCGTA

2101 TGTGCCGCTGGATCCGGAACTGCCCAGCGAACGTCTGGCCTTTTTGCTGGAAGACAGCCG

2161 TCCACGCGCAGTGCTGACCTGCACCGATCTGCAGGATCGTCTGCCAGCCAGCCGCGCCAT

2221 GCTGCGCGTGAGTGTGCTGACACTCGACGACAGCACGGACACGCACAGCGATGATCCCGG

2281 CGCACCGGATGTCCCCGGTCTGTGCCCGGATCACCTGGCCTACGTGATCTACACCTCCGG

2341 CTCCACCGGCCAACCCAAGGGCACCTTGCTCACGCATGCCGGTGCGACGCATTACCTGCA

2401 ATGGGCCATCGAACACTATCGACCGCAGCCCAGCGCGGTGGTGTCCTCGTCGTTGTCCTT

2461 CGACGCGACCTTGACCAGCCTGCTCGCCCCGTTGCTGTGCGGTGCGCAGGTCGAATTGCT

2521 ACCCGAACACGATACCCTGGATGCCTTGCGGCAACGTCTGTGCGATCCGACCCCGTTGGG

2581 GTTGGTCAAGCTCACCCCGGCGCATCTGGAAGTGTTGGGTCAGCAATTGACCGATCAGCA

2641 AACACCGCTGAGCCCTGCGGTCATGGTGATCGGCGGCGAAGCACTGCCGCCCGCCACGCT

2701 GGCCCGTTGGCAAGCCCTCGCACCGCACACGCGTCTGATCAACGAATACGGCCCGACCGA

2761 GACCGTGGTCGGCTGCGCGGTCCACACCACCACCGCCGACGATGCACATGCCTCCAACGG

2821 GCGTGTGCCGATCGGCAAGCCAATCGCGCATCTGCGCCTGTACGTACTCGATGCCCACGG

2881 GCAACCCGCGCCGATGGGCGTGGCCGGCGAATTGCATATCGCCGGGCCGCAACTGGCGCG

2941 CGGATATCTCGGTCGTGCGGACCTAACCGCCGAACGCTTCATCCCCGATCCGTTCGCAGA

3001 GCACCCCGGCCAACGCATGTACCGCAGCGGCGATGTCGCCTGCTGGCGCGCCGATGGCAC

3061 GTTGGAGTATCTGGGCCGCAACGACGACCAGGTGAAACTGCGCGGGTTCCGCATCGAACT

3121 GGGCGAAATCGCCGCCGCGTTGCGTGCCTGCGCGGGCGTGCAGGATGCGGCGGTGTTGCT

3181 GCGTGAGGACACTCCGGGCGAGCCGCGATTGGTCGCCTATGTGGTCACCGACCACGCAGA

3241 ACATGCCGATTCGTTGACGCTGCGCGATGCACTGAGCAGCCGCCTGCCAGCAGTCATGGT

3301 GCCGGCCGTGTATATGCACCTGGAAGCGCTACCGCTGACCCCCAACGGCAAACTGGATCG

3361 CCGCGCCCTGCCGACGCCGGAGCAACGCGACCTGGGCAATGCGCTCTATGTCCCGCCGCA

3421 AGGCACGCTCGAACAGACCCTGGCACAGCTATGGTGCGACCTGCTCGGTGTCGAGCAGGT

3481 CGGTCGCCACGACAGTTTCTTCGCCCTCGGTGGTCACTCGCTGCTGGGAGTCAAATTGAT

3541 CGAGCGTCTACGCCTGCTCGGTTGGCAACTGGAAGTCCGCGACCTATTCAACACCCCGAC

3601 ACTGAGCGGCCTGAGCCAGACGCTCACGACCACGTCCATGGTCAGCGTCCCCGCCAATCC

3661 GATCAGCGCCGGCTGCACTCGCATCACCCCCGAGCTGCTGCCGCTGGTCACGCTCACCCA

3721 GGCGGAAATCGACGGCATCACCAGCGGACTGGATGGCGGTGTCGGCAACGTACAGGACAT

3781 CTACCCGCTCGCGCCGTTGCAACGTGGCCTGTTGTACCACCACATCGCCGCGCCCGAGGA

3841 CGACATCTACCTGAGCAGCACCGTGCTCGCCTTCGATGCCAGCGACAAGCGCGAGCGCTT

3901 CATCGCCGCGCTCGAACAGGTGATCGCCCGTCACGACATCCTGCGCACCGCTGTGTTCTG

3961 GCAAGGATTGCGCGAGCCCGTCCAGCTCGTCCAGCGGCAGGCACCGCTGCCCGTGCAGCA

4021 CCATGCCTGCACGGGCGAGGACATCGCCACGCAACTGCAACATCGCCTGGAAACCCAGCA

4081 CCGCCGCATCGACTTGCAGCAAGCGCCATTACTGCGCGCGCATCTGGCCGACGCTGCACC

4141 ACACGGAAGCTGGCTGCTCGGCCTGCAATATCATCACCTGGTGATGGACCACACCACGCT

4201 GGAACTGGTGGTCGAGGAAGTGCAAGCGCATCTGGACGGACAGCAACAGCACTTGCCCGC

4261 GCCATTGCCGTTCCGCGACTTCGTCGCCCAGGCCTGCCTGGGCGTGAGCGAAGAAGAGCA

4321 CCGTGCGTTCTTCACCCAGATGCTGGCCGATGTCACCGAGCCGACCCTGCCCTTCGGCGT

4381 GAGTGTGGTCGAAGGCCCGCTGCACGAAGCCAATCTGCTCCTGCCCGACGCACTGGCCGC

4441 CATGCTACGCGAACAGGCACGCCGCCTGGGTGTCAGCACGGCCAGTCTGTTCCACCTGAC

4501 GTTTGCGCTGTTACTGGCGCAGATCAGTGGCCGCGACGACGTGGTGTTCGGCACCGTGCT

4561 GTTCGGTCGCCTGCACGCCTCGGCCGGCGCCGACCGCGTGCTGGGCCTGTTCCTCAACAC

4621 CTTGCCGCTGCGCCTGCGGCGCGACCGCACCGGTGTCGAACAGGCCGTCGTGCAGACCCA

4681 GCGCCTGCTCGCGCAACTGCTGCATCACGAACACGCCATGCTCGCGCAAGCCCAGCGCTG

4741 CAGTGGCATCCAGGCACCGGCGCCGTTGTTCGCCGCCCTGTTCAATTATCGCTACCGTGG

4801 CGGTGGCAACGTCCTGGCCGACGACCACGCGCGCCGCGAGGCGATCTGGCAAGGCATCGA

4861 AACCGTGCACTTGCGCGAACGCACGCACTATCCGTTATCGCTGGCGATCAACGACGACCA

4921 CGATCACGGCGGATTTTCGCTGGATGTGCAAACCGGCCAGAACCTGGATCCGGTCGCGCT

4981 GGCGCAGAGCATGGCCGATTTGCTGCAGCACCTGGCCCAGTCACTGGCCGAAGCGCCAAA

5041 CCGCCCCCTGCATGCACTGACCCGACTGCGCGATGCCGAACGCACCCAGGTGCTGGAAGA

5101 TTTCAATGCCAGCGACGCCACGCTCTCGATCACCGGCAGCGTCCATCAGCGATTCGAACA

5161 GCAGGCACGGCAGACACCGGACGCCGTAGCGATCGTCCACGCACAGCGCACGCTGCGTTA

5221 CGCCGAGCTCGATGGCCAGGCCAACCGGCTGGCGCATCACCTGCGCAGCCTCGGCGCGAC

5281 GCCTGGCAGCACCGTTGCCGTGGCCATGGCGCGCTCGCTGGAGCTGATCGTCGCCGAGTT

5341 GGCGATCCTCAAATGCGGCGCAGCCTATGTTCCGCTGGACATCGAACATCCGGCGCAACG

5401 CATGCGCCACATCCTCGACGCGTGCGGCGCAACGCTGCTCATCAGCCATTCGAACCACGA

5461 TATCGCAGCGGGCAACGCCGTGCGCGTGGACCTGGATCGGCTGCACCTCGACGGCTCGCA

5521 CGACGCCCCCTCGGTGCCGGTCTGGCCCGACAGCCCGGCGTATGTCATGTACACCTCCGG

5581 CTCCACCGGCATGCCCAAGGGCGTGGTGATTCCACATCGCGCCGTGCTCAACTTCGCCAG

5641 CCAACCCGGTGATGCCAGAATCCTGGCTCAGGACCGGGTCGCCTTCGCTTCCAATCCCGC

5701 GTTCGACTCGTCCACTCTGGAAGTGTGGAGCAGCCTGCTCAATGGCGCGACGATCGTCAT

5761 CGTTTCGCCACAGGTCCTGCGCGATCCACAGGCGCTGTGCGCGCAGATCGCCGACATGCG

5821 GATTTCGGTGCTGATTCTGGTCGCCGGCGTGCTGCGCGCCTATGCGCCGATGTTGAGCGG

5881 CAAACTCCCCAGCCTGCGTGTCTTGATGACCGGCGGCGATCTGGCCGATCCGCACAGCCA

5941 TGGGCTGATGTTGCAGGCACCTGGCCCGGATGCACTGCTGCAGACCTACGGCCCGACCGA

6001 AGCCACGCAGTTCGTGACCGCGCTTGCCTTGCACCACGCGCCCGATCCACGCCAGACCGT

6061 GCCGATTGGCCGACCGCTGGTCAACAACCGCCTGTTCGTCCTCGACCGCTTCGGTCAGCC

6121 GACGGCGGTCGGCGTCGAAGGCGCCTTGCACATTGCCGGCGCACAACTCGCGCTGGGTTA

6181 TCTCGGCCGCCCCGATCTCACCGCCGCGTGTTTCGTCCCCGATCCTTTCGCCGTGCAACC

6241 CGGTCAACGGATGTACAAGACCGGCGACATGGCACGTTGGCGCGCGGACGGCACCCTGGA

6301 CTTCCTCGGCCGTCGCGACGAGCAAGTCAAGATCCGCGGTTTCCGCATCGAACTGGGCGA

6361 GATCGTCGCGGCGCTGCGCGCCTGTGCCGACGTGCGCGATGCGGTCGTGGTGGTCGATCA

6421 GGACCACGCCGGACATAAACGCCTGGTCGCGTATGTGGTCGGCGCTGCCGACCTCGGTCA

6481 AGCACTGCCCGAGCAACTGGCGACATTGCTACCGGACTACATGATGCCGACCGCCTACGT

6541 GCCGTTGGATGCTCTGCCGTTGACTGCCAATGGCAAGCTCGACCGGCGTGGGTTACCCGC

6601 ACCGGATCGGCAGGACTTCGACACGCAGGCGTACCAGACGCCGCACCCCGGACGCGAGCA

6661 GTGCCTGGCGCAGTTGTGGAGCGAGTTGCTCGGCGTCGCGCAGATCGGCCGCCAGGATCA

6721 CTTCTTCGCCCTCGGCGGACACTCGCTGCTGGCGGTGCAATTGATCGGAAGAATCCGCGC

6781 GCAACTGGGCCTGGATCTGTCCTTGAGCACGCTGTTCGCACATCCGCGCTTGGCCGATCT

6841 GGCCACGGCCATGGACAGTGCCGCCAGCGCCGCGCTTGCGCCGATCCTGCCCGTGCCACG

6901 CACCGGCCCACTGCCGCTGTCCTTTGCCCAGCAGCGCCTGTGGTTCCTGGGCCGTCTGGA

6961 TCCCCACGCCGATCTGGCCTATCTGATGCCGATGGCGCTGCGCTTGCGCGGTACGCTGCA

7021 ACTGCACGCGTTGGAACAGGCGCTGGATCGCAGCGTGGCCCGCCACGAGATCCTGCGCAG

7081 CTACGTGGTGCTGGAAGACCAAACGCCGTTCCAGCACATCGCTCCGCCGCACATCGGCTT

7141 CGCCCTGGAGCGCGTGGACCTGCGCCACGCAGCCGACCCGCAGCAGCAGATCCAGCAGCA

7201 GATCGCACTGGAAACCAGCACCCCCTTCCCCGCCGAACGGCCACTGATCCGCGGGCGCCT

7261 GCTGCGTCTGGCCGACGACGATCATGTTCTGCTCATCACCCTGCACCATCTGGTGTCCGA

7321 TGGCTGGTCGATGGGCGTCCTGGTCCAGGAACTGGCGGCACTGTATCGCGCATACGTCGA

7381 ACAACGCCCCGATCCGCTGCCGCCGCTCTCGCTGCAATACGCCGACATCGCCGTATGGCA

7441 GCGCGATTGGCTCAGTGGCGAGGTGCTGCAACGCCAGCGCGATTTCTGGATCGCGCATCT

7501 GCACGACGCCCCGACGCTGCTGGAACTGCCGACCGACCGGCCACGCCCGGCACGGCAGGC

7561 ATATCCCGGCGCGGCACTGGACGTGGTGCTGGATGCGACGCTCACCGCCGCCTTGAAAGC

7621 AGCGTGCCAACGCCACGGCACCACCTTGTTCATGACCTTGCTCGCGGCGTGGGCGGTACT

7681 GTTGTCGCGTCTGTCCGGCCAGGACCGCGTGGTCATCGGCACGCCGGTCGCCAACCGCCA

7741 CCGTAGCGAATTCGAACCACTGATCGGTCTGTTCGCCAACACCCAGGCGCTGTGCGTCGA

7801 TCTGCGCGCCAACCCGTCGGTGAATGCGCTGCTGGCACAAGTGCGCCGCACTGCGCTGGC

7861 CGCGCAGGAGCACCAGGATCTGCCTTTCGAGCAGGTGATCGAAGCGCTCAACCCGACCCG

7921 CAGCCTGGCCCATCATCCGATCTTCCAGGCGATGTTCACCTGGCAGAACACGCCACTGGA

7981 CGACATCGCGCTGCCAGACCTGCTCGTGGAAGGCGTGGATGCGGCATTACCGACGATCAA

8041 GTTCGATCTGGACCTGTCGCTGCGCGAACACGATGACCGCATCGTCGGCAGCCTCGGCTA

8101 TGCCTGTCACCTGTTCGAACGCAGCACCATCGAACGCCACCTGGCACAGTTCCTCCAGGT

8161 TCTGCGCAGCCTGCTCGACGCCGACACCACCCGCGTCGCCCAGCTACCGCTGTTGCCGCT

8221 GGCCGAGCGCGAATGCGTGTTGCACGCAATGCACCACGCCCTGCAGATACCGCCGGCAAC

8281 CCCATACGTGCACCAGTCGTTCGCAGCACAGGCGCAGCGCACGCCCAGGCACACCGCCCT

8341 GCAGTGCGCGGCGGACACCCTGGACTACGCCGCGCTCGACGCGCGCGCCAACCAGCTCGC

8401 CCACCACCTGATCGCACTGGGCGTGGTGCCGGAAGACCGCGTGGCCGTATGCCTGCCGCG

8461 CGGCATCGACCTGATCGTCGCCCTGCTGGCGACGCTCAAAGCCGGCGCGGCTTACCTGCC

8521 GCTGGATCCACACTATCCACCCGCGCGCCTGGACGCCATGCTCGCCGATGCACAGCCGCG

8581 CGTCCTGCTTGCCCATCGCGACACGGCTTTACCGCTGGCGCAGCGCGAGAGCATGCACAC

8641 GGTGCTGCTGGATGCCGAAATCGCGCGGTGGGACTGCGCCCCCACCCACGCCCCCGACGT

8701 TGCCACGTTGCATCCGCAGCATCCGGCCTACGTCATCTATACCTCCGGCTCCACCGGCCA

8761 ACCCAAGGGCGTGGTGATCTGCCACGCCGCGCTGACCCAGTTCCTCGCCGCGATGCAGGC

8821 GCAACTGCCGTTGTCGCCCGAAGACCGTCTGTTGGCGGTGACCACCGTCTGCTTCGACAT

8881 CGCCGGCCTGGAACTGTTCGCCCCGCTGGTGCAAGGCGCTTGCGTGGTTATCGCCAACGA

8941 TGGAGCAATGCAGGATCCGGCCTGCTGGCGACAGCACCTGGAGCAGCAGGCCATCTCGGT

9001 GGTGCAAGCCACCCCCGCGTTCTGGCAGATGCTGCTGGATGCCGGCTGGCAGAGCCATCC

9061 GGGACTGCGCCTGCTCTGCGGCGGCGACTGCCTGAGCCAGGATCTCGCCCAGCGCCTGCG

9121 CGCCGGCGGTGCGCCACTGTGGAATCTGTACGGACCGACCGAAGCCACCATCTGGGCCAG

9181 CCTGCATCCGGTGCTCGGCGACGACGCCGGCAGCCTCGTGCCGCTCGGCCGTCCCTTGGC

9241 CGACACCCGCATGCGCCTGCTCGACGCACACGGCCATCTGGTCCCACTCGGCGTGCGCGG

9301 CGCGCTGTTCATCGCCGGTCCGCAACTGGCACGCGGTTACCTGGGACGCCCGGATCTGAC

9361 CGCCGAACGCTTCCTCCCCGACCCGTTCGCCGAACATCCCGGACAACGCATGTACAAGAC

9421 CGGCGACCTGGCGCGCTGGCGCGCCGATGGCGTGCTGGAGTTCCTCGGCCGCGACGACGA

9481 CCAGGTCAAGCTGCGCGGCTTCCGCATCGAACTGGGCGACATCGCCGCTGCGCTGCGCGC

9541 CTGCACGGGCGTGCACGAAGCCGTGGTCCTCGCCCGCCAGGACATCCCCGGCGACACCCG

9601 TTTGGTCGCCTATGTGGTCGGCGAACACGCAATGCGCGCCGAACACCTGCGCACGCAGGT

9661 CGCCGCACGCCTGCCCGACTACATGCTCCCCGCCGCCTACGTGCAACTGGATGCACTGCC

9721 GCTCACCGCCAACGGCAAACTGGATCGCAAGGCCTTGCCGGCGCCGGACGCCGACGCACT

9781 GGCGATGCAAGCCTACGCAGCGCCGGAAGGTGAATTGGAAACGCAACTGGCCGAGATTTG

9841 GCGCGAATTGCTCGGCGTCGAACAGCTCGGACGCCACGACAACTTCTTCGCCCTGGGTGG

9901 ACACTCCTTGCTCGCCACCCAGTTGAGCGCACGCGTACGCGCCGTGCTGGGACGCGAGAT

9961 CGCGCTCGCGACCGTCTTCGCACACCCGCAACTGGCTGCATTCGCGCAAGCCGTCGCACA

10021 GGCGACAGCCAGCACCGTGCGTCCGATCCTACCGGTGTCCCGCAGCAATCCGCTGCCGCT

10081 GTCGTTCGCCCAGCAACGTCTGTGGTTTATCGACCAACTCGATGCCGGTGCCGGCAGCGC

10141 CTATCTGATGCCGACCGCGATGCGTCTGCGCGGCGCGCTCAATCGCGACGCCTTGCACGA

10201 TGCACTGGATCGCCTGGTCGCACGCCATGAAGCGCTACGCACATCCGTCGGTTTCGATGG

10261 CGCTACACCTGTACAACGCATCGCGTCGCCCACCATCGGCTTTCCCCTCGAACGCATCGC

10321 AGCACACGATGGCGTCGATCCGGCCGCACTGATCCAGCACCACGCGGCCGTGGAAGCGCA

10381 CACGCCCTTCGATCTGAGCCGTGGCCCGCTGATCCGTGGCCGCCTGGTCTGCCTGGACGT

10441 CGACGACCATGTGTTGTGCGTCACCCTACACCACCTCATCTGCGATGGCTGGTCGATGCG

10501 CCAACTGATCGCCGAACTGGGCACGCTGTACAGCGCATTCGCGCGCGGATTGCCCGACCC

10561 GCTGCCTGCGCTGCCGATCCAGTACGCCGACTATGCGGTGTGGCAGCGCAGCTGGATCGA

10621 TGAAGAAACGCTACAACGCCAACGCCAGTTCTGGATCGACCACCTGCATGACGCGCCCGC

10681 CTTGCTGGATCTGCCGACCGACCGACCACGCCCACCGGTACAGGATTACCGTGGCCACAC

10741 ACTGCCATTCGCACTGGATGCGTCGCTGAGCGCCGCGCTGAAGACACTGAGCCTGCGTCA

10801 CGGCAGCACCTTGTTCATGACCCTGCTGGCGGGCTGGGCGAGCGTGCTGGCGCGCTGTGC

10861 CAATCAAGCACGCCTCGTCATCGGCACCCCCATCGCCAATCGCCCGCGCCACGAACTGGA

10921 ACCGCTGATCGGTCTGTTCGCCAACACCCAGGCCTTGCAGATCGACCTGCGCGACAATCC

10981 AACCGTCGCCGCATTGCTGGCCCAGGTCCGCGAGACCACCCTGGCCGCGCAGGCACATCA

11041 AGACCTCCCCTTCGAGCAGGTCATCGAAGCACTCAATCCCCCCCGCGACCTCGGCCATCA

11101 CCCGCTGTTCCAGACGATGCTGACCTGGGACGCGGAGCACGCCACGGATCTGGCCTGGCC

11161 GCACGTACACACCGAGCTGATCGACCAGGGGAGCGCCACGATCAAGTTCGACCTGCACTT

11221 GGCGCTACAGGAAAGCGATCAGGGCATCGTCGGCAGCCTGGGGTATGCCACGGCGTTGTT

11281 CGAGCGCAGCACCATCGAACGGCATCTGGCCCAGTTCGTGACCCTGTTGCAGGGCATGGT

11341 CGCCGACGACCACGCCCGTGTCGCGCAACTGCCGCTGTTGCCTACCGACGAACGCGCGCA

11401 ATTGCAAACTTTCAACGCCACCGCCAGCGACCTGGATGGCAGCGGCTATCTGCATCGCGC

11461 GATCGAAGCACAGGCGCAGCGCACGCCCGACGCCATCGCCTTGGTGGACGATGGCGTTGA

11521 ACTGCGCTACGCCGACCTCGATGCCCGCGCCAACCAACTCGCCCATCATCTGATCGGGCT

11581 GGGCGTCGTGCCGGAATGCGTGGTCGCGGTGTGCCTGCCGCGTGGCATCGATCTGCTCGT

11641 CGCGCTGTTGGCCGTGCTCAAAGCCGGTGGCGCTTACCTGCCGCTGGATCGCGACGTCCC

11701 ATCGGCGCGCCTGCACGCCATGCTCGCCGATGCGCGGCCCAGTGTGCTGCTCGCCCATCG

11761 CGATACGGCTGCGTCGCTGGCACAGCGCGACGGCATGCACACCGTGCTGCTGGACGCCGA

11821 GCAAACAGCGTGGGCCAGCGTCGCCACGCATGCAGCGGTCGTTGCGAGCTTGCATCCACA

11881 ACATCCGGCCTACGTCATCTACACCTCCGGCTCCACCGGCACGCCGAAGGGCGTGGTCAA

11941 CACCCATGCCGCCATCGACAACCGCCTGCAATGGATGCAGCAGGCATTGCAACTGCAACC

12001 CGAGCAGCGCGTGCTGCAAAAAACTCCGGTCGGTTTCGACGTCTCGGTGTGGGAACTGTT

12061 TTGGCCGCTGCGCGTGGGCGCATGCCTGGTGCTCGCACAACCCGGCGGCCACAAGGACCC

12121 GACCTATCTGCATGCACTGATCGAAGAAGCCGCCATCGATACCGTGCACTTCGTGCCGTC

12181 GATGCTGCGGGTGTTCCTTGACGCCTTGCCGCATGGCGCATGCGCCAGTCTGCGGCGCAT

12241 CGTCTGCAGCGGCGAAGCCTTGCCCGCCGATCTGGCCCAAGCCGCACACGCACGCCTGCC

12301 GCAGGCGCGCCTGTACAACCTCTACGGCCCGACCGAAGCGGCGGTGGACGTCAGCGTCTG

12361 GGAATGCCGTGCGAGCGATGCCACCCAAGTCCCGATTGGCCGCCCCATCGCCAATACACA

12421 ACTGCACGTGTGCGATGCACATGCACAACCGGCTCCCATCGGTGTCGCCGGCGAATTGCA

12481 CATCGCCGGGGTGCAGGTGGCGCGCGGCTATCTGGGCCGTCCCGACCTAACCGCCGAACA

12541 TTTCGTCCCCGATCCATTCGCCGCGCAACCCGGCGCACGCATGTACCGCAGTGGCGATGT

12601 GGCGCGTTGGCGTCGCGATGGCGCACTCGAGTATCTCGGTCGCAACGACGACCAGGTCAA

12661 GCGACGCGGCGTGCGTATCGAACTGGGCGAAATCGCCGCCGCGCTGCGCACCTGCGCGGG

12721 CGTGCAGGATGCAGCGGTGTTGCTGCGCGAGGAAATGCAGGGCGAGCCGCGCCTGGTCGC

12781 CTACGTGGTCGGCGATGCCGACGCG

LOCUS Contig G132 10718 bp DNA linear BCT 30-JAN-2012

DEFINITION Xanthomonas spp. strain XaS3 genomic region encoding NRPS.

SOURCE Xanthomonas spp. strain XaS3

ORGANISM Xanthomonas spp. strain XaS3

Bacteria; Proteobacteria; Gammaproteobacteria; Xanthomonadales;

Xanthomonadaceae; Xanthomonas.

REFERENCE Contig G132 (bases 1 to 10718)

AUTHORS Royer et al. Submitted to BMC Genomics.

TITLE Genome mining indicates that the genus Xanthomonas is a promising

reservoir for new bioactive non-ribosomally synthesized peptides

JOURNAL Submitted to BMC Genomics.

REFERENCE 2 (bases 1 to 10718)

FEATURES Location/Qualifiers

source 1..10718

/organism='Xanthomonas spp.'

/mol_type='other DNA'

/strain='XaS3'

/isolation_source='sugarcane leaf water droplets'

/host='Sugarcane'

/db_xref='taxon: GPE 39'

/country='Guadeloupe'

/collection_date='2003'

/collected_by='Rosiane BOISNE-NOC'

/note='[cultured bacterial source]'

gene complement (3431..10717>)

/gene ='NRPS'

CDS complement (3431..10717>)

/gene ='NRPS'

/note='incomplete NRPS, no start codon'

/codon_start=1

/translation='ILDTHGAPAPIGVVGELYIGGDGVGRGYLNREALTAERFLTDPFSTDPTARMYRTGDLGRWRVDGTIEFVGRNDQQVKIRGFRIELGEIEARLSAHADVRECVLVALEGAAGTDKRLVAYWVGNEAVDAAALRTWLSDVLPDYMVPAAYVRLDRLPLTPNGKLDRKALPAPDGAAYAALTYEAPQGEIEQTIAAIWCELLGLEDIGRHDNFFALGGHSLLGVRLISRIRSALGLELPLATLFAQPRLAELAHALASANASTLPAIVSADRDAPLPLSFAQQRLWFLAQFDERAAQAYTLSGVVDLHGALDLPALQQALDHIVARHEVLRTCFLANDDGATQVIAPANVGFALTCIDLRHTPDAEVAAQHHAEQATRTPFDLSRGPLIRGCLMQLAEQQHRLLISMHHSISDGWSIGILIRELGALYAAFVQGQPNPLPPLPIQYADYSLWQRRVLDGPLLQRQLAFWREHLHGAPALLELPTDHPRPALQDYRGDSVEFAIDGELTAALRAVSQRHGTTVFMTVLAGWAVLLSRLSGQDEVVIGAPVANRTRSELEALIGFFVNAQALRIDLRGAPSVADLLAQVRATALAAQDHQDVPFEQVIEALNPERSLSAQPVFQVVLTWQNVPDAELVLPDIRLQPIQSHTADAKFDLEFSLQEQQDRIVGNLGYATALFERITIERHLAQFVTLLQGMVADDHARVAQLPLLPAEERTQLQRFTVTETTPLAPATCIHRLFDAQVQRTPDAIAVIEGQRTLRYAELDARANQLAQRLRRSGVGLESRVALYLPRSIEQVVAVLATLKAGAAYVPLDPELPSERLAFLLEDSRPRAVLTCTDLQDRLPASRAMLRVSVLTLDDSTETHTDDPGAPDVPGLCPENLAYVIYTSGSTGQPKGTLLTHAGAAHYLQWAIEHYRPQPSAVVSSSLSFDATLTSLLAPLLCGAQVELLPDHDTLDALRQRLCDPTPLGLVKLTPAHLHVLGQQLAEHATPLSPSVMVIGGEALPPATLARWQALAPHTRLINEYGPTETVVGCVVHTTTADDAHAPNGRVPIGQPIAHLRLYVLDAHGQHAPIGVAGHLHIAGPQLARGYLGRADLTAERFVPDPFAEHPGQRMYRSGDVACWRADGTLEYLGRNDDQVKLRGFRIELGEIAAALRACTGVQDAAVLLREDTPGEPRLVVYVVGEVERIDAENLRSDLVNRLPEVMLPTAYVCLETLPLTANGKLDRKALPAPDGAAYAASAYEAPQGEVEQTIAAIWRDLLGLESIGRHDHFFALGGHSLLAVRVASRLRKELGVEIGVAELFVHATLQQLAACVASSSAAILPPILPLQPDAPRVLSFAQQRLWFLSQFEGVSQAYHISGGLRLRGVLDAQALQRALDRIVARHASLRTTFALVDGQALQQIADEESGFHLLTHDLCGVTDREKELEKLLTAEAQAPFALEQGPLIRGRLIRLADDESVLFVTMHHIVSDGWSMGVLINELSVLYRAFAHGEADPLAPLPIQYADYASWQRQWLKGNVLEQQATYWREALSGAPVLLELPTDRPRPAQQDHAGAMLEVIVDPQQTQALKALSQRHGLTLYMTLLASWALLLSRLSGQDDVVIGSPVANRGRSETEGLIGFFVNTLALRVELSGSPTLAQLLASVKNRTLQAQAHQDIPFEQVVELLQPPRSLAHAPLFQVMFAWQNTPQGALDLGAIEASGLGVAQTSAQFDLSDASGLGVAQTSAQFDLSLSLMESDEGIVGSLAYATALFERSTLERWMGHWRHLLEAMVAEGAEHQAVDRLPLLDDAERYQVLTQWNATAADYPRDACVHELFEAQVAHDPSAIAVVQSEVALTYGELNARANRLAHYLRELGVRPDDRVAICVQRSVEMVVAVVAVLKAGGAYVPLDPAYPPERLAYMQSDCGALVMLTDTASRHLVEHLTASTVVVDLQADGQRWVHLSDSNPDRTAVGLTSGHLAYVIYTSGSTGMPKGAMNEHRGVVNRLVWMQEAYTLDRSEVVLQKTPISFDVSVWELFWPLLNGARVQLAQPEGHKDPVYLKTLIRETQVTTLHFVPSMLRVFVEHGDDDPCSWVKRVICSGEALPAALAERAQAVFPSAEIFNLYGPTEAAVDVTAWRYCAESENTGILPIGRPIANTQIYILDVQGAPVPIGVVGELYIGGDGVGRGYLNRDNLTAARFLMDPFSTDPTARMYRTGDLGRWRADGTLEFVGRNDHQVKIRGFRIELGEIEARLSAHAEVRECVVIALDDLARTDKRLVAYWVGAEDVTPESIGPEVLRSWLSGALPDYMIPAAYVQLERLPLTPNGKLDRKLLPAPEGAAYATHAYEAPQGAIEESIAAIWQDLLGLDTIGRYDNFFALGGHSLLAVTLIERMSQQGLQVDLHTLFTTPTLIALATKSSEEIQCKQVELISRNKNKAAIKEFKI'

gene complement (<3..3431)

/gene ='NRPS'

CDS complement (<3..3431)

/gene ='NRPS'

/note='incomplete NRPS, no stop codon'

/codon_start=1

/translation=' MKIWNFLLQLRIKNISLSLSDDGVDLIISCRDEDFTLEIQDFIRSNKALLIEWIICGKLDFLKIDRDFPGNDEPILEISLDEIGVISEITQGGSSNIQDIYPLVPLQEGIFFHHLLQREGDAYLLPNLIAFDSRSRLDAFVDALQRVIDRHDILRTAVVWEGLAAPVQVVWRHAPLVIEEVCLDGTDLDVAAQLQSRFDPRYWRMDVRQAPLMRGFAAHDPANCRWLLQLLSHHLALDHTTLEIVLEEVKCHLCGEAASLPAPLPFRNFVAQVLLGVSQEEHEAYFRTMLGDIDESCAPFGLVDVQGDGSDVEEVQLELPGRLSALLRSQARTLGVSVASLFHLSWAQVVGRATGRESAVFGTVLFGRMQGGAGVGRALGMFINTLPIRIEINSASVLESVRLVQQRLAELLQHEHAPLSLAQRCSGVSAPAPLFTSLLNYRHSGQANSAAKAMAWKGIDTLSSHERTNYPLTVSVDDVGSDFVLKVQSRRPLVADRISAFMLKALEGLADALAYAPETAMRDFDVLPDFERQQVLTQWNATAADYPRDACVHELFEAQVAHDPSAIAVVQGEVSLTYGELNARANRLAHYLRELGVRPDDRVAICVQRSVEMVIAVVAVLKAGGAYVPLDPAYPPERLAYMQSDCGALVVLTDTASRHLVEHLTASTVVVDLQADGQRWVHLSDSNPDRTAVGLTSGHLAYVIYTSGSTGMPKGAMNEHRGVVNRLVWMQEAYTLDRSEVVLQKTPISFDVSVWELFWSLLSGARMQLTESEGHKDPVYLKALIRDTQVTTLHFVPSMLRAFVEHDDDDPCSWVKRVICSGEALPAALAERAQAAFPSAEIFNLYGPTEAAVDVTAWRYCAESENTGILPIGRAIANTQIYILDVHGAPVPIGVVGELYIGGDGVGRGYLNRDDLTAARFLMDPFSTDPTARMYRTGDLGRWRADGTIEFVGRNDQQVKIRGFRIELGEIEARLGTHADVRECVVVALEDATGSDKRLVAYWVGRQDATHTPLGAESLRSWLSDTLPDYMIPAAYVQLDRLPLTPNGKLDRKALPAPDGSAYAAPVYEAPQGAIEQTIAAIWCDLLGLESIGRHDNFFALGGHSLLAVRVASRLRQELDVEIGVAELFANAALKDLAACVAS'

ORIGIN

1 GAAGAGGCCACGCACGCGGCAAGGTCCTTCAGCGCTGCATTCGCGAACAACTCCGCCACG

61 CCGATCTCGACATCCAATTCCTGACGCAGGCGAGAGGCGACCCGCACTGCCAACAACGAA

121 TGTCCACCGAGCGCAAAGAAGTTATCGTGTCGCCCGATGCTCTCCAGACCCAGCAGATCA

181 CACCAAATCGCGGCAATGGTTTGTTCGATCGCGCCCTGAGGTGCTTCATACACAGGCGCT

241 GCATAGGCTGAGCCGTCCGGTGCGGGTAATGCCTTGCGATCCAGCTTGCCGTTCGGGGTC

301 AGCGGTAAGCGATCCAACTGCACATAGGCTGCTGGGATCATGTAATCCGGCAAGGTATCC

361 GACAGCCAACTGCGCAAGCTTTCCGCGCCAAGAGGCGTGTGCGTCGCATCCTGCCTACCA

421 ACCCAATACGCCACCAATCGCTTGTCGCTACCCGTGGCATCTTCCAGCGCCACCACCACA

481 CACTCGCGCACATCCGCATGTGTGCCCAGCCGTGCCTCAATCTCTCCTAATTCAATACGG

541 AAACCACGAATCTTGACCTGCTGATCGTTGCGGCCCACAAACTCAATCGTGCCATCGGCA

601 CGCCAGCGACCCAGATCGCCAGTGCGATACATCCGTGCCGTCGGATCGGTACTGAATGGA

661 TCCATGAGGAAGCGCGCGGCGGTCAAATCGTCACGATTTAGATAACCACGCCCCACGCCA

721 TCGCCGCCAATGTACAACTCGCCCACCACCCCAATCGGCACCGGTGCGCCGTGTACATCC

781 AGGATATAGATCTGCGTGTTCGCAATCGCACGACCAATCGGCAGGATCCCGGTGTTTTCC

841 GACTCGGCGCAGTAACGCCAGGCCGTGACATCCACTGCCGCTTCTGTCGGCCCATAAAGG

901 TTGAAAATCTCAGCTGATGGAAAAGCCGCCTGGGCACGCTCTGCAAGCGCTGCCGGCAAG

961 GCTTCGCCACTACAAATAACCCGCTTGACCCAGGAACACGGATCATCATCGTCATGCTCG

1021 ACAAATGCCCGCAGCATCGACGGCACGAAATGCAGCGTGGTGACTTGCGTATCGCGGATC

1081 AACGCCTTCAGATACACCGGATCTTTATGGCCTTCTGATTCGGTCAACTGCATCCGCGCA

1141 CCGCTCAGCAGCGACCAGAACAGTTCCCAGACCGAGACATCGAAGCTGATCGGTGTTTTT

1201 TGTAAAACAACCTCGGATCGATCCAGCGTGTAGGCTTCTTGCATCCACACAAGGCGGTTG

1261 ACGACGCCGCGATGTTCATTCATCGCGCCCTTCGGCATACCAGTCGATCCGGACGTGTAG

1321 ATCACATACGCCAGATGCCCCGAGGTCAGACCAACGGCGGTGCGATCCGGATTACTGTCG

1381 GACAGGTGTACCCAGCGCTGGCCGTCGGCTTGCAGATCGACGACCACCGTTGAAGCAGTC

1441 AGATGCTCGACCAAGTGGCGGCTGGCGGTATCCGTCAACACCACCAACGCGCCGCAATCT

1501 GACTGCATATAGGCCAGTCGCTCCGGTGGATAGGCTGGGTCCAGCGGTACATACGCACCG

1561 CCCGCCTTCAATACAGCAACCACCGCGATCACCATCTCCACACTACGCTGCACACAGATC

1621 GCCACGCGATCATCCGGGCGCACACCCAACTCACGCAGGTAATGCGCCAGACGGTTGGCA

1681 CGTGCGTTCAACTCGCCATAGGTCAGCGACACCTCGCCCTGCACTACCGCGATGGCAGAT

1741 GGATCATGTGCCACCTGTGCCTCGAACAACTCGTGCACACAGGCATCGCGCGGATAATCT

1801 GCTGCTGTTGCATTCCACTGCGTCAGTACCTGCTGCCGTTCAAAGTCAGGTAGAACATCG

1861 AAATCACGCATTGCTGTCTCTGGCGCATAAGCAAGGGCATCGGCAAGCCCCTCCAACGCC

1921 TTCAACATAAACGCGGATATACGATCCGCGACCAGTGGACGCCGCGACTGCACCTTCAGT

1981 ACAAAATCCGATCCCACATCATCCACAGAAACGGTAAGCGGATAATTCGTTCGTTCATGA

2041 CTCGATAGCGTGTCGATCCCCTTCCATGCCATCGCTTTGGCAGCACTATTCGCTTGGCCA

2101 CTATGGCGGTAGTTGAGCAGCGAGGTAAACAACGGTGCCGGTGCCGAAACACCACTGCAA

2161 CGCTGCGCAAGCGACAGCGGCGCATGCTCGTGCTGCAAAAGCTCGGCAAGTCGCTGTTGT

2221 ACCAAACGCACGCTTTCTAGCACGCTAGCACTATTAATTTCGATGCGTATCGGCAAGGTA

2281 TTGATGAACATTCCCAGTGCACGACCGACCCCAGCTCCGCCCTGCATACGTCCAAACAAC

2341 ACTGTCCCGAACACAGCACTCTCACGCCCCGTGGCCCGCCCCACCACTTGCGCCCAGGAC

2401 AGATGAAACAGACTGGCTACGCTCACGCCGAGCGTGCGTGCCTGACTCCGCAGCAATGCC

2461 GACAGCCGACCCGGTAGTTCGAGCTGCACCTCTTCCACATCGGATCCATCTCCCTGTACA

2521 TCAACCAGACCGAACGGCGCACAGGATTCATCGATATCACCGAGCATCGTGCGGAAATAC

2581 GCCTCGTGCTCCTCTTGGCTCACACCCAGCAGCACCTGCGCGACAAAATTACGAAACGGC

2641 AGGGGGGCAGGCAAGCTAGCCGCTTCACCACACAAGTGACATTTAACCTCTTCCAGCACG

2701 ATCTCCAACGTGGTGTGATCTAGAGCAAGGTGGTGACTGAGCAACTGCAGCAGCCAACGG

2761 CAATTGGCCGGATCGTGCGCCGCAAAACCACGCATCAGCGGCGCCTGACGCACATCCATG

2821 CGCCAGTAGCGCGGATCGAAGCGCGATTGCAGCTGTGCCGCCACATCGAGGTCAGTGCCA

2881 TCCAGGCAAACCTCCTCAATCACCAGTGGCGCATGCCGCCACACGACCTGGACTGGTGCG

2941 GCAAGCCCTTCCCAGACCACCGCTGTACGTAGAATATCGTGACGATCGATGACACGCTGC

3001 AGCGCATCAACAAACGCGTCCAGACGCGAACGGCTGTCGAAAGCGATCAGGTTCGGCAAT

3061 AAATAAGCATCACCCTCGCGCTGCAGCAGATGGTGGAAGAAAATTCCTTCCTGTAGTGGA

3121 ACCAGTGGATAGATGTCCTGGATATTGGACGATCCACCCTGGGTTATTTCAGAAATTACT

3181 CCAATTTCATCCAGTGAAATTTCTAAAATTGGCTCATCATTTCCAGGAAAATCCCTATCA

3241 ATTTTTAAAAAATCTAACTTCCCACAAATTATCCACTCTATCAATAGAGCCTTGTTACTT

3301 CTAATGAAATCCTGAATTTCAAGAGTAAAATCCTCATCCCTGCATGAAATTATTAAGTCA

3361 ACACCATCATCACTCAATGACAGAGATATATTTTTTATTCTCAACTGAAGCAAGAAATTC

3421 CAGATTTTCATATCTTAAATTCTTTGATGGCCGCTTTATTTTTATTTCTTGAAATTAATT

3481 CAACTTGCTTGCACTGAATTTCTTCAGATGATTTAGTAGCTAATGCGATAAGCGTCGGAG

3541 TAGTGAATAAAGTGTGCAAATCGACCTGTAATCCCTGCTGACTCATACGCTCGATCAGCG

3601 TCACTGCCAGCAAGGAATGTCCACCGAGGGCAAAGAAGTTGTCATACCTGCCTATCGTGT

3661 CGAGCCCAAGTAAGTCTTGCCAGATGGCAGCAATAGATTCTTCAATTGCACCCTGTGGTG

3721 CTTCATACGCATGTGTGGCATAAGCCGCACCTTCCGGCGCCGGTAACAGCTTGCGGTCCA

3781 ACTTGCCGTTAGGAGTCAATGGCAAGCGCTCCAACTGCACATAGGCCGCTGGGATCATGT

3841 AGTCCGGTAGTGCACCCGACAGCCAACTGCGCAAAACCTCTGGACCGATGCTCTCGGGCG

3901 TCACATCCTCGGCACCGACCCAATACGCCACCAGTCGTTTGTCGGTACGCGCCAGATCAT

3961 CAAGCGCAATGACAACGCACTCGCGCACCTCCGCATGCGCACTCAACCGCGCCTCGATCT

4021 CGCCCAACTCGATTCGGAAACCACGGATTTTGACCTGGTGATCGTTGCGTCCGACAAACT

4081 CAAGCGTGCCATCGGCACGCCAGCGACCCAGATCGCCAGTGCGATACATCCGCGCCGTCG

4141 GATCGGTACTAAATGGATCCATGAGGAAGCGCGCGGCGGTCAAATTGTCGCGATTTAGAT

4201 AACCACGCCCCACGCCATCGCCGCCAATGTACAACTCGCCCACCACCCCAATCGGAACCG

4261 GTGCACCTTGTACATCGAGGATATAGATCTGCGTGTTCGCGATTGGACGACCAATCGGCA

4321 GGATCCCGGTGTTTTCCGACTCGGCGCAGTAACGCCAGGCCGTGACATCCACTGCCGCTT

4381 CTGTCGGCCCATAAAGGTTGAAAATCTCAGCTGACGGAAAAACCGCCTGGGCACGCTCTG

4441 CAAGCGCTGCCGGCAAGGCTTCGCCACTACAAATAACTCGCTTGACCCAGGAACACGGAT

4501 CATCATCGCCATGCTCGACAAATACCCGCAGCATCGATGGCACGAAATGCAGCGTGGTGA

4561 CTTGCGTCTCGCGGATCAACGTCTTCAAATACACCGGATCTTTATGGCCTTCTGGTTGCG

4621 CCAACTGCACACGCGCACCGTTCAGCAGCGGCCAAAACAGTTCCCAAACCGAGACATCGA

4681 AGCTGATCGGTGTTTTTTGTAAAACAACCTCGGATCGATCCAGCGTGTAGGCTTCTTGCA

4741 TCCACACAAGGCGGTTGACGACGCCGCGATGTTCATTCATCGCGCCCTTCGGCATACCAG

4801 TCGATCCGGACGTGTAGATCACATACGCCAGATGCCCCGAGGTCAGACCAACGGCGGTGC

4861 GATCCGGATTACTGTCGGACAGGTGTACCCAGCGCTGGCCGTCGGCTTGCAGATCGACGA

4921 CCACCGTTGAAGCAGTCAGATGCTCGACCAAGTGGCGGCTCGCGGTATCCGTCAACATCA

4981 CCAACGCACCGCAATCTGACTGCATATAGGCCAGTCGCTCCGGTGGATAGGCTGGGTCCA

5041 GCGGCACATACGCACCGCCAGCCTTCAATACAGCCACCACCGCCACCACCATCTCCACAC

5101 TGCGCTGCACGCAGATCGCCACGCGATCATCCGGGCGCACGCCTAACTCACGCAGGTAAT

5161 GCGCCAGACGGTTAGCACGCGCGTTCAACTCGCCATACGTCAGGGCCACCTCACTCTGCA

5221 CTACCGCGATGGCCGATGGATCATGTGCCACCTGTGCCTCGAACAATTCATGTACACAGG

5281 CATCGCGCGGATAATCTGCTGCTGTTGCATTCCACTGCGTCAGCACCTGATAGCGCTCGG

5341 CATCGTCGAGCAACGGAAGACGATCTACTGCCTGATGTTCGGCACCCTCGGCGACCATCG

5401 CTTCCAACAGATGCCGCCAATGGCCCATCCACCGCTCCAGCGTCGAACGCTCGAACAATG

5461 CCGTGGCATAGGCCAGACTGCCGACGATCCCCTCATCGCTCTCCATCAATGACAGCGACA

5521 GGTCGAACTGCGCGCTTGTCTGCGCAACGCCCAATCCGCTGGCATCCGACAGGTCGAACT

5581 GCGCGCTCGTCTGTGCAACACCCAATCCGCTGGCCTCGATTGCGCCAAGATCAAGCGCGC

5641 CCTGCGGTGTGTTCTGCCACGCGAACATCACTTGGAACAGTGGCGCATGCGCCAGACTGC

5701 GTGGCGGTTGCAGCAGTTCGACCACCTGCTCGAACGGGATATCCTGATGTGCCTGCGCTT

5761 GCAGTGTGCGGTTCTTCACCGAGGCCAGCAGTTGCGCCAACGTAGGTGAGCCGGACAACT

5821 CCACCCGCAACGCCAGCGTGTTGACGAAGAACCCGATCAGCCCTTCGGTTTCCGATCTCC

5881 CACGATTAGCCACCGGACTGCCGATCACCACATCGTCCTGGCCGGAAAGACGCGATAGCA

5941 GCAACGCCCAACTCGCCAGGAGGGTCATGTATAGCGTCAGGCCATGGCGCTGGCTCAGGG

6001 CCTTCAGCGCTTGTGTCTGTTGCGGATCGACGATCACCTCCAACATGGCGCCGGCATGAT

6061 CCTGCTGAGCCGGACGTGGACGATCCGTTGGCAGTTCCAGCAATACCGGTGCGCCGGACA

6121 GTGCCTCACGCCAATACGTGGCCTGTTGCTCAAGTACATTGCCCTTCAACCACTGCCGCT

6181 GCCAGCTTGCATAGTCGGCGTACTGGATCGGCAACGGTGCCAGCGGATCGGCTTCGCCAT

6241 GTGCGAACGCCCGATACAACACGCTCAGCTCGTTGATCAAAACTCCCATCGACCAACCAT

6301 CCGAGACGATGTGGTGCATCGTGACGAAGAGGACGGACTCGTCATCGGCAAGTCGTATCA

6361 GCCGGCCACGGATGAGTGGGCCTTGCTCCAACGCAAATGGCGCTTGCGCTTCCGCGGTCA

6421 GCAGTTTCTCAAGCTCTTTCTCACGATCAGTTACACCGCACAGATCGTGGGTGAGCAAAT

6481 GGAAGCCGCTTTCTTCATCAGCGATTTGCTGCAACGCCTGCCCATCGACCAGCGCGAAGG

6541 TCGTGCGCAGCGACGCATGTCGGGCCACGATGCGATCCAACGCACGTTGCAACGCCTGCG

6601 CATCCAACACACCACGCAAACGCAAGCCGCCGCTGATGTGATACGCCTGACTGACACCCT

6661 CGAACTGCGATAGGAACCAGAGCCGTTGCTGTGCAAACGACAGCACACGCGGCGCATCCG

6721 GCTGCAATGGCAGGATCGGCGGCAAGATCGCAGCGGACGAAGAGGCCACGCAGGCGGCAA

6781 GTTGCTGGAGCGTGGCGTGCACAAACAACTCGGCCACCCCGATCTCTACACCCAATTCCT

6841 TGCGCAAACGCGAAGCAACCCGCACCGCCAGTAGGGAATGCCCGCCGAGCGCGAAGAAGT

6901 GGTCGTGCCGCCCGATGCTCTCCAAACCCAGCAGATCACGCCAGATTGCGGCAATGGTTT

6961 GTTCGACCTCACCTTGCGGTGCTTCATACGCACTGGCCGCATAGGCCGCACCATCTGGTG

7021 CCGGCAATGCCTTGCGATCCAGCTTGCCGTTGGCAGTGAGTGGCAAAGTCTCGAGACACA

7081 CATAGGCCGTCGGCAGCATCACCTCGGGCAGTCGATTGACCAAATCGCTGCGCAGGTTCT

7141 CGGCGTCTATCCGCTCGACCTCGCCCACCACATAGACAACCAATCGTGGCTCGCCCGGAG

7201 TGTCCTCACGCAGCAACACCGCCGCATCCTGCACGCCCGTGCAGGCACGCAACGCGGCGG

7261 CGATTTCGCCCAGTTCGATGCGGAACCCACGCAGTTTGACTTGATCGTCGTTGCGGCCCA

7321 GATACTCCAGCGTGCCGTCGGCGCGCCAGCAGGCGACATCGCCGCTGCGGTACATGCGTT

7381 GTCCGGGGTGCTCTGCGAACGGATCGGGCACGAAGCGTTCGGCGGTCAGATCCGCACGGC

7441 CCAGATACCCGCGTGCAAGCTGAGGCCCCGCAATATGCAGATGACCGGCTACGCCAATCG

7501 GTGCGTGTTGTCCATGCGCATCGAGCACGTACAGGCGAAGATGCGCAATCGGTTGACCGA

7561 TCGGCACACGCCCGTTGGGCGCGTGTGCATCGTCAGCAGTCGTGGTGTGGACTACGCAGC

7621 CGACCACCGTCTCGGTCGGGCCGTATTCGTTGATCAGACGGGTGTGCGGTGCGAGGGCTT

7681 GCCAACGGGCCAACGTGGCGGGCGGCAGTGCTTCGCCGCCGATCACCATCACCGACGGGC

7741 TCAGCGGTGTTGCGTGCTCGGCCAATTGTTGACCCAACACGTGCAGATGCGCGGGCGTGA

7801 GTTTGACCAGACCCAGCGGGGTTGGATCACACAGACGTTGCCGCAAGGCATCGAGGGTGT

7861 CGTGGTCGGGCAGCAGTTCCACCTGCGCACCGCACAACAGCGGGGCGAGCAAACTGGTCA

7921 ACGTGGCGTCGAAGGACAACGACGAGGACACCACCGCGCTGGGCTGCGGTCGATAGTGTT

7981 CGATAGCCCATTGCAGGTAATGCGCCGCACCGGCATGGGTGAGCAAGGTGCCCTTGGGTT

8041 GGCCGGTGGAGCCGGAGGTGTAGATCACGTAGGCCAGATTCTCCGGGCACAGACCGGGGA

8101 CATCCGGTGCGCCGGGATCATCGGTGTGCGTCTCCGTGCTGTCGTCCAGGGTCAACACGC

8161 TCACGCGTAGCATCGCGCGGCTGGCTGGCAGACGATCCTGCAGATCGGTGCAGGTCAGCA

8221 CTGCGCGTGGGCGGCTGTCTTCCAGCAGGAAGGCCAGACGTTCGCTGGGCAGTTCCGGAT

8281 CCAGCGGCACATACGCCGCGCCGGCCTTGAGCGTGGCCAGCACCGCGACCACTTGTTCGA

8341 TGCTGCGCGGCAGATACAAGGCGACCCGACTCTCCAGGCCGACGCCAGAACGGCGCAGAC

8401 GCTGGGCGAGTTGGTTCGCGCGGGCGTCCAGTTCGGCATAGCGCAAGGTGCGTTGTCCTT

8461 CGATCACCGCGATGGCATCGGGCGTGCGTTGCACCTGCGCGTCGAACAAGCGATGGATGC

8521 AGGTGGCCGGAGCCAGCGGCGTGGTCTCGGTGACGGTGAAGCGCTGCAATTGGGTGCGCT

8581 CCTCCGCAGGCAACAGCGGCAATTGCGCGACACGGGCGTGGTCGTCGGCGACCATGCCCT

8641 GCAACAGGGTCACGAACTGGGCAAGATGCCGTTCGATGGTGATGCGCTCGAACAGCGCCG

8701 TGGCATACCCCAGGTTACCAACGATGCGGTCCTGCTGCTCCTGCAGCGAGAACTCCAGAT

8761 CGAACTTGGCATCGGCTGTGTGCGACTGGATCGGCTGCAATCGGATGTCGGGCAGCACCA

8821 ACTCGGCATCGGGCACGTTCTGCCAGGTGAGCACCACCTGGAACACCGGCTGCGCGGACA

8881 AACTCCGTTCCGGGTTGAGCGCCTCGATCACCTGTTCGAACGGCACGTCCTGATGATCCT

8941 GCGCGGCCAATGCCGTGGCACGCACCTGCGCCAGCAAGTCGGCGACGCTCGGTGCACCAC

9001 GTAGGTCGATGCGCAGTGCCTGGGCGTTGACGAAGAAGCCGATCAACGCTTCGAGTTCGC

9061 TACGGGTGCGGTTGGCCACGGGCGCGCCGATGACCACCTCATCTTGACCGGACAGGCGCG

9121 ACAACAGCACCGCCCAGCCGGCCAACACGGTCATGAACACAGTGGTGCCGTGACGCTGGC

9181 TCACGGCGCGCAATGCGGCGGTGAGTTCACCATCGATGGCGAATTCGACACTATCGCCGC

9241 GATAATCCTGCAAGGCCGGACGTGGGTGGTCGGTCGGCAATTCCAGCAGCGCCGGTGCGC

9301 CATGCAGATGCTCGCGCCAGAAGGCGAGTTGGCGTTGCAGCAGCGGTCCGTCGAGCACGC

9361 GGCGTTGCCACAGGCTGTAGTCGGCGTACTGGATCGGCAGGGGCGGCAGCGGATTGGGCT

9421 GACCTTGCACGAAGGCGGCGTACAGCGCGCCAAGTTCGCGAATCAATATCCCAATGGACC

9481 AGCCATCGGAGATACTGTGGTGCATGCTGATCAGCAGGCGGTGTTGCTGCTCGGCCAGCT

9541 GCATCAGGCAGCCACGGATCAACGGACCACGGCTCAGATCGAAGGGCGTGCGGGTTGCCT

9601 GTTCGGCGTGATGTTGGGCGGCGACCTCGGCATCTGGGGTGTGACGTAGATCGATGCAGG

9661 TCAGCGCGAAGCCGACGTTGGCCGGGGCGATCACCTGGGTGGCGCCGTCGTCGTTGGCGA

9721 GGAAGCAGGTGCGCAGTACCTCGTGGCGAGCGACGATGTGGTCCAACGCCTGTTGCAGCG

9781 CGGGCAGGTCCAGCGCACCGTGCAAGTCCACCACGCCAGAGAGGGTGTACGCCTGCGCGG

9841 CGCGTTCGTCGAATTGCGCCAGGAACCATAGCCGTTGTTGCGCGAAGGACAATGGCAGTG

9901 GTGCATCTCGATCCGCTGAGACGATAGCCGGCAAGGTGCTGGCGTTGGCGCTTGCCAGGG

9961 CGTGAGCTAACTCGGCCAGCCGCGGTTGAGCGAACAGCGTGGCCAGCGGCAGTTCCAAGC

10021 CCAGTGCGCTGCGAATGCGCGAAATCAAGCGCACGCCCAGCAACGAATGCCCGCCAAGGG

10081 CAAAGAAGTTGTCGTGCCGACCGATGTCTTCCAGACCGAGTAACTCGCACCAGATCGCGG

10141 CAATGGTTTGTTCGATCTCGCCCTGTGGTGCTTCATACGTCAACGCCGCATAGGCCGCGC

10201 CATCCGGTGCGGGTAACGCCTTGCGATCCAGCTTGCCGTTCGGGGTCAGCGGCAAGCGAT

10261 CCAACCGCACATAGGCGGCTGGCACCATGTAGTCCGGCAGTACATCCGATAGCCAAGTCC

10321 GCAATGCGGCAGCATCAACAGCTTCGTTACCGACCCAATACGCCACCAGCCGCTTATCAG

10381 TGCCTGCCGCACCCTCCAGCGCCACCAACACGCACTCACGCACATCCGCATGCGCGCTCA

10441 AGCGGGCCTCGATCTCTCCCAACTCGATGCGGAAACCACGAATCTTGACCTGCTGATCGT

10501 TGCGTCCCACAAACTCAATCGTGCCATCGACACGCCAACGGCCCAGATCGCCCGTGCGGT

10561 ACATCCGTGCCGTCGGATCGGTGCTGAATGGATCCGTGAGGAAGCGCTCAGCGGTCAATG

10621 CTTCTCGATTGAGATACCCACGCCCGACGCCATCGCCACCGATGTACAACTCGCCCACTA

10681 CCCCAATCGGTGCCGGTGCGCCGTGTGTATCCAAGATG

LOCUS Contig G134 10162 bp DNA linear BCT 30-JAN-2012

DEFINITION Xanthomonas spp. strain XaS3 genomic region encoding NRPS.

SOURCE Xanthomonas spp. strain XaS3

ORGANISM Xanthomonas spp. strain XaS3

Bacteria; Proteobacteria; Gammaproteobacteria; Xanthomonadales;

Xanthomonadaceae; Xanthomonas.

REFERENCE Contig G134 (bases 1 to 10162)

AUTHORS Royer et al. Submitted to BMC Genomics.

TITLE Genome mining indicates that the genus Xanthomonas is a promising

reservoir for new bioactive non-ribosomally synthesized peptides

JOURNAL Submitted to BMC Genomics.

REFERENCE 2 (bases 1 to 10162)

FEATURES Location/Qualifiers

source 1..10162

/organism='Xanthomonas spp.'

/mol_type='other DNA'

/strain='XaS3'

/isolation_source='sugarcane leaf water droplets'

/host='Sugarcane'

/db_xref='taxon: GPE 39'

/country='Guadeloupe'

/collection_date='2003'

/collected_by='Rosiane BOISNE-NOC'

/note='[cultured bacterial source]'

gene <2..10162>

/gene ='NRPS'

CDS <2..10162>

/gene ='NRPS'

/note='incomplete NRPS, no start codon, no stop codon'

/codon_start=1

/translation='KFDLEFSLNEQQDRIVGSLGYATALFERSTIERHLAQFVTLLQGMVADDHARVAQLPLLPADERTQLQRFTVTETAPLAPATCIHHLFEAQVRRTPDAIALREGQRLLRYAELEARANQLAQRLRRSGVGLESRVALYLPRGIDQVVAVLATLKAGAAYVPLDPELPSERLAFLLEDSRPRAVLTCTDLQDRLPASRAMLRVSVLTLDDSTEKHTDDPGAPDVPGLCPDNLAYVIYTSGSTGQPKGTLLTHAGAAHYLQWAIEHYRPQPSAVVSSSLSFDATLTSLLAPLLCGAQVELLPEYDTLDALRQRLCDPTPLGLVKLTPAHLEVLGQQLTDQQTPLSPAVMVIGGEALPPATLARWQALAPHTRLINEYGPTETVVGCVVHTTTADDAHAPNGRVPIGQPIAHLRLYVLDAHGQHAPIGVAGHLHIAGPQLARGYLGRADLTAERFIPDPFAEQPGARMYRSGDVACWRADGTLEYLGRNDDQVKLRGFRIELGEIAAALRACTGVQDAAVLLREDTPGEPRLVAYVVGEVERIDAESLRSDLVNRLPEVMLPTAYVCLETLPLTANGKLDRKALPAPDGAAYAASAYEAPQGEIEQVIAAIWRELLGLESIGRHDNFFALGGHSLLAVRVASRLRKELGVEIGVAELFVHATLQQLAACVASSSAAILPPILPLQPDAPRVLSFAQQRLWFLSQFEGVSQAYHISGGLRLRGVLDAQALQRALDRIVARHASLRTTFALVDGQALQHIADEESGFHLIAHNLCGVADGEVALQQLLVEEAQAPFALEQGPLIRGRLIRLANDESVLFVTMHHIVSDGWSMGILINELSVLYRAFACGEADPLPPLPIQYADYASWQRQWLKGNVLEQQATYWRETLSGAPVLLELPTDRPRPARQDHAGAMLQVIVEPQQAQALKALSQRHGLTLYMTLLASWALLLSRLSGQDDVVIGSPVANRGRSETEGLIGFFVNTLALRVELSGSPTLAQLLALVKNRTLQAQAHQDIPFEQVVELLQPPRSLAHAPLFQVMFAWQNTPQGELDLGELDASGLGVAQTSAQFDLSLSLTESEEGIVGSLAYATALFERSTLERWMGHWRHLLDAMVAEGAEHQAVDRLPLLDDAERYQLLTQWNATAADYPRDACVHELFEAQVAHDPSAIAVVQGEVSLTYDELNARANRLAHYGEVALTYGELNARANRLAHYLRELGVRPDDRVAICVQRSVEMVIAVVAVLKAGGAYVPLDPAYPPERLAYMQSDCGAVVMLTDTVSRHLVEHPAASTVVVDLQADGQRWAHLPDSNPDRTAVGLTSGHLAYVIYTSGSTGMPKGAMNEHRGVVNRLVWMQEAYTLDRSEVVLQKTPMSFDVSVWELFWSLLSGARMQLTESEGHKDPVYLKALIRDTQVTTLHFVPSMLRAFVEHGDDDPCTGVRRVICSGEALPAALAERAQAVFPSAEIFNLYGPTEAAVDVTAWRYCAESENTGILPIGRPIANTQIYILDVQGAPVPIGVVDELYIGGDGVGRGYLNHDDLTAARFLMDPFSADPTARMYRTGDLGRWRADGTLEFVGRNDHQVKIRGFRIELGEIEARLSAHADVRECVVIALDDLARTDKRLVAYWVGAEDVTPESIGPEVLRSWLSGALPDYMIPAAYVQLERLPYMLPAAYVQLDRLPLTPNGKLDRNALPAPDGTAYAIHAYEAPQGEVEQAIAAIWCDLLGLDTIGRHDNFFALGGHSLLAISLIERLRRQGWQLQVRALFSAPTLADLANTLTAVSTLSIPPNRIASDCTRITPELLPLVELSQTEIDAAVATVDGGTANVQDIYPLAPLQEGLLFHHLASPEGDAYLNISVLTFDSRTHLDAFVAALQAVIARHDILRTGFVWQGLRTPVQVVWRHAPLPLQTHVIEDPNVLDALRERMDPSRFRLDVSRAPLIHAHLVEDPAHARWLLGLHSHHLMMDHTTLELLVEEVQAHLHGQQAQLPAPLPFRNFVAHARLGVSEAEHRAFFTQHLGDLETPTAPFDLWDVRGTGADIEQTLQPLPDVLSSAVRQHARQLGVSPSSLFHLACALVLAQASGQDDVVFGTTLFGRMQGGSGADRVLGMFLNTLPIRLRRDGRSVAEAIRQTQQQLAQLLHHEHAPLALAQRCSSIAPPTPLFTALLNYRYVGGSAVQTPSEPTQQPHDWHGLEMLAGLDRNNYPLTISIDDITATGGFAVEVKVNRHIGTERVIALMQQSMQVLIQALEQAPDTALYALSLLPTDERAQLQRFTVTETAPLAPATCIHRLFEAQVQRTPDAIAVREGTHTLRYAELDARANRLAQRLRRAGVGLESRVALYLPRGIDQVVAVLATLKAGAAYVPLDSELPSERLAFLLEDSRPRAVLTCTDLQDRLPASRAMLRVSVLTLDDNTDTQHDDPGAPDVPGLCPDNLAYVIYTSGSTGQPKGTLLTHAGATHYLQYAIDTYRPQPSAVVSSSLSFDATLTSLLAPLLCGAQVELLPEHDTLDALRQRLCDPTPLGLVKLTPAHLEVLGQQLTDQQTPLSPAVMVIGGEALPPATLARWQALAPHTRLINEYGPTETVVGCAVHTTAADDAHASNGRVPIGKPIAHLRLYVLDAHGQPAPMGVAGELHIAGPQLARGYLGRADLTAERFIPDPFAEQPGARMYRSGDVACWRADGTLEYLGRNDDQVKLRGFRIELGEIAAALHACAGVQDATVLLREDVPGEPRLVAYVVGDADAYSTENLRSELANRLPEVMLPTAYVHLEALPLTANGKLDRKALPAPDGAAYAARAYEAPQGAIEQAIAAIWSDILGLETIGRHDNFFALGGHSLLAVSLTKHMRQQGLQADLRTLFANPTLAALAASSGAISVSVPPNLIKPDTEIITPELLPLVTLTQEQIDSIVAMTPGGTPNIQDIYPLAPLQEGIFFHHLLQREGDTYLLSNLIAFDSRSLLDSFVDALQCVIDRHDILRTAIAWEGLAAPVQVVWRHASLVIEDICLAQTDGNAAAQLQSRFDPQHWRLDMRKAPLICGFAAQDVVSGRWLLQLLSHHLVLDQTTLQIVLEEVRCHLCGKAASLPAALPFRNFVAQALLGVSREEHEAYFRTMLGNVDEPCAPFGVINVQGDGSDLEEARLELPDWLSAALRNHARALGVSAASLFHLAWAQVVARATGRERVVLGTILFGRMQGVAGGDQAVGLLLNTLPLRIEIDGTSVLESVHVVQQRLAALLRHEHAPLSLAQRCSDVPAPAPLFTSLLNYRHSVQIKDAADFLTLEGIEILQHNRRTSYPLMASIDDVGPGFVLTVQSQRTLFPERICAFLLKALEVLVDALIHAPQMAVRDLDVLPEAERHQLLTQWNATAVDYPRDACVHELFEMQVARAPSAIAVVQGER'

ORIGIN

1 CAAGTTCGATCTGGAGTTCTCGCTCAACGAGCAGCAGGACCGCATCGTCGGCAGCCTGGG

61 GTATGCCACGGCGCTGTTCGAGCGCAGTACGATCGAACGCCATCTGGCCCAGTTCGTGAC

121 CCTGTTGCAGGGTATGGTCGCCGACGACCACGCCCGTGTCGCGCAACTGCCGCTGTTGCC

181 TGCGGACGAGCGCACGCAATTGCAGCGCTTCACCGTCACCGAGACCGCACCGCTCGCACC

241 TGCCACCTGCATCCACCACCTGTTCGAGGCGCAGGTACGGCGCACACCCGATGCCATCGC

301 GCTGCGCGAAGGACAACGCCTGCTGCGCTACGCCGAACTGGAGGCCCGTGCCAACCAACT

361 CGCCCAGCGTCTGCGTCGCTCTGGCGTTGGTCTAGAGAGCAGGGTCGCGTTGTACTTGCC

421 GCGCGGCATTGACCAAGTGGTCGCGGTGCTCGCCACGCTCAAAGCGGGCGCGGCGTATGT

481 GCCGCTGGATCCGGAATTGCCCAGCGAACGTCTGGCCTTTCTGCTGGAAGACAGTCGTCC

541 ACGCGCAGTGCTGACCTGCACCGATCTGCAGGATCGTCTGCCAGCCAGTCGCGCCATGTT

601 GCGCGTGAGCGTGTTGACCCTGGACGACAGCACGGAGAAGCACACCGATGATCCCGGCGC

661 ACCGGATGTGCCGGGCCTGTGTCCGGACAATCTGGCCTACGTCATCTACACCTCCGGTTC

721 CACCGGCCAACCCAAGGGCACCTTGCTCACGCATGCGGGTGCGGCGCATTACCTGCAATG

781 GGCTATCGAACACTATCGACCGCAGCCCAGCGCAGTGGTGTCCTCGTCGCTGTCCTTCGA

841 CGCGACCTTGACCAGCCTGCTCGCCCCGTTGCTGTGTGGGGCGCAGGTCGAACTGCTGCC

901 CGAGTACGACACGCTCGACGCCTTACGGCAACGTCTGTGCGATCCGACCCCGTTGGGGTT

961 GGTCAAGCTCACCCCGGCGCATCTGGAAGTGTTGGGTCAGCAATTGACCGATCAGCAAAC

1021 ACCGCTGAGCCCTGCGGTCATGGTGATCGGCGGCGAAGCACTGCCGCCCGCCACGCTGGC

1081 CCGTTGGCAAGCCCTCGCACCGCACACCCGTCTGATCAACGAATACGGCCCGACCGAGAC

1141 GGTGGTCGGCTGCGTAGTCCACACCACGACTGCTGACGATGCACACGCGCCCAACGGGCG

1201 TGTGCCGATCGGTCAACCGATTGCGCATCTTCGCCTGTACGTGCTCGATGCGCATGGACA

1261 ACACGCACCGATTGGCGTGGCCGGTCATCTGCATATTGCGGGGCCGCAGTTAGCACGTGG

1321 ATATCTCGGCCGTGCGGATCTGACCGCCGAACGCTTCATCCCCGATCCGTTCGCCGAGCA

1381 GCCAGGCGCACGCATGTATCGCAGCGGCGATGTCGCCTGCTGGCGCGCCGACGGCACGCT

1441 GGAGTATTTGGGTCGCAACGACGATCAAGTCAAACTGCGCGGGTTCCGCATCGAACTGGG

1501 CGAAATCGCCGCCGCGTTGCGTGCCTGCACGGGCGTGCAAGATGCGGCGGTGTTGCTGCG

1561 TGAGGACACTCCGGGCGAGCCACGATTGGTTGCCTATGTGGTGGGCGAGGTCGAGCGGAT

1621 AGACGCCGAGAGCCTACGCAGCGATTTGGTCAATCGCCTGCCCGAGGTGATGCTGCCGAC

1681 GGCCTATGTGTGTCTCGAGACCTTGCCACTCACCGCCAACGGCAAGTTGGATCGCAAGGC

1741 ATTGCCGGCACCAGATGGTGCGGCCTATGCGGCCAGTGCCTATGAAGCACCGCAAGGTGA

1801 GATCGAACAAGTCATTGCGGCAATCTGGCGTGAACTGCTGGGTCTGGAGAGCATCGGGCG

1861 GCACGATAACTTCTTCGCGCTCGGTGGACATTCGTTGTTGGCGGTGCGGGTCGCCTCTCG

1921 CCTGCGCAAGGAATTGGGTGTGGAGATCGGGGTGGCCGAGTTGTTTGTGCACGCCACGCT

1981 CCAGCAGCTTGCCGCGTGTGTGGCCTCTTCGTCCGCTGCGATCTTGCCACCGATCCTGCC

2041 ATTGCAGCCGGATGCGCCGCGTGTGCTGTCGTTTGCACAGCAACGGCTCTGGTTCCTGTC

2101 GCAGTTCGAGGGTGTCAGTCAGGCGTATCACATCAGTGGCGGGCTAAGGTTGCGTGGTGT

2161 GTTGGATGCGCAGGCGTTGCAACGTGCGTTGGATCGCATCGTGGCCCGACATGCGTCGCT

2221 GCGCACGACATTCGCGCTGGTCGATGGACAGGCGTTGCAGCACATCGCTGATGAAGAAAG

2281 CGGCTTCCATCTGATCGCCCACAATCTGTGTGGTGTAGCTGATGGTGAGGTGGCGCTGCA

2341 GCAACTGTTAGTCGAAGAAGCGCAAGCGCCATTTGCGTTGGAGCAAGGCCCACTCATCCG

2401 TGGCCGGCTGATAAGACTTGCCAACGACGAATCCGTCCTCTTCGTCACGATGCACCACAT

2461 CGTCTCGGATGGGTGGTCGATGGGGATCTTGATCAACGAGCTGAGCGTGTTGTATCGGGC

2521 GTTTGCATGTGGCGAAGCCGATCCGCTGCCACCGTTGCCAATTCAGTACGCCGACTATGC

2581 AAGTTGGCAGCGGCAGTGGTTGAAGGGCAATGTACTTGAGCAACAGGCTACGTATTGGCG

2641 CGAGACGCTATCTGGTGCGCCGGTGTTGCTTGAACTGCCCACGGATCGTCCGCGTCCAGC

2701 GCGTCAGGACCATGCCGGTGCGATGTTGCAGGTGATCGTGGAGCCGCAACAGGCACAAGC

2761 GTTGAAGGCGCTGAGCCAGCGCCATGGCCTGACGCTATATATGACCCTGCTGGCGAGTTG

2821 GGCGTTGCTGCTATCGCGTCTATCCGGCCAAGACGATGTGGTGATCGGCAGTCCGGTGGC

2881 AAATCGCGGGAGATCAGAGACCGAAGGACTGATCGGTTTCTTCGTCAATACGCTGGCGTT

2941 GCGAGTGGAGTTGTCCGGCTCGCCGACGTTGGCGCAACTGCTGGCCTTGGTGAAGAACCG

3001 CACACTGCAAGCGCAGGCACATCAGGATATCCCGTTCGAGCAGGTGGTCGAACTGCTGCA

3061 ACCGCCACGCAGTCTGGCGCATGCGCCGCTGTTCCAAGTGATGTTTGCTTGGCAGAACAC

3121 GCCGCAGGGGGAGTTGGATCTTGGCGAGCTCGATGCCAGCGGATTGGGCGTTGCGCAGAC

3181 AAGCGCGCAGTTCGACCTGTCGCTGTCATTGACGGAGAGTGAGGAGGGGATCGTCGGCAG

3241 TCTGGCCTATGCCACGGCATTGTTCGAGCGTTCGACGCTGGAGCGGTGGATGGGCCATTG

3301 GCGGCATCTGTTGGATGCGATGGTCGCCGAGGGTGCCGAACATCAGGCAGTAGATCGTCT

3361 TCCGTTGCTGGACGATGCCGAGCGCTATCAGTTGCTGACGCAGTGGAATGCAACGGCAGC

3421 AGATTATCCGCGCGATGCCTGTGTACATGAATTGTTCGAGGCACAGGTGGCACATGATCC

3481 ATCGGCCATCGCGGTAGTGCAGGGCGAGGTGTCGCTGACCTATGACGAGTTGAACGCGCG

3541 TGCCAACCGTCTGGCGCATTACGGTGAGGTGGCCCTGACGTATGGCGAGTTGAACGCGCG

3601 TGCCAACCGTCTGGCGCATTACCTGCGTGAGTTGGGCGTGCGCCCGGATGATCGTGTGGC

3661 GATCTGTGTGCAGCGTAGTGTGGAGATGGTGATTGCGGTGGTTGCTGTATTGAAGGCGGG

3721 CGGTGCGTATGTACCGCTGGACCCAGCCTATCCACCGGAGCGGCTGGCCTATATGCAGTC

3781 AGATTGTGGCGCGGTGGTGATGTTGACGGATACCGTCAGCCGCCACTTGGTCGAGCATCC

3841 GGCTGCTTCGACAGTGGTCGTCGATCTGCAAGCCGACGGCCAGCGCTGGGCACACCTGCC

3901 CGACAGTAATCCGGATCGCACCGCCGTTGGTCTGACCTCGGGGCATCTGGCGTATGTGAT

3961 CTACACGTCCGGATCGACCGGTATGCCGAAGGGCGCGATGAATGAACATCGTGGGGTCGT

4021 CAATCGTCTCGTGTGGATGCAAGAGGCCTATACGCTGGATCGATCCGAGGTTGTTTTACA

4081 AAAAACACCGATGAGCTTCGATGTCTCGGTCTGGGAACTGTTCTGGTCGCTGCTGAGCGG

4141 TGCGCGGATGCAGTTGACCGAATCAGAAGGCCATAAAGATCCGGTGTATCTGAAGGCGTT

4201 GATCCGCGATACGCAAGTCACCACGCTGCATTTCGTGCCGTCGATGCTGCGGGCATTTGT

4261 CGAGCATGGCGATGATGATCCGTGTACTGGGGTCAGGCGGGTTATTTGTAGTGGCGAAGC

4321 CTTGCCGGCAGCGCTTGCAGAACGCGCCCAGGCGGTTTTTCCATCAGCTGAGATTTTCAA

4381 CCTTTATGGACCGACAGAAGCAGCAGTGGATGTCACGGCCTGGCGTTACTGCGCCGAGTC

4441 GGAAAACACTGGGATCCTGCCGATTGGTCGCCCGATTGCGAACACGCAGATCTATATCCT

4501 CGATGTACAAGGCGCACCGGTTCCGATTGGGGTGGTGGACGAGTTGTACATTGGCGGAGA

4561 TGGCGTGGGGCGTGGTTATCTAAATCACGACGATTTGACTGCCGCGCGCTTCCTCATGGA

4621 TCCATTCAGTGCCGATCCGACGGCACGGATGTATCGCACCGGCGATCTTGGTCGCTGGCG

4681 TGCCGATGGCACGCTTGAGTTTGTCGGACGCAACGATCACCAGGTCAAGATCCGTGGTTT

4741 CCGAATCGAGTTGGGCGAGATCGAGGCGCGGTTGAGTGCGCATGCGGATGTGCGCGAGTG

4801 CGTTGTCATTGCGCTTGATGATCTGGCGCGTACCGACAAACGACTGGTGGCGTATTGGGT

4861 CGGTGCCGAGGATGTGACGCCCGAGAGCATCGGTCCAGAGGTTTTGCGCAGTTGGCTGTC

4921 GGGTGCACTACCGGACTACATGATCCCAGCGGCCTATGTGCAGTTGGAGCGCTTGCCTTA

4981 CATGCTCCCAGCTGCTTATGTGCAGTTGGATCGCCTGCCGCTGACCCCGAACGGCAAGTT

5041 GGATCGCAACGCATTGCCTGCACCGGATGGCACCGCCTATGCGATACATGCGTATGAGGC

5101 ACCGCAAGGCGAGGTCGAGCAGGCCATCGCCGCGATTTGGTGTGATCTGCTGGGTCTGGA

5161 CACGATAGGTAGGCACGACAACTTCTTCGCCCTCGGCGGGCATTCGCTGTTGGCAATCAG

5221 TTTGATCGAACGCCTGCGTCGGCAGGGCTGGCAGTTGCAAGTGCGTGCATTGTTCAGCGC

5281 CCCCACGCTGGCCGACTTGGCCAACACCCTCACCGCCGTATCCACGCTCAGCATCCCGCC

5341 CAACCGCATCGCATCCGATTGCACGCGCATCACGCCAGAGTTGCTGCCGCTGGTCGAACT

5401 CAGCCAGACCGAGATCGATGCCGCCGTGGCCACCGTCGACGGCGGCACCGCCAACGTGCA

5461 GGACATCTATCCGCTGGCGCCACTGCAAGAGGGTCTGCTGTTCCATCACCTGGCCAGCCC

5521 CGAAGGCGATGCCTATCTCAACATCAGCGTACTGACCTTCGACAGCCGCACGCACCTGGA

5581 TGCCTTCGTCGCGGCGTTGCAAGCGGTCATCGCTCGTCACGACATCCTGCGTACCGGCTT

5641 CGTCTGGCAGGGATTGCGTACGCCGGTGCAAGTGGTCTGGCGGCATGCGCCGCTGCCGTT

5701 GCAAACACACGTCATCGAAGATCCCAATGTCTTGGACGCGCTGCGCGAACGCATGGACCC

5761 CAGCCGCTTCCGTCTGGATGTCAGCCGTGCACCGCTGATCCATGCCCACCTCGTCGAAGA

5821 TCCCGCGCACGCACGCTGGCTGCTCGGCCTGCATAGCCATCACCTGATGATGGATCACAC

5881 GACACTGGAGCTGTTGGTCGAGGAAGTGCAGGCGCATCTGCATGGACAGCAGGCGCAATT

5941 GCCGGCGCCGCTGCCGTTCCGCAACTTCGTCGCCCACGCACGCCTGGGGGTGAGCGAGGC

6001 CGAACACCGCGCCTTCTTCACCCAGCACCTGGGCGATCTGGAGACGCCCACCGCTCCGTT

6061 CGACCTGTGGGACGTGCGCGGCACCGGTGCCGACATCGAACAGACGTTGCAGCCGCTGCC

6121 CGACGTCCTGTCCAGCGCGGTGCGCCAGCATGCACGCCAACTCGGCGTCAGTCCCTCCAG

6181 CCTGTTCCATCTGGCCTGCGCACTGGTGTTGGCGCAAGCCAGCGGCCAAGACGATGTTGT

6241 ATTCGGCACGACCTTGTTCGGCCGCATGCAGGGTGGCAGCGGCGCCGACCGCGTCCTGGG

6301 CATGTTCCTCAACACGCTCCCGATCCGCCTGCGACGCGATGGACGCAGCGTCGCCGAAGC

6361 AATCCGCCAGACCCAGCAACAACTGGCGCAATTGCTCCATCACGAACACGCTCCATTGGC

6421 ATTGGCGCAACGCTGTAGCAGCATCGCGCCGCCGACGCCGCTGTTCACCGCATTGCTCAA

6481 CTATCGCTATGTTGGCGGCAGCGCGGTGCAGACACCCAGCGAGCCAACGCAACAGCCGCA

6541 CGACTGGCACGGCCTGGAAATGCTGGCGGGACTGGATCGCAACAACTATCCGCTGACGAT

6601 CTCGATCGATGACATCACGGCAACCGGAGGTTTCGCGGTAGAGGTGAAGGTGAACCGGCA

6661 CATCGGCACCGAGCGGGTCATCGCATTGATGCAACAAAGCATGCAGGTATTGATCCAGGC

6721 GTTGGAGCAGGCGCCGGACACAGCCCTGTACGCACTGTCGCTGTTGCCTACCGACGAACG

6781 CGCGCAATTGCAGCGCTTCACCGTCACTGAGACCGCGCCGCTGGCTCCGGCCACCTGCAT

6841 CCATCGCTTGTTCGAAGCGCAGGTGCAACGCACGCCCGATGCCATCGCGGTGCGCGAGGG

6901 TACGCACACCCTGCGCTATGCCGAACTGGATGCGCGCGCCAATCGGCTTGCTCAGCGTCT

6961 GCGTCGTGCTGGCGTTGGCCTGGAGAGTCGGGTCGCGTTGTATCTGCCGCGCGGCATTGA

7021 CCAAGTGGTCGCGGTGCTCGCCACGCTCAAGGCCGGCGCGGCGTATGTGCCGTTGGATTC

7081 GGAACTGCCCAGCGAACGTCTGGCCTTCCTGCTGGAAGACAGTCGCCCACGTGCAGTGCT

7141 GACCTGCACCGATCTGCAGGATCGTCTGCCGGCCAGCCGCGCCATGTTGCGCGTGAGCGT

7201 GCTGACCCTGGACGACAACACGGACACACAACACGATGATCCCGGCGCGCCAGATGTTCC

7261 CGGCCTGTGTCCGGACAATCTGGCCTACGTCATCTACACCTCCGGCTCCACCGGCCAACC

7321 CAAGGGCACCTTGCTCACCCATGCGGGTGCGACGCATTACTTGCAGTACGCCATCGACAC

7381 CTATCGACCGCAGCCCAGCGCCGTGGTGTCCTCGTCGCTGTCCTTCGACGCCACCTTGAC

7441 GAGTCTGCTTGCTCCGCTGCTGTGCGGTGCGCAGGTCGAACTGCTACCCGAACACGATAC

7501 CCTGGATGCCCTGCGGCAACGTCTGTGCGATCCGACCCCGTTGGGGTTGGTCAAACTCAC

7561 CCCGGCGCATCTGGAAGTGTTGGGTCAGCAATTGACCGATCAGCAAACACCGCTGAGCCC

7621 TGCGGTCATGGTGATCGGTGGCGAGGCGCTACCGCCGGCCACGCTGGCCCGTTGGCAAGC

7681 CCTCGCACCGCACACCCGTCTGATCAACGAATACGGCCCGACCGAGACCGTGGTCGGCTG

7741 CGCGGTCCACACCACCGCCGCCGACGATGCACATGCCTCCAACGGGCGTGTGCCGATCGG

7801 CAAGCCAATCGCGCATCTGCGTCTGTACGTGCTCGATGCCCACGGGCAACCCGCGCCGAT

7861 GGGCGTGGCCGGCGAATTGCACATCGCCGGGCCGCAACTGGCGCGCGGATATCTCGGTCG

7921 TGCGGACCTGACCGCCGAACGCTTCATCCCCGATCCATTCGCCGAGCAGCCAGGCGCACG

7981 CATGTACCGCAGCGGCGATGTCGCCTGCTGGCGCGCCGATGGCACATTGGAGTATCTCGG

8041 CCGCAACGACGATCAAGTCAAACTGCGCGGGTTCCGCATCGAACTGGGCGAAATCGCCGC

8101 CGCGTTGCATGCCTGCGCGGGCGTGCAGGATGCGACGGTGTTGCTGCGCGAGGATGTTCC

8161 GGGCGAACCACGATTAGTGGCCTATGTGGTCGGCGATGCCGACGCGTACAGCACCGAGAA

8221 CCTGCGCAGTGAATTGGCTAATCGCCTGCCCGAGGTAATGCTGCCGACGGCCTATGTGCA

8281 CCTGGAAGCACTCCCGCTGACCGCCAACGGCAAGCTGGATCGCAAGGCATTGCCTGCGCC

8341 GGATGGCGCGGCCTATGCCGCGCGTGCGTATGAAGCGCCGCAGGGAGCGATCGAGCAGGC

8401 CATTGCCGCGATCTGGAGTGACATCTTAGGTCTGGAAACCATCGGACGGCACGATAACTT

8461 CTTCGCTCTCGGGGGACATTCGCTACTAGCGGTGTCGCTGACCAAGCACATGCGCCAGCA

8521 GGGCCTGCAAGCCGACTTGCGCACCTTGTTTGCCAACCCGACGCTGGCGGCACTGGCCGC

8581 GTCGAGTGGTGCCATATCGGTCAGCGTTCCGCCCAATCTTATTAAACCTGATACGGAGAT

8641 AATCACGCCTGAGCTGCTGCCGCTGGTGACACTTACTCAGGAGCAGATCGATAGCATCGT

8701 TGCGATGACACCGGGTGGCACGCCTAATATTCAGGACATTTATCCACTGGCCCCATTGCA

8761 GGAAGGTATTTTCTTTCATCATCTGCTGCAGCGCGAAGGCGATACCTATCTGCTATCGAA

8821 CCTGATCGCGTTCGACAGCCGTTCACTTCTGGATAGTTTTGTCGATGCCTTGCAATGTGT

8881 GATCGATCGTCACGACATCCTGCGCACGGCGATTGCCTGGGAAGGGCTTGCCGCACCAGT

8941 GCAGGTCGTGTGGCGCCACGCGTCGCTGGTAATCGAGGATATTTGTCTGGCGCAAACTGA

9001 TGGCAATGCTGCCGCACAGTTGCAGTCGCGCTTCGATCCGCAGCATTGGCGTCTGGATAT

9061 GCGCAAGGCACCGCTGATATGCGGCTTTGCGGCACAGGATGTAGTCAGCGGCCGCTGGCT

9121 ACTGCAGCTACTCAGTCATCACCTTGTACTTGATCAAACCACGCTGCAGATCGTGCTGGA

9181 AGAAGTTAGATGCCATTTATGCGGTAAAGCTGCCAGCTTGCCGGCGGCTCTACCATTCCG

9241 CAATTTTGTGGCGCAGGCGCTACTTGGCGTAAGTCGCGAGGAGCACGAGGCGTATTTCCG

9301 GACGATGCTCGGTAATGTCGATGAGCCCTGTGCACCATTTGGCGTGATCAATGTACAAGG

9361 CGATGGATCGGACTTGGAAGAAGCACGGCTTGAGCTGCCAGATTGGTTGTCAGCAGCGTT

9421 GCGCAACCATGCGCGGGCGCTCGGCGTGAGTGCAGCCAGCTTGTTCCATCTTGCGTGGGC

9481 GCAAGTGGTGGCGCGGGCGACAGGACGTGAACGTGTTGTGCTCGGAACGATTTTGTTTGG

9541 TCGCATGCAAGGCGTCGCGGGAGGCGATCAGGCGGTTGGTTTGCTCCTCAATACCTTGCC

9601 GCTGCGTATCGAGATTGACGGGACGAGCGTGCTAGAAAGTGTACATGTGGTGCAGCAGCG

9661 TCTTGCCGCGTTATTGCGGCACGAACATGCGCCGCTTTCGCTGGCGCAGCGCTGCAGCGA

9721 TGTTCCAGCACCGGCACCTTTGTTTACCTCGCTACTCAACTATCGGCATAGCGTCCAGAT

9781 AAAAGATGCTGCCGATTTTTTGACCTTGGAGGGGATTGAGATCCTGCAGCATAATAGGCG

9841 GACGAGTTATCCGCTCATGGCATCTATCGACGATGTTGGGCCTGGCTTTGTACTGACGGT

9901 ACAGTCACAGCGGACACTTTTTCCGGAGCGCATATGTGCGTTTCTGTTAAAGGCGCTGGA

9961 AGTGCTTGTCGATGCACTTATCCATGCCCCGCAGATGGCGGTGCGCGATCTTGATGTTCT

10021 GCCCGAGGCCGAGCGTCATCAGCTATTGACGCAATGGAATGCAACAGCGGTGGATTATCC

10081 ACGTGATGCCTGTGTACATGAATTGTTCGAGATGCAGGTAGCACGTGCGCCTTCGGCAAT

10141 CGCAGTTGTACAGGGTGAGCGA

LOCUS Contig G135 9027 bp DNA linear BCT 30-JAN-2012

DEFINITION Xanthomonas spp. strain XaS3 genomic region encoding NRPS.

SOURCE Xanthomonas spp. strain XaS3

ORGANISM Xanthomonas spp. strain XaS3

Bacteria; Proteobacteria; Gammaproteobacteria; Xanthomonadales;

Xanthomonadaceae; Xanthomonas.

REFERENCE Contig G135 (bases 1 to 9027)

AUTHORS Royer et al. Submitted to BMC Genomics.

TITLE Genome mining indicates that the genus Xanthomonas is a promising

reservoir for new bioactive non-ribosomally synthesized peptides

JOURNAL Submitted to BMC Genomics.

REFERENCE 2 (bases 1 to 9027)

FEATURES Location/Qualifiers

source 1..9027

/organism='Xanthomonas spp.'

/mol_type='other DNA'

/strain='XaS3'

/isolation_source='sugarcane leaf water droplets'

/host='Sugarcane'

/db_xref='taxon: GPE 39'

/country='Guadeloupe'

/collection_date='2003'

/collected_by='Rosiane BOISNE-NOC'

/note='[cultured bacterial source]'

gene <3..9026>

/gene ='NRPS'

CDS <3..9026>

/gene ='NRPS'

/note='incomplete NRPS, no start codon, no stop codon'

/codon_start=1

/translation='FIPDPFAEQPGARMYRSGDVACWHADGTLEYLGRNDDQVKLRGFRIELGEIAAALRACAGVQDAAVLLREDTPGEPRLVAYVVGEVDAHSADSLRSQLAARLPEVMLPTAYVHLEALPLTANGKLDRKALPAPDTDALAMQAYAAPEGELETQLAEIWCDLLGVEHVGRHDSFFALGGHSLLGVRLISRIRSALGLELPLATLFAQPRLAELAQALDTAAASTLPAIVPADRDAPLPLSFAQQRLWLLHQLDARAALAYLIAGGVRLSGRLDRVALGKALDRLVARHQALRTVFRTHGEDPIQVIAAPEVGVTLQDIDLRHTQDPEAEVQRHAEQETDTPFDLARGPLIRGRLLRLGEHEHRLLLTLHHLVTDGWSTGIVLREIGALYTAFVQGQPDPLPPLPIQYPDYAVWQRRWLDGPLLQRQLDFWRAHLHDAPTLLELPTDHPRPAVQDYRGGSHEVALDAALGSALRELSQRHGTTVFMTLLAAWGVLLARLAGQDRVVIGTPTANRTRSELEPLIGLFVNTQALHLDLRTDPTVAELLAQVRATALAAQHHQDIPFEQVIEALNPIRSLAHPPLFQVMFAWQNAPSVALELPDLRLESLHSPFPISKFDLELTLQEDGARIVGSLGYAAALFDATTIQRWWHCFEQVLHALTQAEDLHVSQLPWLDAPQRHQVLAHFGSGTTATVPAQPLHRLFEAQAQRTPDAIAVIDAQRCLDYAALDAQANRLAQRLHALGLRAGAHVAIALPRSVELIVAQLAVLKCAAAYVPLDSAHPHERLRALIADAQAEVLIQGPDNPLAPAGVLCLTMAALDDDASTAPAPDIAVPVSATAYVMYTSGSTGTPKGVAVTHAAVLNLVLQDGPAGLHAEDRVGFASNPAFDSATLEVWGSLLNGATVVIVPATTMREPLALGALIAQQRLSVLILVAGVLRAYAPLIAPQLSALRLLITGGDVADPHALARVLAAGGQASVLQTYGPTESTQFVTALPLNAAPDPARRVPIGKPLANSRLYVLDRHGQPVPIGVIGELHIAGAQLAQGYLYRPDLTAERFVPDPFADAAGARMYRSGDLARWRADGQLDFLGRNDAQVKLRGFRIELGEIEATLRACAGVREAVVIAREEAGDKRLIAYVVGEYLDSAQLRTQLSTQLPEYMVPAAYVRLDALPLTVNGKLDRRALPAPDAEALEHAVYAAPCGEREHILAALWSDLLHVEQVGRHDNFFALGGHSLLAVKLIERLRRHDWQLDVRQLFASPTIAGLADHLHPLSRIVVPANRIGPDCTRITPDLLPLLALTQAEIDSVVASVEGGAANVQDIYPLAPLQEGLLFHHLADPHADPYLHSSLLGFPSREQLDGFLDALDQVIARHDILRTGFVWNGLSAPVQVVWRQATLRRYVHVSAGPDVAAHVQTWLHAPEAALVLQQAPLIHAHLAHDAAHGRWLLGLQHHHLVMDHTTLELLIEEVRVHLAGQQHQLPAPLPFRDFVAHTRAGVSTQAHQAFFSEMLADIDAPTAPFGVLAPVSEQASVHEVHRPLTPALAQAVRAQARQHGVSAASVFHLAYALLLARSSGRDEAVFATLLFGRMHASAGVDRVLGMFLNTLPIRLGGRGHSVAQALQHTQQTLARLFHHEHAPLALAQRCSGVDPALPLLNALLNYRYAGGSNVLAETAHDPLQHIQQLGGQERTHYPLVVSINDHTADGGFSLDVQCLEPIGAARVAAMLVQTVQALVQALEQPADTALHTLELLPAEERAELERFNATASDLDGSGYLHRAIEAQAQRTPDAIALVDDGVELRYADLDARANQLAHHLIGLGVVPECVVAVCLPRGIDLLVALLAVLKAGGAYLPLDRDVPSARLHAMLADARPSVLLAHRDTAASLAQRHGMHTVLLDAEQAAWASAATHAPVVASLHPQHPAYVIYTSGSTGTPKGVVNTHAAIDNRLAWGQRALLLQPEQRVLQKTPVGFDVSVWELFWPLRVGACLVLAQPGGHKDPTYLHALIEQATIDTVHFVPSMLRVFLDALPHGACASLRRIVCSGEALPADLARQTRLRLPHVRLYNLYGPTEAAVEVSAWECTAADTRSVPIGHPIANTQLHVLDAQRQQVPIGVTGELQIAGIQLARGYLGRPDLTAERFVPDPFAAHPGARMYRTGDLARWRADGAIEYLGRNDGQLKLRGVRIELGEIESALRGCSGVREAVVIARNDLPGDTRLVAYVVGDAAALDRDALRAHLGTRLPDIMLPSAYVHLDALPLTANGKLDRRALPAPEADALATQPYVAPQGERETLLAALWSELLGVEQIGRHDSFFALGGHSLLAISLIERLRQHGWQLQVRALFNAPALADLASTLTAASTLNIPPNRIAPDCTRITPELLPLVELSQTEIDAAVATVDGGTANVQDIYPLAPLQEGLLFHHLASPEGDAYLNISVLPFDSRTHLDAFLAALQAVIDRHDILRTGFAWQGLRTPVQVVWRHAPLPLQTHIIQAHDVLDALRERMDPSRFRLDVSRAPLIHAHLVEDPAHARWLLGLQSHHLIVDHTTLELLVAEVQAHLHGQQAQLPAPLPFRNFVAQARLGVSEAEHRAFFTQQLGDLETPTAPFGLWQVHGNGTDIVQAHIALPPALCNSLRTRARRLDISPASVFHLACALVLAQASGQDDVVFGTTLFGRMQGGHGADRVLGMFLNTLPIRLRRDGRSVTEALRQTQQQLAQLLHHEHAPLALAQRCSGIVPPAPLFTALLNYRHAGGSAIQMHSESAPQASDLHAVLLQERTNYPLTLSIDDIAADGGFALEIQADQQIGAARVQAMLLQAVQALVQALEQPADTALHTLELLPAEERAELERFNATTSDLDGSGYLHRAIEAQAQHTPDAIALVDDGVELRYADLDTRANQLAHHLIGLGVVPECVVAACLPRGIDLLVALLAVLKAGGAYLPLDRDVPSARLHAMLADARPSVLLAHRDTAASLAQRDGMHTVLLDAEQAAWASAATHAPVVASLHPQHPAYVIYTSGS'

ORIGIN

1 GCTTCATCCCCGATCCATTCGCCGAGCAGCCAGGCGCACGCATGTACCGCAGCGGCGATG

61 TCGCCTGCTGGCACGCCGATGGCACGCTGGAGTATCTGGGCCGCAACGACGATCAAGTCA

121 AACTGCGCGGGTTCCGTATCGAACTGGGCGAAATCGCCGCTGCGTTGCGTGCCTGCGCGG

181 GCGTGCAGGATGCGGCGGTGTTGCTGCGTGAGGACACTCCGGGCGAGCCACGATTGGTTG

241 CCTATGTGGTGGGCGAGGTCGACGCGCACAGCGCCGACAGCCTACGCAGTCAATTGGCCG

301 CTCGCCTGCCCGAGGTGATGCTGCCAACGGCCTATGTGCACCTGGAAGCGCTCCCGCTGA

361 CTGCCAACGGCAAGCTGGATCGCAAGGCATTGCCGGCACCGGATACCGACGCGCTGGCGA

421 TGCAGGCCTACGCAGCGCCGGAAGGCGAGCTGGAAACACAACTGGCCGAGATTTGGTGCG

481 ACTTGCTAGGCGTCGAACACGTCGGCCGCCACGACAGTTTCTTCGCTCTCGGCGGTCACT

541 CGCTGCTGGGGGTGCGCTTGATCTCGCGTATCCGCAGCGCGCTGGGATTGGAACTGCCGC

601 TAGCCACGCTGTTCGCGCAACCACGTCTGGCCGAGTTAGCGCAAGCGCTGGATACCGCTG

661 CCGCCAGCACGTTGCCGGCCATCGTGCCAGCCGACCGCGACGCGCCACTGCCCCTGTCCT

721 TCGCGCAACAACGGCTGTGGTTGTTACACCAACTCGACGCGCGTGCGGCGCTGGCCTATC

781 TGATCGCCGGTGGTGTGCGCCTATCCGGACGCCTGGACCGCGTCGCGCTAGGCAAGGCGC

841 TGGATCGGCTTGTCGCCCGCCATCAGGCATTGCGCACGGTCTTCCGTACGCACGGCGAAG

901 ATCCGATCCAGGTCATCGCAGCGCCCGAGGTCGGCGTGACACTGCAGGACATCGACCTAC

961 GCCACACGCAGGATCCTGAGGCCGAGGTCCAGCGTCATGCCGAACAAGAAACCGACACGC

1021 CCTTCGACCTCGCTCGCGGCCCGTTGATCCGTGGACGCCTGTTGCGCCTGGGCGAGCACG

1081 AGCATCGCTTGCTGCTCACCTTGCATCATCTCGTCACCGACGGCTGGTCCACCGGCATCG

1141 TGCTGCGTGAAATTGGCGCGCTGTACACCGCCTTCGTGCAGGGCCAACCCGATCCGCTAC

1201 CGCCACTACCGATTCAGTATCCGGATTACGCCGTGTGGCAACGCCGCTGGCTCGATGGGC

1261 CGCTGCTGCAACGGCAACTGGATTTCTGGCGCGCGCATCTGCACGACGCGCCGACACTGC

1321 TGGAATTGCCCACCGATCACCCGCGTCCGGCCGTGCAGGACTACCGCGGCGGCAGCCACG

1381 AGGTCGCCCTCGATGCTGCACTCGGCAGCGCCCTGCGCGAGCTGAGCCAACGCCACGGCA

1441 CCACCGTGTTCATGACCCTGTTGGCCGCCTGGGGCGTGCTGCTGGCACGCCTGGCCGGCC

1501 AGGATCGGGTCGTGATCGGCACCCCGACCGCCAACCGCACCCGCAGCGAACTGGAACCGT

1561 TGATCGGCCTGTTCGTCAACACGCAGGCACTGCATCTGGACCTGCGCACGGATCCAACCG

1621 TCGCCGAGTTGCTCGCGCAGGTGCGCGCCACCGCGCTCGCCGCGCAGCACCATCAGGACA

1681 TCCCCTTCGAGCAGGTGATCGAAGCGCTCAATCCCATCCGCAGCCTCGCCCACCCTCCGC

1741 TGTTCCAGGTCATGTTCGCCTGGCAGAACGCCCCCAGCGTCGCGCTGGAATTGCCCGATC

1801 TGCGACTGGAAAGCCTGCACAGCCCCTTCCCCATCAGCAAGTTCGATCTGGAACTCACGC

1861 TGCAGGAGGATGGCGCTCGCATCGTCGGCAGTCTGGGCTATGCCGCCGCGCTCTTCGATG

1921 CGACGACCATTCAACGCTGGTGGCATTGCTTCGAACAGGTCCTGCACGCGCTGACCCAGG

1981 CAGAAGACCTGCATGTTTCGCAACTGCCGTGGCTGGATGCGCCACAACGGCACCAGGTGC

2041 TGGCGCACTTCGGCAGCGGCACGACCGCCACCGTGCCGGCACAGCCGCTGCACCGGCTGT

2101 TCGAAGCGCAAGCGCAGCGCACGCCCGATGCCATCGCCGTGATCGACGCGCAGCGCTGCC

2161 TGGACTACGCCGCGCTCGATGCGCAAGCCAACCGACTGGCGCAGCGGCTGCACGCACTGG

2221 GCCTGCGCGCGGGCGCGCACGTGGCCATCGCGCTGCCGCGTTCGGTCGAGTTGATCGTCG

2281 CGCAACTGGCCGTGCTCAAGTGCGCGGCAGCCTACGTGCCGCTGGATAGCGCGCATCCGC

2341 ACGAGCGTCTGCGCGCATTGATCGCCGATGCACAGGCCGAGGTGCTGATCCAGGGGCCCG

2401 ACAATCCGCTCGCTCCGGCAGGCGTGCTCTGCCTGACCATGGCCGCTCTCGACGACGACG

2461 CCAGCACCGCGCCAGCGCCAGACATCGCCGTGCCTGTCAGCGCCACCGCGTATGTGATGT

2521 ACACCTCCGGCTCCACCGGCACACCCAAGGGCGTGGCGGTGACGCATGCGGCGGTCCTCA

2581 ACCTGGTCCTGCAGGATGGACCGGCGGGGCTGCATGCCGAAGACCGGGTCGGCTTTGCCT

2641 CCAATCCCGCCTTCGACTCGGCCACGCTAGAGGTCTGGGGCAGTCTGCTCAACGGCGCCA

2701 CCGTGGTGATTGTCCCGGCCACGACGATGCGTGAACCGCTCGCATTGGGTGCCTTGATCG

2761 CACAGCAGCGGCTATCGGTACTGATTCTGGTCGCCGGCGTGTTGCGTGCGTACGCGCCAT

2821 TGATCGCGCCACAACTGAGCGCACTGCGTTTGCTCATCACCGGTGGCGATGTCGCCGATC

2881 CGCATGCCCTGGCTCGGGTATTGGCCGCCGGCGGTCAGGCCAGCGTACTGCAAACCTATG

2941 GCCCGACCGAGAGCACGCAGTTCGTCACCGCATTGCCGCTGAACGCCGCCCCGGATCCAG

3001 CGCGGCGCGTGCCGATCGGCAAACCGCTCGCCAATAGCCGCCTGTACGTGCTCGATCGGC

3061 ATGGACAACCTGTCCCCATCGGCGTGATCGGCGAACTGCACATCGCCGGTGCGCAGCTTG

3121 CACAGGGGTATCTGTATCGCCCCGACCTGACCGCAGAGCGCTTCGTCCCCGATCCCTTTG

3181 CCGACGCTGCGGGTGCGCGCATGTACCGCAGCGGCGATCTGGCGCGTTGGCGTGCCGATG

3241 GGCAATTGGATTTCCTCGGACGCAACGATGCGCAAGTCAAACTGCGCGGCTTCCGCATCG

3301 AACTGGGCGAGATCGAGGCCACCCTGCGCGCCTGCGCCGGTGTGCGCGAGGCCGTGGTCA

3361 TCGCCCGCGAGGAGGCGGGCGACAAACGCTTGATCGCCTATGTCGTGGGCGAATACCTCG

3421 ACAGCGCGCAACTGCGCACCCAACTGAGTACGCAGTTACCCGAATACATGGTGCCAGCCG

3481 CCTATGTCCGGCTGGATGCGCTGCCGCTCACCGTCAACGGCAAGCTCGACCGCCGCGCGC

3541 TGCCGGCGCCAGACGCCGAGGCACTGGAGCACGCTGTCTATGCCGCGCCATGCGGCGAAC

3601 GCGAGCACATCCTGGCCGCGCTGTGGAGCGACCTGCTCCACGTCGAGCAGGTCGGGCGTC

3661 ACGACAACTTCTTCGCCCTCGGCGGGCATTCGCTGCTCGCGGTCAAATTGATCGAACGCC

3721 TGCGCCGGCACGACTGGCAACTCGACGTGCGCCAACTGTTCGCCAGCCCCACCATCGCCG

3781 GGTTGGCCGATCACCTGCACCCGCTCTCCCGCATCGTGGTGCCAGCCAACCGTATCGGCC

3841 CGGACTGCACCCGCATCACCCCCGATCTGCTGCCGCTGCTCGCATTGACCCAGGCAGAGA

3901 TCGACAGCGTGGTCGCCAGCGTCGAAGGTGGCGCGGCCAACGTGCAGGACATCTATCCAC

3961 TGGCGCCATTGCAGGAAGGCCTGCTGTTCCATCACCTGGCTGATCCACACGCCGACCCCT

4021 ATCTGCATTCGTCCCTGCTCGGCTTCCCCTCGCGCGAACAACTCGATGGCTTCCTCGATG

4081 CGCTCGATCAGGTCATCGCCCGCCACGACATCCTGCGCACCGGCTTCGTCTGGAATGGCC

4141 TGTCCGCTCCCGTGCAGGTGGTCTGGCGACAGGCGACACTGCGCCGGTATGTGCATGTGT

4201 CCGCCGGACCCGACGTGGCCGCGCACGTGCAGACCTGGCTGCATGCGCCCGAAGCCGCGC

4261 TCGTCCTGCAGCAGGCGCCCCTCATCCACGCCCATCTGGCCCACGACGCCGCACACGGAC

4321 GCTGGCTGCTCGGCCTGCAACACCATCACCTGGTGATGGACCACACCACCCTGGAACTGC

4381 TGATCGAAGAGGTGCGGGTCCATCTGGCCGGACAGCAACACCAGTTGCCGGCGCCCTTGC

4441 CGTTCCGCGACTTCGTCGCGCACACACGGGCCGGCGTGTCCACGCAGGCGCATCAGGCGT

4501 TCTTCAGTGAGATGCTGGCCGATATCGACGCTCCCACCGCGCCGTTCGGCGTGCTGGCGC

4561 CGGTGTCCGAGCAGGCCAGCGTCCACGAGGTGCATCGCCCCCTGACACCCGCACTGGCAC

4621 AGGCCGTGCGCGCCCAGGCGCGCCAACATGGCGTCAGCGCCGCCAGTGTGTTCCATCTGG

4681 CCTACGCGCTGTTGCTGGCGCGTAGCAGTGGTCGCGACGAGGCCGTGTTCGCCACCCTGC

4741 TGTTCGGCCGCATGCATGCCAGTGCCGGCGTGGACCGCGTGCTGGGCATGTTCCTCAACA

4801 CCTTGCCGATCCGCCTGGGCGGTCGTGGCCACAGCGTGGCGCAAGCGCTGCAACACACCC

4861 AACAGACACTGGCCCGCCTCTTCCACCACGAACATGCGCCACTGGCGCTGGCGCAACGTT

4921 GCAGCGGCGTGGATCCGGCGCTGCCGCTGCTCAACGCCCTGCTCAACTACCGCTATGCCG

4981 GTGGCAGCAACGTGCTGGCCGAGACAGCACACGATCCCCTGCAACACATACAGCAACTCG

5041 GCGGACAGGAACGCACCCATTACCCACTGGTGGTGTCGATCAACGACCACACCGCCGACG

5101 GCGGCTTCTCGCTGGACGTGCAGTGCTTGGAACCGATTGGCGCCGCACGCGTGGCGGCCA

5161 TGCTGGTGCAGACCGTGCAAGCACTGGTGCAGGCGCTGGAGCAACCAGCGGACACCGCAC

5221 TGCATACGCTGGAATTGCTACCCGCCGAAGAACGTGCCGAACTGGAACGCTTCAACGCCA

5281 CCGCCAGCGACCTGGATGGCAGCGGCTATCTGCATCGCGCGATCGAAGCACAGGCGCAGC

5341 GCACGCCCGACGCCATCGCCTTGGTGGACGATGGCGTTGAACTGCGCTACGCCGACCTCG

5401 ATGCCCGCGCCAACCAACTCGCCCATCATCTGATCGGGCTGGGCGTCGTGCCGGAATGCG

5461 TGGTCGCGGTGTGTCTGCCGCGTGGCATCGATCTGCTCGTCGCACTGTTGGCCGTGCTCA

5521 AGGCCGGTGGCGCTTACCTGCCGCTGGATCGCGACGTCCCATCGGCGCGCCTGCACGCCA

5581 TGCTCGCCGATGCGCGGCCCAGTGTGCTGCTCGCCCATCGCGATACGGCTGCGTCGCTGG

5641 CACAGCGCCACGGCATGCACACCGTGCTACTGGACGCCGAGCAAGCAGCGTGGGCCAGCG

5701 CCGCCACGCATGCACCGGTCGTTGCGAGCTTGCATCCACAACATCCGGCCTACGTCATCT

5761 ACACCTCCGGCTCCACCGGCACGCCAAAGGGCGTGGTCAACACCCATGCCGCCATCGACA

5821 ACCGATTGGCGTGGGGACAGCGTGCTTTGCTGCTGCAACCCGAGCAGCGCGTGCTGCAAA

5881 AAACTCCGGTCGGTTTCGACGTCTCGGTGTGGGAACTGTTCTGGCCGCTGCGCGTGGGCG

5941 CATGCCTGGTGCTCGCACAACCCGGCGGCCACAAGGACCCGACCTATCTGCATGCACTGA

6001 TCGAACAGGCCACCATCGATACCGTGCATTTCGTGCCGTCGATGCTGCGGGTGTTCCTAG

6061 ACGCCTTGCCGCACGGCGCATGCGCCAGCCTGCGGCGCATCGTCTGCAGCGGCGAAGCCT

6121 TGCCCGCCGATCTAGCACGCCAGACCCGTCTGCGTCTGCCGCACGTGCGGCTGTACAACC

6181 TCTACGGCCCAACCGAAGCGGCGGTGGAAGTCAGCGCATGGGAATGCACTGCGGCCGACA

6241 CCCGCAGCGTGCCGATTGGCCACCCCATCGCCAACACACAACTGCATGTACTCGATGCGC

6301 AGCGGCAGCAGGTGCCGATCGGCGTCACCGGCGAATTGCAGATCGCCGGCATACAATTGG

6361 CCCGTGGTTACCTGGGCCGCCCCGACCTGACCGCCGAACGTTTCGTCCCCGATCCGTTCG

6421 CCGCGCACCCCGGCGCACGCATGTACCGCACCGGCGATCTGGCGCGTTGGCGTGCCGATG

6481 GCGCCATCGAATATCTCGGCCGCAACGATGGACAGCTCAAGCTACGCGGCGTGCGTATCG

6541 AACTGGGCGAAATCGAAAGCGCGCTGCGTGGCTGCTCAGGTGTGCGCGAGGCAGTCGTCA

6601 TCGCCCGCAACGATCTGCCTGGGGATACGCGACTGGTCGCCTACGTGGTCGGCGATGCCG

6661 CTGCACTCGACCGCGATGCCCTGCGCGCCCACCTCGGCACGCGTCTGCCCGACATCATGC

6721 TCCCCAGCGCCTACGTGCACCTGGACGCGCTGCCGCTGACCGCCAATGGCAAACTGGATC

6781 GACGCGCGCTACCAGCGCCAGAGGCCGATGCGCTCGCGACCCAGCCTTACGTCGCCCCGC

6841 AAGGCGAACGCGAAACCCTGCTCGCCGCGCTGTGGAGTGAACTCCTCGGCGTCGAACAAA

6901 TCGGCCGCCACGACAGCTTCTTCGCCCTCGGTGGGCACTCGCTGCTGGCGATCAGTTTGA

6961 TCGAACGCCTGCGCCAGCATGGCTGGCAGTTGCAGGTGCGCGCCTTGTTCAACGCGCCCG

7021 CGCTGGCCGATCTGGCCAGCACCCTCACCGCCGCGTCCACGCTCAACATTCCCCCCAACC

7081 GCATCGCGCCCGATTGCACGCGCATCACACCGGAGTTGTTGCCGCTGGTCGAACTCAGCC

7141 AGACCGAGATCGATGCCGCCGTGGCCACCGTCGACGGCGGCACCGCCAACGTGCAGGACA

7201 TCTATCCGCTGGCGCCACTGCAAGAGGGTCTGCTGTTCCATCACCTGGCCAGCCCCGAAG

7261 GCGATGCCTACCTCAACATCAGCGTGCTGCCCTTCGACAGCCGCACGCACCTGGATGCCT

7321 TCCTCGCCGCGTTGCAAGCCGTCATCGACCGCCACGACATCCTGCGCACCGGCTTCGCCT

7381 GGCAGGGATTGCGCACACCGGTGCAAGTGGTCTGGCGGCATGCGCCGCTGCCGCTGCAGA

7441 CGCACATCATCCAGGCTCACGATGTCTTGGATGCCTTGCGCGAACGCATGGACCCCAGCC

7501 GCTTCCGTCTGGATGTCAGCCGGGCACCACTGATCCACGCCCACCTCGTCGAAGATCCCG

7561 CGCACGCACGCTGGCTGCTCGGCCTGCAAAGCCATCACCTGATCGTCGACCACACCACCC

7621 TGGAGCTACTGGTCGCCGAGGTGCAGGCGCATCTGCACGGACAACAGGCGCAGTTGCCGG

7681 CGCCGTTGCCGTTCCGCAACTTCGTCGCCCAGGCACGCCTGGGGGTGAGTGAAGCCGAAC

7741 ATCGCGCCTTCTTCACCCAGCAACTTGGCGACCTGGAGACCCCCACCGCCCCATTTGGAC

7801 TGTGGCAGGTGCATGGCAACGGCACCGACATCGTGCAAGCCCACATCGCGTTGCCCCCAG

7861 CACTGTGCAATTCACTGCGCACACGCGCACGTCGACTCGACATCAGCCCTGCCAGCGTGT

7921 TCCACCTGGCCTGTGCGCTAGTGTTGGCGCAAGCCAGCGGCCAGGACGACGTGGTGTTCG

7981 GCACGACCTTGTTCGGCCGCATGCAAGGCGGTCACGGTGCCGACCGCGTCCTGGGCATGT

8041 TCCTCAACACGCTCCCGATCCGTCTGCGCCGCGATGGACGCAGCGTCACCGAGGCACTGC

8101 GGCAGACCCAACAACAACTGGCGCAGTTACTGCATCACGAACACGCCCCGTTGGCATTGG

8161 CGCAACGCTGCAGCGGCATCGTGCCGCCGGCCCCGCTGTTCACCGCACTGCTCAACTACC

8221 GCCATGCCGGTGGCAGTGCGATACAGATGCACAGCGAATCCGCGCCGCAAGCGTCGGACC

8281 TGCACGCCGTGCTCTTGCAAGAACGCACCAACTATCCACTGACGCTCTCTATCGATGACA

8341 TTGCGGCGGACGGTGGCTTCGCCCTGGAAATACAGGCGGACCAGCAGATCGGCGCCGCAC

8401 GTGTGCAGGCCATGCTGTTACAGGCTGTGCAAGCACTGGTGCAGGCGCTGGAGCAACCAG

8461 CGGACACCGCACTGCATACGCTGGAATTGCTACCCGCCGAAGAACGTGCCGAACTGGAAC

8521 GCTTCAACGCCACCACCAGCGACCTGGATGGCAGCGGCTATCTGCATCGCGCGATCGAAG

8581 CACAGGCGCAGCACACGCCCGACGCCATCGCCTTGGTGGACGATGGCGTTGAACTGCGCT

8641 ACGCCGACCTCGACACTCGCGCCAACCAACTCGCCCATCATCTGATCGGGCTGGGCGTCG

8701 TGCCGGAATGCGTGGTCGCGGCGTGCCTGCCGCGTGGCATCGATCTGCTCGTCGCGCTGT

8761 TGGCCGTGCTCAAAGCCGGTGGCGCTTACCTGCCGCTGGATCGCGACGTCCCATCGGCGC

8821 GCCTGCACGCCATGCTCGCTGATGCGCGGCCCAGTGTGCTGCTCGCCCATCGCGACACGG

8881 CTGCGTCGCTGGCACAGCGCGACGGCATGCACACCGTGCTGCTGGACGCCGAGCAAGCAG

8941 CGTGGGCCAGCGCTGCCACGCATGCACCGGTCGTTGCGAGCTTGCATCCACAACATCCGG

9001 CCTACGTCATCTACACCTCCGGCTCCA

LOCUS Contig G137 7985 bp DNA linear BCT 30-JAN-2012

DEFINITION Xanthomonas spp. strain XaS3 genomic region encoding NRPS.

SOURCE Xanthomonas spp. strain XaS3

ORGANISM Xanthomonas spp. strain XaS3

Bacteria; Proteobacteria; Gammaproteobacteria; Xanthomonadales;

Xanthomonadaceae; Xanthomonas.

REFERENCE Contig G137 (bases 1 to 7985)

AUTHORS Royer et al. Submitted to BMC Genomics.

TITLE Genome mining indicates that the genus Xanthomonas is a promising

reservoir for new bioactive non-ribosomally synthesized peptides

JOURNAL Submitted to BMC Genomics.

REFERENCE 2 (bases 1 to 7985)

FEATURES Location/Qualifiers

source 1.. 7985

/organism='Xanthomonas spp.'

/mol_type='other DNA'

/strain='XaS3'

/isolation_source='sugarcane leaf water droplets'

/host='Sugarcane'

/db_xref='taxon: GPE 39'

/country='Guadeloupe'

/collection_date='2003'

/collected_by='Rosiane BOISNE-NOC'

/note='[cultured bacterial source]'

gene <3..4253

/gene ='NRPS'

CDS <3..4253

/gene ='NRPS'

/note='incomplete NRPS, no start codon'

/codon_start=1

/translation='TVENIEELVSLQTIVVAGDSLSSSIAKYWSQGRRLINAYGPTEATVCACMHECDPSAMGAPPIGRPIANVRIYILDVNGAPVPIGVVGELYIAGDSVGRGYLNRDDLTAERFLVDPFSDDPTAHMYRTGDLGRWRADGTIEFVVRNDHQVKIRGFRIELGEIEARLSAHADVRECVVMALEDVTGNDKRLVAYLVGSQGATSDHLGAEVLRNWLSATLPDYMVPAAYVQLDRLPLTPNGKLDRKALPTPDGAAYAARAYEAPQGAIEQAIAGIWSDLLGLEMVGRHDNFFAIGGHSLLAVRVASRLRQQLGVEIGVAELFTHATLQRLAECVASSSSVALQPIMPLEPDAPRVLSFAQQRLWFLSQFEGVSQAYHISGGLRLLGVLDAQALQRALDRIVARHASLRTTFALLDGQALQHIADEDNSFHLIDHDLRCVPDREVALQQLLVDEAQAPFALEQGPLIRGRLVRLADDESVLFVTMHHIVSDGWSMGILINELSVLYRAFARDEVDPLPTLPIQYADYASWQRQWLTGDVLEQQATYWRETLSDAPVLLELPTDRARPARQNHAGAMLEVIVDPQQAQALKALSQRHGLTLYMTLLASWALLLSRLSGQDDLVIGSPVANRGRHETEGLIGFFVNTLAMRIELSGSPTLAQLLALVKNRTLQAQAHQDIPFEQVVELIQPPRSLAHAPLFQVMFAWQNTPQGILDLGEVHASELGIAQTSAQFDLSLSLAEGEQGIVGSLTYATSLFEDATLQRWMGHWRHLLDAMVADGAEDLAVDRLPLLGKVERHQLLMEWNATAADYPRDACVHELFEAQVAREPAAIAIVHGELALTYDELNTRANQLAHCLRKLGVRPDDRVAICMQRSIEMVVALLAVLKAGAAYVPLDPAYPPERLAHILADCGAVMVLAVTDAILPAPLEVLRVNVDDTAVNAAQSDSPALHSHGGKSAYVMYTSGSTGMPKGVEIPHRGINRLVLNNGYLPFMPSDCVALAANPSFDAITFEIWGALLNGARLVVIESDVLMNPARLAETVECEGINILWMSTGLFYQYADSMKVGFGRLRYLLIGGDALDPSVVAKVMDGDRPQHFLNFYGTTETTTFASFYEIEGVDDGSRSLPIGRPIANTQIYILDRYGAPVPIGVVGELYIGGDGVGLGYLNREDLNAERFLTDPFSADPTARMYRSGDLGRWRADGTIEFVGRNDHQVKIRCFRIELGEIEVRLSAHPDVRECVVVALEEAGGNDKRLVAYWVGAEGVTSEHLGAEDLRSWLSATLPDYMVPAAYIQLDCLPVNSNGKLDRKALPAPDGAAYVVSAYEAPQGEIEQAIAVIWRDLLGLETIGRHDNFFALGGHSLLAVTLIERMRQLGLQADIQSLFATPTLVALAAHTMPLSDNNQMKYCSSSFDADSIEEVII'

gene 4253..7984>

/gene ='NRPS'

CDS 4253..7984>

/gene ='NRPS'

/note='incomplete NRPS, no stop codon'

/codon_start=1

/translation=''

ORIGIN

1 CTACAGTAGAAAACATTGAAGAACTTGTTTCTTTACAAACGATCGTTGTTGCGGGCGATT

61 CGCTCTCTTCCAGCATCGCGAAATATTGGAGTCAAGGAAGGCGCTTAATCAATGCATATG

121 GACCCACGGAAGCGACGGTCTGTGCCTGCATGCATGAATGCGATCCGAGCGCAATGGGCG

181 CGCCACCGATTGGTCGTCCGATTGCCAATGTGCGCATCTACATCTTGGATGTGAATGGTG

241 CCCCAGTTCCCATCGGCGTGGTGGGTGAGTTGTATATCGCTGGCGATAGCGTGGGGCGTG

301 GGTATCTAAACCGGGATGACTTGACTGCTGAACGCTTCCTGGTGGATCCATTTAGTGACG

361 ATCCGACGGCGCACATGTATCGCACGGGCGATCTAGGGCGCTGGCGAGCCGATGGCACGA

421 TTGAATTTGTTGTGCGCAATGATCACCAGGTCAAGATCCGTGGTTTCCGTATTGAACTCG

481 GCGAGATCGAGGCGCGGTTGAGTGCGCATGCGGATGTGCGCGAGTGCGTGGTGATGGCGC

541 TGGAGGATGTCACCGGCAACGACAAGCGACTGGTGGCGTATTTAGTCGGTAGCCAGGGGG

601 CGACGTCCGACCATCTGGGTGCGGAGGTATTGCGCAATTGGCTGTCCGCCACGCTACCGG

661 ACTACATGGTGCCGGCAGCCTATGTGCAGTTGGATCGCCTGCCATTGACTCCGAACGGCA

721 AACTGGATCGCAAGGCCCTGCCGACACCAGATGGCGCGGCTTATGCGGCGCGTGCGTATG

781 AAGCGCCGCAGGGCGCGATCGAACAGGCCATTGCTGGAATTTGGAGCGACCTGCTTGGTC

841 TGGAGATGGTCGGGCGGCACGATAACTTTTTCGCCATCGGCGGGCATTCGCTGCTGGCGG

901 TGCGGGTCGCTTCGCGCTTGCGTCAGCAGCTGGGTGTCGAGATCGGTGTGGCCGAGTTGT

961 TTACGCATGCGACGTTGCAGCGTCTTGCCGAGTGTGTGGCCTCTTCCTCTAGTGTGGCCT

1021 TGCAGCCGATCATGCCGCTGGAGCCGGATGCGCCGCGCGTGCTGTCGTTTGCTCAGCAAC

1081 GGCTCTGGTTCCTGTCGCAGTTCGAGGGTGTCAGTCAGGCGTATCACATCAGCGGCGGGC

1141 TAAGGTTGCTCGGTGTGTTGGATGCGCAGGCGCTGCAGCGTGCGTTGGATCGCATCGTGG

1201 CTCGACATGCGTCGCTGCGCACGACCTTCGCGTTGCTCGATGGGCAGGCGTTGCAGCACA

1261 TCGCTGACGAGGACAATAGCTTCCATTTGATCGACCACGATCTGCGCTGTGTGCCTGATC

1321 GTGAGGTGGCGCTGCAGCAACTGTTAGTCGATGAAGCGCAAGCGCCATTTGCACTGGAGC

1381 AAGGCCCATTAATTCGTGGCCGATTGGTTCGACTTGCAGACGATGAATCCGTCCTCTTCG

1441 TCACGATGCACCACATCGTCTCGGACGGATGGTCGATGGGAATTTTGATCAACGAGTTGA

1501 GCGTGCTGTATCGAGCTTTTGCACGTGATGAGGTCGATCCGCTGCCAACGTTGCCAATTC

1561 AATACGCCGATTATGCAAGCTGGCAGCGGCAGTGGTTGACCGGAGATGTGCTGGAGCAGC

1621 AGGCGACCTACTGGCGCGAGACGCTGTCCGATGCGCCGGTGTTGCTGGAACTGCCGACGG

1681 ATCGTGCACGTCCAGCTAGACAAAACCATGCCGGCGCGATGTTGGAGGTCATCGTCGATC

1741 CGCAGCAGGCACAGGCGTTGAAGGCCCTGAGCCAGCGCCATGGCTTGACGCTATATATGA

1801 CCCTCCTGGCGAGTTGGGCGTTGCTGCTATCGCGTCTGTCCGGGCAGGACGATTTGGTGA

1861 TTGGCAGTCCGGTGGCCAATCGTGGCCGCCATGAGACCGAAGGATTGATTGGCTTCTTCG

1921 TCAACACGCTGGCGATGCGAATTGAGTTGTCCGGCTCGCCGACGCTGGCGCAATTGCTGG

1981 CGTTGGTGAAGAACCGCACACTGCAAGCGCAGGCACATCAGGATATTCCGTTCGAGCAGG

2041 TGGTCGAACTGATTCAACCGCCACGCAGTCTGGCGCATGCGCCGCTGTTCCAAGTGATGT

2101 TTGCTTGGCAGAACACGCCGCAGGGCATTTTAGATCTCGGTGAAGTCCACGCCAGTGAAC

2161 TTGGCATCGCACAGACGAGTGCGCAGTTCGACCTGTCGTTGTCGTTAGCAGAGGGCGAGC

2221 AGGGAATTGTCGGTAGTCTGACCTATGCCACGTCGCTGTTCGAGGATGCGACATTGCAGC

2281 GGTGGATGGGGCATTGGCGGCATTTGTTGGATGCGATGGTGGCCGACGGCGCTGAAGATC

2341 TTGCGGTGGACCGTCTGCCGTTGCTGGGCAAGGTCGAACGCCATCAGCTCTTGATGGAGT

2401 GGAATGCAACGGCGGCAGATTATCCACGCGATGCCTGTGTGCACGAGTTGTTCGAGGCAC

2461 AGGTGGCCCGTGAGCCCGCGGCAATCGCCATTGTTCATGGCGAATTGGCGCTGACGTATG

2521 ACGAGCTGAATACACGTGCCAATCAGTTGGCCCATTGCCTGCGGAAGTTGGGTGTACGCC

2581 CGGATGATCGCGTGGCGATCTGCATGCAGCGCAGTATTGAAATGGTGGTGGCGCTGCTGG

2641 CGGTACTCAAGGCGGGGGCGGCGTATGTTCCGCTGGATCCAGCCTATCCGCCTGAGCGAT

2701 TGGCCCACATTCTCGCCGATTGCGGTGCCGTGATGGTGTTGGCTGTGACAGATGCCATCC

2761 TGCCAGCGCCGCTTGAGGTCTTGCGGGTCAACGTTGACGATACTGCGGTGAATGCGGCGC

2821 AATCTGACAGTCCAGCATTGCACTCGCATGGCGGTAAGAGTGCTTATGTCATGTACACCT

2881 CCGGTTCGACAGGAATGCCGAAGGGAGTCGAAATCCCGCATCGAGGGATCAATCGTCTGG

2941 TATTGAACAATGGGTATCTTCCCTTCATGCCGAGTGACTGTGTTGCTTTGGCCGCCAACC

3001 CGAGTTTTGATGCGATCACATTCGAAATCTGGGGTGCGCTGCTTAATGGCGCGCGCCTGG

3061 TCGTCATCGAATCCGATGTTCTGATGAACCCGGCACGCTTGGCAGAAACAGTCGAGTGTG

3121 AAGGGATCAATATATTATGGATGTCGACAGGTTTGTTTTATCAATATGCCGATAGCATGA

3181 AGGTTGGATTCGGTCGCTTACGTTATCTGTTGATCGGCGGCGATGCACTTGATCCAAGCG

3241 TAGTGGCGAAGGTGATGGATGGCGATAGGCCACAACATTTCTTGAATTTTTACGGCACAA

3301 CAGAAACGACGACATTTGCTTCATTTTATGAGATCGAGGGTGTCGATGATGGCTCAAGAA

3361 GTCTCCCGATCGGTCGTCCGATCGCGAACACGCAAATCTACATTCTTGATAGGTACGGCG

3421 CGCCAGTCCCGATCGGGGTCGTGGGTGAGTTGTATATCGGAGGCGATGGCGTGGGGCTTG

3481 GGTATTTGAATCGGGAAGATTTGAATGCCGAGCGCTTTCTCACGGATCCATTTAGTGCCG

3541 ATCCGACGGCGCGGATGTATCGCAGCGGCGATCTGGGGCGTTGGCGTGCCGATGGGACGA

3601 TCGAGTTTGTGGGGCGTAACGATCACCAGGTCAAGATCCGCTGCTTCCGTATCGAGCTGG

3661 GCGAGATCGAAGTACGCCTGAGCGCGCATCCGGACGTGCGCGAGTGCGTGGTGGTGGCAT

3721 TGGAAGAGGCAGGGGGTAACGACAAGCGGCTGGTGGCGTATTGGGTTGGTGCCGAGGGCG

3781 TGACCTCCGAACATCTTGGTGCAGAGGATTTGCGCAGCTGGCTGTCGGCGACTCTACCGG

3841 ATTACATGGTGCCTGCCGCTTATATACAGCTAGATTGCTTGCCCGTGAATTCGAACGGCA

3901 AGCTGGATCGTAAGGCATTGCCTGCACCTGATGGTGCTGCCTATGTGGTGAGTGCTTATG

3961 AAGCACCGCAGGGTGAAATCGAACAGGCCATTGCGGTGATCTGGCGTGACCTGCTGGGTC

4021 TGGAGACGATCGGGCGGCACGACAACTTCTTTGCATTAGGTGGACATTCGCTGTTGGCGG

4081 TGACGCTGATTGAGCGCATGCGCCAGCTGGGTCTACAGGCCGATATTCAGAGTCTTTTCG

4141 CTACGCCAACGTTGGTTGCTCTGGCAGCTCATACCATGCCATTAAGTGATAATAATCAAA

4201 TGAAATATTGCTCATCCTCTTTTGATGCTGATTCAATTGAGGAGGTTATCATATGAATAT

4261 CTCAGATCTATTCCTTGAATTTAAATTAAATAAAATATCTCTCTCATTGAGTGATGATGG

4321 ACTCAATCTGCGGATTTCTTGTAGAAAGGGGGCGCTTGATGTAGGTGCTCGTGAGTTAAT

4381 TAAAAATAACAAGGAGGCTATAATTAATGAGTTAAATAAAGGGAATTTCGATCGCTTGAG

4441 GCATGGCGATGAATTTATTCGGGACAATAGGGTTTTATTAAATATATCAAGAGGTGAAAT

4501 TCAAGATATTTCAAAAATGACGCCTGGTGGTTCTGAAAATATCCAGGACGTCTATCCGCT

4561 CGCCCCATTGCAGGAGGGCATCTTTTTCCATCACTTAATGCAGCGCGAAGGCGATGCCTA

4621 TGTGCTGCCGAATCTGATCGCGTTCGACAGCCGTTCGCGTTTGGATGCGTTCGTCGATGC

4681 GCTGCAACGCGTGATCGATCGTCACGACATCCTGCGCACGGCGGTACTCTGGGAAGAGCT

4741 TACCGCACCGGTACAGGTCGTGTGGCGGCATGCGCCGTTGATGATCCAGGAGATTTGCCT

4801 GGATGAGGCCGATGGCGATGTAGCCACACAGTTGCAATCGCGCTTCGATCCACGGCACTG

4861 GCGCATGGATGTGCGTCAGGCACCGCTGATGCGCGGCTTTGCTGCGTACGATCCGGCGAA

4921 TGATCGCTGGTTGCTGCAGCTACTCAGTCATCACCTTGCGCTCGATCACACCACGCTGGA

4981 GATTGTGTTAGAAGAAGTCAAATGTCACTTATGCGGCGAAGCCGCCAGCTTGCCCCCTTC

5041 GGTACCGTTCCGCAATTTTGTGGCGCAGTCGCTACTTGGCGTGAGCCGCGAGGAACACGA

5101 GGCGTATTTCCGCACGCTGCTCGGTGATGTGGATGAATCCTGTGCGCCGTTTGGTCTGGC

5161 CGATGTACAGAGCGATGGATCGGATGTGGAGGAAGTGCGGTTCGAGCTGCCGGCTCGGCT

5221 GTCGGCATTGTTATGCAGCCATGCACGCACACTTGGCGTGAGTGTGGCCAGCCTGTTCCA

5281 TCTGGCCTGGGCGCAGGTGGTGGCGCGTGCTACGGGTCATGAGCGTGTTGTATTCGGAAC

5341 CGTGTTGTTTGGCCGCATGCAGGGCGGCGCGGGCTCCGACCGTGCGCTGGGTATGTTTAT

5401 CAATACTTTGCCGCTGCGGATTGAGATTAATGGGTCCAGCGTGTTGGAGAGTGTGCGTTC

5461 AGTGCAACAACGCCTTGCAGCGCTGTTGCGGCATGAGCATGCACCACTATCGCTGGTGCA

5521 GCGTTGCAGTAATGTCCCAGCGCCGGCACCGTTGTTTACCTCACTACTGAATTATCGGTA

5581 TAGCGTCCAGGCGGAAGGCGCTGCCGAAGCGATGGCCTGGGAGGGAATCCAGATGCTGTC

5641 AGTTCACGAGCGGACGAATTATCCGCTTGGAATCTCTATCGACGATGTAGGCTCGGGTTT

5701 TGTACTGACGGTGCAGTCACAACGGCCACTGGTTCCGGGACGGATCTGCGCATTCATGGT

5761 GAAGGCGCTGGAAGGGCTTGCCGACGCGCTTGTCCATGCGCCTGAAACGGCGGTGCGCGA

5821 TCTCGATGTTCTGCCCGAGGCTGAGCGCCATCAGCTGCTGATGGAGTGGAATGCAACGGC

5881 AGCGGATTATACCCGCGATGCGTGTGTGCATGAATTGTTCCAAGCACAGGTGGTGCGGAC

5941 ACCCTCGGCCATCGCGGTAGTGCAGGGCGAAGTGTCGCTGACGTATGCCGAGTTGAATAC

6001 ACGTGCCAACCGGCTGGCGCATTATCTGCGTGAATTGGGCGTCGGCCCGGATGATCGCGT

6061 GGCAATCTGCGTACAGCGCAGTGTCGAGATGGTCGTGGCGCTGCTGGCGGTGCTGAAGGC

6121 CGGTGGTGCGTATGTGCCGCTGGATCCGGCTTATCCTCCAGAGCGGCTGGCCTACATGCA

6181 AGCCGATTGCGGCGCGGTGGCGGTGCTAACGGACACCACCAGCCGCGGTCTGTTCGAGGA

6241 CAGTGCCACTTCGGCGGTTATTGTTGATCTACACGCCGATGGCGAGCGCTGGGAGCATCT

6301 TCCATACAACAATTCCGACCACCATGCCAATGGTTTGACCGCTCACCATTTGGCGTATGT

6361 CATCTACACATCCGGCTCCACCGGCCGACCCAAGGGCACCTTGCTCACCCATGCCGGTGC

6421 AGCGCATTACCTGCAATGGGCTGTGCAGAACTATCAGCCGCAGCCAAGCGCGGTGGTGTC

6481 CTCGTCGCTGTCCTTCGACGCCACATTGACCAGTCTGCTCGCCCCGTTGCTGTGTGGTGC

6541 CCAGGTCGAACTGCTGCCCGAACATGACACCCTCGATGCCTTGCGGCAACGCCTGTGTGA

6601 TTCGACCCCGCTGGGGTTGGTCAAGCTCACCCCGGCGCATCTGGAAGTGTTGGGTCAGCA

6661 ATTGGCTGGCCATCCAGCACCGCTGAGCCCCGCCGTCATGGTGATCGGCGGCGAAGCCCT

6721 GCCGCCGGCCACGCTAGCCCGTTGGCAAGCCCTTGCACCGCACACCCGTATCATCAACGA

6781 GTACGGCCCCACCGAGACGGTGGTCGGCTGCGCAGTCCATACGACCACTGCCGACGATGC

6841 GCGTGCGCGCAACGGTCGCGTGCCGATCGGTCAACCGATTGCGCATCTGCGCCTGTACGT

6901 ACTCGATGCATACGGGAAATTGGCACCGATTGGCGTGCCCGGTCAGCTGCATATCGCCGG

6961 ACCACAGCTGGCGCGCGGTTATTTGGGACGCCCCGATCTGACCGCCGAACGCTTCGTGCC

7021 CGATCCATTCGCGGAATATCCCGGTGCGCGCATGTACCGCAGCGGCGATCTGGCGCGTTG

7081 GAGTGCAGATGGCAATCTCGATTATCTCGGTCGCAATGACGACCAAATCAAACTGCGCGG

7141 CTTTCGTATCGAGCTGGGCGAGATCCAGGCGCGGTTGAGCGCACATGCGGATGTGCGCGA

7201 GTGCGTGGTCGTGGCGCTGGAAGATGCGACAGGTAACGGCAAACGGCTGGTGGCGTATTG

7261 GGTCGGTGCCGAGGACGTGATGTCTGAAGATCTGGGTGCAGAGGCCCTGCGCAATTGGCT

7321 ATCGGACGTACTGCCAGATTACATGCTTCCCAGCGCATACGTACATCTAGATGACTTGCC

7381 GCTGACCCCGAACGGCAAGCTGGATCGGAAGGCGTTACCCGCACCGGATGCCGCAGCCTA

7441 TGCGGCGTGTGCGTATGAAGCGCCACAGGGCGCGATTGAACAAATCATCGCTGCGATCTG

7501 GTGCGATCTATTGGGTCTAGAGGTCATCGGGCGGCGCGACAACTTCTTCGCGCTCGGTGG

7561 ACATTCGCTGCTGGCGGTGCGGGTCGCTTCGCGCTTGCGCAAGGAATTGGGTGTCGAGAT

7621 TGGCGTGGCCGAGCTGTTTACGCATGCGACGTTGCAAGACCTTGCCGCTTGTGTGGCGTC

7681 TTCGTCCAGCGCGATCTTGCCGCCGATCCTGCCGCTGGAGACAGATGCACCGCGCGTTCT

7741 ATCGTTTGCGCAGCAACGGCTCTGGTTCCTGTCGCAGTTCGAGGGCGTTAGCCAGGCGTA

7801 TCACGTCAGCGGCGGCCTGCGCTTGCGTGGGGCATTGGATACGCAGGCGTTGCAGCGTGC

7861 ATTAGACCGTATCGTGGCCCGGCATGCGTCGCTGCGCACGACCTTCGCGTTGCTCGATGG

7921 GCAGACGTTGCAGCACATTTCTGCTGAGGACATTGGCTTCCATCGGATCGATCACGATCT

7981 GCGCG

LOCUS Contig G140 5840 bp DNA linear BCT 30-JAN-2012

DEFINITION Xanthomonas spp. strain XaS3 genomic region encoding NRPS.

SOURCE Xanthomonas spp. strain XaS3

ORGANISM Xanthomonas spp. strain XaS3

Bacteria; Proteobacteria; Gammaproteobacteria; Xanthomonadales;

Xanthomonadaceae; Xanthomonas.

REFERENCE Contig G140 (bases 1 to 5840)

AUTHORS Royer et al. Submitted to BMC Genomics.

TITLE Genome mining indicates that the genus Xanthomonas is a promising

reservoir for new bioactive non-ribosomally synthesized peptides

JOURNAL Submitted to BMC Genomics.

REFERENCE 2 (bases 1 to 5840)

FEATURES Location/Qualifiers

source 1.. 5840

/organism='Xanthomonas spp.'

/mol_type='other DNA'

/strain='XaS3'

/isolation_source='sugarcane leaf water droplets'

/host='Sugarcane'

/db_xref='taxon: GPE 39'

/country='Guadeloupe'

/collection_date='2003'

/collected_by='Rosiane BOISNE-NOC'

/note='[cultured bacterial source]'

gene complement (<1..5838>)

/gene ='NRPS'

CDS complement (<1..5838>)

/gene ='NRPS'

/note='incomplete NRPS, no start codon, no stop codon'

/codon_start=1

/translation='TVENIEELVSLQTIVVAGDSLSSTIAKYWSQGRRLINAYGPTEATVCACMHECDPSAMGAPPIGRPIANVRIYILDANGAPVPIGVVGELYIAGDSVGRGYLNREALTAERFLTDPFSTDPTARMYRSGDLGRWRADGTVEFVGRNDHQVKIRGFRIELGEIEARLSAHAEVRECVVLALETTWTEKQLVAYWVGHEAVDAAALRTWLSDVLPDYMVPAAYVQLDRLPLTPNGKLDRKALPAPDGSAYAACVYEAPQGEIEQTIAAIWGDLLGLETIGRHDNFFALGGHSLLAVRVASRLRQELGAEIGVAELFTHTTPQRLAACVEASSTSILSPIVPLEPDAPRVLSFAQQRLWFLSQFEGVSEAYHISGGLRLRGVLNTQALQRALDRIVARHTSLRTTFTLVDGQVLQQIAAEDSGFHLIAHDLRDVPDREAALQQLLADEAQAPFVLERGPLIRGRLIRLADDENVLFVTMHHIVSDGWSMGILIDELSVLYRAFARDAADPLEPLPIQYADYASWQRQWLAGEVLQQQANYWREALSGAPVLLELPTDHPRPARQDHAGAMLEVVVDSQQAQALKALSQRHGLTLYMTLLASWALLLSRLSGQDDVVIGSPVANRGRSETEGLIGFFVNTLALRLDLSGSPTLGQLLASVKERALQAQAHQDIPFEQVVELVQPPRSLAHAPLFQVMFAWQNAPQGELDLGDIEGSALGVARTSAQFDLSLSLAESEEGIVGSLTYAAALFERSTLKRWMGHWRHLLDAMVADGAEDQVVDRLPLLDDAERHQVLTQWNATTADYPRDACVHELFEAQVARTPSAIAVVQGEVSLTYGELNARANRLAHCLRELGVRPDDRVAICVQRSVEMVVALLAVLKAGGAYVPLDPAYPPERLAYMQSDCGAVLVLTDTASRHLVEHSTASTVVVDLQADGERWQHLPEHNPDRHTNGLTSRHLAYVIYTSGSTGMPKGVMIEHRGCVNLYHHYTICYLRSGDKVLVLSSFSFDLTLKNIISPLFVGCAVELAPAGVVVGSSILNLLESSGAALINCAPSQLHGVLDDFESKLRVTLLAKLRHIILGGEKIKIDLISEWISGSEQYVFINSYGPTEITDVAVDGVIKGVDVSESMPIGRPIANTRIYILDMHGAPVPIGAVGELYIGGDGVGRGYLNRDDLTAARFLADPFSADPTARMYRSGDLGRWRADGTIEFVGRNDHQVKIRGFRIELGEIEARLSAHADVRECVVVPMEDATGNDKRLVAYWVGRQDATHTPFDVESLRSWLSDMLPDYMVPAAYVQLDRLPLTPNGKLDRKALPAPDATAYAAPAYEAPQGEVEYTIAAIWRELLGLESIGRHDNFFALGGHSLLAVRVVSRLRQELGVEIRVADLFTHTTLQHLAACVASSPSATLPPILPLESDAPRVLSFAQQRLWFLSQFEGVSQAYHISGGLRLRGALDTQALQRALDRIVARHASLRTTFALVDGQALQQIAAEDCGFHLIAHDLCGVANCEEALEKLLTEEAQAPFALEQGPLIRGRLIRLADDESVLFVTMHHIVSDGWSMGILINELSVLYRSFARGQADPLAQLPIQYADYASWQRQWLTGDVLEQQATYWRETLSGAPVLLELPTDRPRPARQDHAGAMLEVVLGPQQAKALKALSQRHGLTMYMTLLASWALLLSRLSGQDDVVIGSPVANRGRSETEGLIGFFVNTLALRVELSGSPTLGQFLASVKERALLAQAHQDIPFEQVVELLQPPRSLAYAPLFQVMFVLQNTPRGELDLDEIKASGLDVAQTSAQFDLSLSLTESEEGIVGSLTYATALFERSTLERWMGYWRHLLKAMVAQSAEHQAVDCLPLLDEAERHQVLTQWNATSADYPRDACVHELFEMQVARAPSAIAVVQGERSLMYGELNARANRLAHYLRELGVRPDERVAICLQRSVEMVVSLLGV'

ORIGIN

1 CACACCTAAGAGCGACACCACCATCTCGACACTACGCTGTAAACAGATCGCCACCCGCTC

61 GTCCGGGCGCACGCCCAGTTCACGCAGGTAATGCGCCAGACGATTCGCCCGCGCGTTCAA

121 CTCACCATACATCAGCGATCGCTCACCCTGCACAACTGCAATTGCCGAAGGCGCACGTGC

181 TACCTGCATCTCGAACAATTCATGGACACAGGCATCACGCGGATAATCCGCTGACGTCGC

241 ATTCCATTGGGTCAACACCTGATGACGCTCGGCCTCGTCCAGCAACGGCAGACAGTCCAC

301 CGCCTGATGTTCAGCGCTTTGGGCCACCATCGCTTTCAATAGATGACGCCAATACCCCAT

361 CCACCGCTCCAGCGTCGAACGCTCGAACAATGCTGTGGCATAGGTCAGGCTGCCAACGAT

421 CCCCTCCTCGCTCTCGGTCAACGACAACGACAGATCGAACTGCGCGCTCGTTTGCGCAAC

481 GTCCAGCCCGCTCGCCTTGATCTCGTCAAGATCCAGCTCACCACGCGGCGTGTTCTGCAA

541 CACGAACATCACCTGGAACAATGGTGCATACGCCAAGCTCCGCGGTGGTTGCAGCAGTTC

601 CACCACTTGCTCGAACGGGATGTCCTGATGCGCCTGCGCCAGCAGCGCACGCTCCTTCAC

661 CGACGCCAGGAATTGCCCCAACGTTGGCGAGCCGGATAGCTCCACCCGTAATGCCAGGGT

721 GTTGACGAAGAATCCGATCAACCCTTCGGTCTCCGAGCGACCACGATTAGCCACCGGACT

781 GCCGATCACCACATCGTCTTGGCCGGACAGGCGTGACAGCAGTAGCGCCCAACTGGCCAG

841 TAACGTCATATACATCGTCAGGCCATGCCGCTGGCTCAGTGCCTTAAGCGCCTTTGCCTG

901 CTGCGGCCCAAGCACTACCTCCAGCATTGCGCCGGCATGGTCCTGACGCGCTGGACGCGG

961 ACGATCCGTTGGCAGCTCCAGCAATACCGGTGCACCGGATAGCGTCTCGCGCCAGTAGGT

1021 TGCTTGCTGCTCCAGCACATCCCCTGTCAACCACTGCCGCTGCCAACTTGCATAGTCGGC

1081 GTACTGGATCGGCAACTGTGCCAGCGGATCGGCTTGGCCACGTGCAAACGACCGATACAA

1141 CACGCTCAACTCGTTGATCAAAATCCCCATCGACCACCCGTCCGAGACGATGTGGTGCAT

1201 CGTGACGAAGAGGACGGACTCGTCATCGGCAAGTCGTATCAGCCGGCCACGGATGAGTGG

1261 ACCTTGCTCCAAAGCAAATGGCGCTTGCGCTTCTTCGGTCAGCAATTTCTCAAGCGCTTC

1321 CTCACAATTAGCTACACCACACAGATCGTGGGCGATCAGATGGAAACCGCAGTCCTCGGC

1381 GGCGATTTGCTGCAATGCCTGTCCATCGACCAGCGCGAAGGTCGTGCGCAGCGATGCATG

1441 CCGGGCCACGATGCGATCCAGCGCACGCTGTAGTGCCTGGGTATCCAGCGCCCCGCGCAA

1501 CCTTAGCCCGCCGCTGATGTGATACGCCTGACTGACACCCTCGAACTGCGACAGGAACCA

1561 AAGCCGCTGCTGTGCAAATGACAACACGCGCGGTGCATCCGACTCCAGTGGCAAGATTGG

1621 CGGTAGGGTCGCGCTTGGCGAGGACGCCACACACGCCGCAAGATGCTGCAGCGTGGTATG

1681 TGTAAACAGATCGGCCACACGGATCTCGACACCCAATTCCTGACGCAAGCGCGAGACGAC

1741 CCGCACCGCCAACAACGAATGTCCACCCAGAGCGAAGAAGTTGTCATGCCGCCCGATGCT

1801 CTCTAGACCCAGCAGCTCACGCCAGATCGCGGCAATGGTGTATTCAACCTCACCCTGCGG

1861 TGCTTCATATGCAGGTGCTGCATAGGCTGTAGCATCCGGTGCGGGTAATGCCTTGCGATC

1921 CAGCTTGCCGTTCGGGGTCAGCGGTAGGCGATCCAACTGCACATAAGCCGCCGGCACCAT

1981 GTAATCCGGCAACATGTCCGACAGCCAACTACGCAAGCTTTCCACGTCAAATGGCGTGTG

2041 CGTCGCATCCTGCCTACCAACCCAATACGCGACCAATCGCTTGTCGTTGCCTGTGGCATC

2101 CTCCATGGGCACCACCACGCACTCGCGCACATCCGCATGTGCGCTCAATCTTGCCTCGAT

2161 CTCGCCGAGTTCAATACGGAAACCACGAATCTTGACCTGGTGATCGTTGCGACCCACGAA

2221 CTCAATCGTGCCATCGGCACGCCAGCGCCCTAAATCGCCACTGCGATACATGCGCGCTGT

2281 CGGATCGGCACTGAATGGATCCGCGAGGAAGCGCGCGGCGGTCAAATCGTCACGATTCAA

2341 ATATCCACGCCCCACACCATCGCCGCCGATATACAACTCGCCCACCGCCCCAATCGGAAC

2401 CGGTGCACCGTGCATATCCAAGATGTAGATGCGCGTGTTAGCGATTGGACGGCCAATCGG

2461 CATCGACTCGGAAACATCAACCCCCTTGATGACGCCATCGACCGCAACGTCGGTGATTTC

2521 AGTTGGCCCATAACTATTTATAAATACATATTGCTCAGAACCTGAAATCCATTCCGAAAT

2581 CAGATCAATTTTTATCTTTTCCCCTCCAAGTATAATATGGCGAAGTTTCGCAAGTAAGGT

2641 TACTCTTAATTTAGACTCGAAATCATCTAAAACTCCATGCAATTGACTTGGCGCGCAATT

2701 TATCAATGCAGCGCCACTGGACTCAAGAAGATTCAGGATACTTGATCCGACCACCACGCC

2761 TGCCGGTGCCAACTCGACAGCACAGCCCACAAACAATGGGGAAATAATATTCTTAAGCGT

2821 CAAATCAAAAGAAAAAGAAGATAAGACCAATACCTTATCACCACTCCTTAAATAGCAAAT

2881 GGTATAGTGATGATATAAGTTGACACAACCCCGGTGCTCGATCATGACACCCTTCGGCAT

2941 ACCAGTCGATCCGGACGTGTAGATCACATACGCCAGATGCCGTGAGGTCAGGCCATTGGT

3001 ATGGCGGTCAGGATTGTGCTCAGGCAGGTGCTGCCAACGTTCACCATCGGCTTGTAGATC

3061 GACGACCACCGTTGAAGCAGTCGAATGCTCGACCAAGTGGCGGCTGGCGGTATCCGTCAA

3121 CACCAACACCGCGCCGCAATCCGACTGCATATAGGCCAGCCGCTCCGGTGGATAAGCCGG

3181 ATCTAACGGCACATACGCACCGCCCGCTTTCAACACCGCAAGCAATGCCACCACCATTTC

3241 TACACTGCGCTGCACACAGATGGCTACACGATCATCCGGGCGCACGCCCAACTCACGCAG

3301 GCAATGCGCCAGACGGTTGGCACGTGCGTTCAACTCGCCATACGTCAGCGACACTTCGCC

3361 TTGCACCACCGCAATTGCAGATGGCGTCCGTGCTACCTGCGCCTCGAACAACTCGTGTAC

3421 ACAGGCATCGCGTGGATAATCTGCTGTCGTTGCATTCCACTGGGTCAGCACCTGATGGCG

3481 CTCGGCATCGTCCAGCAACGGCAGACGATCCACCACCTGATCTTCGGCGCCGTCGGCCAC

3541 CATCGCATCCAACAGATGCCGCCAATGCCCCATCCATCGCTTCAGCGTCGAACGCTCGAA

3601 CAGCGCTGCCGCATAGGTCAGACTACCAACAATCCCCTCCTCACTTTCGGCCAACGACAG

3661 CGACAGGTCGAACTGCGCACTCGTCCGTGCAACACCCAGTGCGCTGCCCTCTATATCGCC

3721 AAGATCCAGCTCACCCTGAGGCGCGTTCTGCCACGCGAACATCACCTGGAACAAAGGTGC

3781 ATGCGCCAGACTCCGCGGTGGTTGAACCAATTCCACTACCTGCTCGAACGGAATATCCTG

3841 ATGCGCCTGTGCTTGTAATGCGCGCTCTTTCACCGATGCAAGCAATTGCCCCAACGTCGG

3901 CGAACCGGACAAATCCAGCCGCAATGCCAGGGTGTTGACGAAGAACCCGATCAGCCCTTC

3961 GGTTTCCGAACGACCACGATTGGCCACTGGGCTGCCGATGACCACATCGTCTTGCCCTGA

4021 GAGTCGCGACAGCAACAGCGCCCAACTGGCCAGTAACGTCATATACAACGTTAGGCCATG

4081 CCGTTGGCTCAGCGCCTTCAGCGCCTGCGCCTGCTGCGAATCGACGACCACCTCCAGCAT

4141 CGCCCCGGCATGGTCTTGCCGCGCTGGACGCGGATGATCCGTCGGCAGTTCCAGCAACAC

4201 CGGTGCGCCGGACAGTGCCTCGCGCCAGTAATTGGCCTGCTGCTGCAACACCTCGCCCGC

4261 CAGCCATTGCCGCTGCCAGCTCGCATAATCGGCGTACTGGATCGGCAATGGTTCCAGCGG

4321 ATCGGCCGCATCGCGTGCAAAGGCTCGATAAAGCACGCTCAACTCGTCGATCAAAATCCC

4381 CATCGACCACCCGTCCGAGACGATGTGGTGCATCGTCACGAACAGGACGTTCTCGTCATC

4441 GGCAAGTCGTATCAACCGACCACGGATCAACGGACCGCGCTCCAGTACGAACGGTGCCTG

4501 CGCCTCATCAGCCAGTAGTTGCTGCAGCGCCGCCTCGCGATCAGGCACATCCCGTAAATC

4561 GTGGGCGATCAGATGGAAGCCGCTATCTTCAGCGGCGATTTGCTGCAACACCTGCCCGTC

4621 AACCAGCGTGAACGTCGTGCGCAGCGACGTATGTCGAGCCACGATGCGATCCAACGCACG

4681 CTGCAATGCCTGAGTATTCAACACACCACGCAAACGCAAGCCACCGCTGATGTGATACGC

4741 CTCACTGACACCCTCGAACTGCGATAGGAACCAGAGCCGCTGCTGCGCAAACGACAGCAC

4801 GCGCGGCGCATCCGGTTCCAGCGGCACGATCGGCGATAAGATCGAGGTGGACGAGGCCTC

4861 CACACAAGCGGCAAGACGCTGCGGCGTCGTATGCGTAAATAGCTCGGCCACGCCGATCTC

4921 GGCCCCTAATTCCTGACGCAAGCGCGAGGCGACCCGCACCGCGAGCAAGGAATGTCCACC

4981 GAGCGCGAAGAAGTTGTCGTGCCGGCCGATCGTCTCCAGACCAAGCAGATCGCCCCAGAT

5041 TGCGGCAATCGTTTGTTCGATCTCACCCTGCGGTGCTTCATACACGCATGCTGCATAGGC

5101 TGAGCCGTCCGGTGCAGGTAATGCCTTGCGATCCAGCTTGCCGTTCGGGGTGAGTGGCAA

5161 GCGATCCAACTGCACATAGGCCGCTGGCACCATGTAATCCGGCAGTACATCCGATAGCCA

5221 AGTGCGCAATGCGGCAGCATCAACAGCTTCGTGACCGACCCAATACGCCACCAGCTGCTT

5281 CTCTGTCCATGTTGTCTCAAGCGCGAGGACCACGCACTCGCGCACCTCCGCATGCGCGCT

5341 CAAGCGTGCCTCGATCTCGCCCAACTCGATACGGAAACCACGGATCTTGACCTGGTGATC

5401 GTTGCGTCCCACGAACTCAACCGTGCCATCGGCACGCCAGCGCCCCAGATCACCGCTGCG

5461 GTACATCCGCGCTGTTGGATCGGTGCTGAATGGATCCGTGAGGAAGCGCTCAGCGGTCAA

5521 TGCCTCTCGATTGAGATACCCACGCCCGACGCTATCGCCAGCGATATACAACTCACCCAC

5581 CACGCCGATGGGAACTGGGGCACCATTCGCATCCAAGATGTAGATACGCACATTGGCAAT

5641 CGGACGACCAATCGGTGGCGCGCCCATTGCGCTGGGATCGCATTCATGCATGCAGGCACA

5701 GACCGTCGCTTCCGTGGGTCCATATGCATTGATTAAGCGCCTTCCTTGACTCCAATATTT

5761 CGCGATGGTGGAAGAGAGCGAATCGCCCGCAACAACGATCGTTTGCAAAGAAACAAGTTC

5821 TTCAATGTTTTCTACTGTAG

LOCUS Contig G143 5242 bp DNA linear BCT 30-JAN-2012

DEFINITION Xanthomonas spp. strain XaS3 genomic region encoding NRPS.

SOURCE Xanthomonas spp. strain XaS3

ORGANISM Xanthomonas spp. strain XaS3

Bacteria; Proteobacteria; Gammaproteobacteria; Xanthomonadales;

Xanthomonadaceae; Xanthomonas.

REFERENCE Contig G143 (bases 1 to 5242)

AUTHORS Royer et al. Submitted to BMC Genomics.

TITLE Genome mining indicates that the genus Xanthomonas is a promising

reservoir for new bioactive non-ribosomally synthesized peptides

JOURNAL Submitted to BMC Genomics.

REFERENCE 2 (bases 1 to 5242)

FEATURES Location/Qualifiers

source 1.. 5242

/organism='Xanthomonas spp.'

/mol_type='other DNA'

/strain='XaS3'

/isolation_source='sugarcane leaf water droplets'

/host='Sugarcane'

/db_xref='taxon: GPE 39'

/country='Guadeloupe'

/collection_date='2003'

/collected_by='Rosiane BOISNE-NOC'

/note='[cultured bacterial source]'

gene complement (<1..5241>)

/gene ='NRPS'

CDS complement (<1..5241>)

/gene ='NRPS'

/note='incomplete NRPS, no start codon, no stop codon'

/codon_start=1

/translation='PFAEHPGQRMYRTGDLARWRSDGTLEFLGRNDAQVKIRGFRVEPAEVEAALRDCPGVREALVLTDTPRQGEHRLVAYVVGNASTPDTLRTRLSARLPAYMVPAAYVLLDALPITPNGKLDRRALPTPETDAFALQTYAAPEGELETLLANVWRELLDLEQVGRHDDFFALGGHSLLAVQLASRVRQRLGVAVGLTEIFAHPRLADLANALTLASVETLPAIVPSPHNGPLPLSFAQQRLWFLDTHGHSGSAYSLTYALQLRGPLDSAALHRALDRIVARHEVLRTRFAMVAGQPQQVIASADCGFALIRHDLSGESDPLQAAHAHADAQAQAPFDLENGPLLRGHLLRLGERDHVLLLTAHHIIADAWSGAVLVNELTALYAAFAQGMADPLPALPIQYADFALWQRRWIAGERLQGQLAHWLTHLRGAPTLLELPTDFPRPAQPDYRGEVVPFHLDAAQTAALKALARRHGATLFMVILAGWATLLARLSGQREVVIGTPIANRHHAELEPLIGLFVNSLALRIDLHQEQSVAALLAQTRATALAAQACQDIPFEHVVEALNPVRSNAHNPIFQVMFAWQNAPEGQLTLPQLTLETLQATHRNAQFDLELSMQESGDGIVGSLGFATALFARTRIQRHVQQLRTMLVRMATDDTSTLHALSAIAPQERALLQDFNATDTPIAAGVGVHALFAQQVQRTPTATALVDAERSLSYAELDAQANRLARHLIALGVGPDERVALYLSRRIDLVVAMLATLKAGGAYVPLDPTYPLQRVAFMLADSAPCVMLTDAQGAAQLRGHARTPVVLLDQAQPAWTQYPSTAPSVPALQAQHLAYVIYTSGSTGTPKGVAVEHGGLRNYCVAAATRYGLRSGDRVLQCSSPSFDIAVDEIFATLSSGATLVLLPSLRLPAIAEFVRVIAQQQINVLNLPTAYWHAWMAEHTATLPTCLRLVICGGEAPNPAHIAHWHALAQGQVPLLNAYGPTETVSGVSFGALAPGEPAHIGGPIANVRLHVLDRYRQPLPIGVNGELYIAGAQLARGYLGRADLTAERFVPDPFAPVAGARMYRSGDVARWREDGTLLFVGRDDHQIKLRGFRIEPSEIEAVLRTAADVQDAAVLLREDRPGERMLVAYVATPSLAVDAMRAHLATRLPDYMVPTRYVRLEALPLTPNGKLDRQALPAPESERSDPAATTTQGHYEQILAQVWCELLGIDRVERDDDFFDLGGHSLLAVQLIARVRDSFGVELQIGDVFTHTQLQALARHLEHSDPHARSDAASIVAVERGAALPLSFAQQRLWFLDRFDPNSQLAYLMPGGVRLRGPLDSLALRRALDRIVFRHEALRTHFAVEHDAPVQRIADAQTGFALQRIDLRALPDPEAAAHAHAEEEASTRLDLEHGPLVRGRLLRLADQDHLLLVTMHHIVSDGWSMGVLIRELGALYTAFAEGLSDPLPPLHVQYADYSSWQRRRISGEMLEQQRDFWRNHLQGAPALLELPTDHPRPARQDYAGDIVPFALDHVQSAALKQLGQRHGTTGYMTLLAAWAVLLSRLSGQSDVVIGTPVANRTHAELEPLIGFFVNTQALRVDLSRNPSVAELLSQVRATALAAQTHQDLPFEQLIETLNPDRNLAAHPLFQAMLSWENLPEAELQLPGLQLQGFGLPAQTIKFDLSMNVQEHDGSIVGTLGYATALFERDTIQRHVAQFLELLQAMVADDTQRVAQLPLLPPQERTQLQHMSQSAPAVDLPQASLH'

ORIGIN

1 GTGCAGGCTTGCCTGCGGCAGGTCCACTGCGGGCGCGCTTTGGCTCATGTGCTGGAGTTG

61 GGTGCGCTCTTGTGGCGGCAACAGCGGCAGTTGCGCGACGCGCTGCGTGTCGTCGGCGAC

121 CATGGCCTGCAGCAGTTCCAGGAACTGCGCGACATGGCGCTGGATCGTGTCGCGCTCGAA

181 CAGCGCGGTGGCATAGCCGAGCGTACCGACGATGCTGCCATCGTGTTCCTGCACGTTCAT

241 GCTCAGGTCGAACTTGATCGTCTGTGCTGGCAGGCCGAATCCCTGCAATTGCAGGCCGGG

301 AAGCTGCAGTTCCGCCTCCGGCAGGTTTTCCCAACTCAACATCGCCTGGAACAGCGGGTG

361 TGCGGCGAGGTTGCGGTCGGGGTTGAGTGTTTCGATGAGTTGCTCGAACGGGAGATCCTG

421 GTGGGTTTGCGCCGCCAGGGCGGTCGCGCGCACCTGCGACAAAAGTTCTGCCACGCTCGG

481 GTTGCGCGACAGATCCACGCGCAGGGCCTGGGTGTTGACGAAGAAGCCGATCAGCGGTTC

541 CAGTTCGGCATGGGTGCGGTTGGCCACCGGCGTGCCGATCACGACATCGGATTGGCCGGA

601 CAGGCGCGACAGCAGCACCGCCCAGGCGGCCAGCAGGGTCATGTACCCGGTGGTGCCATG

661 ACGTTGGCCGAGTTGCTTGAGCGCTGCGCTTTGCACATGATCCAGCGCGAATGGGACGAT

721 GTCGCCGGCATAGTCCTGGCGTGCCGGGCGAGGGTGGTCGGTCGGCAATTCCAGCAGCGC

781 CGGCGCGCCTTGCAGGTGATTGCGCCAGAAGTCGCGTTGTTGTTCCAGCATCTCGCCGCT

841 GATCCGGCGACGTTGCCAACTGCTGTAGTCGGCGTACTGCACGTGTAGCGGTGGCAGCGG

901 GTCGCTGAGGCCTTCGGCGAACGCGGTGTACAGGGCGCCCAGTTCGCGGATCAGCACGCC

961 CATCGACCAACCGTCGGAAACGATGTGATGCATCGTCACCAGCAGCAGATGGTCCTGGTC

1021 GGCCAGGCGTAGCAGCCGGCCACGCACCAAGGGGCCATGTTCCAGGTCCAAACGGGTGCT

1081 GGCTTCTTCTTCGGCGTGTGCGTGCGCCGCCGCTTCTGGATCGGGCAGGGCGCGCAGGTC

1141 GATACGTTGCAGGGCAAAGCCGGTCTGCGCATCGGCGATGCGCTGGACCGGGGCGTCGTG

1201 TTCCACCGCGAAGTGGGTCCGCAGGGCTTCGTGCCGGAACACGATACGGTCCAGGGCGCG

1261 ACGCAATGCCAGGCTGTCCAGCGGGCCGCGCAAGCGCACGCCACCGGGCATCAAATAGGC

1321 GAGCTGGCTATTGGGGTCGAAGCGGTCCAGGAACCAAAGCCGCTGCTGCGCGAAGGACAG

1381 CGGCAGCGCCGCGCCGCGTTCCACCGCGACGATCGACGCGGCATCGCTACGCGCATGAGG

1441 GTCGCTGTGCTCCAGGTGTCGTGCCAGTGCCTGCAATTGGGTGTGGGTGAACACATCGCC

1501 GATCTGCAATTCCACGCCGAAGCTGTCGCGGACGCGTGCGATCAATTGCACCGCCAGCAG

1561 CGAGTGTCCGCCCAGGTCGAAGAAATCGTCGTCGCGCTCGACCCGGTCGATTCCGAGTAA

1621 CTCGCACCAGACCTGCGCCAGGATTTGCTCGTAGTGGCCTTGCGTCGTCGTCGCGGCGGG

1681 ATCGCTGCGCTCGCTCTCCGGCGCCGGCAGCGCCTGGCGGTCGAGTTTGCCGTTGGGCGT

1741 GAGCGGCAGCGCCTCCAGTCGCACGTAACGGGTCGGTACCATGTAGTCCGGCAGCCGTGT

1801 CGCCAGATGCGCGCGCATGGCATCCACCGCCAGCGACGGCGTCGCCACATAGGCGACCAG

1861 CATGCGTTCGCCGGGCCGGTCTTCGCGCAGCAGCACCGCCGCATCCTGCACGTCTGCCGC

1921 CGTGCGTAGCACGGCTTCGATTTCGCTCGGCTCGATGCGGAAACCGCGCAGTTTGATTTG

1981 ATGGTCGTCGCGACCGACGAACAGTAGCGTGCCATCCTCGCGCCAACGCGCCACATCGCC

2041 GCTGCGGTACATGCGTGCGCCGGCCACCGGTGCGAACGGATCGGGGACGAAGCGTTCGGC

2101 GGTCAGATCCGCACGGCCCAGATATCCGCGCGCCAACTGCGCACCGGCGATATACAGTTC

2161 GCCGTTGACGCCGATGGGGAGCGGTTGCCGGTACCGATCGAGCACGTGCAGGCGCACATT

2221 GGCAATCGGTCCGCCGATATGTGCTGGCTCACCGGGCGCCAACGCGCCGAAGCTGACCCC

2281 GGAGACCGTCTCGGTCGGGCCATAGGCGTTGAGCAGTGGCACCTGTCCCTGCGCTAGCGC

2341 ATGCCAGTGTGCGATGTGCGCCGGGTTCGGCGCTTCGCCGCCGCAGATCACCAGTCGCAG

2401 GCACGTCGGCAGCGTCGCGGTGTGCTCGGCCATCCACGCATGCCAGTAGGCGGTGGGTAG

2461 ATTGAGCACGTTGATCTGTTGCTGCGCGATCACCCGCACGAATTCGGCGATGGCCGGCAG

2521 GCGCAGGCTGGGCAGCAGGACCAGGGTGGCGCCGCTGGAGAGCGTGGCGAAGATTTCGTC

2581 CACGGCGATATCGAAGCTGGGCGAACTGCATTGCAGCACCCGGTCGCCGCTGCGCAGGCC

2641 ATAGCGGGTGGCGGCGGCCACGCAATAGTTGCGCAGTCCGCCATGCTCCACCGCCACGCC

2701 CTTGGGCGTGCCGGTCGAGCCTGACGTGTAGATCACATACGCCAGATGCTGTGCTTGCAG

2761 CGCCGGCACGCTGGGCGCGGTGGAAGGGTACTGTGTCCAGGCCGGTTGCGCCTGATCCAG

2821 CAACACCACGGGTGTCCGGGCGTGTCCACGCAACTGGGCCGCGCCTTGTGCATCGGTCAG

2881 CATCACGCACGGCGCGCTATCGGCGAGCATGAACGCCACGCGCTGCAGTGGGTAGGTCGG

2941 ATCCAGCGGCACATAGGCGCCGCCGGCTTTCAGCGTCGCCAACATCGCCACGACCAGGTC

3001 GATGCGACGGCTCAGATACAGAGCCACCCGCTCATCCGGGCCAACGCCCAGTGCGATCAG

3061 GTGCCGCGCCAGACGATTGGCCTGGGCATCGAGTTCGGCATAAGAGAGGCTGCGCTCGGC

3121 ATCCACCAGGGCAGTGGCCGTCGGTGTGCGCTGGACCTGTTGCGCGAACAGCGCATGCAC

3181 ACCCACGCCAGCGGCGATCGGCGTGTCGGTGGCATTGAAGTCCTGCAGCAGCGCTCGCTC

3241 TTGTGGCGCGATGGCCGACAGCGCGTGGAGCGTGCTGGTGTCGTCGGTGGCCATCCGCAC

3301 CAGCATGGTTCGCAACTGCTGCACATGGCGTTGGATGCGCGTGCGCGCGAATAACGCCGT

3361 GGCGAAACCCAGACTGCCGACGATGCCATCGCCGCTTTCCTGCATCGACAGTTCCAGATC

3421 GAACTGCGCGTTGCGATGGGTGGCTTGCAAGGTTTCCAGCGTCAGTTGCGGCAGCGTGAG

3481 TTGACCTTCCGGCGCGTTCTGCCAGGCGAACATCACCTGGAAGATCGGGTTGTGCGCGTT

3541 GCTGCGCACCGGATTGAGTGCTTCGACCACATGTTCGAACGGAATGTCCTGGCATGCCTG

3601 CGCCGCCAGTGCCGTTGCCCGGGTCTGCGCAAGCAGCGCGGCCACGCTCTGCTCCTGGTG

3661 CAGATCGATGCGCAGCGCGAGCGAATTGACGAACAGACCGATCAACGGCTCCAGTTCGGC

3721 ATGATGGCGATTGGCGATCGGCGTGCCGATCACCACCTCGCGCTGGCCGGACAGGCGTGC

3781 CAGCAAGGTCGCCCAGCCGGCCAGGATCACCATGAACAACGTGGCGCCGTGGCGTCGGGC

3841 CAGTGCTTTGAGTGCGGCCGTTTGCGCGGCATCCAGGTGGAATGGCACCACTTCGCCGCG

3901 ATAGTCCGGCTGCGCCGGGCGTGGGAAGTCGGTCGGTAATTCGAGCAGTGTTGGAGCGCC

3961 GCGTAGATGCGTGAGCCAATGCGCCAGTTGACCCTGCAAACGCTCGCCGGCGATCCAGCG

4021 CCGTTGCCAGAGGGCGAAGTCGGCGTACTGGATCGGCAGCGCGGGCAACGGGTCGGCCAT

4081 TCCCTGCGCGAACGCTGCGTACAGGGCGGTCAGCTCGTTGACCAGGACCGCGCCGGACCA

4141 GGCATCGGCAATGATGTGATGGGCGGTCAACAGCAAGACATGGTCGCGCTCGCCCAGGCG

4201 TAGCAGGTGACCGCGCAGCAACGGCCCGTTTTCCAGATCGAACGGCGCCTGCGCTTGCGC

4261 ATCGGCGTGCGCGTGCGCGGCTTGCAACGGATCGCTTTCTCCACTGAGATCGTGCCGGAT

4321 CAGCGCAAATCCGCAATCGGCCGAGGCAATCACCTGCTGCGGTTGTCCCGCCACCATCGC

4381 GAAGCGGGTACGTAGCACCTCGTGGCGTGCGACGATCCGGTCTAGCGCGCGGTGCAAGGC

4441 GGCGCTGTCGAGCGGCCCGCGCAGTTGCAATGCGTAGGTGAGCGAGTAGGCGCTGCCGCT

4501 GTGGCCGTGCGTGTCCAGGAACCACAGTCGCTGCTGCGCGAAGGACAGCGGCAGTGGTCC

4561 GTTGTGCGGACTGGGTACGATGGCCGGTAGGGTCTCGACCGAGGCCAGGGTCAAGGCATT

4621 GGCCAGATCGGCCAGGCGCGGGTGCGCGAAGATCTCGGTCAGGCCGACGGCCACGCCGAG

4681 CCGTTGCCGCACGCGCGAGGCCAACTGCACGGCCAGCAGCGAGTGTCCACCGAGGGCGAA

4741 GAAGTCGTCGTGGCGACCGACCTGTTCGAGGTCGAGCAATTCGCGCCACACATTGGCGAG

4801 CAGGGTTTCCAGTTCGCCTTCGGGTGCGGCGTAGGTCTGCAGGGCGAATGCATCCGTCTC

4861 CGGCGTCGGCAGTGCCCGGCGGTCGAGCTTACCGTTGGGTGTGATCGGCAGTGCATCGAG

4921 CAGGACATAGGCGGCCGGCACCATGTAGGCCGGCAGTCGCGCACTCAGCCGGGTGCGCAG

4981 GGTGTCGGGAGTGCTCGCGTTGCCGACGACATAGGCCACCAGGCGATGTTCGCCCTGGCG

5041 TGGTGTGTCGGTCAGTACCAGCGCTTCGCGCACGCCGGGACAGTCGCGCAGCGCCGCTTC

5101 GACCTCGGCCGGCTCTACGCGGAAGCCCCGGATCTTGACCTGCGCGTCGTTGCGGCCGAG

5161 GAATTCCAGTGTGCCGTCGCTGCGCCAGCGCGCCAGGTCGCCGGTGCGGTACATGCGCTG

5221 GCCGGGGTGTTCGGCGAACGGA

LOCUS Contig G146 4673 bp DNA linear BCT 30-JAN-2012

DEFINITION Xanthomonas spp. strain XaS3 genomic region encoding NRPS.

SOURCE Xanthomonas spp. strain XaS3

ORGANISM Xanthomonas spp. strain XaS3

Bacteria; Proteobacteria; Gammaproteobacteria; Xanthomonadales;

Xanthomonadaceae; Xanthomonas.

REFERENCE Contig G146 (bases 1 to 4673)

AUTHORS Royer et al. Submitted to BMC Genomics.

TITLE Genome mining indicates that the genus Xanthomonas is a promising

reservoir for new bioactive non-ribosomally synthesized peptides

JOURNAL Submitted to BMC Genomics.

REFERENCE 2 (bases 1 to 4673)

FEATURES Location/Qualifiers

source 1.. 4673

/organism='Xanthomonas spp.'

/mol_type='other DNA'

/strain='XaS3'

/isolation_source='sugarcane leaf water droplets'

/host='Sugarcane'

/db_xref='taxon: GPE 39'

/country='Guadeloupe'

/collection_date='2003'

/collected_by='Rosiane BOISNE-NOC'

/note='[cultured bacterial source]'

gene complement (<2..4673>)

/gene ='NRPS'

CDS complement (<2..4673>)

/gene ='NRPS'

/note='incomplete NRPS, no start codon, no stop codon'

/codon_start=1

/translation='EVVNTYGPTETTVSCTAEHLRQDAECDLSVTIGQPLANVRIYILDTHSVPVPIGVVGELYIGGDGVGRGYLNRDDLTAARFLADPFSADPTARMYRTGDLGRWRVDGTIEFVGRNDQQVKIRGFRIELGEIEARLSAHAEVRECVVVALEGAAGTDKRLVAYWVGNEAVDAAALRTWLSDVLPDYMVPAAYVQLDRLPLTPNGKLDRKALPAPDGAAYAALTYEAPQGEIEQAIAAIWCELLGLEDIGRHDNFFALGGHSLLAVRVTSRLRQEMGVEIGVADLFVHATPQQLATCVASSSSTVLSPILPLESDAPRVLSFAQQRLWFLSQFEGVSQAYHISGGLRLRGALDRRTLQRALDRIVARHASLRTAFLMLDGQVQQQIAAEDIGFHLIDHDLRGVPDREAALQQLLADEVQAPFALERGPLIRGRLIQLADDESVLFVTMHHIVSDGWSMGILINELSVLYRAFARGEADPLPPLPIQYADYASWQRQWLAGDVLQQQAAYWRDALSGAPELLELPTDRPRPTRQDHACAMLEVVVGSQQAQALKELSQRHGLTLYMTLLASWALLLSRLSGQDDVVIGSPVANRGRSETEGLIGFFVNTLALRIELSGSPTLAQLLASVKQRALQAQAHQDIPFEQVVELVQPPRSLAYTPLFQVMFAWQNMPRSELDLGDIEVSGLGIAQTREQFDLLLSLAEGEQGIVGSLNYATALFEHSTLQRWMGHWRHLLEAMVAEGAEDQPVDRLPLLNEVERHQLLRQWNATATDYPRDACVHELFEAQVAQTPSAIAVVQGEVSLTYGELNARANRLAHYLRDLGVRPDDRVAICVQRSVEMVVAVVAVLKAGGAYVPLDPAYPPERLAYVRADCGAIVVLTDAASRHLIEESATAAVIVDLQADGERWQHLPDRNPGRHANGLTARHLAYVIYTSGSTGMPKGAMNEHRGVVNMACAQKRKLLLNSNSRILQFASFSFDSCTFEIMIAICWGGSLFISKNNGIIIGDDFVEIVKDKEITHAVVPPAFLSTVLNVEEIFSLQTIISAGDVLSVPIAKRWSQGRRLINGYGPTETTVCACLHECHVDAVGAPPIGRPVDNVRIYILDQNGAPVPIGVAGELYIGGDGVGRGYLNRDDLTAARFLTDPFSTDPTARMYRTGDLGRWRVDGTIEFVGRNDHQVKIRGFRIELGEIEARLSAHADVRECVVVALEGAAGTDKRLVAYWVGHEAVDAAALRSWLSDVLPDYMVPAAYVRLDRLPLTPNGKLDRKSLPAPDAAAYAALTYEAPQGEIEQAIAAIWSDLLGLEKIGRQDNFFALGGHSLLGVRLISRIRSALGLELPLATLFAQPRLAELAQALDNAAASTLPAIVPADRSAPLPLSFAQQRLWFLAQFDSRAAQAYTLYGGVDLHGVLDLPALKQALDRIVARHEVLRTCFVASDDGAIQVIAPADVGFALTCIDLRHTPDAEVAAQHHAEQETHTPFDLSRGPLIRGCLLQLAEQQHRLLISMHHSISDGWSIGILIRELGALYAAFVQGQPNPLPPLPIQYADYSLWQRRWLDGP'

ORIGIN

1 GCGGCCCATCGAGCCAGCGGCGTTGCCACAGGCTGTAGTCGGCGTATTGGATCGGCAGCG

61 GCGGCAGCGGATTGGGCTGGCCTTGCACGAAGGCGGCGTACAACGCGCCAAGTTCGCGAA

121 TCAAGATCCCAATGGACCAGCCATCGGAGATGCTGTGGTGCATGCTGATCAGCAGACGGT

181 GTTGCTGCTCGGCCAGCTGCAGCAGGCAGCCACGGATCAATGGACCACGGCTCAGATCGA

241 AGGGCGTGTGGGTTTCCTGTTCGGCGTGATGCTGCGCGGCAACCTCGGCATCTGGCGTAT

301 GACGCAGATCGATGCAGGTCAGCGCGAAGCCGACATCCGCCGGAGCAATCACCTGGATAG

361 CGCCATCATCGCTGGCGACGAAGCAGGTGCGCAGTACCTCGTGGCGGGCGACGATACGAT

421 CCAATGCCTGCTTGAGTGCGGGCAAATCCAGCACCCCGTGCAGGTCCACGCCGCCATAAA

481 GGGTGTAAGCCTGTGCGGCACGGCTGTCGAATTGCGCCAGGAACCATAGTCGTTGCTGTG

541 CGAAGGATAGCGGCAGCGGAGCGCTGCGGTCAGCTGGCACGATGGCCGGCAGCGTGCTGG

601 CGGCGGCGTTGTCCAGGGCCTGTGCCAACTCGGCCAAACGTGGTTGAGCAAACAACGTGG

661 CCAGCGGCAGTTCCAAGCCCAGCGCGCTGCGGATGCGCGAGATCAAGCGCACGCCCAGCA

721 GCGAATGCCCGCCCAGGGCGAAGAAGTTGTCTTGTCGACCGATCTTTTCCAGACCTAAGA

781 GGTCACTCCAGATCGCGGCAATGGCCTGTTCGATCTCGCCCTGTGGTGCTTCATACGTCA

841 ACGCCGCATAGGCCGCTGCATCCGGTGCGGGTAATGACTTGCGATCCAGCTTGCCGTTGG

901 GAGTCAATGGCAGCCGATCCAACCGCACATAGGCGGCTGGCACCATGTAATCCGGCAGTA

961 CATCCGATAACCAACTGCGCAATGCAGCAGCATCAACAGCTTCGTGACCGACCCAATACG

1021 CCACCAGCCGCTTATCAGTGCCTGCCGCACCCTCCAGCGCCACCACGACGCACTCGCGCA

1081 CATCCGCATGTGCGCTCAAGCGTGCCTCGATCTCACCCAACTCGATGCGGAAACCACGAA

1141 TCTTGACCTGGTGATCGTTGCGTCCGACAAACTCAATCGTGCCATCGACACGCCAACGGC

1201 CCAGATCACCCGTGCGGTACATCCGTGCAGTCGGATCGGTGCTGAACGGATCCGTGAGGA

1261 AGCGCGCGGCGGTCAAATCATCGCGATTCAGATACCCACGCCCCACGCCATCGCCACCGA

1321 TATACAGCTCTCCCGCCACTCCGATCGGAACCGGCGCACCGTTTTGATCGAGGATATAGA

1381 TCCGCACATTATCAACAGGGCGCCCAATCGGTGGCGCACCCACCGCGTCGACATGACATT

1441 CATGCAAACAGGCACAGACCGTCGTTTCCGTAGGTCCATATCCATTGATCAACCGTCTTC

1501 CTTGACTCCAGCGTTTCGCGATAGGAACAGAGAGCACATCACCTGCAGAAATGATCGTCT

1561 GCAAAGAGAAAATTTCTTCAACATTGAGCACGGTAGATAAAAATGCGGGAGGCACCACCG

1621 CATGAGTAATTTCTTTATCCTTTACTATCTCAACAAAATCATCACCAATAATAATTCCAT

1681 TATTTTTTGATATAAATAGTGACCCACCCCAGCAGATCGCTATCATAATTTCGAACGTAC

1741 AAGAATCGAAACTAAAAGAAGCAAACTGCAAGATACGGCTATTAGAATTTAACAACAATT

1801 TCCGTTTCTGAGCACAAGCCATGTTGACGACGCCGCGATGCTCGTTCATCGCGCCCTTCG

1861 GCATCCCCGTCGATCCAGACGTATAAATCACATACGCCAGGTGACGTGCGGTCAGACCAT

1921 TGGCATGGCGGCCGGGATTTCGGTCTGGCAAATGTTGCCAGCGCTCGCCGTCGGCTTGCA

1981 GATCGACGATCACCGCCGCAGTGGCACTCTCTTCGATCAGGTGACGGCTGGCAGCATCCG

2041 TCAGCACGACGATCGCGCCGCAATCAGCGCGCACGTAGGCCAACCGCTCTGGCGGATAGG

2101 CCGGATCCAGAGGTACATACGCACCACCAGCCTTCAGTACCGCCACAACCGCCACCACCA

2161 TCTCCACACTACGCTGTACGCAGATCGCCACGCGATCATCCGGGCGCACACCCAAATCAC

2221 GCAGGTAATGCGCTAGACGGTTAGCCCGCGCATTCAACTCGCCATACGTCAGTGAAACCT

2281 CGCCTTGCACCACCGCGATGGCCGATGGTGTCTGTGCCACCTGTGCCTCGAACAGTTCGT

2341 GCACACACGCATCGCGTGGGTAATCCGTTGCAGTTGCATTCCACTGCCTCAGTAGCTGAT

2401 GGCGCTCGACCTCGTTCAGCAGTGGCAGACGATCCACCGGCTGATCTTCGGCCCCCTCGG

2461 CGACCATCGCTTCCAGTAGGTGGCGCCAATGCCCCATCCAACGCTGTAGCGTCGAATGCT

2521 CGAACAGCGCTGTTGCATAGTTCAAACTGCCGACAATCCCTTGCTCGCCTTCGGCCAACG

2581 ACAGCAATAGATCGAACTGCTCCCTGGTTTGCGCAATACCCAACCCGCTGACCTCGATAT

2641 CGCCAAGATCCAGTTCGCTCCGTGGCATGTTCTGCCACGCGAACATCACCTGGAACAGTG

2701 GCGTATACGCCAGGCTGCGTGGCGGTTGTACCAACTCCACCACCTGCTCGAACGGGATGT

2761 CCTGATGCGCCTGCGCCTGCAGTGCGCGCTGCTTCACCGATGCCAGCAGTTGCGCCAGCG

2821 TCGGCGAGCCGGACAGCTCAATCCGTAACGCCAGCGTGTTGACGAAGAAGCCGATTAGCC

2881 CTTCGGTCTCCGATCTCCCGCGATTGGCCACCGGACTGCCGATCACCACATCGTCCTGGC

2941 CGGAAAGACGCGACAGCAGCAGCGCCCAACTGGCGAGTAGCGTCATATACAGCGTCAACC

3001 CGTGACGTTGGCTCAGTTCTTTCAGCGCTTGTGCCTGCTGCGAACCGACGACCACCTCCA

3061 GCATCGCACAGGCATGGTCTTGCCGGGTTGGACGCGGACGATCCGTTGGCAATTCCAGCA

3121 GCTCTGGCGCACCGGACAGTGCATCGCGCCAGTACGCCGCCTGCTGCTGCAACACGTCAC

3181 CCGCCAGCCATTGCCGCTGCCAGCTTGCATAATCGGCGTATTGAATCGGCAATGGTGGCA

3241 GCGGATCGGCTTCGCCACGTGCGAACGCTCGATACAACACGCTCAACTCGTTGATCAAAA

3301 TCCCCATCGACCACCCGTCCGAGACGATGTGGTGCATGGTCACGAACAGGACGGATTCGT

3361 CGTCGGCCAGTTGTATCAGCCGACCACGGATCAACGGGCCTCGCTCCAATGCAAATGGTG

3421 CTTGCACCTCATCGGCTAATAGTTGCTGCAATGCCGCCTCGCGATCCGGAACACCGCGAA

3481 GATCATGGTCGATCAAATGGAAGCCAATGTCTTCGGCGGCAATCTGCTGCTGCACCTGCC

3541 CGTCGAGCATTAAGAAGGCCGTGCGCAACGACGCATGCCGGGCTACGATCCGATCCAAAG

3601 CACGCTGCAATGTCCGCCTATCCAGCGCTCCGCGCAAGCGCAGTCCACCGCTGATGTGAT

3661 ACGCCTGACTGACGCCTTCGAACTGCGATAGGAACCAAAGCCGCTGCTGCGCAAACGAGA

3721 GCACGCGCGGCGCATCTGACTCCAGAGGCAAGATCGGTGATAAGACAGTGCTGGACGAAG

3781 ATGCCACACAGGTCGCAAGTTGCTGGGGCGTGGCGTGTACGAACAGGTCCGCCACGCCGA

3841 TCTCGACCCCCATTTCTTGACGCAAGCGGGAGGTGACCCGCACCGCCAGCAACGAATGCC

3901 CGCCAAGGGCAAAGAAGTTGTCGTGCCGACCGATGTCTTCCAGACCGAGTAACTCGCACC

3961 AGATCGCGGCAATGGCTTGTTCAATCTCGCCCTGTGGTGCTTCGTACGTCAACGCCGCAT

4021 AGGCCGCGCCATCCGGTGCGGGTAACGCCTTGCGATCCAGCTTGCCGTTGGGGGTGAGTG

4081 GCAAGCGATCCAACTGCACATAGGCGGCTGGCACCATGTAATCCGGCAGTACATCCGATA

4141 GCCAAGTCCGCAATGCGGCAGCATCAACAGCTTCGTTACCGACCCAATACGCCACCAGCC

4201 GCTTATCAGTGCCTGCCGCACCCTCCAGCGCCACCACGACGCACTCACGCACCTCCGCAT

4261 GCGCGCTCAAGCGTGCCTCGATCTCACCCAACTCGATGCGGAAACCACGAATCTTGACCT

4321 GCTGATCGTTGCGTCCTACAAACTCAATCGTGCCATCGACACGCCAACGGCCCAGATCGC

4381 CCGTGCGGTACATCCGTGCAGTCGGATCGGCGCTAAACGGATCAGCGAGGAAGCGCGCGG

4441 CGGTCAAATCATCGCGATTCAGATACCCACGCCCCACGCCATCGCCGCCGATATACAACT

4501 CGCCCACTACCCCAATCGGCACCGGCACACTGTGTGTATCCAGGATGTAGATACGCACAT

4561 TGGCGAGAGGCTGCCCGATGGTGACTGACAAATCGCATTCGGCATCTTGCCTCAGATGTT

4621 CCGCAGTACAAGAAACCGTGGTCTCCGTCGGTCCATAGGTATTGACAACCTCT

LOCUS Contig G147 4580 bp DNA linear BCT 30-JAN-2012

DEFINITION Xanthomonas spp. strain XaS3 genomic region encoding NRPS.

SOURCE Xanthomonas spp. strain XaS3

ORGANISM Xanthomonas spp. strain XaS3

Bacteria; Proteobacteria; Gammaproteobacteria; Xanthomonadales;

Xanthomonadaceae; Xanthomonas.

REFERENCE Contig G147 (bases 1 to 4580)

AUTHORS Royer et al. Submitted to BMC Genomics.

TITLE Genome mining indicates that the genus Xanthomonas is a promising

reservoir for new bioactive non-ribosomally synthesized peptides

JOURNAL Submitted to BMC Genomics.

REFERENCE 2 (bases 1 to 4580)

FEATURES Location/Qualifiers

source 1.. 4580

/organism='Xanthomonas spp.'

/mol_type='other DNA'

/strain='XaS3'

/isolation_source='sugarcane leaf water droplets'

/host='Sugarcane'

/db_xref='taxon: GPE 39'

/country='Guadeloupe'

/collection_date='2003'

/collected_by='Rosiane BOISNE-NOC'

/note='[cultured bacterial source]'

gene <2..4579>

/gene ='NRPS'

CDS <2..4579>

/gene ='NRPS'

/note='incomplete NRPS, no start codon, no stop codon'

/codon_start=1

/translation='LDDAERHQVLTQWNATKADYPRDACVHALFEAQVARDPSAIAIVQNNVALTYGELNARANRLAHYLRELGVCPDDRVAVCVQRSVEMVVALLAVLKAGGAYVPLDPDYPPERLAYILADCGLAQVLTVRDAILPEPLELLRVNIDDAVVSAAQLHNPGVHSHGGTSACVIYTSGSTGLPKGVEIPHRGINRLVLNNGYLPFSPSDCVALAANPSFDATTFEVWGALLNGARLVVIEADVLLNPVRLAETVECAGISILLLAAGLFHQYAESMKKRFGHLRYLLAGGDAIDPSVVAKVLDGNGPQHFLNCYGPTEITTIATAYEAVEIDEDVRSLPIGRPIANTQIYILDAHGAPVPIGVVGELYIGGDGVGLGYLNREDLTAERFLPDPFSADPAARMYRSGDLGRWRADGTIEFFGRNDHQVKIRGFRIELGEIEARLSAHADVRECVVMALEGAVAGTEKRLVAYWVAAEHVTSEPLGAESLRSWLSDTLPDYMVPAAYVQLDRLPLTPNGKLDRKALPAPDGSAYAAPAYEAPQGAIEQTIAAIWCDLLGLESIGRHDNFFALGGHSLLAVRVASRLRQELGVEIGVAELFAHATLKDLAACVTSSSGAILPPILPLQPDAPRVLSFAQQRLWFLSQFEGVSQAYHISGGLRLRGALDTKALQRALDRIVARHASLRTTFALVDGQALQQIAAEESGFHLIAHELCGVPDGEEALEKLLTEEAQAPFALEQGPLIRGRLIRLADDESVLFVTMHHIVSDGWSMGVLINELSVLYRAFARGEADPLAPLPIQYADYASWQRQWLTGDVLEQQATYWREAMSGAPVLLELPTDRPRPAQQDHAGAMLEVIVDPQQAQALKALSQRHGLTLYMTLLASWALLLSRLSGQDDVVIGSPVANRGRSETEGLIGFFVNTLALRVELSGSPTLAQLLASVKSRTLQAQAHQDIPFEQVVELIQPPRSLAHTPLFQVMFAWQNTPQGELDLGAIEASGLGVAQTSAQFDLSLSLMESDEGIVGSLAYATALFERSTLERWMGHWRHLLEAMVADGAEHQAVDRLPLLDDAERHQVLTQWNETAADYPRDACVHELFEAQVARTPSAIAVVQGEVSLTYGELNARANRLAHYLRELGVCPDDRVAICVQRSVEMVVAVLAVLKAGGAYVPLDPAYPVERLTYMLEDSAPVAVLAQTLTSNLLSNLLLTTSAPIINLDESHWQDRSVSNLSMDGLTSAHLAYVIYTSGSTGMPKGVMIEHRNTVNLLAWAQRSFEPSVLAKTLFSTSLNFDLAVYECFAPLICGGAIEVVDNLLALQTGQHDITLINTVPSALKALLESGGLGRSVQTVNVAGEALKRQLVEDIFAKTQVERLCNLYGPSETTTYSSWVSMERSDGFVSHIGTPLDNTQFYVLDTHRQPVPVGVTGELYIGGTGVARGYLNRGDLTAARFLMDPFSADPTARMYRTGDLGRWRADGTLEFVGRNDHQVKIRGFRIELGEIEARLSAHVDVRECVVVALEDATGSDKRLVAYWV'

ORIGIN

1 GCTGGACGATGCCGAGCGCCATCAGGTGCTGACGCAGTGGAATGCGACCAAGGCGGATTA

61 TCCTCGCGATGCTTGTGTGCACGCATTGTTCGAGGCACAGGTGGCGCGTGATCCATCGGC

121 AATTGCGATTGTTCAAAACAATGTCGCGCTGACGTATGGTGAGTTGAATGCACGCGCCAA

181 TCGTCTGGCGCATTACCTACGCGAATTGGGCGTGTGCCCGGATGACCGCGTGGCAGTGTG

241 CGTGCAGCGTAGCGTCGAGATGGTGGTGGCGTTGCTGGCGGTGTTGAAGGCGGGCGGTGC

301 GTATGTGCCGCTGGATCCGGACTATCCGCCGGAGCGTCTGGCTTACATCCTCGCTGATTG

361 CGGCCTCGCTCAGGTGTTGACGGTGAGGGATGCGATCTTGCCAGAGCCGCTGGAGCTCTT

421 GCGGGTCAACATCGACGATGCTGTGGTGAGCGCAGCGCAGTTGCATAATCCTGGAGTGCA

481 CTCGCATGGTGGCACGAGCGCTTGTGTCATCTATACCTCCGGTTCGACAGGACTGCCGAA

541 GGGAGTCGAAATCCCGCATCGAGGGATCAATCGTCTGGTATTGAACAATGGGTATCTTCC

601 CTTCAGTCCGAGCGACTGTGTCGCTTTGGCGGCCAATCCGAGTTTCGATGCGACGACGTT

661 CGAAGTCTGGGGTGCGCTGCTCAATGGCGCACGCTTGGTCGTCATCGAAGCGGATGTTTT

721 GCTGAACCCTGTCAGGTTGGCGGAGACAGTCGAGTGCGCAGGCATCAGCATCTTGCTGTT

781 AGCGGCGGGGTTGTTCCATCAATATGCTGAGAGCATGAAAAAGAGGTTCGGTCACTTGCG

841 TTATCTGTTGGCCGGTGGTGATGCGATTGATCCAAGCGTGGTGGCGAAGGTCTTGGATGG

901 CAACGGCCCACAGCATTTCTTGAATTGCTATGGCCCGACGGAAATCACGACCATCGCGAC

961 GGCCTATGAGGCGGTGGAAATCGATGAGGATGTCAGAAGTCTTCCAATCGGCAGGCCAAT

1021 CGCCAACACGCAGATCTACATTCTTGATGCACACGGTGCGCCGGTTCCGATTGGGGTGGT

1081 GGGCGAGTTGTATATCGGTGGCGATGGCGTGGGTCTTGGATATTTGAATCGAGAGGACTT

1141 GACCGCCGAACGCTTCCTGCCGGATCCGTTTAGTGCCGATCCGGCAGCGCGGATGTATCG

1201 CAGTGGCGATCTGGGGCGTTGGCGTGCCGATGGCACGATTGAGTTTTTCGGGCGCAACGA

1261 TCATCAGGTCAAGATCCGTGGTTTCCGCATCGAATTGGGTGAAATCGAGGCACGGCTGAG

1321 TGCGCATGCGGATGTGCGCGAATGCGTGGTGATGGCGCTGGAAGGTGCGGTGGCTGGTAC

1381 CGAGAAGCGTCTGGTGGCGTATTGGGTCGCTGCCGAGCATGTGACGTCCGAGCCTCTTGG

1441 CGCGGAAAGCTTGCGCAGTTGGTTGTCGGATACCTTGCCGGATTACATGGTGCCGGCGGC

1501 TTATGTGCAGTTGGATCGCTTGCCGCTGACTCCGAACGGCAAGTTGGATCGCAAGGCATT

1561 ACCCGCACCGGATGGCTCGGCCTATGCAGCGCCTGCGTATGAAGCACCGCAGGGCGCGAT

1621 CGAACAAACCATTGCCGCGATTTGGTGTGACCTGCTGGGTCTGGAAAGTATCGGGCGACA

1681 CGATAACTTCTTCGCACTCGGTGGACATTCGTTGTTGGCGGTGCGAGTTGCTTCGCGCCT

1741 GCGTCAGGAATTGGGTGTCGAGATCGGCGTGGCGGAGTTGTTCGCGCATGCAACGCTGAA

1801 GGACCTTGCCGCTTGCGTGACCTCTTCGTCCGGTGCGATCTTGCCGCCGATCCTGCCATT

1861 GCAGCCGGATGCGCCGCGCGTGCTGTCCTTTGCACAGCAACGGCTCTGGTTCCTATCGCA

1921 GTTCGAGGGTGTCAGTCAGGCGTATCACATCAGCGGTGGCTTGCGTTTACGCGGGGCGCT

1981 GGATACGAAAGCGTTGCAACGTGCGTTAGATCGCATCGTGGCCCGGCATGCGTCGTTGCG

2041 CACGACCTTCGCGCTGGTGGATGGGCAGGCGTTGCAGCAAATCGCCGCTGAAGAAAGCGG

2101 CTTCCATCTGATCGCCCACGAGCTGTGTGGGGTACCTGATGGTGAGGAAGCGCTTGAGAA

2161 ACTCCTGACCGAGGAAGCGCAAGCGCCATTTGCGTTGGAGCAAGGCCCACTCATCCGTGG

2221 CCGGCTGATACGACTTGCCGATGACGAGTCCGTCCTCTTCGTCACGATGCACCACATCGT

2281 CTCGGATGGTTGGTCGATGGGAGTTTTGATCAATGAGCTGAGCGTGTTGTATCGGGCGTT

2341 TGCACGTGGCGAAGCCGATCCGCTAGCACCGTTGCCGATCCAGTACGCCGACTATGCAAG

2401 CTGGCAGCGGCAGTGGTTGACGGGTGATGTACTGGAGCAACAGGCCACGTATTGGCGTGA

2461 GGCAATGTCTGGCGCACCGGTGTTGCTGGAACTGCCCACGGATCGTCCACGTCCGGCTCA

2521 GCAGGATCATGCAGGCGCGATGTTGGAGGTGATCGTCGATCCGCAGCAAGCACAAGCGTT

2581 GAAGGCCCTGAGCCAGCGCCATGGTCTGACGCTGTACATGACGCTCCTGGCGAGCTGGGC

2641 ATTGCTGCTATCGCGTCTATCCGGCCAAGACGATGTGGTGATCGGCAGTCCGGTGGCAAA

2701 TCGCGGGAGATCGGAAACAGAAGGATTGATCGGCTTCTTCGTCAACACGCTGGCGTTGCG

2761 GGTGGAATTGTCTGGCTCACCAACGCTGGCGCAACTGCTGGCCTCGGTGAAGAGCCGCAC

2821 ACTGCAAGCGCAGGCACATCAGGATATCCCGTTCGAGCAGGTCGTCGAGCTGATTCAACC

2881 GCCACGCAGTCTGGCGCATACGCCGCTGTTCCAAGTGATGTTCGCGTGGCAGAACACGCC

2941 GCAGGGCGAGTTGGATCTTGGTGCAATCGAGGCCAGCGGATTGGGTGTTGCACAGACAAG

3001 CGCGCAGTTCGACCTGTCGCTGTCATTGATGGAGAGCGATGAGGGGATCGTCGGCAGTCT

3061 GGCCTATGCCACGGCATTGTTCGAGCGTTCGACGCTGGAGCGGTGGATGGGCCATTGGCG

3121 GCATCTGTTGGAAGCGATGGTGGCCGATGGCGCCGAGCATCAGGCAGTAGATCGTCTTCC

3181 GTTGCTGGACGATGCCGAGCGCCATCAGGTGCTGACCCAGTGGAATGAAACCGCAGCAGA

3241 TTATCCGCGCGATGCCTGTGTGCATGAATTGTTCGAGGCACAGGTGGCGCGGACGCCGTC

3301 TGCCATCGCGGTGGTGCAGGGCGAAGTGTCGCTGACCTATGGCGAGTTGAACGCACGTGC

3361 CAACCGTCTGGCGCATTACCTGCGTGAGTTAGGCGTATGTCCGGATGATCGTGTGGCGAT

3421 CTGTGTGCAGCGCAGTGTGGAAATGGTGGTGGCGGTGCTTGCGGTGCTGAAGGCCGGCGG

3481 TGCGTATGTGCCGTTGGACCCGGCTTATCCTGTCGAACGTCTGACCTACATGCTGGAGGA

3541 CAGTGCGCCGGTTGCAGTTCTGGCGCAGACATTGACTTCGAACTTGCTCTCGAACTTGCT

3601 ACTGACCACGTCCGCGCCGATCATCAATTTGGACGAGTCGCACTGGCAGGATCGGTCAGT

3661 GTCGAATCTCTCGATGGATGGTCTGACCTCGGCGCATCTGGCCTATGTGATCTACACCTC

3721 TGGCTCCACGGGCATGCCCAAGGGCGTGATGATCGAGCACCGTAACACAGTGAACCTGCT

3781 GGCCTGGGCGCAGCGCTCCTTCGAGCCGTCGGTTCTGGCTAAAACTCTGTTCTCGACGTC

3841 GTTGAACTTCGACCTGGCGGTCTATGAATGTTTTGCGCCGCTGATATGCGGTGGTGCGAT

3901 TGAAGTAGTCGACAATCTCCTGGCGCTGCAAACAGGTCAACATGACATCACATTGATCAA

3961 CACGGTGCCTTCGGCGCTGAAAGCATTGTTGGAATCCGGTGGATTAGGGCGGAGCGTACA

4021 GACGGTCAACGTCGCCGGTGAGGCACTTAAGCGTCAGCTTGTTGAGGATATTTTTGCCAA

4081 GACGCAAGTCGAACGGCTGTGCAACCTATATGGTCCGTCGGAAACCACGACCTATTCGAG

4141 TTGGGTATCGATGGAGCGCAGCGACGGTTTTGTGTCTCATATCGGCACGCCGTTGGACAA

4201 TACCCAGTTCTATGTGCTGGATACGCATCGTCAGCCTGTGCCGGTGGGTGTGACGGGTGA

4261 ACTGTATATCGGTGGTACCGGTGTGGCGCGTGGTTATCTGAATCGCGGTGATTTGACCGC

4321 CGCGCGCTTCCTCATGGATCCATTCAGTGCCGATCCGACGGCACGGATGTATCGCACTGG

4381 CGATCTGGGTCGCTGGCGTGCCGATGGCACGCTTGAGTTTGTGGGACGCAACGACCATCA

4441 GGTCAAGATTCGTGGTTTCCGCATCGAGTTGGGAGAGATCGAGGCGCGGCTGAGCGCGCA

4501 TGTGGATGTGCGCGAGTGTGTGGTGGTGGCGCTGGAAGATGCCACGGGCAGCGACAAGCG

4561 ATTGGTGGCGTATTGGGTTG

LOCUS Contig G149 4434 bp DNA linear BCT 30-JAN-2012

DEFINITION Xanthomonas spp. strain XaS3 genomic region encoding NRPS.

SOURCE Xanthomonas spp. strain XaS3

ORGANISM Xanthomonas spp. strain XaS3

Bacteria; Proteobacteria; Gammaproteobacteria; Xanthomonadales;

Xanthomonadaceae; Xanthomonas.

REFERENCE Contig G149 (bases 1 to 4434)

AUTHORS Royer et al. Submitted to BMC Genomics.

TITLE Genome mining indicates that the genus Xanthomonas is a promising

reservoir for new bioactive non-ribosomally synthesized peptides

JOURNAL Submitted to BMC Genomics.

REFERENCE 2 (bases 1 to 4434)

FEATURES Location/Qualifiers

source 1.. 4434

/organism='Xanthomonas spp.'

/mol_type='other DNA'

/strain='XaS3'

/isolation_source='sugarcane leaf water droplets'

/host='Sugarcane'

/db_xref='taxon: GPE 39'

/country='Guadeloupe'

/collection_date='2003'

/collected_by='Rosiane BOISNE-NOC'

/note='[cultured bacterial source]'

gene <1..4432>

/gene ='NRPS'

CDS <1..4432>

/gene ='NRPS'

/note='incomplete NRPS, no start codon, no stop codon'

/codon_start=1

/translation='LIDHDLRGVPDREKALEKLLTEEAHAPFALEQGPLIRGRLIRLADDESVLFVTMHHIVSDGWSMGVLINELSVLYRAFAHGEADPLAPLPIQYADYASWQRQWLAGEVLEQQATYWRETLSGAPVLLELPTDRPRPARQDHAGAMLEVVIEPQQAQTLKALSQRHGLTLYMTLLASWALLLSRLSGQDDVVIGSPVANRGRSETEGLIGFFMNMLALRVEFSNSPTLAQLLALVRERALQAQAHQDIPFEQVVELIQPPRSLAHTPLFQVMFAWQNTPQGALDLGEIEVSGLGVAQTSAQFDLSLSLVESQEEIVGSLAYATALFERSTLERWMGHWRHLLDAMVAEGAEHQAVDRLPLLDDAERYQVLTQWNATTKDYPREACVHELFEAQVARTPSAIAVVQGEVSLTYDELNARANCLAHSLRELGVCPDDRVAICMQRSVEMVVAVLAVLKAGGAYVPLDPAYPVERLAYMLEDSAPVAVLAQTSTSNLLLTASAPIINLDESHWQDRSVSNLSMDGLTSAHLAYVIYTSGSTGRPKGVMIEHRNTVNLLAWAQRSFAASVLEKVLFSTSLNFDLSVYECFVPLVCGGAIEVVDNLLAMQADGQGVTLINTTPSALKGWLESGGRGEGVHTVNVCGEVLKRQVVEDLFAKTQVERLCNLYGPSETTTYSSWVSMERSDGFVSHIGTPLDNTQFYVLDTHRQPVPVGVTGELYIGGAGVARGYLNRGDLTAERFLMDPFSADPTARMYRTGDLGRWRADGTLEFVGRNDHQVKIRGFRIELGEIEARLSAHVDVRECVVVALEDATGSDKRLVAYWVATQDAMHESLGVESLRSWLSDTLPDYMVPAAYVQLDRLPLTPNGKLDRKALPAPDATAYAAPAYEAPQGEVEHTIAAIWRELLGLESIGRHDNFFALGGHSLLAVRVASRLRQELGVEIGVAELFANATLKDLAACVASSSGAILPPILPLQPDAPRVLSFAQQRLWFLSQFEGVSEAYHISGGLRLRGVLDAQALQRALDRIVARHASLRTSFALVDGQALQHVADEDSGFHLIDHDLREVPDREAVLEQLLAEEVQTPFALEQGPLIRGRLVRLADDESVLFVTMHHIVSDGWSMGVLINELSVLYRAFARGEADPLAPLPIQYADYASWQRQWLMGDVLEQQASYWRKTLSDAPVLLELPTDRPRPAQQDHAGAMLEVIVDPQQTQALKALSQRHGLTLYMTLLASWALLLARLSGQDDVVIGSPVANRGRSETEGLIGFFVNTLALRVELSSSPTLAQLLASVKERALQAQAHQDMPFEQVVELLQPPRSLAHAPLFQVMFAWQNTPQGELDLGELDASGLGVAQTSAQFDLSLSLVESEEGIVGSLAYATALFERSTLERWMGHWRHLLDAMVAEGAEHQAVDRLPLLDDAERYQVLTQWNATAADYPSDACVHELFEAQVALDPSAIAVVQGEVSLTYGELNARANRLAHY'

ORIGIN

1 TTTGATCGATCATGATCTGCGCGGTGTGCCTGATCGTGAGAAAGCGCTTGAGAAACTGCT

61 GACCGAGGAGGCGCATGCGCCATTTGCGTTGGAGCAAGGCCCACTCATCCGTGGCCGGCT

121 GATACGGCTTGCTGACGACGAATCCGTCCTCTTCGTCACGATGCACCACATCGTCTCGGA

181 TGGTTGGTCGATGGGAGTTTTGATCAACGAGCTGAGCGTGTTGTATCGGGCCTTTGCACA

241 TGGCGAAGCCGATCCGCTAGCACCGCTGCCAATCCAGTATGCCGACTATGCAAGTTGGCA

301 GCGGCAATGGCTAGCGGGCGAGGTACTGGAGCAGCAGGCCACGTATTGGCGCGAGACGCT

361 GTCTGGTGCGCCGGTGTTGCTGGAACTGCCAACGGATCGTCCACGTCCAGCGCGTCAGGA

421 CCATGCCGGGGCGATGTTGGAGGTGGTTATCGAGCCGCAACAGGCACAGACGCTCAAAGC

481 ACTGAGCCAGCGGCATGGTCTGACGCTATACATGACCCTCCTAGCGAGTTGGGCGCTACT

541 GCTGTCACGCCTCTCCGGCCAGGACGATGTCGTCATCGGCAGTCCAGTGGCTAATCGTGG

601 TAGATCCGAGACCGAAGGGCTGATCGGGTTTTTCATGAACATGCTGGCACTGCGGGTGGA

661 GTTCTCCAACTCGCCGACGCTTGCGCAATTGCTGGCCTTGGTGAGGGAGCGTGCACTGCA

721 GGCGCAGGCGCATCAGGACATCCCGTTCGAGCAGGTGGTCGAACTGATTCAACCGCCACG

781 CAGTCTGGCGCATACGCCACTGTTTCAGGTGATGTTCGCGTGGCAGAACACACCGCAGGG

841 CGCGCTGGATCTTGGCGAAATCGAGGTCAGTGGATTGGGTGTTGCACAGACGAGCGCGCA

901 GTTCGACCTGTCGTTGTCGTTGGTCGAGAGTCAGGAGGAGATCGTCGGTAGTCTGGCTTA

961 TGCCACGGCATTGTTCGAGCGTTCGACGCTGGAGCGGTGGATGGGCCATTGGCGGCATCT

1021 GTTGGATGCGATGGTGGCCGAGGGTGCCGAGCATCAGGCAGTAGATCGTCTGCCGTTGCT

1081 GGACGATGCCGAGCGCTATCAGGTACTGACGCAGTGGAATGCAACGACGAAGGACTATCC

1141 ACGCGAGGCCTGTGTACACGAACTGTTCGAGGCACAGGTGGCACGGACACCATCCGCCAT

1201 CGCGGTGGTGCAGGGTGAAGTGTCGCTGACGTATGACGAGTTGAATGCACGTGCCAACTG

1261 TCTGGCGCATTCTCTGCGGGAGTTGGGTGTATGCCCGGATGATCGCGTGGCGATCTGCAT

1321 GCAGCGCAGTGTGGAGATGGTGGTCGCGGTGCTTGCGGTGCTGAAGGCCGGTGGTGCCTA

1381 TGTACCACTAGACCCGGCTTATCCTGTCGAACGTCTCGCCTATATGCTGGAAGACAGCGC

1441 TCCGGTTGCGGTTCTGGCGCAGACATCGACCTCGAATCTGCTACTGACCGCATCCGCGCC

1501 GATCATCAATCTGGATGAGTCGCACTGGCAAGATCGGTCCGTGTCGAATCTGTCGATGGA

1561 TGGTCTGACCTCGGCGCATCTGGCTTACGTGATCTACACCTCCGGCTCCACGGGCCGGCC

1621 CAAGGGCGTGATGATCGAACACCGCAATACGGTGAATCTGCTGGCTTGGGCACAGCGTTC

1681 TTTCGCAGCATCGGTCCTGGAAAAAGTCCTGTTCTCCACGTCGTTGAACTTCGACCTATC

1741 GGTCTATGAATGCTTTGTGCCTTTGGTGTGCGGTGGTGCAATTGAGGTGGTCGACAATCT

1801 GCTCGCCATGCAGGCGGATGGGCAGGGCGTCACGTTGATTAACACCACGCCTTCCGCATT

1861 GAAGGGGTGGTTGGAATCAGGCGGACGAGGCGAGGGTGTCCATACGGTCAACGTGTGTGG

1921 TGAAGTGTTGAAGCGCCAAGTGGTGGAGGATCTTTTTGCCAAGACGCAAGTCGAACGGCT

1981 GTGCAACCTATATGGCCCGTCGGAAACCACGACGTATTCGAGTTGGGTGTCGATGGAGCG

2041 CAGCGACGGTTTTGTGTCTCATATCGGCACGCCGTTGGACAATACGCAGTTCTATGTGCT

2101 GGATACGCATCGTCAGCCTGTGCCGGTGGGTGTGACGGGTGAACTGTATATCGGTGGTGC

2161 CGGTGTAGCGCGTGGTTATCTGAATCGCGGTGATTTGACTGCCGAGCGCTTCCTTATGGA

2221 TCCATTCAGTGCCGATCCGACGGCACGGATGTATCGCACTGGCGATCTGGGGCGCTGGCG

2281 TGCCGATGGCACGCTTGAGTTTGTGGGACGCAACGACCATCAGGTCAAGATTCGTGGTTT

2341 CCGCATCGAGTTGGGAGAGATCGAGGCGCGGCTGAGCGCGCATGTGGATGTGCGCGAGTG

2401 TGTGGTGGTGGCGCTGGAAGATGCCACGGGCAGCGACAAGCGATTGGTGGCGTATTGGGT

2461 TGCGACGCAGGATGCGATGCACGAGTCCCTTGGCGTGGAAAGCTTGCGTAGTTGGCTGTC

2521 GGACACGTTGCCGGATTACATGGTGCCGGCGGCTTATGTGCAGTTGGATCGCCTACCGCT

2581 GACCCCGAACGGCAAGCTGGATCGCAAGGCATTACCCGCGCCGGATGCTACAGCCTATGC

2641 CGCGCCTGCATATGAAGCACCGCAGGGTGAGGTTGAACACACCATTGCCGCGATCTGGCG

2701 TGAGTTGCTGGGTCTGGAGAGCATCGGGCGGCACGATAACTTCTTCGCGCTCGGTGGACA

2761 TTCGTTGTTGGCGGTGCGAGTTGCCTCTCGCCTGCGTCAGGAATTGGGTGTCGAGATCGG

2821 CGTGGCGGAGTTGTTTGCCAATGCAACGCTGAAGGACCTTGCCGCGTGTGTGGCCTCTTC

2881 GTCTGGCGCGATCTTGCCGCCGATCCTGCCCTTGCAGCCGGATGCGCCGCGGGTGCTGTC

2941 GTTTGCACAACAACGGCTCTGGTTCCTATCGCAGTTCGAGGGTGTCAGTGAGGCGTATCA

3001 CATCAGCGGCGGCTTGCGTTTGCGTGGTGTGTTGGATGCGCAGGCGTTGCAACGTGCGTT

3061 GGATCGCATCGTGGCCCGGCATGCGTCGCTGCGCACGAGCTTCGCGCTGGTCGATGGGCA

3121 GGCGTTGCAGCATGTCGCTGACGAGGACAGTGGTTTCCATCTGATCGATCACGATCTGCG

3181 TGAGGTGCCTGATCGCGAGGCTGTGCTGGAGCAGCTATTGGCGGAGGAGGTGCAGACACC

3241 GTTCGCGCTGGAACAAGGTCCGTTGATCCGTGGTCGACTGGTTCGGCTTGCCGATGACGA

3301 GTCCGTCCTCTTCGTCACGATGCATCACATCGTCTCGGATGGATGGTCGATGGGAGTTTT

3361 GATCAACGAGCTGAGCGTGTTGTATCGAGCGTTCGCACGTGGCGAAGCCGATCCGCTAGC

3421 ACCGTTGCCAATCCAGTACGCAGATTATGCAAGCTGGCAGCGGCAGTGGTTGATGGGAGA

3481 TGTGCTGGAGCAGCAGGCGAGCTACTGGCGCAAGACGCTATCGGATGCGCCGGTGTTGCT

3541 GGAGCTGCCCACCGATCGTCCACGTCCGGCTCAGCAGGATCATGCCGGCGCGATGTTGGA

3601 GGTGATCGTCGATCCGCAACAGACACAAGCGCTGAAGGCCCTGAGCCAGCGCCATGGCCT

3661 GACGCTATATATGACCCTCTTGGCGAGTTGGGCGTTGCTGCTAGCGCGTCTTTCCGGTCA

3721 GGACGATGTGGTGATCGGCAGTCCGGTGGCTAATCGTGGGAGATCGGAGACAGAAGGATT

3781 GATCGGCTTCTTCGTCAACACGCTGGCGTTGCGAGTGGAGTTGTCGAGTTCGCCGACGCT

3841 TGCGCAACTGCTGGCGTCAGTGAAGGAGCGCGCACTGCAAGCGCAGGCGCATCAGGACAT

3901 GCCGTTCGAGCAGGTGGTCGAACTGCTGCAACCGCCACGCAGTCTGGCGCATGCGCCGCT

3961 GTTCCAAGTGATGTTTGCTTGGCAGAACACGCCGCAGGGTGAGTTGGATCTTGGCGAGCT

4021 CGATGCCAGTGGACTGGGTGTTGCACAGACGAGCGCGCAGTTCGACCTGTCGTTGTCGTT

4081 GGTCGAGAGTGAGGAGGGGATCGTCGGCAGTCTGGCCTATGCCACGGCATTGTTCGAGCG

4141 TTCGACGCTGGAGCGGTGGATGGGCCATTGGCGGCATCTTTTGGATGCGATGGTGGCCGA

4201 GGGCGCCGAACATCAGGCAGTAGATCGTCTACCGTTGCTGGACGATGCTGAGCGCTATCA

4261 GGTACTGACGCAGTGGAATGCAACTGCGGCAGATTATCCAAGCGATGCCTGTGTGCACGA

4321 GTTGTTCGAGGCACAGGTGGCGCTTGATCCATCAGCCATCGCGGTAGTGCAGGGCGAGGT

4381 ATCGCTGACCTATGGCGAGTTGAACGCGCGTGCCAACCGTCTGGCGCATTACCT

LOCUS Contig G151 4032 bp DNA linear BCT 30-JAN-2012

DEFINITION Xanthomonas spp. strain XaS3 genomic region encoding NRPS.

SOURCE Xanthomonas spp. strain XaS3

ORGANISM Xanthomonas spp. strain XaS3

Bacteria; Proteobacteria; Gammaproteobacteria; Xanthomonadales;

Xanthomonadaceae; Xanthomonas.

REFERENCE Contig G151 (bases 1 to 4032)

AUTHORS Royer et al. Submitted to BMC Genomics.

TITLE Genome mining indicates that the genus Xanthomonas is a promising

reservoir for new bioactive non-ribosomally synthesized peptides

JOURNAL Submitted to BMC Genomics.

REFERENCE 2 (bases 1 to 4032)

FEATURES Location/Qualifiers

source 1.. 4032

/organism='Xanthomonas spp.'

/mol_type='other DNA'

/strain='XaS3'

/isolation_source='sugarcane leaf water droplets'

/host='Sugarcane'

/db_xref='taxon: GPE 39'

/country='Guadeloupe'

/collection_date='2003'

/collected_by='Rosiane BOISNE-NOC'

/note='[cultured bacterial source]'

gene <3..4031>

/gene ='NRPS'

CDS <3..4031>

/gene ='NRPS'

/note='incomplete NRPS, no start codon, no stop codon'

/codon_start=1

/translation='RECVVVALEDATGSDKRLVAYWVATQDATHESLGVENLRSWLSDTLPDYMVPAAYVQLDRLPLTPNGKLDRKALPAPDATAYAAPAYEAPQGEVEHTIAAIWRDLLGLESIGRHDNFFALGGHSLLAVTLSERMRQQGLQADLRTLFTTPTLMALAAASGRVSVSVPPNRIGPDSVAITPEMLPLVALTQEQIDRIVAMTPGGSANIQDIYPLAPLQEGIFFHHLMQQQGDAYLLPNLIAFDSRSRLDRFVDALQRVIDRHDILRTAIAWEGLAAPVQVVWRHAPLPIEEVCLDDAEGDAAAQLQSRFDPRHWRMDMRQAPLMRGFTMQDSSSGRWLLQLLSHHAALDHTTLEIVLEEVSCHLCGEADTLPAPLPFRNFVAQALLGVSREEHEAYFRTMLADVDEPCAPFGLVDGQGDGSDVDEVRVNLQEPLSALLRSQARTLGVSTASLFHLAWAQVVARTTPHERVVFGTVLFGRMQGGVGADRALGMFINTLPLRIEIDGSSVVESVHLMQQRLAELLRHEHASLSLVQRCSGVPAPAPLFTSLLNYRHRIQAEGAAEAMAWEGIETMLAYGRTTYPLMVAIDDAGSGFALTVQAQRPLVAERICAFLVKALERLADALAHAPQTAVRNLDVLPEAERQQVLMQWNATTADYPRDACVHELFEVQVARDPSAIALVQGNESLTYGELNAQANRLAHYLCELGVRADDRVAICLERGTAAIVAMLGVLKSGAAYVPLDPTSPGKRLLALLDDCRASVVIVDQSLPTTSRDGLTLPTISLTDLALIQRATHNPDIAGLQRQHLAYVMYTSGSTGQPKATMITHQGLVAYAAALSERCALQPGDTSLVFTSLHFDLALTGIYPPLLCGGTVQLCAHDSTPADWAHALRQGRAIAPLKLTPSHLMLLQQELGDTLLDGCVRVLVLGGEAPSVDAVRWWRERSPSTQIFNHYGPTETTVGCLMHALCGDEDRIPLGLPLAGVRVYVLDVRGQLCPHGVPGELYIAGHGLARGYLGRADLTAERFVPDPFAQQPGQRMYSSGDLVRWRADSTLEFLGRNDDQIKLRGFRIELGEIQAALRACDGVRDAVVIARQYSAGELRLVAYVVGDVDGIELNPEAVRTQLGARLPDYMVPSAYVHLDDLPLTPNGKLDRKALPAPDAAAYAACAYEAPQGTIEQTIAAIWCDLLGLESIGRRDNFFALGGHSLLAVRVASRLRKELGVEIGVAELFAHATLQDLAACVASSSSAILPPILPLETDAPRVLSFAQQRLWFLSQFEGVSQAYHVSGGLRLRGALDTQALQRALDRIVARHASLRTTFALVDGQTLQHISAEDIGFHRIDHDLR'

ORIGIN

1 TGCGCGAGTGTGTGGTGGTGGCGCTGGAAGATGCCACGGGCAGCGACAAGCGATTGGTGG

61 CGTATTGGGTTGCGACGCAGGATGCGACGCACGAGTCCCTTGGCGTGGAAAACTTGCGCA

121 GTTGGCTCTCGGACACGTTGCCGGATTACATGGTGCCGGCGGCTTATGTGCAGTTGGATC

181 GCCTGCCGCTGACCCCGAACGGCAAGCTGGATCGCAAGGCATTGCCCGCGCCGGATGCGA

241 CAGCCTATGCCGCACCTGCGTATGAAGCACCGCAGGGTGAGGTGGAACACACCATTGCCG

301 CGATCTGGCGTGATCTGCTGGGTCTGGAAAGTATCGGACGCCACGACAACTTCTTCGCGC

361 TCGGTGGACATTCGTTGTTGGCGGTGACGCTGAGCGAGCGGATGCGCCAGCAGGGTTTAC

421 AGGCCGATTTGCGCACACTGTTCACCACTCCGACGCTTATGGCACTGGCTGCGGCGAGCG

481 GCCGCGTGTCAGTCAGCGTGCCGCCCAATCGCATTGGGCCAGACAGCGTAGCGATCACGC

541 CTGAGATGTTGCCGCTGGTAGCACTCACTCAGGAGCAGATCGATCGCATCGTTGCGATGA

601 CCCCAGGCGGTTCGGCCAATATCCAGGACATCTATCCGCTGGCCCCGTTGCAAGAAGGCA

661 TTTTCTTCCATCATCTGATGCAACAACAAGGTGATGCCTATCTGCTGCCAAACTTGATCG

721 CGTTCGATAGCCGTTCGCGTTTGGATAGGTTTGTCGATGCGCTGCAGCGTGTCATTGATC

781 GTCACGACATCCTGCGCACGGCGATCGCCTGGGAAGGGCTTGCCGCACCGGTCCAGGTCG

841 TGTGGCGGCATGCGCCGCTGCCGATCGAGGAGGTGTGTCTGGATGATGCCGAAGGTGATG

901 CGGCGGCACAGCTGCAATCGCGCTTCGATCCACGGCATTGGCGCATGGATATGCGTCAGG

961 CGCCGCTGATGCGTGGTTTTACGATGCAAGATTCGAGCAGTGGCCGCTGGCTGCTGCAGT

1021 TGCTCAGTCACCATGCTGCCTTGGATCACACGACATTGGAGATCGTGTTAGAGGAGGTCA

1081 GTTGCCACTTATGTGGTGAAGCTGACACCTTGCCAGCCCCGCTTCCGTTCCGCAATTTTG

1141 TGGCGCAGGCGCTGCTGGGCGTGAGTCGCGAGGAGCACGAGGCGTATTTCCGGACGATGC

1201 TCGCCGATGTAGATGAGCCCTGTGCCCCGTTCGGTCTGGTTGACGGACAGGGTGATGGAT

1261 CCGACGTGGACGAGGTGCGCGTCAATCTGCAGGAGCCACTATCGGCATTGCTGCGCAGCC

1321 AGGCACGGACGCTCGGCGTGAGCACGGCCAGTCTGTTCCATCTGGCCTGGGCGCAGGTGG

1381 TGGCACGCACAACGCCCCATGAGCGTGTTGTATTCGGAACCGTGCTGTTTGGCCGCATGC

1441 AGGGTGGAGTGGGAGCCGACCGTGCCCTTGGCATGTTCATCAATACCTTGCCGCTGCGGA

1501 TCGAGATTGACGGGTCCAGTGTGGTAGAGAGTGTGCATCTTATGCAGCAACGTCTTGCAG

1561 AGCTCTTGCGGCACGAGCATGCGTCGTTGTCGTTGGTGCAGCGGTGCAGCGGTGTTCCAG

1621 CACCTGCACCGTTGTTTACCTCGCTGCTCAACTACCGGCATCGGATTCAAGCAGAAGGTG

1681 CTGCCGAGGCAATGGCGTGGGAGGGAATCGAAACCATGCTAGCGTATGGGCGCACGACTT

1741 ATCCGCTCATGGTTGCTATCGACGATGCGGGGTCGGGATTTGCGCTGACGGTGCAGGCGC

1801 AGCGGCCACTCGTTGCGGAACGGATATGCGCGTTTCTAGTGAAGGCGCTGGAAAGGCTTG

1861 CCGATGCACTTGCACATGCACCACAGACAGCAGTGCGCAATCTCGATGTTCTACCTGAGG

1921 CCGAAAGGCAGCAGGTCTTGATGCAGTGGAATGCAACGACAGCGGATTATCCACGCGATG

1981 CGTGTGTACACGAATTGTTCGAAGTACAGGTAGCGCGTGATCCATCGGCCATCGCGCTGG

2041 TGCAGGGTAATGAGTCACTCACGTATGGCGAGTTGAATGCACAGGCCAACCGTCTGGCGC

2101 ATTACCTGTGCGAATTAGGCGTGCGTGCAGACGACCGAGTGGCGATTTGCCTGGAACGCG

2161 GCACCGCCGCCATTGTCGCCATGCTCGGTGTTCTCAAATCCGGCGCAGCCTATGTTCCGC

2221 TCGATCCGACATCGCCTGGCAAGCGTTTGCTCGCATTGCTGGACGACTGCCGCGCCAGCG

2281 TCGTCATCGTCGATCAGTCGTTGCCGACGACATCGCGCGATGGATTGACGCTGCCGACAA

2341 TCAGCCTAACCGATCTCGCGCTCATCCAGCGTGCCACGCACAATCCCGATATCGCGGGGT

2401 TGCAGCGGCAGCATCTGGCCTATGTGATGTACACCTCCGGTTCCACCGGCCAGCCCAAGG

2461 CGACAATGATCACCCACCAGGGGCTGGTCGCTTACGCTGCGGCACTGAGCGAACGCTGCG

2521 CCTTGCAGCCAGGCGATACCTCGCTGGTCTTTACTTCGTTGCACTTCGATTTGGCTCTGA

2581 CCGGGATTTATCCGCCGTTGCTGTGCGGCGGTACCGTGCAATTGTGCGCACATGACAGTA

2641 CACCGGCAGATTGGGCGCATGCACTGCGGCAAGGGCGCGCCATTGCTCCACTCAAGCTCA

2701 CTCCCTCTCATCTGATGCTGTTGCAGCAGGAACTTGGCGATACCCTGCTGGACGGATGCG

2761 TGAGAGTGCTGGTGCTCGGCGGCGAAGCACCGTCTGTCGATGCTGTGCGCTGGTGGCGGG

2821 AGCGCTCGCCAAGCACGCAGATCTTCAACCACTACGGCCCGACTGAAACCACAGTGGGTT

2881 GTCTCATGCACGCGCTGTGTGGCGATGAAGACCGCATTCCTCTCGGGCTTCCGCTTGCTG

2941 GTGTGCGAGTTTACGTGCTCGATGTGCGCGGACAGCTTTGCCCACATGGCGTACCGGGTG

3001 AACTGTATATCGCTGGTCATGGTTTGGCGCGTGGCTATCTTGGCCGTGCGGATCTGACGG

3061 CCGAACGTTTCGTGCCTGATCCGTTCGCTCAACAGCCTGGTCAACGCATGTACAGCAGTG

3121 GCGATTTGGTGCGCTGGCGCGCCGACAGCACGCTGGAGTTCCTCGGTCGCAATGACGATC

3181 AAATTAAGCTGCGCGGTTTCCGTATCGAGCTCGGAGAAATCCAGGCTGCCTTGCGTGCTT

3241 GCGACGGCGTGCGTGATGCAGTGGTCATCGCTCGTCAGTACAGCGCTGGCGAGTTGCGTC

3301 TGGTCGCCTATGTGGTGGGTGATGTTGACGGCATCGAGCTTAATCCCGAAGCAGTGCGTA

3361 CACAGCTTGGTGCGCGCTTGCCGGATTACATGGTTCCCAGCGCATACGTACATCTAGATG

3421 ACTTGCCGCTGACCCCGAACGGCAAGCTGGATCGGAAGGCATTACCCGCACCGGATGCCG

3481 CAGCCTATGCGGCGTGTGCGTATGAGGCACCGCAGGGCACGATTGAACAAACCATCGCCG

3541 CGATCTGGTGCGATCTATTGGGTCTAGAGAGCATCGGGCGGCGCGACAACTTCTTCGCGC

3601 TCGGTGGACATTCGTTGCTGGCGGTGCGGGTTGCTTCGCGTTTGCGCAAGGAATTGGGTG

3661 TCGAGATTGGCGTGGCCGAGTTGTTTGCGCATGCGACGTTGCAAGACCTTGCCGCTTGTG

3721 TGGCGTCCTCGTCCAGCGCGATCTTGCCGCCGATCCTGCCACTGGAGACAGATGCACCGC

3781 GCGTTCTATCGTTTGCGCAGCAACGGCTCTGGTTCCTGTCGCAGTTCGAGGGCGTTAGCC

3841 AGGCGTATCACGTCAGCGGCGGCCTGCGCTTGCGTGGGGCATTGGATACGCAGGCGTTGC

3901 AGCGTGCATTGGACCGCATCGTGGCCCGGCATGCGTCGCTGCGCACGACCTTCGCGCTGG

3961 TCGATGGGCAGACGTTGCAGCACATTTCTGCTGAGGACATTGGCTTCCATCGGATCGATC

4021 ACGATCTGCGCG

LOCUS Contig G167 877 bp DNA linear BCT 30-JAN-2012

DEFINITION Xanthomonas spp. strain XaS3 genomic region encoding NRPS.

SOURCE Xanthomonas spp. strain XaS3

ORGANISM Xanthomonas spp. strain XaS3

Bacteria; Proteobacteria; Gammaproteobacteria; Xanthomonadales;

Xanthomonadaceae; Xanthomonas.

REFERENCE Contig G167 (bases 1 to 877)

AUTHORS Royer et al. Submitted to BMC Genomics.

TITLE Genome mining indicates that the genus Xanthomonas is a promising

reservoir for new bioactive non-ribosomally synthesized peptides

JOURNAL Submitted to BMC Genomics.

REFERENCE 2 (bases 1 to 877)

FEATURES Location/Qualifiers

source 1.. 877

/organism='Xanthomonas spp.'

/mol_type='other DNA'

/strain='XaS3'

/isolation_source='sugarcane leaf water droplets'

/host='Sugarcane'

/db_xref='taxon: GPE 39'

/country='Guadeloupe'

/collection_date='2003'

/collected_by='Rosiane BOISNE-NOC'

/note='[cultured bacterial source]'

gene complement (<3..875>)

/gene ='NRPS'

CDS complement (<3..875>)

/gene ='NRPS'

/note='incomplete NRPS, no start codon, no stop codon'

/codon_start=1

/translation='HCAACAVLSERAIALPPLQQPRLDLDQLPPADASPIPAPTLPARSPAYVIYTSGSTGAPKGVVVAHDAVVAFAMGGGHAHLQADDRVAFVANPAFDAATFEVWTTLLHGASLVIVEQHLLLDPPALARHLAAHAVSILHLTAGLLPGYWQALAAWLPRLRCLLTGGDRVDARTIAQLLAHAPPQRLLHCYGPTETTVFCVTHPIAQVEPGSERLPLGRPLPGTRAYVLDAQGQPSPIGSSGELHLAGPQLAQGYLHLPAATAERFVPDPFAEHPGQRMYRTGDLARWRSDG'

ORIGIN

1 GTGCCGTCGCTGCGCCAGCGCGCCAGGTCGCCGGTGCGGTACATGCGCTGGCCGGGGTGT

61 TCGGCGAACGGATCGGGGACGAAGCGTTCGGCGGTCGCCGCCGGCAAGTGCAGATAGCCC

121 TGTGCCAGTTGTGGACCGGCCAGATGCAGTTCGCCGCTGCTGCCGATCGGGCTGGGTTGT

181 CCTTGCGCATCCAGAACGTAGGCGCGGGTGCCGGGCAATGGCCGGCCCAGCGGCAGACGT

241 TCGCTGCCGGGTTCGACCTGTGCGATCGGATGGGTCACGCAGAACACGGTGGTCTCGGTC

301 GGGCCATAGCAGTGCAGCAGGCGTTGCGGCGGCGCGTGCGCGAGCAGTTGGGCGATGGTG

361 CGCGCATCGACGCGGTCGCCGCCGGTCAACAGGCAGCGCAGTCGCGGTAGCCAGGCGGCC

421 AGGGCCTGCCAGTAGCCGGGCAACAGTCCGGCGGTCAGGTGCAGGATGCTGACGGCATGC

481 GCGGCAAGATGCCGCGCCAGGGCCGGCGGATCGAGCAACAGGTGCTGCTCGACGATGACC

541 AGGCTGGCGCCATGCAGCAAGGTGGTCCAGACCTCGAAGGTCGCCGCGTCGAAGGCGGGA

601 TTGGCGACAAAGGCGACCCGGTCGTCGGCCTGGAGATGTGCATGTCCGCCGCCCATGGCG

661 AAGGCGACCACGGCGTCATGGGCGACCACCACGCCCTTGGGTGCGCCGGTGGATCCAGAG

721 GTGTAGATCACGTAAGCGGGACTGCGCGCGGGGAGCGTCGGCGCGGGAATGGGTGAGGCG

781 TCGGCAGGTGGTAACTGATCCAGATCCAGGCGTGGTTGTTGCAATGGCGGCAGGGCGATC

841 GCGCGTTCGCTCAGCACCGCGCAGGCGGCGCAGTGCG

LOCUS Contig G169 824 bp DNA linear BCT 30-JAN-2012

DEFINITION Xanthomonas spp. strain XaS3 genomic region encoding NRPS.

SOURCE Xanthomonas spp. strain XaS3

ORGANISM Xanthomonas spp. strain XaS3

Bacteria; Proteobacteria; Gammaproteobacteria; Xanthomonadales;

Xanthomonadaceae; Xanthomonas.

REFERENCE Contig G169 (bases 1 to 824)

AUTHORS Royer et al. Submitted to BMC Genomics.

TITLE Genome mining indicates that the genus Xanthomonas is a promising

reservoir for new bioactive non-ribosomally synthesized peptides

JOURNAL Submitted to BMC Genomics.

REFERENCE 2 (bases 1 to 824)

FEATURES Location/Qualifiers

source 1.. 824

/organism='Xanthomonas spp.'

/mol_type='other DNA'

/strain='XaS3'

/isolation_source='sugarcane leaf water droplets'

/host='Sugarcane'

/db_xref='taxon: GPE 39'

/country='Guadeloupe'

/collection_date='2003'

/collected_by='Rosiane BOISNE-NOC'

/note='[cultured bacterial source]'

gene <1..822>

/gene ='NRPS'

CDS <1.. 822>

/gene ='NRPS'

/note='incomplete NRPS, no start codon, no stop codon'

/codon_start=1

/translation='VRPDERVAICLQRSVEMVVSLLGVLKAGGAYVPLDPAYPRERLAYMQADCGAVVVLTDTISRHLVGDSGISTVIVDLQADAERWAHLPDSNPDRNASGLTALHLAYVIYTSGSTGTPKGVMIEHRNLCNYALDAVRLFGVTHSDLVLQQNSISFDLSVEEIFPALLGGAALVLAPTIFGAVDHGCTDTSLYPRVTVVHLTMAHWHSLVGAWKQSPELARAQLQGVRLLNVTGDAISPQKLRQWHALRPYDIEVVNTYGPTETTVSCTAEHLRQD'

ORIGIN

1 GTGCGCCCGGACGAGCGGGTGGCGATCTGTTTACAGCGTAGTGTCGAGATGGTGGTGTCG

61 CTCTTAGGTGTGCTTAAGGCGGGCGGTGCGTATGTGCCGCTGGATCCGGCCTATCCACGG

121 GAGCGACTGGCCTACATGCAGGCCGATTGCGGCGCGGTGGTGGTGTTGACGGATACCATC

181 AGCCGCCATCTTGTCGGGGACAGTGGCATTTCTACGGTGATCGTCGATCTGCAAGCCGAC

241 GCCGAGCGCTGGGCACACCTGCCCGACAGTAATCCGGACCGCAACGCCAGTGGCCTGACT

301 GCACTTCACTTGGCGTATGTCATCTATACCTCCGGATCGACTGGTACGCCCAAAGGCGTG

361 ATGATCGAGCACAGGAATCTCTGCAACTACGCGTTGGATGCGGTGAGACTGTTCGGGGTG

421 ACGCACTCCGATCTGGTCTTGCAACAGAATTCGATCAGCTTCGATCTTTCGGTGGAAGAA

481 ATCTTTCCTGCCTTGCTCGGCGGTGCTGCGCTCGTTCTTGCACCAACGATATTCGGCGCT

541 GTCGATCATGGATGCACCGATACCTCTTTGTACCCACGGGTGACGGTCGTGCATTTGACG

601 ATGGCGCATTGGCATAGCCTTGTCGGTGCATGGAAGCAATCGCCGGAACTGGCGCGTGCA

661 CAACTGCAAGGTGTGCGACTGCTCAATGTGACTGGAGATGCGATATCTCCGCAGAAATTA

721 AGGCAGTGGCATGCGTTACGTCCTTACGACATAGAGGTTGTCAATACCTATGGACCGACG

781 GAGACCACGGTTTCTTGTACTGCGGAACATCTGAGGCAAGATGC
