# Supplementary material for: Genome mining reveals the genus Xanthomonas to be a promising reservoir for new bioactive non-ribosomally synthesized peptides
Source: BMC Genomics. 2013 Sep 27;14:658. doi: 10.1186/1471-2164-14-658 (PMC3849588; doi:10.1186/1471-2164-14-658)
Supplement: Additional file 8 — Primers used to determine the 82-kb length region containing META-B in strain BAI-3. [file 1471-2164-14-658-S8.doc]

**Additional file 7: primers used to determined the 82kb-length region containing META-B in strain BAI-3**

| **Names of primer** | **Sequences of primer** | **Clone(s) sequenced with the corresponding primer** |
| --- | --- | --- |
| **Primer located at the 3’ part of the scaffold DNA sequence of 35,406 bp** | | |
| MRK2 | CGGCGTTGAACGCATCAGCC | ABP0AAB4YK08 |
| **Primers located at the 5’ part of the scaffold DNA sequence of 16,409 bp** | | |
| MRK4 | AGCGCGCCGCTGCCGCTGTC | ABP0AAB4YK08 |
| MRK5 | GCTGCCGCCATTGCCGATCC | ABP0AAB4YK08 |
| MRK6 | AGGCGATGCTGACCTGGCAG | ABP0AAB4YK08 |
| MRK7 | GTCGGCCTGGAGAGCCGGGT | ABP0AAB4YK08 and ABP0AAB5YK06 |
| MRK8 | CGTCAGCGTCTGTGCGATGC | ABP0AAB4YK08 and ABP0AAB5YK06 |
| **Primers located at the 3’ part of the scaffold DNA sequence of 16,409 bp** | | |
| MRK12 | CGTGAATCGTCTGCCGCTGC | ABP0AAB5YK06 |
| MRK15 | CGATCTGGTGCGACCTGCTG | ABP0AAB5YK06 |
| MRK16 | GCGATGCGGCCACACAGCTG | Clone ABP0AAB5YK06 deleted with *Sal*I and harbouring an insert of 3.6 kb |
| MRK17 | GGATCGAGGTGGATGGCACC | Clone ABP0AAB5YK06 deleted with *Sal*I and harbouring an insert of 3.6 kb |
| MRK18 | GCATTACTTGCGTGGCTTGG | Clone ABP0AAB5YK06 deleted with *Sal*I and harbouring an insert of 3.6 kb and clone ABP0AAB5YK06 |
| MRK19 | GCAACTGGCCGCACCACAGG | Clone ABP0AAB5YK06 deleted with *Sal*I and harbouring an insert of 3.6 kb and clone ABP0AAB7YB12 deleted with *Sal*I and harbouring an insert of 1.9 kb |
| MRK20 | GCGAGATCGAGGCGCGCTTG | Clone ABP0AAB5YK06 deleted with *Sal*I and harbouring an insert of 3.6 kb and clone ABP0AAB7YB12 deleted with *Sal*I and harbouring an insert of 1.9 kb |
| **Primers located at the 3’ part of the scaffold DNA sequence of 22,029 bp** | | |
| MRK22 | CATGCACCATATCGTCTCGG | ABP0AAB5YK06 |
| MRK23R | CCAGCTCCACCACCTGCTCG | ABP0AAB5YK06 |
| **Primers designed in order to sequence gaps between contigs** | | |
| Contig0111 | CTGCAGCTGCAACCACTGCA | ABP0AAB3YM11 |
| Contig0185 | TGTTGCGCACAGGTATTCCG | ABP0AAB3YM11 |
| Contig0222 | AAGCTCGATCGCAAGGCATT | ABP0AAB6YE03 |
| Contig0226 | CTGCATGCCTGCGACGGTGT | ABP0AAB6YC14 and clone ABP0AAB4YK08 delected with *Sal*I and harbouring an insert of 5.0 kb |
| Contig0110 | CTGGACGATGCGGCAAGCAC | ABP0AAB5YJ04 and ABP0AAB7YB12 |
| **Primers designed in order to sequence the region between NRPS and AvrBs3 (contig00211)** | | |
| Cont0211 | GCACAGCAGGCTGTCGAGGT | ABP0AAB3YM11 |
| EndNRPS3 | TCCGTGCGCGGCATGACGTA | ABP0AAB3YM11 |
| Contig0384 | CATCCACTCAAGCCAGCGAT | ABP0AAB3YM11 |
| NRPS3END2 | CTTGGTCTGATGCATCTCAG | ABP0AAB3YM11 |
| **Primers designed in order to sequence regions containing putative frameshifts** | | |
| CRYPT1 | CCTGTCCGGACATCCTGATC | ABP0AAB3YG10 and ABP0AAB7YD04 |
| CRYPT2 | TGGTTGGATGTGCAGACCGG | ABP0AAB3YG10, ABP0AAB3YM11 and ABP0AAB5YE07 |
| CRYPT3 | GCTGATCTACGCCACGGCAC | ABP0AAB5YE07 and ABP0AAB3YM11 |
