# Supplementary material for: Genome mining reveals the genus Xanthomonas to be a promising reservoir for new bioactive non-ribosomally synthesized peptides
Source: BMC Genomics. 2013 Sep 27;14:658. doi: 10.1186/1471-2164-14-658 (PMC3849588; doi:10.1186/1471-2164-14-658)
Supplement: Additional file 9 — Annotation of the contigs of the published draft genome sequence of X. oryzae pv. oryzae strain X11-5A which were analysed in the current manuscript. [file 1471-2164-14-658-S9.docx]

**Additional file 9: Annotation of the contigs of the published draft genome sequence of *X. oryzae* pv. *oryzae* strain X11-5A which were analysed in the current manuscript**

**List of these contigs:**

- AFHK01000023.1 (see annotation page 1, corresponds to the contig 23.1 on figure 3)
- AFHK010000112.1 (see annotation page 15, corresponds to the contig 112.1 on figure 3 )
- AFHK010000103.1 (see annotation page 22, corresponds to the contig 103.1 on figure 3 )
- AFHK010000191.1 (see annotation page 29, corresponds to the contig 191.1 on figure 3 )
- AFHK010000114.1 (see annotation page 33 corresponds to the contig 114.1 on figure 3 )
- AFHK010000166.1 (see annotation page 39 corresponds to the contig 166.1 on figure 3 )
- AFHK010000167.1 (see annotation page 44 corresponds to the contig 167.1 on figure 3 )
- AFHK010000217.1 (see annotation page 49 corresponds to the contig 217.1 on figure 3 )
- AFHK010000229.1 (see annotation page 52 corresponds to the contig 229.1 on figure 3 )
- AFHK010000260.1 (see annotation page 56 corresponds to the contig 260.1 on figure 3 )
- AFHK010000272.1 (see annotation page 59 corresponds to the contig 272.1 on figure 3 )
- AFHK010000334.1 (see annotation page 62 corresponds to the contig 334.1 on figure 3 )

LOCUS AFHK01000023 32128 bp DNA linear BCT 09-MAY-2011

DEFINITION Xanthomonas oryzae X11-5A Xo_X11-5A_contig_23, whole genome shotgun

sequence.

ACCESSION AFHK01000023 [AFHK01000000](http://www.ncbi.nlm.nih.gov/sites/entrez?db=Nucleotide&cmd=Search&term=AFHK01000000)

VERSION AFHK01000023.1 GI:332357556

DBLINK Project: [66097](http://www.ncbi.nlm.nih.gov/bioproject/66097)

KEYWORDS WGS.

SOURCE Xanthomonas oryzae X11-5A

ORGANISM [Xanthomonas oryzae X11-5A](http://www.ncbi.nlm.nih.gov/Taxonomy/Browser/wwwtax.cgi?id=1009853)

Bacteria; Proteobacteria; Gammaproteobacteria; Xanthomonadales;

Xanthomonadaceae; Xanthomonas.

REFERENCE 1 (bases 1 to 32128)

AUTHORS Triplett,L.R., Hamilton,J.P., Buell,C.R., Tisserat,N.A.,

Verdier,V., Zink,F. and Leach,J.E.

TITLE Genomic Analysis of Xanthomonas oryzae from US Rice Reveals

Substantial Divergence from Known X. oryzae Pathovars

JOURNAL Appl. Environ. Microbiol. (2011). 77(12):3930-7.

PUBMED [21515727](http://www.ncbi.nlm.nih.gov/pubmed/21515727)

COMMENT Base Quality-A custom perl script was used to clean and remove

reads with low complexity and low quality regions. Low quality

regions were defined as an average quality score of < 20 over a

10bp window along the read or > 2 'N' bases in the read. The Velvet parameters used in the final assembly for X11-5A were a hash length of 31, expected coverage of 19.58x, and a k-mer coverage cutoff of 9.79.

Contact Dr. Jan Leach for source DNA or cultures (Department of

BSPM, Colorado State University, Fort Collins, CO).

##Genome-Assembly-Data-START##

Assembly Method :: Velvet v. 0.7.53

Genome Coverage :: 70x

Sequencing Technology :: Illumina GAIIX

##Genome-Assembly-Data-END##

**Annotation was performed by Royer et al. (current manuscript)**

FEATURES Location/Qualifiers

source 1..32128

/organism="Xanthomonas oryzae X11-5A"

/mol_type="genomic DNA"

/strain="X11-5A"

/host="Oryza sativa cv. Lemont"

/db_xref="taxon:[1009853](http://www.ncbi.nlm.nih.gov/Taxonomy/Browser/wwwtax.cgi?id=1009853)"

/country="USA: Texas"

gene complement (<1..5835)

/gene="NRPS"

CDS complement (<1..5835)

/gene="NRPS"

/note=incomplete NRPS (no stop codon)

/codon_start=1

/translation="MRASWMQQTLTARREWKPHDRACPFPYTPTDTGPARQHAG

NRALHLHHVGDCMSVVDLAPPSAQVALHALGSAQQGVWLGQLLAPDQPSYSIGCVMHFEGTLQRDVWEQAVAALIARHDALRIVLVEGSPLPSQRVLEGLSFSLPWHDYADHSDGEQRAWEHIRQATARPFALYGQPLWDIQWLQVSPTRGYCLYRCHHIIADGISMGMLSQQVVDEYNQRLRGHAEQSPSPSCLQALEADRAYLGSSRYQRDLTFWRERLQARPEPLFPSATAALGHQRSVQLRCELDTALFLELSTLSERLGGSFTHLIVACLSSCLSRLGNRQAPIALGLAVHNRRNAREREMFGMLSTQLPLYIAVSPRAEIASAMRTIAAQVRQAMRHASFPLQHALRELGEAGQLAPRPFDLSVSVEDFSAFGDAPIADGACHMLPLHPGYEDTALGVFVRRYSPQHPIVLEFNVNPDRMPVVLAEQALGALRQMLLALRDDPHTPVWRLPLLSPSQRQQVLYAFDERPGHPSSDCQVQHAFERQAAATPDAIALVHEDVTLSYAALEAQANQLAHHLCALGVAPDGRVAICLPRGTAMVVAVLATLKAGAAYVPLDATYPPERLAYLLQDCRASVLLTTPVCAETLPLPADLTVFFADAVQPVWQALPTTAPACAGSPLHAAYVIYTSGSTGRPKGVVMPHGQLLNLLQWEAEHCAAADLHALRTLQFSALGFDASFQELFSTLGTGGTLVLIDDTQRRDAHALYRHLCAQRIERLYMPYIALQSLAEAVLADPTLDALDCPLRQVLTAGEQLRITPAIRAFFAARPACRLHNYYGPTETHVASAHLLPADTAQWPLLPPIGSALPRTPLYVLDAQRQPLPVGAIGELYIAGVQVARGYLHRPALTAERFVADPFATQPGQRMYRTGDLARWRADGEIEFLGRNDDQVKLRGYRIEPGEIEAALRACPGVREAAVLLREDRPGDKRLVAYLVGTGLRVEHVRDQLAASLPDYMVPAACVLVPAIPTTTHGKLDRAALPVPDAGALPLQAYQPPEGERETLLAALWSELLGVEHVGRHDSFFALGGHSLLGVQLISRLRSALGIELPLATLFAHTRLADLAGALAHAAPSALPAIVPTERSAVVPLSFAQQRLWFLAQFDARADLAYLMPGTVALRGTLDVAALQWALNRILARHEALRTRFVATEDGAVQVVDPAGTGVGLDCIDLRQEPDPQAAAQHHAEQETALAFDLEGAPLLRARLLQRADDDHLLLVTLHHLVADGWSIGLLLQELGALYTARVRGQPDPLPPLPIQYTDYTLWQRRWIDVAQLQRQRQFWLEHLRDAPAQLTLPSDRPRPPEQDYAGAAIAVTLGAARTQALIALSQRHGATLFMTVLAAWGVLLARLAGQDQVVVGTPIAQRTRAETAALIGLFVNTQALHLDLRANPTVAELLAQVRATALAAQAHQELPFEQLIEALNPVRSLAHAPVFQVMFTWQNTPHADLALPGLHSEVLPGPARDAKYDLDLDLRLQDGCIVGSLRFATALFDADTIQRHWDSFGVLLDGLRGDDQARVNRLPLLTPAQRQQPRTLHAGDAPIDTLTAAPGDVVQWFAQQAAATPQAIAVVCGDAMLSYQQLERRSNQWAHRLLALGARPDSCVALCLPRGIAQVIAVLAVLKAGAAYLPLEPSQPDERLAAVLADAQPVLLLVDAPERAAFAADQAPPIQTIVALQAAASDEPEHAPTVPPPHAQQLAYVIYTSGSSGRPKGVMVEHHGLSVRLQELIRTYRLGPQDRVLQFATLAFDASVEELFGVLCSGASLVLRDDSWLDTERFWPLCAQARISVVDLPTRFWAQLCAHSLEIPACVRQVIVGGEALTPAMRQTWIQGTRTALLDTYGPTEAIVVATTQSVAADTPTGIGRPLAGTQAHVLDRCAQPLPIGARGELHLAGAALARGYLGRPMNRWVEKWLRVYLKCYINLILFYRNVYPPQSFDYTTYQSFNLPQ"

gene complement (5780..19441)

/gene="NRPS"

CDS complement (5780..19441)

/gene="NRPS"

/codon_start=1

/translation="MDKFAKTLPASFVQKRMWLLARLDPQASISYHIANGVRLIGELDSHALQAALDRIVVRHEVLRTSLVDVNGQIRQRIQPPSGFPLVHEDVGTCAADPAAVARIAQQEARRPFDLELGAPVRGRLLRLTAQEHVLLLTFHHIACDGWSIGVLLRELEALYPVFLRRQPDPLPPLSIQYADYAIWQSEQLQGDMLDTQLRFWTGQLAGAPALLPLPTDRARPALQDYRGAVVERLLPAELGQRLNALAQRHGCSLFVTLLAGWALLLSRISATDDVVIGTPVAGRTHPDLEALIGCFINTLALRLTVSGAPSMAQWLAHVRKAVLNAQDHQELPFERVVEALQPNRSLSHTPVFQVLFSLDGFSGEQTLHLPGLQLEPLPDASDMCAFDLMLSMHASAGGLRACMKYPIALFERSSVERHLDQFIALLHGMVADATARVDRLPLLSAFERQQWLQTLAQTQRVFADDDCLHTVFEQQVAQTPNAIAVVHEDLALSYADLDARANQLAHHLIAHGVGPEDRVALFLQRGIDLVVAILAVLKAGAAYLPLDQAYPAQRLAFMLADAQPRLLLAHAALSATLPTEAGIATVLMDDADAWALRPSHAPMRSDLLSQHPAYVIYTSGSTGTPKGVVVAHAQVVRLLHATRAHVAPSADDVWTLFHSCAFDFSVWELWGALAHGGRLVVVPQRIARDPAAFHALLCQQRVSVLNQTPSAFQALIEAQRHSQVQHHLRLVIFGGEALQPASVATWFAAHGQRTALLNMYGITETTVHVTAHALTEQDVQRSGHSPIGTPLSDLRAYVLGPDAQCLPVGVAGELHVAGAGLARGYLGRPGLTAERFVPDPFATHPGERMYKTGDLARWRADGSLDYLGRNDEQVKIRGFRIELGEIASALRACDGVRQSAVVVREDSTGDKRLVAYVVGDDDIGLPAEALRTQLGARLPDYMLPAAYVPLAALPLTANGKLDRRALPAPEADALATLTYVAPEGELETVLATLWSALLGVERVGRHDDFFALGGHSLLAVQLASRVRQRLGLVIGLAEIFAHARLSDLADAVSNASLDKQPAIVPSPRSGPLPLSFAQQRLWFIDMHGQIGTAYSLAYALRLRGALDVAALGRALNRIVERHEALRTCFVTVNGQPQQVIAPAERGCALLHHDLSSDSDPLQAAHAHAEAEAQAPFDLAKGPLLRGRLLRLGQHDHVLLLTVHHIVADAWSGAVLVDELTALYGAFTQGLADPLKPLPIQYADFALWQRRWIAGERLQRQLAYWVGHLQGAPALLDLPTDFPRPARPDHCGEVLPFRLDATQSAALKALARRHGVTLFMVILAAWAALLARLSGQREVVIGTPIANRHQAELESLIGLFVDSLALRIDLRQDPSVAELLAQTRATALAAQACQDIPFEHVVEALNPVRSNAHNPVFQVMFAWQNAPQGELALPELTLETLERSGRTAQFDLELSMQEDADCIVGSLGFATALFARSSIQRHIRQLLTLLGRMAAHDDAAMSALSVTAPQELQLLQDFNATDTPVAEVAGVHTLFERHAAQAATAIAVVDAARSLSYAELDAQANRLARHLIDLGVGPDQRVALCLGRSIELIVAMLAVLKAGGAYVPLDPAYPLQRLGFMLADSAPCALLTDAQGAAQLGAQASMPTILLDQPQPDWAQLPHTAPTVPALRTQHLAYVIYTSGSTGTPKGVAVDHGGLRNYCVAAATRYSLYSGDRVLQCSSPSFDIAVDEIFATLASGATLVLLPGQRLPAIAEFCRAIDRQRISVLNLPTAYWHAWMAEQTDSAAPLPATLRLVVCGGEALDPAHVARWHVLAEERVQLLNAYGPSETVSGVSFGAVVPGHALHIGGPIANVRLHVLDGRQQPLPIGVNGELHIAGAQLARGYLGRPDLTAERFVPDPFAPVAGARMYRSGDRVRWREDGTLLFVGRDDHQVKLRGFRIEPGEIEAALRTAPGVQDAAVLVREDRPGERSLVAYVATPSLVVEAVRAHVATRLPDYMVPARYVRLDALPLTTNGKLDRRAMPAPDDEHRSDPAGAAPQGGHEQTLAQLWCELLGIDRVSRDDDFFDLGGHSLLAVQLIARVRDSLGVELQIGDIFTHTQLQALALCLERSGGSDAHIESSPIVAVDRSAPLPLSFAQQRLWFLDRFDPQSQLAYLMPAGVRLSGPLDSLALRRALDRIVHRHEALRTRFGVEDGTPVQRIETLRSGFPLQCIDLTDMPDPEAAARDHAAREAGTGFDLQQGQLLRGRLLRLAEQEHVLLVTMHHIVSDGWSMGVFVRELGALYAAFLQGRDDPLPPLRVQYADYSSWQRQRISGQALQRQRDFWRAHLQDAPTLLDLPTDRPRPAQQGYVGDTVAFALEPAHSAALKQLGHRHGTTVYMTVLAAWAVLLARLSGQSDVVIGTPVANRTHAELEPLIGFFVNTQALRVDLSHNPTVATLLAQVRATALAAQTHQDLPFEQLIETLNPERNPGAHPLFQAMLSWENVPQPELQLPGLQLQAIDLQMQTIKFDLSMGLQEHDGCIVGTLGYACALFDRSTIQRHVALFVQLLQTMCAGDTQRVARLPLLPSEEHRQLLEMGVVAPTQQLPEASLFALFAQRAALTPDAPAIVSEAATLSYADLLSQAQRVAQQLMAAGVRPGDRVAVVLPRSAQLIVAELAVLHCGAAYVPLDPAHPAERLGELVGHCAARAVLSDSRVAVPVLELPRLDLDRLPPVAHSLAPPVAVPVLGPAYVIYTSGSTGTPKGVVVSHAAVTTFAMGGGHAQIQHDDRVAFLANPAFDAATFEVWAALLHGAAIVVVEQAVLLDPPALAAHLARHAVSVLHLTAGLVPGYWRALAGWLPRLRCLLTGGDRVDARAIADLLAQAPPQRLLHCYGPTETTVFGVIQAVAAVEPGSERLPLGRPLPGTCAYVLDRYGQPAPVGARGELHLAGAQLAQGYLHLPALTAERFVPDPFASVPGQRMYRTGDLARWRSDGTLDFLGRNDDQVKIRGFRVEPADVETAVRDCPGVREALVLTDAPRAGELRLVAYVVASAVVTPESLRALLSARLPAYMVPAAYVCLDALPLTANGKLDRQRLPAPSSDDLASDAYAPPQGLLEHTLATLWCELLGVARVGRHDNFFALGGHSLLAVTLIERLRRHGWQVDVRSIFAAPTLWGLAATLQTATEVVVPRNRIGADCCQLTPELLPLVELTQAEIDLVVTSVDGGAANVQDIYQLAPLQEGLLFHHLADTARDPYLQHTLLCVDTRERLDDVLAALQAVIARHDILRTAIVWERLGAPVQVVWRHAPLAVHEHAWNGSVERLRQHMAQTPMPLQQAPLLRAHVVQDVRNGRWLLGLQHHHLVMDHTTLELAMAEIQTCLAGRQDRLPPALPFRDFIAHAQLGVPQSDHQAFFTEMLADLDAPTAPFGLPTPADASIDSEHAHLQLSQTLAQNLRAHAGRLGVSVASLFHLAYALIVARSSGRDTVVFGTVLFGRMWAGDGAERVLGMFLNTLPLRVDCDRTGVAQAIHDVQHRLARLLRHEHAPLALAQRCSGVQSPAPLFTAVLNYRHAGDGHSSDPGWEGIEALPAQERTHYALLLSVNDHGDAGGFSLDVQAMPRVGAARAGRMMVQALAALDSALCQAPATPLHALDVLPQQDIATPRAPDAAPLPALRADCIHRLFEQQVARNPQSTAVLFEERALSYAQLNAQANRLAHHLIAHGVGPDTRVALCLERGPEMLIGVLAILKASAAYVPLDPSYPAQRLAFMLEDSAPQCVLSHTAMSARLPHGRIPLVWLDDSDAWAQQPERNPDPAPGFGPHHLAYVIYTSGSSGRPKGVMVQHDAVVNLWRALHPVVHADAASAPPCVSLNASLSFDASVKMWVQLLSGARLAIVPQTVRLDCEALLAWMRRSRLDVLDCTPAQLQLLLDNGLLEATDGVPKTVLIGGEAIAPAMWQRLSQCTQIAFFNVYGPTECTVDVTVAAIRPDALLPTLGTPLANVRLALLDHRCRPVPAGVIGELMVGGIQVARGYLHRPGLSAERFVPDPFAEQSGQRMYRTGDLARWRADGALEFVGRNDGQLKLRGFRIELADVQAALLRCDGVRAAVVIAHDDGRGDVRLVAYWVGTAADRADDSLRTQLSAQLPDYMVPSVYLQLDALPLNANGKLDRQALPLPEAALPDANAYVPPANAIERALAKLWAKVLGQRQIGRNDHFFELGGHSLLVVRLIAAAKRSGLELSVQMVYDCPTLCAQAARLTGAAAQTPGPHALAARRGGTRSPLFVLPTGVGDITYAFELAAHLDRDIPIYALPWPDPLPATMQALAAQTAALIQAVQPHGPYHLLGYSSGGLLAYAIAQHFGMHGESVGLLGLLDCDVPVAQTDTEALDDAIGRALLRQLEGLQHHRPYQERADIQLALRALLDRIGDSPYPDMRSVCESDPVLAQLAGEEQTSVAALLHSCVISTCFNRLWPAFDARPLPATCRLHLFQASEPEPATDAYGWQQLLPDAQIERIAVGGEHTTLIEAEHIATLARRIERALDSAARTAFAPRLAAADAGELDAADADCAPGMETP"

gene (19975..21066)

/gene="dpgA"

CDS (19975..21066)

/gene="dpgA"

/codon_start=1

/translation="MNFNADVASTDARPHIASRIRGVGTATPGSRYSQPDVLERFGIEDQRRRQVFLRNGIDSRSLVLPRREQTEAAPRETQAQLLDKHRETGLEIGEHALRRCLQSIGAPLDQVQYLCCVTTTGLLTPGFSSLLIQRLGLRQDCLRLDVVGMGCNAGLNGFNAAVNWANANTGKLAILLCIEVCSAAYVDDEGIETAVVNSLFGDGAAALAVIADSADAHGGPRVCKFASQVIPEALDAMRFVWDQAQGKFHFRLHKDVPYVVGANAPTVIDRLLEGTGLRRRDIAHWLVHSGGRKVIDAISANLMLTSHDMRHTIEVLREHGNMSSGSFLFSYARLLDEGQVRPGDWGVMMTMGPGSSIETALLCW"

gene (21109..21849)

/gene="dpgB"

CDS (21109..21849)

/gene="dpgB"

/codon_start=1

/translation="MSDPANPMLHLVHLKIDAASALSDEQIQRLTRACDAVEDATSPALLILHLRGTTAPSAAARWPGTVSLDLVGRWEKAMRRFERLQGLSLAWIEDTCSAGMLDLLLATDQRIATPTASICMHGGEAAWPSMAMRRMSTRLGMSAARAVFLFETQLCSARMAQLGVVDQLSDDVQGAIAAWLATLAGVDVQVLPSRRLLLLEGVAHSYEQALGQHLAACDVELRRRQLRALQHAPADGAQDVPAQAVSA"

gene (21849..23123)

/gene="dpgC"

CDS (21849..23123)

/gene="dpgC"

/codon_start=1

/translation="MNPPADDMAQLHALAGWSGQALAALPPPAQRDPRQSQRAAAIHTRCRSARMDFMRRHGGWLYQTLTHHCTLALRLDEVLHAAATRVPGLLPSQAQWAHESACIQSEKEGWEIEQGIVLWGVLRQPECGHHLIESLLRPTARALELLAEFRSQAQVQIGPIHLQRRDGVAWLTVANQTGLNAEDNALVEAMEVAVDLALLDDQVAVGVVRGGPMQHPKYTGRRVFCSGINLQHLHQGKISFVDFLLRREFTYLNKMRRGLSWLGADARERTIEKPWVAAVDAFAIGGGLQLLLVFDHVVAERDAYFVLPAAKEGIVPGSANLRLGLRAGHRVGRDMILRGRIIRADEPDAAGLVDDVVDADDMDAAIARAVEQMRSEAVAPNRRMLRIAEESPEDFRRYMAEFALVQSSRIYADDVLARLRQRWAT"

gene (23147..24466)

/gene="HpgT"

CDS (23147..24466)

/gene="HpgT"

/codon_start=1

/translation="MDDLQPPALAMLDPSQLHPCLGEPVMGAIDFLNEVIDRYPNAISFAPGAPAPALLEPLDLSAYLQTYLAHLTQTEGLSELQVRRRLFQYGPSRGIINALLAQALRQDQGLDIAPSSIVVTVGFQEAMFLVLRALFAAPGDVLAVVQPCFVGAMGAARALDIPLLGIDERDGRVDLEQLHRACTELHAQGRRVRALYVAPDISNPSGSLLDREQRQALLDAADAHDFWLLEDNTYGFTVPHAQSVPSLKAMDRKGRVIHMGTFAKIALPGIRVGFVIANQRVAGRPPRLLADALATLKSMLTVNTSPVCQAIAAGMLLRSGGSLAALGAERAAFYQGKLACLLAALERELGDLAAAHPALRWNRPTGGFFVCLQLGVQVDADLLELSARDHGVLWTPMRMFYLDDGGQHSLRLSCSYLTDAQIDDGVKRLRRFLCDPRVLG"

gene (24466..25260)

/gene="dpgD"

CDS (24466..25260)

/gene="dpgD"

/codon_start=1

/translation="MNDAPVLFERDGHVARITLNRPAVLNALDLATHAALAEIWDAFEADDTLWVAMLSGSGERAFSVGQDLKELAARLADGAPSSSFGSRGAAGWPRLTERFDLSKPVIARVNGLALGGGFELALACDIIVACDTAEFALPEARLGLIPGAGGVFRLTRQLPYRSAMGYLLSGRRIGAARALALGLVNEVVPAAQLDTCVDGWLSDLLACAPLSLRAIKQAAATSASLPLADAFAAQYPWEERRRHSQDSREGPLAFVEKRAPRWCGQ"

gene (25340..27043)

/gene="ABC transporter"

CDS (25340..27043)

/gene="ABC transporter"

/codon_start=1

/translation="MLIQRYLRRHARILAGVSVLSAASAGTSMVLLDYLNTTATDALQIDMRDALLRGALLLVAALVVRLLSARLAARVSSGLMADLRTELSARFLELPLERLMHRKHAVFGALIGDVGRLAQMIQMGPMLLTNSLLSLGGLLYLAWVSLPLFAVVVPFIGLSGALFYVTRRFTGPAYDRMRKAEETLNGLLRTLVEGKKELTLLPARARHFAQAELRPAIERARSTQFDTSMHWGISDACAELIGYGWVLAAILAGRYLFDLPSATILQFVITGLFISGPLNALFDLGAQVGSASASVRHLREMGLDDAPSAPSPAAAAPEVMRMWTTLRLEQVVYRYASDNGDHFQFGPLDFTLRRGETVFVTGGNGSGKSTLLLLLSGLLRPSEGRILVDGHALETSLTLTSYRAMFSAVFFDFMLFSHVIGCDAAPADPAQVQDWLERVDLAAKVAFDKAEGVFASVNLSQGQRKRLALVQACLDDRQIMLFDEFTADQDQAFRERFYTVLLPELRARGKTLVLVTHDAGYRHLADRVLSLDYGHVVAPEPMCPPPFPAEVRGPALEEVQVSGMSPET"

gene (27145..27360)

/gene="mbtH"

CDS (27145..27360)

/gene="mbtH"

/codon_start=1

/translation="MSIQDDEQDQIYVVVINHEEQYALWPDYADVPNGWSVVFGPNPKQACLDYVNEHWTDMRPKSLRDAMAAAGH"

gene complement (27469..29061)

/gene="staM'"

CDS complement (27466..29061)

/gene="staM'"

/note="putative amino acid β-hydroxylase"

/codon_start=1

/translation="MSETRGYLRANAVARPLLNHWILWDMLIPPVQSALVVAKQQLPILESYLRAPEQHAQAARDPALMGGPWINYPSPRTAEIAQLLERTRAAQGDALALAAALQALEDLLRQQATGQSLESLYTQVPGELAGLVELVYDLNDHPGLRLLEGLLYRSRFYRRDLQQFAFGRVDRDWLPYERSTPVLESPDTPVLALPWDDARLDALFRAERVPVVIDDMAEQLGVGASQRAGFAALFTAAPPAQRHRPPAAGVRIRYFGHACLLIETDQVSILTDPFVAYDYPTDLPRYTLADLPERIDYVLITHGHSDHIRPETLLRLRHRIGTVVVPHSAGRRLQDPSLKLMLQALGFERVIELHEFERIALADGAITALPFLGEHSDLDIQGKAGYHVRIKGRSAACLADSCNLDPSLYRHIAAELGPVDALFLGMECEGSPLSWGYGHLLTRRIDPKLDRSRRDRGSHADEAIALIERWPARRAYVYAMGQEPWLNHVLAINQSGEHLGLREADRFLAHCRARGIEGERLFAMKELHLPAG"

gene complement (29079..30659)

/gene="staM"

CDS complement (29079..30659)

/gene="staM"

/note="putative amino acid β-hydroxylase"

/codon_start=1

/translation="MTSHRLRLSEHVRLLPRINGWFAHPYLISPLTFGLYTEHSHLAMMESFLEDPEQHRAALREPDMRGGPFIDHAGDVADIRKLRDQTLQRCAPQLRYAEAISALYEVLHNEAKGAGLAGLYTRIDTRIRDKLEIFYDVSKQPGVRFMERQFYASDAYDTGLQTAVLEPVSEQERAFALSTPQLPSAAGSLELSVPFADPFWDQLCGGMSDADALLDLIVRHAPAGTTRAQARALLTDAPLPAPPAPEGVRVRYFGHACVLIEGAGVSILIDPLISYPGECAIDHFTFDDLPAKIDYLLITHPHQDHVVMEALLRIRHRVGTVVVGRAGGGDLQDISLKLCLEHCGFTKVVELADYEELRFPQGRIVGAPFYGEHADLDIRAKLVHAVELDGKVCVLFADSRPPMVECYAQLKALFPKIDCMFLGMECVGAPATWLYGPLLQKMLTRGEDQSRRLDGCDCALASALQDFFLPERLYVYAMGAEPWVTHITSILYSEDLPQFREARALEEAARAKGRHAELLFGRCEITL"

gene (30858..31808)

/gene="syrP"

CDS (30858..31805)

/gene="syrP"

/note="putative amino acid β-hydroxylase"

/codon_start=1

/translation="MLKYSPLFENQKGPTIISPASGRHSLLDVIVPEMPHYRQELNERGAILLRGFYVSEVKHFDAFVEGVSQQQYRYVYRSTPRTEISNRVSTATNYPARLEIPMHNESAYHTTWPLLLAFCCLEAPAEGGQTPIAAMRGITRNIGQELLERLEEKGVEYIRNYHPNIDLPWQTVFQTEDRSKVDDYCAGSGIASHWGADGLLRTANRAQGIAFHPVTSEKLFFNQAHLFHVSSLGRTQAQAMMNMFGADKLPRHARFGDGTEISEQDLQKIQQAFSSEALLFHWQPGDVLLLDNMKFAHGRKPYKGSRAVFAALMEPSR"

ORIGIN

1 ggggcggccc agatagccgc gtgccagcgc cgcgccggcc agatgcagtt cgccgcgcgc

61 accgatcggc aacggttggg cgcagcgatc cagcacatgc gcctgggtgc cggcgagcgg

121 gcggccgata ccggtcgggg tgtcggcggc aacggattgg gtggtggcta ccacgatggc

181 ttcggtgggg ccgtaggtgt ccagtagtgc ggtacgtgtg ccctggatcc aggtctggcg

241 catggcaggc gtcagcgctt cgccgccgac gatgacctgg cgcacgcacg ctggaatctc

301 cagcgagtgc gcgcacagct gcgcccagaa gcgggtgggc aggtcgacca cgctgatgcg

361 ggcctgggca cacagcggcc agaaccgctc ggtgtccaac cagctgtcgt cgcgcagcac

421 caagctggcg ccgctgcaca ggaccccgaa cagttcttcc accgaggcat cgaaggccag

481 cgtggcgaat tgcagcacgc ggtcctgcgg gccaagcctg taggtgcgga tcaactcctg

541 caggcgcacg ctcaagccgt ggtgctcgac catcacgccc ttgggccggc cgctggagcc

601 ggaggtatag atcacatagg ccagctgctg agcgtgcggt ggcggaaccg tcggggcatg

661 ttcgggctcg tcagaggctg ccgcctgcag cgcgacgatg gtctggatcg gtggggcctg

721 atcggcagcg aacgctgcac gctcgggcgc gtcgacgagc agcagcactg gctgggcatc

781 ggccagcacc gcggcgaggc gttcgtcggg ctggctgggt tccagcggca ggtaggcggc

841 gccggccttg agcacggcca gcacggcaat cacttgcgcg ataccgcgtg gcaggcacag

901 cgccacgcag ctgtccggcc gcgcgcccag cgcgagcagc cggtgcgccc actggttgga

961 gcgacgctcc aactgctggt agctcagcat ggcatcgccg cacaccactg cgatggcctg

1021 cggcgtggcg gcggcctgct gcgcgaacca ctgcaccacg tcgcctggcg cggccgtgag

1081 cgtatcgatg ggcgcatcgc ctgcatgcaa ggtgcgcggt tgctgccgtt gcgcgggcgt

1141 gagcagcggc aaccggttca ctcgcgcctg atcgtcgccg cgcaggccat cgagcaggac

1201 gccgaagctg tcccagtgcc gctggatcgt gtcggcatcg aacagcgctg tggcgaagcg

1261 caggctgccg acgatgcagc cgtcttgcag ccgcagatcc aggtccaggt cgtacttggc

1321 atcgcgggcc gggccgggca ggacctcgct atgaaggcct ggcagcgcca agtcggcgtg

1381 cggcgtgttc tgccaggtga acatcacctg gaacaccggc gcatgggcca ggctgcgtac

1441 cgggttgagc gcttcgatca gctgttcgaa gggcagctcc tggtgcgcct gcgcggccag

1501 cgcggtggcg cggacctggg ccaacaactc ggccaccgtc gggttggcgc gtaggtccag

1561 gtgcaacgcc tgggtgttga cgaacaagcc gatcagcgcg gcggtctcgg cccgcgtgcg

1621 ttgggcgatg ggcgtgccga cgaccacctg gtcctggccg gccaggcgtg ccagcaacac

1681 gccccaggcg gcgagcacgg tcatgaacag cgtggcgcca tgccgctggc tcagtgcgat

1741 cagcgcctgg gtgcgtgcgg cacccagcgt caccgcgatg gcggcgccgg catagtcctg

1801 ctcgggcgga cgcgggcggt cgctgggcaa ggtcagctgc gccggtgcat cgcgcaggtg

1861 ttccagccag aactggcgct ggcgttgcag ctgcgccacg tcgatccagc ggcgctgcca

1921 cagcgtgtag tcggtgtact ggatgggcaa agggggcagc ggatcgggtt ggccacgcac

1981 gcgggcggtg tacagcgcgc caagctcctg cagcagtaag ccgatcgacc aaccatcggc

2041 gaccagatga tgcagcgtga ccagcagcag atggtcgtcg tcggcgcgct gcagcaagcg

2101 cgcgcgcagc agtggcgcgc cctccaggtc gaaggcaagc gcggtctcct gctcggcgtg

2161 gtgctgggcg gcggcttgcg gatcgggttc ctggcgcaga tcgatacagt ccagccccac

2221 gcctgttccg gcaggatcga cgacctggac ggcgccgtcc tcggtggcga cgaagcgggt

2281 acgcaaggct tcatggcggg cgaggatgcg gttcagcgcc cactgcagcg cggccacgtc

2341 cagcgtcccg cgcaaggcga cggtgccggg catcaggtag gccaggtcgg cacgcgcatc

2401 gaactgggcc aggaaccaca gccgttgctg ggcgaaggac agcggcacta cggcgctgcg

2461 ctcggttgga acgatggccg gcaaggcgct gggcgcggcg tgtgcgagcg cgccggccag

2521 atcggccagg cgcgtgtggg cgaacagggt cgccagcggc aattcgatcc ccagcgcgct

2581 gcgcaggcgc gagatcagct gcaccccgag cagcgagtgg ccgccgagcg cgaagaagct

2641 gtcgtggcgg ccgacgtgtt cgacgccgag tagctcgctc cacagcgcgg cgagcagggt

2701 ttcgcgttcg ccttccggcg gctggtaggc ctgcaagggc agggcaccgg catccgggac

2761 cggcagcgcc gcgcgatcca gtttgccgtg cgtcgtggtg gggatggcgg gtaccagcac

2821 gcacgccgct ggcaccatgt agtcgggcaa gcttgccgcg agctggtcgc gaacgtgttc

2881 aacgcgcagc ccggtgccga ccagataggc gaccaggcgc ttgtcgccgg ggcgatcctc

2941 gcgcagcagc accgccgcct cgcgcacgcc agggcaggcg cgcagggcgg cctcgatttc

3001 gccgggctcg atgcgatagc cgcgcagctt gacctgatcg tcgttgcggc cgaggaactc

3061 gatctcgcca tcggcacgcc agcgtgccag gtcgccggtg cgatacatgc gctggccggg

3121 ttgcgtggcg aatggatcgg cgacgaagcg ctccgcggtc agcgccggcc ggtgcagata

3181 cccgcgcgcg acctgcacgc cggcaatgta cagctcgccg atggcgccca ccggcaacgg

3241 ctggcgctgc gcatccagca catacaacgg cgtgcgcggc agggcgctgc cgatcggcgg

3301 cagcagcggc cactgcgcgg tgtcggcagg cagcaagtgt gcggaggcga cgtgggtttc

3361 ggtggggccg tagtagttat gcagccggca ggccggacgc gcagcgaaga atgcgcggat

3421 ggccggggtg atgcgcagct gttcgccggc ggtgagcact tgtcgcagtg gacagtccag

3481 cgcatcgagc gtcggatcgg ccagcaccgc ctcggccagc gactgcaggg cgatgtaggg

3541 catgtacagg cgttcgatgc gctgtgcgca gagatgccgg tacagcgcat gcgcgtcgcg

3601 gcgctgcgta tcgtcgatca gtaccagcgt gccgccagtg ccgagggtac tgaagagttc

3661 ctggaaactg gcatcgaagc cgagcgcgga aaactgcagg gtgcgcaagg catgcaggtc

3721 ggcggcggcg cagtgctcgg cttcccactg gagcagattg agcaattggc catgcggcat

3781 caccacgccc ttggggcggc cggtcgagcc ggaggtatag atgacgtaag cggcgtgcag

3841 cggcgagccg gcgcatgccg gtgcggtggt cggcaaggct tgccataccg gctgcactgc

3901 atcagcgaag aacacggtca gatccgcggg caagggcagt gtctcggcgc agaccggtgt

3961 ggtcagcagc accgacgccc ggcagtcctg cagtaggtag gccaggcgct cgggcggata

4021 cgtcgcgtcc agcggcacat aggccgctcc ggccttaagc gtggccagca ctgccacgac

4081 catggcggta ccgcgcggca ggcagatggc gacgcggccg tcgggggcca cgcccagtgc

4141 gcacaagtga tgcgccagtt ggttggcctg agcctccaat gcggcgtagc tgagggtcac

4201 atcctcgtgc accaatgcga tggcatctgg agtcgcggcg gcctggcgtt cgaacgcgtg

4261 ctgcacctgg cagtcgctgg aaggatggcc ggggcgttcg tcgaaagcgt acagcacctg

4321 ctggcgctgc gatggcgaca gcagtggcaa gcgccacacc ggcgtgtgcg gatcgtcgcg

4381 caacgccagc aacatctgcc gcaatgcgcc gagcgcctgt tcggccagca ccacgggcat

4441 ccggtccggg ttgacgttga actccagcac gatcggatgc tgcgggctgt agcgccggac

4501 gaacacgccg agcgcggtgt cctcgtagcc ggggtgcagc ggcagcatgt gacaggcgcc

4561 gtctgcgatg ggtgcgtcgc caaatgcgct gaagtcttcg accgacacgc tgaggtcgaa

4621 cggccgcggc gccagctgcc cggcctcgcc gagttcgcgc agcgcatgtt gcagcgggaa

4681 gctggcgtga cgcatggcct ggcgcacttg cgcggcgatc gtgcgcatgg cgctggcgat

4741 ctccgcacgt ggcgacactg cgatgtagag cggcaactgc gtggacaaca tgccgaacat

4801 ctcgcgttcg cgggcgttgc ggcgattgtg gacggcaagg ccaagtgcga tcggcgcctg

4861 acggttgcca aggcggctga ggcaactgct caagcaggcg acgatcaggt gggtgaacga

4921 gccgcccagg cgctcgctca gcgtgctcag ctccaggaag agcgcggtgt ccagctcgca

4981 gcgcaattgc acgctgcgct gatggcccag cgccgccgtc gcgctgggaa acagcggctc

5041 cggtcgtgcc tgcaggcgct cacgccagaa cgtcagatcg cgttgatagc ggctggagcc

5101 gagataggcc cggtcggcct cgagcgcttg cagacaggac ggggatgggg actgttcggc

5161 gtggccgcgc aggcgctggt tgtactcgtc cacgacctgt tgcgagagca ttcccatcga

5221 aatgccgtcg gcgatgatgt ggtggcagcg gtacaggcaa tagccacggg tgggggagac

5281 ctgcagccac tggatgtccc acagcggctg gccgtagagc gcgaacggcc gggccgttgc

5341 ttggcggata tgctcccatg cgcgttgctc gccatcgctg tggtcggcgt aatcgtgcca

5401 aggcagggag aaggacagcc cttccagcac gcgttggctt ggcagcggcg acccttcgac

5461 cagcacgatg cggagcgcgt cgtggcgtgc gatcagtgcg gcgacggcct gctcccagac

5521 atcgcgttgc agcgttccct cgaagtgcat cacgcagccg atgctgtagc tgggctggtc

5581 cggtgcgagc agttgcccta gccacacgcc ttgctgagcc gaaccgagcg catgcagcgc

5641 aacctgggcg gaaggagggg ccagatcgac aacgctcatg caatctccca catgatgaag

5701 gtgtagagcg cggttgccgg cgtgctgccg cgccgggccg gtgtctgtcg gtgtgtatgg

5761 gaatgggcaa gcgcggtcat ggggtttcca ttcccggcgc gcagtcagcg tctgctgcat

5821 ccaactcgcc cgcatcggcg gcggcgagcc gcggcgcgaa cgcggtgcgg gcagcgctgt

5881 cgagcgcccg ctcgatgcgg cgcgccagcg tggcgatatg ctcggcttcg atcagcgtgg

5941 tgtgctcgcc gccgaccgca attcgttcga tctgggcgtc tggcagcagt tgttgccagc

6001 cgtaggcatc ggtggccggt tcgggttcgc tggcctggaa caggtgcagt cggcatgtcg

6061 ccggaagtgg ccgcgcatcg aacgccggcc acaagcgatt gaagcaggtg ctgatgacgc

6121 agctatgtag cagggccgcg accgaggtct gctcttcgcc cgccagctgc gccagcaccg

6181 gatcgctttc gcagacactg cgcatgtcag ggtagggact gtcgccgatg cgatccagca

6241 gcgccctcaa cgccagttgg atgtcggcgc gctcctgata ggggcgatgg tgctgcaatc

6301 cctcgagctg gcgtagcaat gcccggccga tcgcatcgtc gagcgcttcc gtgtccgtct

6361 gtgctacggg gacatcgcaa tcgagcagac ccagtaagcc gaccgactcg ccgtgcatgc

6421 cgaagtgctg ggcgatggcg taggcgagca gtcccccgga ggaatagccg agcagatgat

6481 agggaccatg cggctgcacc gcctggatca gcgcggccgt ctgcgccgcc agcgcctgca

6541 tcgtggccgg taacggatcg ggccacggta gggcatagat cgggatgtcc ctgtcgaggt

6601 gtgcggccag ttcgaatgcg taggtgatat cgccgacacc ggtcggtagc acgaacaagg

6661 gcgagcgagt gccgccacga cgggccgcca gcgcgtgcgg tcccggtgtc tgcgctgctg

6721 cgccggtcag acgcgctgcc tgcgcgcaga gggtcgggca gtcgtagacc atctgcacgc

6781 tcagttccag gccgctgcgc ttggccgcgg cgatcagccg caccaccaac aacgaatgtc

6841 cgcccaactc gaagaagtgg tcgttgcgac cgatctgccg ctgtcctagc accttggccc

6901 agagtttggc cagcgcacgt tcgatggcat tggccggcgg cacataggcg ttggcgtccg

6961 gtagcgccgc ctccggtaac ggcaaggctt ggcgatcgag tttgccgttc gcattcaacg

7021 gcagcgcgtc caactgcagg tagacgctcg gcaccatgta atcgggcaac tgtgcagaca

7081 gctgcgtgcg caggctgtcg tcagcacggt ctgctgcagt gccgacccaa taggcgacca

7141 agcgcacatc gccacggcca tcgtcgtgcg cgatgacgac agccgcgcgc acgccgtcgc

7201 aacgaagcaa cgcggcctgg acgtccgcca gctcgatgcg aaagccgcgc agtttgagtt

7261 gaccgtcgtt gcggccgacg aattcgagcg caccatcggc gcgccagcgc gccaggtcgc

7321 cggtgcgata catgcgctga ccggactgct cggcaaatgg atcgggtacg aaccgctcag

7381 cgctcaggcc cgggcggtgc agataaccgc gcgccacctg gatgccgccc accatcagtt

7441 cgccgatgac gccggccggc accggccggc accgatgatc gagcaaggcc aatcgcacgt

7501 tggctaatgg cgtgcccagc gtgggcagca gcgcgtcggg gcggatcgct gccacggtga

7561 cgtcaaccgt gcattcggtc gggccgtaga cattgaagaa ggcgatctgt gtgcactggc

7621 tcaggcgctg ccacatggcc ggcgcaatcg cctcgccacc gatcagcacc gtcttcggca

7681 cgccgtccgt tgcctcgagc aggccgttgt cgagcagcag ctgcagttgc gccggggtgc

7741 aatcgagcac atccaggcgc gatcgccgca tccaggccag cagcgcctcg caatccaggc

7801 gaaccgtctg cggcacgatt gccaggcggg cgccggagag caattgcacc cacatcttga

7861 ccgaggcgtc gaacgacagc gatgcgttga gcgatacgca tggcggcgcc gatgcggcgt

7921 ccgcgtgcac gaccggatgc aaggcccgcc agagattgac gaccgcgtcg tgctgaacca

7981 tcaccccctt gggacgtccg ctggagccgg aggtgtagat cacatacgcc aggtggtgcg

8041 ggccgaaccc tggcgccggg tcggggttgc gctcgggctg ctgcgcccac gcgtcactgt

8101 cgtccagcca gaccagcggg atcctgccgt gcggcaatcg cgcgctcatc gcggtgtggc

8161 tgagcacgca ctgcggtgcg ctgtcttcga gcatgaaggc caggcgttgc gccggatagc

8221 tcggatccaa tggcacgtaa gcggcgctgg ccttgaggat cgccagcacg ccgatcagca

8281 tctccggccc gcgctccagg cacagtgcca cgcgcgtgtc cggccccaca ccgtgcgcga

8341 tcaggtggtg ggcgaggcga ttggcctgcg cgttgagctg cgcataggag agcgcacgct

8401 cctcgaacag caccgcggtg gactgcggat tgcgcgccac ctgctgttcg aataggcgat

8461 ggatgcaatc ggcccgcagc gctggcagcg gtgctgcatc gggggcacgc ggcgttgcaa

8521 tgtcctgttg cggcaagacg tccagcgcat gcagcggggt ggccggcgcc tggcacaagg

8581 cgctgtccag tgcggccaag gcctgcacca tcatccggcc tgcgcgggca gcgccgaccc

8641 gaggcatggc ctggacatcc aacgagaagc cgcctgcatc gccgtgatcg ttgaccgaca

8701 gcaacagcgc gtagtgggtg cgttcctgcg ccggcagcgc ttcgatgccc tcccagccag

8761 ggtcggacga gtggccgtcg ccggcatggc ggtagttcag cacggcagtg aacaggggcg

8821 ccggcgactg cacgccgctg cagcgttgtg ccagcgccaa cggcgcatgc tcatgacgca

8881 gcaaccgggc aagccggtgt tgcacatcgt ggatcgcctg ggccacgccg gttcggtcgc

8941 agtccacccg cagcggcaag gtgttgagga acatgcccag cacccgttcg gcaccatcgc

9001 cggcccacat ccggccgaac agcaccgtgc cgaacaccac cgtgtcgcga ccgctgctgc

9061 gtgccacgat cagcgcatag gccagatgga acaggctggc gacgctgacg cccaggcgcc

9121 cggcatgcgc acgcaggttc tgggcaagcg tctggctcag ttgcagatgc gcgtgctcgc

9181 tatcgatcga cgcgtcggcg ggggtaggca gtccgaatgg tgcggtgggc gcatcgagat

9241 cggccagcat ctcggtgaag aacgcctggt ggtccgactg cggcacgccc aactgcgcgt

9301 gagcgatgaa atcgcggaac ggcagcgccg gcggcaggcg gtcttggcga ccggccaggc

9361 aggtctggat ctccgccatc gccagttcca acgtggtgtg atccatcacc aggtggtggt

9421 gctgcaggcc cagcaaccag cgaccgttgc ggacgtcttg gacgacatgc gcacgtaaca

9481 ggggggcctg ctgcaacggc atcggcgtct gcgccatgtg ctggcgcagg cgttcgaccg

9541 agccgttcca ggcgtgttcg tgcaccgcga gcggtgcatg gcgccagacg acctgcaccg

9601 gtgcgcccag acgctcccag acgatggcgg tgcgcaggat gtcgtgacgg gcgatcaccg

9661 cctgcagcgc ggcaagcacg tcgtccagac gctcgcgcgt atcgacgcag agcagcgtgt

9721 gttgcagata ggggtcgcgc gcagtgtcgg ccaggtggtg gaacagcaat ccctcctgca

9781 gcggcgccaa ctgatagatg tcctggacgt tggcggcacc gccatcgacg ctggtcacca

9841 ccaggtcgat ctcggcctga gtcagctcga ccagcggcaa cagctccggg gtgagctggc

9901 agcagtccgc gccgatgcgg ttgcgcggca cgaccacctc ggtcgccgtt tgcagcgtgg

9961 ccgccaggcc ccacagcgtc ggtgcggcga agatgctgcg cacgtccacc tgccagccat

10021 gcctgcgcag ccgctcgatc agcgtgaccg ccaacagcga gtgcccgccg agcgcgaaga

10081 agttgtcgtg gcgaccgacc cgtgcaacac cgagcaactc gcaccagagc gtggccaggg

10141 tgtgttcgag cagtccttgc ggtggggcgt aggcgtcgct ggcgaggtcg tcgctgctgg

10201 gtgccggcag tcgctgccga tcaagcttgc cgttggcggt cagcggcagc gcatcaaggc

10261 agacatacgc ggcgggcacc atgtaagccg gaagccgggc gctcagcagg gcgcgcagcg

10321 actcgggggt gacgaccgcg ctggcgacga cataggccac cagtcgcagc tcgccggcac

10381 ggggcgcgtc ggtcagcacg agcgcttcgc gcacgccggg gcaatcgcgc actgcggtct

10441 cgacgtcggc cggctccacc cggaacccac ggatcttgac ctgatcgtcg ttgcggccga

10501 ggaagtcgag cgtgccgtcg ctgcgccagc gcgccaggtc gccggtgcgg tacatgcgct

10561 gaccgggcac gctggcgaac ggatcgggga cgaagcgctc ggcggtcagc gccggcagat

10621 gcagatagcc ctgcgccagc tgcgcaccgg caagatgcag ttcgccgcgg gcgccgaccg

10681 gcgccggttg gccgtaacgg tccagcacgt aagcgcaggt accgggaagg ggccgcccta

10741 gcggcagacg ttcgctgccg ggttcgaccg ccgcgaccgc ctggatcacg ccgaagaccg

10801 tggtttcggt cgggccatag caatgcaaca ggcgctgcgg gggcgcctgc gcgagcagat

10861 cggcgatggc gcgcgcatcg acgcggtcgc cgccggtcag caagcagcgc agtctcggca

10921 gccagccagc cagcgcgcgc caatagccgg gcaccagccc tgcagtcaga tgcaggacgc

10981 tgaccgcgtg cctggccaga tgcgcggcca gggccggcgg atcgagcagc accgcctgct

11041 cgacgactac gatcgccgcg ccatgcagca acgccgccca gacttcgaaa gtggctgcgt

11101 cgaatgccgg gttggccaga aacgcgaccc ggtcgtcgtg ctggatctgc gcatgtccgc

11161 cgcccatcgc gaaggtggtc actgcagcat gtgaaaccac cacgcccttg ggcgtaccgg

11221 tcgacccgga tgtgtagatc acataagcgg gaccgagcac gggtaccgcg accggcggcg

11281 ccagggagtg cgccaccggt ggcagacggt ccagatccag gcgcggcagt tccagcaccg

11341 gcactgccac gcgactgtcg ctgagcacgg cacgtgccgc gcagtggccg accaattcgc

11401 ccaggcgttc ggccggatgc gccgggtcca gcggcacgta ggccgcaccg caatggagca

11461 ccgccaattc ggcgacgatc agctgcgcgg accgtggcag caccacggcc acccgatcgc

11521 cggggcgcac gcccgcggcc atcagctgct gcgccacccg ctgcgcctgg ctgagcagat

11581 cggcgtaaga cagcgtcgcg gcctcgctca cgatggccgg ggcatcgggt gtcagcgcgg

11641 cccgctgcgc gaacagtgcg aacaggcttg cctccggcaa ctgctgcgtt ggcgcaacaa

11701 cgcccatctc cagcagttgg cgatgctctt cggacggcag cagtggtaag cgggcaacac

11761 gctgcgtgtc gcctgcgcac attgtctgca gaagctgcac gaacagcgcg acgtggcgct

11821 ggatcgtgct gcggtcgaac agtgcgcagg cgtagccgag cgtgccgaca atgcagccat

11881 cgtgttcctg caggcccatg ctcagatcga acttgatcgt ctgcatttgt agatcgatgg

11941 cctgcagttg caaacccggc agctgcagtt ccggttgcgg cacgttctcc caactcaaca

12001 tcgcctggaa cagcggatgc gcgcccgggt tgcgctcggg gttgagcgtc tcgatcagtt

12061 gctcgaaggg cagatcctgg tgcgtctgcg cggccaatgc ggtagcgcgc acttgcgcta

12121 gcagggttgc cacggtcggg ttgtgcgaca gatccacccg cagcgcctgg gtgttgacga

12181 aaaagccgat caacggctcc agttcggcat gggtcctgtt ggcgaccggc gtgccgatca

12241 caacatccga ctggccggac aggcgcgcca gcagcactgc ccaggcggcc agcacggtca

12301 tgtacacggt cgtgccgtga cggtggccga gctgcttgag cgctgcgctg tgggcgggct

12361 ccagcgcgaa tgcgaccgtg tcgccgacgt agccctgctg ggccgggcgc ggtcggtcgg

12421 tgggcaagtc cagcaaggtc ggcgcatctt gcaggtgcgc gcgccagaaa tcgcgctggc

12481 gctgcagcgc ctgaccgctg atccgctgcc gctgccaact gctgtagtcg gcgtactgca

12541 cccgcagcgg cggcagcggg tcgtcgcggc cttgcaggaa tgcagcgtac aaggcgccca

12601 gttcgcggac gaacacgccc atcgaccaac cgtcggagac gatgtggtgc atggtcacca

12661 gcagcacatg ctcctgctcg gcaagacgca gcaggcggcc gcgcagcaac tggccctgtt

12721 gcagatcgaa gccggtgccg gcttccctgg ctgcatggtc gcgggcggcg gcttcgggat

12781 cgggcatgtc ggtcaggtcg atacactgca gcggaaagcc gctccgcagc gtttcgatcc

12841 gttggactgg cgtgccgtct tccacgccga aacgggtccg caacgcctcg tgccgatgca

12901 cgatccggtc cagtgcgcgg cgcaacgcca agctgtccag cggaccgctc aagcgcacgc

12961 ccgccggcat cagatacgcc agctggctct gcggatcgaa gcggtccaga aaccacaatc

13021 gttgctgggc gaaggacagc ggcaatggcg cgctgcggtc taccgccacg atgggggacg

13081 attcgatgtg tgcgtcgctg ccgccactgc gctccagaca caaggccagt gcctgcagtt

13141 gggtgtgggt gaagatgtcg ccgatctgta gctccacgcc caggctgtcg cgaacccgcg

13201 caatcagttg caccgccagc agcgagtgcc cacccagatc gaagaaatcg tcgtcgcggc

13261 tgacccggtc gatgcccagc aattcgcacc acagctgggc cagggtttgc tcatgcccac

13321 cctgcggcgc ggcgcccgcc ggatcgctgc ggtgttcgtc gtccggcgcc ggcatggcgc

13381 ggcgatcaag cttgccgttg gtggtgagcg gcagcgcgtc caggcgcaca tagcgggccg

13441 gcaccatgta gtccggcagt cgcgtcgcca catgcgcgcg cacagcctcc acgaccagcg

13501 agggcgttgc cacatacgcg accaggctgc gttcgcccgg ccggtcttcg cgcaccagca

13561 ccgccgcatc ctgtacgccg ggcgccgtcc gcagcgcggc ctcgatttcg cctggttcta

13621 tacggaaacc gcgcagcttg acctggtggt cgtcgcgacc gacgaacagc aacgtgccgt

13681 cctcgcgcca acggacccga tccccactgc gatacatgcg cgcgccggcg accggcgcga

13741 acggatcggg aacgaaccgc tcggcggtca gatccggacg gcccagatac ccgcgcgcca

13801 gttgcgcacc cgcgatatgc agctcgccgt tgacgccgat cggcagcggc tgttgccggc

13861 cgtccagcac gtgcaggcgc acattggcga tcggcccgcc gatgtgcagc gcgtgcccgg

13921 ggaccaccgc accgaagctg acaccggaca cggtttcgct cgggccatac gcgttgagca

13981 gttgcacgcg ctcttccgcc agcacatgcc agcgcgcgac atgcgcaggg tcgagcgcct

14041 cgccgccgca caccaccagc cgcaaggttg ccggcagagg tgccgcgctg tcggtttgct

14101 cggccatcca ggcatgccag taggcggtcg gcaggttcag cacgctgatg cgttgccgat

14161 cgatcgccct gcagaactcg gcgatggccg gcaagcgctg gcccggcaac agcaccagcg

14221 tcgcgccgct ggccagcgtg gcgaagattt cgtccacggc gatatcgaaa ctgggcgagc

14281 tgcactgcag gacccggtcg ccgctgtaca agctgtagcg tgtggcagcg gccacgcagt

14341 aattgcgcaa gccgccatgg tccaccgcca cgcctttggg cgtgccggtc gagccagagg

14401 tatagatcac ataggccagg tgctgcgtgc gcaacgccgg caccgtaggg gcggtgtgcg

14461 gcagctgcgc ccaatccggc tgcggttggt ccagcaggat cgtcggcatg ctggcctgcg

14521 cgcccagctg ggccgcaccc tgcgcatcgg tgagcagcgc gcagggcgcg ctgtcggcga

14581 gcatgaagcc cagacgctgc agcggataag caggatccag cgggacgtac gcgccgccgg

14641 ccttgaggac cgccagcatc gccacgatca gttcgatgct gcggcccagg cacagcgcca

14701 cccgctggtc cgggccgacg ccgaggtcga tcaggtggcg cgccaggcga ttggcctggg

14761 cgtccagttc ggcgtaggac agactgcgcg ctgcatcgac cacggcgatg gctgtcgctg

14821 cctgtgccgc gtggcgttcg aacaaggtgt gcaccccggc aacctcggcg accggggtgt

14881 cggtggcatt gaagtcttgc agcagttgca gttcctgcgg cgcggtcacc gacagcgcgg

14941 acatcgccgc atcgtcgtgg gcggccatcc ggccgagcag ggtcagcaac tggcggatgt

15001 ggcgctggat gctcgagcgc gcgaacaagg ccgtggcaaa ccccagactg ccgacgatgc

15061 agtcggcgtc ttcctgcatc gacagttcca gatcgaactg cgcggtgcgg ccgctgcgct

15121 ccagcgtctc caatgtcagc tccggcaacg ccagttcgcc ttgcggggcg ttctgccagg

15181 cgaacatgac ctggaagacc gggttgtgcg cattgctgcg taccgggttg agcgcttcga

15241 ccacatgctc gaagggaatg tcctggcatg cctgtgcggc caatgcggtg gcgcgggtct

15301 gcgccagcaa ctcggccacg ctcgggtcct ggcgcagatc gatgcgcagg gccagcgaat

15361 cgacgaagag cccgatcaag gattcgagtt ccgcctgatg gcggttggcg atcggcgtgc

15421 cgatcaccac ctcgcgttgc cccgacaggc gtgccagcag tgctgcccaa gccgccagaa

15481 tgaccatgaa caaggtgacg ccatgccgcc gggccagcgc tttcagcgcc gccgactggg

15541 tcgcgtccag gcggaagggc agcacttcgc cgcagtggtc cggccgcgcc gggcgtggaa

15601 aatcggtggg caggtcgagc agtgccggag cgccctgcaa gtgtcccacc cagtacgcca

15661 gctggcgttg cagccgctcg cccgcgatcc agcggcgttg ccacagggcg aagtcggcat

15721 actggatcgg caggggtttc agcgggtcgg ccaggccttg cgtaaatgcg ccatacaacg

15781 cggtcaattc atcgaccagc acggcaccgg accaggcgtc ggcaacgatg tggtgcacgg

15841 tcagcagcag cacgtggtcg tgctggccca ggcgtaacag ccgcccgcgc agcaatggtc

15901 ccttggccag atcgaagggc gcctgcgctt ccgcttcggc atgcgcgtgt gcggcctgca

15961 gtgggtcgct gtcgctgctg aggtcgtggt ggagcagggc acatccgcgc tcggccggcg

16021 caattacctg ttgcggctgg ccgttgaccg tgacgaaaca ggtccgcaac gcttcgtgcc

16081 gttcgacgat gcggttcagc gcacggccta gcgctgccac gtccagcgca ccgcgcagcc

16141 gcagcgcgta cgccagcgaa taggcggtgc cgatctggcc atgcatgtcg atgaaccaca

16201 gacgctgctg cgcgaacgac aatggcaacg gaccgctgcg tggactcggc acgatcgccg

16261 gttgcttatc cagcgacgcg ttgcttacgg catcggccaa atcggacaga cgcgcatgtg

16321 cgaagatctc ggccaggcca atgaccaagc caagccgttg ccgcacgcgc gacgccaatt

16381 gcaccgccag caacgagtgt ccacccaagg cgaagaagtc gtcgtggcga ccgacgcgtt

16441 cgacgccgag cagcgcgctc cacagcgtgg ccagcacggt ttccagttcg ccttccggtg

16501 caacataggt cagtgttgcg agcgcatccg cctccggcgc cggtaatgcg cggcgatcga

16561 gtttgccgtt ggcggtgagc ggcaacgcgg cgagtggcac ataggcggca ggcagcatgt

16621 agtcgggcag gcgtgcaccg agttgggtgc gtaatgcctc tgctggcaaa ccaatgtcgt

16681 cgtcgccgac cacgtaggcg accaggcgct tgtcgccggt gctgtcttcg cgcacgacca

16741 ccgcggactg acgcacgccg tcgcaggccc gtaaggccga ggcgatctcg cccagttcga

16801 tgcggaagcc gcggatcttg acctgctcgt cgttgcggcc gagatagtcg aggctgccgt

16861 cggcgcgcca acgcgccagg tcgccggtct tgtacatgcg ctcgccggga tgcgtggcga

16921 acggatcggg gacgaagcgt tcggcggtca atcctgggcg gccaagataa ccgcgcgcca

16981 gaccggcgcc ggccacatgc agttcgccgg ccacgccgac cggcaggcat tgcgcatccg

17041 ggcccagcac ataggcgcgc agatccgaca gcggcgtgcc gatcgggctg tggccactgc

17101 gctgtacatc ctgttccgtt agcgcatgcg cggtcacatg caccgtggtt tcggtgatgc

17161 cgtacatgtt caacagcgcg gtgcgctggc cgtgtgcggc aaaccatgtg gccacgctgg

17221 ccggttgtaa ggcttctcca ccgaagatca ccaggcgcag gtggtgctgc acctggctat

17281 gtcgctgcgc ctcgatcaac gcctggaacg cgctgggggt ttgattgagc acgctgacgc

17341 gctgctggca gagcagggca tggaacgcgg ccgggtcacg cgcaatgcgt tgcggcacca

17401 cgaccagcct gccgccgtgg gcgagcgcgc cccacagctc ccacaccgag aaatcgaagg

17461 cgcaggagtg gaacagtgtc cagacatcgt cagcgctcgg cgcgacgtgt gcgcgggtgg

17521 cgtgcagcag gcgcaccacc tgggcatggg cgaccaccac gcccttgggc gtgccggtgg

17581 agccggaggt gtagatgacg taagccggat gctgcgacag caggtcgctg cgcatcggcg

17641 cgtgactggg ccgaagcgcc caagcatcgg catcgtccat caacacggtg gcgatgcctg

17701 cctcggtcgg caacgtggca gacaacgcag cgtgggcgag cagcagacgc ggctgtgcgt

17761 cggccagcat gaaggccagc cgctgcgcgg gataggcctg gtccagcggc agataggcgg

17821 cgccggcctt gagcacagcg aggatggcca ccaccagatc gataccgcgc tgcaggaaca

17881 gcgccacgcg atcttcgggg ccgacgccgt gcgcgatcag atggtgagcg agctggttgg

17941 cgcgggcgtc gaggtcggca tagctcagcg ccaggtcctc gtgcaccacg gcgatagcgt

18001 ttggcgtctg cgccacctgc tgttcgaaca cggtgtgcag gcagtcatcg tcggcgaaca

18061 cacgctgggt ctgtgccagc gtctgcagcc actgctggcg ctcgaacgcg ctcagcagcg

18121 gcagccggtc aacgcgagcg gtagcgtcgg cgaccatgcc atgcaacaac gcgatgaatt

18181 gatccagatg acgctcgacg ctgctgcgct cgaacagcgc aatcggatac ttcatgcatg

18241 ctcgtagtcc gcccgccgac gcgtgcatgg acagcatcag atcgaacgcg cacatgtccg

18301 acgcgtcggg caacggctcc agctgcagtc cgggcaggtg cagcgtttgc tcgccgctga

18361 agccatctag actgaaaagc acctgaaata ccggcgtatg actcaggctg cggttcggct

18421 gcaacgcttc gacgacgcgc tcgaacggca gctcctgatg atcctgtgcg ttcagcaccg

18481 ccttgcgtac atgcgccagc cattgcgcca tgctgggcgc gccggacacg gtgagccgca

18541 gtgccagcgt attgatgaaa caaccgatca gcgcttccag gtctggatgc gtccggccag

18601 ccaccggcgt cccgatcacc acatcgtcgg tggcgctgat gcgtgacagc agcaatgccc

18661 agccggccaa caacgtgacg aacaacgagc acccgtggcg ctgtgccagc gcattgagcc

18721 gctgccccaa ctccgctggc agcaagcgct cgacgaccgc gccgcggtag tcctgcagcg

18781 ctggcctggc acggtcggtc ggtaacggta gcagggccgg cgcaccagcc aattgcccgg

18841 tccagaagcg cagttgcgta tcgagcatgt cgccctgcaa ctgctcgctc tgccagatcg

18901 catagtccgc atactggatc gacaatggcg gcaatggatc gggctgccgc cgcaggaaca

18961 cggggtacaa ggcttccagc tcgcgcaata gcacgccgat cgaccagcca tcgcaggcga

19021 tgtgatggaa ggtcagcagc agaacgtgtt cctgcgctgt caggcgcaac agacggccgc

19081 gtaccggggc gcccagttcc aggtcgaacg gccgccgggc ctcctgctgc gcgatgcgcg

19141 cgaccgcggc tggatctgca gcgcacgttc ccacgtcctc atgcacgagc ggaaagccag

19201 acggcggttg gatgcgctga cggatctgac cgttgacgtc gaccaggctg gtgcgcagca

19261 cttcgtgccg caccacgatg cgatccagcg cggcctgcaa tgcgtggcta tccagttcgc

19321 cgatcaatcg cacgccgttg gcgatgtggt aactgatgga cgcttgcgga tccagccggg

19381 cgagcaacca catgcgcttt tgcacgaacg aggcgggcaa ggtcttcgca aacttatcca

19441 tactgcacgc gctccttgaa caggcgcgcc cggcggcgca acgcccgctc ggcaggacgt

19501 tgcggacgac acggcatttg gctggcgcat tggagcagga tcatcgatgt cgggcgttgc

19561 acgtacctga ctcaacttgc ggcgtagggc gatttttagt gcgccgggca agtgcgcgct

19621 tgactggcag tgactgcgat ttgcctttgc tggtcgtgtc tacggcaact gcgcaacgac

19681 ttcgccctgc cgccagtccg cgcttggacg cgtgatgggc agatccaggg acgcctgggt

19741 gcgctgttga gtgacgctga caggcaactc atcggcgcac acgagcaagt gccccggcag

19801 ggcgtcgacc accatgccac gcatcgctgt cgccccaagc gtgttgtgta tgttgaggtg

19861 agtgcgaagc ggtcagtggc acgtcgaaac aacgcgtgcg ccgacgctca acgggcatca

19921 cacgcactaa ttgtttggcg atccatcctg ggcaacgctg gaacctggac acgcatgaat

19981 ttcaatgcgg atgttgcatc gacggacgcg cggccccata tcgcgtcgcg cattcgtggc

20041 gtcggtaccg ccacgcctgg cagtcgctat tcgcagccgg atgtgctgga acgtttcggc

20101 atcgaagatc agcgccggcg gcaggtgttt ctgcgcaacg gcatcgactc gcgcagcctg

20161 gtcctgccac ggcgcgaaca gaccgaagcg gcaccgcgcg aaacgcaggc gcagttgctc

20221 gacaagcacc gcgaaaccgg cctggagatc ggcgagcacg cactacggcg ctgtctgcaa

20281 tcgatcggtg ctccactcga ccaggtgcaa tacctgtgct gcgtcaccac caccggattg

20341 ctcacgccgg gcttcagttc gttgctgatc cagcgcctgg gattgcgtca ggactgcctg

20401 cggctggatg tggtcgggat gggctgcaac gccggcttga acggcttcaa cgcggcggtc

20461 aactgggcaa atgccaacac gggaaaactg gcgatcctgc tctgcatcga ggtgtgttcg

20521 gcggcctacg tcgacgatga ggggatcgaa acggcggtgg tcaacagctt gttcggcgac

20581 ggtgccgcag cgctggcagt gatcgccgac agcgccgatg cccatggcgg gccgcgcgtc

20641 tgcaagttcg ccagccaggt gattcccgag gcgctggatg caatgcgctt cgtctgggac

20701 caggcgcagg gcaagttcca tttccgcctg cacaaggacg tcccgtacgt ggtcggcgcg

20761 aatgcaccga ccgtgatcga ccgcctgctc gagggaaccg gtctgcgccg ccgcgacatc

20821 gcccactggc tggtgcactc gggtgggcgc aaggtcatcg acgcgatctc ggccaatttg

20881 atgctcacca gccacgacat gcgccacacc atcgaggtgc tgcgcgaaca cggaaacatg

20941 tccagcggtt cgttcctgtt ctcttacgca cgcctgctcg acgaagggca ggttcgtcct

21001 ggcgattggg gcgtgatgat gacgatgggg ccgggttcgt cgatcgagac cgcgctgctg

21061 tgctggtaaa ccacgcctga ttcccatttt cgatcctttg gatttttcat gagcgatcca

21121 gcaaacccga tgttgcatct cgtgcacctg aagatcgacg ccgccagcgc gctgagcgac

21181 gagcagatcc aacggctgac ccgcgcctgc gatgcggtcg aagacgctac ctcgccagcg

21241 cttctgatcc tgcatttgcg cggcactacc gcaccgtctg cggcggcgcg ctggccgggc

21301 acggtaagcc tggatctggt gggtcgctgg gaaaaggcga tgcgccgctt cgaacgtctg

21361 caaggtctgt cgctggcctg gatcgaggac acctgttctg ccggtatgtt ggatctgctg

21421 ctggcgacgg atcagcgtat cgccacgccc acggccagca tctgcatgca cggcggcgaa

21481 gccgcatggc caagcatggc gatgcggcgc atgagcaccc gtctggggat gagcgcggca

21541 cgcgccgtgt tcctgttcga aacgcagcta tgtagcgcgc gcatggcgca gttgggtgtg

21601 gtcgatcagc tcagcgacga tgtccagggc gcaatcgccg catggctggc gacgctggcc

21661 ggcgtcgatg tgcaggtatt gccgagtcga cgcttgttgc tgctcgaagg cgtcgcccat

21721 tcctacgaac aggcgctggg ccagcatttg gcggcctgcg atgtggagct gcggcgccgt

21781 cagctgcgcg ccctgcagca tgcgcctgcc gacggggcgc aggacgttcc tgcacaggcg

21841 gtttctgcat gaatccgccg gccgacgaca tggcgcaatt gcatgcgctg gccggctggt

21901 ccgggcaggc gctcgccgca ttgccgccgc ctgcgcagcg cgatccgcgg cagtcacagc

21961 gggcagccgc gatccacacg cgctgccggt ccgcacggat ggatttcatg cgccgtcatg

22021 gcggctggtt gtaccaaacg ctgacccacc actgcaccct ggcgctacgc ctggacgagg

22081 tcttgcacgc tgccgccacg cgtgtgcctg ggctgttacc gagccaggcg caatgggcgc

22141 acgagagcgc ctgcatccag tcggaaaagg aaggctggga gatcgaacag ggcattgtgt

22201 tatggggcgt cctgcgccag ccggagtgcg ggcaccatct catcgagagc ctgctgcggc

22261 cgactgcgcg cgcgctcgaa ttactagcag aattccgcag ccaggcgcag gtgcagatcg

22321 gcccgatcca tctgcagcgc cgcgatggcg ttgcctggtt gaccgtggcc aaccagaccg

22381 gcctcaacgc cgaggacaac gccttggtcg aggcgatgga agtggcggtg gatctggcct

22441 tgctcgatga ccaggtcgcg gtcggcgtgg tgcgcggtgg cccgatgcaa caccccaaat

22501 acaccgggcg gcgggtgttc tgctccggca tcaacctgca gcatctgcac caggggaaga

22561 tttccttcgt ggacttcctg ctgcgccgcg agttcaccta tctcaacaag atgcgtcgcg

22621 gcctgagctg gctgggcgcc gatgcgcgcg agcgcacgat tgaaaagccc tgggtggctg

22681 cggtagacgc cttcgccatc ggcgggggcc tgcagttgtt gctggtgttc gaccatgtgg

22741 tggccgagcg cgacgcgtat ttcgtattgc ctgcggccaa agagggcatc gtcccggggt

22801 cggccaatct acgcctgggt ctgcgtgccg gacaccgggt cggccgtgac atgatcctgc

22861 gcggtcgcat catccgcgcc gatgagcccg atgccgcagg cctggtcgac gacgtcgtcg

22921 acgccgacga catggacgcg gcaatcgccc gcgcggtcga gcagatgcgc agcgaagcgg

22981 tggcgcccaa tcggcgcatg ctccgcattg cggaagaatc gccggaggat ttccgccgct

23041 acatggccga attcgccctg gtgcagtcca gccgcatcta tgccgacgac gtgcttgcgc

23101 ggctgcggca gcggtgggct acgtgagcgc cctcaccgac gccgcattgg acgatctgca

23161 gccgccagcg ctggcgatgc tggacccgag ccaattgcat ccctgtctgg gcgaaccggt

23221 catgggagcg atcgattttc tcaacgaggt gatcgaccgc tacccgaatg ccatttcgtt

23281 cgcacccggc gcgccggcgc cggccttgct ggaaccgctc gacctcagcg cgtatctgca

23341 gacctatctc gcgcacctga cgcagaccga gggcctgagc gagctccagg tgcgccgccg

23401 cctgttccag tacggcccca gccgcggcat catcaatgcg ctgctggcgc aggcattgcg

23461 tcaggatcag ggcctggata tcgcgccgtc gagcatcgtg gtcacggtgg ggtttcagga

23521 agcgatgttt ctggtgctac gggcgctgtt tgccgcgccc ggcgacgtgc tggcggtggt

23581 gcagccgtgt ttcgtcggtg cgatgggcgc cgcgcgcgcg ctcgatattc cgctgctggg

23641 catcgatgag cgcgatggcc gcgtcgatct ggagcaattg catcgcgcct gtaccgagct

23701 gcacgcgcag ggccgccgcg tgcgtgcgct ctacgtggcc ccggatattt ccaatccctc

23761 cggcagtctg ctcgatcgcg aacaacgcca ggccttgctt gatgcggcag acgcgcacga

23821 tttctggctg ctggaagaca atacctacgg cttcaccgtc ccgcacgcgc agagcgtgcc

23881 atcgctgaag gcgatggatc gcaagggcag ggtgatccac atgggcacct tcgccaagat

23941 cgccttgccg ggcatccgtg tcggtttcgt gatcgccaat cagcgcgtgg ccggtcggcc

24001 gccacgcttg ctggccgatg cactggctac gctgaaaagc atgctgacgg tcaatacctc

24061 gccggtctgc caggcgatcg ccgccggcat gctgttgcgc agcggtggct cgctcgcggc

24121 gctgggcgcc gagcgggcgg ccttctatca gggcaagctc gcctgcctgc tggccgcgtt

24181 ggaacgcgaa ctcggcgatc tggccgccgc ccatccggcc ctgcgctgga atcggccaac

24241 gggcggcttt ttcgtctgtc tgcaactcgg tgtgcaggtc gatgcggatt tgctggagtt

24301 gtccgcgcgc gaccatggcg tgctgtggac cccgatgcgc atgttctacc tggacgacgg

24361 cggccagcac agtctgcgtc tgtcgtgcag ctatctcacc gacgcgcaga tcgacgatgg

24421 agtcaaacgc ctgcgccgct tcctgtgcga cccacgggtg ctaggatgaa cgacgcgccg

24481 gtgctgttcg aacgcgacgg ccatgtcgcc cgcatcacct tgaaccgccc ggcggtgctc

24541 aatgcgctcg atcttgccac ccatgccgcg ctcgccgaga tctgggacgc cttcgaggcc

24601 gacgatacgt tgtgggttgc gatgctcagc ggtagcgggg agcgcgcgtt ctcggtcggt

24661 caggatctga aggagcttgc cgcgcgcctg gctgatggcg cgccctcgtc atcgttcggc

24721 agccgggggg cggccggttg gccgcgcctg accgagcgct tcgacctgag caagccagtg

24781 atcgccaggg tcaatggtct cgctctgggg ggcggcttcg aacttgccct ggcctgcgac

24841 atcatcgtcg cctgcgacac cgccgaattc gccttgccgg aagcgcggct cggcctgatc

24901 cccggcgccg gtggtgtgtt ccgcctgacc cggcaattgc cataccgcag cgccatgggc

24961 tatctgctca gcggccgacg catcggcgcg gcgcgtgcac tggcgttggg cctggtcaac

25021 gaggtcgtgc cggccgcgca gctggatacg tgcgtggacg gctggttgtc cgatctgctg

25081 gcctgtgcgc cgctctcgct gcgcgcgatc aaacaagcgg cggccacgtc ggcgagcctg

25141 ccgttggccg atgcctttgc ggcccagtat ccgtgggaag aacgtcggcg ccacagccag

25201 gacagccgcg agggaccgct ggcattcgtg gaaaaacgcg cgccgcgctg gtgtggccaa

25261 tgagcgcggc gctgctgcag tcgcttggcc gcgtgttggc tgaggcaagg cgctcacttg

25321 cgatcgccgg agcgcggcca tgctgatcca gcgctatctg cgccgtcatg cgcgcatctt

25381 ggccggcgtg tcggtgctgt cggcagctag cgccggcacc tcgatggtgc tgctggatta

25441 tctcaacacc accgccaccg acgcgctgca gatcgatatg cgcgacgcac tgctgcgtgg

25501 tgcgctgttg ctggtcgccg cgctcgtggt gcgcctgctg tcggcacgtc tggccgcgcg

25561 agtcagcagc ggactgatgg ccgacctgcg cacggagttg tcggcgcgct ttctggaact

25621 gccgctggaa cggttgatgc accgcaagca tgcggtgttc ggcgcgctga tcggcgatgt

25681 cggacgcctg gcgcagatga tccaaatggg ccctatgctg ttgaccaaca gcctgctctc

25741 gctgggcggc ctgctgtacc tggcctgggt gtcgctgccg ttgttcgcgg tggtggtgcc

25801 tttcatcggc ttgtcgggcg cgctgttcta tgtgacccgg cgcttcaccg gcccggccta

25861 cgaccgcatg cgcaaggccg aggaaacgct caacggcttg ctgcgcacgc tggtggaggg

25921 caagaaggaa ctgacgctat tgccggcgcg ggccaggcac ttcgcacagg ccgagctgcg

25981 cccggcaatc gagcgcgccc gcagtacgca attcgacacc agcatgcatt ggggcatcag

26041 cgatgcctgc gccgagttga tcggttatgg ctgggtgctg gccgcgatcc tggccggccg

26101 ctatctgttc gacctgccgt cggccacgat cctgcagttc gtcatcaccg ggttgttcat

26161 cagcggcccg ctcaacgcgc tgttcgatct cggtgcccag gtgggcagcg catcggcgag

26221 tgtgcgccac ctgcgcgaga tgggactcga cgatgcgcca tccgcgccgt cccctgcagc

26281 agcggcgccc gaggtgatgc ggatgtggac cacgctgcgc ctggaacagg tggtctatcg

26341 ctatgccagc gacaacggcg atcactttca gttcggcccg ctggacttca ctctgcggcg

26401 cggcgaaacc gtgttcgtca ccggcggcaa cggcagcggc aaatcgacct tgctgctgct

26461 gctcagcggt ctgctgcgcc ccagcgaagg acgtatcctt gtcgacgggc atgcactgga

26521 aaccagcctg acgctgacca gctaccgggc gatgttcagt gcggtgttct tcgacttcat

26581 gctgttttcc catgtgatcg gttgtgacgc cgcgccggcc gatccagcgc aagtgcagga

26641 ctggctggag cgtgtggacc ttgctgcaaa ggtcgcgttc gacaaggccg aaggtgtgtt

26701 cgccagcgtg aatctgtcgc aggggcagcg caaacgtctg gccttggtgc aggcctgcct

26761 ggacgatcgt caaatcatgc tcttcgatga attcaccgcc gatcaggacc aggcatttcg

26821 ggaacgtttc tacaccgtgc tgttgccgga attgcgcgcg cgcggcaaga cgctggtgct

26881 ggtgacccat gatgcgggtt accgacacct ggccgaccgc gtgctgtcgc tggactacgg

26941 ccacgtggtt gcgccggagc caatgtgccc cccgccgttc cctgcggagg tgcgcgggcc

27001 ggcgctggaa gaagtacagg tgtccggcat gtcgccggag acctgagcaa aaggacacgg

27061 gcgcatcgaa cctgcggcca gcgcccggcg catcgcccga tgcgtgcaac acgccgccat

27121 tcattcccat cagaggacgt acagatgtcg atccaagacg acgagcaaga ccagatctat

27181 gtcgtggtca tcaaccacga agaacaatat gccttgtggc cggactacgc cgacgtccca

27241 aatggctggt cggtcgtgtt cggcccgaac ccgaagcagg cctgcctgga ctacgtcaac

27301 gaacactgga ccgacatgcg tccgaagagt ctgcgcgacg cgatggcagc ggcgggacat

27361 tgagtgtcgc gtcgtcctcc ctcctgacga tacatcgtct ggagggcagg gcggccagga

27421 aagcaagcca gacagcacgc acgtcagtgc gcgcagacaa cctcagccag caggcaggtg

27481 gagttccttc attgcgaaca gacgctcgcc ctcgatgccg cgtgcgcggc agtgcgcaag

27541 gaagcgatcg gcttcgcgca ggcccaagtg ttcgccggac tggttgatcg ccaggacatg

27601 attcaaccag ggctcctggc ccatcgcgta gacataggca cggcgtgccg gccaacgttc

27661 gatcagcgcg atggcctcgt ccgcatgcga gccgcggtcg cgccggctgc gatccagctt

27721 gggatcgatg cggcgggtca acagatgccc atagccccag ctcagcggcg acccttcgca

27781 ttccatgccc agaaacaagg cgtcgacagg accaagttcc gcagcgatgt gtcgatacag

27841 gctgggatcc agattgcagg aatcggcaag acaggcagcg ctgcggccct tgatgcgcac

27901 gtgatagcca gccttgccct ggatgtccag atcgctgtgt tcgccaagga acggcagcgc

27961 ggtaatcgcg ccatcggcga gcgcaatgcg ctcgaactcg tgcagctcga tcacccgctc

28021 gaacccgagc gcctgcagca tcagtttcag cgatggatcc tgcaggcgcc ggccggcgct

28081 atgcggcacc accacggtcc cgatccggtg gcgtagccgc agcaaggttt ccggtcggat

28141 gtggtcgctg tgcccgtggg tgatcagtac gtagtcgatc cgctcgggca gatccgccag

28201 ggtgtagcgg ggcaggtcgg tcggatagtc gtaggccacg aagggatcgg tgaggatgct

28261 gacctggtcc gtttcgatca acaggcatgc gtggccgaaa tagcggattc gcacccccgc

28321 cgcgggcggg cgatggcgct gcgccggcgg cgcggcggtg aacaacgccg caaaccccgc

28381 ccgctgcgac gcgccgacgc cgagctgttc ggccatgtcg tcgatgacca ccggcacgcg

28441 ctcggcgcgg aacaacgcat ccagccgcgc atcgtcccag ggcagggcaa gcaccggggt

28501 atcgggtgat tccaggaccg gcgtgctgcg ttcgtacggc agccagtcgc ggtcgacccg

28561 gccgaaggcg aactgttgca ggtcgcgtcg atagaaacgg ctgcgataca gcaggccttc

28621 gagcaagcgc aggccgggat gatcgttgag gtcgtagacc agctcgacca ggccggcaag

28681 ttcgccgggg acctgggtgt acagcgattc cagcgactgt ccggttgcct gctgccgcag

28741 cagatcctcc agcgcctgca gcgctgccgc cagtgccaat gcatcgcctt gcgccgcccg

28801 ggtgcgttcg agcagctgcg cgatctcggc ggtcctggga gacggataat tgatccacgg

28861 accgcccatc agggccgggt cgcgtgccgc ctgggcgtgc tgttcgggcg cgcgcagata

28921 cgattccagg atcggcagct gttgtttcgc caccaccagc gccgactgca ccggcggaat

28981 cagcatgtcc cacaagatcc agtggttcaa cagcgggcgc gcgaccgcat tggcgcgcag

29041 gtaacctcgt gtctcggaca tgcgtaatac ctgagttaga gcgtgatctc gcaacggccg

29101 aacagcagtt cggcatgacg tcctttcgca cgcgcagcct cttccaatgc gcgcgcctcg

29161 cggaactgcg gcaagtcttc cgaatacaga atgctggtga tgtgcgtcac ccacggttcg

29221 gcacccatcg cgtaaacgta gagccgctcg ggcaggaaga agtcctgcag cgcactggcc

29281 aacgcgcagt cgcagccgtc caggcggcgc gactggtcct cgccccgggt cagcatcttc

29341 tgcagcagcg ggccgtacaa ccacgtcgcc ggcgcgccga cgcattccat gccaaggaac

29401 atgcaatcga tcttcgggaa caacgccttg agttgcgcgt agcactccac catcggcggg

29461 cgcgaatcgg cgaacagcac acacaccttg ccgtccagct ccaccgcatg gaccagtttg

29521 gcgcgaatat ccagatcggc atgctcgccg tagaacggcg cgccgacgat tcttccctgc

29581 ggaaagcgca gctcctcgta gtcggccagt tccaccacct tggtgaagcc gcagtgctcc

29641 aggcacagct tcagcgaaat atcctgcaaa tcaccgccgc ctgcgcgccc caccacgacg

29701 gtaccgacac ggtgacggat acgcagcagt gcttccatca ccacatgatc ctggtgagga

29761 tgggtgatca gcaggtagtc gatcttggcg ggcagatcgt cgaaggtgaa atggtcgatg

29821 gcgcattcgc ccggataact gatcagcgga tcgatcagga tgctgacccc cgcaccttcg

29881 atcagcacgc atgcatggcc aaagtaacgc acgcgcacgc cttccggcgc ggggggggcg

29941 ggcagcggtg catcggtcag cagcgcacgc gcctgcgcac gcgtggtgcc ggccggcgca

30001 tggcgcacaa tcagatcgag cagggcatct gcatcggaca tcccgccgca caactggtcc

30061 cagaacggat cggcaaaggg cacgctcagc tccagcgaac cggcagccga tggcaactgc

30121 ggcgtactca atgcgaacgc acgctcctgt tcgctgacag gttccagcac cgctgtctgc

30181 agaccggtgt cgtaggcgtc gctggcatag aactgacgct ccatgaaacg cacaccaggc

30241 tgcttgctga catcgtagaa gatctccagc ttgtcgcgga tccgcgtatc gatcctggta

30301 tacagaccgg ccaggccggc ccccttggcc tcgttgtgca acacctcata gagtgcggaa

30361 atcgcctcgg cataacgtag ttggggcgca caacgctgca gagtctgatc gcgcagcttg

30421 cggatatcgg caacatcgcc ggcatggtcg ataaaggggc cgccacgcat gtcaggctca

30481 cgcaatgctg cgcggtgctg ctccggatcc tccaggaacg actccatcat cgccagatgc

30541 gaatgttcgg tatacagccc gaacgtcagc ggcgaaatga gatacggatg cgcgaaccag

30601 ccattgatac gcggaagcag acggacgtgc tcggagagcc gcaaacggtg ggatgtcata

30661 aggtagtgac cgcgcagagc atagccatga tgcgtcgcag tctgttttac agctgggacc

30721 gatgcttgct tgatcgtggc agcgacttgc cgcagcgcgc caatgcacgg cggctctcta

30781 ttcaaggcaa aacgcgtcac ggacaatgac gatcgcagcg gcggcgacag cccacgtttc

30841 atgatcaatg aggacatatg ctcaaatatt ccccgttgtt tgaaaaccaa aagggtccga

30901 cgatcatctc gccagcgagc gggcggcact cgctgctcga cgtcatcgtc ccggagatgc

30961 cgcattaccg gcaggaactc aacgaacgtg gcgccatcct gttacgcggc ttctatgtct

31021 ccgaagtgaa acacttcgat gcatttgtcg aaggtgtatc gcaacagcag tatcgatacg

31081 tctatcgctc cacgccgcgg accgagatca gcaaccgggt atcgacggca acgaactatc

31141 cggcgcgcct tgaaattccg atgcacaacg aaagcgcgta ccacacgacc tggccactgc

31201 tgttggcatt ctgctgcctc gaagcgcccg ccgaaggagg gcagacgcca atcgccgcca

31261 tgcgcgggat cactcgcaat attggccagg agctgcttga gcgactggaa gaaaaggggg

31321 ttgaatacat tcgcaattac cacccgaata tcgaccttcc ctggcaaacc gtattccaga

31381 ccgaagatcg atcgaaggtc gacgattact gcgcaggtag tgggatcgcg tcccactggg

31441 gcgccgatgg attgcttcgc accgcgaacc gagcgcaggg catcgcgttt catcccgtca

31501 cgtcagagaa attattcttc aatcaggcgc acctgttcca tgtctccagc ttggggcgca

31561 cacaggccca agccatgatg aatatgttcg gcgccgacaa gctgccaagg cacgcgcgct

31621 tcggcgacgg cacggaaatt tccgagcagg atctgcagaa aatccaacag gcgttctcca

31681 gcgaggcgct cctgtttcac tggcaacccg gcgacgtgct gctgctcgac aacatgaaat

31741 tcgcgcacgg ccgcaaaccg tacaaaggat cgcgcgccgt gttcgcagcg ttgatggagc

31801 ctagtcgctg aattcgcatg cctaacctgt gacttggccg ggagcgaggt gaaaagtcac

31861 aaccttaccg agcccggttg caataacgta attgaggaat tggacctcac ccagctgcgt

31921 gccctgggct atagcgtcag cgtcgtcacc tatggcgtgc gcttgtcagc cggcaagcac

31981 atcatggtgg ccacggcgtg gccctggacc tcgctcattc aggagaagga cgcacggctc

32041 tacaacatgg ccccggatgg gagcggcggc ggcggcgctg accgcaaccg ggtgcgaggc

32101 agggcagtct caaaattcaa gtgcaaca

LOCUS AFHK01000112 13792 bp DNA linear BCT 09-MAY-2011

DEFINITION Xanthomonas oryzae X11-5A Xo_X11-5A_contig_112, whole genome

shotgun sequence.

ACCESSION AFHK01000112 [AFHK01000000](http://www.ncbi.nlm.nih.gov/sites/entrez?db=Nucleotide&cmd=Search&term=AFHK01000000)

VERSION AFHK01000112.1 GI:332357378

DBLINK Project: [66097](http://www.ncbi.nlm.nih.gov/bioproject/66097)

KEYWORDS WGS.

SOURCE Xanthomonas oryzae X11-5A

ORGANISM [Xanthomonas oryzae X11-5A](http://www.ncbi.nlm.nih.gov/Taxonomy/Browser/wwwtax.cgi?id=1009853)

Bacteria; Proteobacteria; Gammaproteobacteria; Xanthomonadales;

Xanthomonadaceae; Xanthomonas.

REFERENCE 1 (bases 1 to 13792)

AUTHORS Triplett,L.R., Hamilton,J.P., Buell,C.R., Tisserat,N.A.,

Verdier,V., Zink,F. and Leach,J.E.

TITLE Genomic Analysis of Xanthomonas oryzae from US Rice Reveals

Substantial Divergence from Known X. oryzae Pathovars

JOURNAL Appl. Environ. Microbiol. (2011) . 77(12):3930-7.

PUBMED [21515727](http://www.ncbi.nlm.nih.gov/pubmed/21515727)

COMMENT Base Quality-A custom perl script was used to clean and remove

reads with low complexity and low quality regions. Low quality

regions were defined as an average quality score of < 20 over a

10bp window along the read or > 2 'N' bases in the read. The Velvet parameters used in the final assembly for X11-5A were a hash length of 31, expected coverage of 19.58x, and a k-mer coverage cutoff of 9.79.

Contact Dr. Jan Leach for source DNA or cultures (Department of

BSPM, Colorado State University, Fort Collins, CO).

##Genome-Assembly-Data-START##

Assembly Method :: Velvet v. 0.7.53

Genome Coverage :: 70x

Sequencing Technology :: Illumina GAIIX

##Genome-Assembly-Data-END##

**Annotation was performed by Royer et al. (current manuscript)**

FEATURES Location/Qualifiers

source 1..13792

/organism="Xanthomonas oryzae X11-5A"

/mol_type="genomic DNA"

/strain="X11-5A"

/host="Oryza sativa cv. Lemont"

/db_xref="taxon:[1009853](http://www.ncbi.nlm.nih.gov/Taxonomy/Browser/wwwtax.cgi?id=1009853)"

/country="USA: Texas"

gene complement (45..1085)

/gene="ortholog of XOO_3096 "

CDS complement (45..1085)

/gene="ortholog of XOO_3096 "

/codon_start=1

/translation="MHDVAFEQALRSGSLFKDEEHLREWRALRAPYDTRLSKIFTLRHVQRRSEWSPVRMLTATFLHGSFDHLPGNMLFLLALATLLEGAIGSGWFLVLYLLGGFRASLASLWWRWGEPGGGLGASGAIAALMGAFCVVWGRRKVRFFYWFFVIFNYARGPAILLLPLWLGWELYSLASSDNGSVAFEALAGEQPHNLDVALARYRAARHAGRSQDSLRHAEVLLTLQTHSAERTRVQLAVADELGKVKIAYGLPARRALIAACVRNGLLADAERLLKEGELGTPRDELAQQWFVRALRYGELQDSAQCTRLLRQVLDQFPEQGTGEKSALFARERVRGFSLMHAVRMSRG"

gene (1491..1994)

/gene="greB; ortholog of XOO_3097"

CDS (1491..1994)

/gene="greB; ortholog of XOO_3097"

/note="Transcription elongation factor, GreA/GreB"

/codon_start=1

/translation="MSRWRPPAEKSTALITPEGHARLKAELDDLWRVRRPEVVRALAAAAAEGDRSENAEYTYRKKQLGEIDRRVRYLSKRLEALRVVDTAPTDPNAVFFGAQVELEDADSGELLRYRIVGPDETDAGRGWISIDSPLARALLKKRVDDEFDAHLPAGKHTFVVVSVDYTSQ"

gene complement (2122..3492)

/gene="rimO ; ortholog of XOO_3098"

CDS complement (2122..3492)

/gene="rimO ; ortholog of XOO_3098"

/note="ribosomal protein S12 methylthiotransferase"

/codon_start=1

/translation="MSQLNPKVGFVSLGCPKALVDSERILTQLRVEGYDIVPSYDAADVVVVNTCGFIDSAVTESLDAIGEAMSANGKVIVTGCLGKRPEQIREAYPQVLAVSGPQDYQSVMAAVHAALPPRHDPFVDLVPDYGIKLTPRHYAYLKISEGCNHRCSFCIIPSMRGDLVSRPVDEVLREAERLVRGGVKELLVVSQDTSAYGVDLKYAERRWRDRVYQTRMKALCEGLSELGVWTRLHYVYPYPHVDDVIPLMAEGKLLPYLDIPFQHASPRILKLMKRPGAVEKTLQRVQRWKAMCPEITVRSTFIVGFPGETDAEFESLLDFLDQAQLDRVGAFAYSPVDGASANALPDPVPEEVKQERLARFMAKQAEISALRLEAKIGSVQQCLVDLIEDDIAVARSRADAPEIDGLVHIQNGGELGLKVGDLVDVEITDSDEHDLFGDALPANVVPQQGRALNLQMV"

gene (3761..4333)

/gene="ortholog of XOO_3099"

CDS (3761..4333)

/gene="ortholog of XOO_3099"

/codon_start=1

/translation="MVIQEIFGVNEHIRAVADDYAARGYEVLAPAFFDLEEKDVQLPYDQESVQRGLALANAVGLERAVEVVKSAATLLTRAGKVGTVGYCWGGSVALLSAIRLVLPSVSYYGGRNTQLLDETPKAPVMFHFGERDASIPPEAIQAHREKLPQMETFVYPTGHAFNRSIDPTHYDADSAERALERTLGFFAAHLG"

gene (4333..4764)

/gene="ortholog of XOO_3100"

CDS (4333..4764)

/gene="ortholog of XOO_3100"

/codon_start=1

/translation="MNGTTPGMPAGDFQLDPRLAADSVFVADGPLSQVRLMDDARFPWLVLVPRVADVSEWIDLDGGQQRLLLAEINQLSQLLRAEPAVSKLNIGALGNIVRQLHVHLVGRHPGDAAWPGPVWGSGSAQRFASDTLQQHVAAWAQRLR"

gene (4795..4959)

/gene="ortholog of XOO_3101"

CDS (4795..4959)

/gene="ortholog of XOO_3101"

/codon_start=1

/translation="MKSNVVAALILILVGLLFLANNLGWTNLSIGRLIATWWPAALVACGVGMLFGRGN"

gene (5536..5715)

/gene="ortholog of XOO_3102"

CDS (5536..5715)

/gene="ortholog of XOO_3102"

/codon_start=1

/translation="MNYRLIPALFLIVLGALFLLDNLGLAHMDVGNLIATWWPVFLIAAGVRQLLHYREKAAAT"

gene complement (5759..6325)

/gene="dcd; ortholog of XOO_3103"

CDS complement (5759..6325)

/gene="dcd; ortholog of XOO_3103"

/codon_start=1

/note="Catalyzes the formation of dUTP from dCTP in

thymidylate biosynthesis"

/translation="MSIKSDRWIKRMAEQHAMIAPFEPGQIKHDAAGQRIVSFGTSSYGYDVRCSREFKIFTNINSTIVDPKHFDPGSFVDVESDVCIIPPNSFALARTVEYFRIPRDTLVVCLGKSTYARCGIIVNVTPLEPEWEGHVTLEFSNTTPLPARIYANEGVAQMLFFQSDEVCETSYKDRGGKYQGQTGVTLPRT"

gene complement (6424..7272)

/gene="ortholog of XOO_3104"

CDS complement (6424..7272)

/gene="ortholog of XOO_3104"

/codon_start=1

/translation="MTGERRVAAHAVQGALAPHARIRNVIAVASGKGGVGKSTTAVNVALALCRLGARVGVLDADIYGPSVPAMLGLSGRPESPDNKSIEPLRAFGIEAMSIGLLVDQDTPMIWRGPMATSALTQLFNDTLWGDLDYLLIDLPPGTGDIQLTLSQKIPVAGAVIVTTPQDIATLDARKALKMFEKVEVPVLGIVENMAVHTCSNCGHREHLFGEGGGERMAAQYGVPLLGSLPLEIAIREQGDAGQPIVVAAPESSAALVYLAAATRLAEELGKRPRASIPISASLL"

gene complement (7619..8200)

/gene="IS"

CDS complement (7619..8200)

/gene="IS"

/note="ISXoo16 transposase"

/codon_start=1

/translation="MRGGIAGLHNALKPGRSRSTGEEEIATLINTALTRKPAGKTHWSRRSLADETGLSKSTVHRYLSLLGLQPQRSKSFTLSNDPFFIEKVRNIVGLYLNPPDHALVLCVDEKSQCQTLERTQPVLPMGLGYVEGIPHDYVRHGTTTLFAALDAANGTVLTQCKPAHRHQEFLALLRHIEANVPEHLDVHLAWVIGP"

gene complement (8805..10052)

/gene="daT"

CDS complement (8805..10052)

/gene="daT"

/note="diaminobutyrate--2-oxoglutarate

aminotransferase"

/codon_start=1

/translation="MIDPRQLFQHHESNVRSYCRAFDALFVRGAGSELFDVQGRRYIDFLAGCGALNYGHNDPDMTEALVAHLRGGGLAMSLDLHSQTKHDFIDNFVALILRPRGLPHRLQFTGPTGANAIEAALKLARKRTGRHNIIAFSNAYHGLSMGALAATGNRHHRMDLLHGGVTRLPYDDYLGSGLDSAALLEAMLDDPSSGIDPPAAIVLELVQGEGGLNVASTSWLHRVFATARRHGALTIVDDIQAGCGRCGNFFSFDDQALVPDLITLSKSLSGFGLPFSLLLVAPEHDSWRPGEHTGTFRGNNHAMLTATVALRKFWADDALRTQIDKRGQILAQALTRMTAHVPEARIKGRGMFVGLDLRTRERAASASAHAFGNGLVIETAGARGEVVKVMAPLTTPDALLREGLEILSDAVVSACG"

gene (10385..11518)

/gene="DH"

CDS (10385..11518))

/gene="DH"

/note="dehydrogenase"

/codon_start=1

/translation="MRTLTCLDDVQALARRRVPRMFYDYATTGSWSQWTVQANRRDFDACSLRQRIGCDVSQRSTATHMLGHAVTMPVALAPTGLAGLIHADGEILGARAAEAFGVPFVLSTMSICSLEQVRAGVRKPCWFQLYPLRDRGVVAALIERARVAGCSTLMVTMDVPFLGQRHADLRNGLSVPPRLRPEVLLDFLTHPRWALGMVRTRRRCFGNLVDYMPAGGDLQTLAQWTAHQFDASLGWDDLAWIRSRWGGALVVKGVLDAEDARQALQSGADALVVSNHGGRQLDGAVSSLRALPAIAELAAGRAEVHLDSGIRCGQDVLKALALGACGTYVGRAWLYGLGALGERGVARVLTLIQRELELTMALCGRTRIAEIDRSVVVD"

gene complement (11669..12985)

/gene="TA"

CDS complement (11669..12985)

/gene="TA"

/note="transaminase"

/codon_start=1

/translation="MARTQADQAAGGDRLDVMTFLNEASSRHPNAISFVSGRPTDSCFDLQAWMRAVPRFATHLAQRHHTSQAGALDLIAQYGTTQGIIEDLVVAQLAAYEGVPASIDRMLITAGCQEALQLCVTELCRQPNDVVLVRNPTYIGITGVADGHGIPIAAFNRDGRTDAEALTETLDALHAAGQRARLLYLVPDFDNPTGTVLPRDQREAIMAVCGAHGVLILEDNPYGLFAFGAERVPTMRALDNHGCVIYLGTYSKTLCPGLRVGFLVLPPTLFGSAAAARTLHQALTRRKSFATLNTSQVTQAIGGGVLLEHGCSLTPLIQAPRALYQRNRDRMLECLSQELGTLTPQIRWNVPAGGFFLIVTLPFVFAAEEAASCARECGVLVMPLSFFAIDSSCGQQVRLAYSNVATDKIAEGIARFARFVRHRLHAAGRLRQLAACPSL"

gene complement(13019..13648)

/gene="hypothetical protein, ortholog of XOO_2168"

CDS complement(13019..13648)

/gene="Hp"

/codon_start=1

/translation="MALNFSPAARMQATLLKSAHMRLPKWFSSAMPRITRWHTEHSMTDSVQQPDSALRYHKIDHIALAVDDLEASIALFRDQLGFVLTGRRHITGKTTGMRSAEMQHGDLIFVLCQGTEPQSQVSRLIAHHGAGVAHIALRVDDAHATARHLRERGLAFDTNVIEGQGLRQIFSERSELTGLSFEFIERNGEVGFQDASVNELFAQLERSGAY"

ORIGIN

1 tttgtggtgc gttgttcaga ggttccctag ctcttgcctg ctcaccctcg cgacattcgg

61 acagcatgca tcaagctgaa acccctcacc cgctctcgag caaaaagcgc gcttttctcg

121 cctgtccctt gctcgggaaa ttgatccagc acctggcgca gcaagcgcgt gcattgcgca

181 ctgtcctgga gttcgccgta ccgcagagca cgcacaaacc attgttgcgc caactcgtca

241 cgtggcgtac ccagctcgcc ttctttcaac aagcgctcgg catcggccag cagtccgttg

301 cgcacgcatg ccgcgatcaa ggccctgcgc gccgggagac cataggcgat cttgaccttg

361 ccgagttcgt cggcgaccgc cagttggaca cgcgtccgtt ccgcgctgtg cgtctgcagt

421 gtcagcagaa cctcggcgtg gcgcaggctg tcttggctgc gtcctgcatg acgtgcggcc

481 cgatagcgcg caagcgcaac gtcaagattg tgcggctgct cgccggccag cgcctcgaag

541 gcaactgatc cgttgtcgct ggatgccagg ctgtacagct cccaacccag ccatagcggc

601 agcagcagga tcgccggacc gcgcgcgtag ttgaagatca cgaaaaacca gtagaaaaag

661 cgcaccttgc gtcgtcccca gaccacgcaa aacgcgccca tcaaggcggc gatggcaccg

721 gaagcaccca aaccgccacc gggctcgccc caccgccacc acaggcttgc caggctggcg

781 cggaatccgc cgagcagata cagcaccagg aaccagccac tgccgatcgc gccttccagc

841 aaggtggcta acgcaagcaa aaacagcatg tttcccggca gatgatcgaa gctgccatgc

901 aggaacgtgg cggtcagcat gcgcactggc gaccattcgg agcgacgttg cacatggcgc

961 agcgtgaata tcttggacag ccgcgtgtcg taaggcgcgc gcaatgcacg ccattcgcgc

1021 agatgctctt cgtccttgaa caagctaccg ctgcgcagcg cctgttcgaa ggcgacatcg

1081 tgcatcgtca ggtgcgccag ataggcgatg cgctgcggtt taggcacgct gtccagtttt

1141 gcctggcgtg ccgcgcgcag cctggcgttg gcggtctgct gcaggtagcg cgcatgcgcg

1201 tcggcctcca gcgcgcccag tccggactgc acgtaatagt gcacggcgct cgctctcgac

1261 caggctgtcg tccttgcgct ggtagcccag gtagaccgcc acgttgatca gcaccagcag

1321 cagcgtcacc caggggatgt tctggcgcgg ccagggttcg tgcagcggca tgatcagcat

1381 gacaacgtcc gtgtccgaga atggcgcaaa gcgtcaccgt gcggcggcag atgcgcaagc

1441 acgtcatgcg cgctagcatc gcggccccgc cgttcgaatc tgcctgcccc atgagccgct

1501 ggcgcccacc tgccgaaaaa agcactgcgc tgatcacgcc cgaaggccat gcccgcctca

1561 aggccgagct ggacgacttg tggcgtgtgc gccggcccga agtggtgcgt gcccttgcgg

1621 cggcggccgc cgaaggcgac cgctctgaaa atgcggaata cacctatcgc aagaagcagc

1681 tcggtgagat cgatcggcgt gtgcgctatc tgagcaagcg gctggaagcg ctgcgcgtgg

1741 tggataccgc ccccaccgac ccgaatgcgg tgttcttcgg cgcgcaggtg gaactggaag

1801 atgccgacag tggtgagctg ctgcgctacc gcatcgtcgg cccggacgag accgatgccg

1861 ggcgcggctg gatcagcatc gattcgccgc tggcgcgcgc actcctgaaa aagcgcgtgg

1921 acgacgaatt cgatgcgcac ttgccggcag gcaagcatac cttcgtggtg gtctcggtgg

1981 actacacgag ccagtagccg cgtgatcgcc atccgctgac gtgtaggagc gcacttgtgc

2041 gcgatggggc gtcatcgata acgcctcttc gcgcgcaagc gcgctcctac ggaacgcggt

2101 gtcccgacac gtggccagtt acaccatctg cagattcaac gcgcgccctt gctggggcac

2161 gacgttcgcc ggcagcgcat cgccgaacag atcgtgctcg tcgctgtcgg tgatttccac

2221 atcgaccaga tcgcccacct tcaacccgag ctcgccgccg ttctggatat gcaccaggcc

2281 atcgatctcc ggcgcatcgg cgcgtgaacg cgccacggcg atgtcgtctt cgatcagatc

2341 aacaaggcac tgctgcacgc tgccgatctt ggcttccaga cgcaacgcag aaatctccgc

2401 ctgtttggcc atgaagcgcg ccaggcgctc ctgcttcact tcttccggca ccgggtccgg

2461 caatgcattg gcgctggcgc catcgacggg cgagtacgcg aacgcaccga cgcggtccag

2521 ttgcgcctga tccaggaagt ccagcaaaga ttcgaactcg gcatcggttt cgccggggaa

2581 accgacgatg aaggtcgaac gcacggtgat ctccgggcac atcgccttcc agcgctgcac

2641 gcgctgcagg gtcttttcca ctgcaccggg acgtttcatc aacttgagga tgcgcgggct

2701 ggcgtgctgg aacgggatgt ccagatacgg cagcagcttg ccttcggcca tcagcggaat

2761 cacatcgtcc acgtgcggat acgggtacac gtagtgcagg cgcgtccaca cgcccagctc

2821 cgacaagccc tcgcacagcg ccttcatgcg ggtctggtac acgcggtcgc gccaccggcg

2881 ctcggcatac ttcaggtcca cgccgtaagc ggaggtgtcc tgcgacacca ccagcaattc

2941 cttgacgcca ccgcgcacca accgctcggc ttcgcgcagc acctcatcga ccggacgcga

3001 gacgagatcg ccgcgcatcg agggaatgat gcagaagctg cagcggtgat tgcagccttc

3061 ggaaatcttg agatacgcgt aatggcgcgg agtcagcttg atgccgtagt ccggcaccag

3121 atccacgaag gggtcgtgac gcggcggcaa agcggcgtgc accgccgcca tcacgctctg

3181 gtagtcctga gggccggaca ccgccagcac ctgcgggtac gcctcacgaa tctgctccgg

3241 gcgcttgccc aggcagccgg tgacgatgac cttgccgttg gcgctcatcg cctcgccgat

3301 cgcgtccagc gactcggtca ctgccgaatc gatgaagccg caggtgttga ccaccaccac

3361 atcggcagca tcgtaactgg gcacgatgtc gtagccctcc acgcgcagct gggtgaggat

3421 gcgttcggaa tcgacgagcg ccttcgggca gccaaggctg acgaagccga ctttggggtt

3481 cagctgggac atggaagcag gagcctggga aacggaacgg cctggggcct gagcgaccca

3541 attatagcgg tgcgtgggca gcgctctgaa ccccgcgccg cgcaagcgag ttcatcttag

3601 cggaaccctc accttcagcc ccgccaatcc agtcgataat gcgcgcctcc gccataccgc

3661 agcaggagtt tccatgggtc actggaccac gctcgacacc ccagacggcc aggttgacgc

3721 ctggcacgcc acacccgctt ccagcccgcg cggcggcctg gtggtgattc aggagatctt

3781 cggcgtcaac gaacacatcc gcgcggtggc cgacgactac gccgcacgcg gctatgaagt

3841 gctggcaccg gccttcttcg atctggaaga aaaagacgtg cagctgccgt acgaccagga

3901 aagcgtccag cgcggcctgg cgctggccaa tgcggtgggc ctggagcgcg ccgtggaggt

3961 ggtgaaatcc gccgccaccc tgctgacccg tgcaggcaag gtgggaacgg tgggctattg

4021 ctggggcggc tcggtcgcgc tgctgtcggc gattcgcctg gtgctgccgt cggtgagcta

4081 ctacggtggc cgcaacaccc agctgctgga cgaaaccccc aaggctccgg tgatgttcca

4141 cttcggcgag cgcgatgcca gcatcccgcc cgaggcgatc caggcccatc gcgagaagct

4201 gccgcagatg gagaccttcg tgtacccgac cgggcacgcc ttcaaccgca gcatcgatcc

4261 gacccactac gacgccgata gcgccgagcg tgcgctggag cgcaccctgg gcttcttcgc

4321 agcgcacctg ggatgaacgg caccacgcca ggcatgcctg cgggcgattt ccaactcgat

4381 ccgcgcctgg cggcggacag cgtgttcgtc gccgacgggc cgctgtcgca ggtgcggttg

4441 atggacgatg cgcgcttccc gtggctggtg ctggtgccgc gggtggccga tgtcagtgag

4501 tggatcgatc tggatggcgg ccagcagcgt ttgttgctgg ccgaaatcaa ccagctctcg

4561 cagctgctgc gcgccgaacc tgcggtgagc aagctcaata tcggcgcgct gggcaatatc

4621 gtgcggcagc tgcatgtgca cctggttggc cgtcatcccg gcgatgcggc ctggccgggg

4681 cccgtctggg gcagcggcag tgcgcagcgc tttgcgtccg acacgttgca gcagcatgtc

4741 gcagcgtggg cgcagcggct acgataggcg cctgtcttcc aaccagtctc ccgcatgaaa

4801 tccaatgtcg tcgccgcgct gatcctgatc cttgtcggcc tgctgttcct ggccaacaac

4861 ctgggatgga ccaatctgag tattggtcgc ctgatcgcta cctggtggcc cgccgctctg

4921 gtcgcttgcg gagtcggcat gctattcggg cgcggtaatt aggtggggct gggattgggg

4981 agttgggagt tgggattggt tagagcgggc tgctggcgct actggtgttc cagcggctgg

5041 gcgggattgg cgcttgcgcg ttgatgtgcg gcgctgcgcc cgggtggcgc ggtcgccgac

5101 tgcgcgcgca gcgtttcgtt ttgccagccg atctcttatc tacgctgacc gcaggcatcg

5161 gtcgccacga agttgggcgg atttggcacc tcaccgatct ggaaccgcca cagcccgtcg

5221 ccggcggcag tctgcttctc ccgttgcgac attgtcctcc ttcatggggg agaaggtgcc

5281 ccgaaggggc ggatgagggt gccggcgaag ccttgtgcac tgtcgatgcc acttcaaaac

5341 gggatgctcg gtccccatca gcgctgctta tcgcgtggac gcggctagcc agcaccggcg

5401 gcacccacac ccccgcccgt cccgccccac gcggcgaccg acatgacgtt tgtcatctgc

5461 gattgttgac ttccggcacc tgatcggcgg gcgcatccgg cgcagcatgg cctcacaccc

5521 aaccctgaca tcgccatgaa ctaccgcctg atccccgccc tgttcctgat cgtcctgggc

5581 gcgctgttcc tgctggacaa cctgggcctg gcccatatgg atgtcggcaa cctgatcgca

5641 acctggtggc cggtatttct cattgccgcg ggcgtgcgcc aattgctgca ctaccgcgag

5701 aaggccgccg cgacctgatg aaggtcacag cagcgccctt gcctcaagag ctgctttacg

5761 tgcgcggcaa cgtcacgccg gtctgaccct gatacttgcc gccgcggtcc ttgtacgagg

5821 tttcgcagac ttcgtcggac tggaaaaaca gcatctgcgc cacgccttcg ttggcgtaga

5881 tgcgcgccgg cagcggcgtg gtgttgctga attccagcgt gacgtggcct tcccactccg

5941 gctccagcgg ggtcacgttg acgatgatgc cgcagcgcgc gtaggtgctc ttgcccaggc

6001 acaccaccag cgtgtcgcgc gggatgcgga agtactccac tgtccgtgcc agcgcaaagc

6061 tgttcggcgg aatgatgcac acatccgatt cgacatcgac aaagctgccc gggtcgaagt

6121 gcttcgggtc gacaatcgtc gagttgatgt tggtgaagat cttgaactcg cgcgagcagc

6181 gcacgtcgta cccgtagctg gaggtgccga aactgacgat gcgctgcccg gcagcgtcgt

6241 gcttgatctg cccgggctcg aacggcgcga tcatcgcgtg ctgttcggcc atgcgcttga

6301 tccagcggtc gctcttgatg ctcatgcggt gttccttgaa gacggaaggc ggcgagccga

6361 gcggcccacg cgaagacggc ggattctacc agcggccggc atccgcagcg ggccggcacg

6421 ctacagcagg gatgccgaaa tcgggatact ggcgcgcggg cgcttgccca gttcctcggc

6481 aagccgcgtg gccgccgcca gatagaccag ggccgccgac gattctggcg cggccaccac

6541 aatcggctga ccggcatcgc cctgctcgcg gatcgcgatc tccagcggca acgatccgag

6601 caacggcacg ccgtactgcg ctgccatgcg ctcgccgccg ccttcgccga acagatgctc

6661 gcgatggccg cagttgctgc aggtatgcac cgccatgttc tcgacgatgc caagcaccgg

6721 cacctcgacc ttttcgaaca tcttcaaagc cttgcgcgca tccagcgtcg caatgtcttg

6781 cggcgtggtg acaatcaccg caccggccac cggaatcttt tgcgacaggg tcagctggat

6841 gtcaccggtg cccggcggca ggtcgatcag cagatagtcc agatcgcccc acagcgtgtc

6901 gttgaacagc tgggtcagag cggaggtcgc catcggcccg cgccagatca tcggcgtgtc

6961 ctgatcgaca agcaggccga tcgacatcgc ctcgatcccg aacgcacgca gcggctcgat

7021 cgacttgttg tccggactct cggggcgacc gctcagaccc agcatcgccg gcacgctggg

7081 gccgtagata tcggcatcca acacacccac ccgcgcgccc agccgacaca acgccagcgc

7141 cacattcacc gccgtggtgg acttgcccac cccacccttg cccgaggcca ccgcaatcac

7201 attgcggatg cgcgcatgcg gcgccagcgc accttgcacc gcatgcgcag caactctgcg

7261 ttcaccggtc atgcgcacac cttttgcgca agtacaaatt cagccacaaa accacgttcc

7321 accattgtgt tcatcgcgtc ccccgacttg tcgtgtacca ccgcgcgcga ctcacacatc

7381 cctctgcgct gcgtcaaaag aagaggcaag tgtaccggct cggctacaga cagcgtgcct

7441 gttcgccagc atcagacgac gcgcgcgcca acgcgcgcta cttcgcaaca aatttcgcga

7501 aaatcaaccc gatattacag gattatgccg gcggctagca gataatatgg cctccgctaa

7561 cactgaggcg gttcactgtt ggctccggca gcgcgaatgc acagagcggc ttacctcacg

7621 gcccgatcac ccacgccaga tgcacgtcca ggtgttcggg cacgttcgcc tcgatgtgcc

7681 tgagcaacgc caggaactcc tgatgacggt gcgcgggttt gcactgcgtg agcacggtgc

7741 cgttggccgc gtccagcgcc gcaaacaacg tcgtggtgcc gtggcgcacg tagtcgtggg

7801 ggatgccctc gacataaccc agccccatcg gcagaacagg ctgggtgcgt tcaagcgtct

7861 ggcactggct cttctcgtcc acgcacagca ccagagcatg atccggcgga ttgaggtaca

7921 gcccgacgat attgcgcacc ttctcgatga agaacggatc gttggacagc gtgaagctct

7981 ttgatcgctg cggctgcagc cccaacagcg acaggtaccg gtgcacggtg ctcttcgaaa

8041 gcccggtctc atcggccagg ctacggcgcg accagtgcgt cttgccggcc ggcttcctgg

8101 tcagcgcggt attgatcaac gttgcgatct cttcctcgcc ggtgctgcgt gagcgtccgg

8161 gcttgagcgc gttgtgcagc ccggcgatac ccccccgcag cgaagcgctt gcgccacaac

8221 gagacggtgg gccgactgat ccggtagcgg cgcgccacca cactgttggt cgcgccgtcc

8281 gccagactca gaatcatctg cgcacggcgc gacaatgcgg ccggaaggct ttgcgatcgg

8341 gcgatcgact cacgctgctt ccggtcctgc gtggtgactg tcaactcggt tgcacgcctt

8401 cccatcaatg catctccaca gtgccaagga tactgcttgg accacgcaaa gaatggaagg

8461 ttacatgcgg gacatcacac tagcaccaga caatcaacga tgcacgcagc cgagcgtggc

8521 cagaaacctg ctgcgaaaag cgtaacgacc gccgctttac ccgtgctcac ttccatgtaa

8581 tcccttttta gagcggctaa caaaacgact gcgctcaccg cccgcgccca cctgacagct

8641 gctcgcaacg tgtttttagc cgctcttaga tgtgtccttc actggaagga ttccacggca

8701 gcgggatcga gactaccttg gggcggcgat ggtcagggca atttgggacg atgaagaagc

8761 gacgcctctg gagtgacgac tgcgtctgcc tctttcgcat ctcagccaca cgccgagaca

8821 acagcatcag acaggatctc caggccctcg cgcagcaatg catccggtgt ggtcaatggg

8881 gccatcacct tcactacctc gccgcgtgcg cccgccgttt cgatcaccag tccgttgccg

8941 aatgcgtgag cgcttgcgga ggcggcgcgt tcgcgcgtgc gcaggtcaag cccgacgaac

9001 atgccgcgtc ctttgatgcg cgcttccggc acgtgcgcgg tcatgcgtgt cagtgcctga

9061 gcaaggattt gaccgcgttt gtcgatctgc gtgcgaagcg catcgtctgc ccagaatttg

9121 cgtagtgcca cggttgcggt gagcatcgcg tggttgttgc cgcggaaggt gccggtgtgt

9181 tcgcccggac gccagctgtc gtgctccgga gctaccagca gcagggaaaa cggcaagccg

9241 aagccagaca aggatttgga caaggtgatc aggtcgggga ccagcgcctg gtcgtcaaaa

9301 ctgaaaaagt ttccgcagcg tccgcagccg gcctggatat catccacgat ggtcagcgcg

9361 ccatgacggc gagcggtagc gaatacccga tgcaaccagg atgtggacgc gacgttgagt

9421 ccgccttcgc cctgaaccag ttccagcacg attgcagcgg gcgggtcgat cccgctggaa

9481 ggatcgtcga gcatcgcctc gagcagggcg gcactgtcga gtccactgcc gaggtagtcg

9541 tcgtagggta gccgggtgac gccgccatgc agcaggtcca tgcgatggtg gcggttgccc

9601 gtcgcggcca gcgctcccat cgataagccg tgatacgcat tgctgaaggc gatgatgttg

9661 tggcgtcccg tgcgcttgcg tgccagcttg agcgcagcct cgattgcatt ggcaccggtc

9721 gggcccgtga attgcagccg gtggggtaag ccgcgcggac gcaggatgag cgcgacgaag

9781 ttgtcgatga agtcgtgctt ggtctgcgag tgtaggtcca gcgacatcgc caggccgcca

9841 ccgcgcagat gcgccaccag agcctcggtc atgtccggat cgttgtggcc gtaattgagt

9901 gcgccgcagc ctgcgagaaa gtcgatgtag cggcgtccct gtacatcgaa tagttcactg

9961 cccgcgccac ggacgaacag cgcgtcgaat gcgcggcaat agctgcgcac attggattca

10021 tggtgctgga acagttggcg cgggtcgatc atgtcggttc cgattcgtga cggcagcatg

10081 cgggcctggg actgccaggc gcgtccggaa ttttttacgt ggtgcctggc agaacgtccg

10141 ccggggacga ctattggcta cgttatctcc ccagcggcca tggacgcgtg gcgctgcgct

10201 gtgacgtact ccgtgccggg gtcggggccg agcggtgaat aaccggcatt ttttaagttg

10261 atgctcgccc agtgtcttga ctggctgtgc cgtgcaattg tctctagcgt cgcagcccgg

10321 aatacggaaa gcacatgcgc agatacgccg tatttccgtc atccgtgcgc gttagcatcg

10381 tccaatgcgg acactgacct gcctggatga tgttcaagcg ttggcgcgtc gccgggtgcc

10441 gcggatgttc tatgactatg cgaccaccgg cagttggtcg caatggacag tgcaggcgaa

10501 tcgccgtgat ttcgacgcgt gttcgttgcg tcagcgcatc ggctgcgatg tttcgcagcg

10561 cagtacagcg acgcacatgc tggggcacgc ggtaacgatg ccggttgcgc tggcgccgac

10621 cgggctggcc ggcctgattc atgccgatgg cgaaattctc ggtgcgcgtg cagccgaggc

10681 gtttggcgta ccgttcgtct tgtcgacgat gagtatttgc tcgttggagc aggtgcgtgc

10741 aggtgtgcgc aagccgtgct ggtttcagct ctatccgctg cgtgaccgag gcgtcgtcgc

10801 agcgttgatc gaacgcgcgc gcgttgccgg ttgcagcact ttgatggtga cgatggatgt

10861 gccgttcctg ggccagcgcc acgccgatct gcgcaatggg ctgagcgtgc cacctcgctt

10921 gcggcctgag gtgctgctgg attttttgac ccatccgcgc tgggcgctgg gaatggtgcg

10981 gacgcgccgc cgttgcttcg gcaatctcgt cgattacatg cccgctggag gcgatctgca

11041 gacgctggcg cagtggaccg cccaccagtt cgatgccagc ctgggctggg atgatctggc

11101 ctggatcaga agccgctggg gtggggcgct ggtagtgaag ggcgtcctcg atgcggaaga

11161 cgcgcgacag gcgttgcagt ccggcgctga tgccttggtg gtgagcaatc atggagggcg

11221 acaactggat ggcgccgtgt ccagccttcg cgccctgccg gcgatcgccg aactcgcggc

11281 cggccgtgct gaagtgcacc tggacagcgg cattcgctgc ggtcaggacg tgctcaaggc

11341 actggcgctt ggtgcatgtg gcacctatgt cggccgcgcg tggttgtacg ggctcggcgc

11401 gctgggtgag cggggcgtgg cacgggtgtt aacgctgatc cagcgcgaac tggaattgac

11461 gatggcattg tgcggacgaa cccggatcgc agagatcgac cgttcggtcg tggtggattg

11521 atcgcaggaa gctggccatt gccagctgac acaggctgcg taagcgccgc agcgtcatct

11581 ggtaaacgaa gctccgtagc gagtctgctg gtgccgtgcc tgccctggct gctgcgcgtt

11641 atcggatgac gcgacatgcg gaattctaga gcgacgggca ggctgccagt tggcgcaggc

11701 ggccggctgc atgcaatcgg tggcgcacga agcgcgcgaa ccgtgcgatg ccttcggcga

11761 tcttgtcggt tgccacattg ctgtaggcaa gccgcacctg ctggccgcag ctgctgtcga

11821 tggcgaagaa cgacagcggc atcaccagga cgccgcactc acgcgcgcaa gacgcggcct

11881 cttcggcggc gaacacgaat ggcagcgtga cgatcaggaa gaagccgccc gccggaacat

11941 tccatcgaat ctgcggtgtc agcgtaccga gttcctggct caggcattcc agcatgcggt

12001 cgcgattgcg ctggtacaag gcgcgcggcg cctggatcaa tggtgtcaag ctgcagccgt

12061 gttcgagcag cacgccgccg ccgatggctt gggtgacctg gctggtgttg agcgttgcga

12121 aacttttgcg ccgtgtcagc gcctggtgca gcgtgcgtgc cgctgccgcg ctgccgaaca

12181 gcgtcggtgg caacaccaaa aagccaaccc gcaggcctgg gcacagcgtc ttggagtagg

12241 tgcccagata gatgacgcac ccatgattgt ccagggcgcg catcgtgggg acacgttctg

12301 ctccgaaggc gaacagccca taaggattgt cctccaggat caaaacgccg tgtgcgccgc

12361 acactgccat gattgcctcg cgttgatcgc ggggtagcac cgttccagtg gggttgtcga

12421 aatccggcac cagatagagc aggcgcgcac gctgcccagc ggcatgtagc gcatccagcg

12481 tctcggtaag cgcttctgcg tcggtgcgac catcgcgatt gaatgcggcg atggggatgc

12541 cgtgaccgtc ggcgacgccg gtgatgccga tataggtcgg attgcggacc agcacaacgt

12601 cgttgggctg gcggcacagt tcggtgacgc acaactgcag cgcttcctgg cagcctgcgg

12661 tgatcagcat gcgatcgatg ctggccggta cgccttcgta cgcagccagt tgggccacga

12721 ccaggtcctc aatgatgccc tgtgtggttc cgtactgtgc gatcagatcg agcgcacctg

12781 cctggctggt gtggtgacgc tgcgccagat gcgtcgcaaa gcgcgggacg gcgcgcatcc

12841 aggcctgcag gtcgaagcag ctgtcggtcg gacgtcctga aacgaacgaa atggcgttgg

12901 ggtggcgcga agatgcctcg ttgagaaagg tcatcacgtc cagacggtcg ccaccagctg

12961 cttggtcggc ctgtgttctt gccatcggct ggctcgtgag gcgcgaggtg ttcgctcaat

13021 acgcgccact acgctctagt tgcgcaaaca attcgttgac cgatgcgtcc tggaaaccga

13081 cctcgccgtt gcgctcgatg aattcgaagc tcaggccggt cagctcgctg cgttcggaaa

13141 agatctgccg caacccctgg ccttcgatca cattggtgtc gaaggccagg ccgcgttcgc

13201 gcaggtgcct ggcggtggca tgggcatcgt cgacgcgcag ggcgatatgc gcgacgcccg

13261 cgccatgatg tgcgatcaat cgcgacacct gcgattgcgg ttcggtacct tggcaaagca

13321 cgaagatgag atcgccatgc tgcatctcgg ccgagcgcat gccggtggtc ttgccggtga

13381 tgtggcgtcg tcccgtcaac acaaaaccga gttgatcgcg aaacagcgcg attgacgctt

13441 ccaagtcgtc taccgccagc gcgatgtggt cgatcttgtg gtaacgcaga gcactatcgg

13501 gttgttggac gctatcggtc attgaatgct ccgtatgcca tcgtgtgatg cgcggcattg

13561 ctgacgaaaa ccatttcggc aagcgcatgt gcgcagattt taaaagagtc gcctgcattc

13621 gcgccgctgg agagaaattc agcgcaagct tcgctcagaa cgcaggttcg aaaataatct

13681 gtcaatacga attttctgtg acacgcatcg atacatttag cgagcgccag accgcgcctg

13741 gatgatgtgg gcgaaatcaa gagcggctaa caaaccgtag cgagcagctg tc

LOCUS AFHK01000103 14592 bp DNA linear BCT 09-MAY-2011

DEFINITION Xanthomonas oryzae X11-5A Xo_X11-5A_contig_103, whole genome

shotgun sequence.

ACCESSION AFHK01000103 [AFHK01000000](http://www.ncbi.nlm.nih.gov/sites/entrez?db=Nucleotide&cmd=Search&term=AFHK01000000)

VERSION AFHK01000103.1 GI:332357396

DBLINK Project: [66097](http://www.ncbi.nlm.nih.gov/bioproject/66097)

KEYWORDS WGS.

SOURCE Xanthomonas oryzae X11-5A

ORGANISM [Xanthomonas oryzae X11-5A](http://www.ncbi.nlm.nih.gov/Taxonomy/Browser/wwwtax.cgi?id=1009853)

Bacteria; Proteobacteria; Gammaproteobacteria; Xanthomonadales;

Xanthomonadaceae; Xanthomonas.

REFERENCE 1 (bases 1 to 14592)

AUTHORS Triplett,L.R., Hamilton,J.P., Buell,C.R., Tisserat,N.A.,

Verdier,V., Zink,F. and Leach,J.E.

TITLE Genomic Analysis of Xanthomonas oryzae from US Rice Reveals

Substantial Divergence from Known X. oryzae Pathovars

JOURNAL Appl. Environ. Microbiol. (2011) . 77(12):3930-7.

PUBMED [21515727](http://www.ncbi.nlm.nih.gov/pubmed/21515727)

COMMENT Base Quality-A custom perl script was used to clean and remove

reads with low complexity and low quality regions. Low quality

regions were defined as an average quality score of < 20 over a

10bp window along the read or > 2 'N' bases in the read. The Velvet parameters used in the final assembly for X11-5A were a hash length of 31, expected coverage of 19.58x, and a k-mer coverage cutoff of 9.79.

Contact Dr. Jan Leach for source DNA or cultures (Department of

BSPM, Colorado State University, Fort Collins, CO).

##Genome-Assembly-Data-START##

Assembly Method :: Velvet v. 0.7.53

Genome Coverage :: 70x

Sequencing Technology :: Illumina GAIIX

##Genome-Assembly-Data-END##

**Annotation was performed by Royer et al. (current manuscript)**

FEATURES Location/Qualifiers

source 1..14592

/organism="Xanthomonas oryzae X11-5A"

/mol_type="genomic DNA"

/strain="X11-5A"

/host="Oryza sativa cv. Lemont"

/db_xref="taxon:[1009853](http://www.ncbi.nlm.nih.gov/Taxonomy/Browser/wwwtax.cgi?id=1009853)"

/country="USA: Texas"

gene complement (<3..11033)

/gene="NRPS"

CDS complement (<3..11033)

/gene="NRPS"

/note="incomplete NRPS; no stop codon"

/codon_start=1

/translation="MHVRIRRRPLPLLATRIFMDRPLKTLPASFAQQRLWFLARLDPRGSTAYHLAGGLRLQGRLDEGALRAALDRIVERHEVLRTCLVEVQGQVRQHILPAAGFCLQQTDLRHHAQPAAAVDAYARAQSCRAFDLAQGPLIRGQLLQLDAQHYVLLMTLHHLVSDGWSMGLLLNELGTLYAAFLSGHPDPLPPLPVQYADYAIWQHKRLGSEALQRHAAYWQAQLGGAPTLLPLPLDRPRPVQQDYAGASVAFVLPTPLSQALLELARSHRCSLFSVLLAAWALLLSRLSGEQDLVIGTAVAGRTRSEIEPLIGLFVNTVALRFQVPPHGRLSDWLQQVRDVVLSAQDHQDLPFERVVELLQPPRALGHTPIYQVMFALDTMQQGQSLALPGLEVAPLCIGQDSIEFDLSLSMQQHGEALSGRLGYATALFDSESMQRHVAQFLQLLQAIGTDANPHLARLSWLPQEQRERLLHDFNATAAPLTPAVPLCQALLAQVQRTPQATAVIDGELGLSYAQVWSRAACLADHLQQQGLQPGQPVALLLPRSASLIIAQLAVLRCGACYVPMDPEQPVERLGQLLRACDARLAVIHAQMPPLDVAGLQVVDPGRWALDGHARLHHVLSHPHALAYVMYTSGSTGMPKGVAVSHAAVLNLVLQDGPARLRADDRVAFASNPAFDSATLEVWGSLLNGATVVVVPAPVMRDPQALGALLMRERLSVLILVAGVLRAYAPLIAAQLGALRLLLTGGDVADPHALATVFAAAGPATVLQTYGPTESTQFVTALALQHAPDATRRVPIGRPLANIRLYVLDRHGQPMPIGVAGELHIAGAQLAQGYLHRPALTAERFVPDPFAEIPGERMYKTGDLACWLADGTLDFLGRNDAQVKIRGFRIEPGEIEAALRACAGVQEAVVVARNDTGDKRLVAYLVSDASMTEPAMLRTRLATHLPDHMLPAAYVQLDSLPLTNNGKLDRAALPAPDDQALDLHVYAAPQGEFEQVLATLWGDLLGVEQVGRHDDFFALGGHSLLAVQLISRLRERLGVELALADIFAHPTLAALASVLAHAAPQTLPPIVPAARTAPLPLSFAQQRLWFLAQLDRQADLAYLMANGLRLRGHLDRQALMHALDRLVARHETLRTRIALQHGEPVQIIDPDGVGFSCTEHDLSTFSDPQDQVRVHIEQEMHTRFDMAQQRLARGRLLRLGESDHVLLVTLHHLICDGWSMGRLVGELSTLYSAFAQGQPDPLPPLSLQYADVAVWQRRWISSEVLQRQREFWGEQLQDAPPLLDLPTDHPRPPLQDYRGDTVDIALDAKLTAALRALSQRHGTTLFMTVLAGWAVLLSRLSGQDQVVIGTPIAGRDRSELEPLIGLFVNSLALQIDLRHSPDTASLLRQVRATTLAAQAHRQLPFDQIVEALNPVRSTAHSPVFQVMFAWQNAPEGRLQLPGLTLRPVALPTQSVQFDLEIAMAEHGDALLGSMGYATALFARSSVEQHVALLIATLQAMAAQTHTPVTRLPLPRPAELAQQPCCHDNIRTVDAVPVHRLVEQQAAQRPNAIAVQDAMQTLDYAGLNLRANRLAHHLIALGVAADVCVALCMQRGVDAIVAMLAVLKAGGAYLPLDAAYPPERLHYMLASSAPRVLITDALSRACLPDAIDAVVLRIDADADAWSAAPSGNPHVQQLHGDHLAYVIYTSGSSGQPKGVMVAHQGLATRLHALIDAYGLGAQDRVLQFATLSFDASVEEVFGALCSGATLLLRDDTWLDAGRFWQQCANAGITVVALPTRFWAQLCAHSLQIPDGVRQVIIGGEALRPAMRQTWVQGTRTALLDTYGPTEAIVVATTQAVAADTPTGIGRPLAGTQAHVLDRRGQPLPIGARGELHLAGVALARGYLGRPDLTAERFIPDPFAEQPGQRMYRTGDLACWRVDGSLSFLGRNDRQLKLRGFRIEPSEIEAALLDGDGVREAVVISRNDTGQDRLVAYLVADQACIDADSLRSRLATRLPNYMLPSAYVQLDALPLTINGKQDLRALSVPPPATTEAIDDVQQAQGAIEQALAALWRQLLGVANIGRDDDFFALGGHSLLAVQLISRVRAGLGVELQIGEVFNHPQLHALARRVASAAASTRPAIVPADRSAPLPLSFAQQRLWFLAQLDTRADLAYLMPNGLRLRGRLDRHALRQALDRIVARHAILRTRIGLHQDDPVHIIAADTIGFPLREHDLSASPDQDAQVQVLARSEAETPFDLAHDILIRGQLLCLGEDAHVLLITLHHLVCDGWSMGVLVQELNTLYAAFAQHQPDPLPALSLQYTDVAVWQRRWIDGPLLQRQLSFWREHLHGAPTLLELPTDRPRPALQDYSGDSSDIALATTLSAALRAVSQRHGTTVFMTLLAAWAVLLARLSGQEQVVIGTPIANRTRTELEPLIGLFVNTQALCIDLRADPSVAELLAQIRATALAAQEHQDIPFEQIIEALNPARNLAHHPVFQAMFTWQNASSSESELALPGLQLQSLSQSLSLSALKFDLDLTLEERDGCIVGSLGYATALFDATTIQRWSRCFEQLLHALTCDDEARVSQLPWLDAPQRRDVLAGFGTGAIAAVPDQPLHQLFEAQARRTPDTIAVVSEHGCLRYAALDTQANRLAQRLRSVGVRAGERVAIALPRSAESIVAQLAVLKCGAAYVPLDVDHPNDRLLALIADAQAVVLIHDADSALAPAQLACMTIEEFDNLDDSSGAVAQGWSATLHADVATQPAIAVPTTATAYVMYTSGSTGMPKGVAVSHGAVAHLVLQDGPARLRSDDRVAFASNPAFDSATLEIWGSLLNGASVVVVPAPVMRDPHALGALLVRERLSVLILVAGVLRTYAPAIAEQLGALRLLITGGDIADPHALATVLDAGGQATILQTYGPTESTQFVTMMALQHAPDAARRVPIGRPLANTRLYVLDRHGQPTPIGVTGQLHIAGAQLAQGYLHRSALTAERFVPDPFAETPGERMYKTGDLACWLADGTLDFLGRNDAQVKIRGFRIEPGEIEAALCACAGVQEAVVVARDDTGEKRLVAYVVADRNAAPAESATLRTQLAAHLPDHMLPAAYVQLDALPLTPNGKLDRAGLPAPDNQALELHAYVAPQGELEQMLATLWSGLLGAAHVGRNDDFFALGGHSLLAVKLIERLRRLGWQIDVRALFARPTLAGLAENLQAASTCVVPPNRIGPDCTRITADLLPLVALTQVEIDAVVATVDGGTANVQDIYPLAPLQEGLLFHHLGDPLADPYLHSSVLGFPSKERRDGFLDALDHIVARHDILRTGFVWQGLSAPVQVVWKTAVVPRHLQHFDGPDPAAQLRAWLHAPGAALGLQHAPLIHAHFANDTTTGRWLLGLQHHHLVMDHTTLELLIEEVRAHLAGRQQQLPTPLPFRDFVAHTHAGLSEQQHRTFFTEMLADIDTPTAAFGVRVPVAEPAGLQELHQPLPQALAQSLRTQARQHGVSAASVFHLAYALLLARTSGSTEAVFATLLFGRMHASAGVDRAFGMFLNTLPIRLGAAHGSVIDAVRHTQRCLAQLLHHEHASLALAQRCSTLDPSTPLLNALLNYRYAGGSTVLSATQEDALQDVQQIGGQERTHYPLVVSVNDHTEEGGFALDVQCVQDIGAERIAVMLLQTVQLLVQALEYAPQTALHALDLLPENERAQLRHFNDT"

gene complement (11214..14591>)

/gene="NRPS"

CDS complement (11214..14591>)

/gene="NRPS"

/note="incomplete NRPS; no start codon"

/translation="RVGRHDSFFALGGHSLLGVRLISRIRSVLGIELPLAALFAQPRLAEMTQALDSAAASTLPAIVPADRSAPLPLSFAQQRLWFLNQLDPRTGATYMMRGGVHLSGNLHVPALTAALNRIVARHETLRTHFANVDDLPIQIIAAPCDIALQLIDLSGEPSPDSAARVHARNEASTGFDLANGPLFRGRLLRLAEHEHVLLLSMHHIVSDGWSIGVLIEELGALYTAFVSDAPDPLPPLPIQYADYAAWQHRWIDNQLQQRQLEFWRAQLHDAPALLELPTDRPRPPLQDTAGDDVQLVLDETLSLRLQALAVQHGISIFGLLLAGWAALLARYSGQTDLVIGTPSAGRNRSELEPLIGFFVNTLPLRIDLSAQPTFLELLNQVQATLLAAQSHQDLPFERIIEAVRPVRSLSHSPLCQTMFSSDTTPARALELPDLQLSAYPNDHHVAQFDLSLDMQIAPTRIAGVLRYATALFERSTMQAYLDNYARLLAALADAPTLAVDRPALLDADGWRALQHWNDTARAVAPLPTIHTAFQAQARSTPDAIAVIDGVRTLRYRELDAYSNRIALQLIEAGVRTGDCVVTLLPRSAELVAAQLGILKAGAAYVPLDPRQPAARHAQLAADCQARAIVHAPGDAPPWTTAPCLDIALTRKPTDVFVAPPLPAAAPAYVMYTSGSSGNPKGVLVPHQAVLNLVRDQNYARWQADDRFAFASNPAFDSSTLEVWAPLLSGGSVVVVPEDVLLDPCVLADFARQHAISVLILVAGVLRVYASELARTLPTLRYLITGGDSADPQAIATLLGGQRAHNLLQTYGPTETTQFVTAVAVSEVPDAGQRIPIGTPIVNLRVYVLDAHRQALPVGMQGELHIAGLGLALGYLRQPGLTAEHFVPDPFSAVPGARMYRTGDLGRWRADGQLECLGRRDAQSKIRGFRLEPGEIEAALRTHPQVDQALVRVCEDTAGQRRLVAYVIGTQIDDSAAHDPNVLRSHLADMLPDYMLPDAYVVMQAWPLTANGKLDVRALPAPDDAQRGIAEIDPPQGETECALAEIWCALLGISTVNRHDNFFDIGGHSLLAVQLATRIQARLGRRLPLSRLFAEPTLARMAVALADSRVASSAPIATLVNRSNYYE"

ORIGIN

1 gtgtcattga agtgtcgcag ctgggcgcgc tcgttttctg gcagcagatc caatgcatgc

61 agagccgtct gcggtgcgta ctccagcgcc tggaccagca actgcacggt ctgcagcagc

121 atcaccgcga tccgctcggc gccgatgtcc tgcacgcatt gcacatcaag tgcgaagccg

181 ccttcttcgg tgtggtcgtt gaccgacacc accagcggat agtgggtgcg ctcctgtccg

241 ccgatctgtt gcacgtcctg cagcgcatcc tcctgcgtgg cgctcaggac ggtgctgccg

301 ccggcatagc ggtagttgag caaggcgtta agcagcgggg tggacggatc cagcgtgctg

361 cagcgctgcg ccaacgccag cgatgcatgt tcgtggtgca gcagttgcgc aagacagcgt

421 tgggtatggc gcacggcatc gatcacgctg ccgtgcgcag cgcccaggcg gatcggcaag

481 gtgttgagga acatcccgaa tgcgcggtct acgccagcgc tggcatgcat gcgtccaaag

541 agcagggtgg caaacactgc ttcggtactg ccgctggtgc gggccagcag cagcgcgtag

601 gcgagatgaa acacgctggc agcgctgacg ccatgctgtc gcgcctgcgt gcgcaaggac

661 tgtgcaagcg cttggggcag tggctgatgc aattcctgca gacctgcagg ttcggcaacc

721 ggtacgcgga cgccgaatgc ggcagtgggc gtgtcgatgt cggcaagcat ttcggtgaaa

781 aacgtcctgt gttgctgttc ggacagcccg gcgtgggtat gggcaacgaa gtcgcggaac

841 ggcaagggcg tcggcagctg ctgttgccgg ccggctaagt gcgcacgcac ttcttcgatc

901 aacagttcca gcgtggtgtg gtccatcacc aggtgatggt gttgcaggcc gagcagccag

961 cggccggtgg tggtgtcatt ggcgaaatgc gcatggataa gcggtgcgtg ttgcaggccg

1021 agcgcggccc cgggagcgtg gagccacgcc cgcaattgcg cggccggatc ggggccgtcg

1081 aagtgctgca ggtgacgcgg gacaacggct gttttccaga ccacctgcac gggtgcagag

1141 agcccttgcc agacaaatcc ggtgcgcaag atgtcgtgtc ttgcaacgat gtgatccagt

1201 gcatcgagaa agccatcgcg ccgctctttc gacggaaagc cgagcacgga cgaatgcagg

1261 tacggatcgg cgagtgggtc gcccagatga tggaacagca ggccttcctg caggggcgcc

1321 agcggataga tgtcctgcac attagccgtg ccgccgtcga cggtggccac gacggcgtcg

1381 atctccactt gcgtcagcgc gaccagcggc aataggtccg cagtgatgcg cgtgcaatcg

1441 ggaccgatgc ggttgggtgg taccacacag gtggatgcgg cctgtaggtt ttcggccaga

1501 cccgcgagtg tggggcgagc gaacaaggcc cgcacgtcga tctgccagcc gagccggcgc

1561 aggcgttcga tcagtttgac ggccagtaac gaatggccgc cgagcgcgaa gaagtcgtcg

1621 ttgcgtccga cgtgtgcggc gccgagcagc ccgctccaca acgtggccag catctgctcc

1681 aattcgcctt gcggagcgac ataggcatgc agttccagcg cctgattgtc tggtgcgggc

1741 agcccggcgc gatcgagttt accgttgggg gtgagcggca atgcatcgag ctgcacgtaa

1801 gcggctggca gcatgtggtc gggaaggtgt gcggccagtt gagtgcgcag cgtcgccgac

1861 tccgcgggtg cggcgttccg atctgcgacc acgtacgcga ccaagcgttt ttcgccggtg

1921 tcgtcgcgtg cgacaacgac agcttcctgc acgccagcgc aggcgcacaa ggcggcttcg

1981 atctcgcccg gctcgatgcg aaagccccgg atcttgacct gcgcatcgtt gcggccaaga

2041 aaatccagcg tgccgtcggc cagccagcaa gccaggtcgc cggtcttgta catgcgctcg

2101 ccgggagtct cggcgaatgg atcgggcacg aagcgttcgg cggtcagggc agagcgatgc

2161 agatagccct gcgcgagctg tgcaccggcg atatgcagct ggccggtcac accgatcggc

2221 gttggttgac catggcgatc gagcacataa agccgggtat tggccagcgg tcggccgatc

2281 ggcacgcggc gggctgcatc gggcgcgtgc tgcaacgcca tcatggtcac gaactgcgtg

2341 ctctcggtcg gaccgtaggt ctgcaggatg gtggcttggc caccggcatc caacaccgtc

2401 gcgagcgcat gcggatcagc gatatcgccg ccggtaatca gcagacgtag ggcacccaac

2461 tgttctgcga tcgctggcgc ataggtgcgc aacacaccgg caaccaggat gagtaccgac

2521 agccgttcgc gcacaagcag cgcgcccagc gcgtgtggat cgcgcatgac cggcgcagga

2581 accacaacca cgctagcgcc attgagcaga ctgccccaga tctccagcgt ggccgagtcg

2641 aaggcggggt tggaggcgaa cgcgacgcgg tcgtcggatc gcagtcgtgc cggaccgtct

2701 tgcaggacga ggtgagccac cgctccatgc gacaccgcca cacccttcgg catgccggtg

2761 gaaccggagg tatacatgac ataggccgtc gctgttgtgg gtacggcgat cgccggctgc

2821 gttgcgacgt cggcgtgcaa ggttgcggac catccttgag caacggcacc gctgctgtcg

2881 tcgagattgt cgaactcctc aatcgtcatg caggcgagct gcgcaggcgc cagcgcactg

2941 tcagcgtcgt ggatcaatac aaccgcttgc gcgtcggcga tcagcgcaag caggcggtcg

3001 ttgggatgat ccacgtccag tggcacatac gccgccccgc acttgagcac ggccagttgc

3061 gcgacgatcg attctgccga acgcggcaac gcgatggcca cgcgttcgcc ggcacgcacg

3121 cccacgctac gcaaacgctg cgcgagtcga ttggcctgcg tgtcgagtgc ggcataacgc

3181 aggcagccgt gctccgacac cactgcgatg gtatccggcg tgcggcgtgc ctgcgcttcg

3241 aacagctggt gcagcggctg gtcgggcacc gcggcaattg cgccagtgcc gaacccggcc

3301 agcacgtccc gacgctgcgg cgcatccaac cacggcaact gcgaaacgcg agcctcgtcg

3361 tcgcaggtca atgcatgcaa caactgctcg aagcagcgcg accagcgttg gatcgtggtc

3421 gcatcgaaca gcgccgtggc gtagcccagg ctgccgacga tgcagccatc gcgttcttcc

3481 agggtcaggt ccagatcgaa cttgagcgcg gacagggaca gggactgcga caacgactgc

3541 agttgcagtc cgggtagcgc gagctcgctc tcactgcttg aggcgttctg ccaggtgaac

3601 atggcctgga atacagggtg atgcgcgagg ttgcgtgccg ggttgagcgc ttcgatgatc

3661 tgctcgaacg ggatgtcctg gtgctcctgc gctgccagcg cagtggcacg aatctgagcc

3721 agcaattcgg cgacggaagg gtccgcgcgc aggtcgatgc acagggcctg cgtgttgacg

3781 aacaggccga tcaacggctc cagttcggtc cgggtgcgat tggcgatggg cgtgccgatc

3841 accacctgct cttgtcccga taggcgtgcc agcagcacag cccatgcagc gagcagggtc

3901 atgaacaccg tagtgccgtg acgttggctg acggcgcgca aggcggcgct cagcgttgta

3961 gcgagtgcga tgtcactgct gtcgccgctg tagtcctgca gcgcagggcg cggccgatcg

4021 gtaggcagtt ccaacagggt cggtgcgccg tgcagatgct cgcgccagaa actcagctgg

4081 cgttgcagta gcgggccatc gatccagcgg cgttgccaca ccgcgacatc ggtgtattgc

4141 agcgacagcg caggtaatgg atcgggctgg tgctgggcga aggcggcata gagagtgttc

4201 agttcctgca ccagcacacc catggaccag ccatcgcaga ccagatgatg cagggtgatc

4261 agcaggacat gcgcgtcctc gcctaaacac agcaactgcc cgcgtatcag gatgtcgtgg

4321 gccaggtcga agggggtctc ggcttcactg cgggcaagca cttggacctg tgcgtcctga

4381 tcggggcttg cactgagatc gtgctcgcgc aatggaaagc cgatggtgtc cgcggcgatg

4441 atgtgtaccg gatcgtcctg atgcaagccg atacgggtgc gcaagatcgc gtggcgggcg

4501 acgatacggt ccaaggcctg gcgcagcgca tggcgatcga gccggccgcg cagacgcagc

4561 ccattgggca tcaggtaagc aagatctgca cgggtgtcga gttgcgccag gaaccacagc

4621 cgctgctggg cgaaggacag cggcaatggc gcgctgcggt cggccggcac aatggccggt

4681 cgcgtgctgg cggcagcgct ggcaacgcgg cgggccaggg cgtgcaactg cggatggttg

4741 aatacctcgc cgatctgcag ttccacgccc agaccggcgc gcacacgcga gatcagctgc

4801 acggccagca acgagtgccc acccagagcg aagaagtcgt cgtcgcgtcc gatgttcgcc

4861 acgccgagca attggcgcca cagcgcggcc agtgcctgct cgatggcgcc ttgcgcctgt

4921 tgcacgtcgt ctatcgcctc ggtggtggca ggtggcggca ccgacaaggc acgcaggtcc

4981 tgtttcccat tgatggttaa cggcaaggca tccaattgca cataggcaga cggcagcatg

5041 tagttcggca gtcgtgtagc cagtcttgaa cgcagcgagt cggcgtcgat gcacgcctgg

5101 tccgcaacca gataggcgac caggcggtcc tggccggtgt cgttgcggga aatcaccact

5161 gcctcgcgga cgccgtcgcc atccagcaac gccgcttcga tctcgctcgg ttcgatgcgg

5221 aagccgcgca gtttcagttg gcggtcgttg cggccgagga aggacaagct gccatcgacg

5281 cgccagcacg ccagatcgcc agtgcggtac atgcgctggc cgggctgttc tgcgaatgga

5341 tcggggatga agcgttcggc ggttagatcg ggacggccca ggtaaccgcg tgccaacgcc

5401 acgccggcca gatgcagttc gccgcgcgcg ccgatcggca acggctggcc acggcgatcc

5461 aacacatgcg cctgggtacc ggcgagcgga cggccaatgc cggtcggggt gtcggcggcc

5521 actgcctgag tggtcgccac cacgatggct tcggtggggc cgtaggtgtc cagcagcgcg

5581 gtgcgcgtgc cctggaccca ggtctggcgc atggccggac gcagcgcttc gccgccgatg

5641 atcacctggc gcacgccgtc ggggatctgc agcgagtgcg cgcacagctg cgcccaaaaa

5701 cgtgtcggca gagcgacgac cgtgatgccg gcattggcgc actgctgcca gaagcgtccg

5761 gcgtccaacc atgtgtcgtc gcgcagcagc agggtggcac cgctgcacag cgcgccgaag

5821 acttcttcga ccgaggcgtc gaaagatagc gtggcgaatt gcagcacgcg gtcctgtgca

5881 cccagcccat acgcatcgat caacgcatgc aggcgggtgg ccagaccctg gtgagcgacc

5941 atcacgcctt tgggctggcc gctggagccg gaggtgtaga tcacgtaggc caggtgatcg

6001 ccgtgcagct gctgcacatg cggattgccc gatggagccg cagaccatgc atccgcgtct

6061 gcatcgatgc gtagcaccac tgcgtcgatg gcgtccggca ggcatgcccg agacaacgcg

6121 tcggtgatca gcacccgcgg tgcgctgctg gcgagcatgt agtgcaggcg ctcgggcgga

6181 taggccgcat ccagcggcag gtacgcgccg cctgccttga gcaccgccag catcgcgacg

6241 atggcgtcga cgccgcgttg catgcacaag gccacgcaca catccgccgc cacgccgagc

6301 gcgatcagat ggtgggccag gcggttggcg cgcaagttca gacctgcata gtcgagcgtc

6361 tgcatcgcat cctgcacggc gatcgcgttg ggccgctgcg cagcctgctg ttccaccagg

6421 cgatgcaccg gcaccgcgtc gacagtgcgg atgttgtcgt ggcagcacgg ctgctgtgcg

6481 agctcggctg gtcttggcaa gggcagacgc gtcactggag tatgcgtctg tgcggccatc

6541 gcctgcagcg tggcaatcaa cagcgcgaca tgctgttcga cactgctgcg cgcgaacagc

6601 gcagtggcat aacccatgct tccgagcagc gcatcgccat gctcggccat ggcaatttca

6661 agatcgaact gcacgctctg cgtcggcaac gcaaccggcc gcagcgtcaa accggggagc

6721 tgcaaacggc cttctggtgc gttttgccag gcgaacatga cctgaaacac ggggctatgc

6781 gcggtgctgc gcacagggtt gagcgcctcg acaatctggt cgaagggcag ctgtcgatgc

6841 gcttgcgccg ccagcgtggt cgcgcgcacc tggcgcagca aactggcggt atcgggcgag

6901 tgccgcagat cgatctgcaa ggccagcgaa ttgacgaaca gaccgatcaa cggctccagt

6961 tcactgcgat cgcgcccggc gatcggagtg ccgatcacca cctgatcctg accggacaag

7021 cgcgacaaca gcaccgccca gcccgccagc acggtcatga agagtgtggt gccgtggcgc

7081 tggctcaacg cgcgcagcgc ggcagtgagt ttggcgtcca acgcgatatc gaccgtgtcg

7141 ccgcgatagt cctgcagcgg agggcgggga tggtcggttg gcaggtccag cagcggcgga

7201 gcatcctgca attgctcccc ccagaactcg cgctggcgtt gcagtacctc gctactgatc

7261 cagcggcgtt gccacacggc gacatcggcg tactgcaacg atagcggcgg cagtggatcg

7321 ggctggccct gggcgaaggc gctgtacagg gtgctcagct cgccgaccag cctccccatg

7381 gaccagccgt cgcagatcag atgatgcaag gtgacgagca atacgtggtc gctctcaccc

7441 aggcgcagca gtcggccacg tgccaggcgc tgctgcgcca tgtcgaagcg cgtgtgcatt

7501 tcctgttcga tatggacgcg tacctggtcc tgtggatcgg aaaaggtgct gaggtcgtgt

7561 tccgtgcagg agaagccgac gccatcgggg tcgatgatct gcaccggctc gccgtgctgc

7621 agggcgatgc gggtgcgcaa ggtttcatgc cgagcgacaa gccggtccaa tgcgtgcatc

7681 aaggcctgcc gatcaagatg gccgcgcaga cgcagaccgt ttgccatcaa ataggcgaga

7741 tccgcctgcc gatcgagttg cgcaaggaac cacagtcgtt gctgtgcgaa ggacaacggg

7801 agcggtgcag tgcgtgctgc cgggacgatg gggggcagcg tctgtggcgc ggcatgagcg

7861 agcacgctcg ccagtgccgc caacgtcgga tgggcgaaga tatcggccag ggcgagttcc

7921 acacccagcc gctcgcgcaa gcgcgagatc aattggaccg ccagcagcga gtgcccgccg

7981 agggcgaaga agtcgtcgtg gcgtccgact tgctcgacgc cgagcaggtc gccccacagc

8041 gtggccaata cctgttcgaa ctcgccttgc ggggcggcat agacgtgcag atccagcgct

8101 tgatcgtcag gtgccgggag cgctgcgcgg tcaagttttc cgttgttggt gagcggcagt

8161 gaatcgagct gcacataagc ggcgggcagc atgtggtcgg gcagatgggt ggccagtcgg

8221 gtacgcagca ttgccggttc ggtcatcgag gcatcgctga cgagatacgc caccaagcgt

8281 ttgtcgccgg tgtcgttgcg cgcgacaacc acggcttctt gcacgccagc gcaagcacgc

8341 aaggcggctt cgatctcgcc gggctcgatg cgaaagccgc ggatcttgac ctgtgcgtcg

8401 ttgcggccga gaaaatccag cgtgccgtcg gccagccagc aagccaggtc gccggtcttg

8461 tacatgcgct cgccgggaat ctcggcgaac ggatcgggca cgaaacgttc ggcggtcaag

8521 gcaggacgat gtagataacc ctgcgcgagt tgcgcacctg cgatatgcag ttcgccagcc

8581 acgccgatcg gcatgggttg accatggcga tcgagcacat agagccggat attggccagc

8641 ggccggccga tcggcacgcg gcgggttgca tccggcgcgt gctgcaacgc cagcgccgtc

8701 acgaattgcg tgctctctgt gggaccgtag gtctgcagaa cggtggccgg gccagcggcg

8761 gcgaacaccg ttgccagtgc atgcgggtcg gcgacatcgc caccggtgag cagcagacgc

8821 agggcgccca actgtgctgc gatgagcggt gcataggcgc gcaatacgcc ggcgaccaga

8881 atcaacaccg acagccgttc gcgcatcagc agcgcgccca gcgcctgcgg gtcgcgcatg

8941 accggcgcgg gaacgacgac aacggtggcg ccattgagca aactgcccca gacctccagc

9001 gtggccgagt cgaaggcggg gttggaggcg aaagcaacac ggtcgtcggc ccgcagccgt

9061 gccggaccat cctgcaggac cagattgagc accgcagcat gcgacaccgc cacgcccttg

9121 ggcatgccag tggagccaga ggtgtacatc acgtaggcga gtgcgtgcgg gtggctgagt

9181 acgtggtgca acctcgcgtg cccatccagc gcccaccggc ccggatccac gacctgcagc

9241 cccgcaacgt cgagcggcgg catttgtgca tggatcacgg ccagccgggc atcacaggcg

9301 cgcagtaact ggccgagacg ttcgaccggc tgttccggat ccatcggcac gtagcaggcg

9361 ccgcagcgga gaacggccag ctgcgcaatg atcaggctgg ccgagcgcgg cagcagcagc

9421 gccaccggct gaccgggttg cagtccctgc tgttgcagat ggtcggccag gcacgcggcg

9481 cggctccaca cctgcgcata gctgagacca agctccccgt cgatcactgc agtcgcctgg

9541 ggggtgcgct gcacctgcgc gagcagggcc tggcacaggg gcacggccgg cgtcagcggc

9601 gcggcggtgg cgttgaagtc gtgcagcagc cgctcgcgct gctcctgcgg cagccacgac

9661 aaccgggcca aatggggatt ggcgtccgtg ccgatcgcct gcaacagctg caggaactgc

9721 gcaacgtggc gctgcatgct ctcgctgtcg aacagcgcgg tggcgtaacc gaggcgcccg

9781 ctaagcgcct cgccatgctg ctgcatggac aagctgagat cgaattcgat gctgtcctgg

9841 ccgatgcaca gcggtgcgac ctccaaccca ggcagtgcca acgattggcc ttgctgcatc

9901 gtgtccagcg cgaacatcac ctggtaaatc ggagtgtggc cgagtgcgcg tggcggttgc

9961 agcagttcga cgacgcgctc gaacggcaga tcctggtgat cctgcgcgct cagcaccaca

10021 tcgcgcacct gctgcagcca atcgctcagc ctgccgtgtg gcggcacctg gaagcgcagc

10081 gccacggtgt tgacgaacag gccgatcaag ggctcgatct cgctgcgcgt gcggccggcg

10141 acggcggtgc cgatgaccag atcctgctcg ccgctgagcc gcgacagcag cagcgcccat

10201 gccgccagca gcacgctgaa cagcgagcag cgatgcgagc gcgccaattc cagtagcgcc

10261 tggctcaacg gcgtcggcag cacgaatgcg acgctggcgc cggcatagtc ttgctgcacc

10321 gggcgcgggc gatccaatgg cagtggtagc agcgtcggtg cgccgccaag ctgggcttgc

10381 caatacgcag cgtggcgttg cagcgcttcg ctgccgagtc gcttgtgttg ccagatcgca

10441 tagtcggcgt attgcaccgg cagcggcggc aacggatcgg gatgtccgga cagaaacgcg

10501 gcgtacaggg tgccgagttc attgagtagc agccccatcg accagccgtc ggagaccagg

10561 tgatggagcg tcatcagcaa cacgtagtgc tgcgcatcca gttgcagcaa ctgcccacgg

10621 atcagcggcc cttgcgccag atcgaacgca cggcaggact gcgcgcgcgc gtaggcgtcg

10681 accgcggcgg cgggctgtgc gtgatgacgc agatcggtct gctgcaggca gaatccggcg

10741 gcggggagga tgtgttgcct gacctggcct tgcacctcga ccagacaggt gcgcaacact

10801 tcgtggcgct ccacgattcg gtccaatgcg gcgcgcaggg cgccttcgtc gagcctgcct

10861 tgcaagcgca gtccaccggc caggtgataa gcggtgctgc cacgtgggtc gagccgcgcc

10921 agaaaccaca ggcgctgttg tgcgaacgaa gccggcaacg tcttgagagg tcgatccatg

10981 aaaattcggg tcgccagcaa tgggaggggg cgccgcctga tacgcacgtg cacacagagc

11041 tgtgcggcgt atcgaaggcg ctccgtgaag cgcctttttc ccttttactg aaccctcacg

11101 cttgcctcaa cgtgacaccc acttgcctga tgcaggacgc atgcatgcag gcaggtcggt

11161 aggccgaatc gaggccatcg cggcgaccgc gcggatcgtg accgggacga tcattcgtag

11221 tagttgctcc tgttcaccaa ggtggcgatc ggggcgctgg acgcgacacg actgtcggcc

11281 agcgcgacgg ccatgcgcgc cagcgtcggc tcggcgaaca accgcgacag cggcaaacgg

11341 cggccgagcc gagcctggat gcgcgtagca agttgcaccg ccaacaagga atgcccgccg

11401 atgtcgaaga aattgtcgtg tcggttgacc gtgctgatgc ccagcaatgc gcaccagatc

11461 tctgcaagtg cgcattcggt ctcgccttgt ggagggtcga tttccgcgat gccgcgttgc

11521 gcatcgtccg gcgcgggcag cgcacgcaca tccagcttgc cattggcggt gagtggccag

11581 gcctgcatga ccacgtaggc atccggcagc atgtagtcgg gcagcatgtc ggccagatgg

11641 ctgcgcaata cgttcgggtc gtgcgccgcg ctgtcgtcga tttgcgtgcc gatgacatag

11701 gcgaccagac gacgttgtcc ggcggtgtcc tcgcacacgc gcaccagcgc ctggtcgacc

11761 tgcggatgcg ttcgcagcgc cgcctcgatc tcgccgggtt ccagccggaa cccgcgaatc

11821 ttgctctgcg catcgcgtcg gcccagacat tcgagctggc cgtcggcacg ccagcggccc

11881 agatcgccgg tcctgtacat gcgcgctcca ggcactgcgc tgaaggggtc gggcacgaaa

11941 tgctcggcgg tcaatcccgg ttgccgcaga tagcccagcg ccagtccgag accggcgata

12001 tgcagttcgc cctgcatgcc gaccggcagc gcctggcgat gcgcatcgag cacgtacacg

12061 cgcagattga cgatcggcgt gccgatcggg atgcgttggc cagcgtctgg cacctcgctt

12121 actgcaacag cagtgacgaa ctgcgtggtt tcggtggggc cgtaggtctg caacaagttg

12181 tgtgctcgct gaccgcccag cagcgtggcg atcgcctgcg gatcggcgct gtcgccaccg

12241 gtgatcaggt agcgcagcgt cggcagcgtg cgcgccagtt cgctcgcgta aacgcgcagc

12301 accccggcga ccaggatcag cacgctgatt gcatgctgac gtgcgaagtc ggccagcacg

12361 cacggatcga gcagcacgtc ctccggcacc accaccacgc tgccgccgct cagcaacggc

12421 gcccagactt ccagggtgct ggagtcgaag gccgggttgg aggcgaaggc gaaacggtcg

12481 tcggcctgcc aacgggcgta gttctggtcg cgcaccaggt tgagcacggc ctggtgcggc

12541 accaagacgc ccttgggatt gccgctcgaa ccggaggtat acatgacata ggcgggcgcg

12601 gcggccggca acggcggggc cacgaacaca tcggtcggtt tccgggtcag cgcgatgtcc

12661 aggcagggcg ctgtcgtcca tggcggcgcg tccccgggcg cgtgcacgat ggcgcgggcc

12721 tgacagtccg cggccagttg ggcatgtctg gccgcgggtt ggcgcggatc cagcggcaca

12781 taggccgcgc ctgccttgag gattcctagc tgtgcggcca ccagttccgc actgcgcggc

12841 agcagcgtca ccacgcaatc gccagtgcgt acgccggctt cgatcagctg cagcgcgata

12901 cggttgctgt acgcatccag ctcgcgatag cgcagggtgc ggacgccatc gatgacggcg

12961 atggcgtccg gtgtgctgcg ggcctgggcc tggaacgcgg tgtggatcgt cggcagtggt

13021 gccaccgccc gtgcggtgtc gttccagtgc tgcagtgcac gccagccgtc tgcatccagc

13081 aacgccggcc ggtccaccgc cagcgtgggc gcgtcggcca gcgcggccag cagacgcgcg

13141 tagttatcga gatacgcctg catggtgctg cgctcgaaca gcgcggtggc atagcgcagc

13201 acgccggcga tccgcgtggg agcgatctgc atgtccagcg acagatcgaa ttgggcgacg

13261 tgatgatcgt tggggtaggc actcagttgc aggtccggca actccagtgc gcgtgccggc

13321 gtggtgtcgg aggagaacat cgtctggcac aacgggctat gcgacagact gcggaccggc

13381 cgcaccgcct cgatgatgcg ttcgaacggc agatcctggt gggactgtgc ggcaagcagc

13441 gtcgcctgga cctggttcag cagttccagg aatgtcggct gcgcggacag atcgatgcgc

13501 aacggcaggg tgttgacgaa gaagccgatc aacggttcca gttcgctgcg attgcggccc

13561 gccgatggtg tgccgatgac cagatcggtc tggccgctgt agcgggccag cagcgctgcc

13621 catccggcca gcaacagacc gaagatgctg atgccgtgct gcacggccag cgcctgcaga

13681 cgcaggctca gggtttcgtc caggaccagt tgcacatcgt cgccggccgt gtcctggagc

13741 ggtggtcgcg gtcgatcggt gggcagctcc agcaacgccg gcgcatcgtg cagctgggcg

13801 cgccagaact ccagctggcg ctgctgcagt tgattgtcga tccagcggtg ttgccaggcc

13861 gcgtagtcgg cgtactggat cggcagcggc ggcagcggat cgggggcatc gctgacgaaa

13921 gccgtataca acgcgccgag ctcctcgatc agcacgccga tggaccagcc gtcggagacg

13981 atgtgatgca tgctcagcag cagcacgtgc tcgtgttcgg ccaggcgcaa cagacggcct

14041 ctgaacaacg gaccgttggc gaggtcgaag ccggtgctgg cctcgttgcg ggcgtgcacg

14101 cgggccgcac tatccgggga cggctcgccg ctcaggtcga tcagttgcaa ggcgatgtcg

14161 cacggtgcag cgatgatctg tatcggcaga tcgtcgacgt tggcgaaatg ggtgcgcagg

14221 gtttcgtgac gcgccacgat gcggttcagg gccgcggtca gcgccggaac gtgcaagttg

14281 ccgctcaggt gcacgccgcc gcgcatcatg taggtcgcgc cggtacgcgg atcgagctgg

14341 ttgaggaacc acaggcgttg ctgggcgaag gacagcggca gcggtgcgct gcgatcggcc

14401 ggcacgatgg cgggcagggt gctggcggcg gcactgtcca gcgcctgcgt catttctgcc

14461 aggcgcggct gggcgaacag ggcagccagc ggcagttcga taccgagcac gctgcggatg

14521 cgcgagatca accgcacgcc gagcagcgag tgtccgccca gggcgaagaa gctgtcgtgg

14581 cggccgacgc gt

LOCUS AFHK01000191 8346 bp DNA linear BCT 09-MAY-2011

DEFINITION Xanthomonas oryzae X11-5A Xo_X11-5A_contig_191, whole genome

shotgun sequence.

ACCESSION AFHK01000191 [AFHK01000000](http://www.ncbi.nlm.nih.gov/sites/entrez?db=Nucleotide&cmd=Search&term=AFHK01000000)

VERSION AFHK01000191.1 GI:332357220

DBLINK Project: [66097](http://www.ncbi.nlm.nih.gov/bioproject/66097)

KEYWORDS WGS.

SOURCE Xanthomonas oryzae X11-5A

ORGANISM [Xanthomonas oryzae X11-5A](http://www.ncbi.nlm.nih.gov/Taxonomy/Browser/wwwtax.cgi?id=1009853)

Bacteria; Proteobacteria; Gammaproteobacteria; Xanthomonadales;

Xanthomonadaceae; Xanthomonas.

REFERENCE 1 (bases 1 to 8346)

AUTHORS Triplett,L.R., Hamilton,J.P., Buell,C.R., Tisserat,N.A.,

Verdier,V., Zink,F. and Leach,J.E.

TITLE Genomic Analysis of Xanthomonas oryzae from US Rice Reveals

Substantial Divergence from Known X. oryzae Pathovars

JOURNAL Appl. Environ. Microbiol. (2011) . 77(12):3930-7.

PUBMED [21515727](http://www.ncbi.nlm.nih.gov/pubmed/21515727)

COMMENT Base Quality-A custom perl script was used to clean and remove

reads with low complexity and low quality regions. Low quality

regions were defined as an average quality score of < 20 over a

10bp window along the read or > 2 'N' bases in the read. The Velvet parameters used in the final assembly for X11-5A were a hash length of 31, expected coverage of 19.58x, and a k-mer coverage cutoff of 9.79.

Contact Dr. Jan Leach for source DNA or cultures (Department of

BSPM, Colorado State University, Fort Collins, CO).

##Genome-Assembly-Data-START##

Assembly Method :: Velvet v. 0.7.53

Genome Coverage :: 70x

Sequencing Technology :: Illumina GAIIX

##Genome-Assembly-Data-END##

**Annotation was performed by Royer et al. (current manuscript)**

FEATURES Location/Qualifiers

source 1..8346

/organism="Xanthomonas oryzae X11-5A"

/mol_type="genomic DNA"

/strain="X11-5A"

/host="Oryza sativa cv. Lemont"

/db_xref="taxon:[1009853](http://www.ncbi.nlm.nih.gov/Taxonomy/Browser/wwwtax.cgi?id=1009853)"

/country="USA: Texas"

gene (<2..6787)

/gene="NRPS"

CDS (<2..6787)

/gene="NRPS"

/note="incomplete NRPS; no start codon"

/translation="LAALPLTTNGKLDRRALPAPGADALAARTYLAPEGEQEILLAALWSELLEIERIGRNDSFFALGGDSLLAIVLIERLQQSGWQLDIGALFNTPVLATLAGALRPAAIAPVPANRIALDCTRITPDVLPLVALTQTEIDAVVATVAGGAANVQDIYPLASLQEGLLFHHLQHIDSDPYVMPGLHAFASRAELDRLLAALNSVIARHDILRTAFVWHGVREAVQVVWRRAPLRLHFHVLESDDALGQLRRCLTSPQARIDPQQAPLLHAHLIHDALNQRWLLGMLYHHLVMDHTSMDLAIEEVGAFLAGTEHSLPAPLPFREFVARARPRDAAQTQTQTQEAFFSKLLGDLTQTTAPYGMLDVHGDGRTVHEARLQVPDALTQALRAQARALDVSMASLFHLAYALVVATTSGRDDVVFGTTLFGRMQGGGGIHRVLGMFLNTLPLRLRIDSTSVADAASTMQGLLAMLMQHEHAPLSLAQRCSGVAAPAPLFTALLNYRHVASNQASDTLPDWAGMQSLGASERTNYPLSLSVNDDERGFWLDVQTGPAAAPESVGQLMLEALRQLSRALTDAPHTALHDLPILPESMRADIVVGFNPTPGDAPAVGVAHLFAAQAARTPQAPALIDADGTILTYAELVQRAERLACRLRASGIGPEARVAVCMQRGIALVATLLGVFQAGAAYVPLDPEYPAQRRADILRDCQPQILVATSACAHALPPQVAVPLLWYDAPDDAAIAAPAAVAAPHATQLAYLIYTSGSSGRPKGVMVEHGALAAYCVAAAELFGLSGDDCVLQQNSINFDLSLEELLPALIAGACLRLAPLPLDAVAAPTPASVLHLTSAHWHALVSAWTQAPEQARQQLQQVRLINITGDVLSPYQLQQWHGLGLQAIALINTYGPTETAVSCTAARLCSEHANATRISIGTALRHVRLYVLDARHRPLPIGVAGELYVAGVQVGRGYHARPALSAQRFLPDPFALGAGQRMYRTGDLARWNADGSLEFLGRNDQQLKLRGFRVELGEIENVLLDYPGVREAAVCARHDATATTRLLAYVVADAIQPDRLHAHLAQRLPDYMLPSAYVRLDALPLTPNRKLDRNALPVPDAGTQCDTQAHVPPHGPFEQTLAMLWADLLGCDHVGRRDSFFALGGHSLLAVRLASRLRSHFGVAVGLADIFAHPRLADFASCVAAAAANSSPPILLVPRDAPLPLSFAQQRLWFLSQLDPQTDLAYLVSTGVHLDGALDLPALCKALDRIVARHETLRTRIVANAGQPQQEVLPADTGFALRHSDLSALDDPASRVRQLCADERQTAFAPGAPLVRGHLLQLATQRHVLLITLHHLVCDGWSMGVLIEELGMLYAAYTRQADDPLPPLALQYADVAVWQRRWLEGDVLQRQLDYWRDRLHDAPRRLALPTDHTRPARQDFHGDNIAFSLPAAVSAGLHALSLRHGVTPFMTLLAAWALLLHRYSGQDEVVIGSPLAGRDRSELEPLIGFFVNTVALRIDLSGRPTIAELLERVRAAVLGAQAHQDLPFDRVIDAVQPSRTLAHAPLCQALFSSDTPPSGALEMPGLQLQPLQGGTPVALADLALEMRVGRDSIAGSLIYATALFERATLQRHLAYYQALLCAFADTSGMQVADDLPLPAPARQHLSQAPLGEMLPSMPTSVHAAFAQQVQRTPTAPAVCDGTLQLSYADLEARSDALAQQLLAAGVGAGAMVATALPRSAALVIAQLAILKLGAAYLPLDPQQPALRLAQLVEDCHAVALLHPPQTDPVWAGALHCLPVDVEALAATATRFAAATLPSNAPAYVMSTSGSTGVPKGVAIAHRGILNLVLAPDYADWNAQDRFAFASNPAFDSTTLEVWAPLLCGACVVVVPQAVVLDPGALAGFIRRHAISVLILVAGVLRAYAPQLAASLPKLRYLITGGDIADPHALALLLGSANAPEQVLQTYGPTEATQFATTLALREPPPPDRRVPIGQAIRHMQAHLLDARGTPVPIGLPGQLYLAGAGLAQGYVGRPGATAERFVPDPFADEPGARMYRTGDLARWREDGSLDFLGRADAQLKLRGFRIEPGEIEAVLASHPQVAQAVVHVRFDSAHQPRLLAYIVADNPDENAAVCQRLRTWLAERLPDYMQPTAYIPLQRLPLTANGKLDRDALPSAAGQACDAAAMQAPQGASEQALARLWRDLIGLECVGRHDDFFEVGGHSLLAVQLIARIESQLQRRIAVSQLFAHSSLAALAALLDATEMHNDDMITTSDREDYFA"

gene complement (7048..8028)

/gene="AraC"

CDS complement (7048..8028)

/gene="AraC"

/note="transcriptional regulator"

/translation=" VQPASSLDIFNSFSTSQIHPGERVHRWLEWMARSVGAEIDEAPGQHKCSLIASESALQGSITQLRLGDLHISKVSANAHALELKLGGAARRDAHVLIAFQCKGTSTLVNDGLSLELRAGEMLALSIRQELRIVNPSNVQQHFIWFEKPRQANRMMEGVVSHRRFGPAPMQRLAFSFVERLFNEPGLCNPESGQFFAQAMIKLLELALKETAVAKPQVEPNRPSREMVLQFVERNLRDPKLSPDTISRALGWSKRTIYRAFKGGNGESLNGYLWRRRIEQCAQELRGPSDLSITGIAYSFGFSSCPHFSRLFKQQMGASPLQYRRGAY

ORIGIN

1 actcgctgcc ctgccgctga ccaccaacgg caagctcgat cgccgcgcac tgccagcgcc

61 gggcgcggat gccctcgccg cgcggaccta tctggcacct gaaggcgagc aggaaatcct

121 gctggctgcg ctgtggagcg aattgctcga gatcgagcgg atcggccgca acgacagctt

181 tttcgccctt ggcggcgatt cgctattggc catcgtgttg atcgagcgcc tgcagcaatc

241 cggttggcag ctggacatcg gcgcgctgtt caatacaccg gtgctggcca cgctggcagg

301 cgcgctgcgt ccggcggcaa tcgcgccggt gccggctaac cggatcgcgc ttgattgcac

361 gcggatcaca cccgatgtat tgccgctggt cgcgctgacc cagactgaga tcgatgcggt

421 ggtggcaacc gtcgccggcg gcgctgccaa cgtgcaggac atctatccgt tggcctcgct

481 gcaggaaggc ctgctgttcc accacttgca acacatcgac agcgacccct atgtcatgcc

541 gggtctgcac gcattcgcca gccgtgcaga gctcgatcgc ttactggccg cattgaatag

601 cgtgatcgcg cgtcacgaca tcctgcgtac cgcctttgtc tggcatggcg tgcgtgaagc

661 ggtgcaagtg gtctggcgac gggcgccgct gcggctgcat ttccatgtgc tggagagcga

721 tgatgcactg gggcaactgc ggcgctgtct gacatcgccg caggcgcgta tcgacccgca

781 acaggcgccg ctgctgcatg cgcacctgat ccacgatgcg ctcaaccagc gttggttgct

841 cggcatgctt taccaccacc tggtgatgga ccacacctcg atggatctgg ccatcgagga

901 agtgggcgcg ttcctggccg gcaccgaaca ctccctgccg gcgccactgc cgttccgcga

961 attcgtcgcc cgcgcccgcc cgcgcgacgc ggcgcagacg cagacgcaga cgcaggaagc

1021 gttcttcagc aaactgctgg gcgatctcac gcagaccacc gcgccctacg gcatgctgga

1081 tgtgcacggc gatggccgca cggtgcacga agcgcgcctg caggttcccg acgcgctgac

1141 ccaggcgctg cgtgcgcagg cgcgtgcgtt ggatgtgagc atggcgagcc tgttccatct

1201 cgcctacgca ttggtggtgg ctaccaccag cggacgcgac gacgtggtgt tcggcaccac

1261 gctgttcggc cgcatgcagg gcggtggcgg catccaccgc gtgctgggca tgttcctcaa

1321 taccttgccg ctgcgcctgc gcatcgactc gaccagcgtg gctgacgccg ccagcacgat

1381 gcagggcttg ttggcgatgc tcatgcagca cgaacacgcg ccgttgagtc tggctcagcg

1441 ttgcagcggc gtcgccgcac ctgcgccgtt gttcaccgcc ttgctcaact atcgccacgt

1501 cgccagcaac caggcgtcgg acacgctgcc ggactgggct ggcatgcaat cgctgggcgc

1561 cagcgagcgc accaactatc cattgtcgtt gtcggtgaac gatgacgagc gcggcttttg

1621 gttggatgtg cagaccggcc ccgctgccgc acccgagtcg gtcggccagc tcatgctcga

1681 agccttgcgg caactttcgc gagcgctgac cgatgcaccg cacaccgcct tgcatgatct

1741 gcctatcctg ccggagtcca tgcgtgccga catcgtggtt ggcttcaacc caacgcccgg

1801 cgacgcaccc gcagtgggcg tcgcgcacct gttcgccgca caggcggcgc gcacgccgca

1861 ggcgccggcg ttgatcgatg cagacggcac catcctcacc tatgccgaac tcgttcaacg

1921 cgccgagcgc ctggcctgcc gcctgcgcgc ctcgggcatc gggccggaag cgcgggtcgc

1981 ggtgtgcatg cagcgcggca tcgctctggt ggcaacgctg ctgggcgtgt tccaggctgg

2041 cgcggcctac gtgccgctgg acccggagta tcccgcgcaa cgccgtgccg acatcctgcg

2101 tgactgccag ccgcagatcc tggtggccac ctcggcatgc gcacacgcgc tgccgccaca

2161 ggtcgcagtg ccgctgctgt ggtacgacgc ccctgacgat gcggcgattg ccgcaccggc

2221 cgctgttgcc gcgccgcatg ccacacaatt ggcctatctg atctacacct ccggttccag

2281 cggtcgtccc aagggagtga tggtcgagca cggcgcgttg gccgcctact gcgtcgccgc

2341 cgccgagctg tttggcctga gcggcgacga ctgcgttctg cagcaaaatt cgatcaactt

2401 cgatctgtcg ttggaagaac tgctgcccgc gctgatcgca ggtgcctgcc ttcggctggc

2461 gccactgccg ctggacgcag tcgccgcgcc tacacccgcc agcgtgctgc atctgaccag

2521 cgcgcattgg cacgcgctgg tgagcgcatg gacgcaagcg cccgaacaag cgcgtcagca

2581 actccagcaa gtgcgattga tcaacatcac cggcgacgtt ctgtcgcctt accaactgca

2641 gcaatggcat gggctcgggc tgcaggcaat cgcgctgatc aacacctatg gccccaccga

2701 aaccgcggtt tcctgcaccg ccgcccgcct gtgcagcgaa cacgccaacg ccacccgcat

2761 cagcatcggc accgcgctgc gccatgtgcg tctgtacgtg ctcgatgctc gccatcgtcc

2821 gctgccaatc ggcgtggccg gcgagctgta tgtcgcaggc gtccaggtcg gtcgtggcta

2881 ccacgcacgg ccggcactga gcgcgcaacg tttcctgccc gacccgttcg cgctcggcgc

2941 tgggcagcgc atgtatcgca cgggcgatct ggcgcgctgg aacgccgacg gcagcctgga

3001 gttcctcgga cgcaacgacc agcagctcaa gctgcgcgga ttccgcgtcg aactgggcga

3061 aatcgaaaac gtgctactgg actatccagg cgtgcgcgag gcggcggtct gcgcgcgcca

3121 cgacgcgacg gcaacgacgc gtttgctcgc ctacgtcgtc gccgacgcga tccaaccgga

3181 ccggctgcat gcgcacctgg cccagcgcct gcccgactac atgctgccca gtgcctatgt

3241 gcggctggac gcactgccgc taacgcccaa tcgcaagctg gatcgcaacg cgttgccagt

3301 gcctgacgcc ggaacgcaat gcgataccca agcccatgtg ccgccgcacg gaccgttcga

3361 gcagaccctg gccatgctgt gggccgacct gctcggctgc gatcacgtcg gtcgccggga

3421 cagcttcttc gcgctgggcg ggcactcgtt gcttgcggta cgcctggcat cgcggctgcg

3481 cagccacttc ggcgtcgctg tcggcctggc cgacatcttc gcccatccgc gtctggccga

3541 tttcgccagc tgcgtcgcgg cggccgcagc gaacagctcg ccgccgatcc tgttggtccc

3601 acgggacgcg ccgctgccgt tgtcctttgc ccaacagcgg ttgtggtttc tgtcgcaact

3661 ggacccgcaa accgaccttg cctatctggt cagcaccggc gtgcatctgg acggtgcgct

3721 ggacctgccg gcgctgtgca aggcgctgga ccgcatcgtc gcgcgccatg aaacgctgcg

3781 cacgcgcatc gtcgccaatg cggggcaacc gcagcaagag gtgttgcccg ccgacaccgg

3841 gttcgccctg cgccacagcg acttgtcggc gctggacgat cctgcgtcgc gcgtacggca

3901 attgtgtgca gacgaacgtc agaccgcttt cgcaccgggc gccccgttgg tacgtggcca

3961 tctgctgcag ttggcgacgc agcggcacgt gctgttgatc accctgcatc acctggtgtg

4021 cgatggctgg tcgatgggcg tgctgatcga agagctcgga atgctgtatg ccgcctacac

4081 ccggcaagca gacgatccgc tgccgccgct ggcgctgcaa tacgccgatg ttgcggtatg

4141 gcagcgccgc tggctggagg gcgacgtgct gcaacgacag ctcgactatt ggcgcgatcg

4201 cctgcacgac gcgccgcggc ggctggcgct gccgaccgac cacacgcgtc cggcacgaca

4261 ggacttccac ggcgacaaca tcgccttctc gctgcctgcg gcggtgagcg caggcttgca

4321 tgcgctgagc ctgcgccatg gcgtcacccc gttcatgacg ctgctcgccg cctgggcgct

4381 gctgctacac cgctacagcg gccaggacga agtggtgatc ggcagcccgc ttgccggccg

4441 cgatcgcagc gagctcgaac ccttgatcgg ctttttcgtc aacaccgttg cgctgcgcat

4501 cgacttgtcc ggacgaccca ccatcgcgga gttgctggaa cgtgtccgcg ccgcggtgct

4561 cggtgcgcag gcgcaccaag acctgccatt cgaccgcgtc atcgacgcgg tccagcctag

4621 ccgcacgctc gcccatgccc ccttgtgcca ggcgctgttt tcgtcggaca ccccgccgtc

4681 gggtgcgctg gagatgcccg gcctgcagct gcaaccactg caaggcggca caccggtcgc

4741 cttggccgat cttgccctgg aaatgcgcgt cggccgcgac agcatcgccg gctcgctgat

4801 ctacgccacg gcactgttcg agcgcgctac gctgcagcgg catctggctt attaccaagc

4861 cctgctgtgc gccttcgccg ataccagcgg gatgcaggtg gccgacgatc tgccgctgcc

4921 agcgcctgcg cggcagcatc tatcgcaggc cccgctcggc gaaatgctgc cgtcgatgcc

4981 gaccagcgtg catgcagcct ttgcgcaaca ggtgcaacgc acgccgacgg cacccgccgt

5041 ttgcgacggg acgctgcagc tgagttacgc cgatcttgaa gcgcgctcgg atgctctagc

5101 gcagcagtta cttgcagccg gtgtcggcgc cggcgcgatg gtggcgacgg ccctgccgcg

5161 cagcgccgcc ctggtgatcg cgcaactggc cattctcaaa ctgggtgcgg cttacctgcc

5221 gctggacccg caacagccag cgctgcggct ggcccagttg gtagaggatt gccatgccgt

5281 cgcgctgctg catccgccac agacagatcc ggtctgggcc ggcgcactgc actgtctgcc

5341 cgtcgatgtg gaggcgctcg ctgccaccgc aacgcgcttt gccgcggcaa cgctaccgag

5401 caacgcgcct gcctacgtga tgtccacctc cggctccacc ggcgtgccca agggcgtggc

5461 cattgcccat cgcggcatcc tcaatctggt gctcgcgccg gactacgccg attggaacgc

5521 gcaggaccgt ttcgccttcg cctcgaaccc cgcgttcgat tccaccaccc tggaagtctg

5581 ggcgccgctg ttgtgcggcg cctgcgtggt cgtcgtgccg caggcggtgg tgctcgatcc

5641 cggcgccttg gcgggcttta tccgcaggca tgcaatcagc gtcctgatcc tggtggccgg

5701 tgtgttgcgg gcgtacgcac cgcagttggc cgcctccctg cccaagctgc gttatctgat

5761 taccggcggc gatatcgccg acccgcatgc gctggcgctg ctactgggct ctgccaatgc

5821 gccggagcag gtgctgcaaa cctacggacc gaccgaggct acccagttcg ccaccaccct

5881 ggccctgcga gagccaccgc cgccggatcg gcgcgttccg atcgggcagg cgatccgcca

5941 catgcaggcg cacctgctcg acgcacgcgg aacaccggtg ccgatcggtc ttccgggcca

6001 gctgtacctg gcgggtgcgg gtctggcgca gggctatgtc ggccgccccg gtgccaccgc

6061 cgaacgcttc gtgccggatc cgttcgccga cgagccggga gcgcgcatgt atcgcaccgg

6121 cgacctggcc cgctggcggg aagacggcag cctggatttc ctcgggcgcg ccgacgcgca

6181 actaaagcta cgcggctttc gcatcgaacc gggcgagatc gaagcggtgc tcgccagcca

6241 cccgcaagta gcgcaggcgg tcgtccatgt gcggttcgat tctgcccacc aaccgcgcct

6301 gctggcgtac atcgtggccg acaatccaga cgaaaacgcc gctgtgtgcc agcgtctgcg

6361 cacctggctg gccgagcgat tgccggacta catgcaaccg accgcataca tcccgctgca

6421 acgactgcca ctgacagcga acggcaaact cgatcgggac gctttgccga gtgccgctgg

6481 ccaggcatgc gacgccgccg cgatgcaggc accgcaaggg gccagcgaac aggccttggc

6541 gcgtctgtgg cgcgatctga tcgggctgga gtgcgtcggc cgccatgacg acttctttga

6601 agtcggcggt cattccttgc tggcggtcca attgattgca cgcatcgaat cgcagttgca

6661 gcgacgcatc gccgtctcgc aactgttcgc ccattccagc ctggccgcgc tggcggccct

6721 gttggacgcg accgagatgc acaacgacga catgatcacg accagcgacc gcgaggacta

6781 cttcgcatga agcggtgcgt agcacagccg cgatcgtacg ccgagggacg cggcacaacg

6841 caacgaagag gcatgccgcg cacacgcgcc ttattgcggt ttggaggatg caatcggcat

6901 gcggaacgtc gaaccaatgc aacgcgggtc cgaacaaggg gctcatgcgg catgcggcga

6961 cgcagaaatc gccgcatgca accgggcgaa agcgaacgct gagcgtcgcg ccctattcgc

7021 tatcggccat ggcgcgcctg ccgctcaata agcgccacgc cgatactgca atggcgacgc

7081 gcccatctgc tgcttgaaca agcggctgaa gtgtgggcaa ctgctgaacc cgaacgaata

7141 ggcgattccc gtaatactca aatcactcgg ccctcgcagt tcctgcgcgc actgttcaat

7201 gcgtcgtcgc cacaaatagc cattcaggct ttcgccgttt ccgcctttga atgcgcggta

7261 aatcgtgcgt ttactccacc ctaaggcgcg cgagatcgtg tcaggcgaca acttcggatc

7321 gcgcagattg cgctcgacga actgcaacac catttcacgc gagggccggt taggttccac

7381 ctgaggcttg gccacggcag tttccttcag cgccagctcc agcagcttga tcatggcttg

7441 cgcaaagaat tggccgctct ccgggttgca cagaccaggc tcgttgaaca agcgctccac

7501 gaagctgaat gccagacgct gcatcggtgc cggcccaaag cgccggtgcg acaccactcc

7561 ctccatcatc ctgttcgcct gccgaggctt ctcgaaccag atgaaatgct gttggacatt

7621 ggatggattg acgatgcgca actcctgccg gatggacagc gccagcatct cccccgctct

7681 cagctccaac gacaggccat cgttcaccag cgtgctggtg cccttgcact gaaacgcaat

7741 cagcacgtgg gcatcacgcc tggcggcgcc accgagcttc aactcaagcg catgcgcatt

7801 agccgatacc ttggatatat gtaaatcgcc caaacgcagt tgggtaatgg agccttgcag

7861 ggccgattcg gaagcaatca gcgaacactt gtgctgcccg ggtgcctcat cgatctctgc

7921 accgaccgaa cgcgccatcc actcaagcca gcgatgcacg cgctcgccag gatggatttg

7981 cgaggttgaa aacgaattga atatatccag tgatgatgcg ggctgcacat ccgttgcgca

8041 atacgaaggt tccacccgtg cgcggcatga cgtattcatc ccatccggcc tctcaggaat

8101 cagatcgagg ggacgggatg ctaggcaggc tttgtgtcaa ctttatgaag acataaaaaa

8161 taaaaatatg tgtgaatttc attaggtgat agcagctaat cccgagcgaa tatcgcaacc

8221 aaattttaaa taaaatatta atgaaatatc catttttcca tatgaatcta cattgcattt

8281 tgaggagttc aattaacgcg catgatctga tttagcgcgg ctaacaaacc gtagcgagca

8341 gctgtc

LOCUS AFHK01000114 13402 bp DNA linear BCT 09-MAY-2011

DEFINITION Xanthomonas oryzae X11-5A Xo_X11-5A_contig_114, whole genome

shotgun sequence.

ACCESSION AFHK01000114 [AFHK01000000](http://www.ncbi.nlm.nih.gov/sites/entrez?db=Nucleotide&cmd=Search&term=AFHK01000000)

VERSION AFHK01000114.1 GI:332357374

DBLINK Project: [66097](http://www.ncbi.nlm.nih.gov/bioproject/66097)

KEYWORDS WGS.

SOURCE Xanthomonas oryzae X11-5A

ORGANISM [Xanthomonas oryzae X11-5A](http://www.ncbi.nlm.nih.gov/Taxonomy/Browser/wwwtax.cgi?id=1009853)

Bacteria; Proteobacteria; Gammaproteobacteria; Xanthomonadales;

Xanthomonadaceae; Xanthomonas.

REFERENCE 1 (bases 1 to 13402)

AUTHORS Triplett,L.R., Hamilton,J.P., Buell,C.R., Tisserat,N.A.,

Verdier,V., Zink,F. and Leach,J.E.

TITLE Genomic Analysis of Xanthomonas oryzae from US Rice Reveals

Substantial Divergence from Known X. oryzae Pathovars

JOURNAL Appl. Environ. Microbiol. (2011). 77(12):3930-7.

PUBMED [21515727](http://www.ncbi.nlm.nih.gov/pubmed/21515727)

COMMENT Base Quality-A custom perl script was used to clean and remove

reads with low complexity and low quality regions. Low quality

regions were defined as an average quality score of < 20 over a

10bp window along the read or > 2 'N' bases in the read. The Velvet parameters used in the final assembly for X11-5A were a hash length of 31, expected coverage of 19.58x, and a k-mer coverage cutoff of 9.79.

Contact Dr. Jan Leach for source DNA or cultures (Department of

BSPM, Colorado State University, Fort Collins, CO).

##Genome-Assembly-Data-START##

Assembly Method :: Velvet v. 0.7.53

Genome Coverage :: 70x

Sequencing Technology :: Illumina GAIIX

##Genome-Assembly-Data-END##

**Annotation was performed by Royer et al. (current manuscript)**

FEATURES Location/Qualifiers

source 1..13402

/organism="Xanthomonas oryzae X11-5A"

/mol_type="genomic DNA"

/strain="X11-5A"

/host="Oryza sativa cv. Lemont"

/db_xref="taxon:[1009853](http://www.ncbi.nlm.nih.gov/Taxonomy/Browser/wwwtax.cgi?id=1009853)"

/country="USA: Texas"

gene complement (<1..4941)

/gene="NRPS"

CDS complement (<1..4941)

/gene="NRPS"

/note= incomplete NRPS (no stop codon)

/codon_start=1

/translation="MTPFDRAAAVGDTVHPLSSAQQDIWLGQLFAPDQPSYTIGCVMTFDGTLHRAVWEQAVAAVIARHDALRMLLVEGAPLPNQRVLETLPFALPWHDYSADTDGEQRVHEHIQQAMKRSFAHYGKPLWDIQWLQATATRGYCLYLCHHIVIDGVSLGMLSQQVIDCYNQRLRGEPDQPPAPSYLQALDADLAYIDSTRYQRDLDYWRQALRARPEPLYPSTSAALGRQPVVQRSFTLQPDLLPALTALAERLGGSVTTLIVACVASCLARLNHHHRAPLALGLTVHNRHTRIERDMFGMLSTQLPLYLAVQPHADLAAAMRGVSSALRPAMRHARLHLRHALRELGETGQLAPRPFDISVSVEDFSAFGDVPIEGGVRSMMPLHGDYEDAALGIVVWRYNAACPTTVLFNIDPQRVPLPLAERILAALQHMLLALQEAPHAPLSQLPLLSADQCAQLQGFNASTIDLGDSDCLHHQIQAQAQRTPQAIALVDDALALSYADLDARANQLAHHLIALGVAPDVRVAVCLPRGVDLVVALLAVLKAGGAYVPLDPIYPPARLGFMLRDSAARCLLTHTALTDLLPDNQLARVWIDDTASWAMHPAYPPATQDLSPHHLAYVIYTSGSSGQPKGVMISHHALTQFLAALQIDLPLSPEDRVLAVTTVCFDIAGLELFAPLVHGARVVIAGDQAIQEPADWSQLVDRHAISVLQATPAFWQMLLDTGWQSRPDLRLLCGGEALRPELAQRLRAGGGQLCNLYGPTEATIWTSRHPVLGDEPGSVVPLGRPLANTRLWVLDASHQWVPQGVAGEIYIAGPQLARGYLGRPDLTAERFVPDPFAGQPGQRMYRTGDLARWRADGVLEYLGRNDEQVKIRGFRIELSEIAAALRACAGVRQAAVIAREDTPGNKRLVAYVVGGTDVVPDTEAWRTQLGARLPDYMLPAAYVPLEALPLTANGKLDRRALPAPDADAIAPRGYVAPEGACETELAALWRDLLGVERVGRHDSFFALGGHSLLGVQLIARIRSALGLELPLATLFAQPRLAELAGALVHATPSALPAIVPVERCAPLPLSFAQQRLWVLGQFDTRADLAYLMPGTVTLRGALDVAALRQALNRILARHETLRTRFLATEDGAAQVIDPAETGVALEYIDLRHAADPHAAAQRHAEQESSLALDREQGPLLRARLLQRADDDHLLLVTLHHLVADGWSIGVLLHELGTLYSAFVHGQPDPLPPLPIQYADYALWQRRWIDAARLQQQRQFWLDHLRDAPALLALPTDRPRPPGQDYAGAAIAVAIDAARTRALLALSHRHAATLFMTLLAAWGTLLARLAGQDQVVVGTPVAQRTRTETAALIGLFVNTQALHLDLRADPSVTELLAQVRATALAAQAHQDLPFEQLIEALHPERSLAHAPVFQVMFTWQNTPTVELAMSGLHSEVLPSATHDAKYDLDLDLRLQDGCIVGSLRFATALFDTDTVQRQWDSFGVLLDGLLGDQQARVRRLPLLPPAQRQQLHAFHAGDRIGDTSAAAPGNVVQWFAQQAAATPQAIALVCGDATLSYQQLERRSNQLAHRLIALGARPDRCVALCLPRGIAQIIAVLAVLKAGAAYLPLEHSQPDERRARVIADARPVLLLVDSTDRVSAATDLAIPAV"

gene complement (4941..13400>)

/gene="NRPS"

CDS complement (4941..13400>)

/gene="NRPS"

/note= incomplete NRPS (no start codon)

/codon_start=1

/translation="LTTNGKLDRHALPAPDADALATQAHVAPEGELETVLAKLWSELIGIERIGRHDDFFALGGHSLLAVRLISRIRTSLGLELPLGTLFAHPRLTELAQALSSAAASTLPTIVPIDRAQPLPLSFAQQRLWFLAQFDAQADLAYLMPNGLHLRGGLDRDALRRALDRIVARHETLRTRIALHNDEPVQIIDADNVGLRLSEHDLSAYPDPDAQVRHHAEEETLTGFDPACDTLARGRLLRLADDHHVLLITLHHLISDGWSMSLLVRELSALYAAFAHGLPDPLPPLSLQYADIVIWQRRWISGEVLQRQREFWVEHLHDAPRVVQLPTDRVRPALPDHGGDAIEIALDAELSAALKALSQRHGTTVFMTMLAAWGVLLARLSGQHQVVIGTPIANRYRSEVEPLIGLFANTQALHIDLRGNPSVAELLAQVRATALAAQDHQDLPFEQVIEALNPVRSLAHHPVFQVMFAWQNTPVSDIALPGLALQPLPQMLAARKFDLELTLEERHSCIVGSIGYATALFERSTIQRVLACFMHLLKGMSTQDSVRVAQLPWLPADQCDQLLRTFNAHEPILRPVATLTRALHAQVQRAPRAAALIDGKLVVSYAELQARAAVLAQRLLASGVTPGACVALLLPRSALLIVAELAVLMCGAAYVALDPAQPPARLRALLDDCAAAALLCDTDCPLDAVCLPRLEVEWAALTGEAVAPHVQLTSASIAYVVYTSGSSGTPKGVAVSQAAVLAFALNQQHEPLQPQDRVAFLANPAFDASTFEVWATLLHGAAIVVVDQPTLLDPIALAQHLTAHDVSILHLTAGLLPGYWPALRTLLPTLRCLLTGGDSVDAGSVAALLAQAPPQRLLHCYGPTEATTFSVVHAVATVADDAARIPLGRPLPGSRVYVLDRHGQPTPIGVAGELHIAGAQLAQGYLHRPALTAERFVPDPFAAQPGERMYKTGDLACWRADGTLNFLGRNDAQVKIRGFRIEPGEIEAALRNCDGVREAVVVARDDTGQKRLVAYLAGDASMLEPATLRSQLATRLPDHMLPAAYVQLAALPLTPNGKLDRAALPAPDDHALDLHAYVAPSGDLEHVLATLWSELLGVEQVGRHDDFFALGGHSLLAIKLIERLRRLGWQIDVRALFAQPTLAGLAVNLQTASAIVVPPNRIAPACTDITPDVLPLISLTQAEIDAIVSSVDGGAANVQDIYPLAPLQEGLLFHHLSDPLADPYLHSSVLGFPSKEQLDRFLDALDQVIARHDILRTGFLWQRLSVPVQVVWREAILPRRAHRFDGPDPAAELLTWLHTPEAAPSLQHAPLIHAHLAHDATTGRWLLGLQQHHLVMDHTTLELVIEEVRAHLAGRQQQLPTPLPFRDFVAHAHADVSEQEHKAFFSAMLADIDAPTAPFGVLAPVRDPAALRFLRVSLPATLATAVRTQARQHGVSAASLFHLAYALLLARSSGSTEAVFATLLFGRMHASAGVDRVLGLFLNTLPIRLNTAHGSVLDAVRQTQLCLAQLLHHEHAPLALAQRCGTLDPSTPLLNALLNYRYSGGSAVLGVEPEVEHVAALDGVHEIAGQERTHYPLLVSVNDEQTSGGFSLDVQCVQQIGTERIAAMLLQTLQGLVQALQHAPQTALHALDLLPENERAQLRHFNDTTADLGGSGYLHRAIALQAQRTPQAVALVEDDVELSYAALDNRANQLAHHLIALGVVPEDRVAVCLPRGIDLVIALLAALKAGAAFMPLDPDAPSNRLERMLADARPRVLLARRPTASPLALSDGLHTVLLDTDQARWNNAATHAPVVAILHPQHPAYVLYTSGSTGQPKGVITTHAGIDNRLTWGQQALELLPTQTVLQKTPVGFDVCVWELFWPLRVGARLVLARHEGHKDPAYLIALIEHTGIDTVHFVPSMLRVFLDVLPAGACTSLQRIVCSGEALTADLAQAVRTRLPRARLYNLYGPTEASVEVSVWECSDADASSVPIGRPIANTRLHVLDAQRARAPIGVPGELQIAGVQLARGYLGRPDLTAERFVPDPFADRPGQRMYRTGDLARWRVDGALEYLGRNDAQIKLRGVRIELGEIETALRGCEGVREAVAIVRDDIGEPRLVAYLVGDAEHLTADALRTQLTACLPDVMLPSAYVHLDALPVSVNGKLDRRALPAPDATAIVCDAFVPPANEIERSLAKLWASVLGPQRIGRDDHFFERGGHSLSAMRLMTSAHRVGLPLTLRLLYAHPTLRAQADCLLGGAHRWGTRALAARRQGTRPPVFVVPTGIADVAYAFELAAHLAAEIPVYALPWPDPLPATLEALAAHMVGMIQVVQPQGPYHLLGYSSGGLLAYAIAQHFGMHDQPVAFLGLLDCDCPDRAPDPLPLDEAVKQRLLTRVQTMLEHGTDRDDAINAALRDLLANAEQASLQELVEQASEDAALNTLAAQEQTSLAKVIATSRMTATFERMWPTYWVQALAPQCPLTVFYASEPLPGDVTLGWTRLLPAGQIRSIAVPGTHVSLIEAEQLPRLGQIVSTIIASSSADVVRHRDEPAFALQSSHAHAPVVVCVPGAGDSVTSFVDLSSALGDACNVIGMQPRGTDGCQLPFGSVELAAQQYLDALPAVTAGAPQLHLVGHSFGGWVVYEMALRLHALGRPAASLTLIDTRPPNRARVPHDFSRDNILDYFLDALQLRLHAPLGIDRQALHRLGQNALLQALHRLMVEHGLMPPRSRPDSVRGSLATFAHCCRTCYIPARPYPGTLQLVLVADTRLQPEQQAAERLRLRQAWAAHAADLRPWHGPGNHMTVLAKPHSQLLAQWWMSSVGDHARPATASTPLSPGTTVIA"

ORIGIN

1 cacggccggt atcgccagat ccgtcgctgc gctcacgcgg tcggtcgaat cgaccagcaa

61 cagcaccggc cgggcatcgg ctattaccct ggcgcggcgt tcgtcgggct ggctgtgttc

121 cagcggcaag taggcggcgc cggccttgag cacggccagc acggcgatga tctgcgcgat

181 gccgcgtggc aggcacagcg ccacacagcg atcgggccgc gcgccgagcg cgatcaaccg

241 atgcgccaac tggttggatc gacgctccag ttgctggtag ctgagcgtgg cgtcgccgca

301 caccagggcg atggcctgcg gtgtcgccgc ggcctgctgc gcgaaccact gcaccacgtt

361 gccgggcgcg gccgcggagg tgtcgccgat acggtcgcca gcgtggaagg cgtgcagctg

421 ctggcgctgt gccggaggga gcagcggcaa gcgacgcacg cgcgcctgtt ggtcgccgag

481 cagtccgtcg agcagtacac cgaaactgtc ccactgccgc tggaccgtgt cggtatcgaa

541 cagcgcggtg gcgaagcgca ggctgccgac gatgcagccg tcttgcagcc gcagatccag

601 gtccaggtcg tacttggcat cgtgggtcgc gctgggcaag acctcgctgt gcagaccgga

661 catcgccagt tcgaccgtcg gggtgttctg ccaggtgaac atcacctgga acaccggcgc

721 atgggccagg ctgcgttcgg gatggagcgc ttcgatcaac tgctcgaacg gcaggtcctg

781 gtgcgcctgc gcggccagtg cggtagcgcg cacctgggcc agcagctcgg tcaccgacgg

841 gtcggcgcgt aggtccagat gcagggcctg ggtgttgacg aacaggccga tcagcgcggc

901 ggtctcggtc cgcgtgcgct gggcgacggg cgtgccgacg accacctggt cctgcccggc

961 caggcgcgcc agcagcgtgc cccaggcggc gagcagggtc atgaacagcg tggcggcatg

1021 gcgatggctc agcgcaagca gcgcccgggt acgcgcagca tcgatggcca ccgcgatggc

1081 ggcgccggca tagtcctgcc cgggcggacg cgggcgatcg gtgggcaggg ccagcagcgc

1141 cggtgcgtcg cgcaggtgat cgagccagaa ctggcgctgc tgctgcagcc gcgccgcatc

1201 gatccagcgg cgctgccaca aggcgtagtc ggcgtactgg atcggcagcg gcggcagcgg

1261 atcgggctgg ccgtggacga acgcgctgta cagcgtgccg agctcgtgca gcagcacgcc

1321 gatcgaccag ccatcggcaa ccagatggtg cagggtgacc agtagcagat gatcgtcgtc

1381 ggcgcgttgc agcaggcgcg cgcgcagcag cggcccctgt tcgcggtcga gggcgaggct

1441 gctctcctgc tcggcatggc gctgggcggc ggcgtgcgga tcggcggcgt ggcgcagatc

1501 gatgtattcc agcgccacgc ccgtctcggc cggatcgatg acctgtgcgg cgccgtcttc

1561 ggtggcgagg aagcgggtgc gcaaggtttc atggcgggcg aggatgcggt tcagcgcctg

1621 gcgcagcgca gcgacatcga gcgcgccgcg caatgtgacc gtgccgggca tcaggtaagc

1681 caggtcggca cgcgtatcga actggcccag cacccacagc cgctgctggg caaaggacag

1741 cggcagcggt gcgcagcgtt caaccggcac gatggccggc aaggcgctgg gggtggcgtg

1801 gacgagcgcg ccggccagtt cagccaggcg cggctgggcg aacaaagtgg ccaacggcag

1861 ttccaggcca agggcgctgc gaatacgcgc gatcagctgc acgccgagca gcgaatgccc

1921 gcccagagcg aagaaactgt cgtgacggcc gacgcgctcc acaccgagca ggtcgcgcca

1981 cagcgcggcg agttcggttt cgcatgctcc ttcgggcgcc acataccccc tcggggcgat

2041 cgcatcggcg tccggcgccg gcagtgcgcg gcgatcgagc ttgccgttgg cggtgagcgg

2101 cagcgcctcc agcggcacat aggccgcggg cagcatgtag tcgggcaggc gtgcaccgag

2161 ctgagtgcgc catgcctcgg tatcgggaac gacatcggtg ccgccgacca cgtaggcgac

2221 cagccgcttg ttgcctggcg tgtcctcgcg ggcgatgacc gccgcctgtc gcacgcctgc

2281 gcaggcgcgc aaggcggcgg cgatttcgct caattcgatg cggaagccgc ggatcttgac

2341 ttgttcgtcg ttgcggccga ggtactccag cacgccgtcg gcgcgccagc gggccagatc

2401 gccggtgcgg tacatgcgct ggccgggttg cccggcgaag ggatcgggca cgaagcgctc

2461 ggcggtcagg tcgggacggc cgaggtagcc gcgcgccaat tgcggaccag cgatatatat

2521 ctcgccggcg acgccctgcg gaacccattg gtgagacgcg tcgagtaccc acaggcgggt

2581 attggccagc ggacggccca gcggcacgac gctgccgggt tcgtcgccaa gcaccgggtg

2641 ccgactggtc cagatggtgg cctcggtcgg tccgtacaga ttgcacagct ggccgccgcc

2701 ggcacgcagg cgctgcgcca actccgggcg cagggcttca ccgccgcaca gcaggcgcag

2761 gtccggacgg ctctgccagc cggtgtccag cagcatctgc cagaacgcgg gggtggcctg

2821 cagcacggaa atggcgtgcc ggtccaccag ctgcgaccag tcggccggct cctggatggc

2881 ctggtcgccg gcgatgacca cgcgcgcgcc atggaccaag ggggcgaaca gttccaggcc

2941 ggcgatatcg aagcagacgg tggtgaccgc cagtacacgg tcttcgggcg agagcggcag

3001 gtcgatctgc agcgcggcca ggaactgggt cagcgcgtga tgggaaatca tcacgccctt

3061 gggctgtccg ctggagccgg aggtgtagat gacgtaggcc agatggtgcg ggctgaggtc

3121 ctgcgtcgcc gggggatacg cgggatgcat cgcccagctg gcggtgtcgt ctatccagac

3181 gcgcgcaagc tggttgtccg gcaacagatc ggtcagcgcg gtgtgggtga gcaggcaacg

3241 cgctgcgctg tctctgagca tgaagcccag acgtgcaggc ggatagatcg ggtccagcgg

3301 cacataggcg ccgccggcct tgagcaccgc cagcaaggcg acgaccagat cgacgccgcg

3361 cggcaggcag accgcgacac gcacgtccgg agcaacgccc agggcgatca gatgatgggc

3421 aagctggttg gcacgcgcgt cgagatcggc gtagctcagt gcgagcgcat cgtcgaccag

3481 cgcgatggcc tgcggtgtgc gttgcgcctg tgcctggatc tggtggtgca gacagtcgct

3541 gtcgccgaga tcgatcgtgc tggcattgaa gccttgcaac tgggcgcatt ggtccgcaga

3601 cagcagcggc agttggctta gcggcgcgtg cggcgcttcc tgcagggcca gcagcatgtg

3661 ctgaagcgcg gccaggatgc gctcggccag cggcagcggc acgcgctgcg ggtcgatgtt

3721 gaacagtaca gtggtgggac aggcggcgtt gtagcgccag accacaatgc ccagcgcagc

3781 gtcctcgtag tcgccgtgca acggcatcat gctgcgcacg ccgccctcga tcggcacatc

3841 gccgaaggcg ctgaagtctt cgaccgagac gctgatgtcg aatggccgcg gcgccagctg

3901 cccggtttcg cccagctcgc gcagcgcatg gcgcaggtgc aggcgcgcat gacgcatggc

3961 cggacgcagc gcgctgctca cgccgcgcat ggccgcggcg agatcggcgt gcggctgcac

4021 ggcaagatag agcggcagtt gcgtagacaa catgccgaac atgtcgcgtt cgatgcgggt

4081 gtggcgattg tggacggtca ggccgagcgc gagcggcgcg cggtggtggt gattcaggcg

4141 cgccaggcag ctggccacac aggcgacgat cagtgtggtg accgagccac ccagacgctc

4201 ggccagcgca gtcaaggccg gcaagaggtc cggctgcaac gtaaaactgc gttgcacgac

4261 cggctggcgg ccgagtgcgg cgctagtgct gggatacagc ggctccggcc gtgcacgcag

4321 ggcctggcgc caataatcca ggtcgcgttg gtaacgcgtg gagtcgatat acgccagatc

4381 ggcatcgagc gcttgcaggt aggagggggc cggcggttgg tcgggctcgc cgcgcagacg

4441 ctggttgtag caatcgatca cctgctggga cagcattccc agcgagacgc catcgatgac

4501 gatgtggtgg cacaggtaca ggcagtagcc gcgggtagcg gtggcctgca gccactggat

4561 gtcccacagc ggtttcccat agtgcgcaaa cgagcgcttc atcgcttgct ggatgtgctc

4621 gtgcacgcgc tgttcgccat cggtatcggc cgagtagtcg tgccagggca gtgcgaaggg

4681 cagggtctcg agcacgcgtt ggttcggcaa gggcgcgcct tcgaccagca gcatgcgcag

4741 cgcgtcgtgg cgagcgatca ctgccgcgac ggcctgttcc cagacagcgc ggtgcagggt

4801 gccatcgaag gtcatcacac agccgatggt gtagctgggc tggtccgggg cgaacagttg

4861 accgagccag atgtcttgct gtgccgaact gagcggatgc acggtgtcgc caaccgccgc

4921 agcgcgatcg aaaggcgtca tgcaatcacc gtcgtgcccg gagacagggg cgtagaggcc

4981 gtggctggac gcgcgtggtc gcccacgctg ctcatccacc actgtgccag gagttggctg

5041 tgcggcttgg cgagcacggt catgtggttg ccgggcccgt gccagggacg caggtcggcg

5101 gcatgggcgg cccaggcctg acgtaggcgc aggcgttcgg cggcttgttg ctcgggctgc

5161 agccgggtgt cggcgaccag gaccagctgc aaggtgcctg ggtagggcct ggcggggatg

5221 tagcaggtgc ggcagcaatg cgcgaaggtc gccagactgc cgcgcaccga gtcgggccgc

5281 gagcgtggcg gcatcagacc gtgctcgacc atgaggcgat gcaaggcctg cagcagcgcg

5341 ttctggccga gtcggtgcag cgcctggcga tcgatgccca atggcgcatg cagacgcagc

5401 tgcaaggcgt ccaggaagta gtccaagatg ttgtcgcggc tgaaatcgtg cgggacgcgg

5461 gcccgattcg gtgggcgggt gtcgatcaag gtcaggctgg cggcggggcg gccgagcgca

5521 tgcagccgca gcgccatctc gtagacgacc cagccgccga aggaatggcc gaccagatgc

5581 agttgcggtg ccccggccgt gactgcgggc aaggcatcga gatactgctg cgcagccagt

5641 tcgaccgagc cgaatggcaa ctgacagcca tcggtgccac gcggctgcat gccgatgacg

5701 ttgcaggcat cgccgagcgc actgctcagg tcgacaaagc tggtcacgct gtcgcccgca

5761 ccgggaacgc agacgacgac cggggcatgc gcatgcgacg actgcagcgc gaaggccggt

5821 tcgtcgcggt gccggacgac gtcggcgctg ctggaagcga tgattgtcga aacgatctgg

5881 ccaaggcgcg gcagctgttc ggcttcgatc aacgagacat gagtaccggg cacggcaatc

5941 gaccgtatct ggcccgccgg caagaggcgc gtccaaccca gcgtcacgtc gccgggcaat

6001 ggctcggagg catagaacac ggtgagtggg cactgcggcg ccagtgcctg tacccaatag

6061 gtgggccaca tgcgttcgaa ggtcgcggtc atgcggctgg tcgcgatgac cttggccaag

6121 ctggtctgtt cctgcgcggc cagcgtgttc agggccgcat cctcggacgc ctgctcgacc

6181 agctcctgca gcgatgcctg ttcggcgttc gcaagcagat cgcgcaatgc ggcattgatg

6241 gcgtcatcgc gatcggtgcc gtgttcgagc atggtctgca cccgggtgag cagacgctgc

6301 ttgaccgcct cgtccagtgg caggggatcc ggtgcgcggt cggggcaatc gcagtcgagc

6361 aggcccagga aggcgacggg ctggtcatgc atgccgaagt gttgggcgat ggcataggcg

6421 agcaggccgc ctgaggagta gccgagcagg tgatacggcc cctgcggttg caccacctga

6481 atcatcccga ccatgtgcgc ggccagcgcc tccagcgtgg ccggtagtgg atcgggccag

6541 ggcagcgcgt agaccggaat ctccgcagcg agatgggccg ccagctcgaa tgcatacgcg

6601 acatcggcga tgccggtcgg caccacgaat accggtgggc gcgtcccttg acggcgcgca

6661 gccagggcgc gggttcccca gcgatgcgcg ccgccgagca ggcaatcggc ctgcgcgcgc

6721 aaggtggggt gcgcatacaa cagacgcagc gtcagtggca ggccgacacg atgggcggac

6781 gtcatcagtc gcatggctga gagcgagtgc ccaccgcgtt cgaagaagtg atcgtcgcgg

6841 ccgatgcgct gcggccccag cacgctggcc cagagcttgg cgaggctgcg ttcgatttca

6901 ttggctggcg gtacaaacgc gtcacacacg atggccgtgg cgtccggtgc aggcaacgcg

6961 cggcgatcga gcttgccatt gacgctcacg ggcagcgcat ccaggtgcac gtaggcgctg

7021 ggcagcatga cgtcgggcag gcatgcggtc agttgggtgc gcagtgcgtc agccgtcagg

7081 tgctccgcgt caccgaccag ataggctacc aggcgcggtt cgccgatgtc gtcgcggacg

7141 atcgcgacgg cttcgcgcac gccctcgcag ccgcgcaggg cggtttcgat ctcgccaagt

7201 tcgatgcgga caccgcgcaa cttgatctgc gcgtcgttgc ggccgagata ctccagcgcg

7261 ccatcgacgc gccagcgcgc cagatcgccg gtgcggtaca tgcgctgacc tggtcgatcg

7321 gcaaacggat cggggacgaa gcgttcggcg gtgaggtcgg gccggcccag atagccacgc

7381 gccagctgca cgccggcgat ctgcagttcg ccgggcacgc cgatcggggc gcgtgcgcgc

7441 tgcgcatcga gcacgtgcag gcgggtgttg gcgatcggcc ggccgatcgg cacgctgctg

7501 gcatcggcat cgctgcattc ccagacactc acttccaccg acgcttcggt cgggccgtac

7561 aggttgtaga ggcgtgcccg tggcaggcgc gtgcgtaccg cttgggccag gtcggcggtc

7621 agggcttcgc cgctgcacac gatgcgttgc aggctggtgc atgcgcctgc tggcaagacg

7681 tccaggaaca cgcgcaacat cgagggcacg aagtgcacgg tgtcgatacc ggtgtgctcg

7741 atcagggcaa tgagataggc cgggtccttg tggccctcgt gtcgggccag taccaggcgc

7801 gcgccgacgc gcagcggcca gaacagctcc cagacgcata cgtcgaaacc gaccggggtt

7861 ttctgcagaa cggtctgcgt gggcagtagc tccagcgctt gttgtcccca tgtcaggcga

7921 ttgtcgatgc cggcatgggt ggtgatcacg cccttgggtt gaccggtgga gccggaggtg

7981 tagagcacgt aggcagggtg ttgcgggtgc aagatcgcaa cgacaggcgc gtgcgtggct

8041 gcgttattcc atcgcgcctg atcggtatcc agcagtaccg tgtgcagacc atcgctgagt

8101 gccagcggcg atgcggtagg gcggcgggcg agcagcacgc gcggccgtgc gtcggcaagc

8161 atgcgttcca ggcgattgga gggtgcgtcc ggatccagcg gcatgaacgc agcgccggcc

8221 ttgagcgcag ccagcaatgc gatgaccaga tcgataccgc gcggcaggca caccgccacg

8281 cggtcttcag gcaccacacc aagagcgatc agatgatggg cgagttggtt ggcgcggtta

8341 tccagcgcgg catagctgag ttccacgtca tcttcgacca gcgcgacagc ctgcggcgta

8401 cgttgcgcct gcaatgcgat ggcgcgatgc agatagccgc tgccgccgag atctgcagtc

8461 gtgtcattga agtgtcgcag ctgggcgcgc tcgttttctg gcagcagatc caatgcatgc

8521 agtgcggtct gcggtgcgtg ctgcaacgcc tggaccagcc cttgcagggt ctgcaggagc

8581 atcgccgcga tacgttcggt accgatctgc tgcacgcact gcacgtccaa cgaaaatcca

8641 ccgctggtct gttcgtcgtt gaccgatacc agcaacgggt agtgcgtgcg ctcctgaccg

8701 gcgatctcat gtaccccgtc cagcgcggcg acgtgttcaa cctctggctc cacgcccagg

8761 accgcactgc cgccggaata gcggtagttg agcaaggcat tgagcagcgg agtggagggg

8821 tccaacgtgc cgcaacgctg cgccaatgcc agcggcgcat gttcgtggtg cagcagttgg

8881 gcaaggcaga gttgcgtctg gcgtaccgca tccagcacgc tgccgtgcgc cgtgttgagg

8941 cggatcggca aggtgttgag aaacaggccg agcacacggt ccacgccggc gctggcatgc

9001 atgcgtccaa agagcagggt ggcaaacacc gcttcagtac tgccgctgct acgggccagc

9061 agcaaggcat aggccagatg gaacaggctg gcggcactga cgccatgttg gcgtgcctga

9121 gtgcgcacag cggtggccag cgttgcaggc agcgacacac gtagaaagcg tagcgcggcc

9181 gggtcgcgca ccggtgcaag cacgccgaac ggtgcggtgg gcgcatcgat atcggcgagc

9241 atcgcgctga aaaacgcctt gtgctcctgc tcggacacgt cggcgtgcgc atgagcgacg

9301 aagtcgcgga acggcaatgg cgtcggtaat tgctgttgtc gtccggccag atgggcgcgc

9361 acttcctcga tcaccagttc cagcgtggtg tggtccatga ccaggtgatg ttgttgcagt

9421 ccgagcagcc agcgtccagt cgtggcgtcg tgggccagat gggcgtggat cagcggagca

9481 tgctgcaggc tcggtgctgc ttcgggggtg tgcagccagg tcagtagctc ggcagcaggg

9541 tctggtccat cgaagcgatg cgcacgccgt ggcaggatcg cctcgcgcca gaccacctgc

9601 accggtacgg acagtctctg ccagaggaat ccggtgcgca ggatgtcgtg gcgggcgatg

9661 acctgatcga gtgcgtcgag gaagcggtcg agttgttctt tggaggggaa gccgagcacg

9721 gacgagtgca gatatggatc ggccagcggg tcggacagat gatggaatag caagccttcc

9781 tgcaatggcg ccagcgggta gatgtcctgc acgttggctg cgccgccgtc gacgctggac

9841 acaatcgcgt cgatctccgc ttgcgtcagc gagatcagcg gcagtacgtc cggggtgatg

9901 tctgtgcacg cgggcgcgat ccgattgggt ggcaccacga tggcggaggc cgtttgcaga

9961 ttgacggcca ggcccgcgag cgttggctgg gcgaacaatg cccgcacatc gatttgccag

10021 ccgagccgac gcaggcgttc gatcagtttg atcgccagca gcgagtggcc gccgagggcg

10081 aagaagtcgt cgtggcgtcc gacctgttcg acgccgagca actcgctcca tagtgtggcc

10141 agcacgtgtt cgaggtcgcc actgggcgcg acataggcgt gcagatccag cgcgtgatcg

10201 tcgggggccg gcaaggcggc gcgatcgagt ttgccgttcg gcgttagcgg caatgccgca

10261 agctgcacgt aagcggctgg cagcatgtgg tcgggcaagc gcgtcgccag ttggctgcgc

10321 aacgttgccg gttccagcat cgatgcatcg cctgccagat aggcaaccag gcgtttctgg

10381 ccggtgtcgt cgcgtgcgac aaccaccgcc tcgcgcacgc cgtcgcaatt gcgcaaggcc

10441 gcttcgatct cgcctggctc gatgcggaag ccgcggatct tgacctgcgc atcgttgcgg

10501 ccgagaaagt tcaacgtgcc gtcggcgcgc caacaagcca ggtcgccggt cttgtacatg

10561 cgctcaccgg gttgcgcggc gaacggatcg ggcacgaagc gttcggcggt cagtgcgggg

10621 cggtgcagat agccctgtgc caattgcgca ccggcgatat gcagttcgcc ggccacgcca

10681 atcggcgtgg gttggccgtg gcgatcgagc acgtaaacgc ggctgcccgg cagcggccgg

10741 ccgagcggaa tccgcgcggc gtcgtcggca actgtcgcga ctgcgtgtac cacgctgaaa

10801 gtggtcgctt cggtggggcc atagcaatgc agcagccgtt gcggtggcgc ttgggccagg

10861 agcgcggcga cgctgccggc atcgacgctg tcgccgccgg tgagcagaca acgcagcgtc

10921 ggcagcaggg tgcgcaacgc gggccagtag ccgggcagca gcccggcggt cagatgcagg

10981 atgctgacgt cgtgggctgt gaggtgctgc gccagtgcga tcgggtcgag cagggttggt

11041 tggtcgacga cgacgatggc cgcgccgtgc agcagcgtgg cccacacttc gaaggtgctg

11101 gcatcgaacg ccggattggc caggaaggcg acgcggtcct gcggctgcaa cggttcatgt

11161 tgctggttga gcgcgaacgc cagcacggcc gcctgcgaca cggccacgcc tttgggagtg

11221 ccgctggagc cggaggtgta gacgacatag gcgatgcttg cggatgtcag ctggacgtgt

11281 ggtgccactg cctcgccagt gagggctgcc cattccactt ccagacgtgg aaggcagacg

11341 gcatcgagcg ggcaatcggt gtcgcacagc agcgcggcgg cggcgcagtc gtccagcagt

11401 gcgcgcaagc gtgccggcgg ctgtgccgga tccagggcga cataggcagc gccgcacatc

11461 aacactgcca gctcggccac gatcaacagc gccgagcgtg gcagcagcag cgctacacag

11521 gcgcccggcg tcacccccga ggcgagcagc cgttgggcca gaacggcggc ccgcgcctgc

11581 agctcggcgt aactcacgac cagtttgccg tcgatcagtg ccgctgcccg tggggcacgc

11641 tggacctgtg cgtgcagtgc ccgggtaagc gtcgcgaccg gcctcaggat gggctcgtga

11701 gcgttgaagg tgcgcagcag ttgatcgcat tggtcggcgg gaagccaggg caactgggcc

11761 acacggacgc tgtcctgcgt gctcatgcct ttgagcagat gcatgaagca ggcgaggacg

11821 cgttggatgg tgctgcgttc gaacaatgca gtggcatagc cgatgctgcc gacaatgcag

11881 ctgtgccgtt cttcgagtgt cagctcaaga tcgaacttgc gcgccgccag catctgcggc

11941 agcggctgca gcgccaatcc gggcagggcg atgtcggaga cgggcgtgtt ttgccacgca

12001 aacatgacct ggaacaccgg atggtgggcc aggctgcgca cagggttgag cgcttcgatc

12061 acctgttcga agggcagatc ctgatgatcc tgcgcagcca gcgcggtggc gcggacctgg

12121 gccagcagct cggcgaccga gggattgccg cgcaagtcga tgtgcagggc ctgggtgttg

12181 gcgaacaggc cgatcagcgg ttcaacctcg ctgcgatagc gattagcgat tggagtgccg

12241 atgaccacct ggtgctgccc ggacaagcgc gccagcagca cgccccaggc cgcgagcatg

12301 gtcatgaaca cggtggtgcc gtggcgctgg ctcagggctt tcaacgccgc actcagttcc

12361 gcatccaggg caatttcgat cgcatcgccg ccgtggtccg gcagtgccgg acgtacgcga

12421 tcggtcggta gttgcactac cctcggtgcg tcgtgcaggt gctcgaccca gaactcgcgc

12481 tggcgttgca acacctcgcc gctgatccag cggcgctgcc atatgacgat atcggcgtat

12541 tgcagcgaca gcggcggcaa gggatcgggc aggccgtgcg cgaaggcggc gtacagcgcg

12601 ctcagttcgc gtaccagcag actcatcgac cagccatcgg agatcagatg atgcagggtg

12661 atcaagagca catggtggtc gtcggccaga cgcagcaaac ggccgcgtgc caacgtgtcg

12721 caggccggat cgaagcccgt cagcgtttct tcttcggcgt ggtgtcggac ctgtgcgtcc

12781 ggatcgggat aagcgctgag atcgtgttcg gaaagccgca agccgacgtt gtcggcgtcg

12841 atgatctgca ccggctcgtc gttgtgcagg gcgatacgtg tgcgcaaggt ttcgtggcgg

12901 gcgacgatgc ggtccaacgc ccggcgtagc gcatcgcgat ccagaccgcc gcgcaggtgt

12961 aggccgttgg gcatcaagta ggccaggtcg gcttgcgcgt cgaactgggc caggaaccat

13021 aggcgctgct gggcgaagga cagcggcagt ggttgggcgc ggtcgatggg cacgatggtt

13081 ggcagggtgc tggcggcggc gctgctcaag gcctgagcca gttcggtcag gcgtggatgg

13141 gcgaacaatg tgcccaaggg cagttccagg ccgagcgagg tgcggatgcg cgagatcagc

13201 cgcactgcca gcaacgagtg tccgcccagt gcgaagaagt cgtcgtggcg accgatgcgc

13261 tcgatgccga tcaactcgct ccacaacttg gccagcacgg tttccagctc gccttccggt

13321 gcaacatggg cctgcgttgc gagtgcatcg gcgtctggcg ccggcaatgc gtggcgatcg

13381 agcttgccgt tggtggtgag cg

LOCUS AFHK01000166 9711 bp DNA linear BCT 09-MAY-2011

DEFINITION Xanthomonas oryzae X11-5A Xo_X11-5A_contig_166, whole genome

shotgun sequence.

ACCESSION AFHK01000166 [AFHK01000000](http://www.ncbi.nlm.nih.gov/sites/entrez?db=Nucleotide&cmd=Search&term=AFHK01000000)

VERSION AFHK01000166.1 GI:332357270

DBLINK Project: [66097](http://www.ncbi.nlm.nih.gov/bioproject/66097)

KEYWORDS WGS.

SOURCE Xanthomonas oryzae X11-5A

ORGANISM [Xanthomonas oryzae X11-5A](http://www.ncbi.nlm.nih.gov/Taxonomy/Browser/wwwtax.cgi?id=1009853)

Bacteria; Proteobacteria; Gammaproteobacteria; Xanthomonadales;

Xanthomonadaceae; Xanthomonas.

REFERENCE 1 (bases 1 to 9711)

AUTHORS Triplett,L.R., Hamilton,J.P., Buell,C.R., Tisserat,N.A.,

Verdier,V., Zink,F. and Leach,J.E.

TITLE Genomic Analysis of Xanthomonas oryzae from US Rice Reveals

Substantial Divergence from Known X. oryzae Pathovars

JOURNAL Appl. Environ. Microbiol. (2011). 77(12):3930-7.

PUBMED [21515727](http://www.ncbi.nlm.nih.gov/pubmed/21515727)

COMMENT Base Quality-A custom perl script was used to clean and remove

reads with low complexity and low quality regions. Low quality

regions were defined as an average quality score of < 20 over a

10bp window along the read or > 2 'N' bases in the read. The Velvet parameters used in the final assembly for X11-5A were a hash length of 31, expected coverage of 19.58x, and a k-mer coverage cutoff of 9.79.

Contact Dr. Jan Leach for source DNA or cultures (Department of

BSPM, Colorado State University, Fort Collins, CO).

##Genome-Assembly-Data-START##

Assembly Method :: Velvet v. 0.7.53

Genome Coverage :: 70x

Sequencing Technology :: Illumina GAIIX

##Genome-Assembly-Data-END##

**Annotation was performed by Royer et al. (current manuscript)**

FEATURES Location/Qualifiers

source 1..9711

/organism="Xanthomonas oryzae X11-5A"

/mol_type="genomic DNA"

/strain="X11-5A"

/host="Oryza sativa cv. Lemont"

/db_xref="taxon:[1009853](http://www.ncbi.nlm.nih.gov/Taxonomy/Browser/wwwtax.cgi?id=1009853)"

/country="USA: Texas"

gene (<3..9710>)

/gene="NRPS"

CDS (<3..9710>)

/gene="NRPS"

/note= incomplete NRPS (no start codon, no stop codon)

/codon_start=1

/translation="GCEGVREAVVIARQNAEEKRLIAYLVANATPTDGADVPTADALRMQLAACLPEVMLPSAYVWLDALPLTVNGKLDRRALPAPHTDALAAQAYVAPKGEQEALLATVWSELLGVERIGRHDSFFALGGHSLLAMRLISRIRNLLGVELPLTTLFAQPCLADLADALDGAAAIALPTIMPADRRKPLPLSFAQQRLWFIAQLDTRANPAFHIPVGLRLQGALAADVLQQALDRIVARHEALRTRFVAADGGAIQQIAPADSGFALRHFDLSPHADAETEILAHAQHEAGEAFDLSHGPLARGRLLRLGDDAHVLFLTLHHLVADGWSIGVLVREFVALYTAMMDGQPDPLPPLPLQYADVAVWQRHTLGEHALQRQRRFWHDHLAGAPELLELPTDRPRPALQDYRGDALTFQVDQPTSIALKALAERHGTTLYITLLAGWAILLARLSCQQEVVIGSPVANRNRSELEPLIGLFLNTQALRIDLSADPSVAALLARVRATALAAQEHQDLPFEQVIEALNPSRSMAHAPLYQVVLAMQNTPQEELTLPGLHITALPTGQVSAQVDLWWSISETDAGLRGSVIYASTLFDRATVQRWTQMWIALLQAMTAQPASRVSALPLLPEDHRTRLLQQFNRSTAPWPDAPLLQPLFNAQCRLTPDAPALSDAQVQLSYAQLDARANRLAHRLIAAGVRPDTRVALYLPRCAERLVALLAVFKAGAAYVPLDPDQPTERVAFMLDDARVRVVLTDTQLQQQLPASRALQQTRVLLLDVPTETADPAHDRAPVIDGLHPDHLAYVVYTSGSSGQPKGVMVSHRGLVNLALAQIAAFDVKPHSRVLQLASIGFDACVSELLMAWLAGACLHVPPADALAGSALLAVLQQQRITHLTVTPTVLASLPEQAHSPGLQTLVLAGEAADAEVARRWQAHTRVLNAYGPTEASVCASVHLDGVQDGERLPIGRPLANVRLYVLDPHAHPVPIGVRGQLHIAGHHLARGYLRRPDLTAERFVPDPFAERPGQRMYKTGDLARWNADGNLEYLGRNDDQIKLRGVRIELGDIESALRSCAGVRDAVVLLRHDQISEPRLVAYVVGDAEHLAADALRMQLTTRLPEVMLPAAYVPLDALPLTPNGKRDRRALPAPDADALATQTYLAPQGELETLLATLWSELLGVEHVGRHASFFALGGHSLLAIKLIERLRQHGWALQVRALFGATTLADLATTLRPSGAVEVPPNRIAADCTRITPALLPLVQLTQDEIDAAVATVEGGVANVQDIYPLTALQEGLLFHHRASAVGDAYLSFSVLAFETRAQLDAFVAALEAVIARHDILRTGFAWQGLSTPVQIVWRHALLPRQEHRIDASDVLAALKRRMDPSSARMDLTQAPLLRGHLVEDPHQARWLLGLQTHHLVIDHTTLELLVEEIQLYLGDRAEQLPIALPFRDFVAQARLGASHDQHRAFFTRQLGDLDTPTTPFGLREVHGNGADIEQAVLPLTVSLCAAARVQTRQLDVSPASLFHLAYALVLAQASGQDDVVFGTTLFGRMHAGAGADRALGMFLNTLPIRLQRDGSSVADAVRHTQARLAELLHHEHASLALAQRCSGIAPPAPLFTALFNYRHTGGGSVQAPSIETPHDQWRGVDMLEAQERTNYPLTLSVDDISDDGGFALVVKVNQRIGAARVAAMMLQAVQALVQALEQAPQTALHSLDLLPPDERARLDGFTSTAAAPHQTDCVHRLFQAQVRRTPHAIALLADGNALSYAALEAHANQLAHQLRRLGVGPEHRVALHLPRCLVLVVALLATLKAGAAYLPLDPDLPDARLAFLLEDSGACMVLTCAELHDRLPAADTMPDIGVLRLDALPMPCAADPGAPGVPGLCPDNLAYVIYTSGSTGQPKGTLLTHRAAAHYLQWAAATYRPHPSALVSSSLAFDATLTSLMAPLLCGATVELLPEHDTLEALRHRLCDATPLGLVKLTPAHLAVLGQQLADHPQPLSPAVMVIGGEALPVATLARWQTLAPQTRLINQYGPTEAAIACVMHEATAADAVSSNDRVSIGRPIAHMRVYLLDQRDRRVPIGIAGHLLIAGIQLARGYLGRPDLTAERFVPDPFAEQPGQRMYRSGDLACWRDDGSLDFLGRNDDQVKLRGFRIALGEIAAALRACNGVQDAAVLLREDTPGEPRLVAYVVGDAGHLAAETLRTQLAVYLPEYMLPTAYVTRDALPLTANGKLDRRALPVPDADALAAQAHAPPHGELETLLAALWRELLGVERVGRHDDFFALGGHSLLAVRLISRIRSRLGLELPLAALFAQPRLAGLADALTNAAASTLPAIVPADRSAPLPLSFAQQRLWFLAQLDAQADLAYLMPSALRLRGHLDRDALRQALNRIVARHETLRTRIALHHDEPVQRIDADSVGFSLSEHDLSRSPDQEAHLQRHVEEETLTAFDLAHDSLARGQLLRLADDHHVLLVTLHHLVSDGWSVALLVRELSTLYAAFAQGMPDPLPPLALQYADIAVWQRRWITGEILQRQRDFWVAHLQGAPTQLDQPTDRPRPALQDARGDTLAFALEAPLTAALKTLGQRHGTTLFMTLLAAWGVLLARLSGQDRVVIGTPVANRHRSEVEPLIGLFVNTQALCVDLRADPSVTELLEQVRTTALAAQHHQDLPFEQVIEALNPPRNLAHPPVFQVMFTWHNTPERSIDLPGLVTEGIAAALPTIKFDLDLSLHEVDDRIAGSLAYATALFDRRTIERHLAQFAQVLAGMAADDAATVAQLPLLPPDERAQLQRFSVTEAAPCAPATCIHHLFEAQARRTPHAIALSEGARALRYAELEARANRLAHRLRSVGVGLESRVALYLPRSIDQIVALLATLKAGAAYLPLDPELPDARLAFLLADSGPSAVLTCAALHAQRQWPGTAMRDVNVLMLDTALEAAAGLHDPGAPTVPGLCPDNLAYLIYTSGSTGQPKGTLLTHAGATHYLQWAIDTYRPFPNAVVSSSLAFDATLTSLLAPLLCGAQVELLPEHDTLDALRQRLCDSTPLGLVKLTPAHLHVLGQQLADHQEPLSPAVMVIGGEALPAATLARWQALAPNTRLINEYGPTETVVGCVVHEASGADAHAAYGRVRIGRPIDHLRIHVLDQRAQLVPIGVAGQLHVAGPQLARGYLDRPDLTAERFVPDPFAEQPGQRMYRSGDLACWHADGTLDYLGRNDEQIKLRGFRIELGEITAALRACNGVQDAAVVLREDTPGQP"

ORIGIN

1 gcggctgcga gggcgtgcgc gaagccgtcg tgattgcccg ccaaaacgca gaagaaaagc

61 gtctgatcgc ctacctggtg gctaacgcaa cgccgacgga cggcgctgat gttcccactg

121 ccgacgcatt gcgcatgcaa ctggccgcgt gcctgcccga ggtcatgcta cccagcgcct

181 atgtctggct ggacgcgttg ccgctgaccg tcaacggcaa gctcgatcgc cgcgcattgc

241 cggcgccgca tacggatgca ctcgcagcgc aggcctacgt tgcaccgaaa ggcgagcagg

301 aagccttact ggccactgtg tggagcgaat tgctcggcgt cgaacgcatc ggtcgccacg

361 acagcttctt tgcgctgggt gggcattcgc tgctagccat gcgactgatc tcgcgcatcc

421 gcaacctgct cggtgtcgaa ctgccgctga ccacgttgtt cgcgcaacca tgcctggccg

481 atcttgccga tgctctggac ggggccgccg ccatcgccct gcccaccatc atgccggccg

541 accgtcgcaa gccgctgccg ctgtccttcg cccagcaacg actgtggttc atcgcgcaat

601 tggacacgcg cgccaatccg gccttccaca ttccggtcgg cctgcgcctg cagggtgcgc

661 ttgcggccga tgtcctgcag caggcgctgg atcggatcgt ggcccggcat gaagcgctgc

721 gcacgcgctt cgtcgcggcc gacggcggag cgatacagca gatcgcaccg gccgacagcg

781 gctttgcact gcgccatttc gatctgtcgc cacatgccga tgccgaaacc gagatcctgg

841 cgcacgcgca acacgaagcc ggcgaggcct tcgatctgag ccatggcccg ctcgcacgcg

901 gacggttgtt gcggctggga gacgatgcgc atgtactgtt cctgaccctg caccatctgg

961 tcgccgacgg ctggtccatc ggtgtgctgg tgcgcgagtt cgttgcgctg tacaccgcaa

1021 tgatggacgg ccagcccgat ccgctaccgc cgttgccgct gcaatacgcc gatgtggcgg

1081 tctggcagcg gcacacgctc ggcgaacacg ctctgcaacg ccagcgtcgc ttctggcatg

1141 accatctcgc cggtgccccc gaactgctgg aactgcccac cgatcggcca cggccggcgc

1201 tgcaggacta tcgcggcgat gcgctgacct ttcaggtgga ccagcccacc agcatcgcgc

1261 tgaaggcgct ggctgagcgc cacggcacca ccttgtacat caccttgctg gccggttggg

1321 cgatcctgct ggcacgcctg tcgtgtcagc aggaggtcgt cattggttct cctgtggcca

1381 accgcaaccg cagcgaactc gaaccgctga tcggcctgtt cctcaacacc caggcattgc

1441 gcatcgacct gtcggccgat ccgtcggtcg ccgcgctgct ggcgcgggtg cgcgcgactg

1501 cgttggccgc acaggaacat caggacctgc cgttcgaaca ggtcatcgaa gcgctcaatc

1561 ccagccgcag catggcgcat gcaccgcttt accaagtggt cctggcgatg cagaacacgc

1621 cgcaagagga gctgacgtta ccgggactgc acatcaccgc gctgccgacc ggacaggtct

1681 ccgcccaggt cgacctgtgg tggtcgatca gcgaaaccga cgcgggcctg cgcggctcgg

1741 tgatctacgc cagcaccttg ttcgaccgcg ccaccgtgca acgctggacg cagatgtgga

1801 tcgcgttgct gcaggccatg accgcgcagc cggccagcag ggtaagcgcg ttgccgctgc

1861 ttcccgaaga ccaccgcacc cggctattgc agcagttcaa ccgcagcact gcgccatggc

1921 cggacgcacc gctgctgcag ccgctgttca atgcgcagtg ccgccttact cccgatgccc

1981 cggcgctgtc cgatgcgcaa gtgcagctgt cgtacgccca gctcgatgcg cgcgccaacc

2041 gcctggcgca tcgcctgatc gccgcaggcg tgcgcccgga cacgcgtgtg gcgctgtatc

2101 tgccgcgttg tgccgagcgc ctggtcgccc tgctcgccgt gttcaaggcc ggtgctgcct

2161 atgtcccgct cgaccccgac cagcccaccg agcgggtcgc cttcatgctc gacgatgcac

2221 gcgtgcgtgt cgtgctcacc gacacgcagt tgcagcaaca attgcccgcc agtcgcgccc

2281 tgcagcagac ccgtgtgctg ctgctcgatg taccgaccga gacagcggac ccggcccacg

2341 accgcgctcc ggtcatcgac ggtctgcacc cagaccacct ggcttatgtc gtctacacct

2401 ccggctccag cggccagccc aagggcgtca tggtcagcca tcgcggcctg gtcaatctgg

2461 ccttggcgca gatcgccgcc ttcgatgtaa agccgcatag ccgcgtgctg cagctggcca

2521 gcatcggctt cgacgcctgc gtgtccgaac tgctgatggc ctggctggcc ggcgcctgcc

2581 tgcatgtgcc gccagctgat gcgttagccg gatcggcctt gctcgcggtg ttgcagcagc

2641 aacgcatcac ccatctgacc gtgaccccga cggtgctggc ctcgctgccc gaacaggccc

2701 acagccccgg cctgcagact ctggtgctgg cgggcgaagc ggccgatgca gaggtggctc

2761 gccgctggca ggcacacacc cgcgtactca acgcctatgg ccccaccgag gccagcgtct

2821 gcgccagcgt gcatctggat ggtgtccagg acggtgagcg cctgccgatc ggcaggccac

2881 tggccaacgt ccgcctgtat gtgctcgatc cgcacgcaca ccccgtgccg atcggcgtgc

2941 gcggccaatt gcacatcgcc ggacaccacc tggcgcgtgg ctatctgcgg cgtccggacc

3001 tgaccgccga gcgcttcgtg cccgatccgt ttgccgaacg gcccggccaa cgcatgtaca

3061 agaccggcga cctggcgcgg tggaatgccg acggcaacct ggagtatctc ggtcgcaacg

3121 acgaccagat caagctgcgc ggcgtgcgca tcgaactggg cgacatcgaa agtgcgctgc

3181 gcagttgcgc tggtgtacgc gatgcggtgg tgctgttgcg gcacgaccag atcagcgaac

3241 cgcgtctggt cgcgtacgtg gtcggtgacg ccgaacacct ggccgccgac gcactgcgca

3301 tgcaactgac aacacgctta cctgaggtca tgctgccagc ggcctatgtg ccgctggacg

3361 cactaccgct gacgcccaac ggcaagcgcg atcgccgcgc cttgccagca ccggatgccg

3421 atgcactcgc cacgcaaacc taccttgcac cgcagggcga actagaaacc ttgctggcca

3481 cgttgtggag cgaactactc ggcgtcgaac acgtcggccg tcatgccagc ttcttcgcgc

3541 tgggcggtca ctcgctgctg gcgatcaaac tgatcgagcg tctgcgccag catggctggg

3601 cgctgcaagt acgtgcgctg ttcggtgcca ccacgctcgc cgatctggcc accacgctgc

3661 gccccagcgg tgcagtcgag gtgccaccca accgcatcgc cgccgattgc acccgcatca

3721 cgcccgcgct cttgccgctg gtgcagctga cccaggacga gatcgacgcg gcggtagcca

3781 ccgttgaagg tggcgtggcc aacgtgcagg acatctatcc gctgaccgcg ctgcaggaag

3841 gcctgttgtt ccatcaccgt gccagcgcgg tgggcgacgc ctacctgagc ttcagtgtgc

3901 tggccttcga gacccgcgcg cagctggacg cgttcgtcgc tgcgctggaa gcggtcatcg

3961 ctcgccacga catcctgcgc accggtttcg cctggcaggg cctgagcacg cccgtgcaga

4021 tcgtctggcg ccatgccctg ctaccgcgcc aggagcaccg catcgatgcg tccgatgtgc

4081 tggcagcgtt gaagcgacgc atggacccat ccagtgcccg catggatctc acccaggcgc

4141 cactgctgcg tggccatctt gtcgaggatc cgcaccaggc acgctggttg ctgggcctgc

4201 agacccatca cttggtgatc gaccacacca ccctggaatt gctcgtcgag gaaatccagc

4261 tctacctcgg cgaccgcgcc gagcagttgc ctatcgcgtt gccgttccgc gacttcgtgg

4321 cccaggcgcg cctgggcgcg agccacgatc agcaccgagc cttcttcacc cgacaactgg

4381 gcgacctcga tacgcccaca acgccattcg ggctgcggga ggtgcacggc aatggcgcgg

4441 acatcgaaca ggcggtgctt ccactgactg tttcgctatg tgcggctgca cgcgtgcaaa

4501 cccgccagct ggatgtcagc cccgccagcc tgttccacct ggcctatgcg ctggtgctgg

4561 cgcaggccag cggccaggac gatgtggtgt tcggcaccac cttgttcgga cgcatgcacg

4621 ccggtgcagg cgcagaccgt gcgctgggca tgttcctcaa tacgctgccg atccgcctgc

4681 agcgcgacgg cagcagcgtc gccgacgccg tgcgccacac ccaggcgcgg ctggccgaat

4741 tgcttcatca cgaacatgcg tcattggccc tggcccaacg ctgcagcggc atcgctccgc

4801 ctgctccgct gttcaccgca ttgttcaact accgccatac cggtggcggt tcggtgcagg

4861 cgccgtcaat cgaaacccca cacgatcaat ggcgcggcgt cgacatgttg gaggcgcagg

4921 agcgcaccaa ttatccgctg acgctatcgg tcgacgacat cagtgacgat ggcggattcg

4981 cgctggtggt gaaggtcaac cagcgcatcg gcgccgcacg tgtcgcagcc atgatgctgc

5041 aggccgtaca ggcgctggtg caggccctgg agcaagcgcc gcagaccgcc ttgcacagcc

5101 tggacctgct gccgccggac gaacgcgccc gattggacgg ctttacgtcc accgcagccg

5161 cgccacacca gacggactgc gtccatcgac tgttccaagc gcaggtccgc cgcactccgc

5221 atgccatcgc gctactcgcc gacggcaacg cactgagtta tgccgcgctt gaggcccacg

5281 ccaatcagct agcgcaccaa ctccgtcgct tgggcgtcgg gccagaacat cgcgtggcct

5341 tgcatctgcc gcgctgcctg gtgctggtgg tggcgctgtt ggccacgctc aaggccggcg

5401 ccgcctatct gccgctggac ccggatcttc ccgacgcacg cctggccttc ctgctcgagg

5461 acagcggcgc atgcatggtg ctgacctgcg ccgagctgca cgatcggctc cctgccgcag

5521 acaccatgcc cgacatcggc gtgctgcgcc tggacgcgct gccgatgccc tgcgccgccg

5581 atcccggtgc accgggtgtc ccgggcctgt gcccggacaa cctcgcctac gtgatctata

5641 cctccggttc caccggacaa cccaagggca ccctgctcac ccaccgcgcg gctgcccact

5701 acctgcaatg ggcggccgcg acctatcgcc cgcatcccag cgcattggtg tcgtcctcgc

5761 tggccttcga cgccacgctg accagcttga tggcgccgct gctgtgcgga gcaacggtcg

5821 aactactgcc tgagcacgac acgctggagg ccttgcgcca tcgtctgtgc gatgccaccc

5881 cgctgggcct ggtcaagctc actccagccc atctcgcagt gctgggccag caactggcag

5941 atcacccaca accgctgagc cccgcagtga tggtgatcgg tggcgaagcc ctgccggtcg

6001 caacgctcgc acgctggcaa acgctcgcgc cacagacgcg cctcatcaac caatacggcc

6061 ccaccgaagc cgcgattgcc tgcgtgatgc atgaagccac cgccgccgat gcagtgtcca

6121 gcaacgacag ggtctcgatc ggccggccca tcgcgcacat gcgtgtctac ctgctcgatc

6181 agcgcgaccg acgcgttccg atcggcatcg ccgggcatct gctgatcgcc gggatccagc

6241 tggcacgcgg ctacctgggc cgccccgatc tgactgccga gcgcttcgtc cccgatccgt

6301 tcgcagaaca gccaggccag cgcatgtacc gcagcggcga cctggcctgc tggcgcgacg

6361 atggcagcct ggacttcctc ggtcgcaacg acgatcaggt caagctgcgc ggcttccgca

6421 ttgcattggg cgaaatcgcc gccgcgttac gcgcctgcaa tggcgtgcag gacgccgccg

6481 tgctgctgcg tgaggacacg ccgggcgagc cgcgtctggt cgcttatgtg gttggcgatg

6541 ccgggcatct ggcagccgaa acgctgcgca cgcagctggc cgtgtacctg cccgagtaca

6601 tgctgcccac cgcttacgtc acgcgcgatg cgctgccgct gaccgccaac ggcaagctcg

6661 atcgtcgtgc cctgccggtg ccggacgccg acgcgctcgc cgcacaggcc catgcgccgc

6721 cccacggcga gctggagacc ctgctcgccg cgctatggcg cgagctgctc ggcgtcgaac

6781 gcgtcggtcg ccacgacgac tttttcgccc tcggcgggca ctccttgctt gcggtgcgcc

6841 tgatctcgcg catccgcagt cgcctcggcc tggaactgcc cctggccgcg ctgttcgccc

6901 agccacgcct ggccggactg gccgatgccc tgaccaacgc cgccgccagc accttgccgg

6961 ccatcgtgcc ggccgaccgc agcgcgccgc tgccgctgtc cttcgcccaa cagcggctgt

7021 ggttcctcgc ccagctcgac gcccaggccg atctggccta cctgatgccc agcgccctgc

7081 gcctgcgcgg tcacctcgat cgcgatgcgc tgcgccaagc cctgaaccgc atcgtcgccc

7141 gccatgagac cttgcgcacc cgcatcgcac tgcaccacga cgaaccggtg caacgcatcg

7201 atgccgacag tgtcggtttc tcgctgtccg aacacgatct cagtcgctct cccgatcagg

7261 aagcacatct ccaacgccac gtcgaagaag aaacgctgac ggccttcgac ctggcccacg

7321 acagcctggc acgcggccaa ttactgcgcc tggccgacga ccaccatgtc ctgctcgtca

7381 ccttgcacca tctggtctcc gatggctggt cggtggcatt gctggtgcgc gagctgagca

7441 ccctctacgc cgccttcgcc caaggcatgc ccgatccgtt accgccgctg gcgctgcagt

7501 acgccgacat cgccgtgtgg caacgccgat ggatcaccgg cgagatcctg caacgccagc

7561 gcgacttctg ggtcgcgcat ctgcagggcg caccgaccca gttggaccag cccaccgacc

7621 ggccacggcc agcgctgcag gacgcccgcg gcgacacgct ggccttcgca ctggaagcgc

7681 cgctgacggc ggcactgaaa accctcggcc agcggcacgg caccacgctg ttcatgaccc

7741 tgctcgcggc ctggggcgtg ctcctggcgc gcctgtccgg ccaggatcgg gtggtcatcg

7801 gcaccccggt ggccaaccgc catcgtagcg aggtcgagcc gctgatcggt ctgttcgtca

7861 acacccaggc cctgtgcgtc gacctgcgcg ccgatccctc ggtcaccgag ctgctggagc

7921 aggtgcgcac caccgccctg gccgcacagc accaccagga cctgcccttc gagcaggtca

7981 tcgaagccct caacccaccg cgcaacctgg cccacccacc ggtgttccag gtcatgttca

8041 cctggcacaa cacccccgaa cgcagcatcg acctgccggg actcgtcacc gagggcatcg

8101 ccgcggcctt acccacgatc aagttcgatc tggacctgtc cctgcacgag gtcgacgacc

8161 gcatcgccgg cagcttggcg tatgccaccg cactgttcga ccgccgcacc atcgagcgcc

8221 acctggccca gtttgcccag gtgctcgccg gcatggcggc cgacgacgcg gctaccgtcg

8281 cgcagctgcc gctgctgccc cccgacgaac gcgcgcagtt gcaacgcttc agcgtcaccg

8341 aagccgcgcc gtgcgcaccg gccacctgta tccatcacct gttcgaagcg caggcacggc

8401 gcacgcccca tgcgatcgca ctgagcgaag gcgcgcgcgc actgcgctac gccgaactgg

8461 aagcacgcgc caaccggttg gcgcatcgcc tgcgcagcgt gggggtcggc ctggagagcc

8521 gggtcgccct gtacctgccg cgcagcatcg accagatcgt tgcactcctc gccacgctca

8581 aggccggcgc cgcctacctg ccgctggatc cggagctgcc cgacgcacgc ctggcgttcc

8641 tgcttgcaga cagtggtcca agtgcggtgc tgacctgcgc tgcactgcat gcgcagcggc

8701 agtggcctgg cactgcgatg cgagacgtca atgtgctgat gctggacacc gcgctggagg

8761 cggccgcagg tctgcacgac cccggcgcac cgacggttcc cggcctgtgc cccgataacc

8821 tcgcgtacct catctacacc tccggctcca ccgggcagcc caaaggcacg ttgctcacgc

8881 atgccggggc gacgcattac ctgcaatggg ccatcgacac gtatcgccca ttccccaacg

8941 cggtagtgtc ttcttcgctc gccttcgatg cgacgctgac cagcctgctg gcgccgctgc

9001 tgtgcggtgc gcaggtcgaa ctgctgcccg aacacgacac cctcgatgcc ctgcgccaac

9061 gcctgtgcga ttccaccccg ctgggactgg tcaagctcac cccggcgcat ctgcacgtgc

9121 tggggcagca gttggccgac catcaggaac cgctcagtcc cgcggtgatg gtgatcggtg

9181 gcgaagcgct gcccgccgca actctcgcac gctggcaagc gctcgcaccg aatacccgcc

9241 tgatcaacga atacggaccc accgaaaccg tggtcggctg tgtggtgcac gaagcgagcg

9301 gcgcagatgc acatgccgcg tatggccgcg tgcggatcgg ccggccgatc gaccatctgc

9361 gcatccatgt gctcgatcaa cgcgctcagc tggtcccgat cggcgtcgca ggccagttgc

9421 atgtcgccgg accgcagctg gcacgtggct atctcgaccg ccccgatctg accgccgaac

9481 gcttcgtccc cgatccgttc gccgagcagc ccggccaacg catgtaccgc agcggcgatc

9541 tggcctgctg gcatgccgat ggcaccctgg actatctcgg tcgcaacgac gagcagatca

9601 aactgcgcgg tttccgtatc gaactcggcg aaatcaccgc cgcgctgcgc gcctgcaacg

9661 gcgtgcagga cgccgcagtc gtgctgcgcg aagacacgcc cggccagccg c

LOCUS AFHK01000167 9589 bp DNA linear BCT 09-MAY-2011

DEFINITION Xanthomonas oryzae X11-5A Xo_X11-5A_contig_167, whole genome

shotgun sequence.

ACCESSION AFHK01000167 [AFHK01000000](http://www.ncbi.nlm.nih.gov/sites/entrez?db=Nucleotide&cmd=Search&term=AFHK01000000)

VERSION AFHK01000167.1 GI:332357268

DBLINK Project: [66097](http://www.ncbi.nlm.nih.gov/bioproject/66097)

KEYWORDS WGS.

SOURCE Xanthomonas oryzae X11-5A

ORGANISM [Xanthomonas oryzae X11-5A](http://www.ncbi.nlm.nih.gov/Taxonomy/Browser/wwwtax.cgi?id=1009853)

Bacteria; Proteobacteria; Gammaproteobacteria; Xanthomonadales;

Xanthomonadaceae; Xanthomonas.

REFERENCE 1 (bases 1 to 9589)

AUTHORS Triplett,L.R., Hamilton,J.P., Buell,C.R., Tisserat,N.A.,

Verdier,V., Zink,F. and Leach,J.E.

TITLE Genomic Analysis of Xanthomonas oryzae from US Rice Reveals

Substantial Divergence from Known X. oryzae Pathovars

JOURNAL Appl. Environ. Microbiol. (2011). 77(12):3930-7.

PUBMED [21515727](http://www.ncbi.nlm.nih.gov/pubmed/21515727)

COMMENT Base Quality-A custom perl script was used to clean and remove

reads with low complexity and low quality regions. Low quality

regions were defined as an average quality score of < 20 over a

10bp window along the read or > 2 'N' bases in the read. The Velvet parameters used in the final assembly for X11-5A were a hash length of 31, expected coverage of 19.58x, and a k-mer coverage cutoff of 9.79.

Contact Dr. Jan Leach for source DNA or cultures (Department of

BSPM, Colorado State University, Fort Collins, CO).

##Genome-Assembly-Data-START##

Assembly Method :: Velvet v. 0.7.53

Genome Coverage :: 70x

Sequencing Technology :: Illumina GAIIX

##Genome-Assembly-Data-END##

**Annotation was performed by Royer et al. (current manuscript)**

FEATURES Location/Qualifiers

source 1..9589

/organism="Xanthomonas oryzae X11-5A"

/mol_type="genomic DNA"

/strain="X11-5A"

/host="Oryza sativa cv. Lemont"

/db_xref="taxon:[1009853](http://www.ncbi.nlm.nih.gov/Taxonomy/Browser/wwwtax.cgi?id=1009853)"

/country="USA: Texas"

gene complement (<3..9587>)

/gene="NRPS"

CDS complement (<3..9587>)

/gene="NRPS"

/note= incomplete NRPS (no start codon, no stop codon)

/codon_start=1

/translation="MLPSAYVWLESLPLTANGKLDRRALPAPEQSDLGNAAFVPPQSLLEQMLAQLWSELLGVERVGRHDSFFALGGHSLLGVKLIERLRLLGWQLDVRALFSTPTLLGLAEALKTASMVNVPANPIVCDCAHITPELLPLVALDQTEIDSIVERVEGGAGNIQDIYPLAPLQHGLLYHHVAAPLDDIYLSSTVIAFDACDKRERFIAALDQVIARHDILRTAVFWEGLREPVQVVRRHAPLPVQSHRFAGSDVAAHLQQCLETQHRRIDLQQAPLLRAHVVDDAERECWLLGLQYHHLVMDHTTLELVVEEVQAHLDGQQHHLPQPLPFRDFVAQSCLGVSEDEHRAFFTQMLADVTEPTLPFGVSQVEGALQEANLGLPDALADALRIQARRLGVSTASLFHLAFALLLSQISGRDDVVFGTVLFGRMHASAGADRVLGMFLNTLPLRLRRDHTGVEQAVLQTQRLLAQLLHHEHALLAQAQRCSGIQAPASLFAALFNYRYRGGGNVLADDRARSETIWQGIETVHLRERTHYPLSLAINDDHDSGGFSVDVQTGQRLDPATLAQTMVDILQQLAQALTVSPTRPVHALTRLHDRERTQVLQAFNATDAARPAVDGVHQRFEHQARQTPNACAVLDEHRTLSYADLDSQANRMAHRLRALGATPGSSIAVALARSADLIIAELAILKCGAAYVPLDIEHPAQRMRRILDECGARLLISHSASDVPNGSATRVDMDRLDAEGPTHAPSVAVWPDSAAYVMYTSGSTGTPKGVVIPHRAIVNFASQPGAARVLPTDRVAFASNPAFDSSTLEVWSSLLNGAAIVIVPPSVLRDPQALCAQIAQMQISVLILVAGVLRAYAPMLTGQLPTLRLLITGGDLADPHSHALMLQAPGPDVLLQTYGPTEATQFVTALALHSGSDPQPPVPIGRPLANSRVFVLDRFGQPVPIGVEGALHIGGAQLALGYLGRPDLTAATFLPDPFAAQPGQRMYRTGDLARWRADGTLDFLGRRDDQVKLRGFRIELGEIAAAVRACNGVRDAVVVIDQDTDGHKRLVAYVVGAADVCQALPAHLGTSLPDYMVPAAYVPLDALPLTANGKLDRRALPAPDRQDFGTQCFQAPEPGREQALALIWTELLGVAQIGRHDDFFALGGHSLLAVQLIARIRTRLGVEVPVATLFAHPRLAELARAMDSTAAAMLAPIVPVARSGRLPLSFAQQRLWFLGRLDPHADLAYLMPMALRLRGVLQAAALGRALDRIVARHEVLRTYVALDEQTPSQCIASPEIGFALEDVDLTDVSEPQIQIQQYIDLETATPFPAERPLCRGRLLRLADDDHVLLITLHHLVGDGWSMGVLVQELAALYAAFVDERPDPLPPLTLQYADVAAWQRGWISGEVLQRQRDFWIAHLHGAPTLLDLPTDHPRPNQQAYRGDALDVVLDAELTNALKACCQRHGTTLFMTMLAAWAVLLARLSGQEQVVIGTPVANRHRSEFEPLIGLFANTQALHIDLRGNPAFAELLAQIRSTALAAQEHQDLPFEQVIEALNPTRNLAHHPVFQVMFTWQNTPPTDIALPGLVAEGVETVLPTIKFDLDLSLREHAGCIVGNLGYATALFERSTIESHLAQFVTLLRGLVDADRTCVAHLPVLPADQRQWLPHQVHDALPTPSALPYVHRLFEAQVLRTPRHVALQCDAESLDYATLDTRANQLAHHLIELGVGPEDRVAVCLPRGSDLVVAVLATLKAGAAYLPLDPAYPPARLDDMLADARPRVLLAHRDTAAPLAHPDKLHTVLLDAQPTAWTSAPTHAPVVPALHPQHPAYVIYTSGSTGRPKGVVIGHGALSHFLAALHTHLPFSPEDRLLAVTTVCFDIAGLELFAPLLQGARVVIAGDQAMQDPGDWLRLLEQHAISVLQATPAFWQMLLDAGWQSRPGMRLLCGGEALSQTLAQRLRAGGGQLWNLYGPTEATIWASLHPVLGDDPGSVVPLGGPLAGTSLRLLDTYGQLAPLGARGALSIAGPQLARGYLGRPDLTAERFIPDPFADQPGQRMYQTGDLARWRAEGVLEFLGRNDDQVKLRGFRIELGDIAAALRACEGVHDAVVIARHDTPGDMRLVAYMAGDASALTPERLRQHLTLRLPDYMVPAAYVPLDALPLTANGKLDRAALPAPDRQALDAEAYEAPHPGLEERLAVLWAELLGVERVGRNDSFFALGGHSLLATQLSGRLRAALGREVPLATLFAQPRLRGFAQAVAQTTSSALPPIIPVPRTGQLPLSFAQQRLWFLDRLDAGASTAYLMPTSVRLLGALNRDALRRALDRLVARHEALRTHVGFDADTPTQRIASPEIGFCLEIVAAVDAADLQQVIQQHAALEASTPFDLRRGPLLRGRLVCLGTDDHVLFVTLHHLICDGWSMRQLIAELRTLYSAFAKGAADPLPALPIQYADYAAWQRCWITDETLQHQRQFWVDHLQGAPALLDLPTDWPRPATQDYCGDTLKFALDPALSAALRTLSLEHGTTLFMTLLAAWATLLARWSSQPRVVIGTPIANRPRSELEPLIGLFANTQALQLDLSGNPTVAALLAQVRSTALAAQAHQDLPFEQVIEAVNPPRNLSHHPLFQVMLTWDVQHAMDLTWPHLQTQDIDQASTTIKFDLHLALQERADGIGGGLSYATALFDRSTIARQLEQFVTLLRGMVAASRSTVSDLPLLPPSEHAQLQRFNATDADLGGSGYLHRAIEAQARRMPQAVALVQGACELSYAQLDARANQLAHHLIALGVVPEDRVAVCLPRGIDLVVALLAVVKAGGAYLPLDTDVPPPRLDGMLADAQPGVLLAHRDTAALLAHRDALHTVLLDAEPAAWMSSPTHAPVVSALHPQHPAYVLYTSGSTGQPKGVVNTHAGIDNRLQWMQHALQLQPGQRVLQKTPVGFDVSVWELFWPLRVGACLVLAQPGGHKDPGYLIDLIEQTGIDTVHFVPSMLRVFLDAVPQGACPNLQRIVCSGEALPADLAADTRARLPQTQLYNLYGPTEAAVDVSVWECSDADVSSVPIGRPIANTRLHVLDAHRALAPIGVTGELQIAGLQLARGYLGRPDLTAERFVPDPFADQPGQRMYRTGDLARWRMDGAVEYLGRNDEQIKLRGVRIELGEIAAALRACDGVREAVVIARDDASEKRLIAYLVGDAAHLAAEALRTQLAARLPEVMLPSAYVWL"

ORIGIN

1 agccacacat aagcgctggg cagcatgacc tcgggcaggc gcgcggccag ttgcgtgcgc

61 agtgcctcgg ctgccaggtg cgctgcatcg ccaaccagat aggcgatcaa gcgtttctcg

121 cttgcatcgt cgcgggcaat gacgaccgcc tcgcgcacgc catcgcaggc acgcaatgcg

181 gcggcgatct cgcccagttc gatgcgcacg ccgcgcagtt tgatctgctc gtcgttgcgt

241 cccagatact ccactgcgcc atccatgcgc cagcgcgcca gatcgccagt gcggtacatg

301 cgctggccgg gctggtcggc gaacggatcg ggcacgaaac gctcggcggt cagatcgggc

361 cgacccagat agccacgtgc cagttgcagg ccggcgatct gcagttcgcc ggtgacaccg

421 atcggggcga gtgcgcggtg cgcatcgagg acgtgcaggc gggtgttggc gatcggtcgg

481 ccgatcggca cgctgctgac atcggcgtcg ctgcattccc agacgctgac gtccaccgcc

541 gcttcggtcg gtccgtagag gttgtacaac tgcgtctgcg gcaagcgagc acgtgtgtca

601 gctgccagat ctgcaggcaa ggcttcgccg ctgcagacga tgcgttgcag attcgggcat

661 gcgccttgcg gtacggcgtc aaggaacacg cgcagcatcg aaggcacgaa gtgcacggtg

721 tcgatgccgg tctgttcgat caggtcgatc agataccccg gatctttgtg gccgccgggc

781 tgggccagta ccagacacgc acccacgcgc agcggccaga acagctccca caccgagacg

841 tcgaagccga ccggggtttt ttgcaacacg cgctgtcccg gttgcagttg caacgcgtgc

901 tgcatccatt gcagacggtt gtcgatgccg gcgtgggtgt tgaccacgcc cttgggctgg

961 ccggtggagc cggaggtgta gagcacgtag gccggatgtt gcgggtgcaa ggcggacacg

1021 acgggagcat gcgtgggaga cgacatccat gcggcgggct cggcatccaa cagtacggtg

1081 tgtagcgcgt cgcggtgcgc cagcagcgcg gcggtatcgc gatgtgcaag cagaacaccg

1141 ggctgcgcat cggcgagcat gccgtccagg cgcgggggtg gtacatcggt gtccagcggg

1201 agataggcac cgccggcctt gaccacggcg agcagcgcga cgaccagatc gataccgcgt

1261 ggcaagcaca cggccacacg gtcttcgggg accacaccga gggcgatcag atgatgcgca

1321 agttggttgg cacgcgcatc gagctgggcg tagctcaact cgcacgcacc ttgcaccaac

1381 gcgacagcct gcggcatgcg ccgcgcctgc gcttcgatgg cacgatgcag gtagccgctg

1441 ccgccgagat cggcgtcggt ggcgttgaag cgctgcaact gcgcatgctc ggacggcggc

1501 agcagcggaa gatccgaaac cgtggaccga ctggcggcga ccatgccgcg cagcaaggtg

1561 acgaactgct ccagctgacg ggcaatggtg ctgcggtcga acaacgcggt ggcgtagctc

1621 aggccgccgc cgatgccgtc cgccctctcc tgcagcgcca gatgcagatc gaacttgatc

1681 gtggtgctgg cctggtcgat gtcctgggtc tgcaagtgcg gccaggtcag atccatggcg

1741 tgctgcacat cccaggtcag catcacctgg aataacgggt gatggctgag gttgcgcggt

1801 gggttgacgg cctcgatcac ctgctcgaag ggcaggtctt gatgcgcctg tgcggccagg

1861 gcggtactgc gcacctgggc caacagcgcg gcgactgtcg ggttaccgct tagatcgagc

1921 tgcaaggcct gcgtgttggc gaacagaccg atcaatggtt ccagttcgct gcgcgggcga

1981 ttggcgatgg gcgtgccgat gacgacccgc ggttggctgg accagcgcgc cagcagggtt

2041 gcccaggcag ccagcagggt catgaagagc gtcgtgccat gctccaggct cagggtccgc

2101 aacgcagcgc tcaatgcggg atccagcgcg aacttcaggg tgtcgccgca gtaatcctgc

2161 gttgcgggac gcggccagtc tgtcggtaaa tccagcaacg ccggagcgcc ctgcaggtga

2221 tcgacccaga attggcgctg atgctgcagc gtctcatcgg tgatccagca gcgttgccat

2281 gccgcatagt cggcgtattg gatcggcagt gcggggagcg gatcggctgc tcctttcgcg

2341 aatgcgctgt agagcgtgcg cagttccgca atcagttgcc gcatcgacca gccatcgcag

2401 atcagatggt gcagggtgac gaacagcaca tggtcgtccg tgcccaggca gaccaggcgg

2461 ccgcgcagca acggaccacg gcgcagatcg aacggggtgc tcgcctccag cgcggcatgc

2521 tgctggatga cctgctgcag atcggcggca tccaccgctg ccacgatctc caggcagaag

2581 ccgatctccg gcgacgcaat ccgttgtgtg ggcgtgtccg catcgaaccc gacatgcgtg

2641 cgcagcgctt catggcgtgc gacgaggcga tccagtgcgc ggcgcaaggc atcgcgattc

2701 agcgcgccga gcagacgcac cgaggtcggc atcagatatg cggtgctggc gccggcatcg

2761 agccggtcca gaaaccacag gcgttgctgg gcgaaggaca gcggcagttg gccggtacgt

2821 ggcaccggga tgatcggcgg caatgcgctg gaggtggtct gcgcgactgc ctgagcgaat

2881 ccacgcagcc gtggctgggc gaacaatgtc gccagcggca cctcgcgtcc caacgcggcg

2941 cgcagccgcc cgctgagttg cgtggccagc agggaatgtc cgcccagggc gaagaagctg

3001 tcgttgcgtc ccacacgttc gacaccgagc agttccgccc agagcacggc caggcgttct

3061 tccaatccgg ggtgtggcgc ctcgtatgcc tcggcatcca gggcctgacg gtccggcgcc

3121 ggcaacgcag cgcggtcgag tttgccgttg gcggtcagcg gcagggcgtc cagcggcaca

3181 tacgccgccg ggaccatgta gtcgggcagg cgcagggtca agtgctggcg caggcgctcg

3241 ggggtgagcg cgctggcgtc gccggccatg taagcgacca gacgcatgtc gccgggcgtg

3301 tcgtgacggg caatcaccac cgcgtcgtgc acgccctcgc atgcgcgcag tgcggcggcg

3361 atgtctccca gttcgatgcg gaaaccgcgc aacttgacct gatcgtcgtt gcggccgaga

3421 aactccagca cgccctcggc gcgccagcgc gccagatcgc ctgtctggta catgcgctgg

3481 ccgggctggt cggcgaacgg gtcggggatg aagcgctcgg cggtcaggtc cgggcggccc

3541 aggtagccgc gcgccagctg cggaccggcg atggagagcg cgccgcgcgc gcccagcggc

3601 gccaactggc catacgtatc gagcaggcgt agggaggtgc ccgccaacgg cccgccaagc

3661 ggcaccacgc tgccggggtc gtcgccgagc accggatgca ggctggccca gatggtggcc

3721 tcggtcgggc catacaggtt ccacagttgc ccaccgccag cgcgcaggcg ctgcgccagt

3781 gtctggctca aggcttcgcc gccgcacagc agacgcatcc cgggacggct ctgccagccg

3841 gcgtccagca gcatctgcca aaacgccgga gtggcttgca acaccgagat ggcgtgctgt

3901 tccagcaagc gcagccaatc gcccgggtcc tgcatcgcct ggtcgccagc gatgaccacg

3961 cgcgcaccct ggagcaacgg agcgaacagt tccagcccgg cgatatcgaa gcagacggtg

4021 gtgactgcca gcagacggtc ctcgggcgag aacggcaagt gggtgtgcag cgccgccagg

4081 aagtggctca gcgcgccgtg gccgatcacc acgcctttgg gccggccggt ggagccggag

4141 gtgtaaatca cataggccgg atgctgcggg tgcaatgccg gtacgacggg ggcgtgtgta

4201 ggtgccgacg tccatgcggt cggttgggca tccagcagca cggtgtgcag tttgtcggga

4261 tgggcgagcg gcgcggcggt gtcgcgatgg gcaagcagca cgcgcggccg tgcgtcggcg

4321 agcatgtcgt ccaggcgtgc gggcggatag gccgggtcca gcggcagata ggcggcgccg

4381 gccttgagcg tggccagcac ggccaccacc agatcgctgc cgcgcggcag gcacacggcc

4441 acgcggtctt ccggtccgac acccagctcg atcagatggt gagcgagctg attggcacgc

4501 gtgtcgagcg tggcgtaatc aagcgactct gcgtcgcact gcagcgcgac gtgccgcggc

4561 gtgcgcagca cctgcgcttc gaacaggcga tgcacgtacg gcaaggccga cggtgtcggc

4621 agcgcgtcgt gtacctgatg cggcagccac tggcgctggt ctgctggcag gacgggcagg

4681 tgcgccacgc acgtgcggtc ggcatcgacc aggccgcgca acagcgttac gaactgggcc

4741 agatggcttt cgatggtgct gcgttcgaac agcgcggtgg catatcccag gttaccgacg

4801 atgcatccgg catgctcgcg cagcgacagg tccagatcga acttgatcgt cggcagcacg

4861 gtctccacgc cttcggcgac cagccccggc agtgcgatgt cggtgggcgg ggtgttctgc

4921 caggtaaaca tgacctggaa caccggatga tgcgccagat tacgggtcgg attgagcgct

4981 tcgatgactt gctcgaaggg cagatcctgg tgctcctgcg cggccagcgc agtgctgcgg

5041 atctgggcca gcaattcggc gaacgcgggg ttgccgcgca ggtcgatatg cagggcctgg

5101 gtattggcga acaggccgat caacggttcg aactcgctgc ggtggcggtt ggccacgggc

5161 gtgccgatca cgacctgctc ctggccggac aggcgcgcca acagcaccgc ccaggcggcg

5221 agcatggtca tgaatagggt ggtgccgtga cgctggcaac acgccttcaa ggcgttggtg

5281 agctccgcat ccagcacaac atccagcgca tcgccgcgat aggcctgctg gtttgggcgt

5341 ggatggtcgg tgggcagatc cagcagcgtc ggcgcgccgt gcaggtgcgc gatccagaag

5401 tcgcgctggc gttgcaacac ctcgccactg atccagccac gctgccaggc ggcgacatcg

5461 gcgtattgca gggtgagcgg tggcagcgga tcgggacgtt cgtcgacgaa tgcggcatat

5521 agcgcggcca gttcctggac cagcacgccc atcgaccagc catcgccgac cagatggtgc

5581 agggtgatga gcagcacatg atcgtcgtcg gccaggcgca gcaggcggcc acggcacagc

5641 ggtcgttcgg ccgggaaggg cgtcgcggtt tccagatcga tgtactgctg gatctggatc

5701 tgtggctccg acacgtcggt caggtccacg tcctccagcg caaacccgat ctctggcgat

5761 gcgatgcact gcgacggtgt ctgctcgtcc aaggcaacat aggtgcgcag cacttcatgg

5821 cgggccacga tgcgatccaa cgcccgcccc agcgcagcag cctgcaggac gccgcgcaag

5881 cgcagcgcca tcggcatcag ataggccaga tcggcgtgcg ggtccagacg gccaaggaac

5941 cagaggcgtt gctgggcgaa cgacagcggt aaccggccac tgcgggcgac cggcacgatc

6001 ggcgccagca tcgcggcagc ggtgctgtcc atcgcccggg ccagttcggc cagacgcgga

6061 tgcgcgaaca gcgtggccac gggcacttcc acgccgagcc gtgtgcggat tctggcaatg

6121 agctggaccg ccagcagcga atgcccgccc agcgcgaaga aatcgtcgtg ccggccgatc

6181 tgcgcgacgc ccagcagttc ggtccagatc agtgcgaggg cttgttcgcg tccgggttcc

6241 ggcgcttgga agcactgcgt gccgaagtcc tggcgatcgg gcgcgggcag cgcacggcga

6301 tcgagtttgc cgttggcggt cagcggcagc gcatccaacg gcacgtaggc cgccggcacc

6361 atgtagtccg gcagcgatgt tcccaggtgt gcaggcaggg cctggcagac gtcggctgcg

6421 ccgaccacat aggcgaccaa tcgtttgtgg ccgtcggtgt cctgatcgat cacgaccacc

6481 gcatcgcgca cgccgttgca ggcgcgcact gcagcggcga tttcgcccag ttcgatgcgg

6541 aagccgcgca gcttgacctg atcgtcgcgg cgcccgagga agtccagcgt gccgtccgcg

6601 cgccaacgtg ccagatcgcc ggtcctgtac atgcgctggc cgggctgcgc ggcgaaagga

6661 tcggggagga acgtggctgc ggtgaggtcg gggcgaccga gataacccag cgcgagctgc

6721 gcgccgccga tatggagcgc gccttcgacg ccgatcggta ccggttggcc aaaacggtcg

6781 aggacgaaga cacggctatt ggccagcggc cggccgatcg gtactggcgg ctgcggatcg

6841 ctaccgctat gcagcgccag cgcggtcacg aactgggtgg cctcggtcgg accgtatgtc

6901 tgcagcagca cgtccggacc gggcgcctgc agcatcaagg catggctgtg cggatcggcc

6961 aggtcgccgc cggtgatcaa caggcgcagc gtgggtagct ggccggtcag catcggcgcg

7021 taggcgcgca gcacaccggc gaccaggatc agcaccgaga tctgcatctg tgcgatctgc

7081 gcgcacaacg cctgcggatc gcgcagaacc gatggtggga cgatcacgat cgccgcgccg

7141 ttgagcaggc tgctccacac ctccagcgtg gacgagtcga aggcgggatt ggaagcgaag

7201 gcgacccgat cggtgggcag gaccctggcc gcgccgggct ggctggcgaa attgacgatg

7261 gcgcggtgcg ggatcaccac gcccttgggc gtgccggtcg atccggacgt gtacatgaca

7321 taggcggcgc tatcgggcca taccgccacc gacggtgcat gggtggggcc ttctgcatcc

7381 aggcgatcca tgtccacacg cgtggcgctg ccattgggga cgtcgctggc cgagtggctg

7441 atcagcagac gcgcgccgca ctcgtcgagg atacggcgca tgcgctgggc cggatgttcg

7501 atgtccagcg gcacataggc tgcaccacat ttgaggatcg ccaattcggc aatgatcaga

7561 tccgcagaac gcgccagcgc gacagcgatg ctgctgcccg gcgtcgcgcc aagcgcgcgc

7621 aggcgatgcg ccatccggtt ggcctggctg tccaggtcgg cgtagctgag cgtgcgatgt

7681 tcgtccagaa ccgcgcaggc gttcggcgtc tgcctggcct ggtgttcgaa tcgctgatgg

7741 acgccatcta ccgcaggtcg cgcggcgtcg gtggcgttga aggcctggag cacctgcgtg

7801 cgttcgcgat cgtgcagacg cgtcaacgca tgcactggcc gcgtcggcga caccgtcagg

7861 gcctgggcca actgctgcag gatgtcgacc atcgtctgcg ccagcgtagc cggatccagg

7921 cgctggccgg tctgcacatc caccgagaat ccaccgctat cgtggtcgtc gttgattgcc

7981 agcgacagcg gatagtgcgt gcgctcgcgc aggtgcacgg tctcgatgcc ttgccagatc

8041 gtctcgctgc gcgcgcggtc gtcggccagc acgttgccgc cgccacggta gcgatagttg

8101 aacagcgcgg cgaacagcga cgctggcgcc tgaatcccac tgcagcgttg cgcttgggcc

8161 agcaacgcgt gctcgtgatg cagcagctgg gccagcaggc gctgggtctg cagcaccgct

8221 tgctcgacgc cggtatggtc gcgacgcagt cgcagcggca atgtattgag gaacatgccc

8281 agtacccggt cggcaccggc cgaggcgtgc atgcggccga acagcacggt gccgaacacc

8341 acgtcgtcgc ggccgctgat ctgcgacagc agcagcgcga acgccagatg gaacaggctg

8401 gcggtactga cccccaggcg acgcgcttgg atgcgtagcg cgtcggccag cgcgtcgggc

8461 aatcccaggt tggcttcctg caacgcgccc tcgacctggc ttaccccgaa cggcaaggtc

8521 ggttcggtga cgtcggccag catctgggtg aagaaggcac ggtgctcgtc ttcgctcacg

8581 ccaagacagg actgggcgac gaagtctcgg aacgggagcg gctggggtaa atggtgttgt

8641 tgcccgtcca gatgcgcttg cacttcctct accaccagtt ccagcgtggt gtggtccatc

8701 accaggtggt gatattgcag acccagcagc cagcattcgc gttcggcgtc atcgacgaca

8761 tgtgcgcgca gtaatggcgc ctgctgcagg tcgatgcggc gatgctgcgt ttccagacac

8821 tgctgcaagt gtgcggcgac atcgctgcca gcgaagcgat gggattgcac gggcaggggc

8881 gcatgtcggc ggacgacctg gactggctcg cgcaggcctt cccagaacac cgcggtgcgc

8941 aggatgtcgt ggcgtgcgat gacctgatcg agcgcagcga tgaagcgctc gcgcttgtcg

9001 caggcatcga aggcgatcac ggtgctgctc agatagatgt catccagcgg cgcggccacg

9061 tggtggtaga gcagtccatg ctgcaacggc gccaacggat agatatcctg aatgttgcca

9121 gcgccgcctt ccactcgttc gacgatgctg tcgatttccg tctgatcgag cgcgaccagc

9181 ggcaacagct cgggagtgat gtgggcgcag tcgcagacga tcgggttggc gggcacgttg

9241 accatggagg cggtcttgag cgcctcggcc aagccgagca acgtcggtgt gctgaacaag

9301 gcccgcacgt ccagctgcca gccgagcagg cgcaggcgct cgatcagttt gacgccgagc

9361 agcgagtgtc cgccgagcgc gaagaagctg tcgtggcggc cgacgcgttc gacgccgagc

9421 agctcgctcc acagctgtgc cagcatttgt tcgagcaggc tttgtggcgg gacgaaggcc

9481 gcgttaccca gatcgctttg ctctggggct ggcagagcgc gtcgatcgag tttgccgttg

9541 gctgtcagcg gcaaggactc cagccacaca taggcgctcg gcagcatga

LOCUS AFHK01000217 7068 bp DNA linear BCT 09-MAY-2011

DEFINITION Xanthomonas oryzae X11-5A Xo_X11-5A_contig_217, whole genome

shotgun sequence.

ACCESSION AFHK01000217 [AFHK01000000](http://www.ncbi.nlm.nih.gov/sites/entrez?db=Nucleotide&cmd=Search&term=AFHK01000000)

VERSION AFHK01000217.1 GI:332357166

DBLINK Project: [66097](http://www.ncbi.nlm.nih.gov/bioproject/66097)

KEYWORDS WGS.

SOURCE Xanthomonas oryzae X11-5A

ORGANISM [Xanthomonas oryzae X11-5A](http://www.ncbi.nlm.nih.gov/Taxonomy/Browser/wwwtax.cgi?id=1009853)

Bacteria; Proteobacteria; Gammaproteobacteria; Xanthomonadales;

Xanthomonadaceae; Xanthomonas.

REFERENCE 1 (bases 1 to 7068)

AUTHORS Triplett,L.R., Hamilton,J.P., Buell,C.R., Tisserat,N.A.,

Verdier,V., Zink,F. and Leach,J.E.

TITLE Genomic Analysis of Xanthomonas oryzae from US Rice Reveals

Substantial Divergence from Known X. oryzae Pathovars

JOURNAL Appl. Environ. Microbiol. (2011). 77(12):3930-7.

PUBMED [21515727](http://www.ncbi.nlm.nih.gov/pubmed/21515727)

COMMENT Base Quality-A custom perl script was used to clean and remove

reads with low complexity and low quality regions. Low quality

regions were defined as an average quality score of < 20 over a

10bp window along the read or > 2 'N' bases in the read. The Velvet parameters used in the final assembly for X11-5A were a hash length of 31, expected coverage of 19.58x, and a k-mer coverage cutoff of 9.79.

Contact Dr. Jan Leach for source DNA or cultures (Department of

BSPM, Colorado State University, Fort Collins, CO).

##Genome-Assembly-Data-START##

Assembly Method :: Velvet v. 0.7.53

Genome Coverage :: 70x

Sequencing Technology :: Illumina GAIIX

##Genome-Assembly-Data-END##

**Annotation was performed by Royer et al. (current manuscript)**

FEATURES Location/Qualifiers

source 1..7068

/organism="Xanthomonas oryzae X11-5A"

/mol_type="genomic DNA"

/strain="X11-5A"

/host="Oryza sativa cv. Lemont"

/db_xref="taxon:[1009853](http://www.ncbi.nlm.nih.gov/Taxonomy/Browser/wwwtax.cgi?id=1009853)"

/country="USA: Texas"

gene complement (<3..7067>)

/gene="NRPS"

CDS complement (<3..7067>)

/gene="NRPS"

/note= incomplete NRPS (no start codon, no stop codon)

/codon_start=1

/translation="AAAQAAAADDPAHAPALPPLHPQQLAYVIYTSGSTGQPKGVMVAHHGLGVRLHALIDTYRLGPQDRVLQFATLAFDASVEELFGALCSGAALVLRDDDWLDTERFWSRCAQAGASVVALPTRFWAQLCTAPLDIPACVRQVIVGGEALTAAMRQRWLHGPRIPLLDTYGPTEAIVAATAQAVAADAPNGIGRPLDATRAYVLDRTGELLPIGARGELHLGGAALARGYLGRPGLTAERFVPDPFATQPGERMYRTGDLARWRADGTLDYLGRNDEQVKIRGFRIALGEVAAALRACDGVRQAEVVVREDYGDTRLVAYLVVDHDGAFRAEALRTQLSARLPDYMLPAAYVPLDALPLTANGKLDRRALPAPDADAIATGVYAAPEGEREILLATLWHELLGVERVGRDDNFFALGGHSLLALRLIARLRTLLGLELTLAALFAQPRLADLAQAVARAAASALPAIVPTDRAQPLPLSFAQRRLWFLAQLDDRANQAYAMPGGVELRGALKLLALRKALDRIVARHDALRTTFVANGDSATQVIVTPEVGFALDYSDLRHAHDPDADAQRLAAQEANTPFDLEHGPLIRGRLLRLGEDRHRLLVSMHHLVSDGWSIDVLLRELGALYAAFVQGQPDPLPPLPIQYADYTLWQRRWLDGPRLQRQLEFWRDHLRDAPALLALPTDRPRPALQDYRGDSVDLVLGADLTQPLTALSQRRGTTLFMTVLAAWGTLLARLSSQDQVVIGTPIANRTCSALEPLIGFFVNTQAMRIDLRANPTVADLLAQVRSTVLVAQDHQDLPFEQVIEALNPARDLGHHPIFQAMLVWQNNAEVVLDLPGLQSRVLEQANPTAKFDLQLTLQVQDARIAGQITYATALFERDTIERQRAQFVALLHGLVADDHCRVDRLPLSSAIERQQWLHMLARQHTAFDDTRCLPAVFEQQVARTPQAIALVDGDSALSYAELDARANRLAHHLIAHGVGPEDRVALFLQRDSDLVVAVLAVLKAGAAYLPMDPAYPAERLAFMLDDAQPRALLAHRQLAAMLPTETRMAAVWLDDASVWAQQPAHAPQRSDLRPQHPAYVIYTSGSTGTPKGVVVAHAQVVRLLHATRSHVAPSAEDAWTLFHSCAFDFSVWELWGALAHGGRLVVVPQHIARDPAAFNALLCRHRVSVLNQTPSAFQALIEAQRHRDVQHHLRLVIFGGEALQPTNLAPWFARYGERTALLNMYGITETTVHVSVHAVTAQDVQRSGHSPIGTPLADLRAYVLGPDGQCLPVGVAGELHVAGAGLVRGYLGRPGLTAERFVPDPFAERPGERMYKTGDLARWQADGSLAYLGRNDEQVKVRGFRIELGDIAAALRDCDGVRQAAVIAREDSTGDKRLVAYVVGDTNVVLHAEGLRTQLGARLPDYMLPAAYVQLTALPLTANGKLDRRALPAPDADALAARAYVAPEGELETELATLWSALLGVERVGRHDDFFALGGHSLLAVRLISRIRQQLGVDVVLADLFAQPQLAALAARVACSQPSTLPPIVPVPRTQPPPMSFAQQRLWFLSQLDPAAGLAYLIDVGLHLHGALRADVLQQALDRIVARHASLRTRFFNSGDGTPVQHILPAGTVPLPYHDLSHQPDPDAALREHAHEEAAHPFDLTGESLIRGRLLRLAEHHHVLLLTLHHLVADGWSLRILVDELRTLYAAFVQGHADPLAPLTLHYADVAAWQRQWIDADTLQRQRAFWLAHLDGAPAQLSVPTDRQRPAIHDYQGASIQIEIDADLTAALKHLSGRHRTTLFMAVLAAWGVLLARLSHQDQAVIGSPSANRSRSELEPLIGLFVNTQALRIDLRDTPSVAELLAQVRGTVLAAQAHQDLPFEQVIEALNPQRSLAHHPVFQAMLAWQEQWDAPQDLPGLRVRPRDPGESSAKFDLQLTLQTQDARIVGQLTYATALFDRCTIERHLAQFVTLLHGMVANDNTRIDRLPLLRAPERQQWLQMLARTQRTFADDACLPSLFERHVAQRPRAIAVVHEELALSYGELDARANQLAHHLIAQGIGPEDRVALYLQRGIDLVVAILAVLKAGAAYLPLDPAYPPQRLAFMLADAQPRLLLAHAALAHTLPSENGTATVLLDDAAAWAHRPKHAPQRGDLLPQHPAYVIYTSGSTGTPKGVVVAHAQVVRLLHATHADVAPGADDVWTLFHSCAFDFSVWELWGALAYGGRLVVVPQHVARDPVAFHALLCQQRVSVLNQTPSAFQALIEAQRHSDVQHHLRLVIFGGEALQPATLAPWFAQHGQRTALLNMYGITETTVHVTAHALTEHDVTRADLSPIGTPLADLRAYVLGPDGQCLPIGVAGELHVAGAGLARGYL"

ORIGIN

1 agataaccgc gcgccagacc ggcgccggcc acatgcagtt cgccggccac accgatcggc

61 aggcattggc catctggacc cagcacatag gcacgcagat ccgcgagcgg tgtgccgatc

121 gggctgagat cggcgcgcgt gacatcgtgt tccgttagcg catgcgcagt cacgtgcacg

181 gtggtctcgg tgatgccata catgttgagc agcgcggtgc gctggccatg ttgtgcgaac

241 cacggagcca gcgtggccgg ttgcagcgct tcgcccccga agatcaccag ccgcaggtgg

301 tgctgcacgt cgctatgtcg ctgcgcctcg atcaaggcct gaaatgcgct gggcgtttga

361 ttgagcacgc tgacgcgctg ctggcagagc agggcatgga aggcgaccgg atcgcgcgcc

421 acgtgctgcg gcaccacgac cagcctgccg ccgtaagcga gggcgcccca cagttcccag

481 accgagaaat cgaaggcgca ggagtggaac agggtccaga catcgtcggc gcctggcgcg

541 acgtcggcgt gggtggcatg cagcaatcgc accacctggg catgcgcaac gaccacgccc

601 ttgggggtgc cggtagagcc agaggtgtag atgacgtagg ccggatgctg cggcagcagg

661 tcgccgcgct gcggggcgtg cttgggccga tgcgcccaag cagctgcatc gtccagcaac

721 acggtcgcgg tgccgttttc cgagggcagc gtgtgagcca gcgcagcatg ggcgagcagc

781 aaacgcggct gcgcgtcggc cagcatgaaa gccagccgct gggggggata ggccgggtcc

841 aagggtaggt aggcggcacc ggccttgagc acggcgagta tcgctacgac caagtcgatg

901 ccgcgctgca ggtacagcgc aacgcgatct tccggaccaa taccttgagc gatcagatga

961 tgggcgagct ggttggcgcg ggcatcgagt tcgccatagc tcagcgccaa ttcctcgtgg

1021 accacggcga tggcgcgcgg cctctgcgca acgtgtcgtt cgaacaaact cggcaggcaa

1081 gcatcgtcgg cgaatgtgcg ctgcgtgcgc gccagcatct gcagccactg ttggcgctcg

1141 ggcgcgcgca gcagcggcag cctgtcgatg cgcgtgttgt cgttggcgac catgccgtgc

1201 aacagcgtca cgaactgggc caggtggcgc tcgatggtgc agcggtcgaa cagcgcggtg

1261 gcataggtga gctggccaac aatgcgcgcg tcctgcgttt gcagcgtcag ttgcaggtcg

1321 aattttgcgc tggattcgcc ggggtcgcgc gggcgaactc gcaggcccgg cagatcctgc

1381 ggtgcatccc actgttcctg ccatgccagc atggcctgaa acaccgggtg atgggccagg

1441 ctgcgctgcg ggttgagtgc ttcgatgacc tgttcgaacg gcagatcctg gtgcgcttgc

1501 gccgccagga cggtgccgcg cacctgcgcc agcagctcgg cgaccgaggg ggtgtcgcgc

1561 agatcaatgc gcagtgcctg ggtgttgacg aacaggccaa tcagcggctc caactcgctg

1621 cggctgcggt tggcgctcgg actgccgatg acggcctgat cctgatgaga cagccgcgcc

1681 agcaaaacgc cccatgccgc cagaaccgcc atgaacaacg tcgtgcgatg acggccgctc

1741 aggtgtttca acgcagcagt aaggtcggcg tcgatctcta tctggatcga tgcaccctgg

1801 taatcatgga tggccggccg ttgtcgatcg gtcggcacgg aaagttgcgc cggcgcgcca

1861 tccagatgcg ccagccaaaa ggcacgctgc cgctgcaagg tgtcggcatc gatccattgc

1921 cgttgccatg ccgcgacatc ggcatagtga agcgtgagcg gagccaacgg gtcggcatgt

1981 ccctggacga atgcggcgta caaggtgcgc aattcgtcga caaggatgcg caaggaccaa

2041 ccgtcggcga cgagatggtg cagcgtgagc aacaagacgt ggtggtgctc ggccagacgc

2101 agcagacggc cgcggatcag cgattcgccc gtcagatcga aggggtgcgc tgcttcctca

2161 tgcgcgtgtt cgcgcagcgc agcgtcggga tcgggctggt gcgagagatc gtggtaaggc

2221 agcggaacgg tacctgcagg caagatatgc tgcacaggcg tgccgtcgcc gctgttgaaa

2281 aaacgtgtac gcaacgacgc gtggcgagcg acgatccggt ccagcgcttg ttgcaacaca

2341 tccgcgcgca atgcgccgtg caggtgcagt cccacatcga tgagataagc aagaccggct

2401 gcgggatcga gctggctcag gaaccacagg cgttgctgcg caaaggacat cggcggcggc

2461 tgggtacgcg ggaccggcac gatcggcggc agcgtactgg gttgcgaaca ggccacgcgc

2521 gcggccaacg ctgccagctg cggttgtgcg aataggtcgg ccaggacgac atccacgccc

2581 agttgctggc gaatccgcga gatcagtcgc accgccagca atgagtgtcc acccaaggcg

2641 aagaagtcgt cgtggcgacc gacgcgttcg acgccgagca gcgcgctcca cagcgtggcc

2701 agctcggttt ccagctcgcc ttccggtgcg acataggccc gcgcggcgag cgcatcggca

2761 tccggtgccg gcagagcgcg gcgatcgagt ttgccgttgg cagtcagcgg cagtgcggtg

2821 agttgtacat aggccgccgg cagcatgtag tcgggcaggc gcgcgcccag ctgggtgcgc

2881 agtccctctg cgtgcagaac aacgttggtg tcgccgacga cataggcgac cagacgtttg

2941 tcgccggtgc tgtcctcgcg ggcgatgacc gcggcctgac gcacgccgtc gcaatcacgc

3001 aatgcggcgg cgatgtcgcc cagctcgatg cggaagccgc gaaccttgac ctgttcgtcg

3061 ttgcggccga gatatgccag gctgccgtcg gcctgccagc gcgccagatc gccggtcttg

3121 tacatccgtt cacccggtcg ctcggcaaac gggtcgggca cgaagcgctc ggcagtcaat

3181 cccgggcggc ccagataacc gcgcaccaga ccggcgccgg ccacatgcag ttcgccggcc

3241 acaccgaccg gcaggcactg cccatccgga ccaagtacat aagcacgcag atccgcgagc

3301 ggtgtgccaa tcgggctgtg cccactgcgc tggacatcct gcgccgtcac cgcatgcacg

3361 ctcacatgca cggtggtttc ggtgatgcca tacatattga gcagcgcagt gcgctcgccg

3421 taccgggcga accaaggcgc caggttggtc ggctgcaggg cttcgccacc gaagatcacc

3481 aggcgcaggt ggtgttgcac gtcgcggtgt cgctgcgcct cgatcagtgc ctgaaatgcg

3541 cttggggttt gattgagcac gctgacgcgg tgccggcaga gcagggcatt gaaggcggcc

3601 ggatcgcgtg caatgtgctg tggcaccacg accaggcggc caccgtgggc gagcgcgccc

3661 cacaactccc agaccgagaa atcgaaggcg caggaatgga acagcgtcca cgcatcctca

3721 gcgctcggcg cgacgtggct gcgggtggca tgcaacaggc gcaccacctg ggcatgggcg

3781 acgaccacac ccttgggcgt gccggtggag ccagaggtgt agatgacgta agccggatgc

3841 tgcgggcgca gatcgctgcg ctgcggggcg tgggccggct gttgcgccca caccgacgcg

3901 tcatccagcc acacggcggc catgcgggtt tcggtgggca gcatggcggc cagttggcga

3961 tgggccagca gcgcgcgcgg ttgcgcatcg tcgagcatga aggccaggcg ttcggcggga

4021 taggccgggt ccatcggcag gtaggcggcg ccagccttga gtacggccag cacggcaacg

4081 accaggtcgc tgtcgcgctg caggaacagc gccacgcgat cttccgggcc gacgccgtgg

4141 gcgatcaggt gatgcgcgag ccggttggcg cgtgcgtcga gctcggcgta ggacagcgcg

4201 ctatcgccat cgaccagggc aatagcctgc ggggtgcggg cgacctgctg ttcgaacacc

4261 gcgggaaggc agcgggtatc gtcgaaggcc gtgtgctggc gggcgagcat gtgcagccac

4321 tgctggcgct cgatcgcgct cgacaagggc agccgatcca cgcggcaatg gtcatcggca

4381 acgaggccgt gcagcagcgc gacgaattgg gcccgctgcc gttcgatggt gtcgcgctcg

4441 aacagcgccg tggcataggt gatttgaccg gcgatgcgtg cgtcctgcac ctgcaaggtg

4501 agttgcagat cgaacttggc ggtggggttg gcctgttcca gcacgcggct ttgcaggccg

4561 ggcagatcca gcacgacctc cgcgttgttt tgccagacca gcatggcctg gaagatgggg

4621 tgatggccga gatcgcgcgc cgggttgagc gcttcgatga cctgctcgaa gggcaggtcc

4681 tgatgatcct gcgcgaccag gacggtgctg cggacctggg ccagcaggtc ggcgaccgtg

4741 ggattggcgc gcaggtcgat acgcatggcc tgggtgttga cgaaaaagcc gatcaacggt

4801 tccaacgcgc tgcaggtgcg gttggcgatc ggggtgccga tgaccacctg gtcctggctg

4861 gacaggcgcg ccagcagggt gccccaggca gccaatacgg tcatgaacag cgtggtgccg

4921 cggcgctgac tcaaggccgt caggggctgg gtgagatcgg caccgaggac cagatccaca

4981 ctgtcgccgc ggtagtcctg cagggcaggg cgcgggcgat ctgtgggcaa tgccagcagc

5041 gccggtgcgt cccgcagatg gtcgcgccag aactccagct ggcgttgcaa ccgcgggcca

5101 tcgagccagc ggcgctgcca cagggtgtag tcggcgtact ggatgggcag cggcggcagc

5161 ggatcgggct ggccctggac gaaggcggcg tacaaggcgc cgagttcgcg cagcaatacg

5221 tcgatcgacc agccatcgct gacgaggtga tgcatgctga ccagcaggcg atggcggtct

5281 tcgcccaggc gcagcaggcg gccgcggatc agcgggccgt gttccagatc gaagggcgtg

5341 ttggcttcct gcgcggccag gcgttgcgcg tctgcatcgg gatcgtgcgc atgacgcagg

5401 tcgctgtagt ccagcgcaaa gccgacctcg ggtgtgacga tgacctgcgt ggcgctgtcg

5461 ccgttggcca cgaaggtggt acgcaacgcg tcatgccgag cgacgatgcg atccagtgcc

5521 ttccgcagcg ccagcagctt gagcgcgccg cgcagttcca cgccgccggg catcgcgtag

5581 gcctggttgg cccggtcatc gagttgcgcc aggaaccaca ggcgccgctg ggcaaaagac

5641 agcggaagcg gttgggcgcg gtcggtcggc acgatggccg gcagtgcgct ggccgcggca

5701 cgggccacgg cctgggccag gtcggccagg cgcggttggg cgaacagcgc cgccaatgtc

5761 agttccaggc cgagcaaggt gcgcagacgc gcgatcagcc gcagcgccag cagcgaatgt

5821 ccgccgaggg cgaagaagtt atcgtcacgc ccgacgcgtt caacaccgag cagctcgtgc

5881 cacagcgtgg ccagcaggat ttcgcgttcg ccttcgggag ccgcatagac cccggtggca

5941 atcgcgtcgg catccggtgc gggcagtgcg cggcgatcga gcttgccgtt ggcggtcagc

6001 ggcagcgcat cgagcggcac atacgccgcg ggcagcatgt agtcgggtag acgtgcgctg

6061 agctgggtgc gtagtgcctc cgcgcggaag gcgccgtcgt ggtcgacaac cagataggcg

6121 accaggcgcg tgtcgccgta gtcctcgcgc acgaccacct cggcctggcg cacgccgtcg

6181 caggcgcgca gggcggcggc gacttcgccc agcgcgatac ggaagccgcg gatcttgacc

6241 tgctcgtcgt tgcggccgag gtaatccaac gtaccgtcgg cgcgccagcg ggccaggtcg

6301 ccggtgcggt acatgcgctc accgggttgc gtggcgaacg gatcggggac gaagcgttcg

6361 gcggtcaggc cgggacgacc gagatagcca cgggccagcg ccgcaccgcc cagatgcagt

6421 tcgccgcgcg ccccgattgg cagcagctca ccggtacggt ccagcacata ggcgcgcgtg

6481 gcgtcgagcg gacggccgat gccattgggc gcgtcggcgg ccacggcctg ggcggtcgcc

6541 gccacgatcg cttcggtggg cccataggtg tccagcaagg gaatgcgcgg gccgtgcagc

6601 cagcgctggc gcatggctgc cgtcagcgcc tcgccaccga cgatgacctg gcgcacgcaa

6661 gccgggatat ccagtggcgc cgtgcacagc tgtgcccaga aacgggtggg cagagcgacc

6721 acgctggcgc cggcctgcgc gcagcgtgac cagaagcgtt cggtatcgag ccaatcgtcg

6781 tcgcgcagca ccagggcggc accgctgcac agggcaccga agagttcttc gaccgaggca

6841 tcgaaggcca gcgtggcgaa ctgcagcacg cgatcctgcg gaccaagccg gtaggtgtcg

6901 atcagtgcgt gcaggcgcac gcccagcccg tggtgggcga ccatcacgcc cttgggctgg

6961 ccggtcgagc cggaggtgta gatcacatag gccaactgct gagggtgcag tggcggcaat

7021 gccggagcat gtgcggggtc gtcggcggcc gccgcttgtg cggcggcg

LOCUS AFHK01000229 6441 bp DNA linear BCT 09-MAY-2011

DEFINITION Xanthomonas oryzae X11-5A Xo_X11-5A_contig_229, whole genome

shotgun sequence.

ACCESSION AFHK01000229 [AFHK01000000](http://www.ncbi.nlm.nih.gov/sites/entrez?db=Nucleotide&cmd=Search&term=AFHK01000000)

VERSION AFHK01000229.1 GI:332357142

DBLINK Project: [66097](http://www.ncbi.nlm.nih.gov/bioproject/66097)

KEYWORDS WGS.

SOURCE Xanthomonas oryzae X11-5A

ORGANISM [Xanthomonas oryzae X11-5A](http://www.ncbi.nlm.nih.gov/Taxonomy/Browser/wwwtax.cgi?id=1009853)

Bacteria; Proteobacteria; Gammaproteobacteria; Xanthomonadales;

Xanthomonadaceae; Xanthomonas.

REFERENCE 1 (bases 1 to 6441)

AUTHORS Triplett,L.R., Hamilton,J.P., Buell,C.R., Tisserat,N.A.,

Verdier,V., Zink,F. and Leach,J.E.

TITLE Genomic Analysis of Xanthomonas oryzae from US Rice Reveals

Substantial Divergence from Known X. oryzae Pathovars

JOURNAL Appl. Environ. Microbiol. (2011). 77(12):3930-7.

PUBMED [21515727](http://www.ncbi.nlm.nih.gov/pubmed/21515727)

COMMENT Base Quality-A custom perl script was used to clean and remove

reads with low complexity and low quality regions. Low quality

regions were defined as an average quality score of < 20 over a

10bp window along the read or > 2 'N' bases in the read. The Velvet parameters used in the final assembly for X11-5A were a hash length of 31, expected coverage of 19.58x, and a k-mer coverage cutoff of 9.79.

Contact Dr. Jan Leach for source DNA or cultures (Department of

BSPM, Colorado State University, Fort Collins, CO).

##Genome-Assembly-Data-START##

Assembly Method :: Velvet v. 0.7.53

Genome Coverage :: 70x

Sequencing Technology :: Illumina GAIIX

##Genome-Assembly-Data-END##

**Annotation was performed by Royer et al. (current manuscript)**

FEATURES Location/Qualifiers

source 1..6441

/organism="Xanthomonas oryzae X11-5A"

/mol_type="genomic DNA"

/strain="X11-5A"

/host="Oryza sativa cv. Lemont"

/db_xref="taxon:[1009853](http://www.ncbi.nlm.nih.gov/Taxonomy/Browser/wwwtax.cgi?id=1009853)"

/country="USA: Texas"

gene (<1..6441>)

/gene="NRPS"

CDS (<1..6441>)

/gene="NRPS"

/note= incomplete NRPS (no start codon, no stop codon)

/codon_start=1

/translation="VKLRGFRIELGEIAAALRACDGVQDAAVLLREDTPGQPRLVAYLVGDAIETADTLRTQLATRLPEVMLPSAYVRLDALPLTTNGKLDRRALLAPDADTLAVQAYTPPQGELETLLADLWSELLGIERVGLHDNFFALGGHSLLAVRLISRIRSTLGIELPLATLFAQPCLTDLAQSLHGAAASTLPAIVPADRSALLPLSFAQQRLWFLAQLDARADLAYLMPNGLRLRGRLDRHALRMALDRLVARHETLRTRIGLHQDEPVQRIDADTVGFPLREHDLSGHPDPEAELRRLAEHETRTPFDLANDTLARGQLLRLGEDAHVLLITLHHLICDGWSMGLLVQELSVLYAAFAQHQPDPLPPLPLQYADIAVWQRRWIDGEVLQRQRNFWIEHLHDAPALLELPCDRPRPSVQDHRGEAMAFALNAQLSASLKISSQRHGSTVFMTLLAAWGVLLARLSGQEQVVIGTPIANRTRSELEPLIGLFVNTQALCIDLRADPSVAELLAQVRAIALAAQEHQDIPFEQVIEALNPARNLAHHPVFQAMFTWQNTSTSEHDLALPGLQLQSLSQSLSALKFDLELTLEERDGCIVGSLGYATALFDATTIQRWWRCFEQLLHALTRDDEVHVWQLPLLDAPQRQRLLADFGTGATATVPAQQLHGLFEAQARRTPEAIAVVSERRCLAYAALDAQANRLAQRLRDAGLRAGQHAAIALPRSATLIVAQLAVLKCGAAYVPLDVDHPSQRLLALIDDAQVSVLIHAADSVLAPAQVACLTIGDLDDLEGSDDDVTTSPAIAMPATAAAYLMYTSGSTGTPKGVTVSHGAVANLVLHDGPARLRSDDRVAFASNPAFDSATLEVWGGLLNGATVVVVPAAAMRDPQTLGMLLAHERLSVLILVAGVLRAYAPFIASQLSALRLLLTGGDVADPHALATVFAAGGAATVVQTYGPTESTQFVTALALQRAPDASRRVPIGRPLANTRLYVLDRHGEPVPIGVAGELHLAGAQLSQGYLHRPALTAERFLPDPFARIPGERMYRTGDLACWRDDGTLDFLGRNDAQVKIRGFRIEPGEIEAALRACAGVQEAVVVARDDTGEKRLVAYVVADRQAARAEPATLRSRLATRLPDHMLPAAYVQLDALPLTPNGKLDRTALPAPDDQALELHACVAPQGELECVLATLWSNLLGVEQVGRDDDFFALGGHSLLAVQLVSRVRQHLGVELPLAEVFAHPQMAQLASTLARAAPQTLPPIVPVPRDRPLPLSFAQQRLWFVAQLDPQAHLAYLMLLRLRIRGRLHAHALQDALNQLVARHEPLRTRIGVVDGVAVQEIAAATVGFPLEKIDLQGDGAQETQIQHHAELESTTAFEATDVSLVRGRLLRLANDEHVLLLTVHHLVSDGWSMELLTHELAALYVASVQKRPNPLPALPIQYADVAVWQRRWLAGERLQRQRAFWLEHLREAPALLDLPTDRPRPALQDYQGDAVELVVPVAVTAALNAVSQRHGSTLYMTVLAAWGVLLSRLSGQQQVVIGTPIANRTRSEFEPLIGLFVNTQALHIDLRGNPSVTELLAQVRATALAAQHNQDLPFEQVIEALNPDRNLAHHPVFQVTFAWQNTPTAIIELPDLALQAIQAARGAIKIDLELSLREIDECIVGSLRYAAALFDRSTVERHLGQFVQLLAGMANDTQLRVEQLPLLPADERAQLRHFTATETAPLAQAECIHHLFEAQARRTPEAIALCADHLELGYAALDARANRLAHRLCELGVGPEHRVALFLPRGIEQVVALLATLKAGAAYLPLDPELPDARLAFLLADSRPRAQLTCADLHARLQSLGSAVQGSSVLTLDTDIDADLHDPGAPMVPGLCPDNLAYVIYTSGSTGQPKGTLLTHAGATHYLQWAVDTYRPFPSAVVSSSLAFDATLTSLLAPLLCGARVELLPEHDTLNALRQRLCDPTPLGVVKLTPAHLELLGQQLADHPEPLGPKVMVIGGEALPAATLARWQALAPNTRLINEYGPTETVVGCVVHDATHDAPGESRRVPIGRPIAHLRVHVLDRHGQPAPIGIAGHLQIAGPQLARGYLARPDLTAERFIPDPFAEQPGQRMYRSGDLACWHADGTLDYLGRNDDQVKLRGFRIELGEIAAALRACDGVQD"

ORIGIN

1 gtcaaactgc gcggtttccg catcgagctc ggggaaatcg ccgctgcgtt gcgcgcctgc

61 gatggcgtgc aagacgccgc cgtgctgctg cgcgaagaca cgcccggcca gccgcgtctg

121 gttgcgtatc tggtcggcga tgccatcgaa actgccgaca ccttgcgtac gcaactggcc

181 acgcgcctgc ccgaggtcat gctgccaagc gcctatgtgc gcctcgacgc cttgccgctg

241 accaccaacg gcaagctgga tcgccgcgcg ctgctggcac ccgatgccga tacgctcgcc

301 gtccaggcct acacgccacc acaaggcgaa ctggaaaccc tgctggctga cctgtggagc

361 gagctgctcg gcatcgaacg cgtcggcctc cacgacaact tcttcgccct cggcggtcac

421 tcgctgctgg cggtgcggct gatctcgcgc atccgcagca ccctgggaat cgagttgccg

481 ttggcgacac tgttcgcaca gccgtgcttg accgatctgg cccaatcgct gcacggcgcc

541 gccgccagca ccttgccggc catcgtgccg gccgaccgca gcgcactgct gccgctgtcc

601 ttcgctcagc agcggctgtg gttcctggca cagctcgatg cgcgcgcaga cctggcctat

661 ttgatgccca atgggctgcg tctgcgcggt cggctcgatc gccacgcgct gcgcatggcg

721 ctggaccgcc ttgtcgcccg acatgaaacc ctgcgtaccc gcatcggcct gcaccaggac

781 gaaccggtgc agcgcatcga tgccgacact gtcggcttcc ctttgcgcga gcacgatctc

841 agcggtcatc cggatcccga agccgagctg cggcgtcttg ccgaacacga aacccgcacg

901 cccttcgacc tggccaacga caccttggcg cgcggtcaac tactgcgcct gggcgaagac

961 gcgcatgtcc tgctgatcac cctgcaccac ctgatctgcg atggctggtc catgggtttg

1021 ctcgtgcagg aactcagcgt cctctacgcc gccttcgcac agcaccagcc cgatccgtta

1081 ccgcccctgc cactgcaata cgccgatatc gccgtgtggc agcgccgctg gatcgatggc

1141 gaggtactgc aacgccagcg caacttctgg attgaacatc tgcatgatgc tcccgccctg

1201 ctggagctgc cctgcgatcg gccacggccg tcagtgcagg atcatcgcgg cgaagcgatg

1261 gcatttgcgt tgaatgctca actgagcgca tcgctgaaaa tctcgagcca gcgtcacggc

1321 agcactgtgt tcatgacctt gctcgcggcc tggggtgtct tgttggcacg cctgtcagga

1381 caagaacagg tagtgatcgg tacacctatc gccaatcgca cccgcagcga actggagccg

1441 ttgatcggac tgttcgtcaa cacccaggcg ttgtgcatcg acctgcgtgc agatccttcc

1501 gtcgccgaat tgctggctca ggtgcgtgcc attgcgctgg ccgcgcagga gcatcaggac

1561 attcccttcg agcaggtcat cgaagcgctc aacccggcac gcaacctcgc gcatcaccct

1621 gtgttccagg ccatgttcac ctggcagaac acctcgacca gcgagcacga cctggcgcta

1681 cccgggttgc agttgcagtc gttatcgcaa tccctgtccg cgctcaaatt cgatctggag

1741 ctgacgctgg aggaacgcga tggctgcatc gtcggcagtc ttggctacgc cacagcgctg

1801 ttcgatgcga ccacgatcca acgctggtgg cgctgcttcg agcaattgtt gcatgcgctg

1861 acgcgcgacg atgaggttca cgtctggcaa ttgccattgt tggatgcgcc gcaacgtcag

1921 cgtttgctgg ccgactttgg cactggcgcg accgccactg tgcccgcgca gcagctgcac

1981 gggttgtttg aagcgcaggc gcgccgcacg ccagaggcca ttgcggtggt gtcggagcga

2041 cgttgccttg cctacgccgc acttgatgcc caggccaatc gactggcgca gcgcttgcgc

2101 gacgcgggct tgcgtgccgg ccaacacgcg gcaattgcgc tgccgcgttc ggccacgctg

2161 atcgtcgcgc aactggccgt gctcaagtgc ggagcagcct atgtgccgct ggacgtcgac

2221 catcccagcc agcgcctgct tgcgctgatc gacgatgcgc aggtgtctgt gctcatccac

2281 gctgccgata gcgtgctggc gcctgcacag gtggcatgtc tgaccatcgg cgaccttgac

2341 gatctcgaag gcagcgacga cgacgtcaca acgtcgccgg cgatcgccat gccggccaca

2401 gctgctgcct acctcatgta tacctctggg tccaccggca cgcccaaggg tgtgacggtg

2461 tcgcatggag cagtcgccaa cctcgtcctg cacgatggtc cagcacgact gcggtccgac

2521 gaccgcgtcg ccttcgcctc caatcctgcc ttcgactcgg ccacgctgga ggtttggggc

2581 ggtctgctta atggcgctac cgtcgtcgtg gttcccgctg ctgccatgcg cgatccccag

2641 acactgggca tgttgctagc gcacgaacgc ctatcggtct tgatcctggt cgccggcgtg

2701 ctgcgcgcct atgcgccatt catcgcatcg cagctgagcg cgctacgttt gttgctcacc

2761 ggcggcgatg tcgccgatcc gcatgcactg gcaacggtgt ttgccgccgg tggcgcggcc

2821 accgttgtgc agacctacgg accgaccgag agcacgcaat tcgtgaccgc gctggcgttg

2881 cagcgtgcgc ccgatgcaag ccgacgcgtg ccgattggtc ggccgctggc caatacccgt

2941 ctctatgtgc tcgaccgcca tggtgagccg gttccgattg gcgtggccgg cgaactgcat

3001 ctggccggtg cgcagttatc gcagggctat ctgcatcgtc ccgcgttgac cgccgaacgc

3061 ttcctacctg atccgttcgc caggattccc ggcgagcgca tgtacaggac tggcgacctt

3121 gcctgttggc gtgacgacgg cacgctcgac ttcctcggcc gcaacgatgc gcaggtcaag

3181 atccgtggct tccgcatcga gccgggcgag atcgaagccg ccttgcgtgc ttgcgctggc

3241 gtgcaggaag ccgtggttgt cgcgcgcgac gacaccggcg aaaaacggtt ggtcgcgtat

3301 gtggtcgcag atcggcaagc cgctcgtgcg gagccggcga cgttacgcag ccgactcgcg

3361 acgcgcctgc ccgatcacat gctgccggcc gcttacgtgc aactcgatgc actgccgctg

3421 acccctaacg gcaaactcga tcgcaccgca ctcccggcac ccgacgatca ggcgctggaa

3481 ctgcatgcct gcgtcgcccc gcaaggcgaa ttggagtgcg tactggccac gttatggagc

3541 aacctccttg gcgtcgaaca ggtggggcgt gacgacgact tcttcgcact gggcggtcac

3601 tcgctgctgg cggtccaatt ggtctcacgt gtgcgtcaac acctcggcgt ggaactgccg

3661 ctggccgagg tattcgctca tccgcagatg gcgcaattgg ccagcacgct tgccagggcc

3721 gcgccgcaga ccctgccacc gatcgttccg gttccgcgcg accgtccgct tccactgtct

3781 tttgcccagc agcggttgtg gttcgtcgca cagctcgatc cgcaggctca cctggcctat

3841 ctgatgttgc tgcgtctgcg cattcgaggg cgactacatg cccacgccct gcaagatgcc

3901 ttgaaccagc ttgtcgcccg acatgagcct ttgcgcacac gcatcggtgt tgtcgatggc

3961 gtcgccgtgc aagagatcgc cgctgcaaca gtcggattcc cactggaaaa aatcgacctc

4021 cagggggatg gcgcgcagga aacgcagatt cagcatcacg ccgaactgga atccacgact

4081 gcattcgaag ccaccgatgt ctcgctcgtg cgaggacgcc tgttgcgcct ggccaatgac

4141 gagcatgtgc tgctgctcac cgtgcatcac ctggtatcgg atggctggtc gatggagctg

4201 ctgacgcacg agctggcagc gctgtacgtc gcatctgtgc agaaacggcc caatccactg

4261 ccggcactgc cgatccaata tgcagacgtt gcagtctggc aacgccgctg gctcgccggc

4321 gagcgactgc agcgccagcg cgccttttgg ctggagcatc tgcgcgaggc acccgcactg

4381 ctcgacctgc cgaccgatcg cccacgccct gcgctgcagg actaccaggg cgatgccgtg

4441 gaactggtcg tgccggtggc agtgacggcg gccttgaatg cggtgagcca acgccacggc

4501 agcacgttgt acatgaccgt gctggcggcc tggggcgtgc tgttgtcgcg cctgtccggc

4561 cagcaacagg tggtgatcgg cacacccatc gccaaccgta cccgtagcga attcgaacca

4621 ctgatcggcc tgttcgtcaa cacccaggcg ttgcacatcg atctgcgcgg caacccttcc

4681 gtcaccgagc tgttggctca ggtccgcgct accgcattgg ctgcccagca caatcaggac

4741 ctgcccttcg aacaagtcat cgaagcactc aatcccgacc gcaacctggc ccaccatccg

4801 gtcttccagg tcacgtttgc ctggcaaaac acgccgaccg cgatcatcga gttgcccgac

4861 ctggcactgc aggcgatcca ggctgcacgc ggagcaatca agatcgatct ggaactgtcg

4921 ctgcgcgaga tcgatgagtg catcgtcggc agcctgcgct atgcagcggc gttgttcgat

4981 cgcagcacgg tcgaacgtca tctgggtcag ttcgtccaat tgctggctgg catggctaac

5041 gacacgcaac tccgcgtcga gcaactgccg ttgctgcctg ccgacgaacg cgcacagctg

5101 cgccacttca ctgccaccga aacggctccg ctcgcgcaag cggagtgcat ccatcacctg

5161 ttcgaagcgc aggcacgtcg cacacccgaa gccatcgcgc tgtgcgccga tcacctcgaa

5221 ctcggctacg cggcgctgga tgcgcgcgcc aaccggttag cgcatcgcct atgcgaacta

5281 ggcgtcgggc cggaacatcg cgttgccctg ttcctgccgc gcggtatcga acaggtcgtc

5341 gcgctactgg ccacgctcaa ggccggcgct gcctatctgc cgctggaccc ggaactgccc

5401 gacgcacgcc tggccttcct gctcgcagac agccgcccgc gtgcgcagct gacctgtgcc

5461 gacctgcacg cgcggttgca gtctctcggc agtgccgtgc aaggcagcag cgtgctgacg

5521 ctggatacgg atattgatgc ggatctgcac gatcccggtg caccgatggt gcccggcctc

5581 tgcccggaca acctggccta cgtcatctac acctccggct ccaccgggca gcccaagggc

5641 acgctgctga cgcatgccgg cgccacgcat tacctgcagt gggccgtcga cacctatcgc

5701 ccgttcccga gcgccgtggt gtcttcctcg ctggccttcg acgccacgct gaccagcctg

5761 ctggctccgc tgctgtgtgg cgcaagagtc gaactgctgc ccgagcacga caccctcaac

5821 gcattacgcc agcgcctgtg cgatcccacc ccattaggcg tggtcaaact cactccagcc

5881 catctggaat tgctgggaca acagctcgca gatcatccgg aaccgcttgg cccgaaggtg

5941 atggtgatcg gtggcgaggc gctgcccgcc gcaactctcg cacgctggca agcgctcgca

6001 ccgaataccc gcctgatcaa cgaatacgga cccaccgaaa ccgtggtcgg ctgtgtggtg

6061 cacgacgcaa cgcatgatgc tcccggcgag agtcgtcgcg tgccgatcgg ccgcccaatc

6121 gcgcatctgc gcgtccatgt gctcgatcga cacggtcaac cggcaccgat cggcatcgcc

6181 ggccacctgc agattgccgg accgcaactc gcacgcggct acctcgctcg ccccgacctg

6241 accgccgaac gcttcatccc cgatcccttt gcagaacagc ccggtcaacg catgtaccgc

6301 agtggcgatc tggcctgctg gcatgccgat ggcacgttgg attatctcgg ccgcaacgac

6361 gaccaggtca aactgcgcgg cttccgcatc gagctcgggg aaatcgccgc cgcgttgcgc

6421 gcctgcgatg gcgtgcaaga c

LOCUS AFHK01000260 5151 bp DNA linear BCT 09-MAY-2011

DEFINITION Xanthomonas oryzae X11-5A Xo_X11-5A_contig_260, whole genome

shotgun sequence.

ACCESSION AFHK01000260 [AFHK01000000](http://www.ncbi.nlm.nih.gov/sites/entrez?db=Nucleotide&cmd=Search&term=AFHK01000000)

VERSION AFHK01000260.1 GI:332357080

DBLINK Project: [66097](http://www.ncbi.nlm.nih.gov/bioproject/66097)

KEYWORDS WGS.

SOURCE Xanthomonas oryzae X11-5A

ORGANISM [Xanthomonas oryzae X11-5A](http://www.ncbi.nlm.nih.gov/Taxonomy/Browser/wwwtax.cgi?id=1009853)

Bacteria; Proteobacteria; Gammaproteobacteria; Xanthomonadales;

Xanthomonadaceae; Xanthomonas.

REFERENCE 1 (bases 1 to 5151)

AUTHORS Triplett,L.R., Hamilton,J.P., Buell,C.R., Tisserat,N.A.,

Verdier,V., Zink,F. and Leach,J.E.

TITLE Genomic Analysis of Xanthomonas oryzae from US Rice Reveals

Substantial Divergence from Known X. oryzae Pathovars

JOURNAL Appl. Environ. Microbiol. (2011). 77(12):3930-7.

PUBMED [21515727](http://www.ncbi.nlm.nih.gov/pubmed/21515727)

COMMENT Base Quality-A custom perl script was used to clean and remove

reads with low complexity and low quality regions. Low quality

regions were defined as an average quality score of < 20 over a

10bp window along the read or > 2 'N' bases in the read. The Velvet parameters used in the final assembly for X11-5A were a hash length of 31, expected coverage of 19.58x, and a k-mer coverage cutoff of 9.79.

Contact Dr. Jan Leach for source DNA or cultures (Department of

BSPM, Colorado State University, Fort Collins, CO).

##Genome-Assembly-Data-START##

Assembly Method :: Velvet v. 0.7.53

Genome Coverage :: 70x

Sequencing Technology :: Illumina GAIIX

##Genome-Assembly-Data-END##

**Annotation was performed by Royer et al. (current manuscript)**

FEATURES Location/Qualifiers

source 1..5151

/organism="Xanthomonas oryzae X11-5A"

/mol_type="genomic DNA"

/strain="X11-5A"

/host="Oryza sativa cv. Lemont"

/db_xref="taxon:[1009853](http://www.ncbi.nlm.nih.gov/Taxonomy/Browser/wwwtax.cgi?id=1009853)"

/country="USA: Texas"

gene complement (<1..1356)

/gene="NRPS"

CDS complement (<1..1356)

/gene="NRPS"

/note= incomplete NRPS (no stop codon)

/codon_start=1

/translation="MSSPPALPAQLSTLSADEIQRLWTLLDDADATAADSDGIAPHAPGQPIPLSFAQQRLWLLAQLDPRSASAYLIPAGVRLHGVLQTQALQQALERIVARHAALRTHIVTVNGVAEQRIDPAGNGFALTYVDLSGQGLEEAEAEAQRHALQEATIAFDLAHGPLIRGRLLRLAAHDHVLLLTMHHIVSDGWSMGVLIHELGKLYTALAQGLDDPLPALSLQYADIVAWQRRQADGPALQRQRDFWRAHLRHAPTALSLPGDRPRPAVQDHAGDRVEIVLDTDLSSALAALSKRHGVTLFVTMLASWGSLLARQSGQDSVVIGSPVANRHRAEFEPVIGFFANTQALHVDLSGNPTVAALLAQLRALAAAAQAHQDVPFEQLVEMLNPVRDLSRHPLFQALLGWQEGAPETLALPGLRLEHFRADHHSAKFDLELSLRNTGRHIAGQLTYATALF"

gene complement (1366..5151>)

/gene="NRPS"

CDS complement (1366..5151>)

/gene="NRPS"

/note= incomplete NRPS (no start codon)

/codon_start=1

/translation="ALRACDGVQDAAVLLREDTPGQPRLVAYLVGDAINESTPVALREALATRLPEVMLPSAYLRLDALPLTTNGKLDRRALLAPDADALAVQTYTPPQGELQTLLATLWSELLEVEQVGCQDDFFALGGHSLLAISLIESLRQRGWQLDVPALFTHSTLLEMATQLRAGQVTVPPSRIVAGCTRITPDLLPLIELDQAEIDAIATAVNGGAANIQDIYPLAPLQAGMLFHHLANATADAYLQTSLMAFDTRDRVDRFLAALHWVVARHDILRTGLVWKELRSPVQVVWRQAPPRLHEHVFDENDDVAEALQAVLSSAGYRIDLQQAPLLHAHIAYDRTHTRWLVNLAYHHLVLDHTTLQGAMEEIDAHLSGRTHALPPPIPFRNFIFETGTKIAEAAHERFFTEMLGDVDGTTAPFGLSDVNGDGSRLRSLQLAVPSASALAIRRHARQLGISPAAIFHLAYALVLAVASGRDEVVFGSVLFGRLNGGAGADRAMGMFLNTLPLRLRRDATPLSQALRHTHARLAELVHHEHAPLALAQRCSALGASAPLFCALLNYRYNNRPDSGLHAQAGTFWEGVQGLHKRDVNNYPLTLSVNDGGDDFTLDIKLDHSVAAERVGALMLHCLAQMLQALTLRPHTPLHALPMLPAAERQALPHTASAASAPALPARCIHAAFQLQARRTPHATALICGEQMLSYRQLDQQAEQLAVTLHALGVRASTRVAIYLTRSIDMVIAWLATLKSGAAYVPLDPAYPADRLAYMLDDCRPRAVLTSAALEGQLPTCRAMHTARVLVLDAQQNDAPATSPPLALPTVSASELAYVIYTSGSSGRPKGVMIEHRQLDNLVRWHGERFGLQTGEHCTALAGLSFDAAAWEIWPALCHGACVRLAPAAASADPSRLLAWWSAQQAQLSFLPTPLAELALQQQQWPAGLRVLLTGGDRLGAVRQALPFALVNNYGLTETAVVATSGRVDVGAGLPSIGTPIDRLRAHVLDRWGHLVPLGAMGELHLAGPSLARGYLGRPALTAERFVPDPFAAQPGQRMYRTGDLVRWTQAHTLDFLGRNDQQVQVRGVRIEPGDVEAVLRNATGVREVAVVARQNQEGNILLVAYVTGRPLAIDVVRAHAIARLPDAMVPAAYVHLDALPLTPNGKLDRNALPAPDDRAFGVQPYAPPQGPVEERLADLWRELLGVERIGRYDNFFDIGGHSLLAVQLASRVRTHLHTEMPLDRFFANPQLHELAQHVLASRLERRAQADAQTLLARAKTGA"

STMSSPPALPAQLSTLSADEIQRLWTLLDDADATAADSDGIAPHAPGQPIPLSFAQQRLWLLAQLDPRSASAYLIPAGVRLHGVLQTQALQQALERIVARHAALRTHIVTVNGVAEQRIDPAGNGFALTYVDLSGQGLEEAEAEAQRHALQEATIAFDLAHGPLIRGRLLRLAAHDHVLLLTMHHIVSDGWSMGVLIHELGKLYTALAQGLDDPLPALSLQYADIVAWQRRQADGPALQRQRDFWRAHLRHAPTALSLPGDRPRPAVQDHAGDRVEIVLDTDLSSALAALSKRHGVTLFVTMLASWGSLLARQSGQDSVVIGSPVANRHRAEFEPVIGFFANTQALHVDLSGNPTVAALLAQLRALAAAAQAHQDVPFEQLVEMLNPVRDLSRHPLFQALLGWQEGAPETLALPGLRLEHFRADHHSAKFDLELSLRNTGRHIAGQLTYATALF"

ORIGIN

1 gaacagcgcg gtggcatagg tgagctggcc ggcgatgtgc ctgccggtat tgcgcaacga

61 cagttccagg tcgaacttcg cactgtgatg gtcggcgcgg aaatgttcca ggcgcagtcc

121 gggcagtgcc agcgtctccg gcgcaccctc ctgccacccc agcaacgcct ggaacagcgg

181 atggcgggaa aggtcgcgga ccggattgag catctcgaca agttgctcaa atggcacgtc

241 ctgatgcgcc tgtgccgctg cggcgagagc gcgcagttgg gcaagcaatg cggcgactgt

301 gggattgccg gaaagatcga catgcaacgc ctgggtattg gcaaaaaagc cgatcaccgg

361 ctcgaactct gcgcgatggc gattggcgac cgggctgccg atcaccacac tgtcctgtcc

421 ggactgccgc gccagcaaac tgccccagct cgccagcatg gtcacgaaca aggtcacgcc

481 atggcgcttg ctgagtgcgg cgagtgcgct gctcagatcg gtgtcgagca cgatctccac

541 ccgatcgccg gcgtgatcct gcaccgccgg gcgtggacga tcgccgggta ggctgagcgc

601 agtcggcgca tggcgaagat gcgcacgcca aaaatcgcgc tgccgctgaa gcgcgggacc

661 atcggcctgc cgccgttgcc aggccacgat gtcggcgtat tgcaacgaca atgccggtaa

721 cggatcgtcc agcccctgcg ccagggccgt gtacagcttg cccagctcgt ggatcagcac

781 gcccatcgac cagccatccg agacgatgtg gtgcatggtc aacagcagca catgatcgtg

841 tgcggccaga cgcagcaaac ggccgcggat cagcggaccg tgcgccaggt cgaaggcgat

901 ggtggcttcc tgcagtgcgt gccgctgcgc ctcggcctcg gcctcctcaa gaccttgccc

961 ggaaagatcg acgtacgtga gtgcgaagcc gttgccggcc ggatcgatcc gctgttcggc

1021 aacgccgttc accgtcacga tgtgggtacg caacgcggca tgccgggcga cgattcgttc

1081 cagcgcctgc tgcaaggcct gggtctgcaa cacgccgtgc aggcggaccc cggccggaat

1141 caggtacgcc gatgcgctgc gtggatccag ctgtgcaagt aaccacaacc gttgctgcgc

1201 gaacgacagc gggatcggct ggcctggagc gtgcggcgca atgccgtcgc tgtccgcagc

1261 ggtcgcatcg gcatcgtcca acagcgtcca caggcgctgg atttcatcgg cgctcagcgt

1321 ggacagttgt gcaggcagtg cggggggcga tgacatggtc gatcaagctc cagtttttgc

1381 gcgggcgagc agcgtctgtg catctgcctg cgcacgccgt tccaggcggc tggccagcac

1441 gtgttgcgca agttcatgca gctgtggatt agcgaagaaa cgatccagcg gcatctccgt

1501 atgcaggtgg gtccgtacgc gtgaggcaag ctgcacggcg agcagggaat gtccgccgat

1561 gtcgaagaag ttgtcgtacc gtccgatccg ctcgacgcca agcaactcgc gccagagatc

1621 ggccaggcgt tcctcgacag ggccttgtgg tggtgcgtag ggctgcacgc cgaacgctcg

1681 atcgtccggc gccggtagcg cgttccgatc cagcttgccg ttgggcgtca gcggcaacgc

1741 atccagatgc acatacgcgg ccgggaccat ggcgtcaggc agccgggcga tggcatgcgc

1801 ccgaacgaca tcgatggcga gcggtcgacc ggtcacatac gccaccagca ggatgttgcc

1861 ttcctggttc tgtcgcgcga ccaccgccac ttcgcgcacg ccggtcgcat tgcgcagcac

1921 ggcctcgacg tcgccgggct cgatacgcac gccgcgcacc tggacctgct gatcgttgcg

1981 accgaggaag tccagcgtat gcgcctgcgt ccagcgcacc agatcgccgg tgcgatacat

2041 gcgctggcca ggctgcgcag cgaatggatc ggggacaaaa cgttcggcgg tcagcgcggg

2101 gcgtcccaga tagccgcgcg ccaggctggg gccggccagg tgcaattcgc ccatggcacc

2161 gagcggcacc agatggcccc aacgatcgag cacatgggcg cgcaggcggt cgatgggcgt

2221 gccgatgctc ggcaatccgg cgccgacgtc gacgcggcca gacgtggcga ccaccgcggt

2281 ctcagtgagg ccgtagttgt tgaccagcgc gaacggcagc gcttgccgca ctgcgccgag

2341 ccggtcaccg ccggtcagca gcacgcgcag gcccgccggc cattgctgtt gttgcagcgc

2401 gagctcggcc aatggcgtgg gcagaaaact cagctgcgcc tgctgcgcgc tccaccaggc

2461 gagcaagcgc gacggatccg cgcttgctgc agcgggcgcc aggcgcacgc aggcaccgtg

2521 acagagcgcc ggccatatct cccaggcagc ggcgtcgaac gacagcccgg ccagcgcagt

2581 gcagtgttcg ccggtttgca gaccgaagcg ttcgccatgc catcggacca gattatctaa

2641 ttgccgatgt tcgatcatca cgcccttggg acgtccgctc gagccggagg tgtagatcac

2701 gtaggccagt tcgctggcgg acaccgttgg caaggccaag ggcggggagg ttgctggcgc

2761 atcgttctgt tgcgcgtcga gcacgagcac gcgtgcggta tgcatcgcgc ggcaggttgg

2821 cagctgtcct tcaagcgcgg cagaggtcag caccgcccgt ggccgacaat cgtcgagcat

2881 gtaggccaga cggtcggctg gataggcagg gtccagcggc acataggctg cgccgctctt

2941 caacgtggcc agccatgcga tcaccatgtc gatgctgcgc gtgaggtaaa tcgccacgcg

3001 cgtggaagct cggacgccca gcgcatgcaa ggtgacggcc agttgctcgg cctgttgatc

3061 caattgcctg taggacagca tctgctcgcc gcagatcagt gcggtggcat gcggcgtgcg

3121 gcgggcctgg agctggaatg ccgcatggat gcagcgcgct ggcaacgcgg gcgccgatgc

3181 tgcgctcgca gtatgcggca gtgcctgccg ttcggccgcg ggcagcatcg gcagcgcatg

3241 caacggcgtg tgcggacgca gcgtaagagc ctgcagcatt tgggcgagac aatgcagcat

3301 cagcgcgccc acgcgctcgg cggcaacgct gtgatccagc ttgatatcca gcgtgaagtc

3361 gtcgccgcca tcgttgaccg acaacgtcaa cggatagtta ttgacgtcgc gtttgtgcag

3421 gccctgcaca ccctcccaga acgtgcctgc ctgtgcgtgg agcccgctgt ctggacgatt

3481 gttgtagcga taattcagca gcgcgcagaa caatggcgcc gatgcaccaa gcgcgctgca

3541 ccgttgggcc agggccaacg gcgcatgctc gtggtggacc agctcggcca ggcgcgcatg

3601 ggtgtgccgc aatgcttgcg agagtggcgt ggcgtcgcgt cgtaagcgta atggcaaggt

3661 gttgagaaac atgcccatgg cgcggtcggc accggcgccg ccgttcaagc gcccgaacag

3721 cacgctgccg aacaccacct cgtcgcgccc actggcgacg gccagcacga gtgcataagc

3781 aaggtgaaag attgccgcag ggctgatgcc gagctggcgc gcatgccgac ggatcgctag

3841 cgccgacgcg ctcggaaccg ccaattgcag actacgtagc cgcgagccgt cgccgttgac

3901 gtcgctcaaa ccaaacggcg cggtggtgcc gtctacatcg ccaagcatct cggtaaagaa

3961 gcgttcgtgt gctgcctcgg cgatcttggt gccggtctcg aagatgaagt tgcggaatgg

4021 tattggcggc gggagtgcat gcgttcgccc gctaaggtgg gcgtcgattt cctccatcgc

4081 accctgcagc gtggtgtggt ccaacaccag gtggtgatag gccagattca ccaaccagcg

4141 tgtgtgggtg cggtcgtagg cgatgtgcgc atggagtaga ggagcctgtt gcaggtcgat

4201 gcgatagccc gctgaggaca acacggcttg caatgcctcc gccacatcgt cgttttcgtc

4261 gaagacatgc tcatgcaggc ggggaggcgc ctgtcgccag accacttgca ccgggctgcg

4321 cagctctttc cagaccaggc cagtgcgcaa gatgtcgtga cgtgcgacca cccagtgcag

4381 cgcagcgagg aagcgatcga cgcgatcgcg cgtgtcgaat gccatcaggc tggtctgcag

4441 ataggcgtct gcagtagcgt tggcgagatg gtgaaacagc atgccggcct gcaagggcgc

4501 cagtgggtag atgtcctgga tattggctgc gccaccgttc acagcggtag cgatggcatc

4561 gatctcggcc tggtcgagtt cgatgagcgg caagagatcg ggagtgatgc gggtacaacc

4621 ggcgacgatg cggctgggcg gaaccgtgac ctgaccggca cgcagttgcg ttgccatctc

4681 cagcagcgtg gagtgcgtga acagtgccgg tacatccagt tgccatccac gctggcgaag

4741 cgattcgatc aagctgatcg ccaacaacga gtggccgccg agggcgaaga agtcgtcttg

4801 gcagccgacc tgttccacct cgagcagctc gctccatagc gtggcgagca aggtttgcag

4861 ctcgccttgc ggaggcgtgt aggtctggac ggcgagcgca tcggcatcgg gtgccagcag

4921 cgcgcggcga tccagcttgc cgttggtggt cagcggcaag gcgtcgaggc gcagataggc

4981 gcttggcagc atgacctcgg gcaggcgcgt ggccaaggct tcgcgtagcg ccaccggggt

5041 gctctcgttg atggcatcgc cgaccagata cgcaaccaga cgcggctggc cgggcgtgtc

5101 ttcgcgcagc agcacggcgg cgtcttgcac gccatcgcag gcgcgcaacg c

LOCUS AFHK01000272 4876 bp DNA linear BCT 09-MAY-2011

DEFINITION Xanthomonas oryzae X11-5A Xo_X11-5A_contig_272, whole genome

shotgun sequence.

ACCESSION AFHK01000272 [AFHK01000000](http://www.ncbi.nlm.nih.gov/sites/entrez?db=Nucleotide&cmd=Search&term=AFHK01000000)

VERSION AFHK01000272.1 GI:332357056

DBLINK Project: [66097](http://www.ncbi.nlm.nih.gov/bioproject/66097)

KEYWORDS WGS.

SOURCE Xanthomonas oryzae X11-5A

ORGANISM [Xanthomonas oryzae X11-5A](http://www.ncbi.nlm.nih.gov/Taxonomy/Browser/wwwtax.cgi?id=1009853)

Bacteria; Proteobacteria; Gammaproteobacteria; Xanthomonadales;

Xanthomonadaceae; Xanthomonas.

REFERENCE 1 (bases 1 to 4876)

AUTHORS Triplett,L.R., Hamilton,J.P., Buell,C.R., Tisserat,N.A.,

Verdier,V., Zink,F. and Leach,J.E.

TITLE Genomic Analysis of Xanthomonas oryzae from US Rice Reveals

Substantial Divergence from Known X. oryzae Pathovars

JOURNAL Appl. Environ. Microbiol. (2011). 77(12):3930-7.

PUBMED [21515727](http://www.ncbi.nlm.nih.gov/pubmed/21515727)

COMMENT Base Quality-A custom perl script was used to clean and remove

reads with low complexity and low quality regions. Low quality

regions were defined as an average quality score of < 20 over a

10bp window along the read or > 2 'N' bases in the read. The Velvet parameters used in the final assembly for X11-5A were a hash length of 31, expected coverage of 19.58x, and a k-mer coverage cutoff of 9.79.

Contact Dr. Jan Leach for source DNA or cultures (Department of

BSPM, Colorado State University, Fort Collins, CO).

##Genome-Assembly-Data-START##

Assembly Method :: Velvet v. 0.7.53

Genome Coverage :: 70x

Sequencing Technology :: Illumina GAIIX

##Genome-Assembly-Data-END##

**Annotation was performed by Royer et al. (current manuscript)**

FEATURES Location/Qualifiers

source 1..4876

/organism="Xanthomonas oryzae X11-5A"

/mol_type="genomic DNA"

/strain="X11-5A"

/host="Oryza sativa cv. Lemont"

/db_xref="taxon:[1009853](http://www.ncbi.nlm.nih.gov/Taxonomy/Browser/wwwtax.cgi?id=1009853)"

/country="USA: Texas"

gene complement (<1..4875>)

/gene="NRPS"

CDS complement (<1..4875>)

/gene="NRPS"

/note= incomplete NRPS (no start codon, no stop codon)

/codon_start=1

/translation="DALAVQTYTPPEGELETLLATLWSELLGVERVGRHDSFFALGGHSLLGVRLISRIRSALGIELPLAALFAQPRPAELARALDSADTSILPAIVPTDRSAPLPLSFAQQRLWLLDRLDDRAALAYLIAGGVRLSGDLDRDALGKALDQLLIRHQSLRTVFSSNDDSPTQLVVAAGAGFALDCIDLRQSPDPAAEAQRHAEQETRTPFDLARGPLIRGRLLQLAAHEHRLLITMHHIVADGWSISVLLQELGALYTAFAQGKGDPLPALPIQYPDYAVWQRRWIDGPLLQRQLRFWREHLHGAPMLLELPTDRPRPAVQDYTGNSVDIALDADLTAALRSISKRHGTTVFMTLLAAWGALLARLSGQEQVVIGMPTANRTRSELEPLIGLFVNTQALRIDLRTDPSVAQLLSQVRTTALAAQQHPDVPFEQVIELLNPPRTLSHPPLFQVMFAWQNTPTVALELPDLRLESVQSPFPVSKFDLELTLQEDGARIVGSLGYATALFDATTIQRWWRCFEQLLRALTGDDAAHVSQLPWLDAPQRQQLLADFGTGAIAAVPAHALHQLFEAQARRTPDAVAVVSEQGCMHYAALDAQANRLAQRLRDVGLHAGQRVAIALPRSAGLIVAQLAALKCGAAYVPLDVAHPNERLLALIADAHVAVLIRAADSALAPAQVACLTIDDLHGSDAESATPPAIEVPATATAYVMYTSGSTGMPKGVTVSHEAVANLVLQDGPARLRSDDRVAFASNPAFDSATLEVWGSLLNGATVVVVPAPVMRDPQTLGALLTRERLSVLILVAGVLRAYAPAIAPQLGALRLLLTGGDVADPHALATVLDAGGPVTVLQTYGPTESTQFVTALALQDAPDPGQRVPIGRPLANTRLYVLDRQGQPTPIGVAGELHLAGAQLAQGYLHRPALTAERFVPDPFAAHPGERMYKTGDLARWRDDGTLDFLGRNDAQVKIRGFRIEPGEIEAALRSCDGVCEAVVVAREDTGEKRLVAYVVADQHATPAEPATLRSQLAARLPDHMLPAAYVQLDALPLTPNGKLDRAAFPAPDDQAMELHAYVAPQGELEQVLATLWSELLGVQQVGRHDDFFALGGHSLLAVKLIERLRRLGWQIDVRALFAQPTLAGLAANLQAASTIAVPPNRIGPDCDRITPDLLPLVALTQQEIETVVASVDGGATNVQDIYPLAPLQEGLLFHHLSDPLADPYLHSSVLGFPSRAVLDAFLDALDQVVARHDILRTGFVWHGLSAPVQVVWRTAVVPRHLQRVDGPDPAAHLQAQLHAPDAALCLQQAPLIHAHLAHDSATGRWLLGLQHHHLVMDHTTLELLIEEVRAHLAGRQRQLPSPLPFRDFVAHTLGAMSEQAHQAFFTAMLADVDTPTAAFGVHAPVAEPACLQELHQPLPHALAQTLRAQARQHGVSAASLFHLAYALVLARSSGSTEAVFATLLFGRMHASAGVDRVLGMFLNTLPIRLSAARGSVLDAVRHTQRCLAQLLHHEHAPLALAQRCSALDPSTPLLNALLNYRYAGGSAVLTDTQEDALQDVQQIGGQERTHYPLVVSVNDHIDEGGFSLDVQCVQDIGAERIATMLLHTLHVLAQALEQAPQSALHILDPLPDNERAQLH"

ORIGIN

1 gtgcaactgc gcgcgttcgt tatccggcag cggatccaga atatgcaacg cgctttgcgg

61 tgcctgctcc aacgcctggg ccagcacgtg cagggtgtgc agcagcatcg tggcgatgcg

121 ttcggcaccg atgtcctgca cgcattgcac gtcgagcgag aaaccgcctt cgtcaatgtg

181 gtcgttgacc gataccacca gcggatagtg ggtgcgctcc tggccaccga tctgctgcac

241 gtcctgcaac gcatcctctt gcgtatcggt caggacggcg ctgccgccgg catagcggta

301 attgagcaag gcgttgagca gcggggtgga tgggtccaac gcgctgcagc gctgcgccaa

361 tgcaagcggc gcatgttcgt ggtgcagcag ttgcgccagg cagcgctggg tgtggcgcac

421 ggcgtccagt acgctgccgc gcgcagcact taggcggatc ggcaaggtgt tgaggaacat

481 gccgagcacg cggtccacgc cggcgctggc atgcatgcgc ccgaacagca gggtggcgaa

541 caccgcctcg gtgctgccgc tgctacgggc cagcaccagc gcataggcca gatggaacag

601 gctggcggcg ctgacgccat gctggcgcgc ctgcgcgcgc agcgtctgcg caagtgcgtg

661 gggcagtggc tgatgcagct cctgcaggca cgcaggctcg gccaccggcg cgtgcacgcc

721 gaacgcggcg gtaggcgtgt cgacgtcggc cagcatggca gtgaagaagg cctggtgcgc

781 ctgctccgac atcgcaccga gggtatgggc gacgaagtcg cggaacggca atggcgaggg

841 caattgccgt tgccgaccgg ccagatgggc gcgcacttct tcgatcagca gttccagcgt

901 ggtgtggtcc atcaccaggt gatggtgttg caggccgagc agccagcggc cggttgcgct

961 gtcgtgggcc agatgggcgt ggatcagcgg cgcctgctgc aggcagagcg cggcgtcggg

1021 agcgtgcaac tgggcctgca agtgcgcggc aggatcggga ccgtcgacgc gctgcaggtg

1081 acgcgggaca accgcggttc gccagaccac ttgcaccggt gcagacagcc catgccagac

1141 gaacccggtg cgcaggatgt cgtggcgggc gacgacctga tcgagcgcat cgagaaaggc

1201 atcgagcact gcgcgcgacg ggaagccgag caccgacgaa tgcagatagg gatcggcgag

1261 cggatcggac agatggtgaa acagcaggcc ttcctgcaat ggtgccagcg gatagatgtc

1321 ctgcacgttg gttgcgccgc cgtcgacgct ggccaccacc gtctcgatct cctgctgggt

1381 caacgcgacc aatggcagca ggtccggcgt gatgcggtcg caatcgggac cgatgcggtt

1441 ggggggcacg gcgatggtgg atgcggcctg cagattggcg gccaggcccg cgagcgtggg

1501 ttgggcaaat agcgcgcgca catcgatctg ccagccgagc cggcgcagac gttcgatcag

1561 tttgactgcc aacagcgagt ggccgccgag ggcgaagaag tcgtcgtggc gtccgacctg

1621 ttgcacgccg agcagttcgc tccacagcgt ggccagcacc tgttcgagct cgccttgggg

1681 ggcgacatag gcatgcagct ccatcgcctg atcgtccggt gccgggaacg cggcgcgatc

1741 gagtttgccg ttgggggtga gcggcaatgc atcgagctgc acatacgctg cgggcagcat

1801 gtggtcgggc aggcgtgcgg cgagttggct gcgcagcgtt gccggttccg caggcgtggc

1861 gtgctgatcg gcgaccacat acgcgaccag acgtttttca ccggtgtcct cgcgcgcgac

1921 aactaccgct tcacacacac catcgcagct gcgcagtgcc gcttcgatct cgcccggctc

1981 gatgcggaag ccacggatct tgacctgcgc atcgttgcgg ccgaggaagt cgagcgtgcc

2041 gtcgtcgcgc cagcgcgcca ggtcgccggt cttgtacatg cgctcgccgg ggtgtgcggc

2101 gaacggatca ggcacgaagc gttcggcagt cagggcaggg cggtgcagat acccttgtgc

2161 cagttgtgca ccggcgagat gcagttcgcc ggccacgccg atcggcgtcg gctggccctg

2221 gcgatcgagc acatagagcc gggtgttggc cagcggccgc ccgatcggca cacgctggcc

2281 tggatcgggc gcatcttgca aggccagtgc ggtgacgaac tgggtgctct cggtcggtcc

2341 gtaggtctgc agcacggtga caggacctcc tgcgtccaac acagtcgcta gtgcatgcgg

2401 atcggcaaca tcgccgccgg taagcagcag acgcagcgcg cccaattgcg gcgcgatcgc

2461 tggcgcatag gcgcgcagga caccagcgac caggatcagc accgacaggc gttcgcgtgt

2521 cagcaacgcg cccagcgtct gcgggtcgcg cattaccggt gcaggcacga cgacgacggt

2581 ggcgccgttg agcaggctgc cccagacctc cagcgtggcc gagtcgaagg caggattgga

2641 agcgaaagcg acgcggtcat cggagcgcag ccgtgctggc ccatcctgca ggaccaggtt

2701 cgccaccgcc tcatgcgaca ccgtcacccc cttcggcatg ccggtggaac cggaggtgta

2761 catcacgtag gccgtcgctg tggcaggcac ctcgatcgcc ggcggcgttg cactctcggc

2821 gtcgctgccg tggaggtcgt cgatcgtcag gcacgccacc tgcgcaggtg ccagcgcact

2881 gtcggcggcg cggatcaaca ccgccacgtg cgcatcggca atcagcgcaa gcaagcgctc

2941 gttgggatgg gcgacgtcca gcggcacata ggccgccccg cacttgagcg cggccagttg

3001 cgccacgatc aatccggccg agcgtggcag cgcgatcgcc acgcgttggc cggcatgcaa

3061 gcccacatcg cgcaagcgct gcgccagtcg gttggcctgc gcatcgagcg cggcgtaatg

3121 catgcaacct tgctccgaca ccactgcaac ggcatccggc gtgcggcgtg cctgcgcttc

3181 gaacagctgg tgcagcgcgt gtgcgggcac ggcggcaatc gcgccggtgc cgaagtcggc

3241 cagcaattgc tgccgctgtg gcgcatccag ccacggcaac tgcgagacat gggcagcgtc

3301 gtcgccggtc agtgcccgca gcagctgctc gaagcagcgc caccagcgtt ggatcgtggt

3361 cgcatcgaac agcgccgtgg cgtagcccag gctgccgacg atgcgtgcgc cgtcttcctg

3421 cagcgtcagc tcaagatcga acttgctgac ggggaatgga ctctgaacgc tctcaagacg

3481 caggtcgggc aattccagcg cgacggttgg cgtgttctgc caggcgaaca tcacctggaa

3541 tagcggcggg tggctcaagg tgcgcggcgg attgagcagc tcgatgacct gttcgaacgg

3601 aacatccggg tgttgctgcg cagccaacgc ggtggtgcgg acctggctta gcaactgggc

3661 cacgctcgga tctgtacgca gatcgatgcg cagcgcctga gtgttgacga acaggccgat

3721 cagcggttcc agttcgctgc gcgtgcggtt cgcggtcggc atgccgatga cgacctgctc

3781 ctggccggac aggcgcgcca gcagcgcgcc ccaggcggcg agcaaggtca tgaaaacggt

3841 agtgccgtga cgcttgctga tgcttcgcag cgctgcggtg agatctgcat ccagcgcaat

3901 atcgacacta ttgccggtgt aatcctgcac cgccggacgc gggcgatcgg tgggcaactc

3961 cagtagcatc ggtgcgccgt gcagatgctc acgccagaag cgcagctggc gttgcagcaa

4021 tggaccatcg atccagcggc gctgccacac ggcgtagtcc ggatactgga tcggcagcgc

4081 cggcagcgga tcgcccttgc cttgtgcaaa agcagtgtaa agggctccga gttcctgcaa

4141 caggacgctg atggaccaac catcggcgac gatgtgatgc atggtgatca acagacgatg

4201 ctcgtgcgca gccagttgca gcaggcgacc gcgaatcagc gggccgcgcg ccagatcgaa

4261 gggcgtgcgg gtttcctgtt cggcgtggcg ctgggcctcg gcggcgggat ccggcgactg

4321 gcgcagatcg atgcagtcca gggcaaagcc ggcgcccgct gcgaccacaa gctgggtcgg

4381 gctgtcgtcg tttgaagaaa aaactgtccg cagcgattga tgacggatca acagttgatc

4441 aagcgccttg cccagtgcat cgcgatccag gtcgccggac agacggactc caccggcgat

4501 caggtacgcc agtgccgcac gatcatcgag ccggtctagc aaccacaggc gttgctgggc

4561 gaaggacagc ggcagcggtg cactgcggtc ggtcgggacg atggcgggca ggatgctggt

4621 gtcggcgctg tccagcgctc gggccagttc ggccgggcgc ggttgtgcga acaacgcggc

4681 cagcggcagt tcgataccaa gcgcgctgcg gatgcgcgag atcagtcgca caccgagcag

4741 cgagtgcccg ccgagcgcga agaagctgtc gtggcggccg acgcgttcga cgccgagcag

4801 ctcgctccac agggtggcca gcaaggtttc caactcgcct tccggtggcg tgtaggtctg

4861 gacggcgagc gcatcg

LOCUS AFHK01000334 3134 bp DNA linear BCT 09-MAY-2011

DEFINITION Xanthomonas oryzae X11-5A Xo_X11-5A_contig_334, whole genome

shotgun sequence.

ACCESSION AFHK01000334 [AFHK01000000](http://www.ncbi.nlm.nih.gov/sites/entrez?db=Nucleotide&cmd=Search&term=AFHK01000000)

VERSION AFHK01000334.1 GI:332356932

DBLINK Project: [66097](http://www.ncbi.nlm.nih.gov/bioproject/66097)

KEYWORDS WGS.

SOURCE Xanthomonas oryzae X11-5A

ORGANISM [Xanthomonas oryzae X11-5A](http://www.ncbi.nlm.nih.gov/Taxonomy/Browser/wwwtax.cgi?id=1009853)

Bacteria; Proteobacteria; Gammaproteobacteria; Xanthomonadales;

Xanthomonadaceae; Xanthomonas.

REFERENCE 1 (bases 1 to 3134)

AUTHORS Triplett,L.R., Hamilton,J.P., Buell,C.R., Tisserat,N.A.,

Verdier,V., Zink,F. and Leach,J.E.

TITLE Genomic Analysis of Xanthomonas oryzae from US Rice Reveals

Substantial Divergence from Known X. oryzae Pathovars

JOURNAL Appl. Environ. Microbiol. (2011). 77(12):3930-7.

PUBMED [21515727](http://www.ncbi.nlm.nih.gov/pubmed/21515727)

COMMENT Base Quality-A custom perl script was used to clean and remove

reads with low complexity and low quality regions. Low quality

regions were defined as an average quality score of < 20 over a

10bp window along the read or > 2 'N' bases in the read. The Velvet parameters used in the final assembly for X11-5A were a hash length of 31, expected coverage of 19.58x, and a k-mer coverage cutoff of 9.79.

Contact Dr. Jan Leach for source DNA or cultures (Department of

BSPM, Colorado State University, Fort Collins, CO).

##Genome-Assembly-Data-START##

Assembly Method :: Velvet v. 0.7.53

Genome Coverage :: 70x

Sequencing Technology :: Illumina GAIIX

##Genome-Assembly-Data-END##

**Annotation was performed by Royer et al. (current manuscript)**

FEATURES Location/Qualifiers

source 1..3134

/organism="Xanthomonas oryzae X11-5A"

/mol_type="genomic DNA"

/strain="X11-5A"

/host="Oryza sativa cv. Lemont"

/db_xref="taxon:[1009853](http://www.ncbi.nlm.nih.gov/Taxonomy/Browser/wwwtax.cgi?id=1009853)"

/country="USA: Texas"

gene (<2..3133>)

/gene="NRPS"

CDS (<2..3133>)

/gene="NRPS"

/note= incomplete NRPS (no start codon, no stop codon)

/codon_start=1

/translation="SLLAVRLISRMRSTLGIELPLATLFAQPRLAELAQSLHGAAASTLPAIVPADRSAALPLSFAQQRLWFLAQLDPQAGLAYLMPNGLRLHGRLDRRALRLALNRIVARHETLRTRIALHQDEPVQRIDADDVGLLLREHDLSGHPDPDAELLRLAEHETRTPFDLAHDTLARGRLLRLGEDAHVLLVTLHHLVADGWSMGVLVHELGTLYTAFAQGQPDPLPPLPIQYADYSLWQRRWLAGPLLQRQLDFWRDHLQDAPALLELPTDRPRPARQDARGDTIECVLDAELSAALTVLSQRHGSTVFMTLLAAWGVLLARLSGQEQVVIGTPIANRTRSELEPLIGLFVNTQALCIDLRGEPSFGDLLGQVRTTALAAQAHQDVPFEQVIEALNPARNLAHHPLFQVMFAWQNTPASSIELPELALHAVPQRLNTLKFDLDLALQARDGAIVGRLGYATALFDAATIQRWWGCFEQLLHALTRAEDTCIWQLPWLNAPQRQHLLAAFGTGATAAVPDQPVHRLFETQAQRTPDAIAVVANQQCVSYAALDARANQLAHHLVALGVAPESVVAVCLPRGIDLIVALLAILKAGAAYLPLDINAPPARLDAMLVAARAPVLLAHRQIATQLAAREDPRWVLIDVDAASWASAPAHAPSAAAWHPQHPAYVIYTSGSTGQAKGVVISHHALVNFLAALHAQLPLSPQDRLLAVTTICFDIAALELFAPLIHGACVVIASTQIQEPTQWMQLLADERISVLQATPTFWQMLLNAGWQSTPTLRLLCGGEALPQDLAQRLQAGGGRLWNLYGPTEATIWASAHRVLGNARGSVVSLGRPLSNTRMHVLDAHRQLLPQGVTGELCIAGPQLARGYLGRPDLTAERFVPDPFAAQPGQRMYLSGDLACWRADGNLEYLGRSDDQIKLRGFRIEPGEIESALRRCEGVREAVVVVHTHGSDTRLVAYLVGTKVLNAERLRAALAARLPDYMIPGAYVQLDALPLTPNGKLDRRALPEPDANALAAQAYAPPESALEILLSTLWSELLGIERVGRH"

ORIGIN

1 ctcgctgctg gcggtgcggc tgatctcgcg gatgcgcagc actctgggaa tcgagctgcc

61 attggcaacc ctgttcgcac agccgcgcct tgccgagttg gcgcaatcgc tgcacggcgc

121 cgccgccagc accttgccgg ccatcgtgcc ggccgaccgc agcgcagcgt tgccgctgtc

181 cttcgcccag caacggctgt ggttcctcgc ccagctcgat ccgcaagcgg gtctggccta

241 cctcatgccc aacggcctgc gcctgcacgg ccggctggac cgccgcgccc tgcgcctggc

301 gctgaaccgc atcgtcgccc gccatgaaac cttgcgcacc cgcatcgccc tgcaccagga

361 cgaaccggta cagcgcatcg atgccgacga tgtcggcctg ctcctgcgcg aacacgatct

421 cagcggtcat ccagaccccg acgccgagct gctgcgtttg gccgaacacg agacccgcac

481 gcccttcgac ctggcccacg acaccctggc gcgcggtcga ctgctgcgcc tgggcgagga

541 cgcgcatgtc ctgctcgtca ccctgcacca cctggtggcc gacggctggt cgatgggcgt

601 gctggtgcat gaactgggca cgctctacac cgccttcgcg caaggccagc ccgatccgct

661 gccgccattg ccgatccagt acgccgacta cagcctgtgg caacgccgct ggcttgcggg

721 gccgctgctg caacgccagc tcgacttctg gcgcgaccac ctgcaggacg cgcccgctct

781 gctggaattg cccaccgacc gaccgcgccc ggcgcgacag gacgcgcgcg gcgacacgat

841 cgagtgcgtg ctcgatgccg agctcagcgc tgcgttgacg gtgctgagcc aacgccatgg

901 cagcaccgtg ttcatgacct tgctcgcggc ctggggtgtc ttgttggcac gcctgtcagg

961 acaagaacag gtggtgatcg gtacgcccat cgccaaccgc acccgcagcg aactggagcc

1021 gttgatcggc ctgttcgtca acacccaggc gttgtgcatc gatctgcggg gcgagccttc

1081 gttcggcgac ctgctgggcc aggtgcgcac gaccgcgctg gccgcgcagg cacaccagga

1141 cgtccccttc gagcaggtga tcgaagcgct caacccggcc cgcaacctgg cccatcaccc

1201 gctgttccag gtcatgttcg cctggcagaa cacgcctgcc agcagcatcg aactaccgga

1261 actggcactg cacgccgtgc cgcagcgcct gaacacactc aagttcgacc tggatctggc

1321 gctgcaagcg cgcgacggtg ccatcgtcgg cagactcggc tatgccacgg cactgttcga

1381 tgcggccacc atccaacgct ggtggggttg cttcgaacag ttgctgcatg cactgacacg

1441 cgcggaagac acctgcatct ggcaactgcc ctggctgaat gcgccgcaac ggcaacacct

1501 gctggcggcg ttcggcacgg gagcgaccgc cgcggtgccg gaccagcccg tgcaccggct

1561 gttcgaaacg caagcgcagc gcacgcccga tgccatcgcc gtggtcgcca accagcagtg

1621 cgtcagttat gccgcgctcg atgcccgcgc caaccaactg gcccaccatc tggttgcgct

1681 gggcgtagcg ccggaaagcg tcgtggccgt gtgcctgcca cgcggcatcg acctgatcgt

1741 ggctttgctg gcgatcctca aggccggcgc ggcttatcta ccgctggaca tcaatgcgcc

1801 gcccgcgcgt ctggacgcga tgcttgtcgc cgcgcgcgcg ccggtgttgc tcgcgcaccg

1861 gcagatcgcc acgcagctgg cagcgcgtga agatccgcgt tgggtgttga ttgatgtcga

1921 tgcggcgtcg tgggcatccg ctcccgcgca cgcgcccagc gcggcggcat ggcatccgca

1981 acacccggcc tacgtcatct acacctccgg ctccacgggc caagccaagg gggtggtgat

2041 ctcccatcac gccctggtga acttcctcgc tgccctgcac gcccagctgc cgctgtcgcc

2101 gcaggaccgc ctgttggccg tcacgaccat ctgcttcgac atcgccgcgc tggaactgtt

2161 cgcaccgctg atccatggcg cctgcgtcgt catcgcttca acgcagatcc aggagccgac

2221 gcagtggatg cagctgctgg ccgacgagcg tatttcggtg ctgcaagcga cgccgacgtt

2281 ctggcagatg ctgctcaatg ccggttggca gagcacgccg accctacgcc tgctctgcgg

2341 tggcgaagcc ttgccgcagg atctggcgca acgcctgcaa gcgggcggcg gccggctgtg

2401 gaatctgtac ggccccaccg aggccaccat ttgggcgagc gcacaccgcg tactcggcaa

2461 cgcgcgtggc agcgtcgtat cgctggggcg gcccctgtcc aatacccgca tgcatgtgct

2521 cgacgcgcat cggcagttgc tgccgcaggg cgtgaccgga gaactgtgca tcgccggccc

2581 gcagctggcc cgtggttatc tcggtcgccc tgacctgacc gccgagcgct tcgtgcccga

2641 tcccttcgcc gcgcagccgg gccagcgcat gtaccttagc ggcgatctgg cgtgctggcg

2701 cgccgacggc aacctggagt atctgggccg cagcgacgat cagatcaagc tgcgcggctt

2761 ccgcattgaa cccggcgaga tcgaatcagc attgcgcaga tgcgagggcg tgcgcgaggc

2821 agtggtcgtg gtgcacacgc acggcagcga cacccggctg gtcgcctatc tggtcggcac

2881 caaggtgttg aacgccgagc gcctgcgtgc cgcgttggcc gcgcgtctgc ccgattacat

2941 gatcccgggc gcctatgtgc aactggatgc gctgccgctg acgcccaatg gcaaactcga

3001 ccgtcgcgcc cttcccgaac ccgacgcgaa cgcgcttgcc gcgcaggcct acgcgccacc

3061 ggaaagcgcg ttggaaatac tgctgtccac actgtggagc gaactgctcg gcatcgaacg

3121 cgtcggccgc cacg
